# HomoTherm: An Open‐Source Approach to Modelling Heat Exchange in Humans and Other Hominins in Diverse Environments

**DOI:** 10.1111/gcb.70830

**Published:** 2026-04-01

**Authors:** Michael R. Kearney, Duncan Mitchell, Shane K. Maloney

**Affiliations:** ^1^ School of BioSciences The University of Melbourne Melbourne Victoria Australia; ^2^ Brain Function Research Group, School of Physiology The University of the Witwatersrand Johannesburg South Africa; ^3^ School of Human Sciences The University of Western Australia Perth Western Australia Australia

**Keywords:** climate change, dehydration, heat budget, hominin, human, hyperthermia, microclimate

## Abstract

Climate change is increasing human exposure to novel environments and generating serious practical challenges in human health, while the role of past climates in shaping hominin evolution remains a fascination. Models of human heat exchange vary from simple environmental indices to extremely detailed physiological calculations. Physiologically explicit models vary in their physical explicitness, and significant trade‐offs exist between model realism and computational speed. We presently lack agile models that are not so over‐parameterised that they are slow, that maintain generality across diverse and complex physical environments, and that have sufficient biological realism to capture human (and, more generally, hominin) diversity in physiology, body size and proportions, activity level, clothing, hair, and skin. Here we present a model (HomoTherm) to fill this gap, built upon the general endotherm model from the NicheMapR package for biophysical modelling in R. This steady‐state heat budget model is unique among existing open‐source models in that it solves for the metabolic rate, evaporation rate, skin temperature, and clothing temperature of a multi‐part (head, torso, and limbs) human of variable shape, size, or colour and at variable levels of physical activity. The model can handle complex microclimates, including environments that differ in downwelling and upwelling radiation, and connects directly with the microclimate modelling capabilities of NicheMapR. Because of its general nature, it can easily be adapted to model other hominins by changing proportions, fur properties, and any other known physiological trait. We illustrate the model's capabilities through comparisons with actual human responses in published empirical studies of indoor and outdoor exposures to heat and cold, and compare HomoTherm's performance with that of other models. Vignettes and an open‐source Shiny app facilitate the use of HomoTherm in addressing fundamental and applied problems in human thermal biology.

## Graphical abstract

We present ‘HomoTherm’, a human heat budget model for simulating humans and related species with diverse traits in diverse environments. It is part of the NicheMapR package for biophysical modelling and is a multi‐part extension of the ‘endoR’ function. We test the model against a wide range of empirical studies of humans of different shapes and sizes in indoor and outdoor environments and compare it to existing open‐source approaches to modelling human heat budgets. A Shiny implementation of the HomoTherm human heat balance model (pictured) allows the user to simulate heat exchange for a human under realistic outdoor microclimatic conditions anywhere in the world using a historical monthly climatology at hourly time steps across a day. The model allows many traits to vary, including size, shape, clothing, activity level, and thermal physiology. Comparisons can be made with other indices of human heat load including the UTCI and wet bulb temperature. An R function can be produced to replicate the simulation.

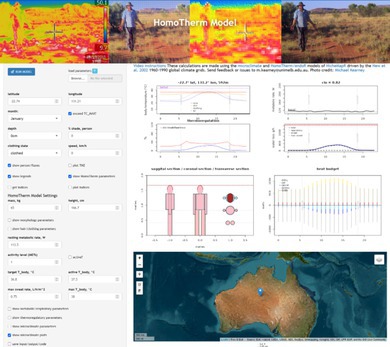

## 1 Introduction

Without thermoregulatory pre‐adaptations to open arid environments, hominins would not have coped with the effects of climate change in the past (Wheeler 1991; Ruxton and Wilkinson 2011a, 2011b; Dávid‐Barrett and Dunbar 2016; Notley et al. 2024). Now, inferences of the direct effects of climate on human heat load will be vital to our success in adapting to global warming (McMichael and World Health Organization 2003; McMichael and Dear 2010; Sherwood and Huber 2010; Dunne et al. 2013; Raymond et al. 2020; Buzan and Huber 2020; Powis et al. 2023). Emma Ramsay and colleagues recently warned that a billion people in informal settlements could be in danger because the heat stress that they will be exposed to has not been adequately assessed (Ramsay et al. 2024). Ideally the inferences of the effects of climate should be based on models that are grounded in known physical principles (e.g., Monteith 1974; Parsons 2014). Because of the diverse range of responses of humans to heat (Matthews et al. 2024), the models must be generalisable to diverse human forms, physiology, and clothing, and applicable to diverse environments including outdoor settings, and to diverse activity levels. Detailed models of human heat balance exist that are both physically‐explicit (e.g., Myrup and Morgan 1972; Maloney and Forbes 2010) and physiologically‐explicit (e.g., Fiala and Havenith 2015; Ioannou et al. 2019; Vanos et al. 2023), yet most studies of human responses to climate change rely on stress or strain indices (Notley et al. 2023) or greatly simplified models (Maloney et al. 2024). For example, the Universal Thermal Comfort Index (UTCI) is derived from a statistical description of the output from a detailed heat budget model but for a single parameterisation that includes assumed changes in clothing and behaviour (Jendritzky et al. 2012).

Why are the more detailed, physically and physiologically, explicit models so rarely applied? We suggest that the main issues have been the accessibility of existing models (e.g., proprietary or closed‐source software and unfamiliar coding language), prohibitive computational requirements, missing environmental input requirements, and/or limited formulations for thermal budget. Examples of the rare applications of physically/physiologically explicit models include that of Maloney and Forbes (2010). They applied an Excel encoding of the MANMO model (Myrup and Morgan 1972) to an Australian city using weather station data to assess how outdoor activity might be restricted under climate change, with an emphasis on the importance of acclimation to heat as well as physiological (sweat limitation) and physical (evaporative potential) limits on the potential to achieve heat balance. The Predictive Heat Strain (PHS) model, incorporated in ISO 7933:2004, is in widespread use in health and industry (Malchaire et al. 2001; Ioannou et al. 2019). Vanos et al. (2023) generated the Human Heat Budget (HHB) model, a model that is simpler physically than both the MANMO and the PHS models (Parsons 2014; Cramer and Jay 2019), but generalises across different aged people and at a global scale. Skin temperature must be specified in advance for the HHB model, yet skin temperature is a dependent variable in the human thermal response. Neither the MANMO or HHB models solve for metabolic rate or clothing temperature, the PHS does not directly incorporate solar radiation, and none of these models is explicit about the shape and clothing of body parts, all of which can limit their generalisability to different people and environments. Thus, a gap exists in our capacity to model the heat budget of diverse people in diverse environments.

Here we present the HomoTherm model that aims to fill this gap in modelling capacity. The HomoTherm model is a multi‐part extension of the general endotherm model of the NicheMapR package for the R programming environment. It aims to strike a balance between computational speed and physical/physiological realism to enable practically useful calculations of how complex environments interact with diverse human characteristics to influence heat exchange so that we can answer general questions about physiological responses to both hot and cold environments. We include HomoTherm as part of the NicheMapR package to permit the direct use of that package to model microclimate, and we present an interactive web‐based Shiny application of the model. In addition to a detailed description of the model, we include tests and demonstrations of the model against empirical responses that were measured during laboratory and outdoor exposure of humans. We have developed open‐source R implementations of the MANMO and HHB models (provided as part of the Supporting Information) that illustrate how the HomoTherm model extends our capacity to model the response to thermal stress in humans.

## 2 Methods

### 2.1 General Overview of the NicheMapR Endotherm Model, ‘endoR’

The NicheMapR package includes the function ‘endoR’, a modularised version of a program that solves the heat budget of a generalised endotherm (Porter et al. 1994; Mathewson and Porter 2013; Kearney et al. 2021). An organism can be modelled as a single object or as a combination of multiple parts. The program can handle ellipsoidal, cylindrical, or plate‐shaped objects that can be insulated with a layer of fat and/or a layer of porous material (e.g., fur). Here we briefly explain how the heat budget is solved for a single part, and then we describe a multi‐part extension that can be used to model a human or other species of hominin.

Heat exchange between an organism and its environment is a non‐linear function of the surface temperature of that organism, whether that surface be the bare skin ($T_{\text{skin}}$) or the surface of an insulating layer (e.g., clothing or hair or feathers; $T_{\mathrm{ins}}$). For endotherms in complex thermal environments, $T_{\text{skin}}$ and $T_{\mathrm{ins}}$ are typically unknown, and must be derived. The main algorithm in the endoR model, ‘simulsol’, solves simultaneously for the required total metabolic heat generation, $Q_{\mathrm{gen}}$, and the associated $T_{\text{skin}}$ and $T_{\mathrm{ins}}$, for the organism to achieve a user‐specified core temperature, $T_{\text{core}}$, under steady‐state exchange with its environment. The pathways of heat exchange with the environment include shortwave $Q_{\mathrm{sol}}$ and net longwave $Q_{\mathrm{rad}}$ radiative exchange, convection (from the skin/clothing $Q_{\text{conv}}$ and respiration/ventilation $Q_{\text{resp},\text{conv}}$), conduction $Q_{\text{cond}}$, and evaporation $Q_{\text{evap}}$ via three pathways: respiratory evaporation $Q_{\text{resp},\text{evap}}$, evaporation from the skin surface (cutaneous, including the eyes) $Q_{\text{evap},\mathrm{cut}}$, and evaporation from the surface of the insulating layer $Q_{\text{evap},\mathrm{ins}}$. The proportion of evaporation from the skin that is covered by insulation (no forced mass‐transfer) is specified via the PCTBAREVAP parameter.

The net delivery of metabolic heat is $Q_{\mathrm{gen},\mathrm{net}}=Q_{\mathrm{gen}}-Q_{\text{resp}}$, where $Q_{\text{resp}}=Q_{\text{resp},\text{conv}}+Q_{\text{resp},\text{evap}}$ and is computed via the molar balance of air flow into the lungs. This air flow is a function of the metabolic demand for oxygen implied by $Q_{\mathrm{gen}}$, together with the gas composition in the environment (CO_2_, N_2_, and O_2_), the respiratory exchange ratio (respiratory quotient at the cell level), and the oxygen extraction efficiency. A root finder is used to compute $Q_{\text{resp}}$ and thus $Q_{\mathrm{gen},\mathrm{net}}$ given that $Q_{\mathrm{gen}}-Q_{\text{resp}}-Q_{\mathrm{gen},\mathrm{net}}=0$.

At steady state, all of the heat that is generated by the body other than that lost from respiration and cutaneous evaporation must pass through the subcutaneous insulation layer of the skin, flow $Q_{\mathrm{ins}}$, and thus, overall:

$$\begin{array}{c} Q_{\mathrm{gen}}-Q_{\text{resp},\text{conv}}-Q_{\text{resp},\text{evap}}-Q_{\text{evap},\mathrm{cut}}=Q_{\mathrm{ins}}=Q_{\mathrm{rad}}+Q_{\text{conv}}\\ +Q_{\text{cond}}+Q_{\text{evap},\mathrm{ins}}-Q_{\mathrm{sol}}. \end{array}$$

Solar radiation is assumed to be absorbed at the exposed surface, whether that be on the insulation or the bare skin. Environmental variables, insulation parameters, and reflectance to shortwave radiation can be distinct dorsally and ventrally to account for complex radiative settings (e.g., different ground and sky temperatures and different combinations of direct and diffuse solar radiation). The thermal conductivity of the insulation can be specified directly or can be computed as a function of the length, diameter, density, and depth of fibres that make up the insulation (for theory see Kowalski 1978; Kowalski and Mitchell 1979; Conley and Porter 1986; Kearney et al. 2021). The thermal conductivity of fat and ‘flesh’ (non‐fatty tissue or ‘lean’ tissue) is specified directly, and fat can be distributed evenly or as a subcutaneous layer. The organism's mass, density, and relative dimensions (e.g., ratio of diameter to length for a cylinder) can be specified, from which absolute dimensions and surface areas are computed internally by a geometry subroutine.

Because the ‘endoR’ algorithm computes the metabolic rate that is required to balance the steady state heat budget for a given core temperature, a minimum allowable metabolic rate, $Q_{\min}$, must be specified as a parameter. This minimum allowable metabolic rate can be the basal or resting metabolic rate or a field metabolic rate that is higher than basal due to digestion, movement, or other metabolic activity. If the computed value of $Q_{\mathrm{gen},\mathrm{net}}$ is below $Q_{\min}$, and hence physiologically impossible, thermoregulatory algorithms are invoked that systematically change model parameters, in series or in parallel, to attempt to find a viable solution to achieve thermal steady state in the environment in question. Options include a change in shape (i.e., posture), an increase or decrease the thermal conductivity of the flesh (i.e., vasodilation or vasoconstriction and associated changes in blood flow), panting (not used by humans and therefore omitted in HomoTherm, see below), an increase in core temperature (with *Q*_10_ effects on metabolic rate that affect the calculations of heat balance), and sweating. In this way, given an environment, the model can compute if it is possible to maintain thermal homeostasis, the required energy or water that must be expended to achieve that thermal homeostasis, and the resulting temperature of the core, skin, and insulation.

### 2.2 Adaptation of ‘endoR’ to Humans and Other Hominins

**FIGURE 1:**
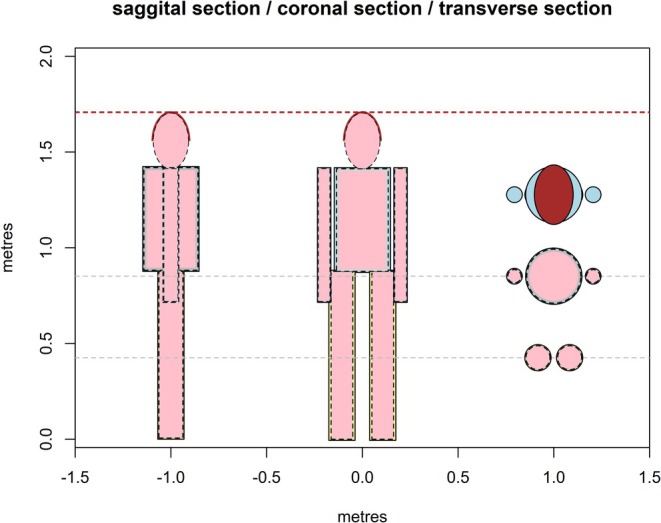
Output of the ‘plot_human’ function of NicheMapR that plots a to‐scale sagittal, coronal, and transverse section of the human geometry that is specified by the user. Pink is flesh, grey is fat, brown is hair, blue and yellow are clothing. The red‐dashed line is the target height, and the black line (here overlapping the red) is the resultant height. The two grey dashed lines indicate where the transverse sections are cut. The top ‘transverse section’ is a birds‐eye view. Black dots indicate the mid‐points or each body part calculated by the ‘get_heights’ function; at each point the local air temperature, wind speed, and relative humidity can be estimated.

The new R function ‘HomoTherm’ is a multi‐part application of ‘endoR’ to humans, which partitions the endotherm's body into an ellipsoidal head and cylindrical trunk, legs, and arms (Figure 1), cylinders being a suitable shape to model humans (Joshi et al. 2024). As in ‘endoR’, the initial estimate of $Q_{\mathrm{gen},\mathrm{net}}$ is set to zero and a while‐loop is applied on the condition that $Q_{\mathrm{gen},\mathrm{net}}$ is lower than $Q_{\min}$, with an internal loop through each body part. The original ‘endoR’ function is called for each body part (once per limb, with the results doubled) but with the arguments ‘THERMOREG’ and ‘RESPIRE’ set to 0 so that no thermoregulation or respiration is initially simulated (but see below). The part‐specific state of peripheral blood flow of the human can be set to be in a fully vasoconstricted state (KFLESH at a minimum) at the beginning of the algorithm, to assess the maximum potential to defend against hypothermia in cold environments, or any other initial state of interest. After each part has been simulated, the total metabolic rate is summed across all parts and a surface area‐weighted mean core temperature, and dorsal and ventral insulation and skin temperatures, are obtained. As in ‘endoR’, the temperature of the upper respiratory tract (for ventilatory heat and water exchange) is computed as the temperature half way between the core and the beginning of the fat layer (see Appendix S1, all appendices available at https://figshare.com/s/9c235ba5b373509b0bf7), and expired air temperature is either the mean of air and respiratory tract temperature, or respiratory tract temperature, whichever is lowest. Respiratory heat loss due to evaporation and convection in the airway is computed to obtain the final estimate of $Q_{\mathrm{gen},\mathrm{net}}$. If this value is higher than the minimum allowable metabolic rate, $Q_{\min}$, then the human is compensating for some degree of cold stress, i.e., is below the lower critical (air) temperature (LCT), and the model has estimated by how much the metabolic rate must be elevated to stay at the required core temperature. If $Q_{\mathrm{gen},\mathrm{net}}<Q_{\min}$, then the human is either within the thermoneutral zone (TNZ) or is under heat strain and compensatory mechanisms must be in place.

**FIGURE 2:**
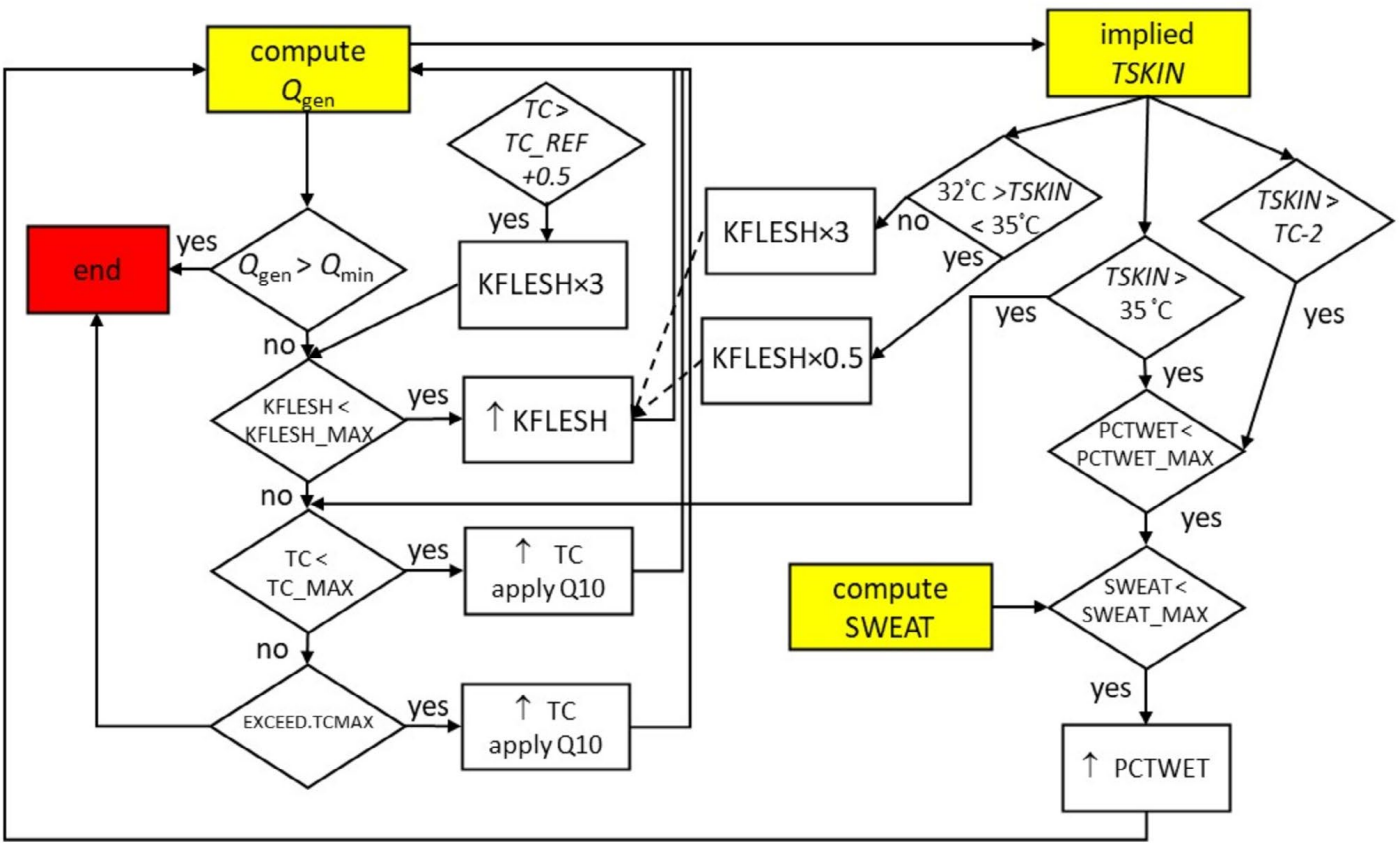
Human thermoregulatory algorithm employed in the HomoTherm model that simulates vasodilation and an increase in core temperature, rising in sequence to achieve thermal steady state within the target range of core temperature (36.8°C–38°C). In addition, skin and core temperature‐based thresholds drive augmented sweating, vasodilation, and rise in core temperature, all of which can potentially increase in parallel. Vasodilation was coded to capture the tendency for skin temperature to be controlled close to 35°C under heat strain. See main text for more detail.

When the human is not below the LCT, i.e., when $Q_{\mathrm{gen},\mathrm{net}}<Q_{\min}$ (and thus potential heat storage is implied), then a human‐specific thermoregulatory algorithm (Figure 2) is activated. With each iteration, the heat generation *Q*_*gen*_ required to meet the current core temperature target TC_REF is computed along with the implied skin temperature TSKIN. Heat generation lower than the allowable minimum *Q*_*min*_ triggers an increase in flesh conductivity KFLESH that simulates peripheral vasodilation, at a step size that is driven by the current estimate of skin temperature (KFLESH × 3 or KFLESH × 0.5). The influence of any simulated subcutaneous fat on heat flow is gradually removed (not shown in diagram) once the flesh conductivity exceeds 0.5 W m^−1^ K^−1^ (which marks the transition from vasoconstriction to vasodilation). Once the thermal conductivity of the flesh reaches its maximum value (default 5 W m^−1^ K^−1^), the core temperature is simulated to rise at the user‐defined step size up to the user‐specified maximum (default of 38°C). Metabolic rate is always elevated when core temperature increases according to a user‐specified *Q*_10_ value. In parallel, sweating is invoked when the skin temperature rises to within 2°C of the core temperature by incrementing the percentage of the skin of each body part that is wet, parameter ‘PCTWETs’, by a user‐specified step size. Functionally, an increase in PCTWET models the stimulation of sweating. The maximum allowable PCTWET value is specified by the user and further constrained by a user‐specified maximum sweat rate (accounting for the dripping of sweat, that will not contribute to evaporation, via the standard equation for evaporation efficiency *r* = 1—w^2^/2 (Parsons 2014, 43)). If skin temperature exceeds 35°C, then the sweat rate and core temperature are additionally incremented, with PCTWET increasing at 10× the user‐specified step size (omitted from figure). If core temperature reaches the user specified maximum ‘TC_MAX’ and all physiologically permissible options are exhausted, the default behaviour (parameter ‘EXCEED.TCMAX’ = TRUE) is to continue to increment the core temperature to find the theoretical steady state core temperature (up until a maximum number of iterations specified by ‘MAXITER’). Alternatively, if ‘EXCEED.TCMAX’ = FALSE, the simulation exits with a solution that implies heat storage ($Q_{\mathrm{gen}}<Q_{\min}$) and therefore with a metabolic rate below the stated minimum.

The function returns all of the outputs that ‘endoR’ returns per body part, plus a weighted summary for the whole body (core, skin, and insulation temperature, skin wetness, all heat flows, sweat rate, evaporation rate, and flesh conductivity). A detailed overview with examples can be found in Appendix S1.

### 2.3 Environmental Variables

The ‘HomoTherm’ algorithm requires the variables that are specified in Appendix S1 and can be called directly per environmental condition or with the ‘HomoTherm_var’ function that loops through vectors of environmental conditions. The microclimate model of NicheMapR can be used to generate the required microclimatic variables and a range of functions is available in the package to run the microclimate model from different weather/climate, terrain, and soil datasets (Kearney, Shamakhy, et al. 2014; Kearney and Porter 2017; Kearney and Maino 2018; Kearney et al. 2020; Kearney 2020; Klinges et al. 2022). Gridded datasets of historical microclimate are also available (Kearney, Isaac, and Porter 2014; Kearney 2019a, 2019b; Meyer et al. 2023).

### 2.4 Parameters and Geometry

A full list of parameters that are required for the human that is being simulated by ‘HomoTherm’ is given in Appendix S1. Humans of different shape and size were mimicked by assembling the simple geometrical shapes (ellipsoids and cylinders) while known scaling relations of surface area and silhouette area with size and height were maintained. The body parts were ‘joined’ appropriately so that the connected parts of each shape did not exchange heat with the environment. The function ‘get_body.sa.props’ contains body masses from Plagenhoef et al. (1983, their table 4) and surface areas from Cross et al. (2008, their table 5), and produces vectors of relative proportions of surface area and mass of the head, trunk, arms combined, and legs combined.

Given a total mass and a surface area of the human, the user can compile the desired shape via the ‘SHAPE_Bs’ argument, which specifies the ratio of the length to the diameter for the cylindrical parts and the semi‐major to semi‐minor axes of the ellipsoid head (prolate spheroid). The function ‘GET_SHAPES’ adjusts the initial specification of the SHAPE_Bs values for each body part as well as the fraction of each body part that is connected to other body parts, given the known total surface area and total mass, and the fractions of surface area and mass that each body part contributes to the whole body. It essentially breaks the body into separate parts, works out their shapes, then puts them back together to match the final sum of areas with the given areas of the parts. That function then reports back the proportionality constants for the modified SHAPE_Bs and the fraction of each body part that is connected to the rest of the body. The latter fractions are assumed to undergo conductive heat exchange to a substrate at core temperature, which has the effect of removing these joining surface areas from any form of heat exchange with the environment. At present, it is assumed that there is convection and radiation but no conduction to the substrate. The function ‘plot_human’ generates a to‐scale plot of the resulting body form in coronal, sagittal, and transverse sections, showing clothing, hair (hair/beard), and fat insulation (Figure 1). The function ‘get_heights’ determines the height above ground of the mid‐point of each body part (Figure 1).

In outdoor environments it is important to determine the amount of direct and diffuse solar radiation that is absorbed by the various body surfaces (Mitchell et al. 2024). The relevant surface area for the direct absorption of solar radiation is the silhouette area, $A_{\mathrm{sil}}$, which is a function of the solar zenith angle ɵ. Underwood and Ward (1966) developed such a function for a standing human. The ‘GEOM_ENDO’ function of NicheMapR computes $A_{\mathrm{sil}}\left(ɵ\right)$ but typically overestimates the radiant heat load compared to values that are determined empirically, and therefore would overestimate the heat load from direct solar radiation that is absorbed by a human. To account for this discrepancy, rather than the application of an internal correction for $A_{\mathrm{sil}}\left(ɵ\right)$, a correction factor for each specific zenith angle $\Delta_{\mathrm{sil}}\left(ɵ\right)$ was computed by modelling the ratio of empirical to observed values as a polynomial function (8th order). The direct and diffuse radiation was then computed using the empirical and geometric methods and their ratio was used to adjust the total incoming solar radiation.

## 3 Results

### 3.1 Example Simulations

A detailed vignette (Appendix S1) explains all the inputs and outputs of the model and provides example calculations. The simplest execution of the model is that applicable to an indoor environment (where the radiant temperature is equal to the air temperature) for the default human (parameters in Appendix S1):

> library(NicheMapR)

> HomoTherm.out <‐ HomoTherm(TA = 21, VEL = 0.1, RH = 50) # run calculation

> HomoTherm.out$balance

HomoTherm.out$respire

> HomoTherm.out$trunk.treg

> HomoTherm.out$trunk.enbal

> HomoTherm.out$trunk.morph

The ‘balance’ output reports the core, lung, skin, and clothing temperature, the skin wetness and flesh thermal conductivity, the litres of water that will be lost per hour from cutaneous and respiratory evaporation, and that lost by sweating, as well as all of the heat flow terms in the heat budget. The ‘respire’ output gives the hourly air and oxygen flow in litres and the inspired and expired mass flow of air and oxygen in moles. There are three additional output tables for each body part. The ‘treg’ output tables give the core temperature, temperatures of the skin and insulation (hair/clothing) on the dorsal and ventral surfaces, skin wetness, flesh and insulation thermal conductivity, and the *Q*_10_ value that is associated with the core temperature. The ‘enbal’ tables report the heat flows in W, the overall energy balance, the number of iterations required for a solution, and whether a solution was found successfully. Finally, the ‘morph’ tables provide the mass, area, volume, characteristic dimension, mass of fat, fat thickness, flesh volume, length width and height of the part, radius to skin and to insulation, areas of the skin, silhouette area, area for evaporation, and area joined to another body part, as well as the radiation configuration factors to the sky and ground.

**FIGURE 3:**
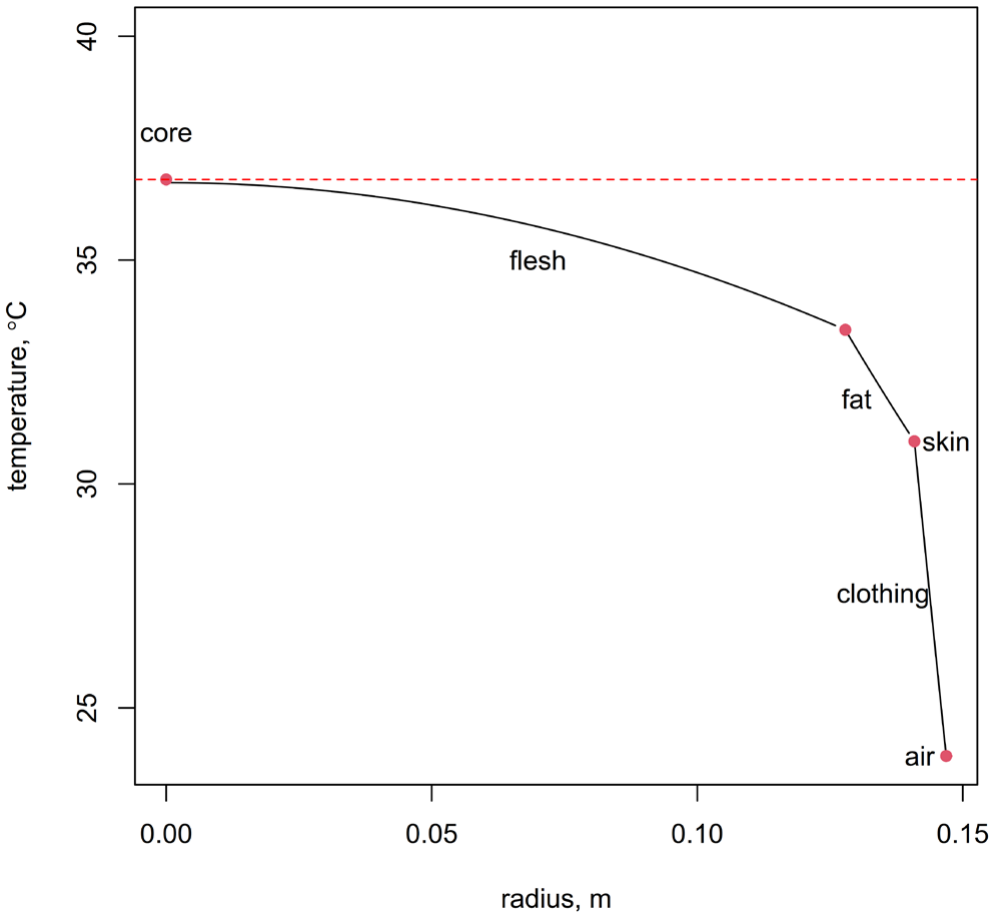
Temperature profile from core to fat to skin to clothing predicted by the HomoTherm model for the trunk under default settings. Dashed horizontal line indicates the target core temperature.

The vignette (Appendix S1) shows how to compute the profile of temperature through the body from the core to the outer edge of the insulation (e.g., Figure 3 for the default settings of HomoTherm).

**FIGURE 4:**
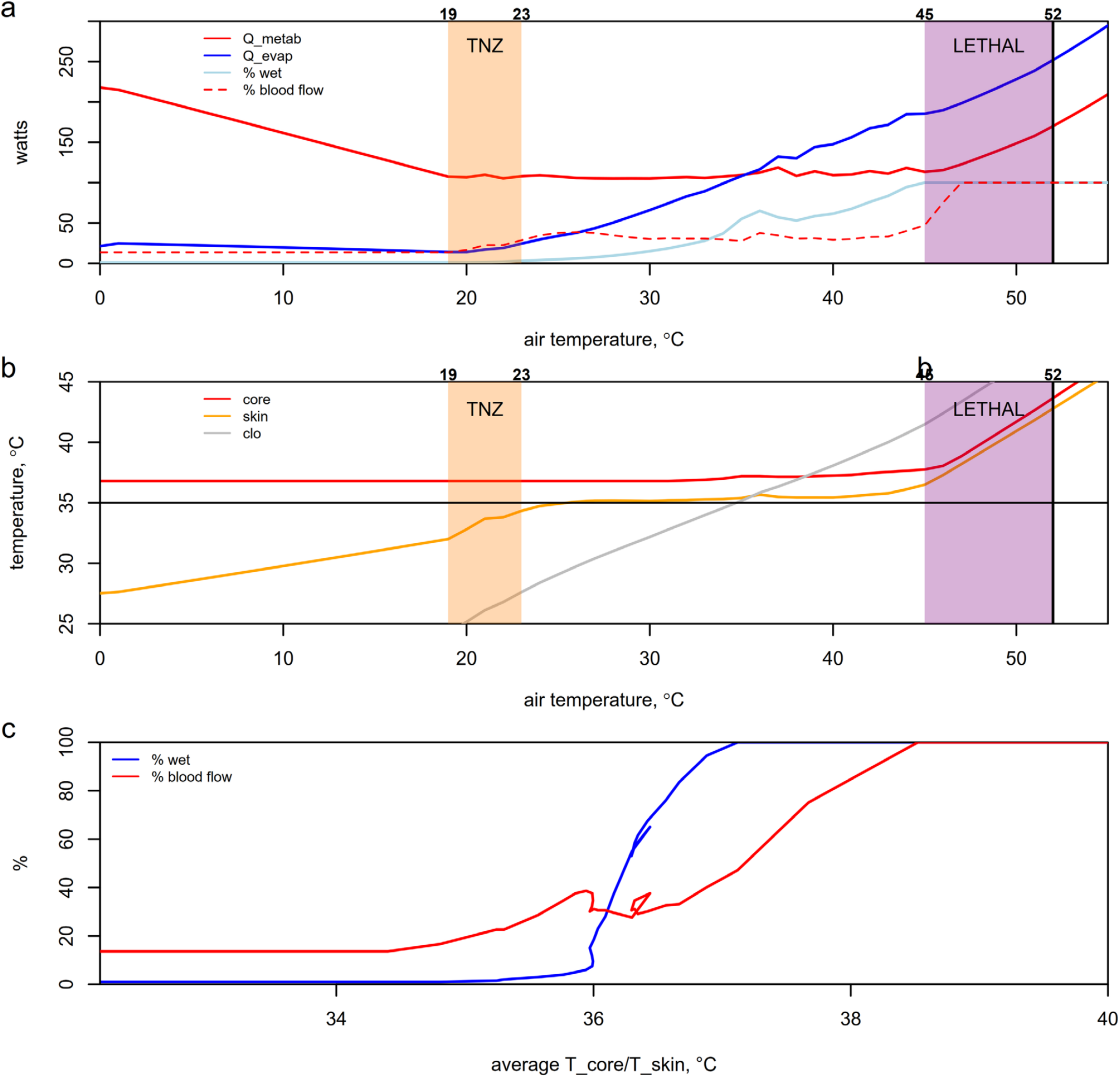
The thermoneutral zone (TNZ), with the upper critical temperature defined arbitrarily as that air temperature when skin wetness exceeds 2%, and the lethal zone for a given human (a), the associated body temperatures (b), and the change in skin wetness/peripheral blood flow against the mean of core and skin temperature (c), as predicted with the HomoTherm_var function using default settings (light clothing).

The next example shows how the HomoTherm_var function can generate a plot of the thermoneutral zone at 50% relative humidity and low wind speed (Figure 4, see Appendix S1 for the full code and further examples):

> TAs <‐ seq(0, 55) # sequence of air temperatures, °C

> VELs <‐ rep(0.1, length(TAs)) # keep wind speeds constant, m/s

> RHs <‐ rep(50, length(TAs)) # keep humidity constant, %

> # simulate across the air temperatures

> HomoTherm.out <− HomoTherm_var(TAs = TAs, VELs = VELs, RHs = RHs)

### 3.2 Shiny Web Application

We have developed a Shiny web application for the HomoTherm model that facilitates the use of the model: http://bioforecasts.science.unimelb.edu.au/app_direct/HomoTherm/. The app is one of a series of apps for the NicheMapR models that allow simulations to be run at different geographic locations, with a facility to vary all of the main morphological and physiological parameters. The app then produces plots of the main outputs and tables of output data as well as R scripts to run the simulations locally. The apps all interface with different climatic forcing datasets; the HomoTherm application currently applies the monthly long‐term average dataset of New et al. (2002). The HomoTherm app additionally computes several thermal comfort indices (Fanger's 1970, thermal sensation index and Winslow et al.'s 1938 ‘pleasantness’ index), the Universal Thermal Climate Index (UTCI: Bröde et al. 2012), the wet‐bulb temperature, and the wet‐bulb‐globe temperature, and the thermoneutral zone (for all hours of the year). The app includes an option to produce R code that will run the simulations and produce the plots.

### 3.3 Tests and Applications of the Model

To assess how well the model captures the empirical thermal responses of human subjects we have applied the HomoTherm model to data that are available in a wide range of published studies. For each of the examples, we provide vignettes and R code that generates the figures and comparisons (included as Appendices [Link], [Link], [Link], [Link], [Link], [Link], [Link]). In most cases we compare the results from HomoTherm with the MANMO model of Myrup and Morgan (1972). The code for MANMO was carefully checked, corrected, and tested (Appendix S9) and the R implementation is provided in Appendix S10. Where possible, we compare the results to the Human Heat Balance (HHB) model of Vanos et al. (2023) (R versions in Appendices S11 and S12), and the PHS model (Malchaire et al. 2001) via the calcIso7933 function of the ‘comf’ R package (Schweiker 2016). A comparison of the HomoTherm model is made with the Wheeler model for extinct hominins as implemented by Ruxton and Wilkinson (2011a) (Appendix S13). Finally we compare model predictions against observations made hourly from 1 to 9 h of humans exposed to a hot environment (40°C, 10% humidity; Meade et al. 2023) to illustrate how our steady state calculations compare with steady state scenarios (Appendix S14). Below we present the results of four illustrative examples.

### 3.4 Elevation of Metabolic Rate in the Cold

**FIGURE 5:**
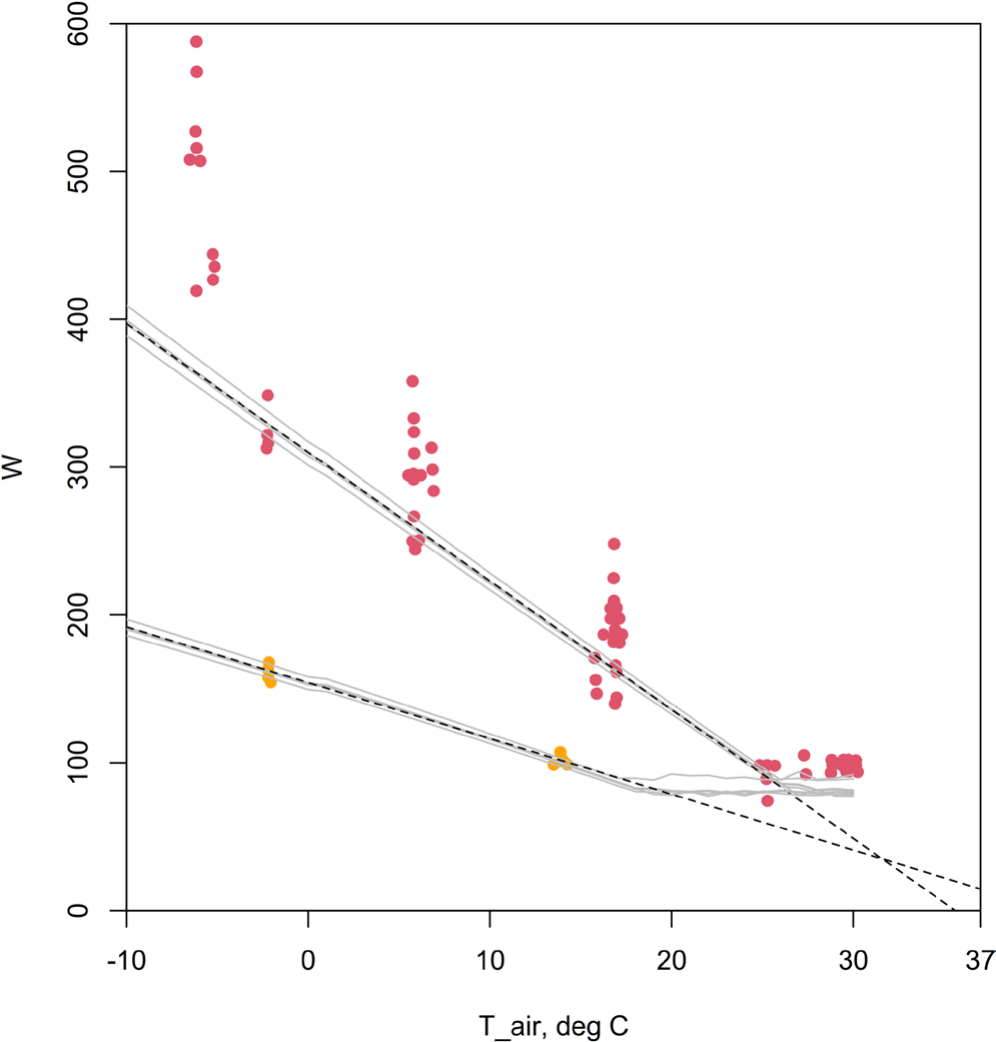
Metabolic rate versus air temperature for five men who cycled on an ergometer wearing either shorts (red dots) or heavy winter clothing (orange dots), with model predictions (grey lines) and the slopes of regressions through the model predictions (dashed black lines).

Because the HomoTherm model solves for the metabolic rate that balances the heat budget of the modelled human, it can estimate the elevation of metabolic rate that is required to maintain thermal homeostasis in the cold. Erikson et al. (1956) presented data on five men who cycled on an ergometer at a range of ambient temperatures, wearing either just shorts or very heavy clothing. The HomoTherm model captured the lower bound of metabolic rates under each clothing scenario with all of the settings at default values other than the body mass and the thickness of the clothing (Figure 5, Appendix S2).

### 3.5 Diverse Body Shapes in Still Air

The pioneering study of Winslow et al. (1937) on human heat exchange considered two human subjects, one ‘pyncic’ (stout, 104 kg, 170 cm) and the other ‘leptosomic’ (slender, 48 kg, 165 cm). The researchers measured the metabolic rate (via respirometry) and the water loss rate (via gravimetry) along with rectal temperature and skin temperature at 15 locations on the body over a wide range of air and radiant temperatures, at ~45% relative humidity and minimal air movement. The radiant and air temperature were integrated into a single metric: the ‘operative temperature’. We assessed the ability of the HomoTherm, MANMO, and HHB models to predict their findings; for the MANMO and HHB model, the observed metabolic rate was given as input, as was the skin temperature for the HHB model, whereas the HomoTherm model was given only the resting metabolic rate as input (see Appendix S3 for all comparisons).

**FIGURE 6:**
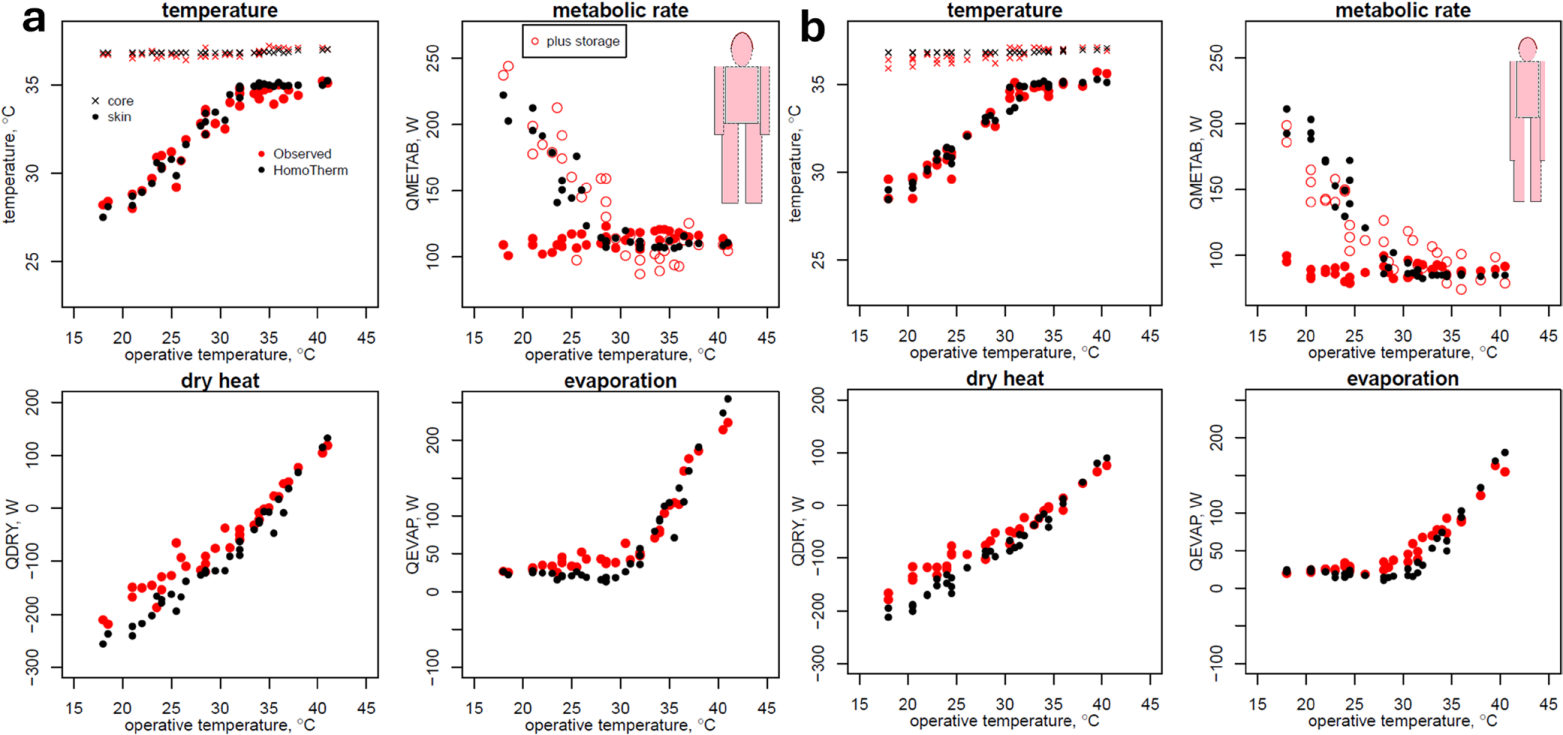
Observed (red symbols) and predicted (black symbols) body temperature (skin—circles, rectal—crosses), metabolic rate, dry heat exchange, and evaporative heat exchange for a stocky subject (a) and a slender subject (b). The observed metabolic rates are reported (closed circles) together with metabolic rates adjusted to account for loss of heat from the body in the transient state (open circles).

**FIGURE 7:**
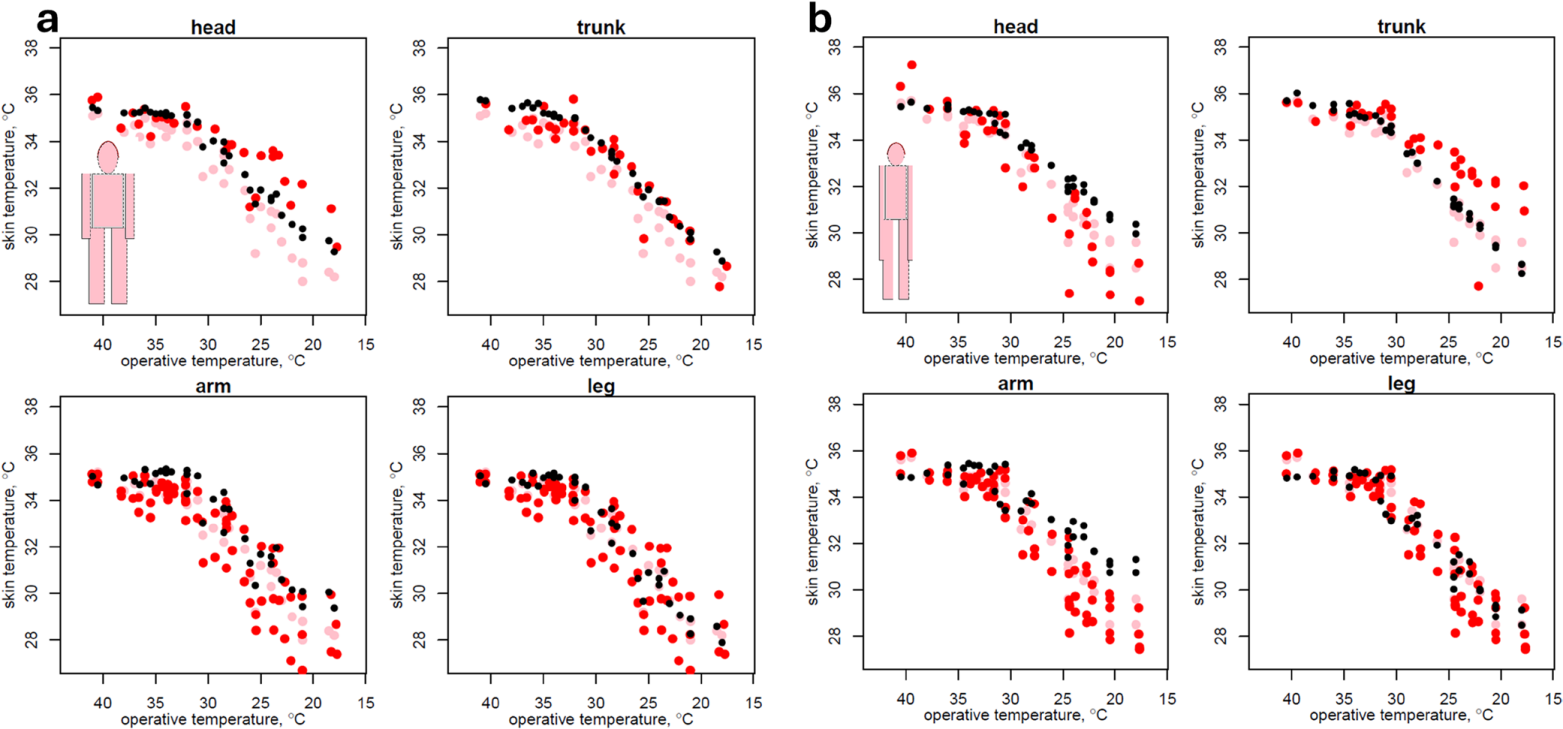
Observed (red) and predicted (black) skin temperatures for different parts of the body for the stocky subject (a), and the slender subject (b). Pink points indicate the observed weighted mean skin temperatures for reference (identical in each plot). The ‘observed’ values for the leg and arm are not specifically for those body parts, but are measurements of ‘extremities’ for both the upper and the lower region of the legs and arms.

The HomoTherm model closely captured the empirical patterns and the qualitative differences between the two body morphs (Figures 6 and 7) but underestimated the metabolic rate during exposure to low operative temperatures (Figure 6, solid black points). That discrepancy was due to the HomoTherm predictions being steady state and the observations having been made in a transient state. The duration of exposure was not reported in the paper, but indications in earlier papers from the same team suggest that it was less than that required to reach thermal steady state (Meade et al. 2024). The subjects, not in steady state, were losing heat. If metabolic rate was increased to the value that would have countered the rate of heat loss, as inferred by Winslow et al. (1937) (Figure 6, open red points), then the adjusted values coincide with the HomoTherm predictions.

The Homotherm model captured the qualitative and quantitative differences between the body morphs in the skin temperature of the different body parts (Figure 7) under the default assumption that vasoconstriction (controlled by flesh thermal conductivity) was higher for the limbs than it was for the head and torso, and that the resting ‘core temperature’ of the arms (35°C) and legs (36.7°C) was lower than the head and trunk (36.5°C).

### 3.6 Diverse Convective Environments

Mitchell et al. (1968) measured the heat exchange of two humans who were exposed to a wide range of environments with a mean radiant temperature that was equal to air temperature and with low humidity at different wind speeds at near thermal steady‐state (though not for cold environments). Like Winslow et al. (1937), evaporation was determined gravimetrically, metabolic rate via respirometry, skin temperature was measured intermittently at 12 sites, and rectal temperature was measured continuously. Radiant and convective heat flows were measured directly, with calorimetric instruments that were custom‐made for that purpose.

**FIGURE 8:**
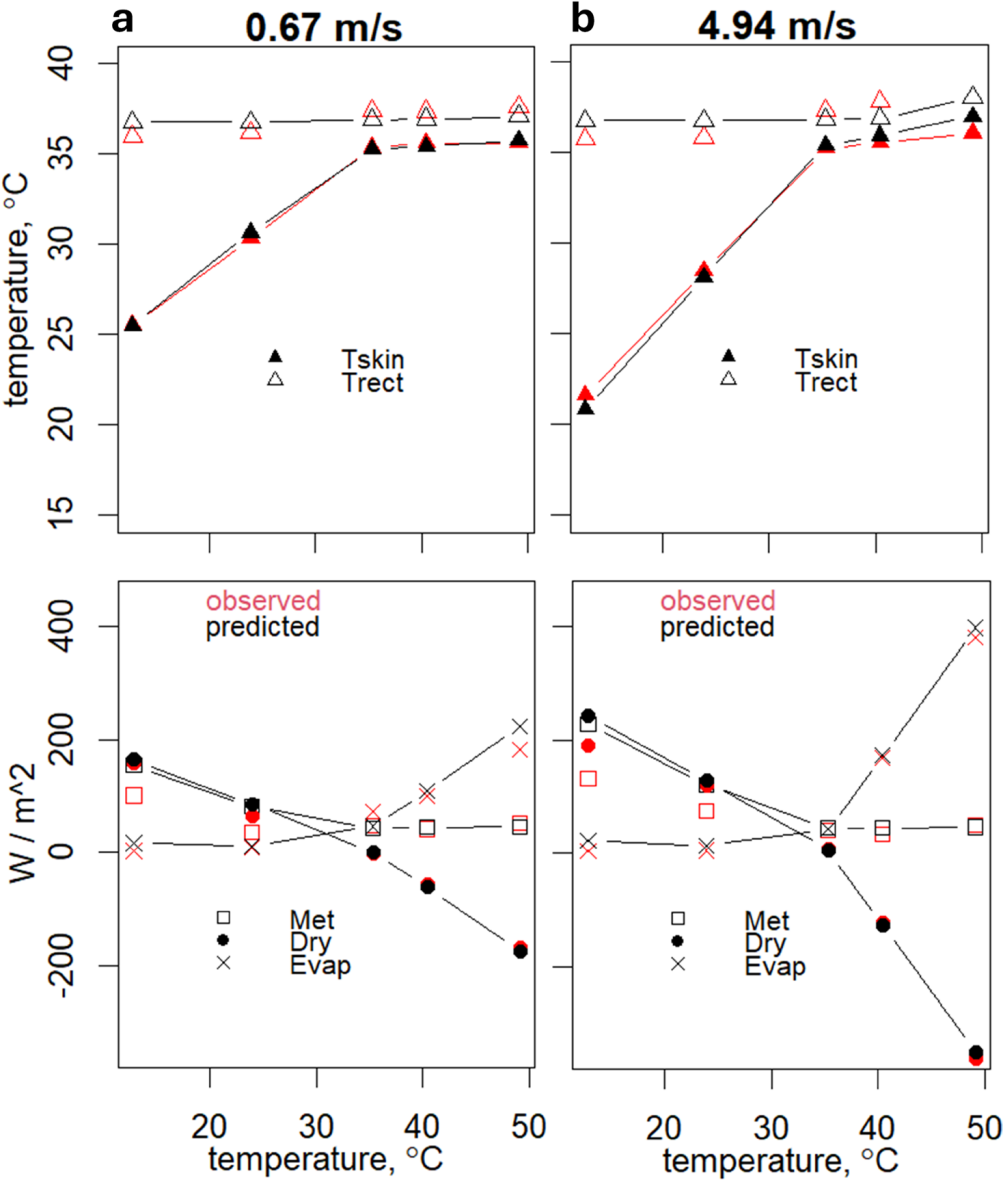
Observed (red) and HomoTherm predicted (black) skin and core temperatures (left panels) and heat fluxes for metabolism, dry heat exchange (convection and radiation), and evaporation (right panels) for experimental observations from Mitchell et al. (1968) at the two extremes of wind speed that were applied in the experiments.

The HomoTherm model accurately estimated the changes in skin temperature and heat flows at the different wind speeds (Figure 8, with Appendix S4 showing comparisons with the other models), while the metabolic rate at the lower ambient temperature was not as precise, as expected, due to exposures being too short to achieve thermal steady‐state. The MANMO model underestimated skin temperature and dry heat flows but accurately predicted evaporation at most wind speeds (Appendix S4). Although the HHB model was given the observed skin temperature as input, its accuracy at predicting evaporation and dry heat flux declined as wind speed increased (Appendix S4). The PHS model closely predicted dry heat gain and evaporative heat loss well except at the extremes of air temperature and wind speed, and poorly predicted the core and skin temperature (Appendix S4). Overall, the HomoTherm predictions had the highest correlations and lowest root mean square deviations compared to the other three models (Table 1).

**TABLE 1:** Summary statistics comparing the fit of the different models to skin and rectal temperature (°C) and energy fluxes (W/m^2^) (reported in Mitchell et al. 1968) for the HomoTherm, MANMO, Predictive Heat Strain model (PHS) and the Human Heat Balance models (HHB).

	Correlation coefficient				Root mean square deviation			
	HomoTherm	MANMO	PHS	HHB	HomoTherm	MANMO	PHS	HHB
Skin temperature	0.997	0.996	0.919		0.44	2.81	2.93	
Rectal temperature	0.502		0.943		0.8		1.31	
Metabolism	0.947				40.16			
Radiation	0.988	0.984	0.988		24.74	29.85	25.47	
Convection	0.989	0.979	0.943		34.52	38.43	49.66	
Dry heat flux	0.997	0.978	0.963	0.997	15.6	49.14	49.96	39.25
Evaporation	0.992	0.984	0.985	0.981	16.83	29.85	24.92	36.18

### 3.7 Bedouin Robes—Clothing Colour in the Desert Sun

**FIGURE 9:**
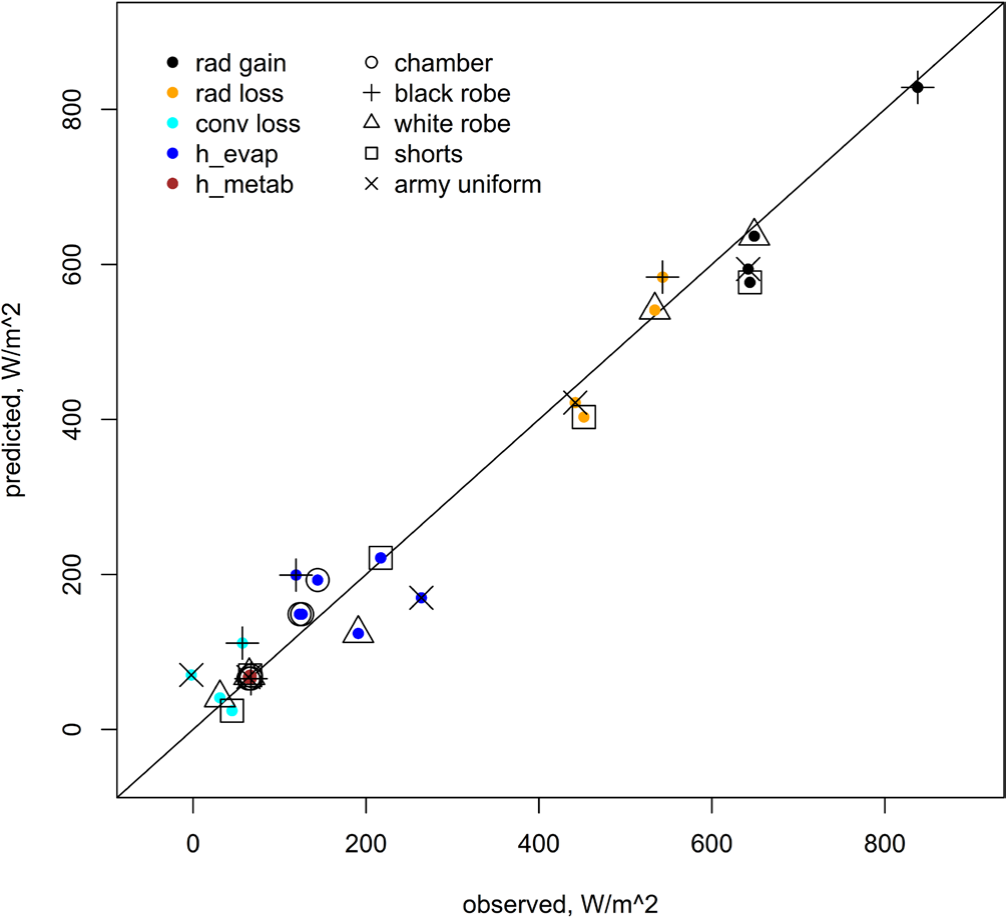
Observed heat fluxes that were measured by Shkolnik et al. (1980) of a human subject outdoors in the Israeli desert wearing different clothing, and in a metabolic chamber at 48°C plotted against the predictions from HomoTherm. The predictions were made for microclimatic conditions simulated for midday in June 1978 with the ‘micro_terra’ function of NicheMapR.

Famously, and counterintuitively, Shkolnik et al. (1980) showed that the heat that reached the skin of a human subject exposed to hot, sunny outdoor conditions and wearing a black Bedouin robe was the same as it was when the subject wore a white Bedouin robe. They quantified the evaporative, metabolic, and storage heat fluxes of subjects who wore the different coloured robes or a tan army uniform, or only shorts (semi‐nude). They also measured the temperature of the skin and outer clothing, under outdoor conditions and in a temperature‐controlled room. The field measurements were made at the Hatzeva Field School in Israel at some time before 1980. The HomoTherm model was used to calculate the expected heat fluxes of a human subject wearing the same clothing types as in the Shkolnik experiments (Appendix S5), using microclimate computed for midday in June 1978 (via the micro_terra function of NicheMapR). The output showed strong correspondence with the observations (Figure 9). The HomoTherm model is unique among open‐source models of human heat budget in that it explicitly calculates the clothing temperature. The predicted outer temperatures of the white and black robe were 39.2°C and 45.1°C, respectively, versus the observed 41°C and 47°C. In contrast, the MANMO model (which uses an empirical function to estimate clothing temperature) substantially underestimated the convective and radiative fluxes and predicted lower and less contrasting temperatures between the two robes (38.5°C for white and 39.2°C for black). Overall, the correlation for the HomoTherm model was 0.99 and 0.96 for MANMO, and the respective root mean square deviations were 41.0 and 71.4 W/m^2^ (see SI Appendix S5 for full details including the MANMO predictions).

## 4 Discussion

Our empirical validations of the HomoTherm model illustrate that the model can predict human thermal responses across a wide range of physical environments and human biological diversity. These include body part‐specific morphological traits such as body proportions, size, clothing, and hair. Genetic and plastic variability in physiology that may be associated with age, fitness, sex, and reproductive state can be incorporated through changes in parameters that define resting or active metabolic rate, vasodilation/vasoconstriction capacity, and sweat rate.

In our tests, comparisons with other models of heat balance that are available show that the HomoTherm model performed as well or better than the other models (with the default physiological parameter settings), although that performance came at a greater computational cost. As indicated in Table 2, the HomoTherm model is physically explicit about more processes than is either the MANMO, PHS, or HHB model, which is reflected in the lines of code (not including data preparation, definition of parameters and variables, or comments) that are required for the computations in each model. The simulation time of the HomoTherm model is fast in absolute terms, given the number of calculations involved (around 0.01 s per solution for a given environment). It is much slower in relative terms, at around three orders of magnitude longer than the HHB model, although the difference is largest when a subject is simulated under heat load because the HomoTherm simulation steps through a sequence of physiological responses when the recruitment of heat loss effectors is required. The differences lie in the extent to which each model compresses the various physical processes into empirical or semi‐empirical functions. The UTCI index (not included in Table 2) can be viewed as an extreme case, with the physical outcomes of a particular set of model runs (including assumptions about behaviour such as clothing changes) compressed into one extremely large polynomial function.

**TABLE 2:** Comparison of the HomoTherm human heat budget model with four existing models of human heat budget, the UTCI‐Fiala model (Fiala et al. 1999, 2001, 2012; Fiala and Havenith 2015), the MANMO model (Myrup and Morgan 1972; Maloney and Forbes 2010), the Predictive Heat Strain (PHS) model (Malchaire et al. 2001), and the Human Heat Balance (HHB) model (Cramer and Jay 2019; Vanos et al. 2023). The comparison includes whether skin temperature *T*_*skin*_, clothing temperature *T*_*ins*_, clothing thermal conductivity *k*_*ins*_, and metabolic rate *Q*_*gen*_ are computed explicitly or are required as input, to what extent the other heat budget terms are based on empirical functions or physically explicit equations, the presence of explicit thermoregulatory algorithms, the computational intensity and complexity (lines of code involved in the calculation), and the availability of the source code. Because it is a proprietary model, some features of the UTCI‐Fiala model are unknown to us.

	HomoTherm	UTCI‐Fiala	MANMO	PHS	HHB
Body parts	4	12	1	1	1
Nodes	28	187	2	3	2
Fat layer	Yes	Yes	No	No	No
*T*_*skin*_	Computed	Computed	Computed	Computed	Given
*T*_*ins*_	Computed	?	Given	Computed	Implicit
*k*_*ins*_	Computed/given	?	Given	Given	Given
*Q*_*gen*_	Computed	Given	Given	Given	Given
*Q*_*sol*_	Explicit	Explicit	Explicit	Radiant offset	Radiant offset
*Q*_*resp*_	Explicit	?	Empirical	Empirical	Empirical
Thermoregulation	Yes	Yes	No	Yes	No
Computational costs	High	?	Medium	Medium	Low
Lines of code	~1000	?	~130	~130	24
Speed comparison^a^	8.5 s	?	0.28 s	5.2 s	0.02 s
Availability	Open	Closed	Open	Open	Open

^a^ Time to simulate heat budget across air temperatures from 0°C to 55°C under 15% relative humidity, replicated ×10.

The simpler models can be applied fruitfully to many important questions about human heat exchange but require caution in their interpretation and execution when extreme or complex environmental settings are modelled. The simpler models need more ‘dependent’ variables to be known (particularly skin temperature for HHB, clothing temperature for MANMO, and metabolic heat generation for both), and the simpler models are less able to capture the range of diversity in physiology and anatomy.

**FIGURE 10:**
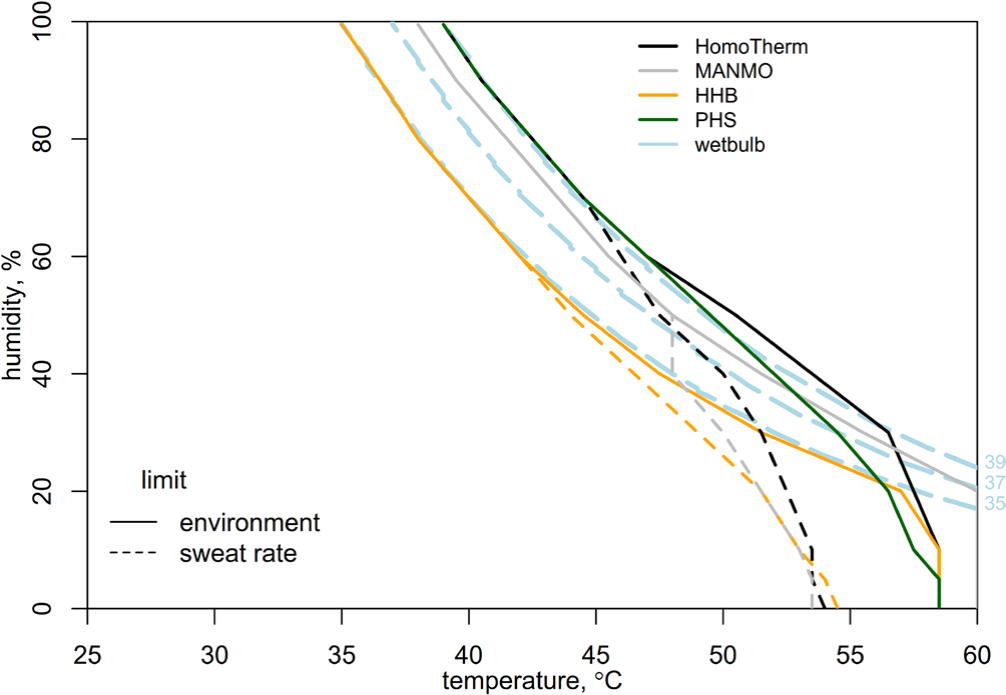
Survivability limits calculated following Vanos et al. (2023) for different combinations of air temperature and relative humidity for the HomoTherm and HHB models. Wet‐bulb temperature contours for 35°C, 37°C and 39°C are indicated by the dashed light blue lines. Situations in which the limits to survival are imposed by the capacity of the environment to accept evaporated sweat (solid) and by the capacity of human subjects to produce sweat (dashed) are shown separately.

To illustrate some of the issues that arise when models are simplified under heat exposure at the extremes that threaten human life, consider calculations of the survivability limit with the HHB and HomoTherm models under different combinations of air temperature, humidity, and radiation for a nude human at 1 m/s wind speed, following Vanos et al. (2023) (Figure 10; for code and further comparisons see Appendix S6). The survivability limits were calculated with the HHB model following Vanos et al. (2023) under the assumptions that skin temperature was constant at 35°C and that the lethal core temperature was 43°C. For the HomoTherm (and MANMO and PHS models in Appendix S6), simulations that produced skin temperature > 40°C were excluded.

The HHB model predicted that the survivability limit was 35°C wet‐bulb if sweating was non‐limiting, whereas the other three models predicted survivability limits of > 37°C wet‐bulb. The HHB model infers steady‐state core temperature assuming a 35°C skin temperature and no *Q*_10_ effect on the metabolic rate. The HomoTherm model allows core temperature to increase as a thermoregulatory response, with an associated increase in metabolic heat production (via a *Q*_10_ of 2), and an increase in skin temperature as the simulation evokes peripheral vasodilation. The nonlinear consequences of these effects interacted in such a way that the steady state core temperature increased earlier, but more slowly, with air temperature at a given humidity than was implied by the HHB model (Appendix S6). When the skin temperature from the output of the HomoTherm model was used as input to the HHB model, the HHB model predicted survivability limits at much higher humidities for a given air temperature (Appendix S6), illustrating the consequences of a fixed skin temperature in the HHB model.

## 5 Conclusion

Overall, the HomoTherm model (with default settings) performed better than the MANMO, PHS, and HHB models for all the comparisons that we made (Table 1). This performance came at a non‐trivial computational cost that was required by the greater physical explicitness and associated generality. The HomoTherm model should be particularly beneficial under extreme conditions where non‐linear effects, resulting especially from predicted changes in the temperature of the skin and clothing, induce outcomes that may differ substantially from the empirical or semi‐empirical functions in the other models. Whether large scale analyses (e.g., across gridded datasets) should use the more computationally intensive HomoTherm approach will of course depend on the question being asked. We recommend that preliminary assessments be made via model comparisons that are grounded by high quality empirical data.

The current algorithm includes a number of threshold states (skin and core temperature) and thermoregulatory step‐sizes that we found match well with known human biology and empirical data, but there may be more computationally efficient or robust alternatives. Moreover, the model is for steady state conditions (like all existing open‐source models) and is yet to be adapted to capture transient states. It is thus most appropriate for understanding exposures in the order of 1–8 h (Meade et al. 2023, 2024; see Appendix S14 for a comparison with Meade et al. 2023). The algorithms and code of the HomoTherm model are freely available (GPLv3 license) as part of the NicheMapR package and will ultimately be developed into a Julia package within BiophysicalEcology.jl (which should lead to gains in computational efficiency). We hope that interested readers will contribute to the packages to maintain, improve, and extend the model.

The HomoTherm model provides greater physical and biological explicitness in modeling human heat budgets than is afforded by current open‐source alternatives. It should therefore allow more robust, reliable, and experimentally testable inferences to be made about the ways that diverse humans respond to diverse environments in the past, present, or future.

## Author Contributions

**Michael R. Kearney:** conceptualization, data curation, formal analysis, visualization, methodology, investigation, software, validation, funding acquisition, supervision, project administration, resources, writing – original draft, writing – review and editing. **Duncan Mitchell:** methodology, writing – review and editing. **Shane K. Maloney:** conceptualization, writing – review and editing, funding acquisition.

## Funding

This work was supported by Wellcome Trust, 227174/Z/23/Z and Australian Research Council, FL240100088.

## Conflicts of Interest

The authors declare no conflicts of interest.

## Supporting information


**Appendix S1:** gcb70830‐sup‐0001‐Appendix 1.pdf.

**Appendix S2:** gcb70830‐sup‐0002‐Appendix 2.pdf.

**Appendix S3:** gcb70830‐sup‐0003‐Appendix 3.pdf.

**Appendix S4:** gcb70830‐sup‐0004‐Appendix 4.pdf.

**Appendix S5:** gcb70830‐sup‐0005‐Appendix 5.pdf.

**Appendix S6:** gcb70830‐sup‐0006‐Appendix 6.pdf.

**Appendix S7:** gcb70830‐sup‐0007‐Appendix 7.pdf.

**Appendix S8:** gcb70830‐sup‐0008‐Appendix 8.pdf.

**Appendix S9:** gcb70830‐sup‐0009‐Appendix 9.pdf.

**Appendix S10:** gcb70830‐sup‐0010‐Appendix 10.pdf.

**Appendix S11:** gcb70830‐sup‐0011‐Appendix 11.pdf.

**Appendix S12:** gcb70830‐sup‐0012‐Appendix 12.pdf.

**Appendix S13:** gcb70830‐sup‐0013‐Appendix 13.pdf.

**Appendix S14:** gcb70830‐sup‐0014‐Appendix 14.pdf.

## Data Availability

The code and data that support the findings of this study are openly available at https://github.com/mrke/NicheMapR, at https://doi.org.10.26188/31808632 and in the Supporting Information section at the end of this article.

## References

[gcb70830-bib-0001] Bröde, P. , D. Fiala , K. Błażejczyk , et al. 2012. “Deriving the Operational Procedure for the Universal Thermal Climate Index (UTCI).” International Journal of Biometeorology 56: 481–494. 21626294 10.1007/s00484-011-0454-1

[gcb70830-bib-0002] Buzan, J. R. , and M. Huber . 2020. “Moist Heat Stress on a Hotter Earth.” Annual Review of Earth and Planetary Sciences 48: 623–655.

[gcb70830-bib-0003] Conley, K. E. , and W. P. Porter . 1986. “Heat Loss From Deer Mice (*Peromyscus*): Evaluation of Seasonal Limits to Thermoregulation.” Journal of Experimental Biology 126: 249–269. 3805994 10.1242/jeb.126.1.249

[gcb70830-bib-0004] Cramer, M. N. , and O. Jay . 2019. “Partitional Calorimetry.” Journal of Applied Physiology 126: 267–277. 30496710 10.1152/japplphysiol.00191.2018 PMC6397408

[gcb70830-bib-0005] Cross, A. , M. Collard , and A. Nelson . 2008. “Body Segment Differences in Surface Area, Skin Temperature and 3D Displacement and the Estimation of Heat Balance During Locomotion in Hominins.” PLoS One 3: e2464. 18560580 10.1371/journal.pone.0002464 PMC2409965

[gcb70830-bib-0006] Dávid‐Barrett, T. , and R. I. M. Dunbar . 2016. “Bipedality and Hair Loss in Human Evolution Revisited: The Impact of Altitude and Activity Scheduling.” Journal of Human Evolution 94: 72–82. 27178459 10.1016/j.jhevol.2016.02.006 PMC4874949

[gcb70830-bib-0007] Dunne, J. P. , R. J. Stouffer , and J. G. John . 2013. “Reductions in Labour Capacity From Heat Stress Under Climate Warming.” Nature Climate Change 3: 563–566.

[gcb70830-bib-0008] Erikson, H. , J. Eirog , K. L. Andersen , and P. F. Scholander . 1956. “The Critical Temperature in Naked Man.” Acta Physiologica Scandinavica 37: 35–39. 13339450 10.1111/j.1748-1716.1956.tb01339.x

[gcb70830-bib-0009] Fanger, P. O. 1970. Thermal Comfort: Analysis and Applications in Environmental Engineering. Danish Technical Press.

[gcb70830-bib-0010] Fiala, D. , and G. Havenith . 2015. “Modelling Human Heat Transfer and Temperature Regulation.” In The Mechanobiology and Mechanophysiology of Military‐Related Injuries, edited by A. Gefen and Y. Epstein , 265–302. Springer International Publishing.

[gcb70830-bib-0011] Fiala, D. , G. Havenith , P. Bröde , B. Kampmann , and G. Jendritzky . 2012. “UTCI‐Fiala Multi‐Node Model of Human Heat Transfer and Temperature Regulation.” International Journal of Biometeorology 56: 429–441. 21503622 10.1007/s00484-011-0424-7

[gcb70830-bib-0012] Fiala, D. , K. J. Lomas , and M. Stohrer . 1999. “A Computer Model of Human Thermoregulation for a Wide Range of Environmental Conditions: The Passive System.” Journal of Applied Physiology 87: 1957–1972. 10562642 10.1152/jappl.1999.87.5.1957

[gcb70830-bib-0013] Fiala, D. , K. J. Lomas , and M. Stohrer . 2001. “Computer Prediction of Human Thermoregulatory and Temperature Responses to a Wide Range of Environmental Conditions.” International Journal of Biometeorology 45: 143–159. 11594634 10.1007/s004840100099

[gcb70830-bib-0014] Ioannou, L. G. , L. Tsoutsoubi , K. Mantzois , and A. D. Flouris . 2019. “A Free Software to Predict Heat Strain According to the ISO 7933:2018.” Industrial Health 57: 711–720. 30918161 10.2486/indhealth.2018-0216 PMC6885605

[gcb70830-bib-0015] Jendritzky, G. , R. de Dear , and G. Havenith . 2012. “UTCI—Why Another Thermal Index?” International Journal of Biometeorology 56: 421–428. 22187087 10.1007/s00484-011-0513-7

[gcb70830-bib-0016] Joshi, A. , S. H. Viswanathan , A. K. Jaiswal , et al. 2024. “Characterization of Human Extreme Heat Exposure Using an Outdoor Thermal Manikin.” Science of the Total Environment 923: 171525. 38458460 10.1016/j.scitotenv.2024.171525

[gcb70830-bib-0017] Kearney, M. R. 2019a. “MicroclimOz—A Microclimate Data Set for Australia, With Example Applications.” Austral Ecology 44: 534–544.

[gcb70830-bib-0018] Kearney, M. R. 2019b. “microclimUS: Hourly Estimates of Historical Microclimates for The United States of America With Example Applications.” Ecology 100: e02829. 31323121 10.1002/ecy.2829

[gcb70830-bib-0019] Kearney, M. R. 2020. “How Will Snow Alter Exposure of Organisms to Cold Stress Under Climate Warming?” Global Ecology and Biogeography 29: 1246–1256.

[gcb70830-bib-0020] Kearney, M. R. , N. J. Briscoe , P. D. Mathewson , and W. P. Porter . 2021. “NicheMapR—An R Package for Biophysical Modelling: The Endotherm Model.” Ecography 44: 1595–1605.

[gcb70830-bib-0021] Kearney, M. R. , P. K. Gillingham , I. Bramer , J. P. Duffy , and I. M. D. Maclean . 2020. “A Method for Computing Hourly, Historical, Terrain‐Corrected Microclimate Anywhere on Earth.” Methods in Ecology and Evolution 11: 38–43.

[gcb70830-bib-0022] Kearney, M. R. , A. P. Isaac , and W. P. Porter . 2014. “Microclim: Global Estimates of Hourly Microclimate Based on Long‐Term Monthly Climate Averages.” Scientific Data 1: 140006. 25977764 10.1038/sdata.2014.6 PMC4387738

[gcb70830-bib-0023] Kearney, M. R. , and J. L. Maino . 2018. “Can Next‐Generation Soil Data Products Improve Soil Moisture Modelling at the Continental Scale? An Assessment Using a New Microclimate Package for the R Programming Environment.” Journal of Hydrology 561: 662–673.

[gcb70830-bib-0024] Kearney, M. R. , and W. P. Porter . 2017. “NicheMapR—An R Package for Biophysical Modelling: The Microclimate Model.” Ecography 40: 664–674.

[gcb70830-bib-0025] Kearney, M. R. , A. Shamakhy , R. Tingley , et al. 2014. “Microclimate Modelling at Macro Scales: A Test of a General Microclimate Model Integrated With Gridded Continental‐Scale Soil and Weather Data.” Methods in Ecology and Evolution 5: 273–286.

[gcb70830-bib-0026] Klinges, D. H. , J. P. Duffy , M. R. Kearney , and I. M. D. Maclean . 2022. “mcera5: Driving Microclimate Models With ERA5 Global Gridded Climate Data.” Methods in Ecology and Evolution 13: 7.

[gcb70830-bib-0027] Kowalski, G. J. 1978. An Analytical and Experimental Investigation of the Heat Loss Through Animal Fur. PhD. University of Wisconsin.

[gcb70830-bib-0028] Kowalski, G. J. , and J. W. Mitchell . 1979. An Analytical and Experimental Investigation of the Heat Transfer Mechanisms Within Fibrous Media. Page American Society of Mechanical Engineers.

[gcb70830-bib-0029] Malchaire, J. , A. Piette , B. Kampmann , et al. 2001. “Development and Validation of the Predicted Heat Strain Model.” Annals of Occupational Hygiene 45: 123–135. 11182426

[gcb70830-bib-0030] Maloney, S. K. , and C. F. Forbes . 2010. “What Effect Will a Few Degrees of Climate Change Have on Human Heat Balance? Implications for Human Activity.” International Journal of Biometeorology 55: 147–160. 20461416 10.1007/s00484-010-0320-6

[gcb70830-bib-0031] Maloney, S. K. , M. R. Kearney , and D. Mitchell . 2024. “Indices of Human Heat Stress in Times of Climate Change.” Acta Physiologica 240: e14196. 38953744 10.1111/apha.14196

[gcb70830-bib-0032] Mathewson, P. D. , and W. P. Porter . 2013. “Simulating Polar Bear Energetics During a Seasonal Fast Using a Mechanistic Model.” PLoS One 8: e72863. 24019883 10.1371/journal.pone.0072863 PMC3760880

[gcb70830-bib-0033] Matthews, T. , E. E. Ramsay , F. Saeed , et al. 2024. “Humid Heat Exceeds Human Tolerance Limits and Causes Mass Mortality.” Nature Climate Change 15: 4–6.

[gcb70830-bib-0034] McMichael, A. J. , and K. B. G. Dear . 2010. “Climate Change: Heat, Health, and Longer Horizons.” Proceedings of the National Academy of Sciences 107: 9483–9484. 10.1073/pnas.1004894107 PMC2906883 20483994

[gcb70830-bib-0035] McMichael, A. J. , and World Health Organization . 2003. Climate Change and Human Health: Risks and Responses. World Health Organization.

[gcb70830-bib-0036] Meade, R. D. , S. R. Notley , A. P. Akerman , et al. 2023. “Physiological Responses to 9 Hours of Heat Exposure in Young and Older Adults. Part I: Body Temperature and Hemodynamic Regulation.” Journal of Applied Physiology 135: 673–687. 37439239 10.1152/japplphysiol.00227.2023

[gcb70830-bib-0037] Meade, R. D. , S. R. Notley , and G. P. Kenny . 2024. “Time to Reach Equilibrium Deep Body Temperatures in Young and Older Adults Resting in the Heat: A Descriptive Secondary Analysis.” American Journal of Physiology. Regulatory, Integrative and Comparative Physiology 327: R369–R377. 39102464 10.1152/ajpregu.00089.2024

[gcb70830-bib-0038] Meyer, A. V. , Y. Sakairi , M. R. Kearney , and L. B. Buckley . 2023. “A Guide and Tools for Selecting and Accessing Microclimate Data for Mechanistic Niche Modeling.” Ecosphere 14: e4506.

[gcb70830-bib-0039] Mitchell, D. , S. K. Maloney , E. P. Snelling , V. F. Carvalho Fonsêca , and A. Fuller . 2024. “Measurement of Microclimates in a Warming World: Problems and Solutions.” Journal of Experimental Biology 227: jeb246481. 38958209 10.1242/jeb.246481

[gcb70830-bib-0040] Mitchell, D. , C. H. Wyndham , A. R. Atkins , et al. 1968. “Direct Measurement of the Thermal Responses of Nude Resting Men in Dry Environments.” Pflügers Archiv ‐ European Journal of Physiology 303: 324–343. 5749160 10.1007/BF00596389

[gcb70830-bib-0041] Monteith, J. L. 1974. “Specification of the Environment for Thermal Physiology.” In Heat Loss From Animals and Man, edited by J. L. Monteith and L. E. Mount , 1–17. Butterworth‐Heinemann.

[gcb70830-bib-0042] Myrup, L. O. , and D. L. Morgan . 1972. Numerical Model of the Urban Atmosphere. Volume I the City‐Surface Interface. University of California.

[gcb70830-bib-0043] New, M. , D. Lister , M. Hulme , and I. Makin . 2002. “A High‐Resolution Data Set of Surface Climate Over Global Land Areas.” Climate Research 21: 1–25.

[gcb70830-bib-0044] Notley, S. R. , D. Mitchell , and N. A. S. Taylor . 2023. “A Century of Exercise Physiology: Concepts That Ignited the Study of Human Thermoregulation. Part 1: Foundational Principles and Theories of Regulation.” European Journal of Applied Physiology 123: 2379–2459. 37702789 10.1007/s00421-023-05272-7

[gcb70830-bib-0045] Notley, S. R. , D. Mitchell , and N. A. S. Taylor . 2024. “A Century of Exercise Physiology: Concepts That Ignited the Study of Human Thermoregulation. Part 4: Evolution, Thermal Adaptation and Unsupported Theories of Thermoregulation.” European Journal of Applied Physiology 124: 147–218. 37796290 10.1007/s00421-023-05262-9

[gcb70830-bib-0046] Parsons, K. C. 2014. Human Thermal Environments: The Effects of Hot, Moderate, and Cold Environments on Human Health, Comfort, and Performance. CRC Press.

[gcb70830-bib-0047] Plagenhoef, S. , F. G. Evans , and T. Abdelnour . 1983. “Anatomical Data for Analyzing Human Motion.” Research Quarterly for Exercise and Sport 54: 169–178.

[gcb70830-bib-0048] Porter, W. P. , J. C. Munger , W. E. Stewart , S. Budaraju , and J. Jaeger . 1994. “Endotherm Energetics: From a Scalable Individual‐Based Model to Ecological Applications.” Australian Journal of Zoology 42: 125–162.

[gcb70830-bib-0049] Powis, C. M. , D. Byrne , Z. Zobel , K. N. Gassert , A. C. Lute , and C. R. Schwalm . 2023. “Observational and Model Evidence Together Support Wide‐Spread Exposure to Noncompensable Heat Under Continued Global Warming.” Science Advances 9: eadg9297. 37682995 10.1126/sciadv.adg9297 PMC10491292

[gcb70830-bib-0050] Ramsay, E. E. , P. Hamel , S. L. Chown , and G. A. Duffy . 2024. “Humid Heat Stress Overlooked for One Billion People in Urban Informal Settlements.” One Earth 7: 2–5.

[gcb70830-bib-0051] Raymond, C. , T. Matthews , and R. M. Horton . 2020. “The Emergence of Heat and Humidity Too Severe for Human Tolerance.” Science Advances 6: eaaw1838. 32494693 10.1126/sciadv.aaw1838 PMC7209987

[gcb70830-bib-0052] Ruxton, G. D. , and D. M. Wilkinson . 2011a. “Thermoregulation and Endurance Running in Extinct Hominins: Wheeler's Models Revisited.” Journal of Human Evolution 61: 169–175. 21489604 10.1016/j.jhevol.2011.02.012

[gcb70830-bib-0053] Ruxton, G. D. , and D. M. Wilkinson . 2011b. “Avoidance of Overheating and Selection for Both Hair Loss and Bipedality in Hominins.” Proceedings of the National Academy of Sciences of the United States of America 108: 20965–20969. 22160694 10.1073/pnas.1113915108 PMC3248486

[gcb70830-bib-0054] Schweiker, M. 2016. “Comf: An R Package for Thermal Comfort Studies.” R Journal 8: 341.

[gcb70830-bib-0055] Sherwood, S. C. , and M. Huber . 2010. “An Adaptability Limit to Climate Change due to Heat Stress.” Proceedings of the National Academy of Sciences of the United States of America 107: 9552–9555. 20439769 10.1073/pnas.0913352107 PMC2906879

[gcb70830-bib-0056] Shkolnik, A. , C. R. Taylor , V. Finch , and A. Borut . 1980. “Why Do Bedouins Wear Black Robes in Hot Deserts?” Nature 283: 373–375.

[gcb70830-bib-0057] Underwood, C. R. , and E. J. Ward . 1966. “The Solar Radiation Area of Man.” Ergonomics 9: 155–168. 5930131 10.1080/00140136608964361

[gcb70830-bib-0058] Vanos, J. , G. Guzman‐Echavarria , J. W. Baldwin , C. Bongers , K. L. Ebi , and O. Jay . 2023. “A Physiological Approach for Assessing Human Survivability and Liveability to Heat in a Changing Climate.” Nature Communications 14: 7653. 10.1038/s41467-023-43121-5 PMC10687011 38030628

[gcb70830-bib-0059] Wheeler, P. E. 1991. “The Thermoregulatory Advantages of Hominid Bipedalism in Open Equatorial Environments: The Contribution of Increased Convective Heat Loss and Cutaneous Evaporative Cooling.” Journal of Human Evolution 21: 107–115.

[gcb70830-bib-0060] Winslow, C.‐E. A. , L. P. Herrington , and A. P. Gagge . 1937. “Physiological Reactions of the Human Body to Varying Environmental Temperatures.” American Journal of Physiology‐Legacy Content 120: 1–22.

[gcb70830-bib-0061] Winslow, C.‐E. A. , L. P. Herrington , and A. P. Gagge . 1938. “Physiological Reactions and Sensations of Pleasantness Under Varying Atmospheric Conditions.” Transactions of the American Society of Heating and Ventilating Engineers 1081: 179–196.

