# Supplementary material for: HomoTherm: An Open‐Source Approach to Modelling Heat Exchange in Humans and Other Hominins in Diverse Environments
Source: Glob Chang Biol. 2026 Apr 1;32(4):e70830. doi: 10.1111/gcb.70830 (PMC13044332; doi:10.1111/gcb.70830)
Supplement: Supplementary file 1 — Appendix S1: gcb70830‐sup‐0001‐Appendix 1.pdf. [file GCB-32-e70830-s007.pdf]

HomoTherm overview and tutorial

Michael Kearney

2026-01-10

Overview

This vignette explains the input and output of the HomoTherm and HomoTherm_var functions of NicheMapR for modelling human heat budgets and provides some example applications. The model is fully described in Kearney et al. (2026) and is a multi-part application of the NicheMapR 'endoR' function for an ellipsoidal head and cylindrical trunk, arms and legs.

First the document summarises all the input variables and parameters and their dimensions and default settings. Then the outputs are described for a simulation for one environment (using 'HomoTherm') with all of the parameters set to default. This includes an example of how to plot the core to clothing temperature gradient, the geometry of the person, and how to set the insulation in terms of 'clo' units. Examples to calculate thermoneutral zones for different clothing and humidity levels (using 'HomoTherm_var') are demonstrated. Finally, an example is given for simulating the responses of a human to a sequence of outdoor environmental conditions.

Load the libraries

To run these examples you will need to install the NicheMapR package. Details can be found here.

```
library(devtools)
install_github('mrke/NicheMapR')
```

```
library(NicheMapR)
```

Environmental variables

The environmental or 'forcing' variables include air temperature, humidity, wind speed, longwave radiation (via sky and ground temperature specifications), direct and diffuse shortwave radiation, barometric pressure and gas (O_2, CO_2 and N_2) concentrations. The 'HomoTherm_var' function allows vectors of environmental conditions to be passed to the program, indicated by the lowercase 's' appended to the variable name. E.g. for HomoTherm you pass 'TA', a single value of air temperature, but for HomoTherm_var you pass 'TAs', a vector of air temperatures. HomoTherm_var optionally calculates vertical profiles in air temperature, wind speed and humidity via NicheMapR's 'get_profile' function (do '?get_profile' to see how this works) as well as accounting for the effects of movement on the wind speed that is experienced on the human body.

Name	Units	Default	Typical Range	Description
TA(s)	°C	21	-89 to 57	Air temperature
TSKY(s)	°C	21	-50 to 60	Sky 'temperature'
TGRD(s)	°C	21	-90 to 85	Substrate temperature

Name	Units	Default	Typical Range	Description
RH(s)	%	50	0 to 100	Relative humidity
VEL(s)	m/s	0.15	0.1 to 30	Wind speed
QSOLR(s)	W/m^2	0	0 to 1,000	Horizontal plane solar radiation
PDIF(s)	-	0.15	0.1 to 1	Fraction of solar radiation that is diffuse
Z(s)	°	20	0 to 90	Solar zenith angle
SHADE(s)	%	0	0 to 100	Shade on human
ABSSB	-	0.85	0 to 1	Fraction of solar radiation absorbed by substrate
BP(s)	Pa	101325	50,000 to 101,325	Barometric pressure[1]
ELEV	m	0	0 to 8,849	Elevation (used to compute atmospheric pressure)
GRAV	m/s^2	9.80665	0 to 9.832	Acceleration due to gravity
O2GAS	%	20.95	5 to 20.95	Oxygen percentage in air
CO2GAS	%	0.0422	0.03 to 0.1	Carbon dioxide percentage in air
N2GAS	%	79.02	79.02	Nitrogen percentage in air
REFHYT[2]	m	2	1 to 2	Reference height for meteorological observations
RUF[2]	m	0.004	0.00001 to 0.02	Roughness height
TAREF(s)[2]	°C	21	-89 to 57	Air temperature at reference height
VREF(s)[2]	°C	0.15	0.1 to 30	Wind speed at reference height
RHREF(s)[2]	%	50	0 to 100	Relative humidity at reference height
SPEED[3]	m/s	0	0 to 10	Movement speed
CONV_ENHANCE	-	1	1 to 1.4	Convection enhancement factor (> 1 for outdoors)[4]

[1] If barometric pressure is unknown, it can be set to a negative value which will trigger elevation to be used to compute barometric pressure.

[2] These values are used when the NicheMapR microclimate model has been used to obtain environmental conditions (see below) so that air temperature, wind speed and relative humidity can be specified at the height of each body part. It uses the 'get_profile' function of NicheMapR which requires values of these environmental variables at a reference height, as well as the aerodynamic properties assumed in the calculation of the microclimate ('RUF').

[3] Will use the maximum of wind speed and movement speed as the air flow in the calculation.

[4] Correction factor to convection (specifically Nusselt number) to capture the fact that, under turbulent outdoor conditions, measurements of convection coefficients from wind tunnel experiments can underestimate convection. See Kowalski and Mitchell (1976).

Parameters and their default values

The following parameters apply to the whole body.

Name	Units	Default	Typical Range	Description
QMETAB_REST	W	105	see[1]	Resting metabolic rate
MASS	kg	70	0.2 to 635	Total body mass
ACTIVE	-	FALSE	TRUE/FALSE	Is the person active?
MET	-	1	1 to 5	Multiplier on QMETAB when active
Q10	-	2	2 to 3	Q10 response of QMETAB to body temperature[1]

Name	Units	Default	Typical Range	Description
RQ	-	0.8	0.7 to 1	Respiratory quotient, CO_2/O_2
EXTREF	%	25	20 to 30 at rest	O_2 extraction efficiency
MAXSWEAT	$L/h/m^2$	0.75	0.5 to 1.5	Maximum sweat rate
EXCEED.TCMAX	-	TRUE	TRUE/FALSE	Allow body temperature to continue to rese above TC_MAX?

[1] The Q10 effect is the factor by which a physiological process changes with a 10 degree change in body temperature from a reference temperature. The factor is calculated as Q10mult=Q10^((mean(TCs)-TC_REF)/10) where TC_REF is the mean of the user input TC_RESTs. Body part-specific morphological parameters and TCs are the current values being used of core temperatures in solving the heat balance.

Next are the morphological parameters per body part.

Name	Units	Head	Trunk	Arm	Leg	Description
DENSITYs	kg/m^3	1050	1050	1050	1050	Density of body
MASSFRACs	-	0.0761	0.501	0.049	0.162	Mass fraction
AREAFRACs	-	0.083	0.327	0.110	0.185	Subcutaneous fat fraction
SHAPE_Bs	-	1.6	1.9	12.0	7.0	Body shape factor B
PJOINs	-	0.0267	0.0809	0.0200	0.0333	Fraction of part surface joined to body
SUBQFATs	-	1	1	1	1	Subcutaneous fat indicator
FATPCTs	%	3.5	25.2	7.0	16.1	Fat percentage
FGDREFs	-	0.35	0.35	0.35	0.35	Ground radiation configuration factor
FSKREFs	-	0.40	0.35	0.35	0.35	Sky radiation configuration factor
heights	m	NA	NA	NA	NA	Height of mid-point of each body part
EMISANs	-	0.95	0.95	0.95	0.95	Emissivity each body part

These are the part-specific physiological parameters.

Name	Units	Head	Trunk	Arm	Leg	Description
TC_RESTs	°C	36.8	36.8	36.5	36.7	Resting target core temperature
TC_ACTIVESs	°C	37.5	37.5	37.5	37.5	Active target core temperature
TC_INCs	°C	0.05	0.05	0.05	0.05	Core temperature increment
TC_MAXs	°C	38	38	38	38	Maximum voluntary core temperature
KFLESHs	$W/m \cdot K$	1.1	0.9	0.5	0.5	Flesh thermal conductivity
KFLESH_MAXs	$W/m \cdot K$	5	5	5	5	Maximum flesh thermal conductivity
KFLESH_INCs	-	0.05	0.05	0.05	0.05	Flesh conductivity increment factor
KFATs	-	0.23	0.23	0.23	0.23	Fat thermal conductivity
PCTWETs	%	1	1	1	1	Skin wetness
PCTWET_INCs	%	1	1	1	1	Skin wetness increment
PCTWET_MAXs	%	100	100	100	100	Maximum skin wetness

Name	Units	Head	Trunk	Arm	Leg	Description
PCTBAREVAPs	%	60	0	0	0	Bare evaporation area
CLOWETs	%	0	0	0	0	Clothing surface wetness percentage

[1] Using the Harris–Benedict equation modified by Mifflin et al. (1990), for women: QMETAB_REST = (10 * weight in kg) + (6.25 * height in cm) - (5 × age in years) - 161 and for men: QMETAB_REST = (10 × weight in kg) + (6.25 * height in cm) - (5 × age in years) + 5 in units of kcal/day, so multiply by 1000 (kcal to cal), 4.184 (cal to J) and divide by 86400 (days to seconds) to get QMETAB in Watts.

Finally, we have the insulation-related parameters per body part.

Name	Units	Head	Trunk	Arm	Leg	Description
KCLOs	$W/m \cdot K$	0	0	0	0	Hair/clothing thermal conductivity
DHAIRDs	m	7.5e-5	1e-6	1e-6	1e-6	Hair/fibre diameter (D)
DHAIRVs	m	7.5e-5	1e-6	1e-6	1e-6	Hair/fibre diameter (V)
LHAIRDs	m	50e-3	50e-3	50e-3	50e-3	Hair/fibre length (D)
LHAIRVs	m	1e-9	50e-3	50e-3	50e-3	Hair/fibre length (V)
INSDEPDs	m	1e-2	6e-3	6e-3	6e-3	Insulation depth (D)
INSDEPVs	m	1e-9	6e-3	6e-3	6e-3	Insulation depth (V)
INSDENDs	$\#/m^3$	3e8	3e8	3e8	3e8	Hair/fibre density (D)
INSDENVs	$\#/m^3$	3e5	3e8	3e8	3e8	Hair/fibre density (V)

Example output for the default environment and parameters

There are two HomoTherm functions, 'HomoTherm' which is for the simulation of a single environment and 'HomoTherm_var' that can be used to simulate across an environmental sequence.

We will first look at the output from the simulation of a single environment (i.e., 'HomoTherm' function) with the default settings: an indoor environment (air and radiant temperatures equal) with 21 °C air temperature, 0.1 m/s wind speed, 50% relative humidity, for a 70 kg person at complete rest wearing the equivalent of 1 clo in insulation (basal metabolic rate is 105 W).

```
HomoTherm.out <- HomoTherm()
```

The results are returned as a list of tables, here called **HomoTherm.out**.

The main output is **balance** which gives outputs for the whole body, including the full partitioning of heat fluxes.

```
balance <- HomoTherm.out$balance
knitr::kable(t(balance[1:6]), digits = 3)
```

T_CORE	T_LUNG	T_SKIN	T_CLO	PCTWET	K_FLESH
36.8	35.908	32.046	24.53	1	0.681

```
knitr::kable(t(balance[7:12]), digits = 3)
```

EVAP_CUT_L	EVAP_RESP_L	SWEAT_L	QMETAB	QSLR	QRAD_IN
0.007	0.013	0.007	108.089	0	641.662

```r
knitr::kable(t(balance[13:18]), digits = 3)
```

QRAD_OUT	QCONV_RESP	QEVAP_RESP	QEVAP_CUT	QCONV	AREA
672.472	-2.152	-8.841	-4.74	-69.878	2.001

The variables in **balance** are:

- **T_CORE** - core body temperature (°C)
- **T_LUNG** - lung temperature (°C)
- **T_SKIN** - skin temperature (°C)
- **T_CLO** - hair/insulation temperature (°C)
- **PCTWET** - skin wettedness (%)
- **K_FLESH** - flesh thermal conductivity (W/m^2)
- **EVAP_CUT_L** - cutaneous water loss via evaporation (L/h)
- **EVAP_RESP_L** - respiratory water loss (L/h)
- **SWEAT_L** - sweat rate (including dripping) (L/h)
- **QMETAB** - metabolic heat production (W)
- **QSLR** - solar heat gain (W)
- **QRAD_IN** - infrared radiation gain (W)
- **QRAD_OUT** - infrared radiation loss (W)
- **QCONV_RESP** - convective heat gain from respiration (W)
- **QEVAP_RESP** - evaporative heat gain from respiration (W)
- **QEVAP_CUT** - evaporative heat gain from skin (W)
- **QCONV** - convective heat gain (W)
- **AREA** - body surface area (including insulation) (m^2)

The other whole-body output is **respire** which reports the computed air and oxygen flow through the lungs.

```r
respire <- HomoTherm.out$respire
knitr::kable(respire, digits = 3)
```

AIR_L	O2_L	O2_mol_in	O2_mol_out	AIR_mol_in	AIR_mol_out
394.704	20.667	3.688	2.766	17.61	17.425

The variables in **respire** are:

- **AIR_L** - air flow, L/h
- **O2_L** - oxygen flow at standard temperature and pressure, L/h
- **O2_mol_in** - oxygen flow in, mol/h
- **O2_mol_out** - oxygen flow out, mol/h
- **AIR_mol_in** - oxygen flow in, mol/h
- **AIR_mol_out** - air flow out, mol/h

The body part specific outputs are also available, with a separate table for the thermoregulatory state of each body part, for morphology and for energy balance. To get these tables for the trunk, for example:

```r
trunk.treg <- HomoTherm.out$trunk.treg
trunk.enbal <- HomoTherm.out$trunk.enbal
trunk.morph <- HomoTherm.out$trunk.morph
```

The **treg** outputs for each body part include the following variables:

```r
knitr::kable(t(trunk.treg[1:5]), digits = 3)
```

T_CORE	TSKIN_D	TSKIN_V	TCLO_D	TCLO_V
36.8	30.511	31.396	23.785	24.069

```r
knitr::kable(t(trunk.treg[6:10]), digits = 3)
```

PCTWET	K_FLESH	K_CLO_D	K_CLO_V	Q10
1	0.9	0.041	0.041	2

- **T_CORE** - core body temperature (°C)
- **T_SKIN_D** - skin temperature, dorsal (°C)
- **T_SKIN_V** - skin temperature, ventral (°C)
- **T_CLO_D** - hair/insulation temperature, dorsal (°C)
- **T_CLO_V** - hair/insulation temperature, ventral (°C)
- **PCTWET** - skin wettedness (%)
- **K_FLESH** - flesh thermal conductivity, $W/m \cdot K$
- **K_CLO_D** - insulation thermal conductivity, dorsal, $W/m \cdot K$
- **K_CLO_V** - insulation thermal conductivity, ventral, $W/m \cdot K$
- **Q10** - Q10 effect on metabolic rate, -

The **enbal** outputs for each body part include the following variables:

```r
knitr::kable(t(trunk.enbal), digits = 3)
```

QMETAB	QSLR	QRAD_IN	QRAD_OUT	QEVAP	QCONV	ENB	NTRY	SUCCESS
18.063	0	213.679	222.058	1.052	12.227	-2.9	1	1

- **QMETAB** - metabolic heat production (W)
- **QSLR** - solar heat gain (W)
- **QRAD_IN** - infrared radiation gain (W)
- **QRAD_OUT** - infrared radiation loss (W)
- **QEVAP** - evaporative heat gain (W)
- **QCONV** - convective heat gain (W)
- **ENB** - the total heat balance (W)
- **NTRY** - how many iterations needed to find a solution, -
- **SUCCESS** - indicator of whether a solution was found (1) or not (0)

The **morph** outputs for each body part include the following variables:

```r
knitr::kable(t(trunk.morph[1:7]), digits = 3)
```

MASS	AREA	VOLUME	CHAR_DIMENSION	MASS_FAT	FAT_THICK	FLESH_VOL
35.049	0.641	0.033	0.294	8.832	0.013	0.025

```r
knitr::kable(t(trunk.morph[8:13]), digits = 3)
```

LENGTH	WIDTH	HEIGHT	R_SKIN	R_INS	AREA_SILHOUETTE
0.547	0.294	0.294	0.141	0.147	0.114

```r
knitr::kable(t(trunk.morph[14:19]), digits = 3)
```

AREA_SKIN	AREA_SKIN_EVAP	AREA_CONV	AREA_JOIN	F_SKY	F_GROUND
0.599	0.598	0.589	0.052	0.42	0.42

- **MASS** - mass (kg)
- **AREA** - area (m^2)
- **VOLUME** - volume (m^3)
- **CHAR_DIMENSION** - characteristic dimension for convection (m)
- **MASS_FAT** - mass of body fat (kg)
- **FAT_THICK** - thickness of fat layer (m^3)
- **FLESH_VOL** - volume of flesh inc. fat layer (m^3)
- **LENGTH** - length (m)
- **WIDTH** - width (m)
- **HEIGHT** - height (m)
- **R_SKIN** - radius from core to skin (m)
- **R_INS** - radius from core to insulation (m)
- **AREA_SILHOUETTE** - silhouette area (m^2)
- **AREA_SKIN** - skin surface area (m^2)
- **AREA_SKIN_EVAP** - area for evaporative heat exchange (m^2)
- **AREA_CONV** - area for convective heat exchange (m^2)
- **AREA_JOIN** - area joined to another body part (m^2)
- **F_SKY** - radiation configuration factor to sky (-)
- **F_GROUND** - radiation configuration factor to ground (-)

Plotting and adjusting the person's dimensions

To help assess the realism of the model, a function 'plot_human' is included that plots the geometry of the person and their insulation in saggital, coronal and transverse sections. The 'plot_human' function requires the height, mass, mass fractions per body part, density, fat presence and percent, and hair/clothing depth, and ratios of long/short axes of parts ('SHAPE_Bs'). These are the default values for the HomoTherm functions:

```r
MASS <- 70 # kg
HEIGHT <- 170 # cm
INSDEPDs <- c(1e-02, rep(6e-03, 3)) # m
```

```
INSDEPVs <- c(1e-09, rep(6e-03, 3)) # m
FATPCTs <- c(5, 36, 10, 23) * 0.7 # %
MASSFRACs <- c(0.076, 0.501, 0.049, 0.162) # -
SHAPE_Bs <- c(1.6, 1.9, 12, 7.0) # -, default values
```

These values can be passed to the 'plot_human' function to see how the model person looks.

```
plot_human(MASS = MASS,
           HEIGHT = HEIGHT,
           INSDEPDs = INSDEPDs,
           INSDEPVs = INSDEPVs,
           FATPCTs = FATPCTs,
           SHAPE_Bs = SHAPE_Bs)
```

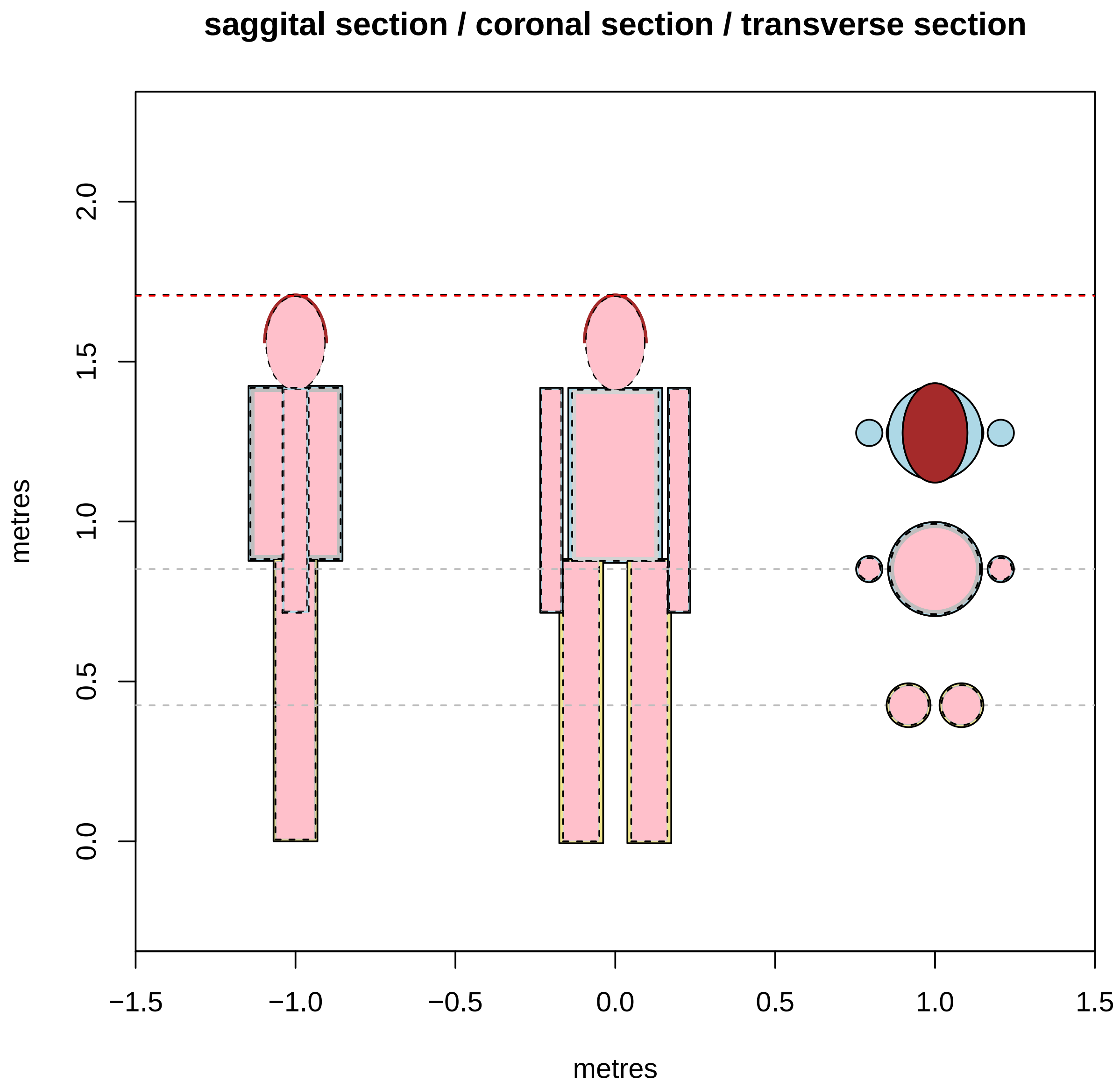

```
## [1] 1.708793
```

The surface area of a human is of course critical for the heat budget calculations. The skin surface area is often calculated using the DuBois formula. For the default case above:

```
AREA_DuBois <- 0.00718 * MASS ^ 0.425 * HEIGHT ^ 0.725 # DuBois area, m2
AREA_DuBois
```

```
## [1] 1.8087
```

From the output of the simulation above the area of the simulated human in m^2, based on the combined areas of all the body parts, is:

```
HomoTherm.out$balance[18]
```

```
##     AREA
## 2.001349
```

That area includes clothing. Without clothing the result is:

```
HomoTherm(INSDEPDs = c(1e-02, rep(0, 3)), INSDEPVs = rep(0, 4))$balance[18]
```

```
##    AREA
## 1.80498
```

```
plot_human(MASS = MASS, HEIGHT = HEIGHT, SHAPE_Bs = SHAPE_Bs,
           FATPCTs = FATPCTs, INSDEPDs = rep(0, 4),
           INSDEPVs = rep(0, 4))
```

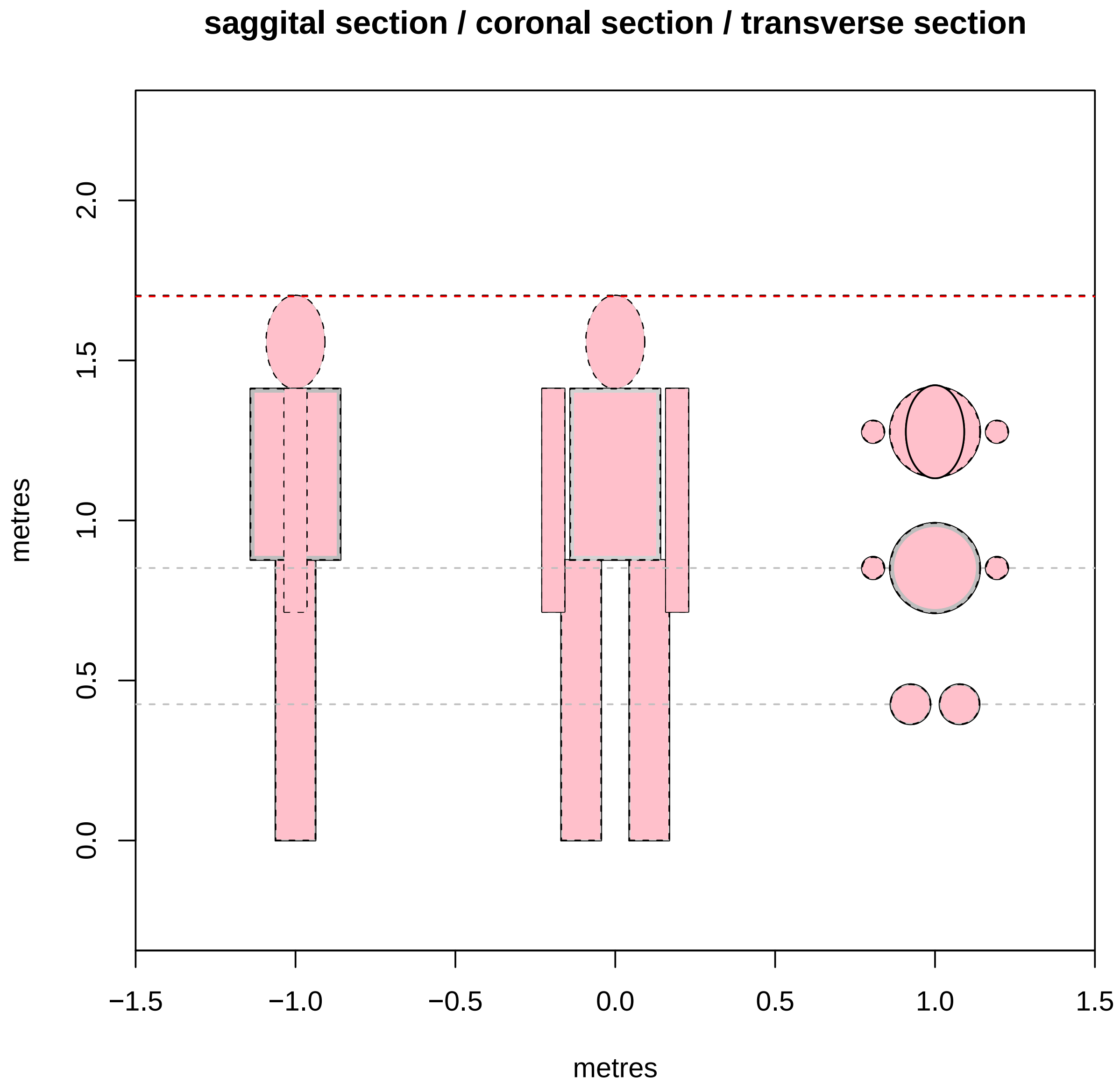


```
## [1] 1.702793
```

Thus the DuBois predicted area of 1.809 m is very close to the value of 1.805 that is obtained for the multi-part geometry of HomoTherm.

Plotting the core to skin/clothing gradient

The temperature gradients across flesh, fat and insulation can be calculated from the output of the model. The 'endoR' model underlying the HomoTherm model assumes distributed (uniform) heat generation in the flesh. The formula for this for a cylindrical geometry is:

$$T_s = T_c - Q_{gen,net}\frac{R_g^2}{4k_g V_g} + \frac{R_g^2}{2k_i V_g}\ln\left(\frac{R_s}{R_g}\right) \quad (1)$$

where T_c and T_s are core and skin temperature, respectively, subscript g means flesh (heat generating tissue), subscript i means fat (insulating tissue), subscript s means skin surface, k is the thermal conductivity (units $\frac{\mathrm{W}}{\mathrm{m}^\circ\mathrm{C}}$), and R is the radial dimension (m). The result is a nonlinear change in temperature from core to the skin.

The gradient and the subsequent gradients within the fat and insulation layers can be calculated knowing the metabolic rate, skin temperature and clothing temperature from the model. The following is an example for the trunk based on the output of the simulation with default parameters:

```
# get output from simulation
par(oma = c(4, 2, 2, 2) + 0.1) # margin spacing
par(mar = c(4, 4, 1, 1) + 0.1) # margin spacing
par(mgp = c(3, 1, 0) ) # margin spacing
trunk.treg <- as.data.frame(t(HomoTherm.out$trunk.treg))
trunk.enbal <- as.data.frame(t(HomoTherm.out$trunk.enbal))
trunk.morph <- as.data.frame(t(HomoTherm.out$trunk.morph))

k_fat <- 0.23 # W/mK, fat conductivity, assumed in HomoTherm
k_flesh <- trunk.treg$K_FLESH # flesh conductivity
T_core <- trunk.treg$T_CORE # core temperature
T_skin <- (trunk.treg$TSKIN_D + trunk.treg$TSKIN_V) * 0.5
T_clo <- (trunk.treg$TCLO_D + trunk.treg$TCLO_V) * 0.5
R_skin <- trunk.morph$R_SKIN
R_clo <- trunk.morph$R_INS
V_gen <- trunk.morph$FLESH_VOL
R_gen <- R_skin - trunk.morph$FAT_THICK
QGEN <- trunk.enbal$QMETAB
T_s <- T_core - (QGEN * R_gen ^ 2) / (4 * k_flesh * V_gen) -
  ((QGEN * R_gen ^ 2) / (2 * k_fat * V_gen)) * log((R_skin / R_gen))

# sequence of distances
R_core2fat <- seq(0, R_gen, 0.002)
# get temp at flesh/fat interface
T_fat <- T_skin + ((QGEN * R_gen ^ 2 ) / (2 * k_fat * V_gen)) *
  log((R_skin / (R_gen)))
# gradient from core to flesh/fat interface
T_core2fat <- T_fat + (QGEN * (R_gen ^ 2 - R_core2fat ^ 2)) / (4 * k_flesh * V_gen)

# general equation for the gradient in insulation
get.gradient <- function(R, R_i, R_o, T_i, T_o){
  log(R/R_i) / log(R_o/R_i) * (T_o - T_i) + T_i
}

# fat to skin
R_fat2skin <- seq(R_gen, R_skin, 0.001)
T_fat2skin <- get.gradient(R_fat2skin, R_gen, R_skin, T_fat, T_skin)
# skin to clothing
R_skin2clo <- seq(R_skin, R_clo, 0.001)
T_skin2clo <- get.gradient(R_skin2clo, R_skin, R_clo, T_skin, T_clo)
```

```
plot(R_core2fat, T_core2fat, xlim = c(0, R_clo), type = 'l',
     ylim = c(min(T_clo), 40), xlab = 'radius, m', ylab = expression("temperature, "*degree*C))
points(R_fat2skin, T_fat2skin, type = 'l', pch = 16)
points(R_skin2clo, T_skin2clo, type = 'l', pch = 16)
points(c(0, R_gen, R_skin, R_clo), c(T_core, T_fat, T_skin, T_clo),
       col = 2, pch = 16)
abline(h = 36.8, lty = 2, col = 'red')
text(c(0, 0.07, 0.13, R_skin + 0.006, 0.135, R_clo - 0.005), c(T_core + 1, 35, 31.9, T_skin, 27.5, T_cl
```

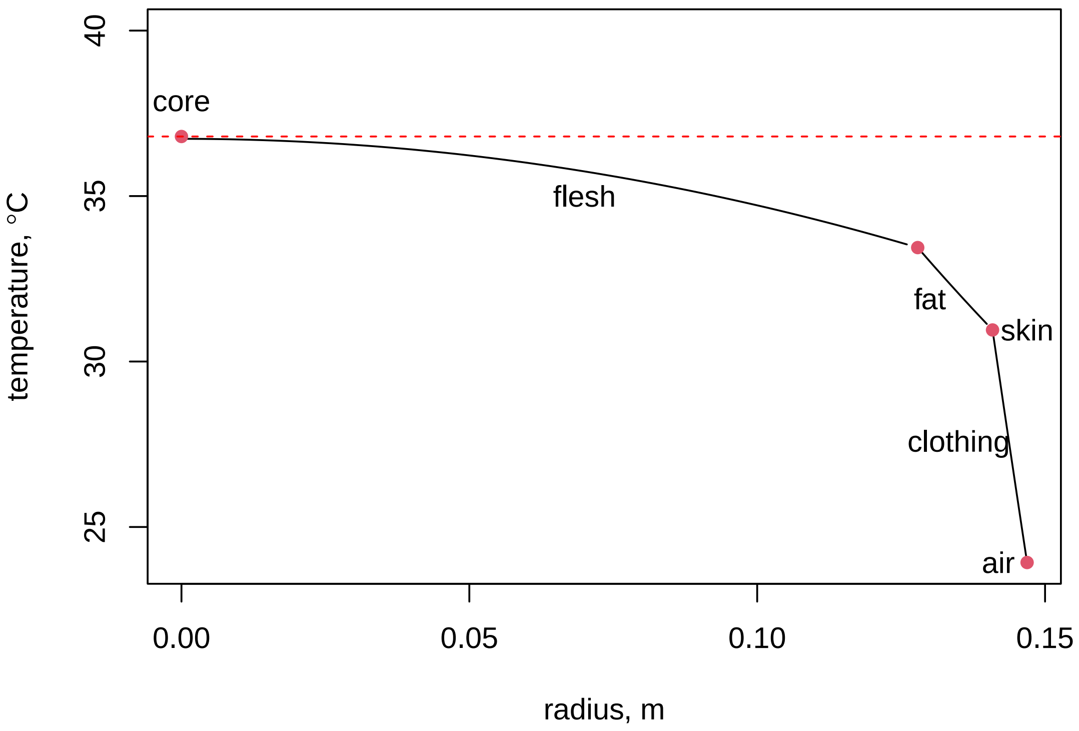

The simulation of different clothing/insulation types and computation of 'clo' values

Hair and clothing insulation can be simulated in two ways with the 'endoR' and hence the 'HomoTherm' model. In the first approach, a thermal conductivity value can be specified directly for each body part via the parameter 'KCLOs' together with the corresponding insulation depths via 'INSDEPDs' and 'INSDEPVs'.

The second approach is to specify the diameter, length and density of the hairs/fibres of the insulation (via 'DHAIRDs/DHAIRVs', 'LHAIRDs/LHAIRVs' and 'INSDENDs/INSDENVs', respectively). From this the model computes the air and fibre densities to get the effective thermal conductivity, as well as computing the contribution of infrared radiation through the insulation to get the final thermal conductivity.

Note that the 'hair' properties can be specified differently for dorsal and ventral e.g. 'INSDEPDs' vs. 'INSDEPVs'), which is a hangover from the 'endoR' model to allow differences in the insulation to be present on

the belly vs. on the back. The approach can be useful to allow the head to have hair but no beard, or to simulate someone lying on bedding.

By default, the second approach is in operation. If a human indoors at 20 °C with 1 cm of hair on their head, and 1 cm deep clothing and the default settings for hair diameter, length and depth, is simulated, the model can work out what that translates to in terms of clothing insulation as follows:

```
INSDEPDs <- c(1e-02, rep(1e-02, 3)) # 'dorsal' clothing depth, m
INSDEPVs <- c(1e-09, rep(1e-02, 3)) # 'ventral' clothing depth, m
HomoTherm.out <- HomoTherm(TA = 20,
                           VEL = 0.1,
                           RH = 50,
                           INSDEPDs = INSDEPDs,
                           INSDEPVs = INSDEPVs)
trunk.treg <- HomoTherm.out$trunk.treg
knitr::kable(t(trunk.treg[1:5]), digits = 3)
```

T_CORE	TSKIN_D	TSKIN_V	TCLO_D	TCLO_V
36.8	32.402	32.915	23.702	23.862

```
knitr::kable(t(trunk.treg[6:10]), digits = 3)
```

PCTWET	K_FLESH	K_CLO_D	K_CLO_V	Q10
2	1.35	0.051	0.051	2

The calculated thermal conductivity is reported in the *.treg output tables (* standing for body part). Thus, for the trunk, we get 0.051 $W/m \cdot K$ in this case.

Alternatively, if the first approach is used, the 'KCLOs' can be specified:

```
INSDEPDs <- c(1e-02, rep(1e-02, 3)) # 'dorsal' clothing depth, m
INSDEPVs <- c(1e-09, rep(1e-02, 3)) # 'ventral' clothing depth, m
KCLOs <- rep(0.04, 4) # clothing thermal conductivity, W/m·K
HomoTherm.out <- HomoTherm(TA = 20,
                           VEL = 0.1,
                           RH = 50,
                           INSDEPDs = INSDEPDs,
                           INSDEPVs = INSDEPVs,
                           KCLOs = KCLOs)
trunk.treg <- HomoTherm.out$trunk.treg
knitr::kable(t(trunk.treg[1:5]), digits = 3)
```

T_CORE	TSKIN_D	TSKIN_V	TCLO_D	TCLO_V
36.8	33.721	33.989	23.489	23.559

```
knitr::kable(t(trunk.treg[6:10]), digits = 3)
```

PCTWET	K_FLESH	K_CLO_D	K_CLO_V	Q10
4	1.95	0.04	0.04	2

The results for the two approaches are similar but there was some heat stress leading to elevated blood flow (an increase in flesh thermal conductivity) and skin wetness in the second scenario.

The insulation is specified as a thermal *conductivity* in $W/m \cdot K$, i.e., ease of heat movement linearly through the insulation. Thermal *conductance* is conductivity per surface area, i.e. $W/m^2 \cdot K$. The inverse of conductance is resistance. The 'clo' unit, that is frequently used in human thermal physiology, is equal to a thermal resistance of 0.155 $m^2 \cdot K/W$. Thus, to get from thermal conductivity to clo units the insulation depth is divided by the insulation conductivity multiplied by 0.155 $m^2 \cdot K/W$. So for the last example, for the trunk:

```
INSDEPDs[2] / (0.155 * trunk.treg[8])
```

```
##  K_CLO_D
## 1.612903
```

i.e., 1.6 clo units. One clo is the insulation required for a typical sized male to be comfortable at rest at 21 °C with relative humidity less than 50% and air movement less than 1 m/s. Thus to get down to this value of clo with clothing of the same thermal conductivity in the previous example we need the insulation depth to be 6.15 mm deep:

```
INSDEPDs <- c(1e-02, rep(6.15e-03, 3)) # 'dorsal' clothing depth, m
INSDEPVs <- c(1e-09, rep(6.15e-03, 3)) # 'ventral' clothing depth, m
KCLOs <- rep(0.04, 4) # clothing thermal conductivity, W/m·K
HomoTherm.out <- HomoTherm(TA = 20,
                      VEL = 0.1,
                      RH = 50,
                      INSDEPDs = INSDEPDs,
                      INSDEPVs = INSDEPVs,
                      KCLOs = KCLOs)
trunk.treg <- HomoTherm.out$trunk.treg
knitr::kable(t(trunk.treg[1:5]), digits = 3)
```

T_CORE	TSKIN_D	TSKIN_V	TCLO_D	TCLO_V
36.8	31.583	32.228	24.109	24.34

```
knitr::kable(t(trunk.treg[6:10]), digits = 3)
```

PCTWET	K_FLESH	K_CLO_D	K_CLO_V	Q10
1.5	1.2	0.04	0.04	2

```
INSDEPDs[2] / (0.155 * trunk.treg[8])
```

```
##   K_CLO_D
## 0.9919355
```

To get the overall clo value for the person from the HomoTherm output, the NicheMapR function 'get_clo' can be used as follows:

```
get_clo(HomoTherm.out,
        INSDEPDs = INSDEPDs,
        INSDEPVs = INSDEPVs)
```

```
##        clo
## 0.9765554
```

And, from the metabolic state of the person, they would indeed be comfortable with 1 clo of insulation at rest in this environment (QMETAB is near QMETAB_REST = 106 W).

```
balance <- HomoTherm.out$balance
knitr::kable(t(balance[1:6]), digits = 3)
```

T_CORE	T_LUNG	T_SKIN	T_CLO	PCTWET	K_FLESH
36.8	36.074	33.338	25.135	1.5	0.981

```
knitr::kable(t(balance[7:12]), digits = 3)
```

EVAP_CUT_L	EVAP_RESP_L	SWEAT_L	QMETAB	QSLR	QRAD_IN
0.012	0.013	0.012	105.756	0	634.9

```
knitr::kable(t(balance[13:17]), digits = 3)
```

QRAD_OUT	QCONV_RESP	QEVAP_RESP	QEVAP_CUT	QCONV
679.27	-2.27	-8.748	-8.013	-50.495

Simulating a varying sequence of environments

The 'HomoTherm_var' function loops through a sequence of environmental conditions and runs the 'HomoTherm' function for each set of conditions in the sequence. For example, the function can be used to calculate the thermoneutral zone for a human across a range of air temperatures as follows (with the insulation specified as 1 clo, based on the settings in the previous section). Note that the lower critical temperature 'LCT' is defined as when flesh thermal conductivity starts to increase (KFLESH variable) and the upper critical temperature (UCT) is defined somewhat arbitrarily as when skin wetness exceeds 2% - note that the UCT is less well defined in humans than in other mammals, see Pallubinsky et al. (2019).

```
# plot TNZ
TAs <- seq(0, 55, 1) # sequence of air temperatures, °C
VELs <- rep(0.15, length(TAs)) # keep wind speeds constant, m/s
RHs <- rep(50, length(TAs)) # keep humidity constant, %

# simulate across the air temperatures
HomoTherm.out <- HomoTherm_var(INSDEPDs = INSDEPDs,
```

```
                                INSDEPVs = INSDEPVs,
                                TAs = TAs,
                                VELs = VELs,
                                RHs = RHs,
                                EXCEED.TCMAX = TRUE)
balance <- HomoTherm.out$balance
T_lethal <- 43
Ta_lethal <- TAs[balance$T_CORE > T_lethal][1]
Ta_lethal_box <- Ta_lethal
if(is.na(Ta_lethal)){Ta_lethal_box <- max(TAs)}
LCT <- TAs[which(balance$K_FLESH > balance$K_FLESH[1] * 1.1)[1]-1]
UCT <- TAs[balance$PCTWET > 2][1]
T_crit <- TAs[balance$PCTWET >= 99.9][1]
```

We now plot the thermoneutral zone, percent skin wetness, percent vasodilation (flesh thermal conductivity variable) and the associated body temperatures. Note that the thermoneutral zone (TNZ) is between 19 °C and 23 °C air temperature, water loss plateaus at 45 °C and death occurs at 52 °C air temperature.

```
par(mfrow = c(3, 1))
par(oma = c(4, 2, 2, 2) + 0.1) # margin spacing
par(mar = c(4, 4, 1, 1) + 0.1) # margin spacing
par(mgp = c(3, 1, 0) ) # margin spacing
plot(TAs, balance$QMETAB, type = 'l', col = 'red', lwd = 1.5, ylim = c(0, 300),
     ylab = 'watts', xlab = expression("air temperature, "*degree*C),
     xaxs = 'i', yaxs = 'i')
points(TAs, (balance$QEVAP_RESP + balance$QEVAP_CUT) * -1, type = 'l',
       col = 'blue', lwd = 1.5)
points(TAs, balance$PCTWET, type = 'l', col = 'lightblue', lwd = 1.5)
legend(x = 5, y = 300, legend = c("Q_metab", "Q_evap", "% wet", "% blood flow"),
       col = c("red", "blue", "lightblue", "red"), lty = c(1, 1, 1, 2),
       bty = "n", cex = 0.75)
plot_thresh <- function(thresh){
  usr <- par("usr")  # xmin, xmax, ymin, ymax
  x_coord <- thresh  # X-coordinate for the number
  y_line <- usr[4]  # Top of the plot
  y_coord <- y_line + 0.03 * (usr[4] - usr[3])  # Slightly above the plot
  text(x = x_coord, y = y_coord, labels = thresh, col = "black",
       cex = 0.85, font = 2, xpd = TRUE)
}
plot_thresh(LCT)
plot_thresh(UCT)
plot_thresh(T_crit)
plot_thresh(Ta_lethal)
rect(
  xleft = T_crit, xright = Ta_lethal_box,
  ybottom = par("usr")[1], ytop = par("usr")[4],
  col = rgb(0.5, 0, 0.5, 0.3), # Purple color with 30% opacity
  border = NA               # No border
)
rect(
  xleft = LCT, xright = UCT,
  ybottom = par("usr")[1], ytop = par("usr")[4],
  col = rgb(1, 0.5, 0, 0.3), # Orange color with 30% opacity
```

```
  border = NA              # No border
)
abline(v = Ta_lethal, col = 'black', lwd = 1.5)
points(TAs, balance$K_FLESH / 5 * 100, type = 'l', lty = 2, col = 'red')
text(x = LCT + (UCT - LCT) / 2, y = par("usr")[4] * 0.9, "TNZ")
text(x = T_crit + (Ta_lethal_box - T_crit) / 2, y = par("usr")[4] * 0.9, "LETHAL")

plot(TAs, balance$T_CORE, type = 'l', col = 'red', lwd = 1.5, ylim = c(25, 45),
     ylab = expression("temperature, "*degree*C),
     xlab = expression("air temperature, "*degree*C), xaxs = 'i', yaxs = 'i')
points(TAs, balance$T_SKIN, lwd = 1.5, type = 'l', col = 'orange')
points(TAs, balance$T_CLO, lwd = 1.5, type = 'l', col = 'grey')
legend(x = 5, y = 45, legend = c("core", "skin", "clo"), col =
         c("red", "orange", "grey"), lty = 1, bty = "n", cex = 0.75)
plot_thresh(LCT)
plot_thresh(UCT)
plot_thresh(T_crit)
plot_thresh(Ta_lethal)
rect(
  xleft = T_crit, xright = Ta_lethal_box,
  ybottom = par("usr")[1], ytop = par("usr")[4],
  col = rgb(0.5, 0, 0.5, 0.3), # Red color with 30% opacity
  border = NA              # No border
)
rect(
  xleft = LCT, xright = UCT,
  ybottom = par("usr")[1], ytop = par("usr")[4],
  col = rgb(1, 0.5, 0, 0.3), # Red color with 30% opacity
  border = NA              # No border
)
abline(v = Ta_lethal, col = 'black', lwd = 1.5)
text(x = LCT + (UCT - LCT) / 2, y = par("usr")[4] * 0.95, "TNZ")
text(x = T_crit + (Ta_lethal_box - T_crit) / 2, y = par("usr")[4] * 0.95, "LETHAL")
abline(h = 35)

xmin <- min((balance$T_CORE + balance$T_SKIN) / 2)
plot((balance$T_CORE + balance$T_SKIN) / 2, balance$PCTWET, type = 'l',
     col = 'blue', lwd = 1.5, ylim = c(0, 100), xlim = c(xmin, 40),
     ylab = '%', xlab = 'average T_core/T_skin, °C', xaxs = 'i', yaxs = 'i')
points((balance$T_CORE + balance$T_SKIN) / 2, balance$K_FLESH / 5 * 100,
       lwd = 1.5, type = 'l', col = 'red')
legend(x = xmin, y = 100, legend = c("% wet", "% blood flow"), col =
         c("blue", "red"), lty = 1, bty = "n", cex = 0.75)
```

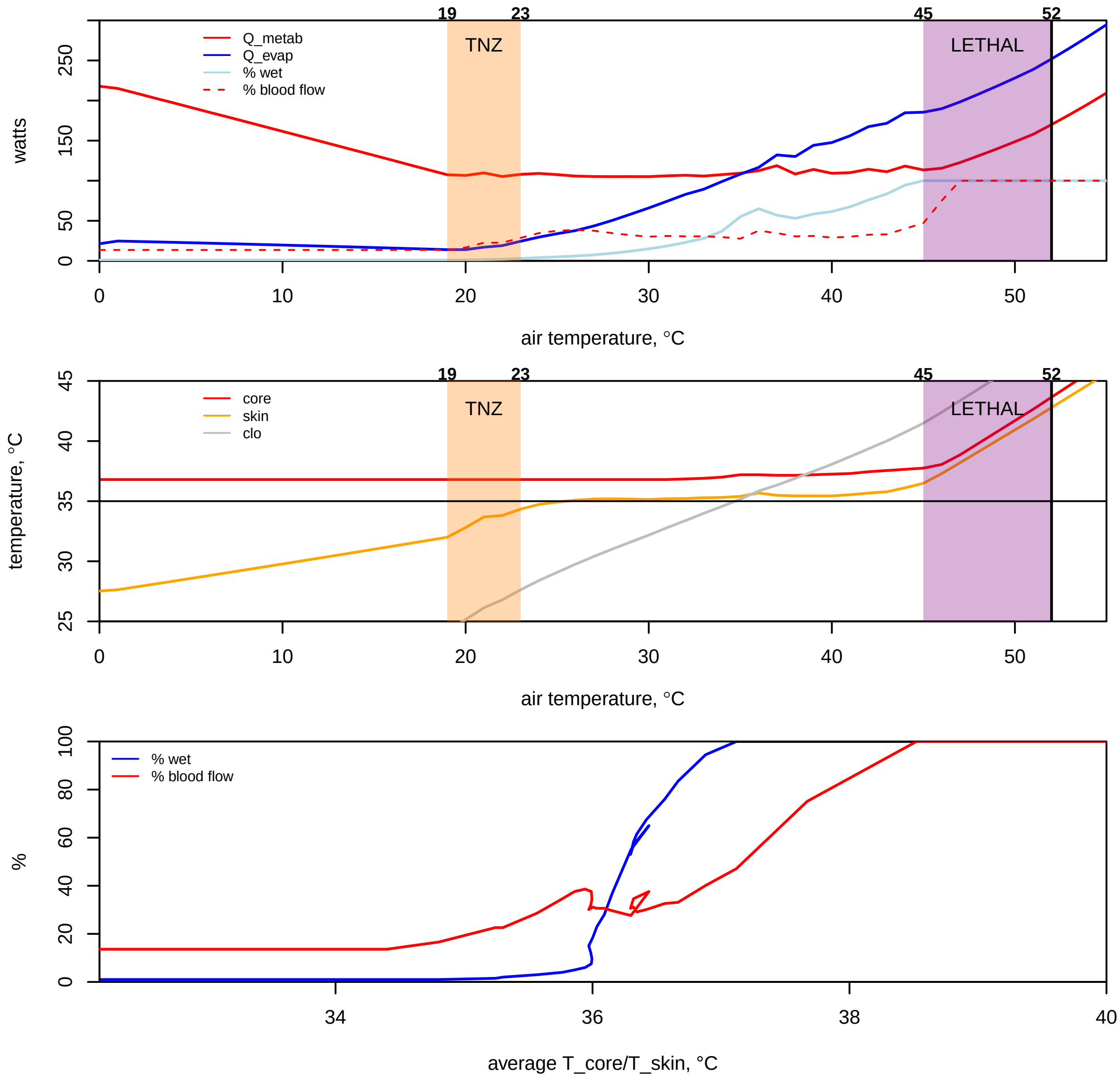

If the same analysis is performed for an unclothed human (changing INSDEPDs and INSDEPVs to zero) a narrower thermoneutral zone of 25 to 29 °C is calculated, water loss plateaus at 44 °C air temperature and the upper lethal limit is 51 °C air temperature.

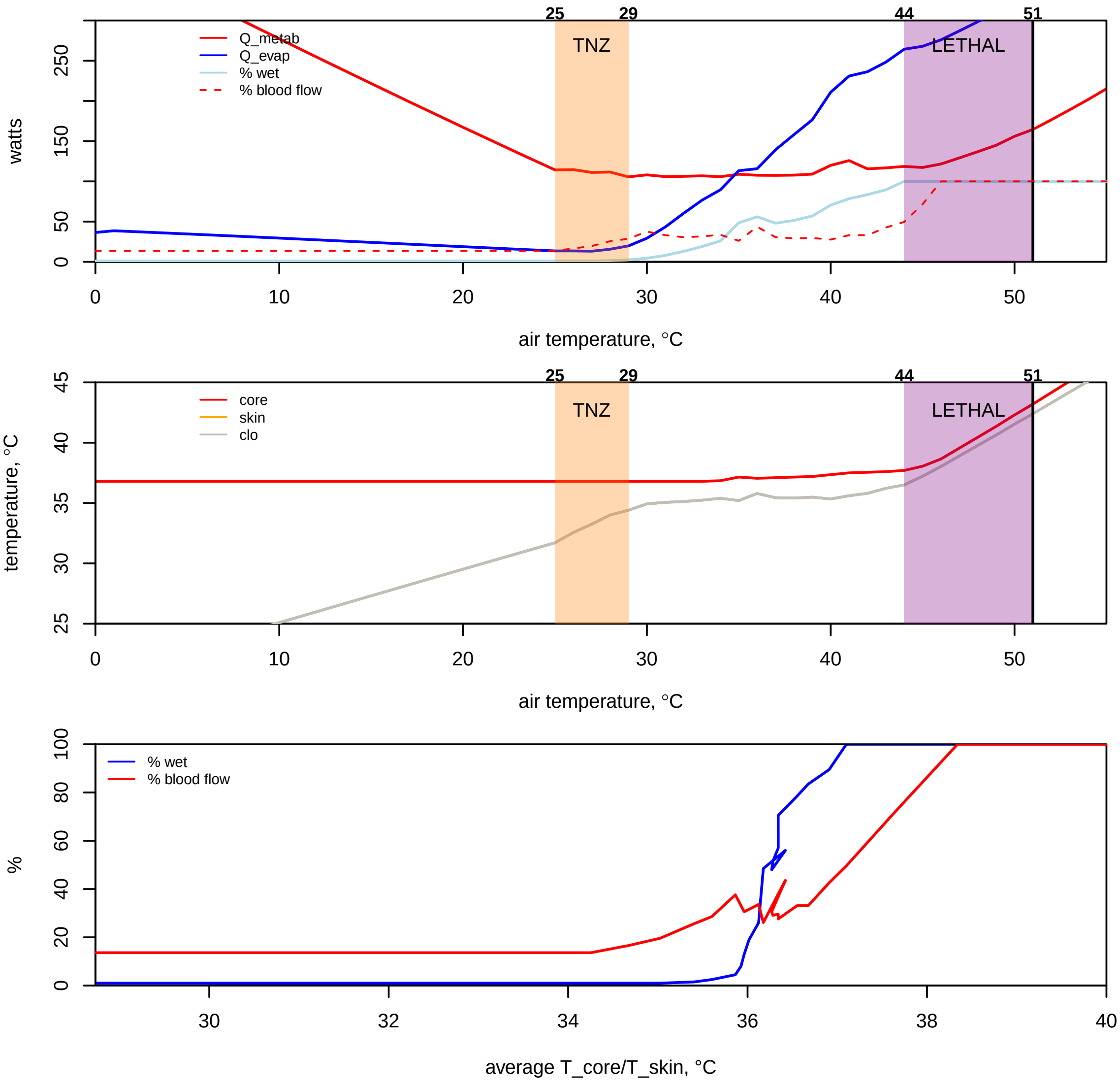

Finally, if the humidity is increased to 100% then the lower critical temperature remains at 25 °C, the upper critical temperature remains at 29 °C and the lethal becomes 40 °C. The air temperature at which evaporative heat loss plateaus is 34 °C.

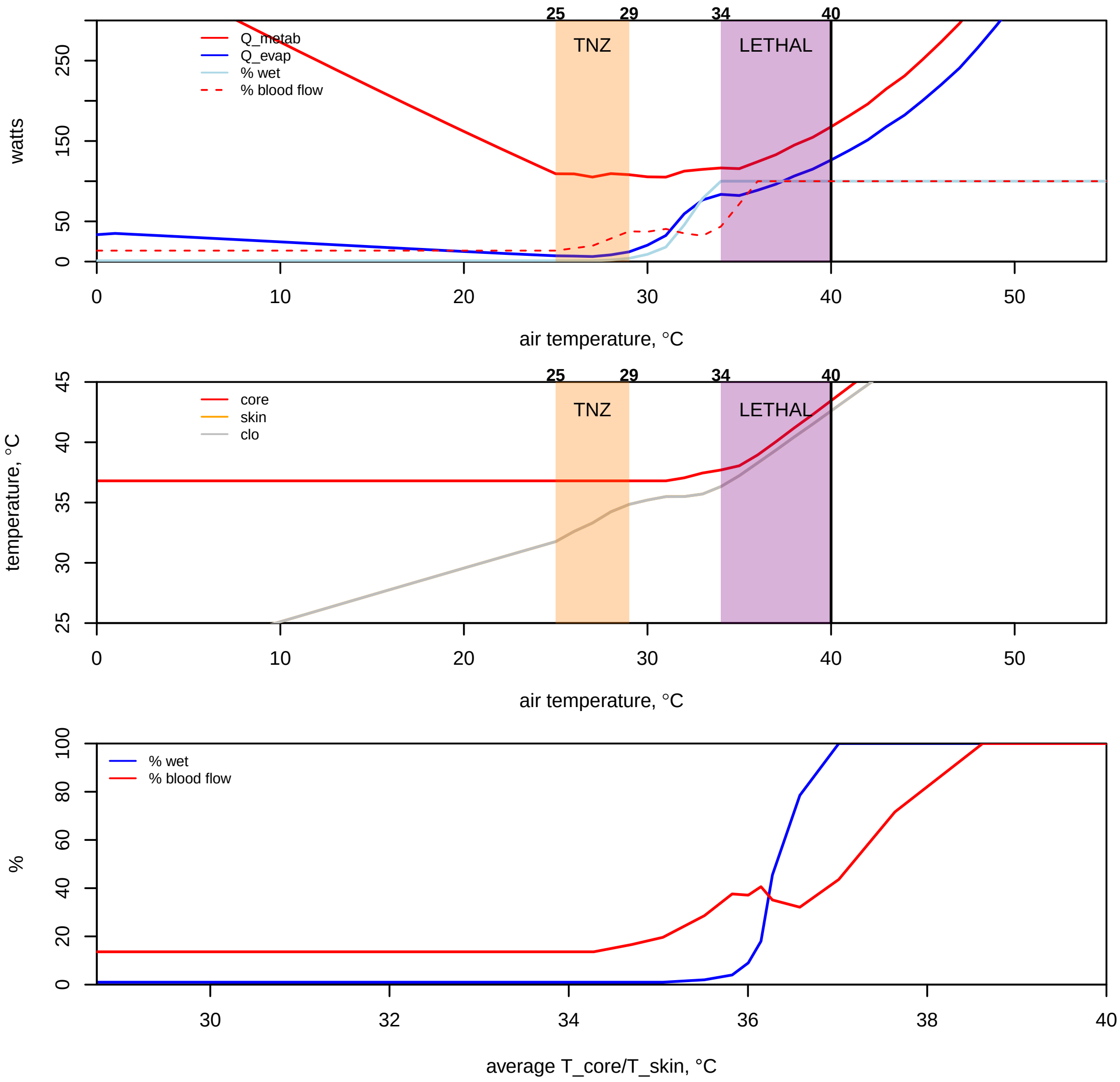

Simulating outdoor environments with the microclimate model

In this final example a human is simulated when standing outdoors in Death Valley, California USA under a typical day based on the from 1960 to 1990 monthly climate averages for each month of the year.

First the microclimate model (Kearney and Porter, 2017; Kearney and Maino, 2019; Kearney 2020) is run using the 'micro_global' function of NicheMapR. Note that the 'microclima' parameter is set to 1 to allow diffuse and direct solar radiation to be computed.

```
# select a location and run the microclimate model
options(timeout = 1900)
Sys.setlocale("LC_CTYPE", "en_US.UTF-8")
```

```
## [1] "en_US.UTF-8"
```

```
Sys.setenv(CURL_CONNECT_TIMEOUT = 1900)
Sys.setenv(CURL_TIMEOUT = 1900)
loc <- c(-116.9325, 36.5323) # Death Valley
dem <- microclima::get_dem(lat = loc[2], long = loc[1], resolution = 30, xdims = 100, ydims = 100)
micro <- NicheMapR::micro_global(loc = loc, microclima = 1, dem = dem)
```

```
## using microclima and elevatr to adjust solar for topographic and vegetation effects

## Downloading digital elevation data
```

The aboveground conditions ('metout' output table) and soil temperatures ('soil' output table) are extracted and the required input variables are assigned.

```
# extract microclimatic conditions
metout <- as.data.frame(micro$metout) # above ground microclimatic conditions, full sun
soil <- as.data.frame(micro$soil) # soil temperatures, full sun
dates <- micro$dates

TAs <- metout$TALOC
RHs <- metout$RHLOC
VELs <- metout$VLOC
VREFs <- metout$VREF
RHREFs <- metout$RH
QSOLRs <- metout$SOLR
Zs <- metout$ZEN
TSKYs <- metout$TSKYC
TGRDs <- soil$D0cm
TBUSHs <- metout$TALOC
TAREFs <- metout$TAREF
SHADEs <- rep(0, length(TAs))
BPs <- rep(101325, length(TAs))
ABSSB <- 1 - micro$REFL # -, substrate absorptivity
PDIFs <- micro$diffuse_frac
CONV_ENHANCE <- 1.4 # turbulent conditions
```

Next the heights above ground are obtained for each body part - the mid point of each part - with the 'get_heights' function. This function is used to make each part experience the height-adjusted air temperature, wind speed and relative humidity.

```
heights <- get_heights(MASSs = MASS * MASSFRACs,
                       HEIGHT = HEIGHT,
                       DENSITYs = rep(1050, 4),
                       SHAPE_Bs = SHAPE_Bs)
round(heights, 2)
```

```
## [1] 1.55 1.14 0.99 0.44
```

```
plot_human()
```

```
## [1] 1.708793
```

```r
points(c(0, 0, 0.21, 0.11), heights, pch = 16)
```

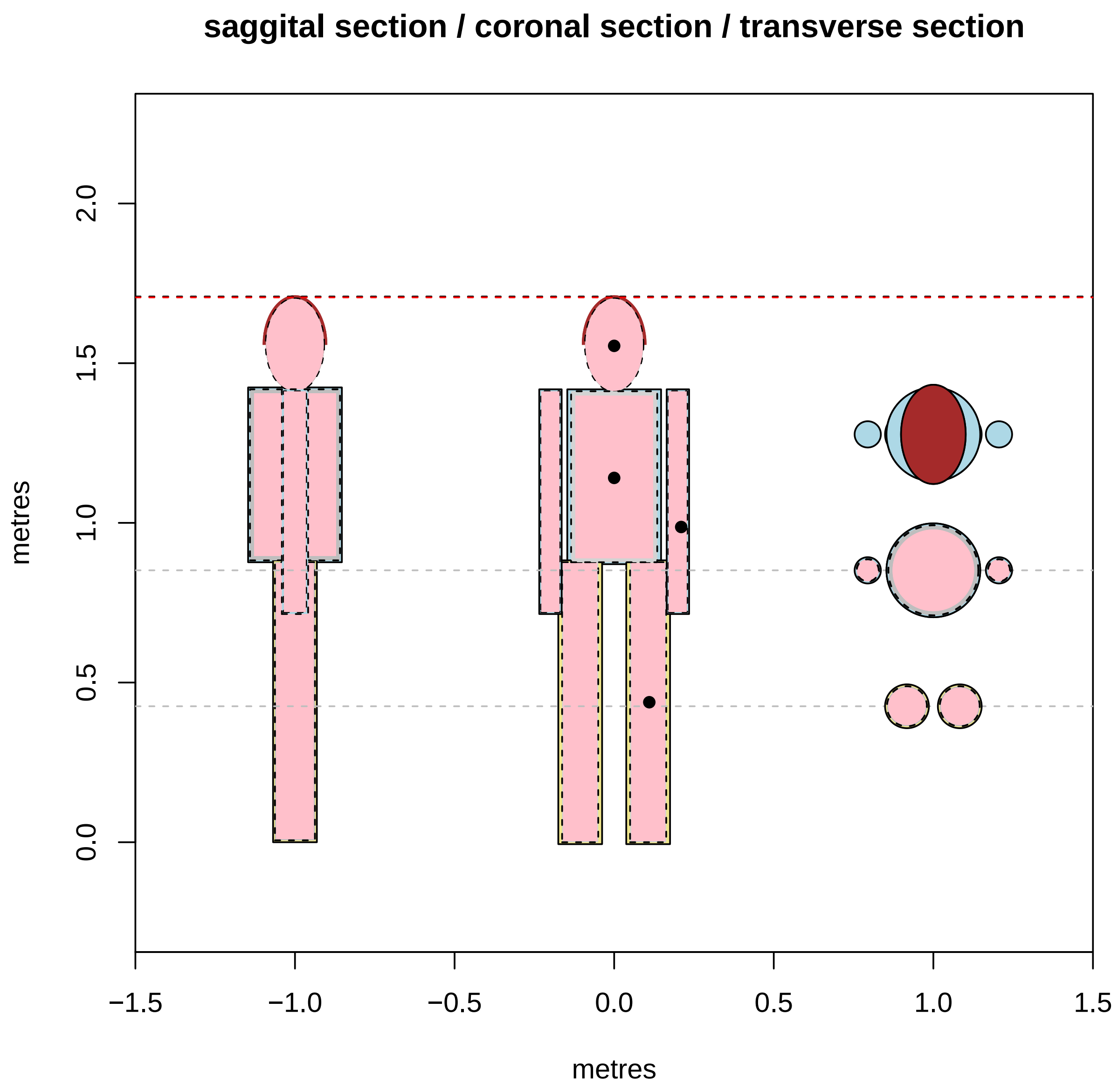

The simulation is run for each hour of each typical day of each of the 12 months.

```r
HomoTherm.out <- HomoTherm_var(INSDEPDs = INSDEPDs,
                               INSDEPVs = INSDEPVs,
                               KCLOs = KCLOs,
                               TAs = TAs,
                               RHs = RHs,
                               VELs = VELs,
                               VREFs = VREFs,
                               RHREFs = RHREFs,
                               QSOLRs = QSOLRs,
```

```
                                Zs = Zs,
                                PDIFs = PDIFs,
                                TSKYs = TSKYs,
                                TGRDs = TGRDs,
                                TAREFs = TAREFs,
                                SHADEs = SHADEs,
                                BPs = BPs,
                                ABSSB = ABSSB,
                                heights = heights,
                                CONV_ENHANCE = CONV_ENHANCE
                                )
balance <- HomoTherm.out$balance
```

The results are plotted for a given month - here July.

```
month <- 7 # 7 = July
par(mfrow = c(2, 1))
par(oma = c(4, 2, 2, 2) + 0.1) # margin spacing
par(mar = c(4, 4, 1, 1) + 0.1) # margin spacing
par(mgp = c(3, 1, 0) ) # margin spacing
subs <- which(ceiling(dates) == month) - 1 # get the hours for the month chosen
plot(seq(0, 23), balance$QMETAB[subs], type = 'l', col = 'red', lwd = 2,
     ylim = c(0, 400), ylab = 'watts', xlab = 'hour of day')
points(seq(0, 23), (balance$QEVAP_RESP + balance$QEVAP_CUT)[subs] * -1,
       type = 'l', col = 'blue', lwd = 2)
points(seq(0, 23), balance$K_FLESH[subs] / 5 * 100, type = 'l', col = 'red',
       lwd = 2, lty = 2)
points(seq(0, 23), balance$PCTWET[subs], type = 'l', col = 'lightblue', lwd = 2)
legend(x = 0, y = 385, legend = c("Q_metab", "Q_evap", "% wet", "% blood flow"),
       col = c("red", "blue", "lightblue", "red"), lty = c(1, 1, 1, 2),
       bty = "n", cex = 0.75)

plot(seq(0, 23), balance$T_CORE[subs], type = 'l', col = 'red', lwd = 2,
     ylim = c(5, 60),
     ylab = expression("temperature, "*degree*C), xlab = 'hour of day')
points(seq(0, 23), balance$T_SKIN[subs], type = 'l', col = 'orange', lwd = 2)
points(seq(0, 23), balance$T_CLO[subs], type = 'l', col = 'grey', lwd = 2)
legend(x = 0, y = 60, legend = c("core", "skin", "clo"),
       col = c("red", "orange", "grey"), lty = 1, bty = "n", cex = 0.75)
```

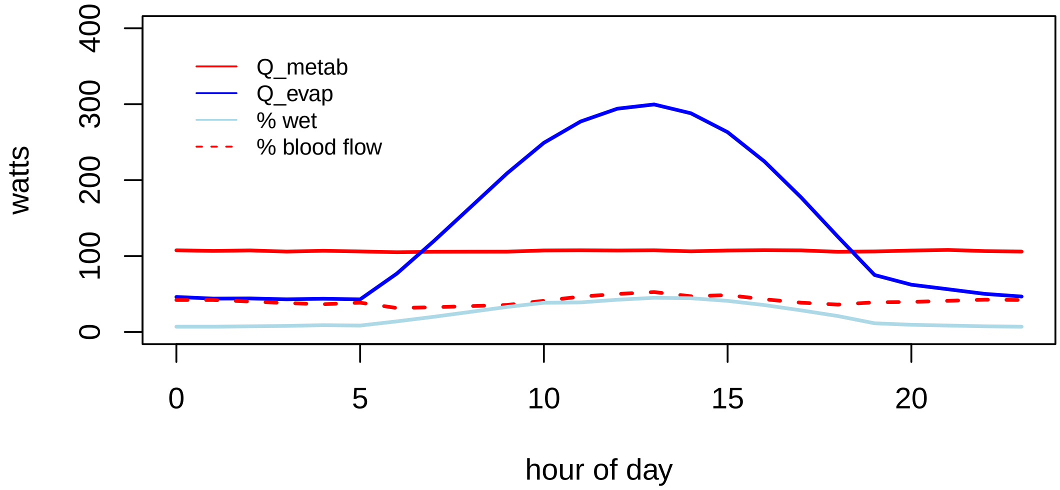

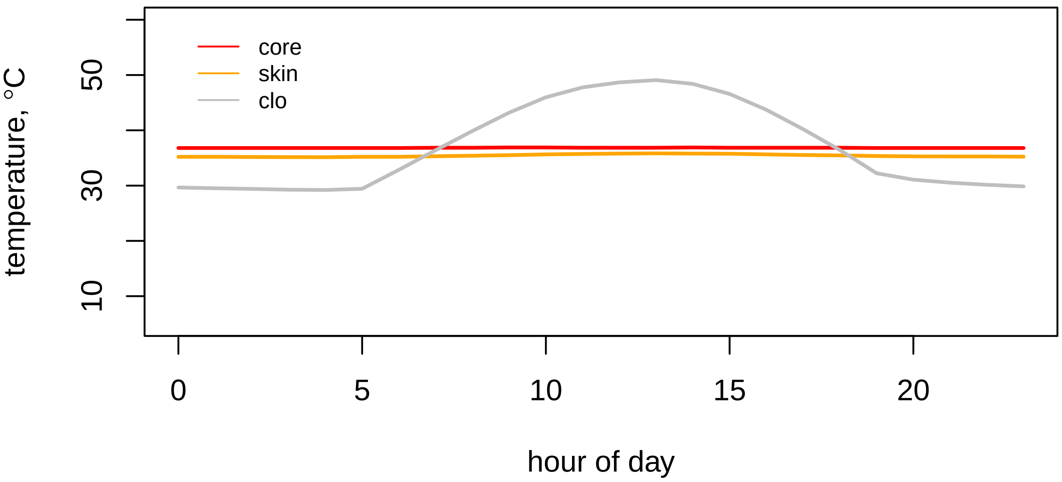

References

Mifflin, M., St Jeor, S., Hill, L., Scott, B., Daugherty, S., & Koh, Y. (1990). A new predictive equation for resting energy expenditure in healthy individuals. The American Journal of Clinical Nutrition, 51(2), 241–247. https://doi.org/10.1093/ajcn/51.2.241

Kearney, M. R., and W. P. Porter. 2017. NicheMapR - an R package for biophysical modelling: the microclimate model. Ecography 40:664–674.

Kearney, M. R., and J. L. Maino. 2018. Can next-generation soil data products improve soil moisture modelling at the continental scale? An assessment using a new microclimate package for the R programming environment. Journal of Hydrology 561:662–673.

Kearney, M. R. 2020. How will snow alter exposure of organisms to cold stress under climate warming? Global Ecology and Biogeography 29:1246–1256.

Kearney, M. R., Mitchell, D., & Maloney, S. K. (in review). HomoTherm: An open-source approach to modelling heat exchange of diverse people in diverse environments.

Kowalski, G. J., and J. W. Mitchell. 1976. Heat Transfer From Spheres in the Naturally Turbulent, Outdoor Environment. Journal of Heat Transfer 98:649–653.

Pallubinsky, H., L. Schellen, and W. D. van Marken Lichtenbelt. 2019. Exploring the human thermoneutral zone – A dynamic approach. Journal of Thermal Biology 79:199–208.

Underwood, C. R., & Ward, E. J. (1966). The Solar Radiation Area of Man. Ergonomics, 9(2), 155–168. https://doi.org/10.1080/00140136608964361
