# Supplementary material for: HomoTherm: An Open‐Source Approach to Modelling Heat Exchange in Humans and Other Hominins in Diverse Environments
Source: Glob Chang Biol. 2026 Apr 1;32(4):e70830. doi: 10.1111/gcb.70830 (PMC13044332; doi:10.1111/gcb.70830)
Supplement: Supplementary file 2 — Appendix S2: gcb70830‐sup‐0002‐Appendix 2.pdf. [file GCB-32-e70830-s010.pdf]

Test of HomoTherm against Erickson 1956

Michael Kearney

2026-01-07

Overview

A test of the model against the data reported in Erickson et al. (1956).

Load the libraries and data

```
library(NicheMapR)
localpath <- 'c:/Users/mrke/Dropbox/Current Research Projects/mammal_projects/manmo analysis/'
source(paste0(localpath, 'code/MANMO/MANMO_R.R')) # the MANMO function
source(paste0(localpath, 'code/MANMO/run.MANMO.R'))
source(paste0(localpath, 'code/HHB/HHB.R'))
source(paste0(localpath, 'code/HHB/run_HHB.R'))
```

Load and plot the Erickson et al. observations

```
Erickson <- read.csv(paste0(localpath, 'data/Erickson/Erickson1956 Fig.1.txt'),
                     skip = 1, head = F)
par(oma = c(2, 2, 1, 2) + 0.1) # margin spacing stuff
par(mar = c(4, 4, 1, 1) + 0.1) # margin spacing stuff
par(mgp = c(3, 1, 0) ) # margin spacing stuff
plot(Erickson, pch = 16, col = 2, ylab = 'W', xlab = 'T_air, deg C',
     xlim = c(-10, 37), ylim = c(0, 600), xaxs = 'i', yaxs = 'i')
axis(1, at = 37)
Erickson_clothed <-
  read.csv(paste0(localpath, 'data/Erickson/Erickson1956 Fig.1_clothed.txt'),
           skip = 1, head = F)
points(Erickson_clothed, pch = 16, col = 'orange', ylab = 'W', xlab = 'T_air, deg C',
       xlim = c(-10, 37),  ylim = c(0, 600))
```

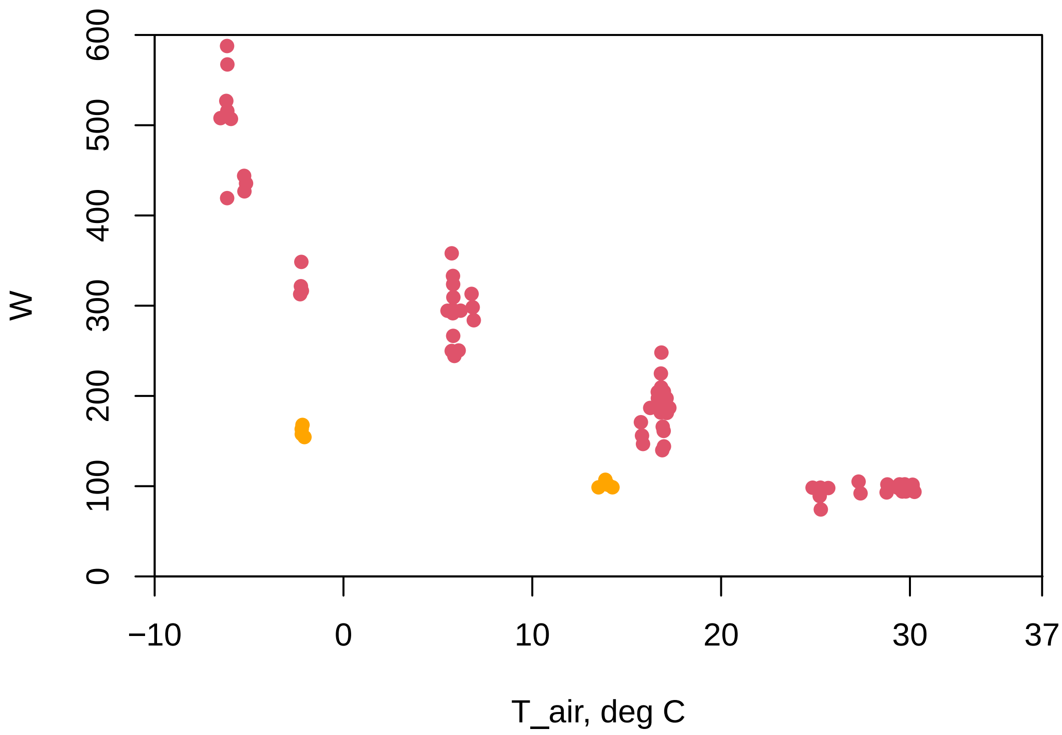

Environmental conditions

```
# environmental variables
TAs1 <- seq(-10, 30, 1)#Erickson$V1 # air temperatures, deg C
TRADs1 <- TAs1 # radiant temperatures, deg C
RHs1 <- rep(55, length(TAs1)) # relative humidities, %
VELs1 <- rep(0.3, length(TAs1))# wind speeds, m/s

TAs2 <- seq(-10, 30, 1)#Erickson_clothed$V1 # air temperatures, deg C
TRADs2 <- TAs1 # radiant temperatures, deg C
RHs2 <- rep(55, length(TAs2)) # relative humidities, %
VELs2 <- rep(0.3, length(TAs2))# wind speeds, m/s
```

Person parameters

```
# person parameters
MASSs <- c(74, 74, 73, 70, 78)
HEIGHTs <- c(176, 170, 173, 179, 190)
AGEs <- c(50, 35, 37, 40, 31)
QMETAB_RESTs <- ((10 * MASSs) + (6.25 * HEIGHTs) - (5 * AGEs) + 5) * 1000 *
  4.184 / 86400
AREAs <- 0.00718 * MASSs ^ 0.425 * HEIGHTs ^ 0.725 # DuBois area, m2
```

```
MASSFRACs <- c(0.0761, 0.501, 0.049, 0.162)
AREAFRACs <- c(0.08291887, 0.32698460, 0.11025155, 0.18479669)
```

```
# first in shorts
INSDEPDs <- c(0.01, 0.002, 0.001, 0.0001) # fur depth, dorsal (m)
INSDEPVs <- c(0.0001, 0.002, 0.001, 0.0001) # fur depth, ventral (m)
for(i in 1:length(MASSs)){
  SHAPE_Bs <- c(1.6, 1.3, 6, 5)#c(1.6, 1.9, 11, 7.0)
  shapes <- GET_SHAPES(MASSs = MASSs[i] * MASSFRACs,
                       AREA = AREAs[i],
                       SHAPE_Bs = SHAPE_Bs,
                       SHAPE_Bs.min = c(1.6, 1.2, 6, 5),
                       SHAPE_Bs.max = c(1.6, 1.7, 11, 7.0))
  SHAPE_Bs <- shapes$SHAPE_Bs
  PJOINs <- shapes$PJOINs
  HomoTherm.out <- HomoTherm_var(MASS = MASSs[i],
                                 QMETAB_REST = QMETAB_RESTs[i],
                                 INSDEPDs = INSDEPDs,
                                 INSDEPVs = INSDEPVs,
                                 SHAPE_Bs = SHAPE_Bs,
                                 PJOINs = PJOINs,
                                 TAs = TAs1,
                                 TSKYs = TRADs1,
                                 TGRDs = TRADs1,
                                 RHs = RHs1,
                                 VELs = VELs1,
                                 CONV_ENHANCE = 1)
  balance <- cbind(TAs1, HomoTherm.out$balance)
  if(i == 1){
    HomoTherm.light <- balance
    par(oma = c(2, 2, 1, 2) + 0.1) # margin spacing stuff
    par(mar = c(4, 4, 1, 1) + 0.1) # margin spacing stuff
    par(mgp = c(3, 1, 0) ) # margin spacing stuff
    plot(Erickson, pch = 16, col = 2, ylab = 'W', xlab = 'T_air, deg C',
         xlim = c(-10, 37), ylim = c(0, 600), xaxs = 'i', yaxs = 'i')
    axis(1, at = 37)
    Erickson_clothed <-
      read.csv(paste0(localpath, 'data/Erickson/Erickson1956 Fig.1_clothed.txt'),
               skip = 1, head = F)
    points(Erickson_clothed, pch = 16, col = 'orange', ylab = 'W', xlab = 'T_air, deg C',
           xlim = c(-10, 37),  ylim = c(0, 600))
  }else{
    HomoTherm.light <- rbind(HomoTherm.light, balance)
  }
  points(TAs1, balance$QMETAB, type = 'l', col = 'grey')
}
slope.light <- lm(HomoTherm.light$QMETAB[HomoTherm.light$TAs1 < 25] ~
                    HomoTherm.light$TAs1[HomoTherm.light$TAs1 < 25])
abline(slope.light, lty = 2)

# clothed (Wool underwear, shirt, knickers, heavy sweater, wool socks
# and heavy shoes, trench coat and wool cap, mittens, scarf).
INSDEPDs <- c(0.02, 0.02, 0.02, 0.02) # fur depth, dorsal (m)
```

```
INSDEPVs <- c(0.02, 0.02, 0.02, 0.02) # fur depth, ventral (m)
for(i in 1:length(MASSs)){
  SHAPE_Bs <- c(1.6, 1.3, 6, 5)#c(1.6, 1.9, 11, 7.0)
  shapes <- GET_SHAPES(MASSs = MASSs[i] * MASSFRACs,
                       AREA = AREAs[i],
                       SHAPE_Bs = SHAPE_Bs,
                       SHAPE_Bs.min = c(1.6, 1.3, 6, 5),
                       SHAPE_Bs.max = c(1.6, 1.3, 6, 5))
  SHAPE_Bs <- shapes$SHAPE_Bs
  PJOINs <- shapes$PJOINs
  HomoTherm.out <- HomoTherm_var(MASS = MASSs[i],
                                 QMETAB_REST = QMETAB_RESTs[i],
                                 INSDEPDs = INSDEPDs,
                                 INSDEPVs = INSDEPVs,
                                 SHAPE_Bs = SHAPE_Bs,
                                 PJOINs = PJOINs,
                                 TAs = TAs2,
                                 TSKYs = TRADs2,
                                 TGRDs = TRADs2,
                                 RHs = RHs2,
                                 VELs = VELs2,
                                 CONV_ENHANCE = 1)
  balance <- cbind(TAs2, HomoTherm.out$balance)
  if(i == 1){
    HomoTherm.heavy <- balance
  }else{
    HomoTherm.heavy <- rbind(HomoTherm.heavy, balance)
  }
  points(TAs2, balance$QMETAB, type = 'l', col = 'grey')
}
slope.heavy <- lm(HomoTherm.heavy$QMETAB[HomoTherm.heavy$TAs2 < 15]
                  ~ HomoTherm.heavy$TAs2[HomoTherm.heavy$TAs2 < 15])
abline(slope.heavy, lty = 2)
```

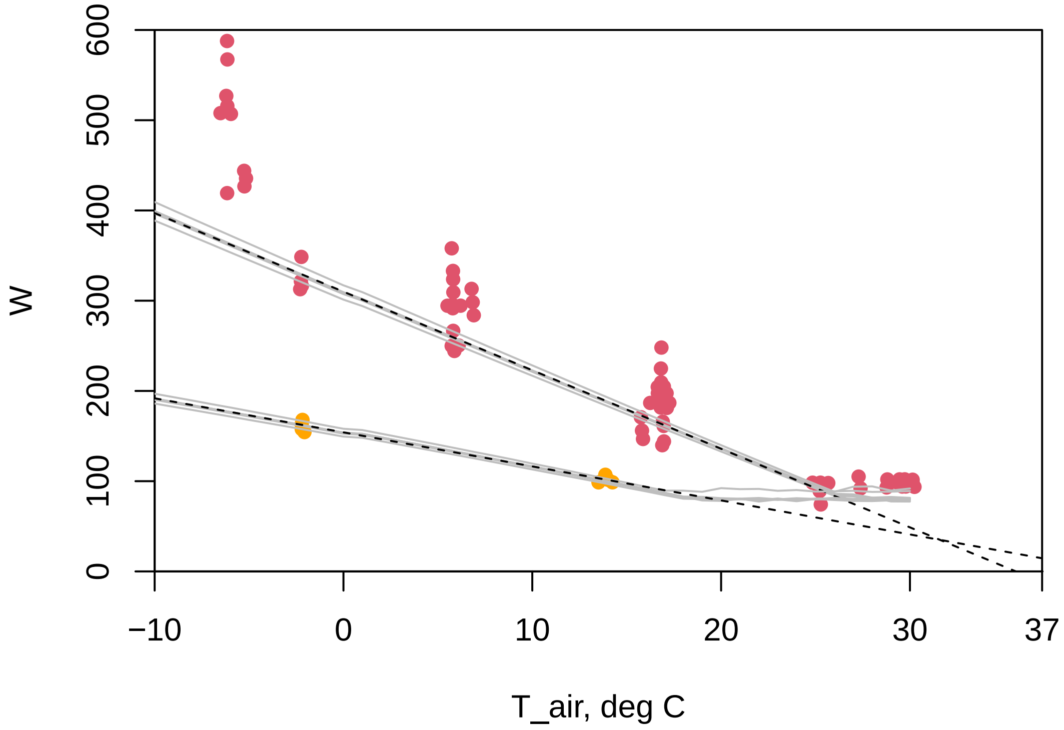

References

Erikson, H., J. Eirog, K. L. Andersen, and P. F. Scholander. 1956. The Critical Temperature in Naked Man. Acta Physiologica Scandinavica 37:35–39.
