# Supplementary material for: HomoTherm: An Open‐Source Approach to Modelling Heat Exchange in Humans and Other Hominins in Diverse Environments
Source: Glob Chang Biol. 2026 Apr 1;32(4):e70830. doi: 10.1111/gcb.70830 (PMC13044332; doi:10.1111/gcb.70830)
Supplement: Supplementary file 3 — Appendix S3: gcb70830‐sup‐0003‐Appendix 3.pdf. [file GCB-32-e70830-s003.pdf]

Test of HomoTherm model against Winslow et al. 1937

Michael Kearney

2026-01-09

Overview

A test of the HomoTherm model against the data reported in Winslow, Herrington and Gagge (1937). This study applied a new method of partitional calorimetry developed by the authors that allowed differentiation of different pathways of heat exchange over short time periods, i.e. without having to wait for thermal steady state.

The chamber had nine interior copper surfaces with which the radiation environment could be controlled independently of air temperature. The subjects sat on a cloth-covered metal chair in a reclined position. Skin temperature was the weighted average of 15 points.

The chair was on a balance and weight loss was used to determine evaporative water loss. Metabolism was determined by oxygen consumption, radiation by the Stefan-Boltzmann equation given skin and mean wall temperature, convection also by an equation incorporating skin and air temperature and an empirically determined convection coefficient. A non-zero sum of these fluxes reflects storage which can be reflected in changed body temperature, but not necessarily rectal temperature because under strong cooling conditions the peripheral areas cool off more slowly than the core.

In these simulations the HomoTherm model is only given the resting metabolic rate and insluation depth (nude), all other parameters are default.

Load the libraries and other functions

Note a different version of the 'plot_human' function of NicheMapR is used here, that only plots the coronal section.

```
library(NicheMapR)
library(readxl)
localpath <- 'c:/Users/mrke/Dropbox/Current Research Projects/mammal_projects/manmo analysis/'
#source(paste0(localpath, 'code/HomoTherm/plot_human2.R'))
```

Load the Winslow et al. observations

The data are for around 250 experiments with each subject, averaged by groups according to the atmospheric conditions.

```
obs <- read.csv(paste0(localpath, 'data/Winslow1937/Tables1to3.csv'))
obs$T_B <- obs$T_S + obs$deltaTb
obs1 <- subset(obs, Subject == 1)
obs2 <- subset(obs, Subject == 2)
```

```r
Tskins_I_head <- read.csv(paste0(localpath,
  'data/Winslow1937/Winslow1937_Fig2a_head.txt'), head = FALSE, skip = 1)
Tskins_I_trunk <- read.csv(paste0(localpath,
  'data/Winslow1937/Winslow1937_Fig2a_trunk.txt'), head = FALSE, skip = 1)
Tskins_I_upper_ex <- read.csv(paste0(localpath,
  'data/Winslow1937/Winslow1937_Fig2a_upper_extremities.txt'), head = FALSE,
  skip = 1)
Tskins_I_lower_ex <- read.csv(paste0(localpath,
  'data/Winslow1937/Winslow1937_Fig2a_lower_extremities.txt'), head = FALSE,
  skip = 1)

Tskins_II_head <- read.csv(paste0(localpath,
  'data/Winslow1937/Winslow1937_Fig2b_head.txt'), head = FALSE, skip = 1)
Tskins_II_trunk <- read.csv(paste0(localpath,
  'data/Winslow1937/Winslow1937_Fig2b_trunk.txt'), head = FALSE, skip = 1)
Tskins_II_upper_ex <- read.csv(paste0(localpath,
  'data/Winslow1937/Winslow1937_Fig2b_upper_extremities.txt'), head = FALSE,
  skip = 1)
Tskins_II_lower_ex <- read.csv(paste0(localpath,
  'data/Winslow1937/Winslow1937_Fig2b_lower_extremities.txt'), head = FALSE,
  skip = 1)
```

Now assign the environmental conditions for each subject.

```r
# environmental variables
TAs1 <- obs1$T_A # air temperatures, deg C
TRADs1 <- obs1$T_W # radiant temperatures, deg C
TBs1 <- obs1$T_B # body temperatures, deg C
QGENs1 <- obs1$M * 4184 / 3600 # metabolic rate, W
TOs1 <- obs1$T_O # operative temperatures, deg C
RHs1 <- obs1$RH # relative humidities, %
VELs1 <- rep(20 * 0.3048 / 60, length(TAs1)) # wind speeds, from ft/min to m/s

TAs2 <- obs2$T_A # air temperatures, deg C
TRADs2 <- obs2$T_W # radiant temperatures, deg C
TBs2 <- obs2$T_B # body temperatures, deg C
QGENs2 <- obs2$M * 4184 / 3600 # metabolic rate, W
TOs2 <- obs2$T_O # operative temperatures, deg C
RHs2 <- obs2$RH # relative humidities, %
VELs2 <- rep(20 * 0.3048 / 60, length(TAs2)) # wind speeds, from ft/min to m/s
```

Plot People

Two subjects were used, one 'pycnic' (stocky) and the other 'leptosomic' (slender).

```r
# person parameters
MASS <- 104.326 # MASS, kg
HEIGHT <- 170.18 # height, cm
AREA <- 0.00718 * MASS ^ 0.425 * HEIGHT ^ 0.725 # DuBois area, m2
INSDEPDs <- c(0.01, 0, 0, 0) # fur depth, dorsal (m)
INSDEPVs <- c(0, 0, 0, 0) # fur depth, ventral (m)
MASSFRACs <- c(0.0761, 0.501, 0.049, 0.162)
```

```r
SHAPE_Bs <- c(1.6, 1.73, 10, 5)#c(1.6, 1.9, 11, 7.0)
shapes <- GET_SHAPES(MASSs = MASS * MASSFRACs,
                     AREA = AREA,
                     SHAPE_Bs = SHAPE_Bs,
                     SHAPE_Bs.min = c(1.6, 1.2, 6, 5),
                     SHAPE_Bs.max = c(1.6, 1.73, 10, 7.0))
SHAPE_Bs <- shapes$SHAPE_Bs
PJOINs <- shapes$PJOINs
HEIGHT_out <- shapes$HEIGHT_out
AREA_out <- shapes$AREA_out
#rbind(AREA, AREA_out, HEIGHT/100, HEIGHT_out)

par(mfrow = c(1, 1))
plot_human(MASS = MASS,
           HEIGHT = HEIGHT,
           INSDEPDs = INSDEPDs,
           INSDEPVs = INSDEPVs,
           SHAPE_Bs = SHAPE_Bs)
```

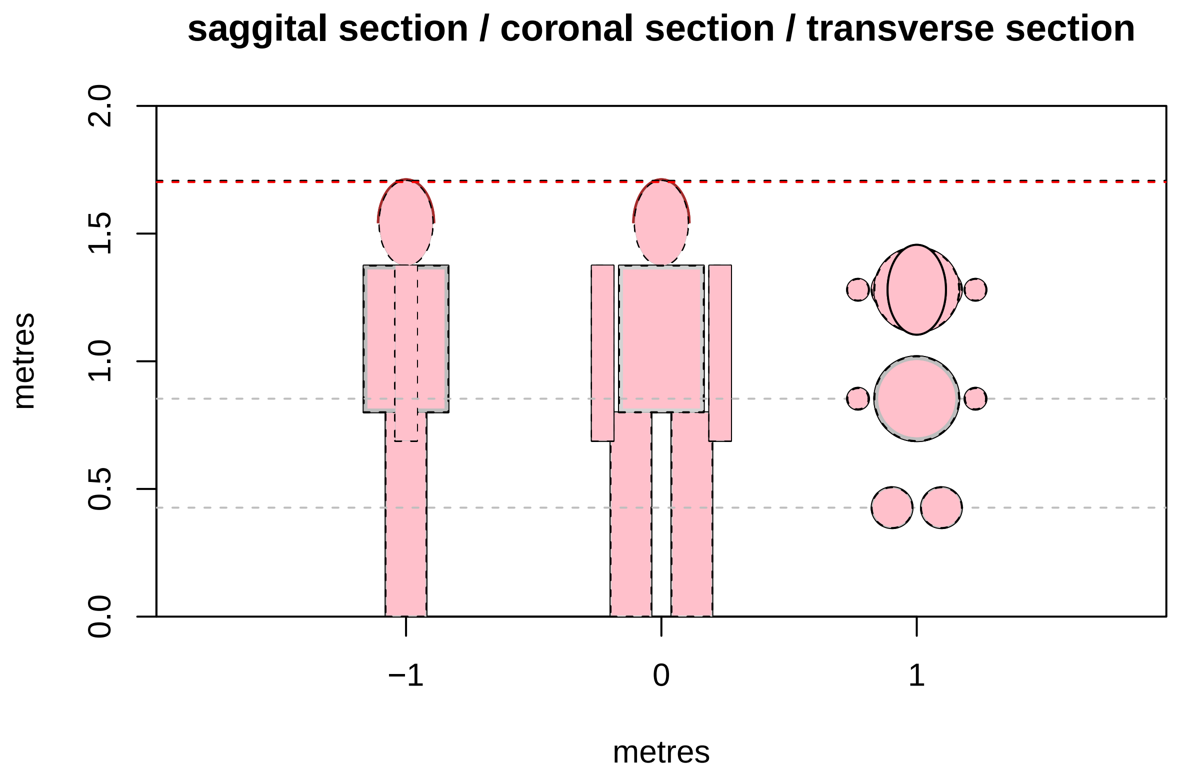


```
## [1] 1.706764
```

```r
# person parameters
MASS <- 47.6 # mass, kg
HEIGHT <- 165 # height, cm
AREA <- 0.00718 * MASS ^ 0.425 * HEIGHT ^ 0.725 # DuBois area, m2
```

```r
SHAPE_Bs <- c(1.4, 1.9, 22, 8)#c(1.6, 1.9, 11, 7.0)
shapes <- GET_SHAPES(MASSs = MASS * MASSFRACs,
                     AREA = AREA,
                     SHAPE_Bs = SHAPE_Bs,
                     SHAPE_Bs.min = c(1.6, 1.2, 6, 5),
                     SHAPE_Bs.max = c(1.4, 2.5, 22, 8))
SHAPE_Bs <- shapes$SHAPE_Bs
PJOINs <- shapes$PJOINs
HEIGHT_out <- shapes$HEIGHT_out
AREA_out <- shapes$AREA_out
#rbind(AREA, AREA_out, HEIGHT/100, HEIGHT_out)

plot_human(MASS = MASS,
           HEIGHT = HEIGHT,
           INSDEPDs = INSDEPDs,
           INSDEPVs = INSDEPVs,
           SHAPE_Bs = SHAPE_Bs)
```

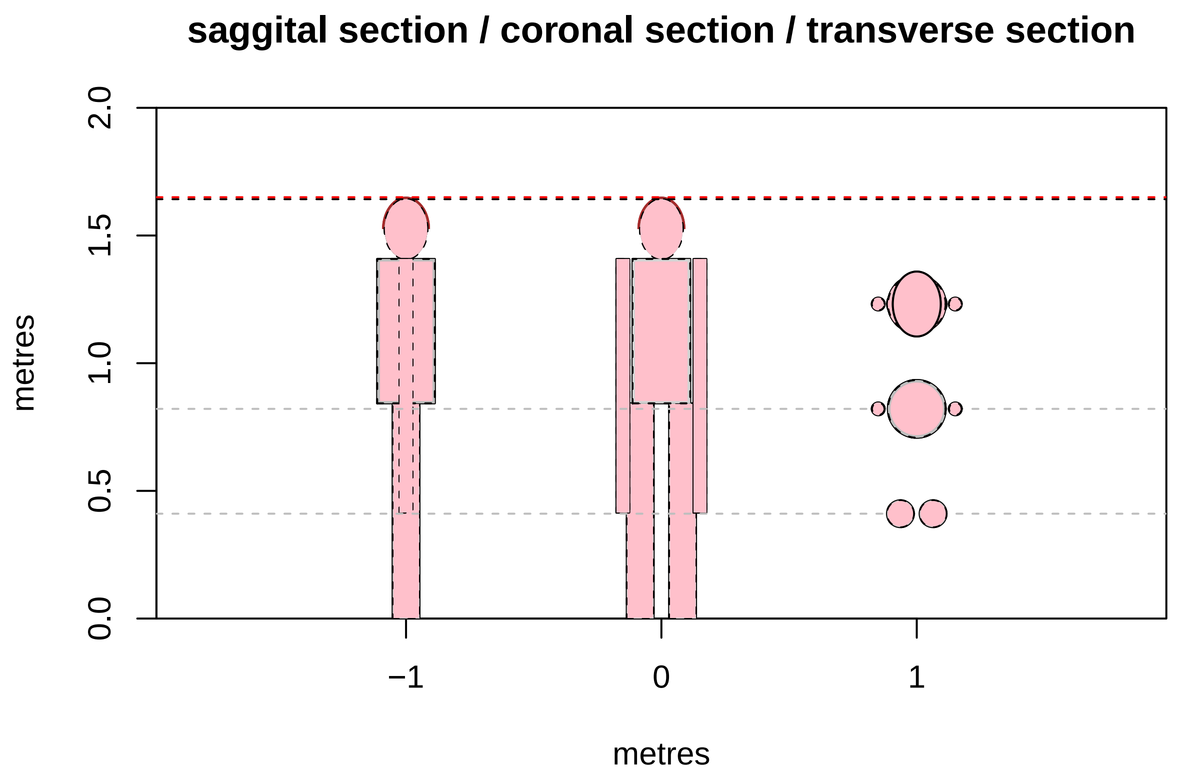


```
## [1] 1.642289
```

Subject I

Set person parameters to the ‘pycnic’ (stout) case and run simulations for the HomoTherm model, then the MANMO model, then the HHB model.

```r
# person parameters
MASS <- 104.326 # MASS, kg
HEIGHT <- 170.18 # height, cm
AREA <- 0.00718 * MASS ^ 0.425 * HEIGHT ^ 0.725 # DuBois area, m2
QMETAB_REST <- quantile(obs1$M, 0.1) * 4184 / 3600 # basal metabolic rate, W
PCTBAREVAPs <- c(60, 99, 99, 99)
SHAPE_Bs <- c(1.6, 1.73, 10, 5)#c(1.6, 1.9, 11, 7.0)
shapes <- GET_SHAPES(MASSs = MASS * MASSFRACs,
                     AREA = AREA,
                     SHAPE_Bs = SHAPE_Bs,
                     SHAPE_Bs.min = c(1.6, 1.2, 6, 5),
                     SHAPE_Bs.max = c(1.6, 1.73, 10, 7.0))
SHAPE_Bs <- shapes$SHAPE_Bs
PJOINs <- shapes$PJOINs
HEIGHT_out <- shapes$HEIGHT_out
AREA_out <- shapes$AREA_out
rbind(AREA, AREA_out, HEIGHT/100, HEIGHT_out)
```

```
##                 [,1]
## AREA       2.144618
## AREA_out   2.193644
##            1.701800
## HEIGHT_out 1.706274
```

```r
# run HomoTherm simulation
for(i in 1:length(TAs1)){
  HomoTherm.out <- HomoTherm_var(MASS = MASS,
                                 QMETAB_REST = QMETAB_REST,
                                 INSDEPDs = INSDEPDs,
                                 INSDEPVs = INSDEPVs,
                                 SHAPE_Bs = SHAPE_Bs,
                                 PJOINs = PJOINs,
                                 PCTBAREVAPs = PCTBAREVAPs,
                                 TAs = TAs1[i],
                                 TSKYs = TRADs1[i],
                                 TGRDs = TRADs1[i],
                                 RHs = RHs1[i],
                                 VELs = VELs1[i],
                                 CONV_ENHANCE = 1.4)
  balance <- HomoTherm.out$balance
  head.treg <- HomoTherm.out$head.treg
  trunk.treg <- HomoTherm.out$trunk.treg
  arm.treg <- HomoTherm.out$arm.treg
  leg.treg <- HomoTherm.out$leg.treg

  if(i == 1){
    HomoTherm.I <- balance
    HomoTherm.I.head <- head.treg
    HomoTherm.I.trunk <- trunk.treg
    HomoTherm.I.arm <- arm.treg
    HomoTherm.I.leg <- leg.treg
  }else{
    HomoTherm.I <- rbind(HomoTherm.I, balance)
```

```
    HomoTherm.I.head <- rbind(HomoTherm.I.head, head.treg)
    HomoTherm.I.trunk <- rbind(HomoTherm.I.trunk, trunk.treg)
    HomoTherm.I.arm <- rbind(HomoTherm.I.arm, arm.treg)
    HomoTherm.I.leg <- rbind(HomoTherm.I.leg, leg.treg)
  }
}

all.I <- cbind(obs1, HomoTherm.I)

HomoTherm.I <- cbind(TOs1, HomoTherm.I)
colnames(HomoTherm.I)[1] <- 'TOs'
HomoTherm.I <- HomoTherm.I[order(HomoTherm.I$TOs), ]
```

Plot observed and predicted skin temperatures for different parts of the body. Winslow et al. (1937) measured the head, trunk and the upper and lower extremities, whereas the simulation outputs head, trunk, arms and legs. So the upper and lower extremity values were both plotted in the figures below.

```
par(mfrow = c(2, 2))
par(oma = c(4, 1, 1, 1) + 0.1) # margin spacing
par(mar = c(3, 3, 1, 1) + 0.1) # margin spacing
par(mgp = c(2, 1, 0) ) # margin spacing

with(obs1,
     plot(T_O, T_S, ylim = c(27, 38), xlim = c(43, 16), ylab =
            expression("skin temperature, "*degree*C), xlab =
            expression("operative temperature, "*degree*C),
          col = 'pink', pch = 16, cex = 1.25, main = 'head'))
points(Tskins_I_head$V1, Tskins_I_head$V2, col = 'red', pch = 16, cex = 1.25)
points(rev(HomoTherm.I$TOs), HomoTherm.I.head$TSKIN_V, col = "black", pch = 16)

with(obs1,
     plot(T_O, T_S, ylim = c(27, 38), xlim = c(43, 16), ylab =
            expression("skin temperature, "*degree*C), xlab =
            expression("operative temperature, "*degree*C),
          col = 'pink', pch = 16, cex = 1.25, main = 'trunk'))
points(Tskins_I_trunk$V1, Tskins_I_trunk$V2, col = 'red', pch = 16, cex = 1.25)
points(rev(HomoTherm.I$TOs), HomoTherm.I.trunk$TSKIN_V, col = "black", pch = 16)

with(obs1,
     plot(T_O, T_S, ylim = c(27, 38), xlim = c(43, 16), ylab =
            expression("skin temperature, "*degree*C), xlab =
            expression("operative temperature, "*degree*C),
          col = 'pink', pch = 16, cex = 1.25, main = 'arm'))
points(Tskins_I_lower_ex$V1, Tskins_I_lower_ex$V2, col = 'red', pch = 16,
       cex = 1.25)
points(Tskins_I_lower_ex$V1, Tskins_I_lower_ex$V2, col = 'red', pch = 16,
       cex = 1.25)
points(Tskins_I_upper_ex$V1, Tskins_I_upper_ex$V2, col = 'red', pch = 16,
       cex = 1.25)
points(rev(HomoTherm.I$TOs), HomoTherm.I.arm$TSKIN_V, col = "black", pch = 16)

with(obs1,
     plot(T_O, T_S, ylim = c(27, 38), xlim = c(43, 16), ylab =
```

```
            expression("skin temperature, "*degree*C), xlab =
            expression("operative temperature, "*degree*C),
          col = 'pink', pch = 16, cex = 1.25, main = 'leg'))
points(Tskins_I_lower_ex$V1, Tskins_I_lower_ex$V2, col = 'red', pch = 16,
       cex = 1.25)
points(Tskins_I_lower_ex$V1, Tskins_I_lower_ex$V2, col = 'red', pch = 16,
       cex = 1.25)
points(Tskins_I_upper_ex$V1, Tskins_I_upper_ex$V2, col = 'red', pch = 16,
       cex = 1.25)
points(rev(HomoTherm.I$TOs), HomoTherm.I.leg$TSKIN_V, col = "black", pch = 16)
```

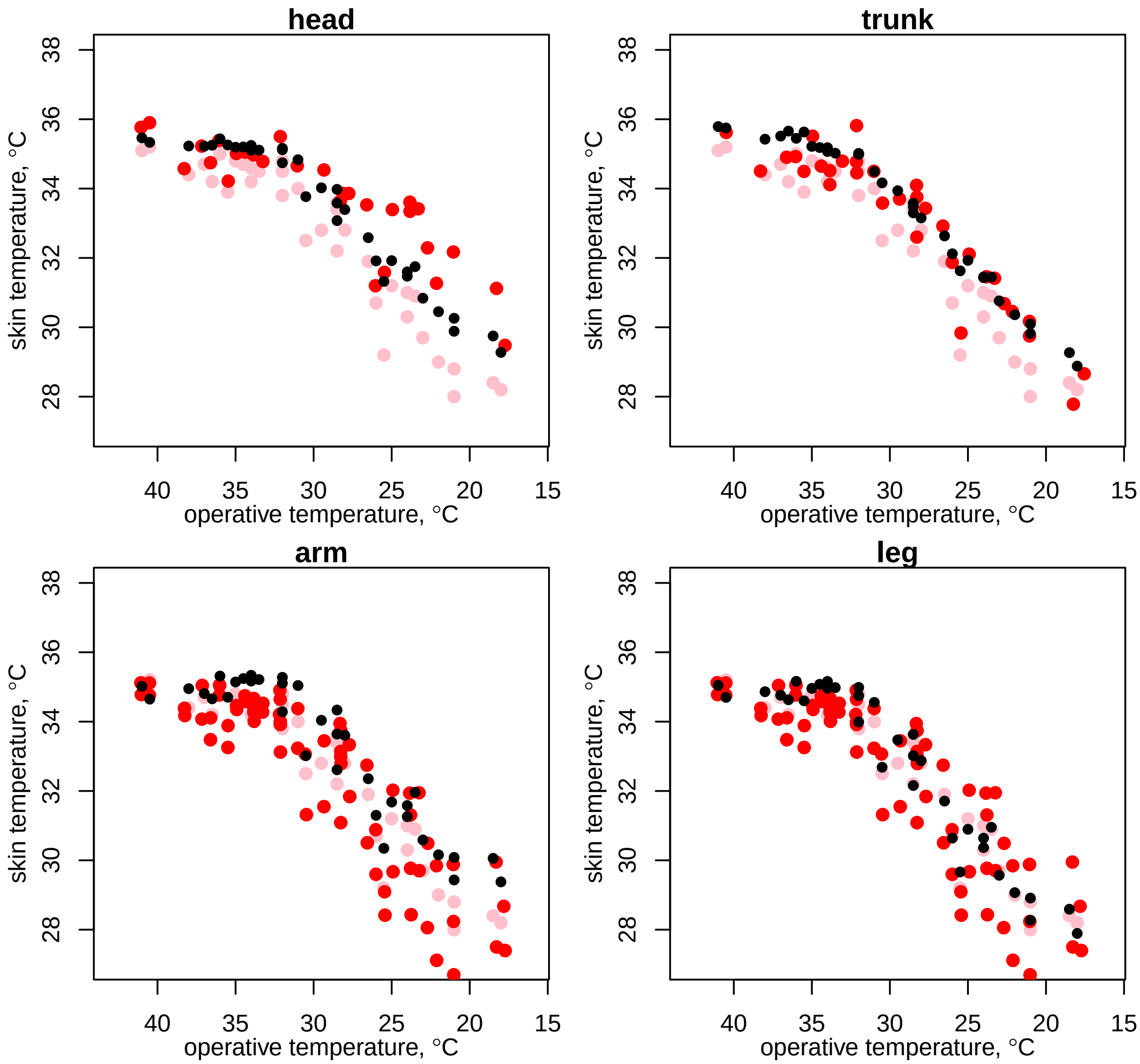

Plot the other predictions and observations against operative temperature. Not that here the convective and radiative exchange is being summed to compare with the HHB model, which does not report them separately.

```r
par(mfrow = c(2, 2))
par(oma = c(4, 1, 1, 1) + 0.1) # margin spacing
par(mar = c(3, 3, 1, 1) + 0.1) # margin spacing
par(mgp = c(2, 1, 0) ) # margin spacing

with(obs1,
     plot(T_O, T_S, ylim = c(23, 38), xlim = c(15, 45), ylab =
            expression("temperature, "*degree*C), xlab =
            expression("operative temperature, "*degree*C), col = 'red',
          pch = 16, cex = 1.25, main = 'temperature'))
with(obs1, points(T_O, T_B, col = 'red', pch = 4, cex = 0.75))
points(HomoTherm.I$TOs, HomoTherm.I$T_SKIN, col = "black", pch = 16)
points(HomoTherm.I$TO, HomoTherm.I$T_CORE, col = "black", pch = 4, cex = 0.75)
legend(33, 32, cex = 0.8, legend = c('Observed', 'HomoTherm'),
       col = c('red', 'black'), pch = 16, bty = 'n', ncol = 1)
legend(16, 35, cex = 0.8, legend = c('core', 'skin'),
       col = c('black', 'black'), pch = c(4, 16), bty = 'n', ncol = 1)

with(obs1, plot(T_O, M * 4184 / 3600, ylim = c(70, 270), xlim = c(15, 45),
                ylab = "QMETAB, W", xlab =
                  expression("operative temperature, "*degree*C),
                col = 'red', pch = 16, cex = 1.25, main = 'metabolic rate'))
with(obs1, points(T_O, (S + M) * 4184 / 3600, col = 'red', pch = 1, cex = 1.25))
points(HomoTherm.I$TOs, HomoTherm.I$QMETAB, col = "black", pch = 16)
legend(20, 275, cex = 0.8, legend = c('plus storage'),
       col = 'red', pch = 1, ncol = 1)

with(obs1, plot(T_O, (C + R) * 4184 / 3600, ylim = c(-300, 200), xlim =
                  c(15, 45), ylab = "QDRY, W", xlab =
                  expression("operative temperature, "*degree*C),
                col = 'red', pch = 16, cex = 1.25, main = 'dry heat'))
points(HomoTherm.I$TOs, HomoTherm.I$QRAD_IN - HomoTherm.I$QRAD_OUT +
         HomoTherm.I$QCONV + HomoTherm.I$QCONV_RESP, col = "black", pch = 16)
with(obs1, plot(T_O, -E * 4184 / 3600, ylim = c(-100, 250), xlim = c(15, 45),
                ylab = "QEVAP, W", xlab =
                  expression("operative temperature, "*degree*C), col = 'red',
                pch = 16, cex = 1.25, main = 'evaporation'))
points(HomoTherm.I$TOs, -(HomoTherm.I$QEVAP_RESP + HomoTherm.I$QEVAP_CUT),
       col = "black", pch = 16)
```

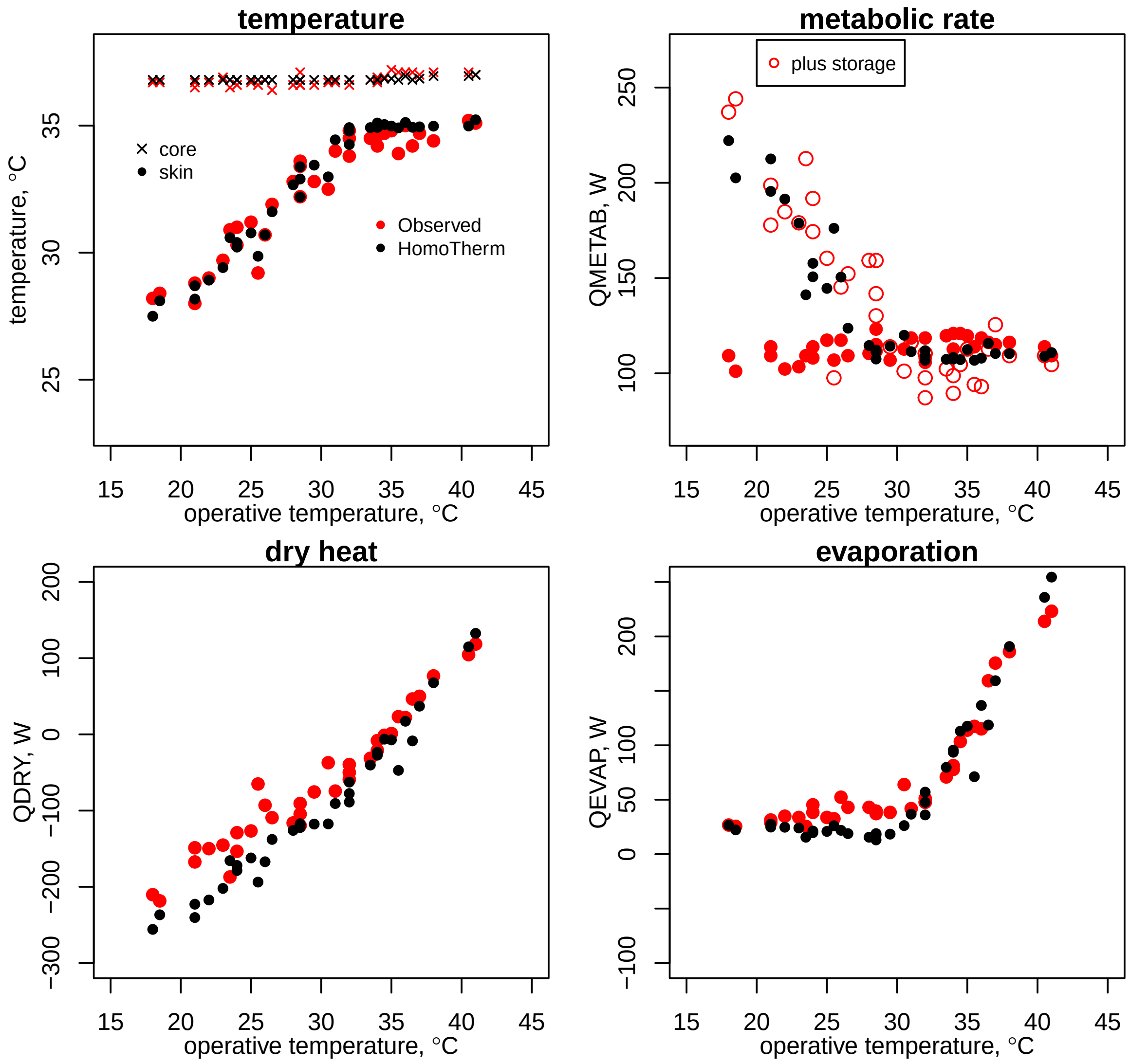

Now plot the correlations of predictions and observations.

```
par(mfrow = c(2, 2))
par(oma = c(4, 1, 1, 1) + 0.1) # margin spacing
par(mar = c(3, 3, 1, 1) + 0.1) # margin spacing
par(mgp = c(2, 1, 0) ) # margin spacing

with(all.I,
     plot(T_S, T_SKIN, ylim = c(23, 38), xlim = c(23, 38), ylab =
            expression("pred, "*degree*C),
          xlab = expression("obs, "*degree*C), col = 'black', pch = 16,
          cex = 1.25, main = 'temperature'))
with(all.I, points(T_B, T_CORE, col = "black", pch = 4, cex = 1.25))
abline(0, 1)
```

```r
with(all.I,
     plot(M * 4184 / 3600, QMETAB, ylim = c(70, 270), xlim = c(70, 270), ylab =
            "pred, W", xlab = "obs, W", col = 'black', pch = 16, cex = 1.25,
          main = 'metabolic rate'))
with(all.I, points((S + M) * 4184 / 3600, QMETAB, col = "black", pch = 1,
                   cex = 1.25))
abline(0, 1)

with(all.I,
     plot((C + R) * 4184 / 3600, QCONV_RESP + QCONV + QRAD_IN - QRAD_OUT,
          ylim = c(-300, 250), xlim =  c(-300, 250), ylab = "pred, W",
          xlab = "obs, W", col = 'black',
          pch = 16, cex = 1.25, main = 'dry heat'))
abline(0, 1)

with(all.I,
     plot(-E * 4184 / 3600, -(QEVAP_RESP + QEVAP_CUT), ylim = c(-100, 250),
          xlim = c(-100, 250),
          ylab = "pred, W", xlab = "obs, W", col = 'black', pch = 16,
          cex = 1.25, main = 'evaporation'))
abline(0, 1)
```

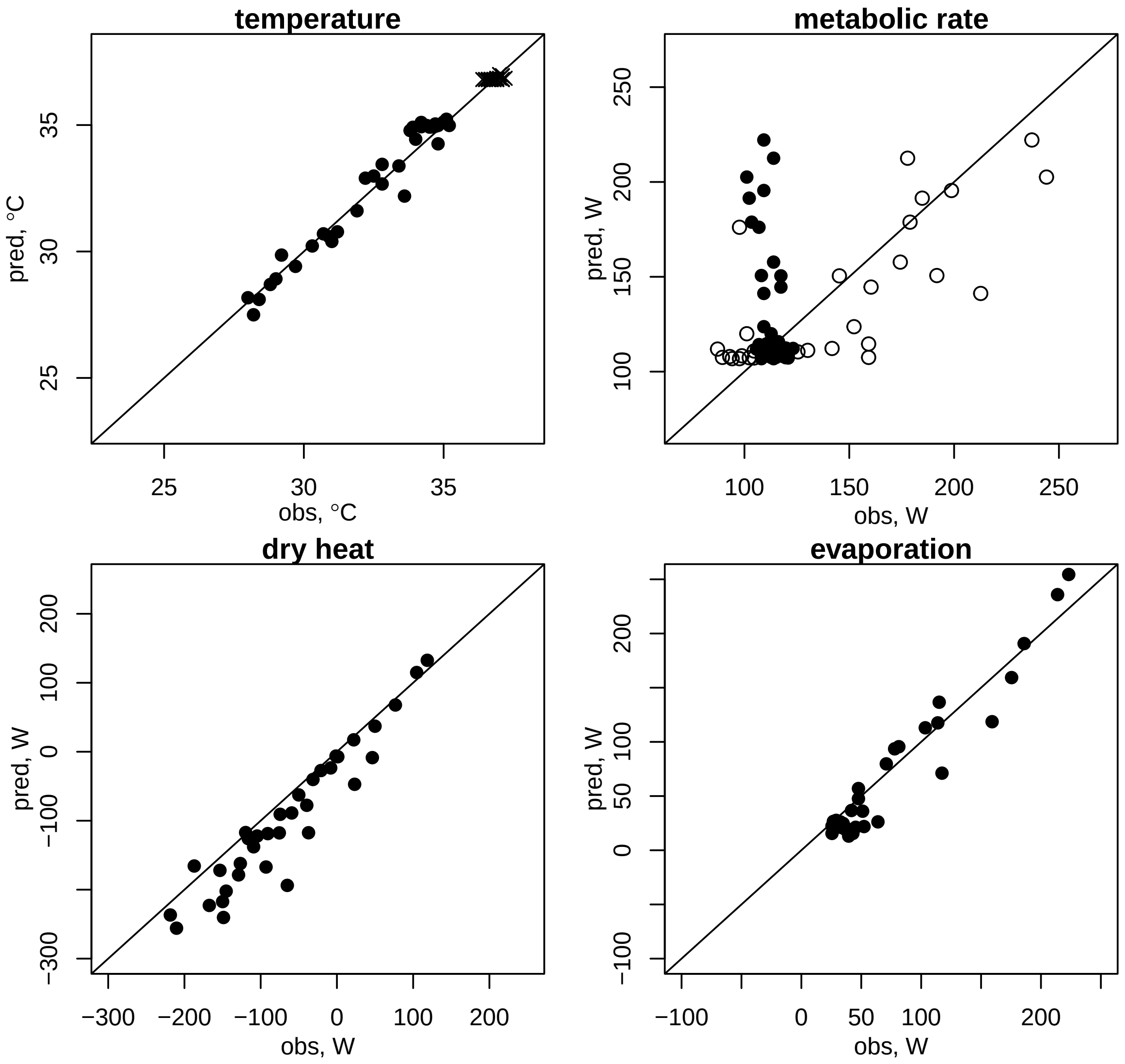

Subject II

Set subject II parameters - the leptosomic person. Note that in addition to changing the mass, height and associated resting metabolic rate, the minimum core body temperature has been dropped to reflect the observed situation and the fat/flesh conductivity starting values altered to better fit the different skin temperatures observed.

```
# person parameters
MASS <- 47.6 # mass, kg
HEIGHT <- 165 # height, cm
AREA <- 0.00718 * MASS ^ 0.425 * HEIGHT ^ 0.725 # DuBois area, m2
QMETAB_REST <- quantile(obs2$M, 0.1) * 4184 / 3600 # basal metabolic rate, W
```

```r
SHAPE_Bs <- c(1.4, 1.9, 22, 8)#c(1.6, 1.9, 11, 7.0)
shapes <- GET_SHAPES(MASSs = MASS * MASSFRACs,
                     AREA = AREA,
                     SHAPE_Bs = SHAPE_Bs,
                     SHAPE_Bs.min = c(1.6, 1.2, 6, 5),
                     SHAPE_Bs.max = c(1.4, 2.5, 22, 8))
SHAPE_Bs <- shapes$SHAPE_Bs
PJOINs <- shapes$PJOINs
HEIGHT_out <- shapes$HEIGHT_out
AREA_out <- shapes$AREA_out
rbind(AREA, AREA_out, HEIGHT/100, HEIGHT_out)
```

```
##                [,1]
## AREA       1.502392
## AREA_out   1.492342
##            1.650000
## HEIGHT_out 1.642035
```

```r
plot_human(MASS = MASS,
           HEIGHT = HEIGHT,
           INSDEPDs = INSDEPDs,
           INSDEPVs = INSDEPVs,
           SHAPE_Bs = SHAPE_Bs)
```

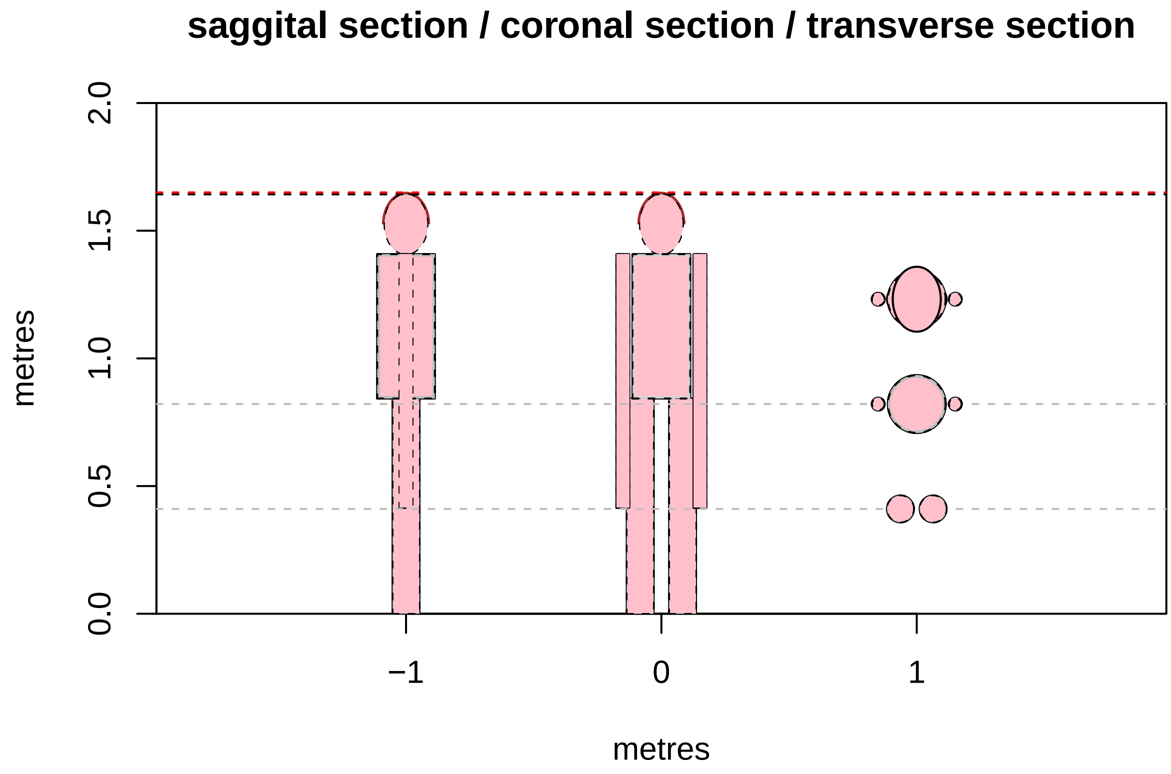


```
## [1] 1.642289
```

Run simulations.

```
# run HomoTherm simulation
for(i in 1:length(TAs2)){
  HomoTherm.out <- HomoTherm_var(MASS = MASS,
                                 QMETAB_REST = QMETAB_REST,
                                 INSDEPDs = INSDEPDs,
                                 INSDEPVs = INSDEPVs,
                                 SHAPE_Bs = SHAPE_Bs,
                                 PJOINs = PJOINs,
                                 PCTBAREVAPs = PCTBAREVAPs,
                                 TAs = TAs2[i],
                                 TSKYs = TRADs2[i],
                                 TGRDs = TRADs2[i],
                                 RHs = RHs2[i],
                                 VELs = VELs2[i],
                                 CONV_ENHANCE = 1.4)
  balance <- HomoTherm.out$balance
  head.treg <- HomoTherm.out$head.treg
  trunk.treg <- HomoTherm.out$trunk.treg
  arm.treg <- HomoTherm.out$arm.treg
  leg.treg <- HomoTherm.out$leg.treg

  if(i == 1){
    HomoTherm.II <- balance
    HomoTherm.II.head <- head.treg
    HomoTherm.II.trunk <- trunk.treg
    HomoTherm.II.arm <- arm.treg
    HomoTherm.II.leg <- leg.treg
  }else{
    HomoTherm.II <- rbind(HomoTherm.II, balance)
    HomoTherm.II.head <- rbind(HomoTherm.II.head, head.treg)
    HomoTherm.II.trunk <- rbind(HomoTherm.II.trunk, trunk.treg)
    HomoTherm.II.arm <- rbind(HomoTherm.II.arm, arm.treg)
    HomoTherm.II.leg <- rbind(HomoTherm.II.leg, leg.treg)
  }
}
all.II <- cbind(obs2, HomoTherm.II)

HomoTherm.II <- cbind(TOs2, HomoTherm.II)
colnames(HomoTherm.II)[1] <- 'TOs'
HomoTherm.II <- HomoTherm.II[order(HomoTherm.II$TOs), ]
```

Plot skin temperatures.

```
par(mfrow = c(2, 2))
par(oma = c(4, 1, 1, 1) + 0.1) # margin spacing
par(mar = c(3, 3, 1, 1) + 0.1) # margin spacing
par(mgp = c(2, 1, 0) ) # margin spacing

with(obs2,
     plot(T_O, T_S, ylim = c(27, 38), xlim = c(43, 16), ylab =
            expression("skin temperature, "*degree*C), xlab =
            expression("operative temperature, "*degree*C),
```

```
          col = 'pink', pch = 16, cex = 1.25, main = 'head'))
points(Tskins_II_head$V1, Tskins_II_head$V2, col = 'red', pch = 16, cex = 1.25)
points(rev(HomoTherm.II$TOs), HomoTherm.II.head$TSKIN_V,
       col = "black", pch = 16)

with(obs2,
     plot(T_O, T_S, ylim = c(27, 38), xlim = c(43, 16), ylab =
            expression("skin temperature, "*degree*C), xlab =
            expression("operative temperature, "*degree*C),
          col = 'pink', pch = 16, cex = 1.25, main = 'trunk'))
points(Tskins_II_trunk$V1, Tskins_II_trunk$V2, col = 'red', pch = 16,
       cex = 1.25)
points(rev(HomoTherm.II$TOs), HomoTherm.II.trunk$TSKIN_V, col =
         "black", pch = 16)

with(obs2,
     plot(T_O, T_S, ylim = c(27, 38), xlim = c(43, 16), ylab =
            expression("skin temperature, "*degree*C), xlab =
            expression("operative temperature, "*degree*C),
          col = 'pink', pch = 16, cex = 1.25, main = 'arm'))
points(Tskins_II_lower_ex$V1, Tskins_II_lower_ex$V2, col = 'red', pch = 16,
       cex = 1.25)
points(Tskins_II_lower_ex$V1, Tskins_II_lower_ex$V2, col = 'red', pch = 16,
       cex = 1.25)
points(Tskins_II_upper_ex$V1, Tskins_II_upper_ex$V2, col = 'red', pch = 16,
       cex = 1.25)
points(rev(HomoTherm.II$TOs), HomoTherm.II.arm$TSKIN_V, col = "black", pch = 16)

with(obs2,
     plot(T_O, T_S, ylim = c(27, 38), xlim = c(43, 16), ylab =
            expression("skin temperature, "*degree*C), xlab =
            expression("operative temperature, "*degree*C),
          col = 'pink', pch = 16, cex = 1.25, main = 'leg'))
points(Tskins_II_lower_ex$V1, Tskins_II_lower_ex$V2, col = 'red', pch = 16,
       cex = 1.25)
points(Tskins_II_lower_ex$V1, Tskins_II_lower_ex$V2, col = 'red', pch = 16,
       cex = 1.25)
points(Tskins_II_upper_ex$V1, Tskins_II_upper_ex$V2, col = 'red', pch = 16,
       cex = 1.25)
points(rev(HomoTherm.II$TOs), HomoTherm.II.leg$TSKIN_V, col = "black", pch = 16)
```

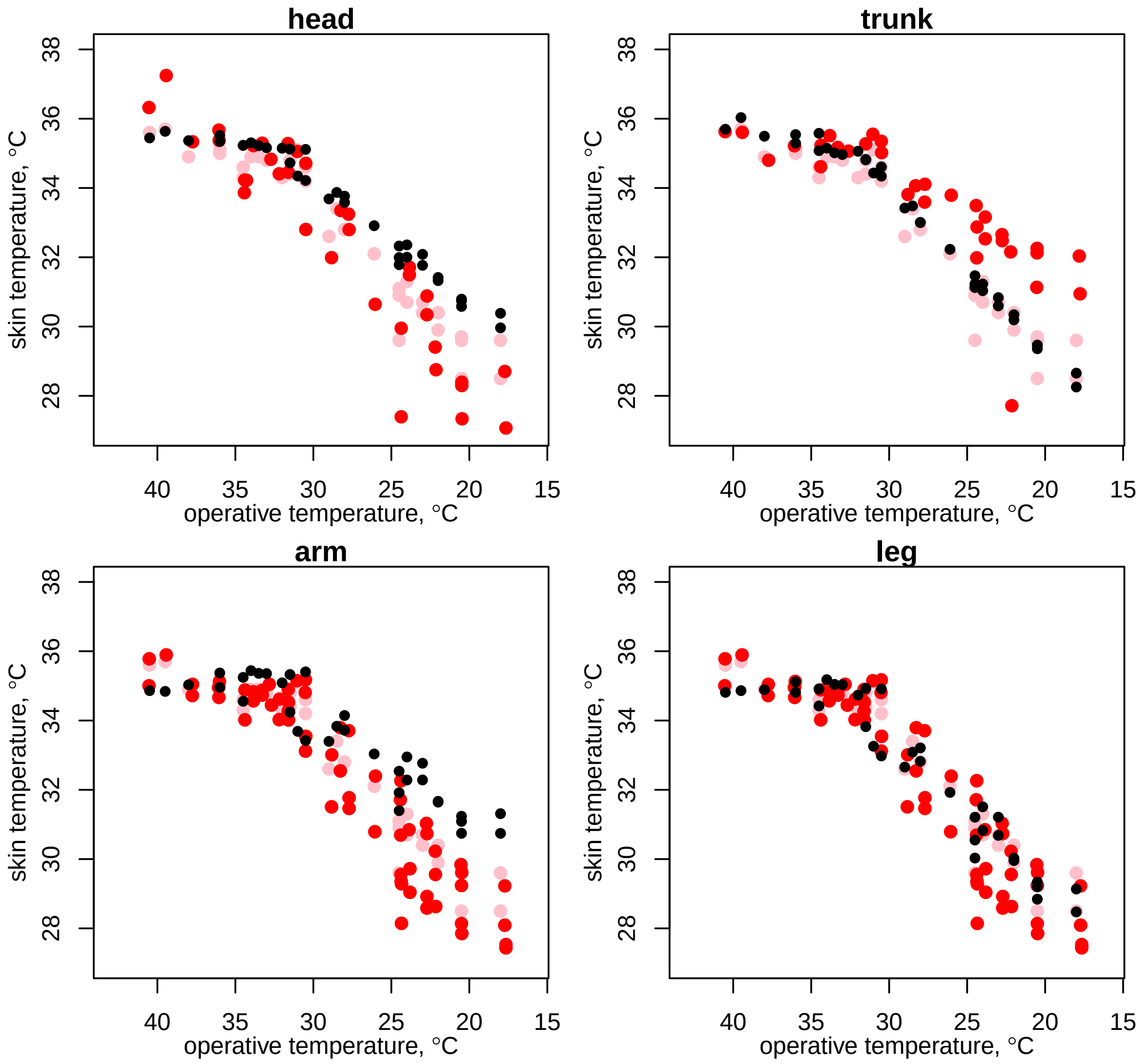

Plot the other predictions and observations against operative temperature.

```
par(mfrow = c(2, 2))
par(oma = c(4, 1, 1, 1) + 0.1) # margin spacing
par(mar = c(3, 3, 1, 1) + 0.1) # margin spacing
par(mgp = c(2, 1, 0) ) # margin spacing

with(obs2,
     plot(T_O, T_S, ylim = c(23, 38), xlim = c(15, 45),
          ylab = expression("temperature, "*degree*C), xlab = expression("operative temperature, "*degr
          pch = 16, cex = 1.25, main = 'temperature'))
with(obs2, points(T_O, T_B, col = 'red', pch = 4, cex = 0.75))
points(HomoTherm.II$TOs, HomoTherm.II$T_SKIN, col = "black", pch = 16)
points(HomoTherm.II$TO, HomoTherm.II$T_CORE, col = "black", pch = 4, cex = 0.75)
```

```
with(obs2, plot(T_O, M * 4184 / 3600, ylim = c(70, 270), xlim = c(15, 45),
                ylab = "QMETAB, W", xlab =
                  expression("operative temperature, "*degree*C), col = 'red',
                pch = 16, cex = 1.25, main = 'metabolic rate'))
with(obs2, points(T_O, (S + M) * 4184 / 3600, col = 'red', pch = 1, cex = 1.25))
points(HomoTherm.II$TOs, HomoTherm.II$QMETAB, col = "black", pch = 16)

with(obs2, plot(T_O, (C + R) * 4184 / 3600, ylim = c(-300, 200), xlim =
                  c(15, 45), ylab = "QDRY, W", xlab =
                  expression("operative temperature, "*degree*C),
                col = 'red', pch = 16, cex = 1.25, main = 'dry heat'))
points(HomoTherm.II$TOs, HomoTherm.II$QRAD_IN - HomoTherm.II$QRAD_OUT +
         HomoTherm.II$QCONV + HomoTherm.II$QCONV_RESP, col = "black", pch = 16)

with(obs2, plot(T_O, -E * 4184 / 3600, ylim = c(-100, 250), xlim = c(15, 45),
                ylab = "QEVAP, W", xlab =
                  expression("operative temperature, "*degree*C), col = 'red',
                pch = 16, cex = 1.25, main = 'evaporation'))
points(HomoTherm.II$TOs, -(HomoTherm.II$QEVAP_RESP + HomoTherm.II$QEVAP_CUT),
       col = "black", pch = 16)
```

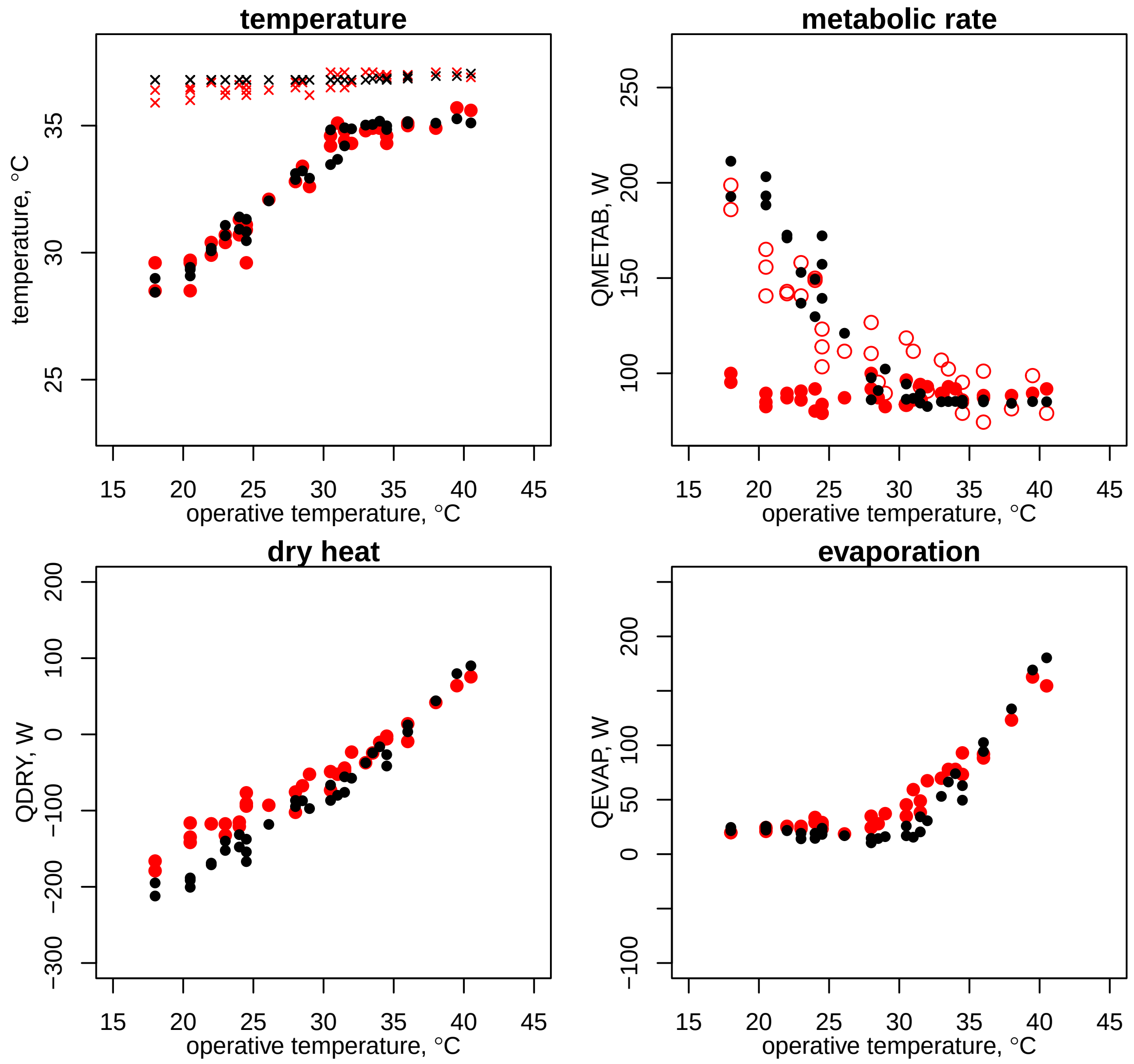

Now plot the correlations of predictions and observations.

```
par(mfrow = c(2, 2))
par(oma = c(4, 1, 1, 1) + 0.1) # margin spacing
par(mar = c(3, 3, 1, 1) + 0.1) # margin spacing
par(mgp = c(2, 1, 0) ) # margin spacing

with(all.II,
     plot(T_S, T_SKIN, ylim = c(23, 38), xlim = c(23, 38),
          ylab = expression("pred, "*degree*C), xlab = expression("obs, "*degree*C), col = 'black', pch
          cex = 1.25, main = 'temperature'))
with(all.II, points(T_B, T_CORE, col = "black", pch = 4, cex = 1.25))
abline(0, 1)
```

```r
with(all.II,
     plot(M * 4184 / 3600, QMETAB, ylim = c(70, 270), xlim = c(70, 270), ylab =
            "pred, W", xlab = "obs, W", col = 'black', pch = 16, cex = 1.25,
          main = 'metabolic rate'))
with(all.II, points((S + M) * 4184 / 3600, QMETAB, col = "black", pch = 1,
                    cex = 1.25))
abline(0, 1)

with(all.II,
     plot((C + R) * 4184 / 3600, QCONV_RESP + QCONV + QRAD_IN - QRAD_OUT,
          ylim = c(-300, 250), xlim =
            c(-300, 250), ylab = "pred, W", xlab = "obs, W", col = 'black',
          pch = 16, cex = 1.25, main = 'dry heat'))
abline(0, 1)

with(all.II,
     plot(-E * 4184 / 3600, -(QEVAP_RESP + QEVAP_CUT), ylim = c(-100, 250),
          xlim = c(-100, 250), ylab = "pred, W", xlab = "obs, W", col = 'black',
          pch = 16, cex = 1.25, main = 'evaporation'))
abline(0, 1)
```

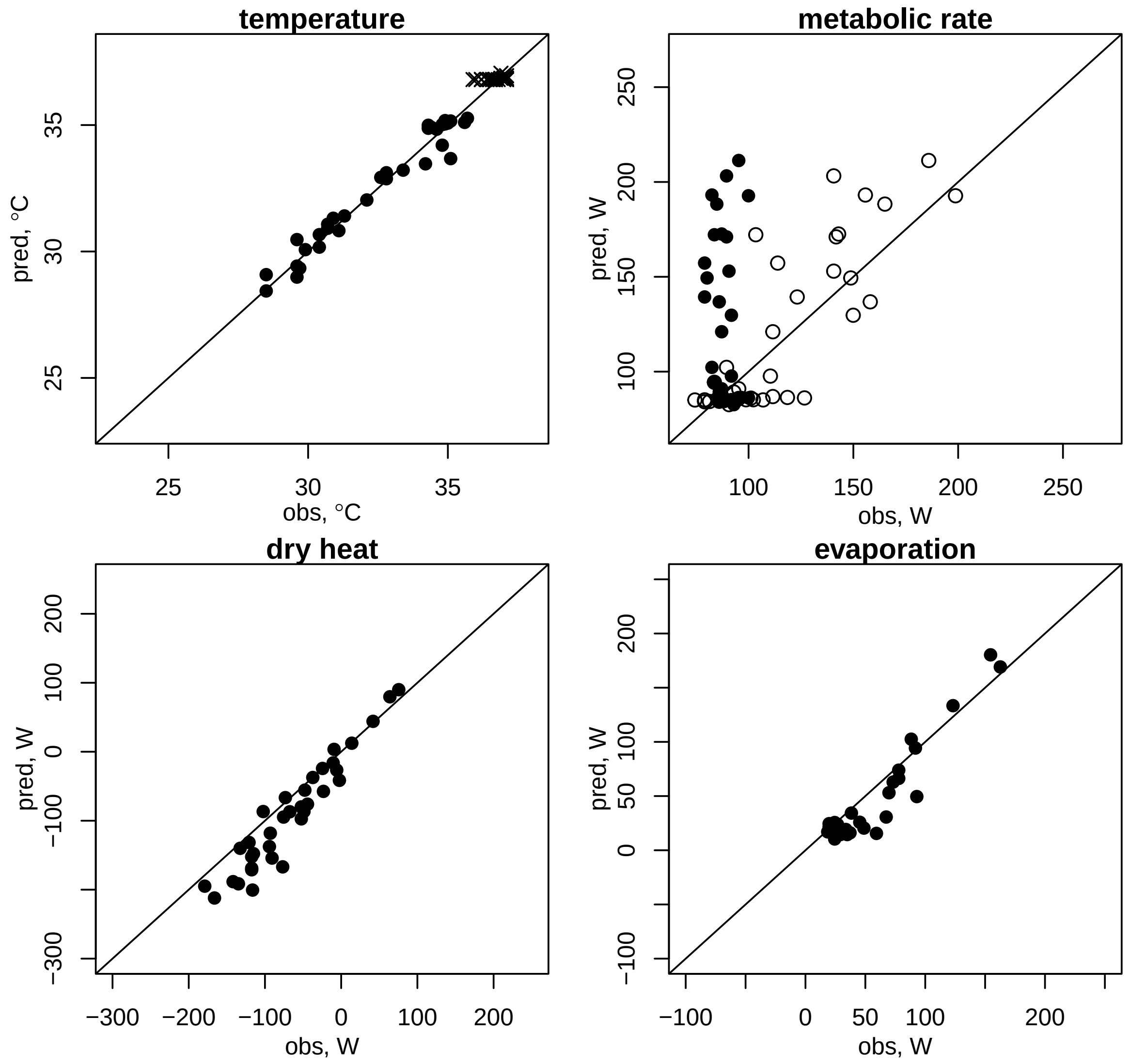

Discussion

Initially, it was difficult to reconcile the high skin temperatures but low metabolic rates in the cooler conditions. Low metabolic rate could be achieved by imposing vasoconstriction as the starting point (head and trunk flesh conductivity at 0.7 $W/m \cdot K$, arms and legs at 0.412 $W/m \cdot K$), and making the clothing depth of half of the trunk and legs 1 mm (perhaps justified by the effect of the chair). However, this produced much lower skin temperature than observed. The resolution of the issue comes from the fact that these measurements were not in steady state and the people were stated not to have been shivering - the authors note that the stout person was probably near the upper critical temperature and the lean person near the lower. If the storage term computed by Winslow et al. (1937) is added to the metabolic rate measured, then a very good match is obtained when starting in a non-vasoconstricted state (and reducing the fat layer to simulate bypassing of this insulation). This implies that the body was in a transient state of cooling, the peripheral parts of the body were cooling but the core wasn't yet affected, and in steady-state the metabolic

rate would have to have risen to avoid hypothermia.

Note that this paper is the origin of the concept of ‘operative temperature’, as in “physically operative”, as an effective radiant temperature.

References

Winslow, C.-E. A., L. P. Herrington, and A. P. Gagge. 1937. Physiological reactions of the human body to varying environmental temperatures. American Journal of Physiology-Legacy Content 120:1–22.
