# Supplementary material for: HomoTherm: An Open‐Source Approach to Modelling Heat Exchange in Humans and Other Hominins in Diverse Environments
Source: Glob Chang Biol. 2026 Apr 1;32(4):e70830. doi: 10.1111/gcb.70830 (PMC13044332; doi:10.1111/gcb.70830)
Supplement: Supplementary file 4 — Appendix S4: gcb70830‐sup‐0004‐Appendix 4.pdf. [file GCB-32-e70830-s005.pdf]

Test of HomoTherm, MANMO, HHB and PHS models against Mitchell et al. 1968

Michael Kearney

2026-01-12

Overview

A test of the HomoTherm, MANMO (Myrup and Morgan, 1972), PHS (Predictive Heat Strain model; Malchaire et al. 2001) and HHB (Human Heat Budget model; Vanos et al, 2023) models against the data reported in Mitchell et al. (1968) for two heat-acclimated individuals under a range of blackbody thermal conditions with low humidity and different wind speeds.

Load the libraries and scripts

```
library(NicheMapR)
library(comf)
```

```
## Warning: package 'comf' was built under R version 4.5.2
```

```
localpath <- 'c:/Users/mrke/Dropbox/Current Research Projects/mammal_projects/manmo analysis/'
source(paste0(localpath, 'code/MANMO/MANMO_R.R')) # the MANMO function
source(paste0(localpath, 'code/MANMO/run.MANMO.R'))
source(paste0(localpath, 'code/HHB/HHB.R'))
source(paste0(localpath, 'code/HHB/run_HHB.R'))
source(paste0(localpath, 'code/PHS/calcIso7933_Tcl.R'))
```

Load the observations

```
# read in data
source(paste0(localpath, 'code/tests/Mitchell1968/read_Mitchell_data.R'))
```

Environmental conditions

```
# environmental variables, Table 2
TAs <- c(12.8, 23.9, 35.3, 40.4, 49.1) # dry bulb temperatures, °C
TWbs <- c(6.7, 13.3, 17.8, 19.7, 23.2) # air temperatures, °C
RHs <- WETAIR(db = TAs, wb = TWbs, rh = -1)$rh  # relative humidities, %
TAs2 <- rep(TAs, 2)
TAs2 <-TAs2[order(TAs2)]
VELs.all <- c(0.67, 1.27, 1.86, 3.11, 4.94) # wind speeds, m/s
```

Set parameters

Men stated to be acclimated to heat - might be manifested in the high starting values of KFLESHs, higher TC_INCs, lower KFLESH_INCs, all of which help match observed skin temperatures, esp. at high temps. Note that starting value of PCTWET needs to be lower than default.

```
# person parameters
# subjects D & M
HEIGHT_D <- 165.5
MASS_D <- 62.2
AREA_D <- 0.00718 * MASS_D ^ 0.425 * HEIGHT_D ^ 0.725 # DuBois area, m2
AREA.measured_D <- 2.02
AREA.radiant_D <- 1.63
HEIGHT_M <- 166.2
MASS_M <- 53.9
AREA_M <- 0.00718 * MASS_M ^ 0.425 * HEIGHT_M ^ 0.725 # DuBois area, m2
AREA.measured_M <- 1.88
AREA.radiant_M <- 1.53
AREA.measured <- (AREA.measured_D + AREA.measured_M) / 2
AREA.radiant <- (AREA.radiant_D + AREA.radiant_M) / 2
AREA <- (AREA_D + AREA_M) / 2
MASS <- (MASS_D + MASS_M) / 2
HEIGHT <- (HEIGHT_D + HEIGHT_M) / 2

QMETAB_REST <- 34.8 * 4184 / 3600 * AREA * 1.07 # basal metabolic rate, W
INSDEPDs <- c(0.01, 1e-9, 1e-9, 1e-9) # fur depth, dorsal (m)
INSDEPVs <- c(1e-9, 1e-9, 1e-9, 1e-9) # fur depth, ventral (m)
```

Run simulations.

```
plot_vels <- 1
grp <- c(1, 1, 2, 2, 3, 3, 4, 4, 5, 5)

results <- matrix(data = NA, nrow = length(TAs) * length(VELs.all), ncol = 26)

par(oma = c(4, 1, 1, 1) + 0.1) # margin spacing
par(mar = c(3, 3, 1, 1) + 0.1) # margin spacing
par(mgp = c(2, 1, 0) ) # margin spacing

for(i in 1:4){
  par(mfrow = c(1, 2))
  if(i == 1){
    plot.manmo <- 0
    plot.HomoTherm <- 1
    plot.ISO <- 0
    plot.HHB <- 0
  }
  if(i == 2){
    plot.manmo <- 1
    plot.HomoTherm <- 0
    plot.ISO <- 0
    plot.HHB <- 0
  }
  if(i == 3){
```

```r
    plot.manmo <- 0
    plot.HomoTherm <- 0
    plot.ISO <- 1
    plot.HHB <- 0
  }
  if(i == 4){
    plot.manmo <- 0
    plot.HomoTherm <- 0
    plot.ISO <- 0
    plot.HHB <- 1
  }
  for(k in 1:length(VELs.all)){
    if(k == 1){
      Mitchell_M <- aggregate(Mitchell1968_5a_M$M, by = list(grp),
                              FUN = 'mean')$x
      Mitchell_E <- aggregate(Mitchell1968_5a_E$E, by = list(grp),
                              FUN = 'mean')$x
      Mitchell_C <- aggregate(Mitchell1968_5a_C$C, by = list(grp),
                              FUN = 'mean')$x
      Mitchell_R <- aggregate(Mitchell1968_5a_R$R, by = list(grp),
                              FUN = 'mean')$x
      Mitchell_Tr <- aggregate(Mitchell1968_5a_Tr$Tr, by = list(grp),
                               FUN = 'mean')$x
      Mitchell_Ts <- aggregate(Mitchell1968_5a_Ts$Ts, by = list(grp),
                               FUN = 'mean')$x
    }
    if(k == 2){
      Mitchell_M <- aggregate(Mitchell1968_5b_M$M, by = list(grp),
                              FUN = 'mean')$x
      Mitchell_E <- aggregate(Mitchell1968_5b_E$E, by = list(grp),
                              FUN = 'mean')$x
      Mitchell_C <- aggregate(Mitchell1968_5b_C$C, by = list(grp),
                              FUN = 'mean')$x
      Mitchell_R <- aggregate(Mitchell1968_5b_R$R, by = list(grp),
                              FUN = 'mean')$x
      Mitchell_Tr <- aggregate(Mitchell1968_5b_Tr$Tr, by = list(grp),
                               FUN = 'mean')$x
      Mitchell_Ts <- aggregate(Mitchell1968_5b_Ts$Ts, by = list(grp),
                               FUN = 'mean')$x
    }
    if(k == 3){
      Mitchell_M <- aggregate(Mitchell1968_5c_M$M, by = list(grp),
                              FUN = 'mean')$x
      Mitchell_E <- aggregate(Mitchell1968_5c_E$E, by = list(grp),
                              FUN = 'mean')$x
      Mitchell_C <- aggregate(Mitchell1968_5c_C$C, by = list(grp),
                              FUN = 'mean')$x
      Mitchell_R <- aggregate(Mitchell1968_5c_R$R, by = list(grp),
                              FUN = 'mean')$x
      Mitchell_Tr <- aggregate(Mitchell1968_5c_Tr$Tr, by = list(grp),
                               FUN = 'mean')$x
      Mitchell_Ts <- aggregate(Mitchell1968_5c_Ts$Ts, by = list(grp),
                               FUN = 'mean')$x
```

```
  }
  if(k == 4){
    Mitchell_M <- aggregate(Mitchell1968_5d_M$M, by = list(grp),
                            FUN = 'mean')$x
    Mitchell_E <- aggregate(Mitchell1968_5d_E$E, by = list(grp),
                            FUN = 'mean')$x
    Mitchell_C <- aggregate(Mitchell1968_5d_C$C, by = list(grp),
                            FUN = 'mean')$x
    Mitchell_R <- aggregate(Mitchell1968_5d_R$R, by = list(grp),
                            FUN = 'mean')$x
    Mitchell_Tr <- aggregate(Mitchell1968_5d_Tr$Tr, by = list(grp),
                             FUN = 'mean')$x
    Mitchell_Ts <- aggregate(Mitchell1968_5d_Ts$Ts, by = list(grp),
                             FUN = 'mean')$x
  }
  if(k == 5){
    Mitchell_M <- aggregate(Mitchell1968_5e_M$M, by = list(grp),
                            FUN = 'mean')$x
    Mitchell_E <- aggregate(Mitchell1968_5e_E$E, by = list(grp),
                            FUN = 'mean')$x
    Mitchell_C <- aggregate(Mitchell1968_5e_C$C, by = list(grp),
                            FUN = 'mean')$x
    Mitchell_R <- aggregate(Mitchell1968_5e_R$R, by = list(grp),
                            FUN = 'mean')$x
    Mitchell_Tr <- aggregate(Mitchell1968_5e_Tr$Tr, by = list(grp),
                             FUN = 'mean')$x
    Mitchell_Ts <- aggregate(Mitchell1968_5e_Ts$Ts, by = list(grp),
                             FUN = 'mean')$x
  }
  VELs <- rep(VELs.all[k], length(TAs)) # wind speeds, m/s

  cal2W <- 1.162222 # kcal / h / m2 to W / m2
  Mitchell_M <- Mitchell_M * -1 * cal2W
  Mitchell_E <- Mitchell_E * cal2W
  Mitchell_C <- Mitchell_C * cal2W
  Mitchell_R <- Mitchell_R * cal2W
  Mitchell_D <- Mitchell_C + Mitchell_R

  # run HomoTherm
  HomoTherm.out <- HomoTherm_var(MASS = MASS,
                                 QMETAB_REST = QMETAB_REST,
                                 INSDEPDs = INSDEPDs,
                                 INSDEPVs = INSDEPVs,
                                 TAs = TAs,
                                 RHs = RHs,
                                 VELs = VELs)
  HomoTherm.out.all <- HomoTherm.out
  HomoTherm.out <- HomoTherm.out.all$balance
  head.treg <- HomoTherm.out.all$head.treg
  trunk.treg <- HomoTherm.out.all$trunk.treg
  arm.treg <- HomoTherm.out.all$arm.treg
  leg.treg <- HomoTherm.out.all$leg.treg
```

```
HomoTherm_Ts <- HomoTherm.out$T_SKIN
HomoTherm_Tr <- HomoTherm.out$T_CORE
HomoTherm_M <- HomoTherm.out$QMETAB / AREA
HomoTherm_R <- (HomoTherm.out$QRAD_OUT - HomoTherm.out$QRAD_IN) / AREA
HomoTherm_C <- (HomoTherm.out$QCONV + HomoTherm.out$QCONV_RESP) * -1 / AREA
HomoTherm_E <- (HomoTherm.out$QEVAP_RESP + HomoTherm.out$QEVAP_CUT) * -1 / AREA
HomoTherm_D <- HomoTherm_C + HomoTherm_R

# run MANMO_R
G_m.G2s <- Mitchell_M  # enter observed metabolic rates into MANMO
#clo <- colMeans(get_clo(HomoTherm.out, INSDEPDs = INSDEPDs, INSDEPVs = INSDEPVs))
clo <- 0.1
MANMO.output <- run.MANMO(W = rep(1, length(TAs)) / 100,
                          Ht.H4 = HEIGHT,
                          Wt.W4 = MASS,
                          D3 = c(mean(INSDEPDs[2:4]), rep(1e-10, 3)),
                          Maximum.SR = MAXSWEATs[1] / 60 / AREA,
                          G_m.G2s = G_m.G2s,
                          CLO.C4 = clo,
                          CLO.mode = 0,
                          T_clo.T9s = HomoTherm.out$T_CLO,
                          TAs = TAs,
                          RH.H2s = RHs / 100,
                          VELs = VELs)
MANMO.Mitchell <- MANMO.output

manmo_Ts <- MANMO.Mitchell$Tskin
manmo_M <- MANMO.Mitchell$M_m.M / AREA
manmo_R <- MANMO.Mitchell$I_m.I * -1 / AREA
manmo_C <- MANMO.Mitchell$H_m.H * -1 / AREA
manmo_E <- MANMO.Mitchell$E_m.E * -1 / AREA
manmo_D <- manmo_C + manmo_R

HHB <- lapply(1:length(TAs),
              function(x){run_HHB(AD = AREA,
                                  M = G_m.G2s[x] * AREA,
                                  Tsk_C = Mitchell_Ts[x],
                                  Icl = clo,
                                  Ta_C = TAs[x],
                                  humidity = RHs[x],
                                  Av_ms = VELs[x],
                                  mrt_C = TAs[x],
                                  Mass = MASS
              )})
HHB.Mitchell <- as.data.frame(do.call(rbind, HHB))

HHB_E <- HHB.Mitchell$Ereq / AREA
HHB_CR <- HHB.Mitchell$Dry_Heat_Loss / AREA

# Iso7933
Iso7933 <- lapply(1:length(TAs),
                  function(x){calcIso7933_Tcl(accl = 100,
                                              posture = 1,
```

```
                                                  Ta = TAs[x],
                                                  Pa = WETAIR(db = TAs[x],
                                                              rh = RHs[x])$e
                                                  / 1000,
                                                  Tr = TAs[x],
                                                  Va = VELs[x],
                                                  Tsk = TAs[x],
                                                  Met = QMETAB_REST / AREA,
                                                  Icl = clo,
                                                  weight = MASS,
                                                  height = HEIGHT / 100,
                                                  Adu = AREA,
                                                  Tre = 36.8,
                                                  Tcr = 36.8,
                                                  SWp = 0.1)
                  })
ISO.Mitchell <- as.data.frame(do.call(rbind, Iso7933))
ISO_Ts <- ISO.Mitchell$Tskeq
ISO_Tr <- ISO.Mitchell$Tre
ISO_E <- ISO.Mitchell$Eres + ISO.Mitchell$SWp
ISO_D <- ISO.Mitchell$Dry
ISO_C <- ISO.Mitchell$Conv
ISO_R <- ISO.Mitchell$Rad

results[((k-1)*5+1):((k-1)*5+5), ] <- c(TAs, VELs, Mitchell_Ts, Mitchell_Tr,
                                        Mitchell_M, Mitchell_R, Mitchell_C,
                                        Mitchell_E, HomoTherm_Ts, HomoTherm_Tr,
                                        HomoTherm_M, HomoTherm_R, HomoTherm_C,
                                        HomoTherm_E, manmo_Ts, manmo_M, manmo_R,
                                        manmo_C, manmo_E, HHB_E, HHB_CR, ISO_Ts,
                                        ISO_Tr, ISO_R, ISO_C, ISO_E)

if(plot_vels){
  plot(TAs, Mitchell_Ts, pch = 17, col = 'red', type = 'b',
       ylab = expression("temperature, "*degree*C),
       xlab = expression("temperature, "*degree*C),
       ylim = c(15, 40), main = paste0(VELs.all[k], ' m/s'))
  if(plot.HomoTherm == 1){
    mtext('HomoTherm', side = 3, outer = TRUE)
    points(TAs, HomoTherm_Ts, pch = 17, type = 'b')
    legend(20, 25, c('Tskin', 'Trect'), pch = c(17, 2), bty = 'n', cex = 0.8)
  }
  if(plot.manmo == 1){
    mtext('MANMO', side = 3, outer = TRUE)
    points(TAs, manmo_Ts, pch = 17, type = 'b')
  }
  points(TAs, Mitchell_Tr, pch = 2, col = 'red')
  if(plot.HomoTherm == 1){
    points(TAs, HomoTherm_Tr, pch = 2, type = 'b')
  }
  if(plot.ISO == 1){
    mtext('PHS', side = 3, outer = TRUE)
    points(TAs, ISO_Ts, pch = 17, type = 'b')
```

```
  points(TAs, ISO_Tr, pch = 2, type = 'b')
}
plot(TAs, Mitchell_M, pch = 0, col = 'red', ylab = 'W / m^2',
     xlab = expression("temperature, "*degree*C), ylim = c(-350, 450))
legend(15, -75, c('Met', 'Dry', 'Evap'), pch = c(0, 16, 4),
       bty = 'n', cex = 0.8)
text(25, 450, 'observed', col = 2, cex = 0.85)
text(25, 400, 'predicted', col = 1, cex = 0.85)

if(plot.HomoTherm == 1){
  points(TAs, HomoTherm_M, pch = 0, type = 'b')
}
points(TAs, Mitchell_D, pch = 16, col = 'red')
if(plot.HomoTherm == 1){
  points(TAs, HomoTherm_D, pch = 16, type = 'b')
}
if(plot.manmo == 1){
  points(TAs, manmo_D, pch = 16, type = 'b')
}
if(plot.ISO == 1){
  points(TAs, ISO_D, pch = 16, type = 'b')
}
if(plot.HHB == 1){
  mtext('HHB', side = 3, outer = TRUE)
  points(TAs, HHB_CR, pch = 16, type = 'b')
}
# points(TAs, Mitchell_R, pch = 16, col = 'red')
# if(plot.HomoTherm == 1){
#   points(TAs, HomoTherm_R, pch = 16, type = 'b')
# }
# if(plot.manmo == 1){
#   points(TAs, manmo_R, pch = 16, col = 'grey', type = 'b')
# }
# points(TAs, Mitchell_C, pch = 1, col = 'red')
# if(plot.HomoTherm == 1){
#   points(TAs, HomoTherm_C, pch = 1, type = 'b')
# }
# if(plot.manmo == 1){
#   points(TAs, manmo_C, pch = 1, col = 'grey', type = 'b')
# }
points(TAs, Mitchell_E, pch = 4, col = 'red')
if(plot.HomoTherm == 1){
  points(TAs, HomoTherm_E, pch = 4, type = 'b')
}
if(plot.manmo == 1){
  points(TAs, manmo_E, pch = 4, type = 'b')
}
if(plot.ISO == 1){
  points(TAs, ISO_E, pch = 4, type = 'b')
}
if(plot.HHB == 1){
  points(TAs, HHB_E, pch = 4, type = 'b')
}
```

```
    }
  }
}
```

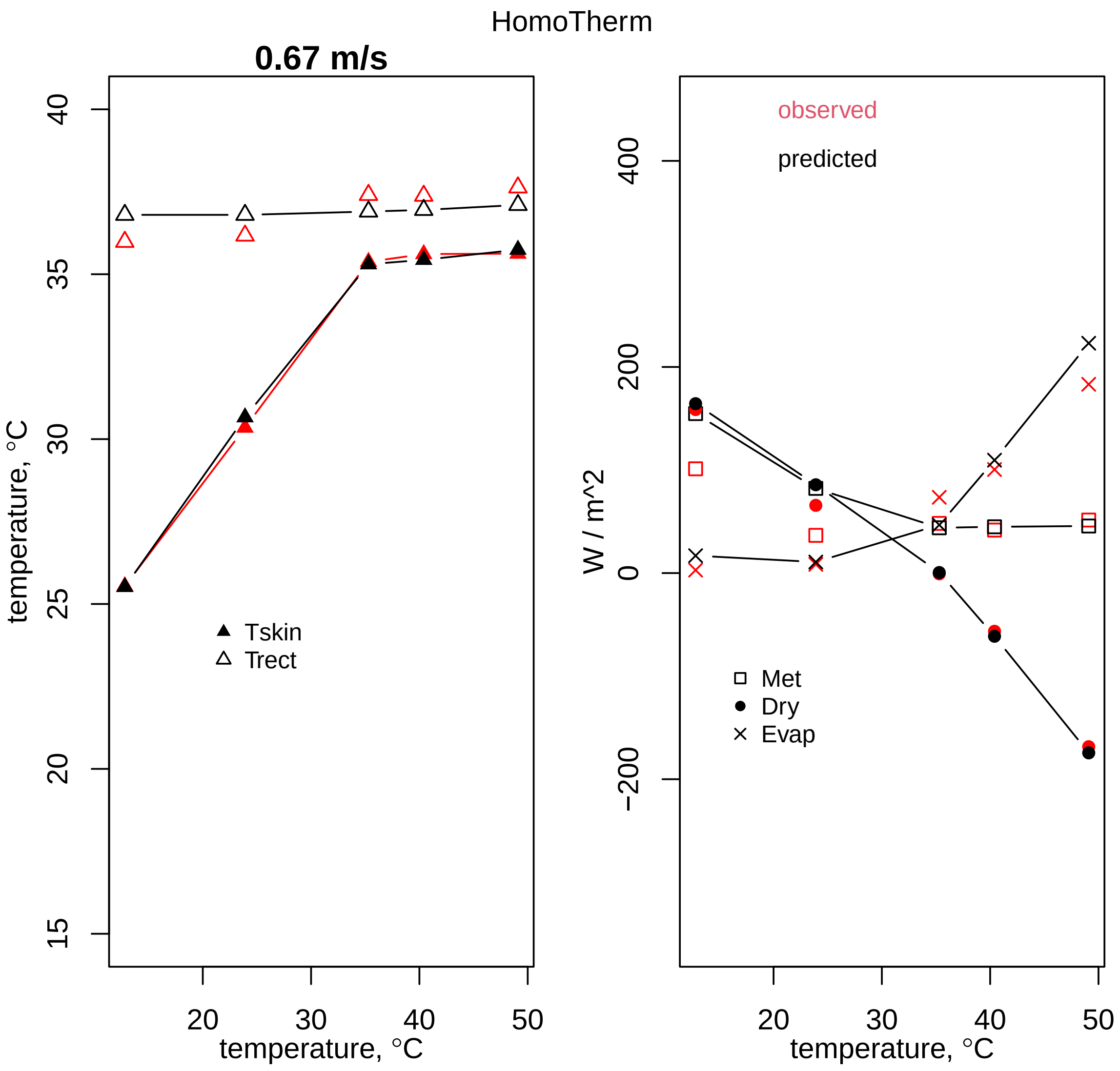

HomoTherm
0.67 m/s
temperature, °C
40
35
30
25
20
15
20
30
40
50
temperature, °C
Tskin
Trect
observed
predicted
W / m^2
400
200
0
−200
20
30
40
50
temperature, °C
Met
Dry
Evap

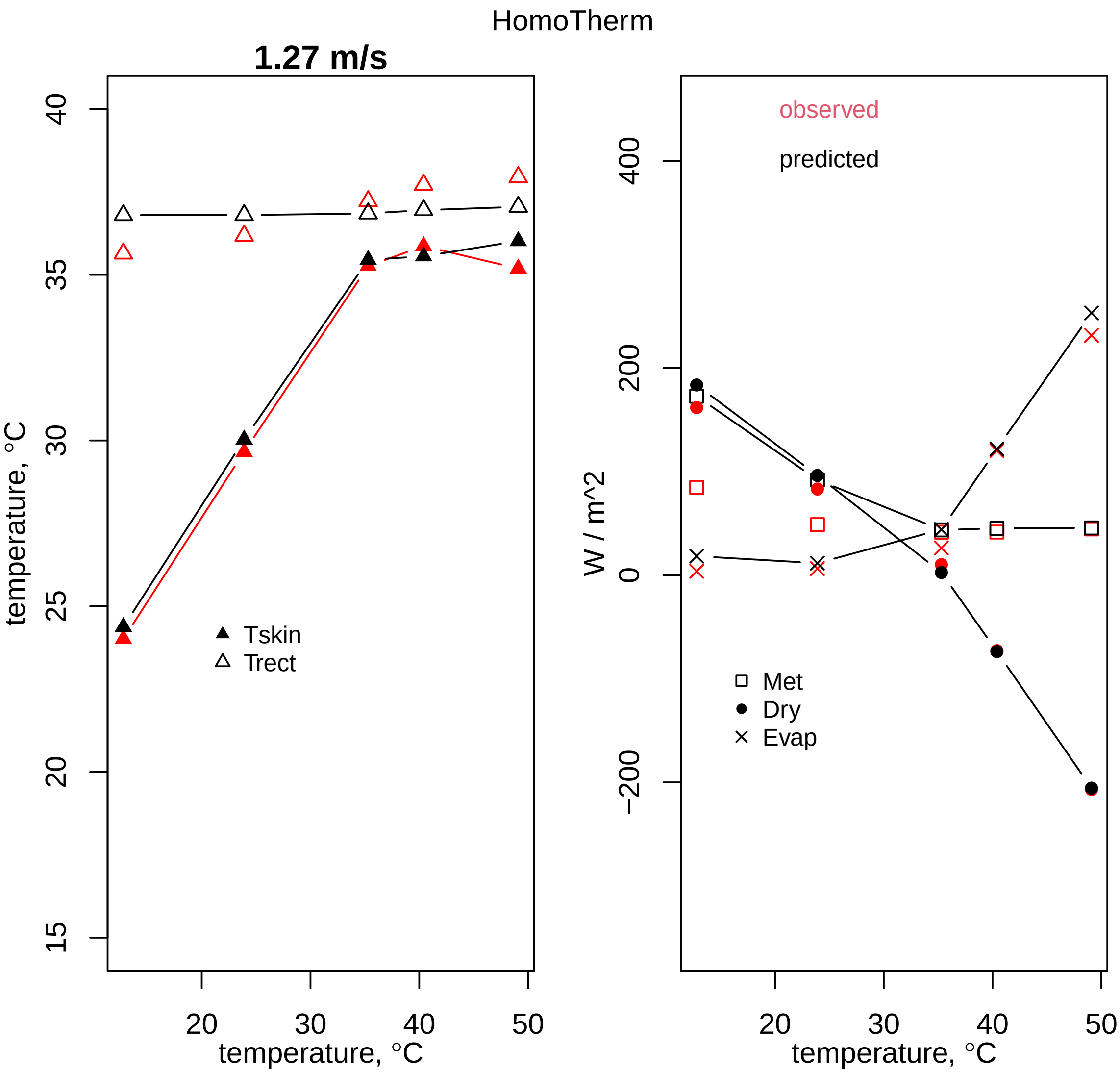

HomoTherm
1.27 m/s
temperature, °C
15
20
25
30
35
40
20
30
40
50
temperature, °C
Tskin
Trect
observed
predicted
W / m^2
−200
0
200
400
Met
Dry
Evap
20
30
40
50
temperature, °C

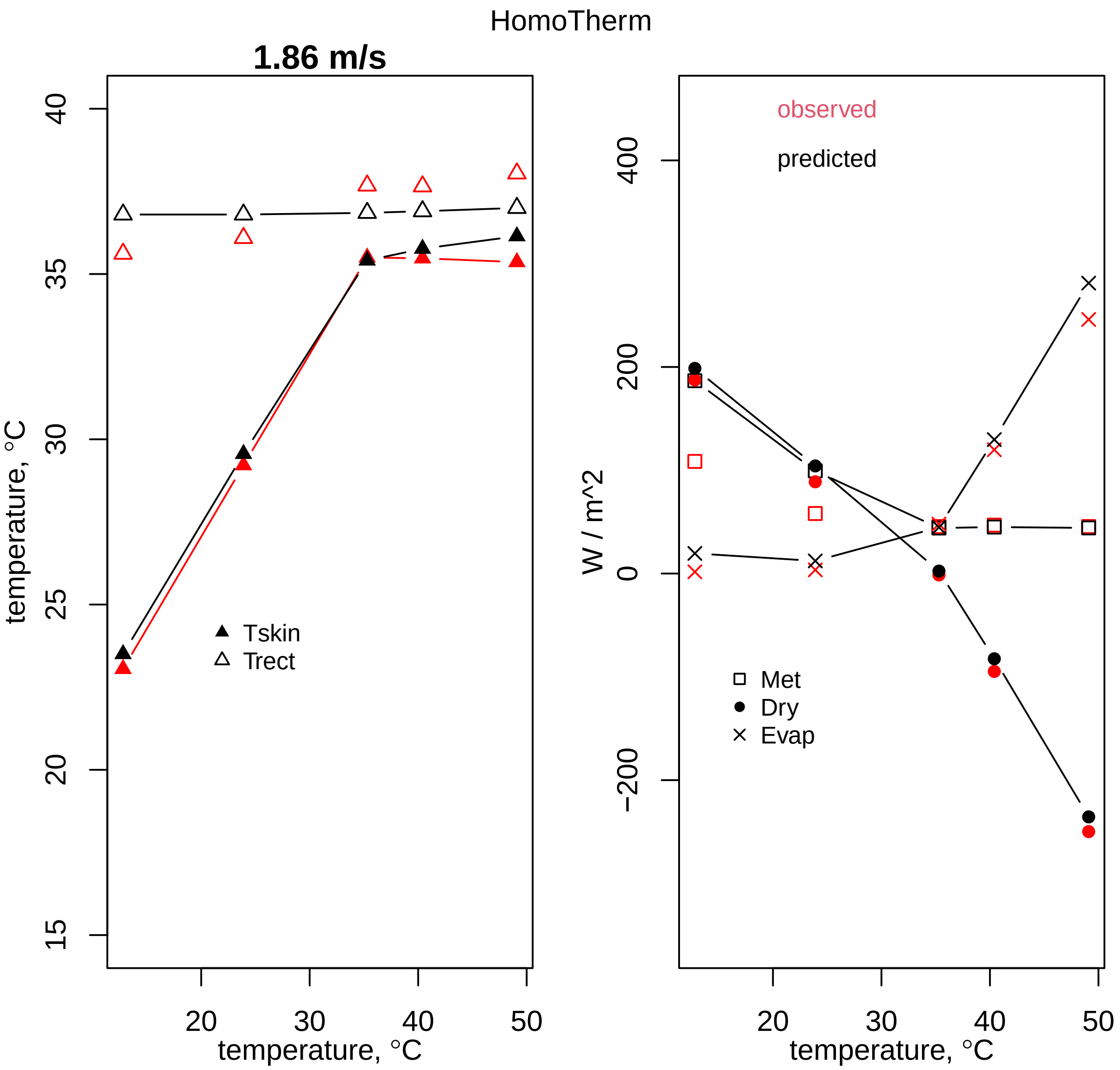
HomoTherm
1.86 m/s
temperature, °C
Tskin
Trect
observed
predicted
W / m^2
Met
Dry
Evap
temperature, °C
temperature, °C
40
35
30
25
20
15
400
200
0
−200
20
30
40
50
20
30
40
50

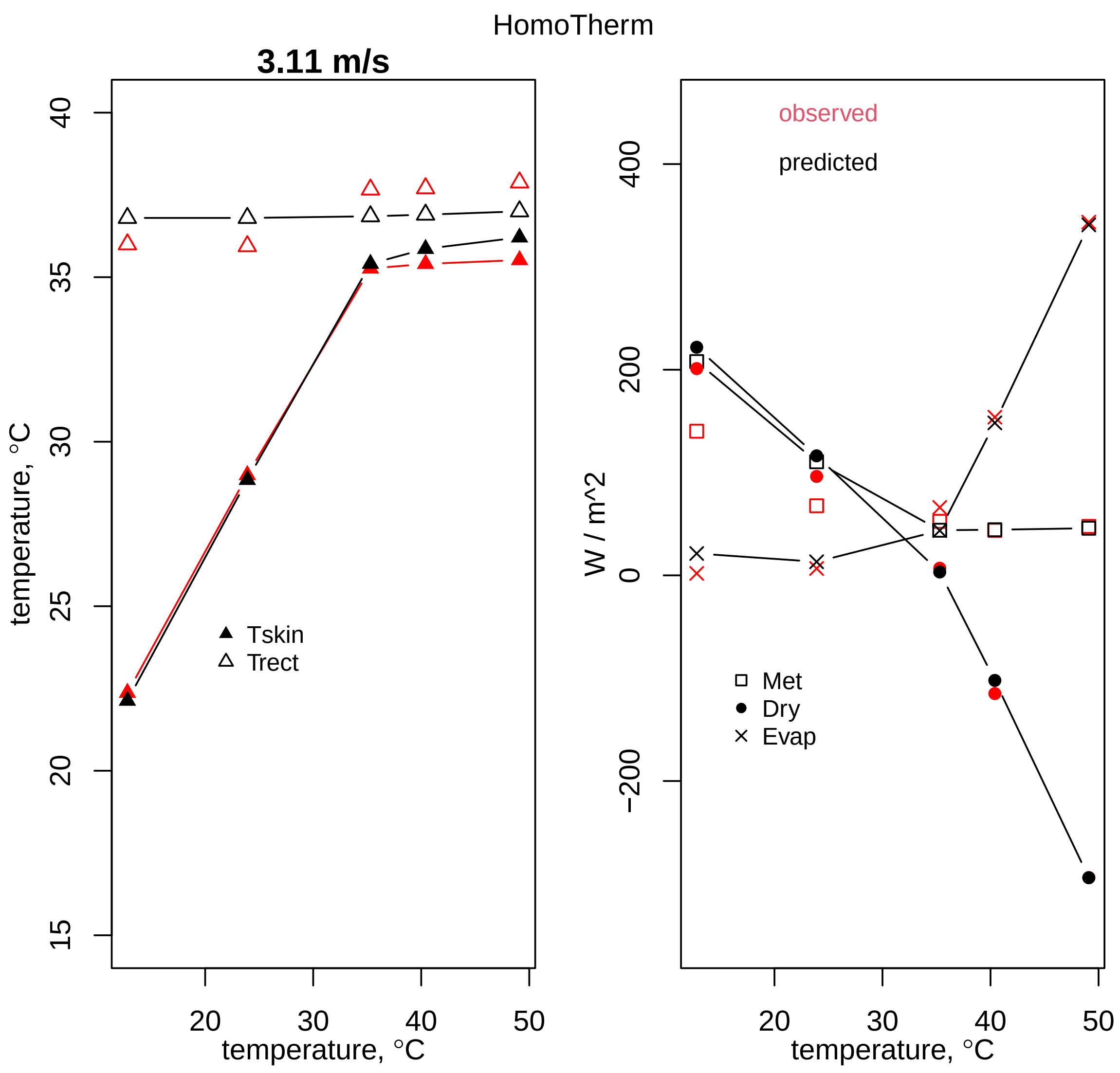
HomoTherm
3.11 m/s
temperature, °C
Tskin
Trect
15
20
25
30
35
40
20
30
40
50
temperature, °C
W / m^2
observed
predicted
Met
Dry
Evap
−200
0
200
400
20
30
40
50
temperature, °C

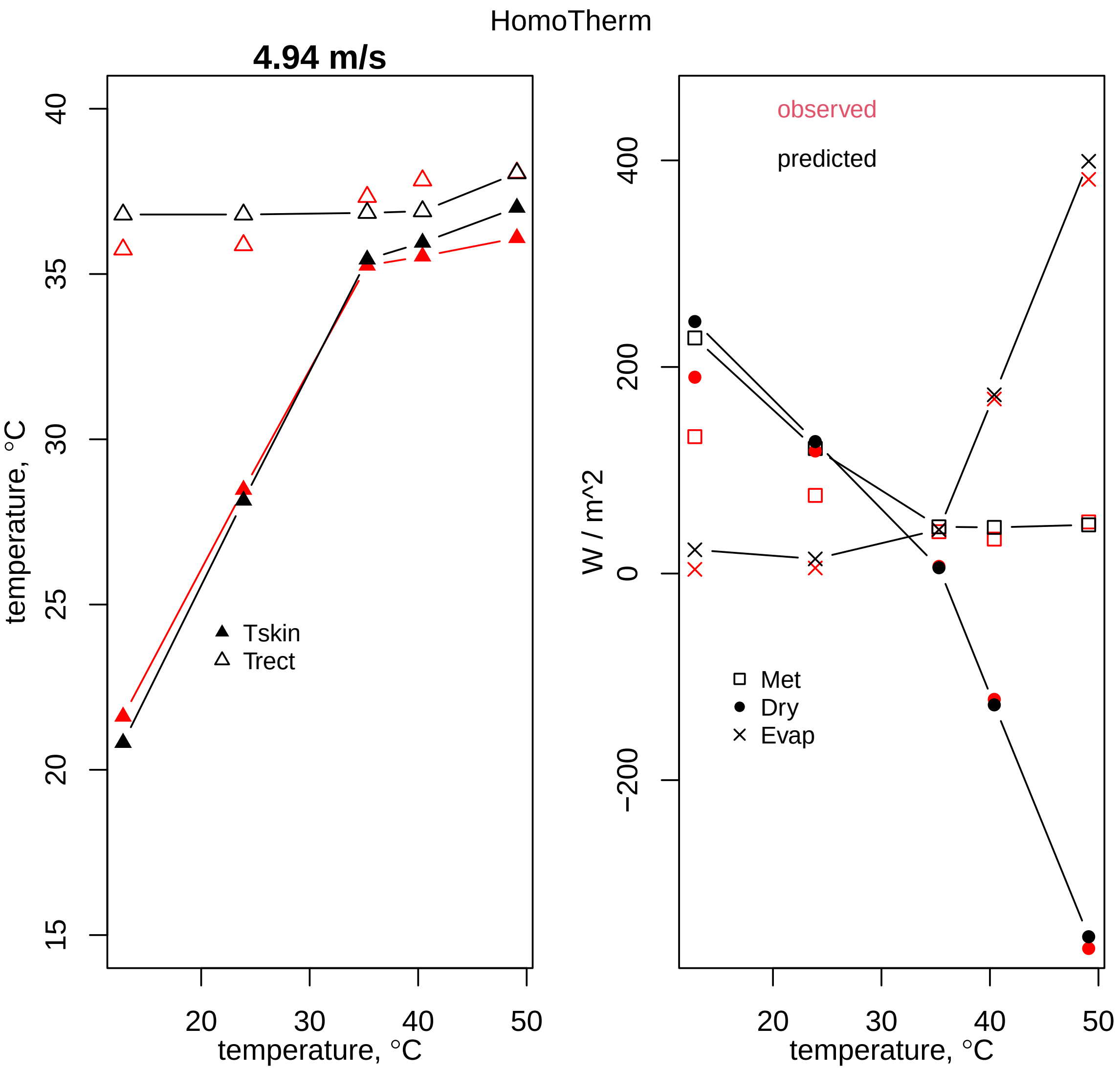

HomoTherm
4.94 m/s
temperature, °C
40
35
30
25
20
15
Tskin
Tract
20
30
40
50
temperature, °C
observed
predicted
W / m^2
400
200
0
−200
Met
Dry
Evap
20
30
40
50
temperature, °C

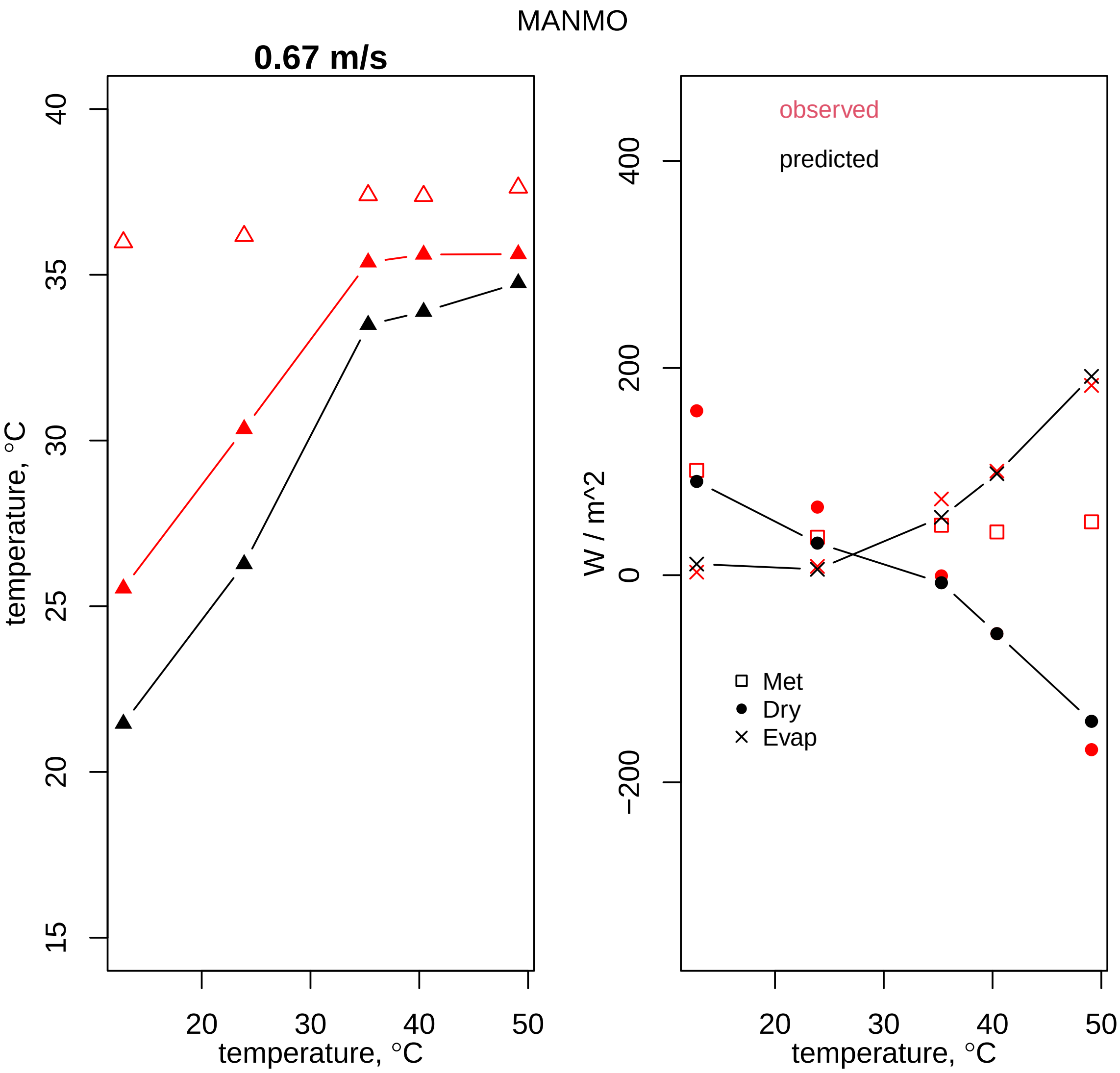

MANMO
0.67 m/s
temperature, °C
15
20
25
30
35
40
20
30
40
50
temperature, °C
observed
predicted
Met
Dry
Evap
W / m^2
−200
0
200
400
20
30
40
50
temperature, °C

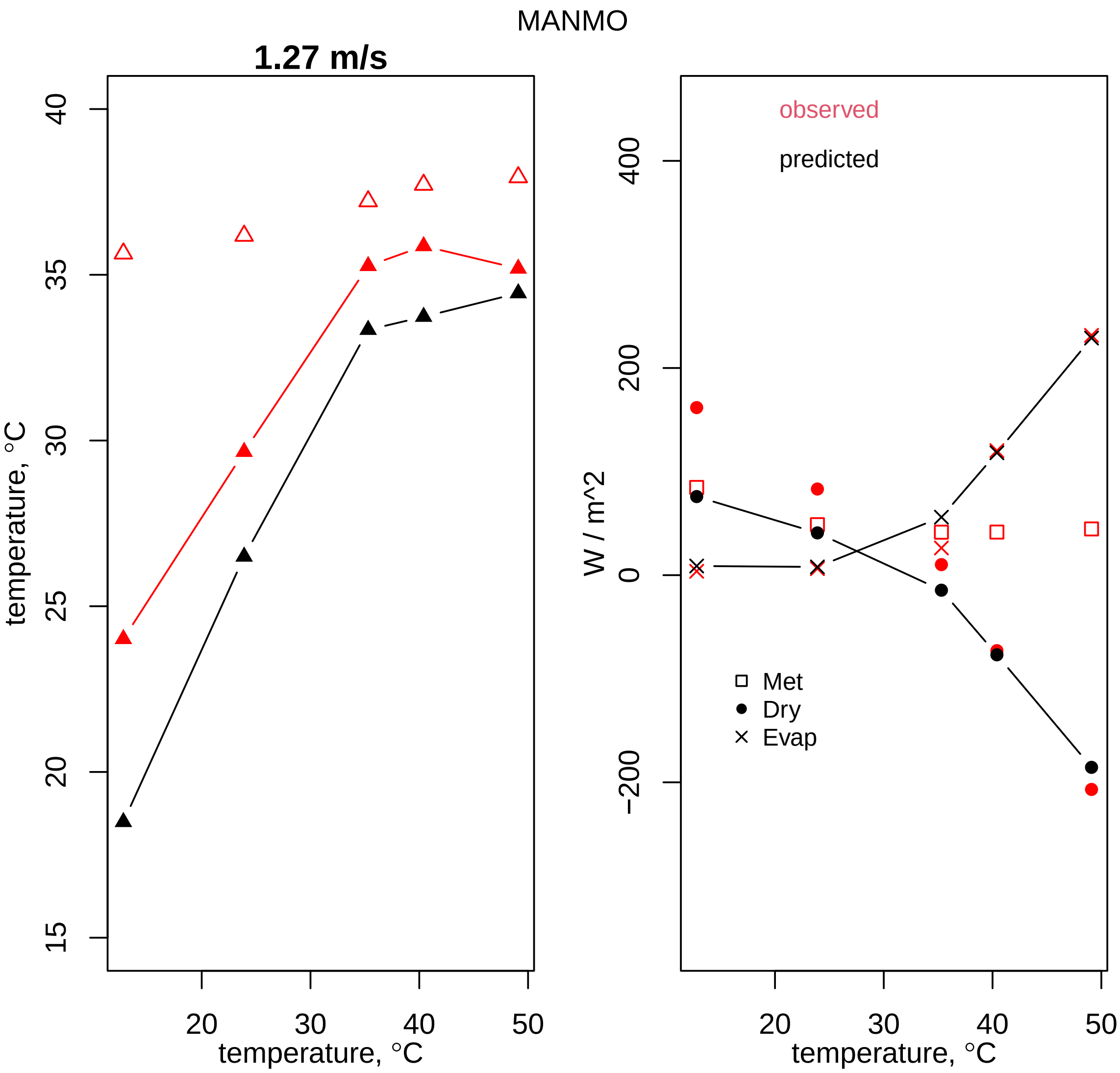

MANMO
1.27 m/s
temperature, °C
15
20
25
30
35
40
20
30
40
50
temperature, °C
W / m^2
−200
0
200
400
observed
predicted
Met
Dry
Evap
20
30
40
50
temperature, °C

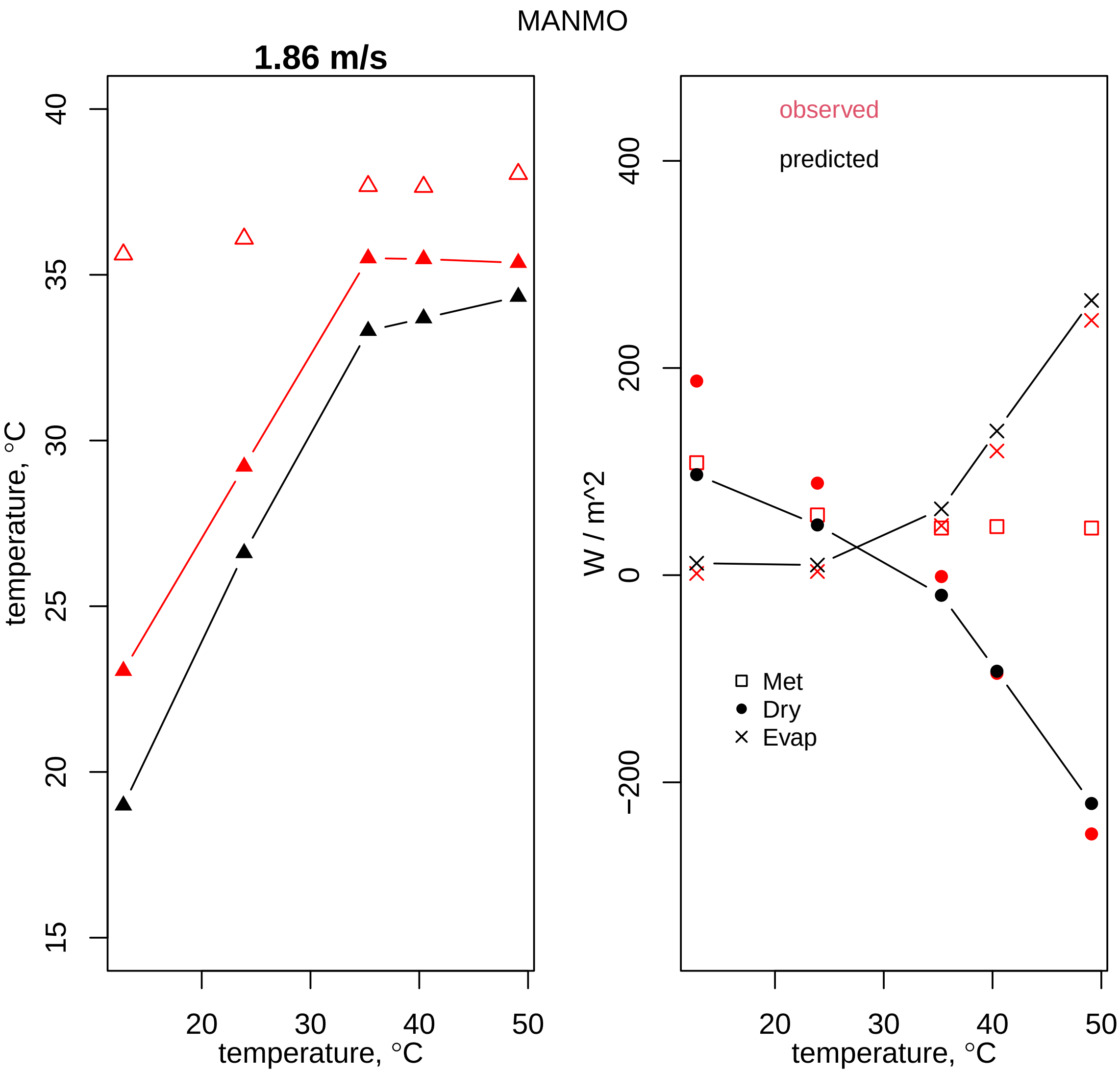

MANMO
1.86 m/s
temperature, °C
40
35
30
25
20
15
20
30
40
50
temperature, °C
observed
predicted
W / m^2
400
200
0
−200
Met
Dry
Evap
20
30
40
50
temperature, °C

MANMO

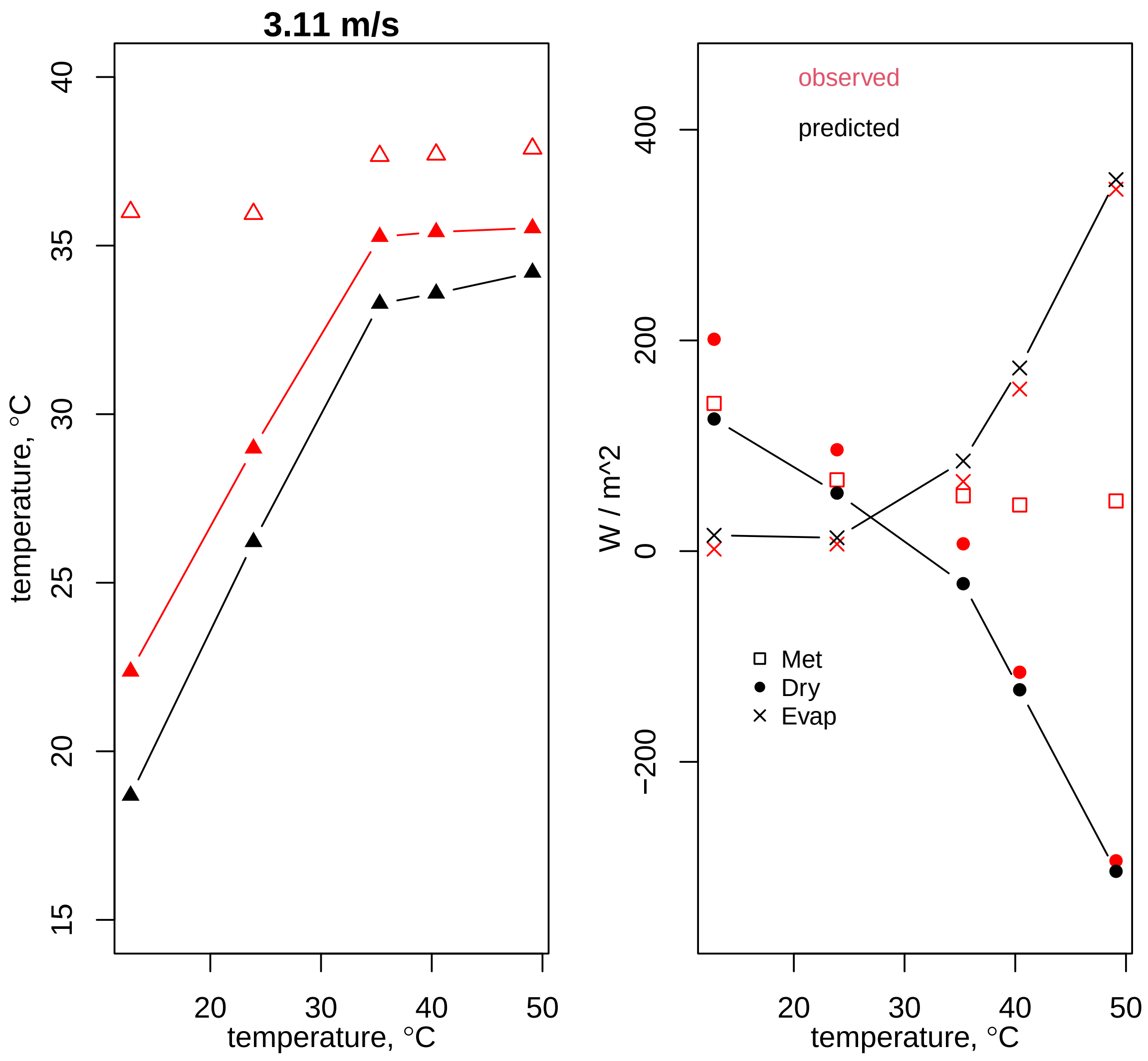
3.11 m/s
temperature, °C
15
20
25
30
35
40
20
30
40
50
temperature, °C
observed
predicted
W / m^2
−200
0
200
400
Met
Dry
Evap
20
30
40
50
temperature, °C

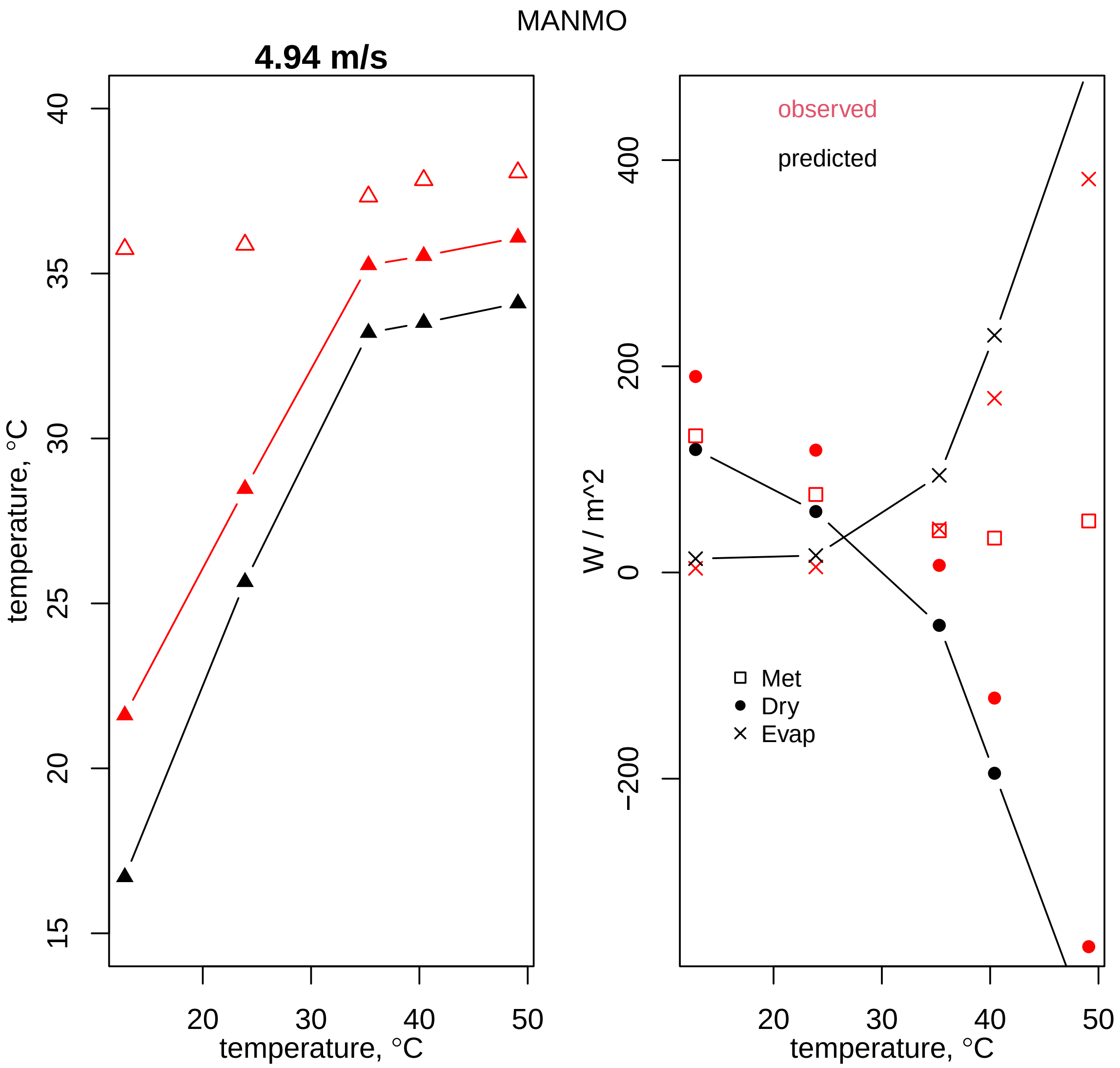

MANMO
4.94 m/s
temperature, °C
temperature, °C
15
20
25
30
35
40
20
30
40
50
W / m^2
−200
0
200
400
observed
predicted
Met
Dry
Evap
temperature, °C
20
30
40
50

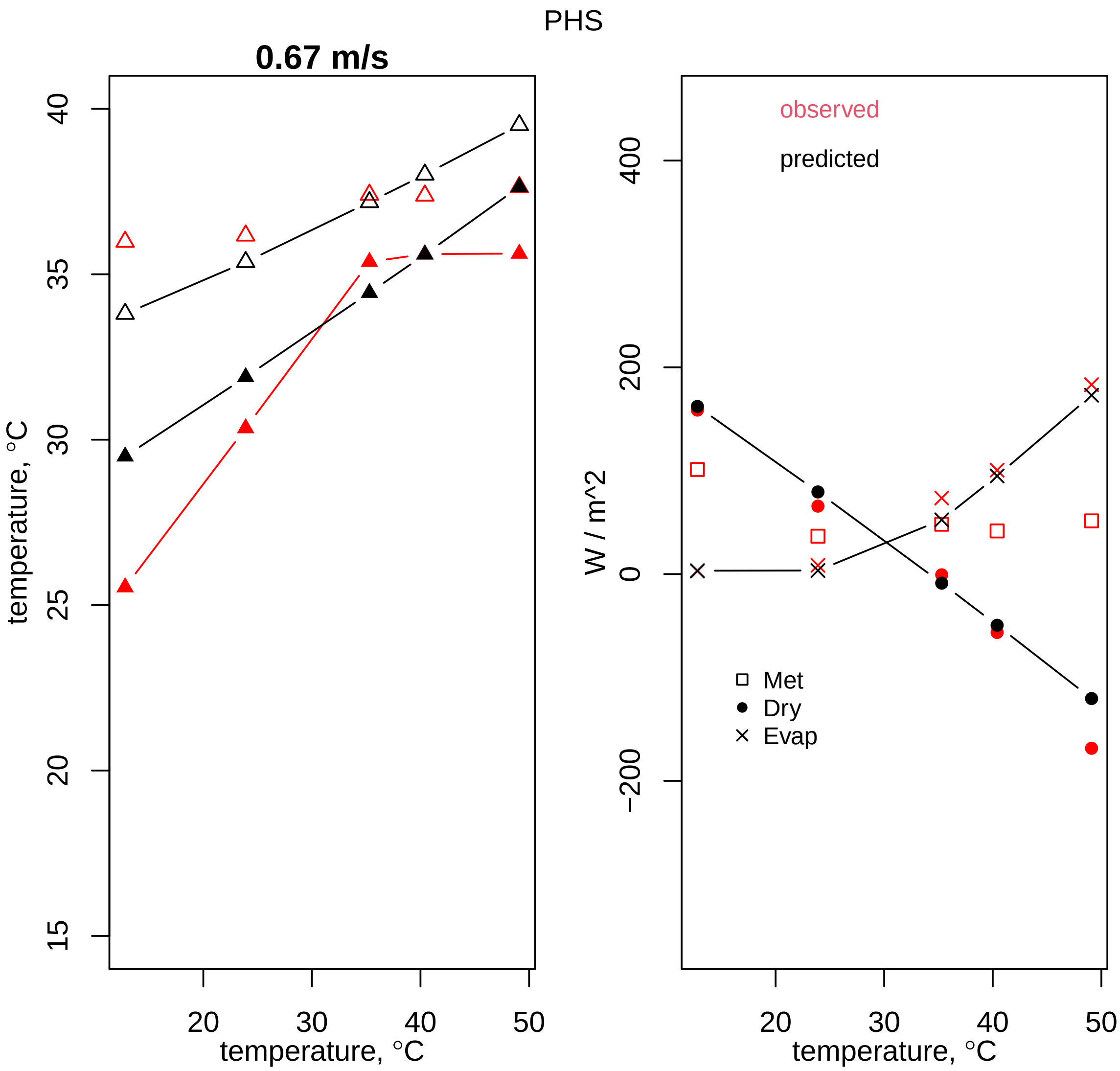

PHS
0.67 m/s
temperature, °C
15
20
25
30
35
40
20
30
40
50
temperature, °C
observed
predicted
W / m^2
−200
0
200
400
Met
Dry
Evap
20
30
40
50
temperature, °C

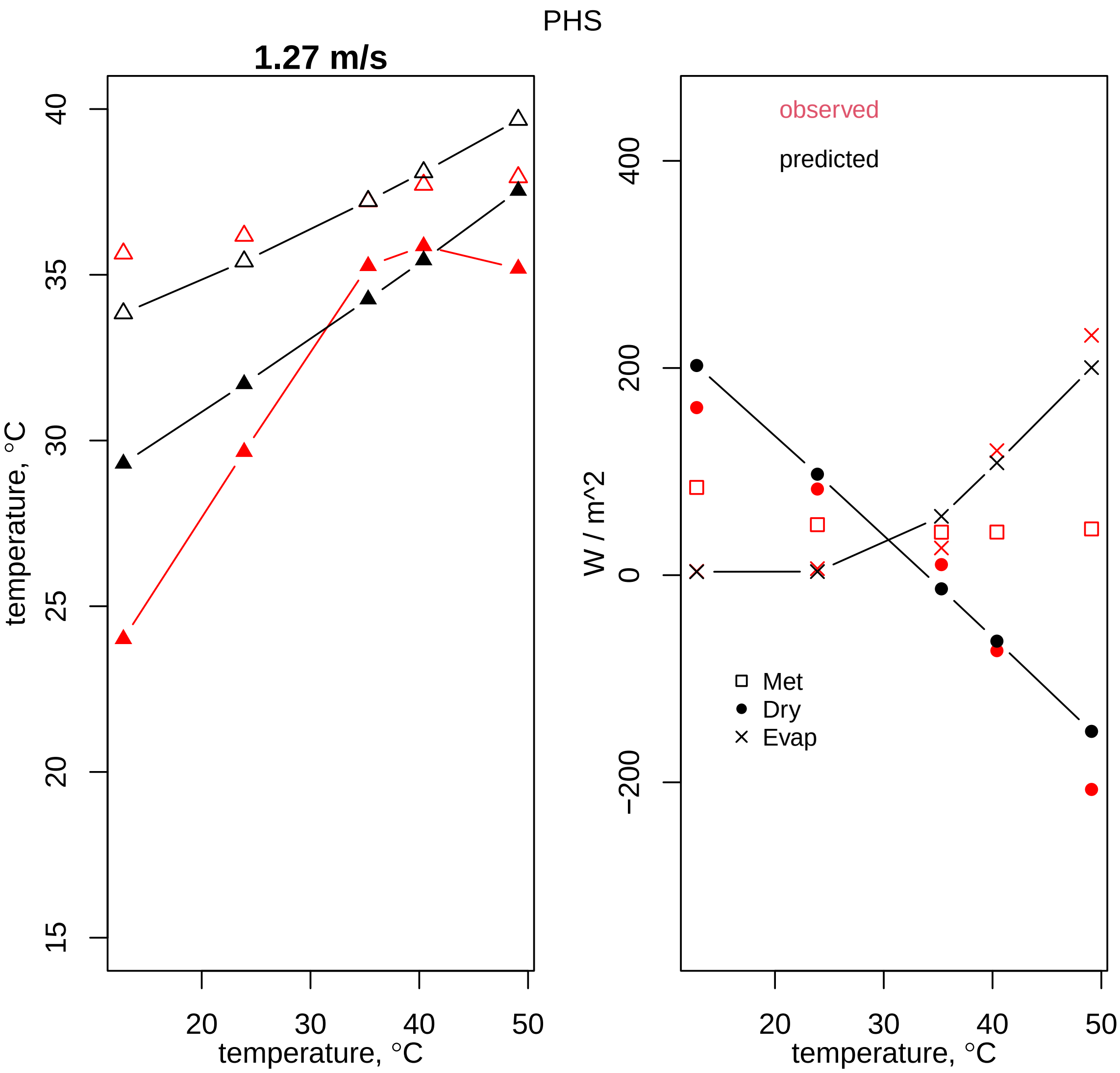

PHS
1.27 m/s
temperature, °C
15
20
25
30
35
40
20
30
40
50
temperature, °C
observed
predicted
W / m^2
−200
0
200
400
Met
Dry
Evap
20
30
40
50
temperature, °C

PHS

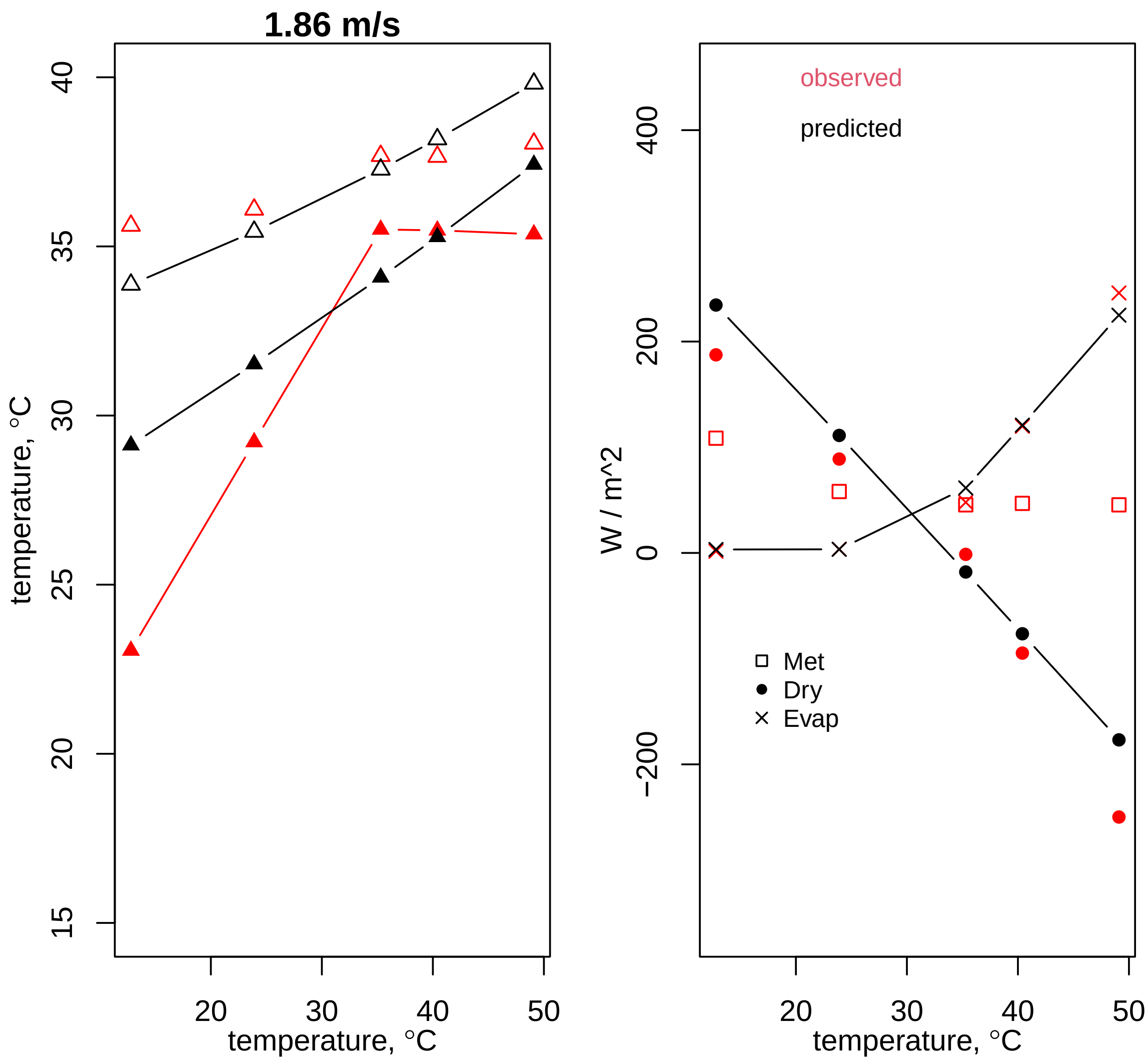
1.86 m/s
temperature, °C
40
35
30
25
20
15
20
30
40
50
temperature, °C
observed
predicted
W / m^2
400
200
0
−200
Met
Dry
Evap
20
30
40
50
temperature, °C

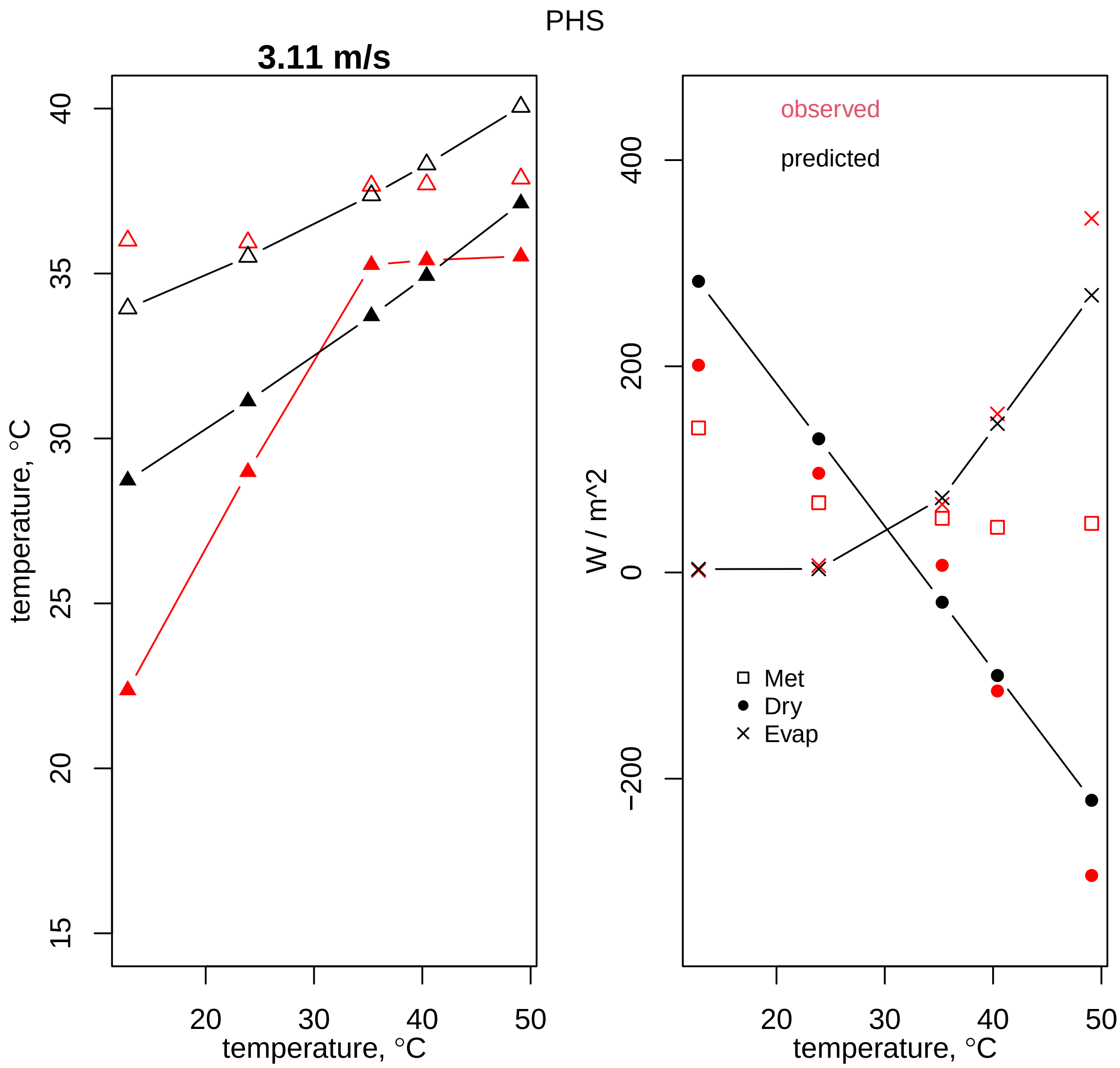
PHS
3.11 m/s
temperature, °C
15
20
25
30
35
40
20
30
40
50
temperature, °C
observed
predicted
W / m^2
−200
0
200
400
Met
Dry
Evap
20
30
40
50
temperature, °C

PHS

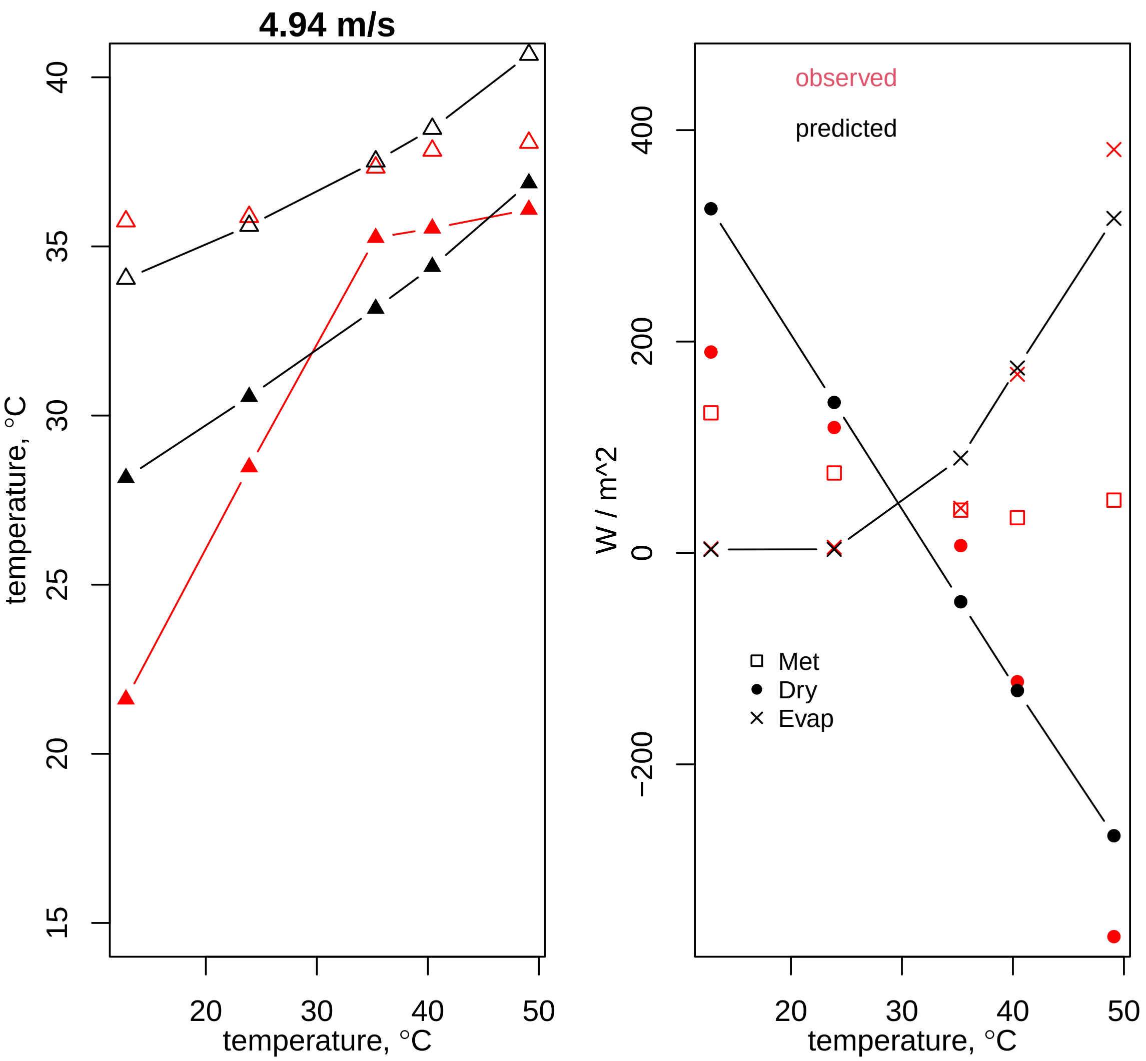
4.94 m/s
temperature, °C
temperature, °C
15
20
25
30
35
40
20
30
40
50
observed
predicted
W / m^2
−200
0
200
400
Met
Dry
Evap
temperature, °C
20
30
40
50

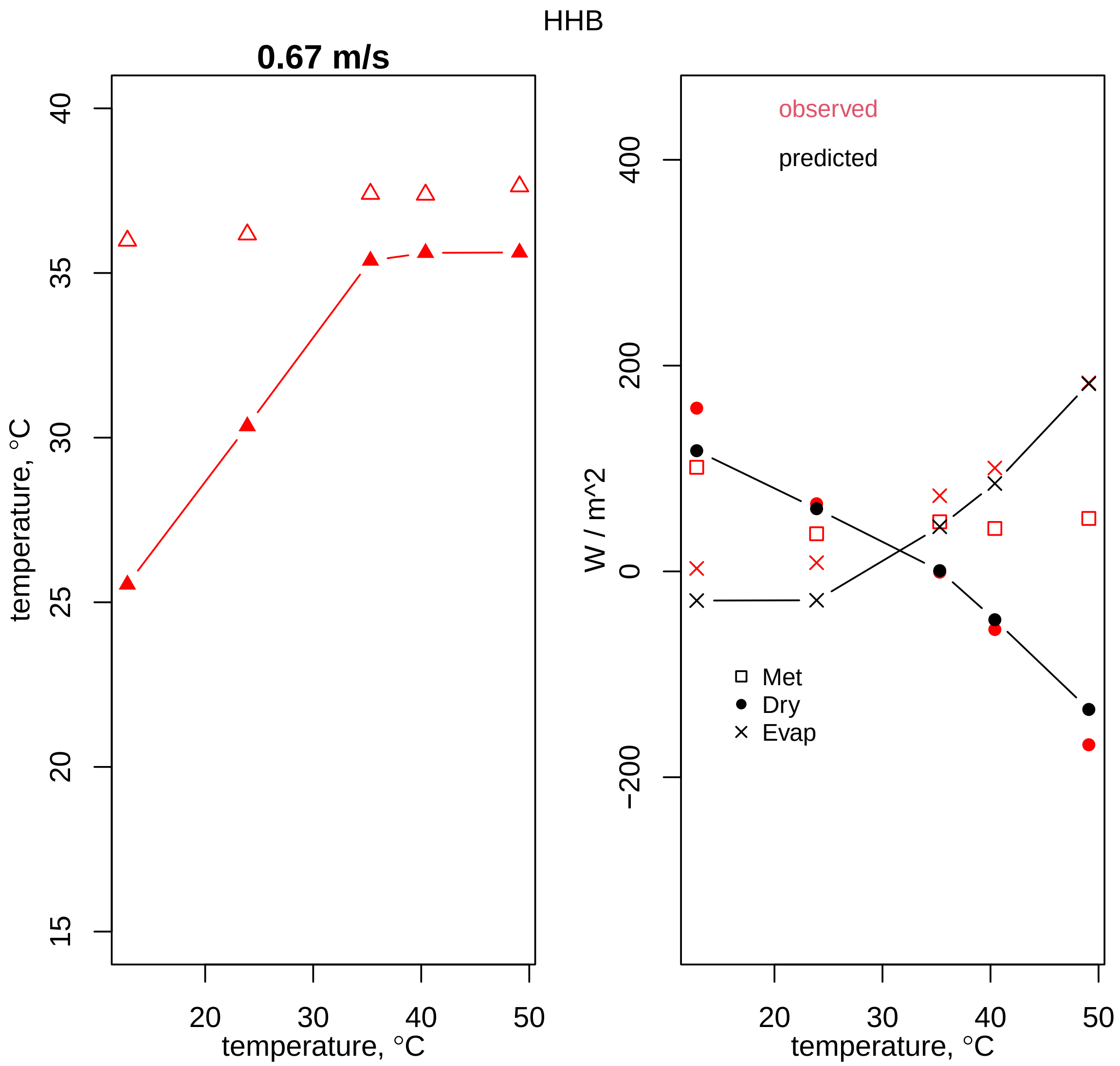
HHB
0.67 m/s
temperature, °C
15
20
25
30
35
40
20
30
40
50
temperature, °C
observed
predicted
W / m^2
−200
0
200
400
Met
Dry
Evap
20
30
40
50
temperature, °C

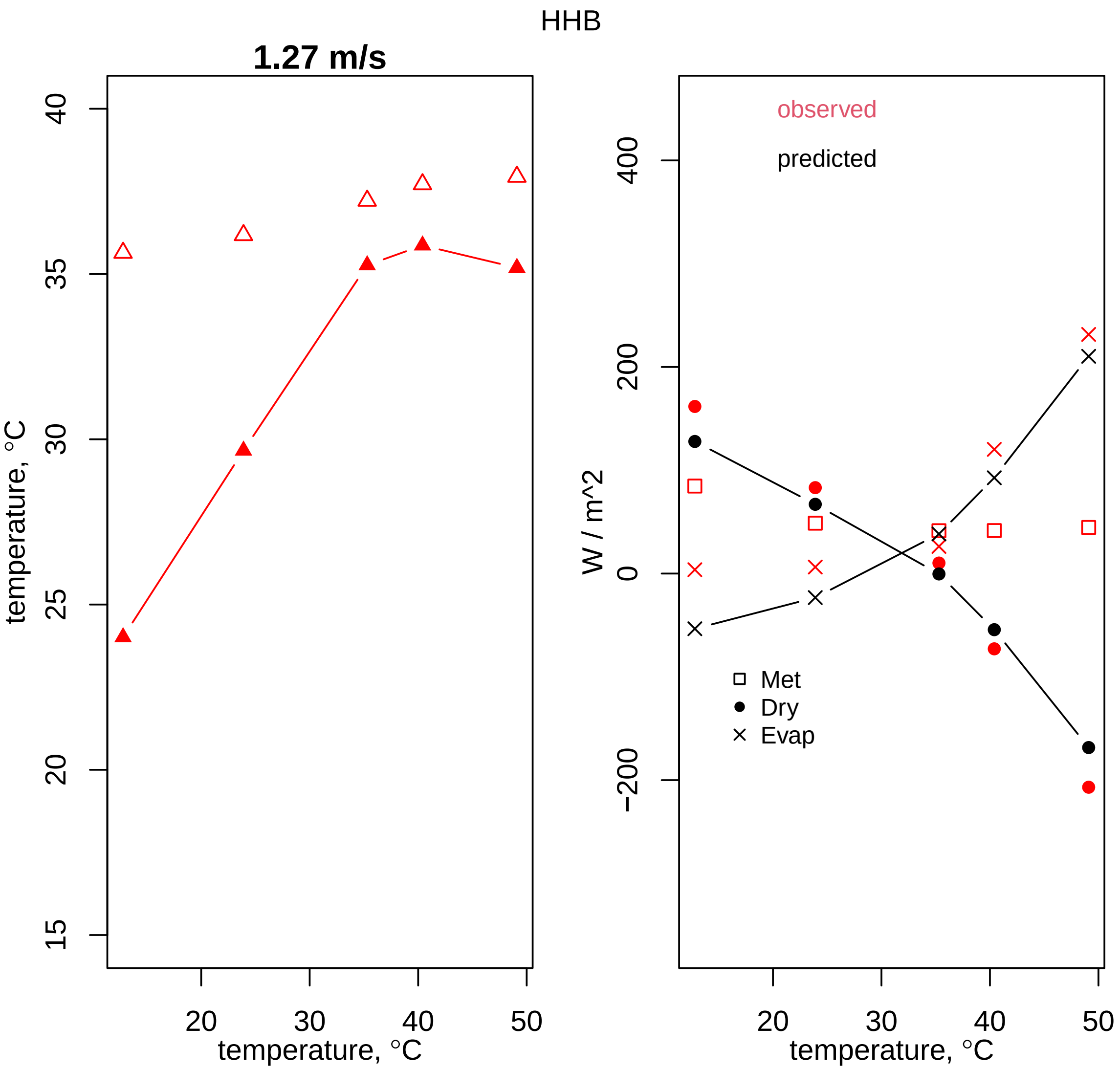

HHB
1.27 m/s
temperature, °C
15
20
25
30
35
40
20
30
40
50
temperature, °C
observed
predicted
W / m^2
−200
0
200
400
Met
Dry
Evap
20
30
40
50
temperature, °C

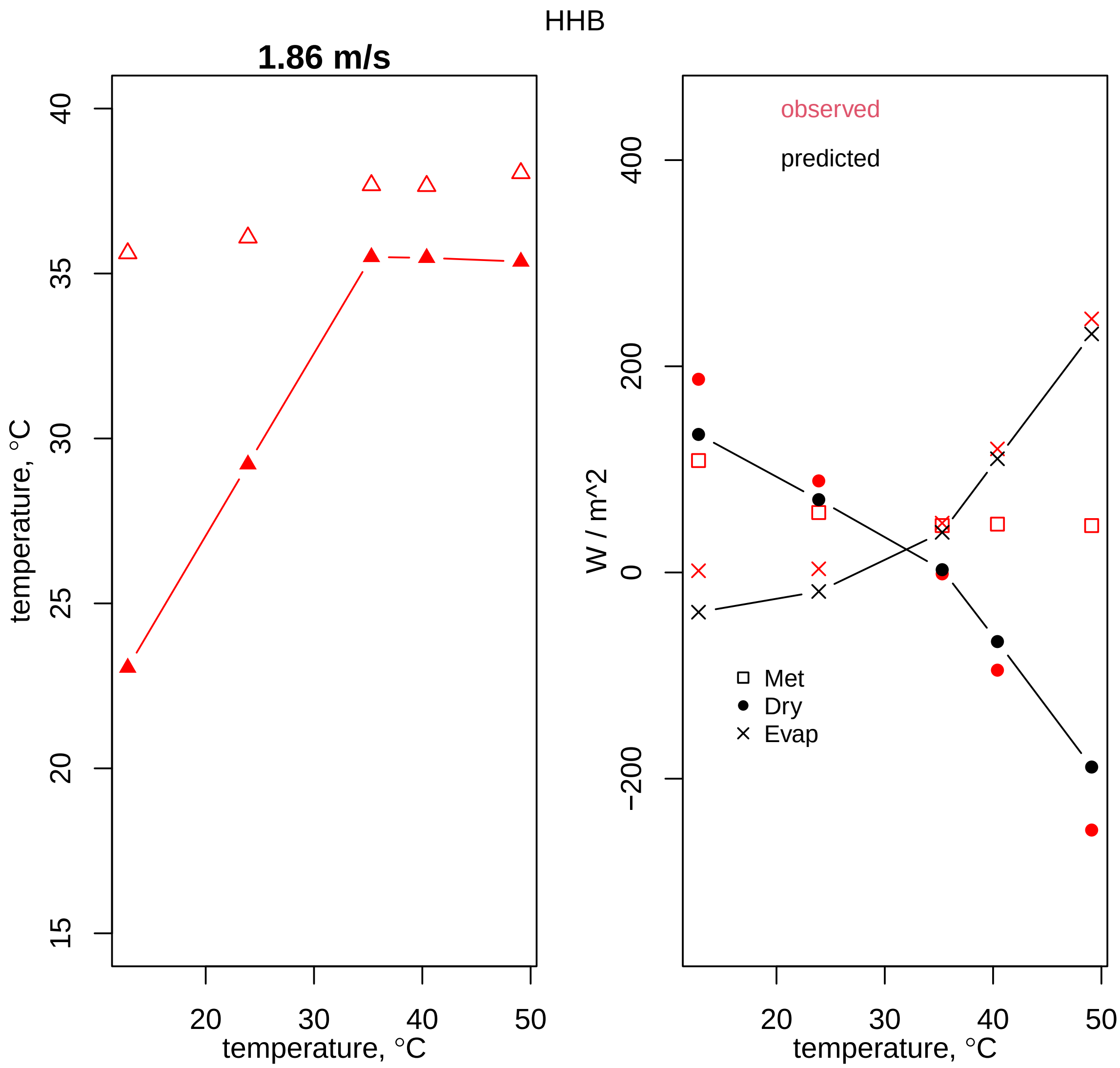
HHB
1.86 m/s
temperature, °C
15
20
25
30
35
40
20
30
40
50
temperature, °C
observed
predicted
W / m^2
−200
0
200
400
Met
Dry
Evap
20
30
40
50
temperature, °C

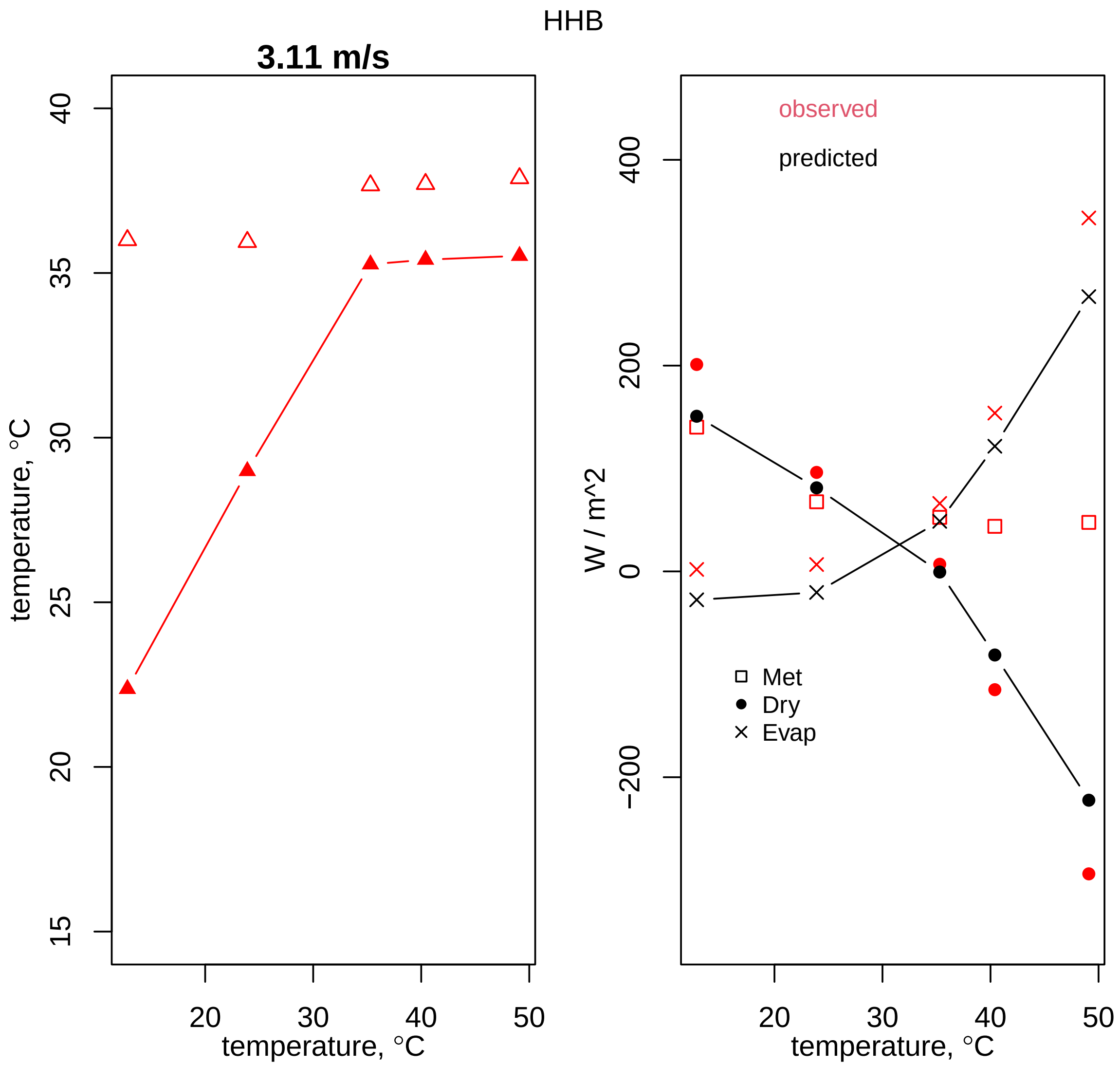
HHB
3.11 m/s
temperature, °C
15
20
25
30
35
40
20
30
40
50
temperature, °C
observed
predicted
W / m^2
−200
0
200
400
Met
Dry
Evap
20
30
40
50
temperature, °C

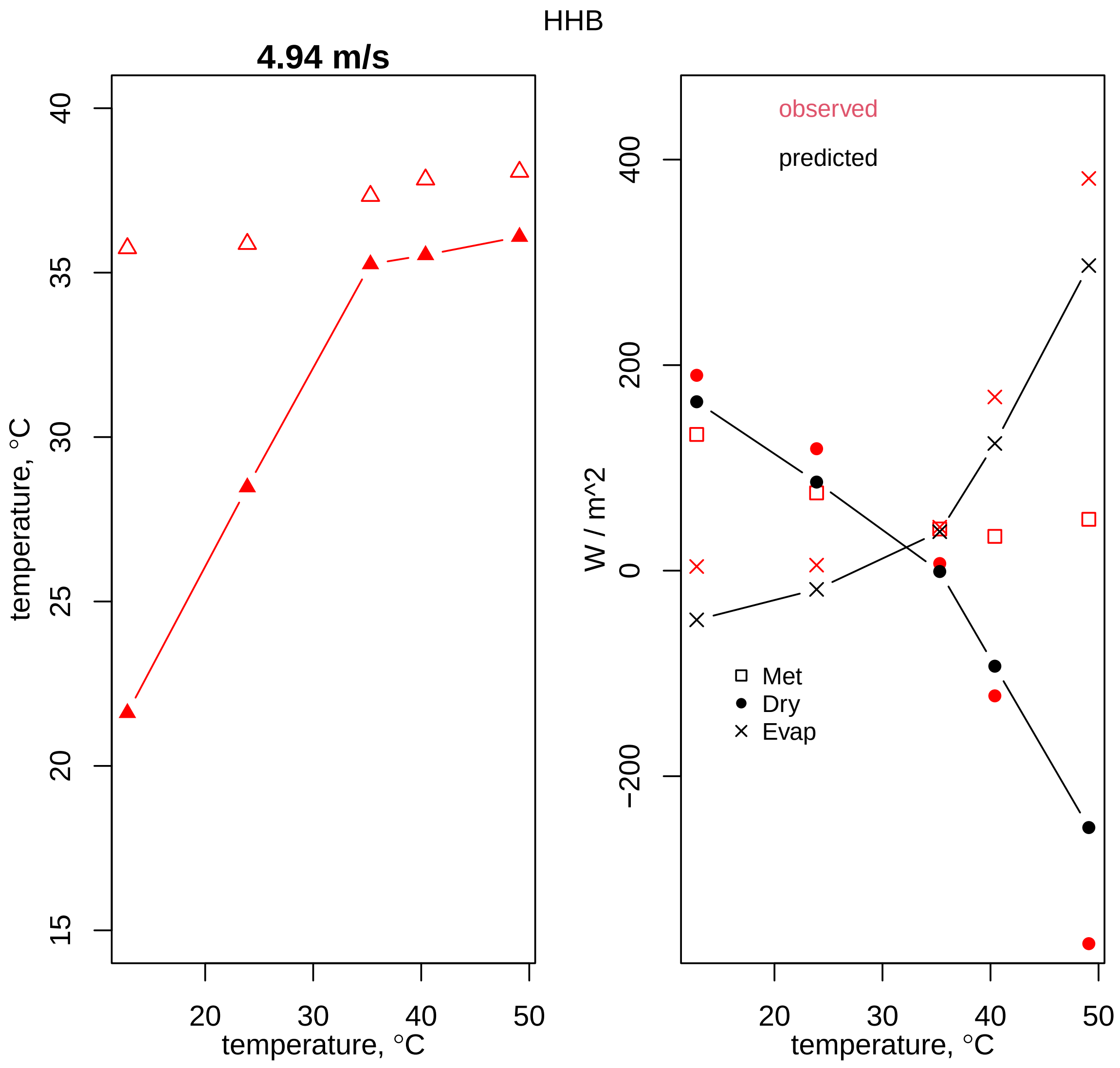

Correlations and plot

```
results <- as.data.frame(results)
colnames(results) <- c('TA', 'vel', 'Mitchell_Ts', 'Mitchell_Tr', 'Mitchell_M',
                       'Mitchell_R', 'Mitchell_C', 'Mitchell_E', 'HomoTherm_Ts',
                       'HomoTherm_Tr', 'HomoTherm_M', 'HomoTherm_R',
                       'HomoTherm_C', 'HomoTherm_E', 'manmo_Ts', 'manmo_M',
                       'manmo_R', 'manmo_C', 'manmo_E', 'HHB_E', 'HHB_CR',
                       'ISO_Ts', 'ISO_Tr', 'ISO_R', 'ISO_C', 'ISO_E')
r.HomoTherm_Ts <- cor(results$Mitchell_Ts, results$HomoTherm_Ts)
r.HomoTherm_Tr <- cor(results$Mitchell_Tr, results$HomoTherm_Tr)
r.HomoTherm_M <- cor(results$Mitchell_M, results$HomoTherm_M)
r.HomoTherm_R <- cor(results$Mitchell_R, results$HomoTherm_R)
r.HomoTherm_C <- cor(results$Mitchell_C, results$HomoTherm_C)
```

```r
r.HomoTherm_CR <- cor(results$Mitchell_C + results$Mitchell_R,
                      results$HomoTherm_C + results$HomoTherm_R)
r.HomoTherm_E <- cor(results$Mitchell_E, results$HomoTherm_E)
r.manmo_Ts <- cor(results$Mitchell_Ts, results$manmo_Ts)
r.manmo_M <- cor(results$Mitchell_M, results$manmo_M)
r.manmo_R <- cor(results$Mitchell_E, results$manmo_E)
r.manmo_C <- cor(results$Mitchell_C, results$manmo_C)
r.manmo_E <- cor(results$Mitchell_E, results$manmo_E)
r.manmo_CR <- cor(results$Mitchell_C + results$Mitchell_R,
                  results$manmo_C + results$manmo_R)
r.HHB_E <- cor(results$Mitchell_E, results$HHB_E)
r.HHB_CR <- cor(results$Mitchell_C + results$Mitchell_R, results$HHB_CR)
r.ISO_Ts <- cor(results$Mitchell_Ts, results$ISO_Ts)
r.ISO_Tr <- cor(results$Mitchell_Tr, results$ISO_Tr)
r.ISO_R <- cor(results$Mitchell_R, results$ISO_R)
r.ISO_C <- cor(results$Mitchell_C, results$ISO_C)
r.ISO_CR <- cor(results$Mitchell_C + results$Mitchell_R, results$ISO_C + results$ISO_R)
r.ISO_E <- cor(results$Mitchell_E, results$ISO_E)

get.rmsd <- function(observed, predicted){
  mean((observed - predicted) ^ 2) ^ 0.5
}

rmsd.HomoTherm_Ts <- get.rmsd(results$Mitchell_Ts, results$HomoTherm_Ts)
rmsd.HomoTherm_Tr <- get.rmsd(results$Mitchell_Tr, results$HomoTherm_Tr)
rmsd.HomoTherm_M <- get.rmsd(results$Mitchell_M, results$HomoTherm_M)
rmsd.HomoTherm_R <- get.rmsd(results$Mitchell_R, results$HomoTherm_R)
rmsd.HomoTherm_C <- get.rmsd(results$Mitchell_C, results$HomoTherm_C)
rmsd.HomoTherm_CR <- get.rmsd(results$Mitchell_C + results$Mitchell_R,
                              results$HomoTherm_C + results$HomoTherm_R)
rmsd.HomoTherm_E <- get.rmsd(results$Mitchell_E, results$HomoTherm_E)
rmsd.manmo_Ts <- get.rmsd(results$Mitchell_Ts, results$manmo_Ts)
rmsd.manmo_M <- get.rmsd(results$Mitchell_M, results$manmo_M)
rmsd.manmo_R <- get.rmsd(results$Mitchell_E, results$manmo_E)
rmsd.manmo_C <- get.rmsd(results$Mitchell_C, results$manmo_C)
rmsd.manmo_CR <- get.rmsd(results$Mitchell_C + results$Mitchell_R,
                          results$manmo_C + results$manmo_R)
rmsd.manmo_E <- get.rmsd(results$Mitchell_E, results$manmo_E)
rmsd.HHB_E <- get.rmsd(results$Mitchell_E, results$HHB_E)
rmsd.HHB_CR <- get.rmsd(results$Mitchell_C + results$Mitchell_R,
                        results$HHB_CR)
rmsd.ISO_Ts <- get.rmsd(results$Mitchell_Ts, results$ISO_Ts)
rmsd.ISO_Tr <- get.rmsd(results$Mitchell_Tr, results$ISO_Tr)
rmsd.ISO_R <- get.rmsd(results$Mitchell_R, results$ISO_R)
rmsd.ISO_C <- get.rmsd(results$Mitchell_C, results$ISO_C)
rmsd.ISO_CR <- get.rmsd(results$Mitchell_C + results$Mitchell_R, results$ISO_C + results$ISO_R)
rmsd.ISO_E <- get.rmsd(results$Mitchell_E, results$ISO_E)

par(mfrow = c(2, 2))
par(oma = c(2, 1, 2, 2) + 0.1)
par(mar = c(3, 3, 1.5, 1) + 0.1)
par(mgp = c(2, 1, 0))
plot(results$Mitchell_Ts, results$HomoTherm_Ts, pch = 16, ylim = c(15, 40),
```

```r
     xlim = c(15, 40), main = 'temperature', xlab = expression("obs, "*degree*C),
     ylab = expression("pred, "*degree*C))
points(results$Mitchell_Ts, results$manmo_Ts, col = 'grey', pch = 16)
points(results$Mitchell_Ts, results$ISO_Ts, col = 'darkgreen', pch = 16)
text(x = 19, y = 39,
     paste0('rmsd = ', sprintf("%.1f", round(rmsd.HomoTherm_Ts, 1))), cex = 0.8)
text(x = 19, y = 37,
     paste0('rmsd = ', sprintf("%.1f", round(rmsd.manmo_Ts, 1))),
     cex = 0.8, col = 'darkgrey')
text(x = 19, y = 35,
     paste0('rmsd = ', sprintf("%.1f", round(rmsd.ISO_Ts, 1))),
     cex = 0.8, col = 'darkgreen')
abline(0, 1)
legend(29, 25, c('HomoTherm', 'MANMO', 'HHB', 'ISO'), cex = 0.8, pch = 16,
       col = c('black', 'grey', 'orange', 'darkgreen'), bty = 'n')
points(results$Mitchell_Tr, results$HomoTherm_Tr, pch = 4, ylim = c(35, 38.2),
       xlim = c(35, 38.2), main = 'T_rectal',
       xlab = expression("obs, "*degree*C),
       ylab = expression("pred, "*degree*C), col = 'black')
points(results$Mitchell_Tr, results$ISO_Tr, pch = 4, ylim = c(35, 38.2),
       xlim = c(35, 38.2), main = 'T_rectal', xlab = expression("obs, "*degree*C),
       ylab = expression("pred, "*degree*C), col = 'darkgreen')
abline(0, 1)

plot(results$Mitchell_M, results$HomoTherm_M, pch = 16, ylim = c(20, 250),
     xlim = c(20, 220), main = 'metabolism', xlab = 'obs, W / m2',
     ylab = 'pred, W / m2')
#points(results$Mitchell_M, results$manmo_M, col = 'grey', pch = 16)
text(x = 50, y = 240,
     paste0('rmsd = ', sprintf("%.1f", round(rmsd.HomoTherm_M, 1))), cex = 0.8)
abline(0, 1)

plot(results$Mitchell_R + results$Mitchell_C, results$HomoTherm_R +
       results$HomoTherm_C, pch = 16, ylim = c(-400, 250),
     xlim = c(-400, 250), main = 'dry heat', xlab = 'obs, W / m2',
     ylab = 'pred, W / m2')
points(results$Mitchell_R + results$Mitchell_C, results$manmo_R +
         results$manmo_C, col = 'grey', pch = 16)
points(results$Mitchell_R  + results$Mitchell_C, results$HHB_CR,
       col = 'orange', pch = 16)
points(results$Mitchell_R  + results$Mitchell_C, results$ISO_C + results$ISO_R,
       col = 'darkgreen', pch = 16)
text(x = -300, y = 200,
     paste0('rmsd = ', sprintf("%.1f", round(rmsd.HomoTherm_CR, 1))), cex = 0.8)
text(x = -300, y = 150,
     paste0('rmsd = ', sprintf("%.1f", round(rmsd.manmo_CR, 1))), cex = 0.8,
     col = 'darkgrey')
text(x = -300, y = 100,
     paste0('rmsd = ', sprintf("%.1f", round(rmsd.ISO_CR, 1))), cex = 0.8,
     col = 'darkgreen')
text(x = -300, y = 50,
     paste0('rmsd = ', sprintf("%.1f", round(rmsd.HHB_CR, 1))), cex = 0.8,
     col = 'orange')
```

```
abline(0, 1)

plot(results$Mitchell_E, results$HomoTherm_E, pch = 16, ylim = c(-50, 450),
     xlim = c(-50, 450), main = 'evaporation', xlab = 'obs, W / m2',
     ylab = 'pred, W / m2')
points(results$Mitchell_E, results$manmo_E, col = 'grey', pch = 16)
points(results$Mitchell_E, results$HHB_E, col = 'orange', pch = 16)
points(results$Mitchell_E, results$ISO_E, col = 'darkgreen', pch = 16)
text(x = 30, y = 400,
     paste0('rmsd = ', sprintf("%.1f", round(rmsd.HomoTherm_E, 1))), cex = 0.8)
text(x = 30, y = 360,
     paste0('rmsd = ', sprintf("%.1f", round(rmsd.manmo_E, 1))), cex = 0.8,
     col = 'darkgrey')
text(x = 30, y = 320,
     paste0('rmsd = ', sprintf("%.1f", round(rmsd.HHB_E, 1))), cex = 0.8,
     col = 'orange')
text(x = 30, y = 280,
     paste0('rmsd = ', sprintf("%.1f", round(rmsd.ISO_E, 1))), cex = 0.8,
     col = 'darkgreen')
abline(0, 1)
```

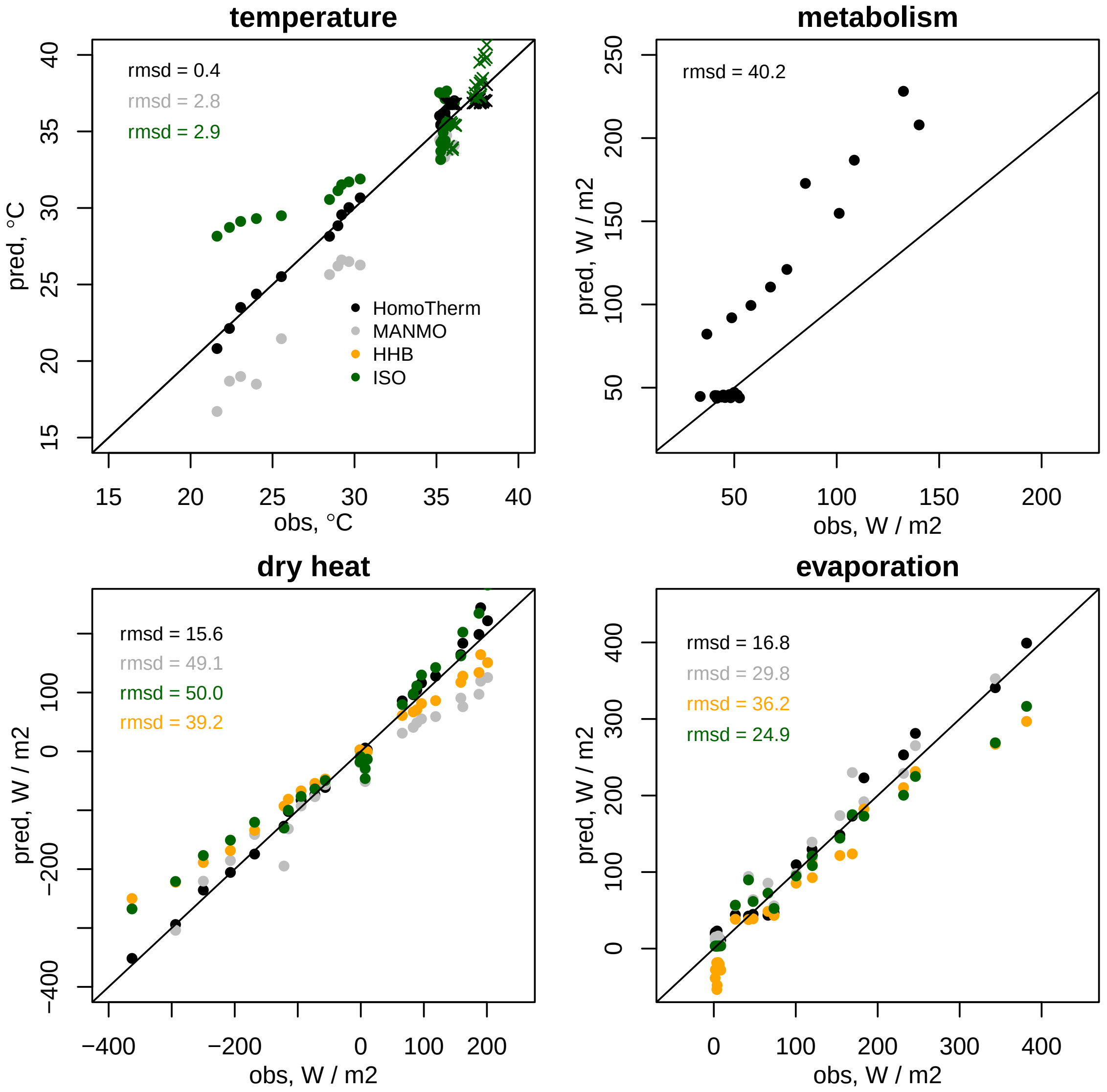

Summary statistics table.

```
correlations <- c(
  r.HomoTherm_Ts,
r.HomoTherm_Tr,
r.HomoTherm_M,
r.HomoTherm_R,
r.HomoTherm_C,
r.HomoTherm_CR,
r.HomoTherm_E,
r.manmo_Ts,
NA,
NA,
r.manmo_R,
```

```
r.manmo_C,
r.manmo_CR,
r.manmo_E,
r.ISO_Ts, r.ISO_Tr, NA, r.ISO_R, r.ISO_C, r.ISO_CR, r.ISO_E,
NA, NA, NA, NA, NA, r.HHB_CR, r.HHB_E)

rmsds <- c(
  rmsd.HomoTherm_Ts,
  rmsd.HomoTherm_Tr,
  rmsd.HomoTherm_M,
  rmsd.HomoTherm_R,
  rmsd.HomoTherm_C,
  rmsd.HomoTherm_CR,
  rmsd.HomoTherm_E,
  rmsd.manmo_Ts,
  NA,
  NA,
  rmsd.manmo_R,
  rmsd.manmo_C,
  rmsd.manmo_CR,
  rmsd.manmo_E,
  rmsd.ISO_Ts, rmsd.ISO_Tr, NA, rmsd.ISO_R, rmsd.ISO_C, rmsd.ISO_CR, rmsd.ISO_E,
  NA, NA, NA, NA, NA, rmsd.HHB_CR, rmsd.HHB_E)

summary.r <- matrix(data = round(correlations, 3), nrow = 7, ncol = 4)
summary.r <- data.frame(summary.r, row.names = c('T_skin', 'T_rectal', 'metabolism', 'radiation', 'conve
colnames(summary.r) <- c('Homotherm', 'MANMO', 'PHS', 'HHB')

summary.rmsd <- matrix(data = round(rmsds, 2), nrow = 7, ncol = 4)
summary.rmsd <- data.frame(summary.rmsd, row.names = c('T_skin', 'T_rectal', 'metabolism', 'radiation',
colnames(summary.rmsd) <- c('Homotherm', 'MANMO', 'PHS', 'HHB')

knitr::kable(summary.r)
```

	Homotherm	MANMO	PHS	HHB
T_skin	0.997	0.996	0.919	NA
T_rectal	0.502	NA	0.943	NA
metabolism	0.947	NA	NA	NA
radiation	0.988	0.984	0.988	NA
convection	0.989	0.979	0.943	NA
dry heat flux	0.997	0.978	0.963	0.997
evaporation	0.992	0.984	0.985	0.981

```
knitr::kable(summary.rmsd)
```

	Homotherm	MANMO	PHS	HHB
T_skin	0.44	2.81	2.93	NA
T_rectal	0.80	NA	1.31	NA
metabolism	40.16	NA	NA	NA
radiation	24.74	29.85	25.47	NA

	Homotherm	MANMO	PHS	HHB
convection	34.52	38.43	49.66	NA
dry heat flux	15.60	49.14	49.96	39.25
evaporation	16.83	29.85	24.92	36.18

References

Malchaire, J., A. Piette, B. Kampmann, P. Mehnert, H. Gebhardt, G. Havenith, E. den Hartog, I. Holmer, K. Parsons, G. Alfano, and B. Griefahn. 2001. Development and validation of the predicted heat strain model. The Annals of Occupational Hygiene 45:123–135.

Mitchell, D., C. H. Wyndham, A. R. Atkins, A. J. Vermeulen, H. S. Hofmeyr, N. B. Strydom, and T. Hodgson. 1968. Direct measurement of the thermal responses of nude resting men in dry environments. Pflugers Archiv European Journal of Physiology 303:324–343.

Myrup, L. O., and D. L. Morgan. 1972. Numerical model of the urban atmosphere. Volume I The city-surface interface. University of California, Davis.

Vanos, J., G. Guzman-Echavarria, J. W. Baldwin, C. Bongers, K. L. Ebi, and O. Jay. 2023. A physiological approach for assessing human survivability and liveability to heat in a changing climate. Nature Communications 14:7653.
