# Supplementary material for: HomoTherm: An Open‐Source Approach to Modelling Heat Exchange in Humans and Other Hominins in Diverse Environments
Source: Glob Chang Biol. 2026 Apr 1;32(4):e70830. doi: 10.1111/gcb.70830 (PMC13044332; doi:10.1111/gcb.70830)
Supplement: Supplementary file 5 — Appendix S5: gcb70830‐sup‐0005‐Appendix 5.pdf. [file GCB-32-e70830-s011.pdf]

Test of HomoTherm and MANMO models against Shkolnik et al. 1980

Michael Kearney

2026-01-11

Overview

This document illustrates an outdoor simulation with the HomoTherm model, comparing it to the MANMO model, using the micro_terra microclimate model of NicheMapR to generate the outdoor microclimatic conditions. In particular, it tests the findings against Shkolnik et al.'s (1980) famous study of Bedouin robes.

Load the libraries and data

```
library(NicheMapR)
library(readxl)
localpath <- 'c:/Users/mrke/Dropbox/Current Research Projects/mammal_projects/manmo analysis/'
source(paste0(localpath, 'code/MANMO/MANMO_R.R')) # the MANMO function
source(paste0(localpath, 'code/MANMO/run.MANMO.R'))
source(paste0(localpath, 'code/PHS/calcIso7933_Tcl.R'))
```

Load the Shkolnik et al. observations

```
# read in observations from Shkolnik et al. 1980
obs <- read.csv(paste0(localpath, 'data/Shkolnik/bedouin_robes.csv'))
```

Environmental conditions

Simulate microclimate at Hatzeva resarch station where study was undertaken. Choosing 1978 but doesn't make much difference if 1977 or 1979. The specific year isn't stated in the original paper.

```
loc <-  c(35.238676, 30.775372)
#micro <- micro_terra(loc = loc,
#                     elevatr = 1,
#                     microclima = 1,
#                     ystart = 1978,
#                     yfinish = 1978,
#                     Usrhyt = 0.8, REFL = 0.4)
#save(micro, file = 'output/micro_Hatzeva.Rda')
load(paste0(localpath, 'output/micro_Hatzeva.Rda'))
# extract microclimate tables
```

```
# above ground microclimatic conditions, min shade
metout <- as.data.frame(micro$metout)
# above ground microclimatic conditions, max shade
shadmet <- as.data.frame(micro$shadmet)
soil <- as.data.frame(micro$soil) # soil temperatures, minimum shade
shadsoil <- as.data.frame(micro$shadsoil) # soil temperatures, maximum shade

# choose month to calculate heat stress for
month <- 6 # month was not stated but assuming June
range <- (month * 24 - 23):(month * 24)
T_chamber <- 48 # temperature in metabolic chamber study, deg C
```

Code to set and plot the resulting conditions. Shkolnik additionally reported results conditions in a metabolic chamber under blackbody conditions with a radiant temperature of 48 deg C, 15% relative humidity and low wind speed, so the 'set_environment' function can either set outdoor conditions for a particular month or make it uniform chamber conditions.

```
# plotting above-ground conditions in minimum shade for month of interest
set_environment <- function(month = month,
                            micro = micro,
                            T_chamber = T_chamber,
                            outdoors = FALSE,
                            plotenv = TRUE){

  # extract microclimate tables
  # above ground microclimatic conditions, min shade
  metout <- as.data.frame(micro$metout)
  # above ground microclimatic conditions, max shade
  shadmet <- as.data.frame(micro$shadmet)
  soil <- as.data.frame(micro$soil) # soil temperatures, minimum shade
  shadsoil <- as.data.frame(micro$shadsoil) # soil temperatures, maximum shade

  # choose range
  range <- (month * 24 - 23):(month * 24)
  minshade <- micro$minshade[1]

  # define environment
  BPs <- rep(101325, length(range))
  Zs <- metout$ZEN[range]
  PDIFs <- micro$diffuse_frac[range]

  if(outdoors){
    CONV_ENHANCE <- 1
    TAs <- metout$TALOC[range]
    RHs <- metout$RHLOC[range]
    VELs <- metout$VLOC[range]
    VREFs <- metout$VREF[range]
    RHREFs <- metout$RH[range]
    QSOLRs <- metout$SOLR[range]
    TSKYs <- (metout$TSKYC[range] + soil$D0cm[range]) / 2
    TGRDs <- (metout$TSKYC[range] + soil$D0cm[range]) / 2
    TBUSHs <- metout$TALOC[range]
    TAREFs <- metout$TAREF[range]
```

```
  SHADEs <- rep(0, length(range))
}else{
  CONV_ENHANCE <- 1
  TAs <- metout$TALOC[range] * 0 + T_chamber
  RHs <- metout$RHLOC[range] * 0 + 15
  RHREFs <- metout$RH[range] * 0 + 15
  VELs <- metout$VLOC[range] * 0 + 0.1
  VREFs <- metout$VREF[range] * 0 + 0.1
  QSOLRs <- metout$SOLR[range] * 0
  TSKYs <- metout$TSKYC[range] * 0 + T_chamber
  TGRDs <- soil$D0cm[range] * 0 + T_chamber
  TBUSHs <- metout$TALOC[range] * 0 + T_chamber
  TAREFs <- metout$TAREF[range] * 0 + T_chamber
}
dates <- micro$dates[range]

  if(plotenv){
  par(mfrow = c(4, 2))
  par(oma = c(2, 1, 2, 2) + 0.1)
  par(mar = c(3, 3, 1.5, 1) + 0.1)
  par(mgp = c(2, 1, 0))
  with(metout, {plot(TAs ~ dates, xlab = "Date and Time",
                     ylab = "Air Temperature (°C)",
                     type = "l", main = paste("air temperature, ",
                                              minshade, "% shade",sep = ""))})
  legend(month - 1, 38, legend = c('0.8 m', '2 m'), lty = c(1, 2),
         col = c(1, 'blue'), bty = 'n')
  with(metout, {points(TAREFs ~ dates, xlab = "Date and Time", ylab =
                         "Air Temperature (°C)", type = "l",lty = 2,
                       col = 'blue')})
  with(metout, {plot(RHs ~ dates, xlab = "Date and Time",
                     ylab = "Relative Humidity (%)", type = "l",
                     ylim = c(0, 100),
                     main = paste("humidity, ",minshade, "% shade",sep=""))})
  with(metout, {points(RHREFs ~ dates, xlab = "Date and Time",
                       ylab = "Relative Humidity (%)", type = "l",
                       col = 'blue',lty = 2, ylim = c(0, 100))})
  with(metout, {plot(TSKYs ~ dates, xlab = "Date and Time",
                     ylab = "Sky Temperature (°C)", type = "l",
                     main = paste("sky temperature, ",
                                  minshade, "% shade", sep=""))})
  with(metout, {plot(VREFs ~ dates, xlab = "Date and Time",
                     ylab = "Wind Speed (m/s)", type = "l",
                     main = "wind speed", col = 'blue',ylim = c(0, 15))})
  with(metout, {points(VELs ~ dates, xlab = "Date and Time",
                       ylab = "Wind Speed (m/s)", type = "l", lty = 2)})
  with(metout, {plot(Zs ~ dates,xlab = "Date and Time", ylab =
                       "Zenith Angle of Sun (deg)", type = "l",
                     main = "solar angle")})
  with(metout, {plot(QSOLRs ~ dates, xlab = "Date and Time",
                     ylab = "Solar Radiation (W/m^2)", type = "l",
                     main = "solar radiation")})
  with(soil, {plot(TGRDs ~ dates, xlab = "Date and Time",
```

```
                     ylab = "Soil Temperature (°C)", type = "l",
                     main = "soil surface temperature", ylim = c(10, 70))})
  }

  return(list(TAs = TAs, TAREFs = TAREFs, RHs = RHs, RHREFs = RHREFs,
              VELs = VELs, VREFs = VREFs, TSKYs = TSKYs, TGRDs = TGRDs,
              TBUSHs = TBUSHs, QSOLRs = QSOLRs, Zs = Zs, PDIFs = PDIFs,
              CONV_ENHANCE = CONV_ENHANCE, dates = dates))
}
```

Plot the resulting outdoor microclimate.

```
env.out <- set_environment(month = month, micro = micro,
                        T_chamber = T_chamber, outdoors = TRUE, plotenv = TRUE)
env.out.chamber <- set_environment(month = month, micro = micro, T_chamber =
                                   T_chamber, outdoors = FALSE, plotenv = FALSE)
dates <- env.out$dates
REFHYT <- 2 # m, reference height (met station)
RUF <- 0.004 # m, roughness height
```

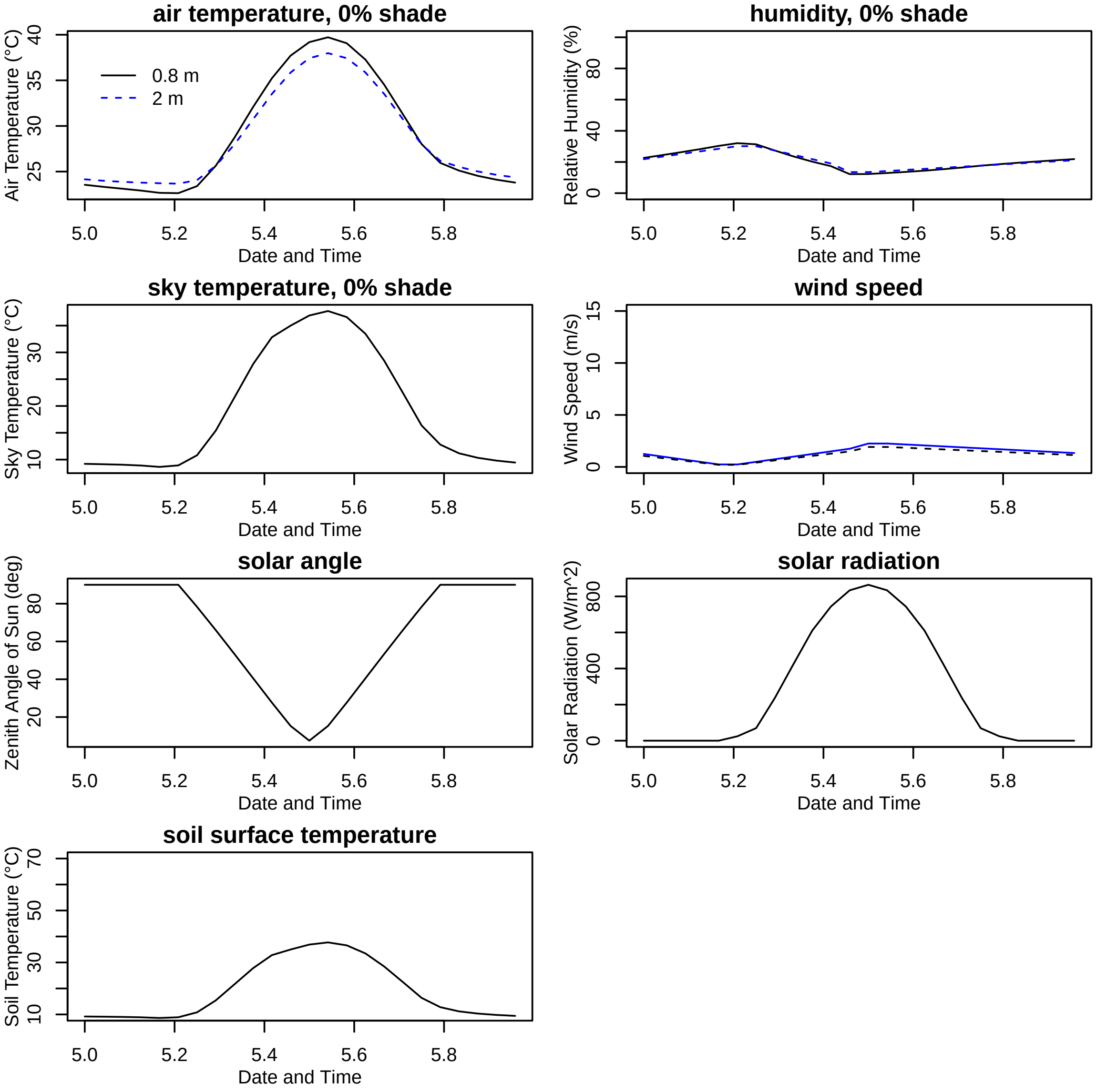

Nude

First a nude person is simulated. No observtions were made for a nude person, so this is just for interest.

Set parameters.

```
# person parameters
MASS <- 63 # MASS, kg
HEIGHT <- 172 # height, cm
AREA <- 0.00718 * MASS ^ 0.425 * HEIGHT ^ 0.725 # DuBois area, m2
QMETAB_REST <- mean(obs$h_metab) * AREA # mean observed metabolic rate, W
G_m.G2 <- QMETAB_REST / AREA
CLO.mode <- 2 # MANMO CLO.mode = 2 means clothing temp equals skin temp
MASSFRACs <- c(0.07609801, 0.50069348, 0.04932963, 0.16227462)
```

```r
MASSs <- MASS * MASSFRACs
INSDEPDs <- c(0.01, 1e-9, 1e-9, 1e-9) # fur depth, dorsal (m)
INSDEPVs <- c(1e-9, 1e-9, 1e-9, 1e-9) # fur depth, ventral (m)
# albedo (mean value in Gates for Caucasian)
REFLDs <- rep(0.3, 4) # skin dorsal reflectivity dec %,
REFLVs <- REFLDs # skin ventral reflectivity dec %
PCTBAREVAPs <- c(60, 90, 90, 90)
SHAPE_Bs <- c(1.6, 1.9, 11, 8.0)
shapes <- GET_SHAPES(MASSs = MASSs,
                     AREA = AREA,
                     SHAPE_Bs = SHAPE_Bs,
                     SHAPE_Bs.min = SHAPE_Bs,
                     SHAPE_Bs.max = SHAPE_Bs)
SHAPE_Bs <- shapes$SHAPE_Bs
PJOINs <- shapes$PJOINs
HEIGHT_out <- shapes$HEIGHT_out
AREA_out <- shapes$AREA_out
#rbind(AREA, AREA_out, HEIGHT/100, HEIGHT_out)

par(mfrow = c(1, 1))
plot_human(MASS = MASS,
           HEIGHT = HEIGHT,
           INSDEPDs = INSDEPDs,
           INSDEPVs = INSDEPVs,
           SHAPE_Bs = SHAPE_Bs)
```

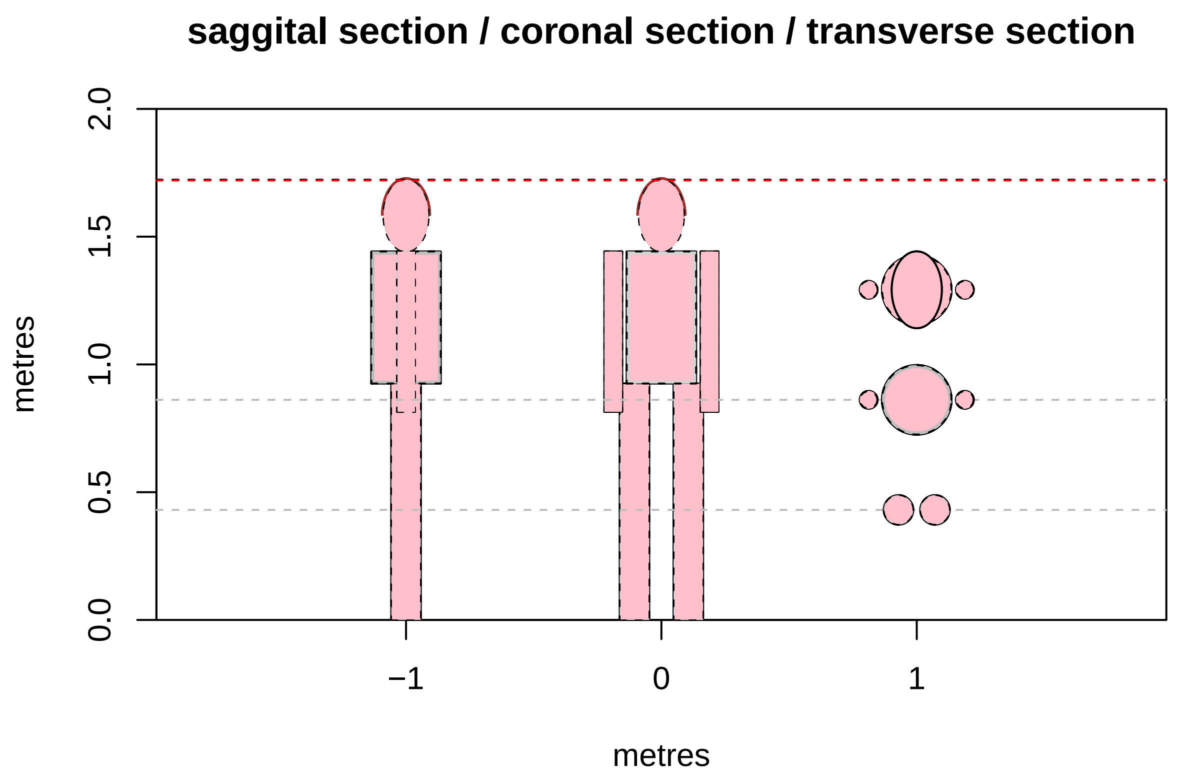

```
## [1] 1.722834
```

```r
heights <- get_heights(MASSs = MASSs, # kg, masses per part
            SHAPE_Bs = SHAPE_Bs)
duration <- 4 # hrs duration for Iso7933
```

Run simulations.

```r
# run HomoTherm simulation
HomoTherm.out_nude <- HomoTherm_var(MASS = MASS,
                                    QMETAB_REST = QMETAB_REST,
                                    INSDEPDs = INSDEPDs,
                                    INSDEPVs = INSDEPVs,
                                    SHAPE_Bs = SHAPE_Bs,
                                    PJOINs = PJOINs,
                                    PCTBAREVAPs = PCTBAREVAPs,
                                    REFLDs = REFLDs,
                                    REFLVs = REFLVs,
                                    heights = heights,
                                    REFHYT = REFHYT,
                                    RUF = RUF,
                                    TAs = env.out$TAs,
                                    TAREFs = env.out$TAREFs,
                                    TSKYs = env.out$TSKYs,
                                    TGRDs = env.out$TGRDs,
                                    RHs = env.out$RHs,
                                    RHREFs = env.out$RHREFs,
                                    VELs = env.out$VELs,
                                    VREFs = env.out$VREFs,
                                    QSOLRs = env.out$QSOLRs,
                                    Zs = env.out$Zs,
                                    PDIFs = env.out$PDIFs,
                                    ELEV = micro$elev,
                                    ABSSB = 1 - micro$REFL,
                                    CONV_ENHANCE = env.out$CONV_ENHANCE)

# run MANMO simulation
clo <- colMeans(get_clo(HomoTherm.out_nude,
                        INSDEPDs = INSDEPDs,
                        INSDEPVs = INSDEPVs))
G_m.G2s <- HomoTherm.out_nude$balance$QMETAB / AREA # W/m^2
#G_m.G2s <- rep(G_m.G2, length(env.outTAs))
Q_hs <- env.out$QSOLRs / AREA # global solar radiation, W/m^2
q_hs <- env.out$QSOLRs * env.out$PDIFs / AREA # diffuse solar radiation, W/m^2
MANMO.out_nude <- run.MANMO(W = rep(1 / 100, length(env.out$TAs)),
                          Ht.H4 = HEIGHT,
                          Wt.W4 = MASS,
                          D3 = c(mean(INSDEPDs[2:4]), rep(1e-10, 3)),
                          Maximum.SR = 1000 / 60 / AREA,
                          G_m.G2s = G_m.G2s,
                          CLO.C4 = clo,
                          CLO.mode = CLO.mode,
                          a_skn.B4 = REFLDs[2],
```

```
                          a_clo.B5 = REFLDs[2],
                          TAs = env.out$TAs,
                          TSKYs = env.out$TSKYs,
                          TGNDs = env.out$TGRDs,
                          RH.H2s = env.out$RHs / 100,
                          Q_hs = Q_hs,
                          q_hs = q_hs,
                          Zs = env.out$Zs,
                          VELs = env.out$VELs,
                          a.B3 = micro$REFL)

# Iso7933
Iso7933.out <- lapply(1:length(env.out$TAs),
                           function(x){
                             calcIso7933_Tcl(accl = 100,
                                             Duration = 60 * duration,
                                             posture = 2,
                                             Ta = env.out$TAs[x],
                                             Pa = WETAIR(db = env.out$TAs[x],
                                                  rh = env.out$RHs[x])$e / 1000,
                                             Tr = (env.out$TGRDs[x]
                                                   + env.out$TSKYs[x]) / 2 +
                                               (env.out$QSOLR[x] / 1366) * 30,
                                             Va = env.out$VELs[x],
                                             Tsk = env.out$TAs[x],
                                             Met = QMETAB_REST / AREA,
                                             Icl = clo,
                                             weight = MASS,
                                             height = HEIGHT / 100,
                                             Adu = AREA,
                                             Tre = 36.8,
                                             Tcr = 36.8,
                                             SWp = 0.5
                             )})
Iso7933.out_nude <- as.data.frame(do.call(rbind, Iso7933.out))
```

Plot results.

```
plot.output <- function(HomoTherm.out, MANMO.out, Iso7933.out, env.out){
  par(mfrow = c(3, 2))
  par(oma = c(2, 1, 1, 1) + 0.1) # margin spacing
  par(mar = c(4, 4, 1, 1) + 0.1) # margin spacing
  par(mgp = c(2, 1, 0) ) # margin spacing
  balance <- HomoTherm.out$balance
  plot(dates, balance$T_CLO, type = 'l', ylim = c(-10, 60),
       main = 'clothing temperature', ylab = 'temperature, deg C',
       xlab = 'month')
  points(dates, MANMO.out$T_clo.T9, type = 'l', col = '2')
  points(dates, Iso7933.out$Tcl, type = 'l', col = 'darkgreen')
  points(dates, env.out$TAs, type = 'l', col = 'blue', lty = 2)
  abline(h = 36.5, col = 'orange', lty = 2)
  legend(dates[1], 65, legend = c('HomoTherm', 'MANMO', 'PHS'), lty = 1,
         col = c(1, 2, 'darkgreen'), bty = 'n')
```

```
  legend(dates[16], 65, legend = 'T_air', lty = 2, col = 'blue', bty = 'n')
  plot(balance$T_CLO, MANMO.out$T_clo.T9, type = 'p', pch = 16,
       ylim = c(-10, 60),  xlim = c(-10, 60), xlab = 'HomoTherm',
       ylab = 'MANMO/PHS', main = 'clothing temperature correlation')
  points(balance$T_CLO, Iso7933.out$Tcl, type = 'p', pch = 16,
         col = 'darkgreen')
  abline(0, 1)
  abline(h = 36.5, col = 'orange', lty = 2)
  abline(v = 36.5, col = 'orange', lty = 2)
  plot(dates, balance$T_SKIN, type = 'l', ylim = c(-10, 60),
       main = 'skin temperature', ylab = 'temperature, deg C', xlab = 'month')
  points(dates, MANMO.out$Tskin, type = 'l', col = '2')
  points(dates, Iso7933.out$Tskeq, type = 'l', col = 'darkgreen')
  points(dates, env.out$TAs, type = 'l', col = 'blue', lty = 2)
  abline(h = 36.5, col = 'orange', lty = 2)
  plot(balance$T_SKIN, MANMO.out$Tskin, type = 'p', pch = 16,
       ylim = c(-10, 60),  xlim = c(-10, 60), xlab = 'HomoTherm',
       ylab = 'MANMO/PHS', main = 'skin temperature correlation')
  points(balance$T_SKIN, Iso7933.out$Tskeq, type = 'p', pch = 16,
         col = 'darkgreen')
  abline(0, 1)
  abline(h = 36.5, col = 'orange', lty = 2)
  abline(v = 36.5, col = 'orange', lty = 2)
  plot(dates, balance$EVAP_CUT_L + balance$EVAP_RESP_L, type = 'l',
       ylim = c(0, 1.5), main = 'evap rate', ylab = 'L per hour', xlab = 'month')
  points(dates, MANMO.out$evap.L.h, type = 'l', col = 2)
  #points(dates, (Iso7933.out$Ep + Iso7933.out$Eres) * AREA / (2.5012e6-2.3787e3*Iso7933.out$Tsk) * 360
    points(dates, Iso7933.out$SWtotg / duration / 1000, type = 'l',
           col = 'darkgreen')
  plot(balance$EVAP_CUT_L + balance$EVAP_RESP_L, MANMO.out$evap.L.h,
       type = 'p', pch = 16, ylim = c(0, 1.5), xlim = c(0, 1.5),
       xlab = 'HomoTherm', ylab = 'MANMO/PHS', main = 'evap rate correlation')
    points(balance$EVAP_CUT_L + balance$EVAP_RESP_L, Iso7933.out$SWtotg
           / duration / 1000, type = 'p', pch = 16, col = 'darkgreen')

  abline(0, 1)
}
plot.balance <- function(HomoTherm.out, MANMO.out){
  balance <- HomoTherm.out$balance
  par(mfrow = c(1, 1))
  plot(balance$QSLR, col = 'red', type = 'l', ylim = c(-700, 700),
       main = 'heat fluxes', ylab = 'power (W)')
  points(MANMO.out$R_m.R, col = 'red', type = 'l', lty = 2)
  points(balance$QRAD_IN - balance$QRAD_OUT, col = '1', type = 'l', lty = 2)
  points(MANMO.out$I_m.I, col = '1', type = 'l')
  points(balance$QCONV, col = 'grey', type = 'l')
  points(MANMO.out$H_m.H, col = 'grey', type = 'l', lty = 2)
  points(balance$QEVAP_CUT + balance$QEVAP_RESP, col = 'blue', type = 'l')
  points(MANMO.out$E_m.E, col = 'blue', type = 'l', lty = 2)
  points(balance$QMETAB, col = 'brown', type = 'l')
  points(MANMO.out$M_m.M, col = 'brown', type = 'l', lty = 2)
  abline(h = QMETAB_REST, col = 'brown', lwd = 2)
  legend(0, 700, bty = 'n',
```

```
        legend = c('solar', 'net rad', 'conv', 'evap', 'metab'),
        col = c(2, 1, 'grey', 'blue', 'brown'), lty = 1)
  legend(15, 700, bty = 'n', legend = c('HomoTherm', 'MANMO'),
        col = 1, lty = c(1, 2))
}
plot.output(HomoTherm.out_nude, MANMO.out_nude, Iso7933.out_nude, env.out)
```

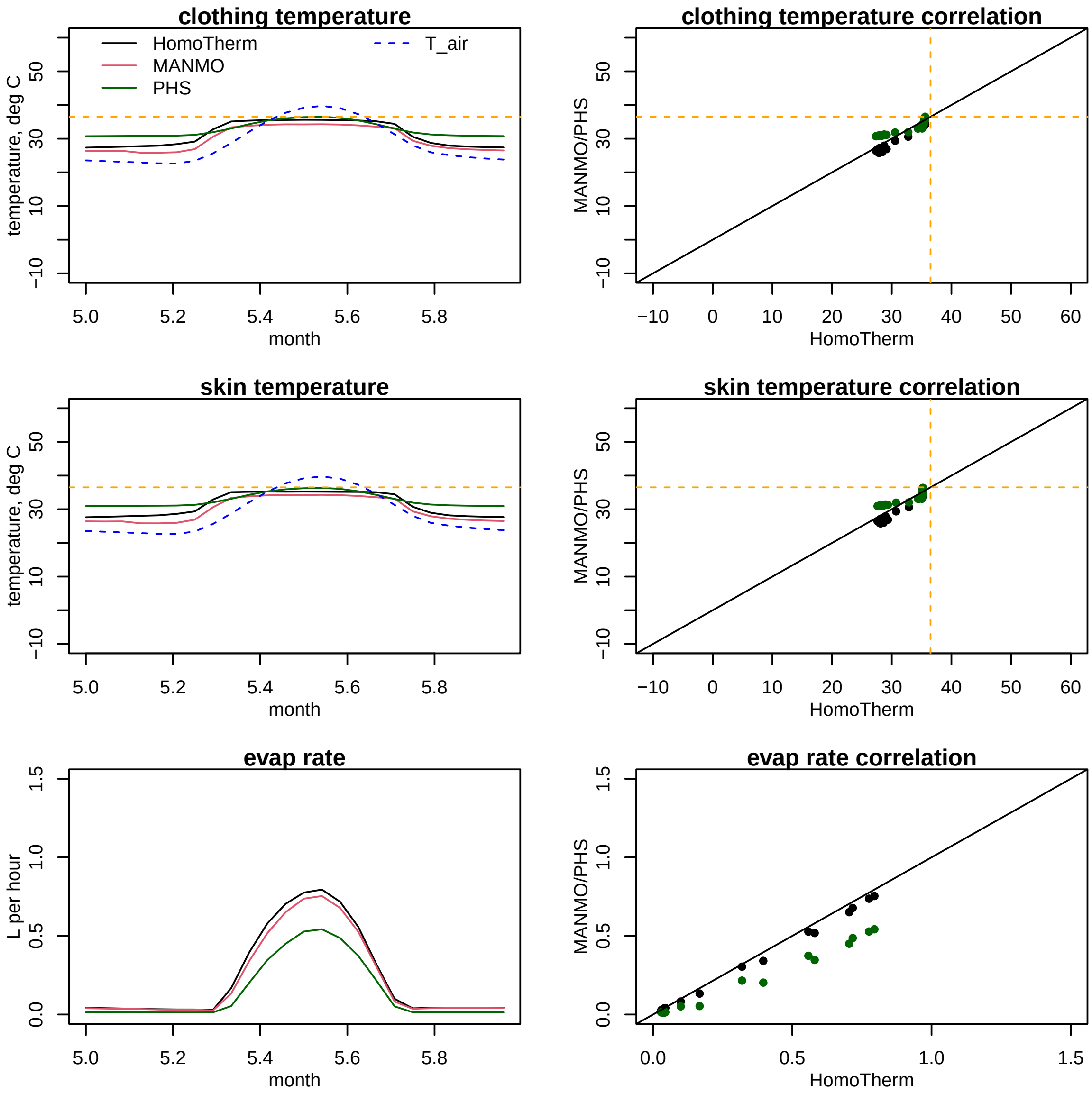


```
plot.balance(HomoTherm.out_nude, MANMO.out_nude)
```

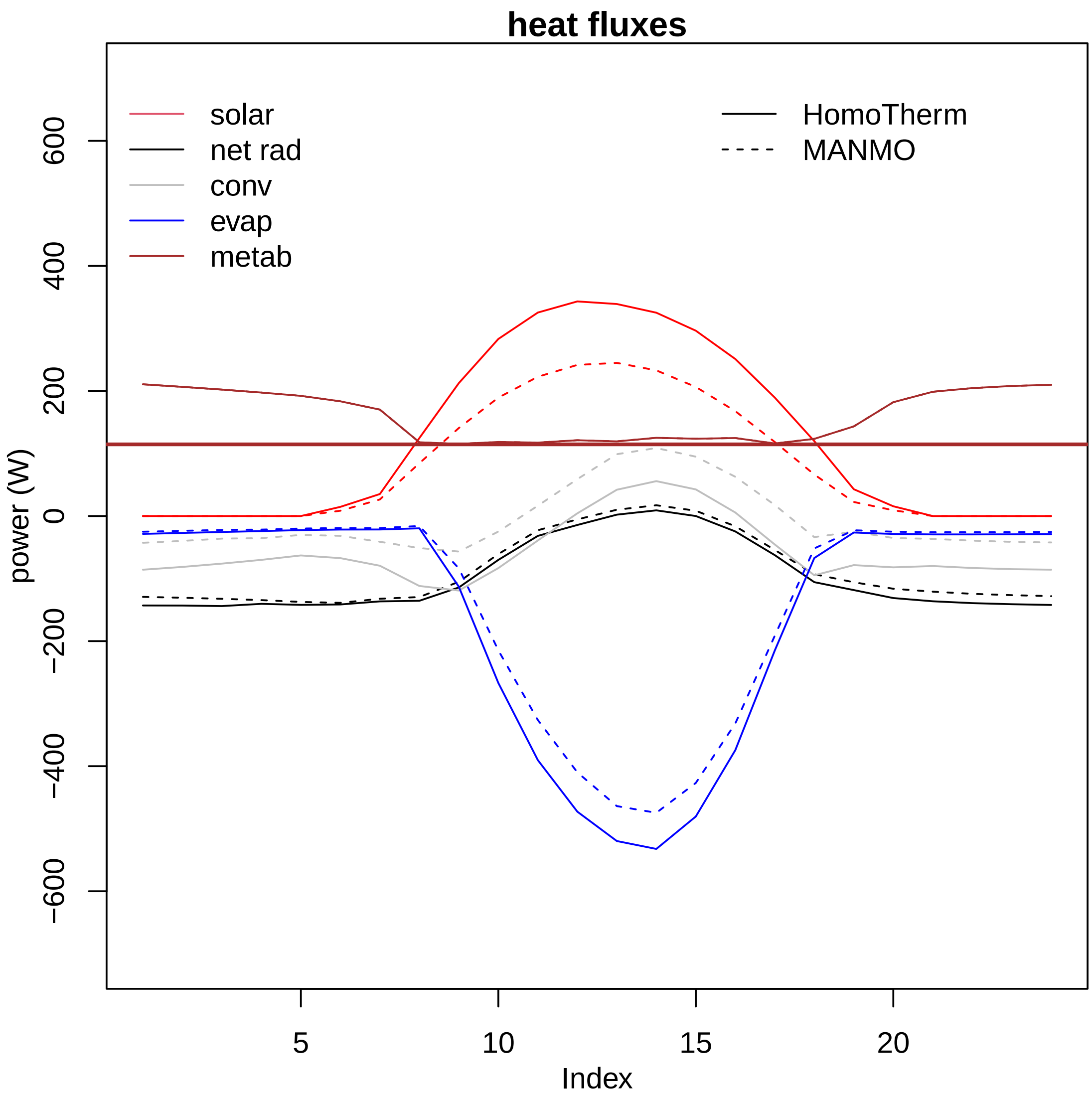

Light-coloured Clothing

Now contrast this with light-coloured clothing 3 mm deep. Again no observations were made for this scenario.

Set parameters.

```
PCTBAREVAPs <- c(60, 10, 10, 10)
INSDEPDs <- rep(0.003, 4) # fur depth, dorsal (m)
INSDEPVs <- c(1e-9, 0.003, 0.003, 0.003) # fur depth, ventral (m)
CLO.mode <- 0 # empirical function for MANMO clothing temperature
par(mfrow = c(1, 1))
plot_human(MASS = MASS,
           HEIGHT = HEIGHT,
```

```
          INSDEPDs = INSDEPDs,
          INSDEPVs = INSDEPVs,
          SHAPE_Bs = SHAPE_Bs)
```

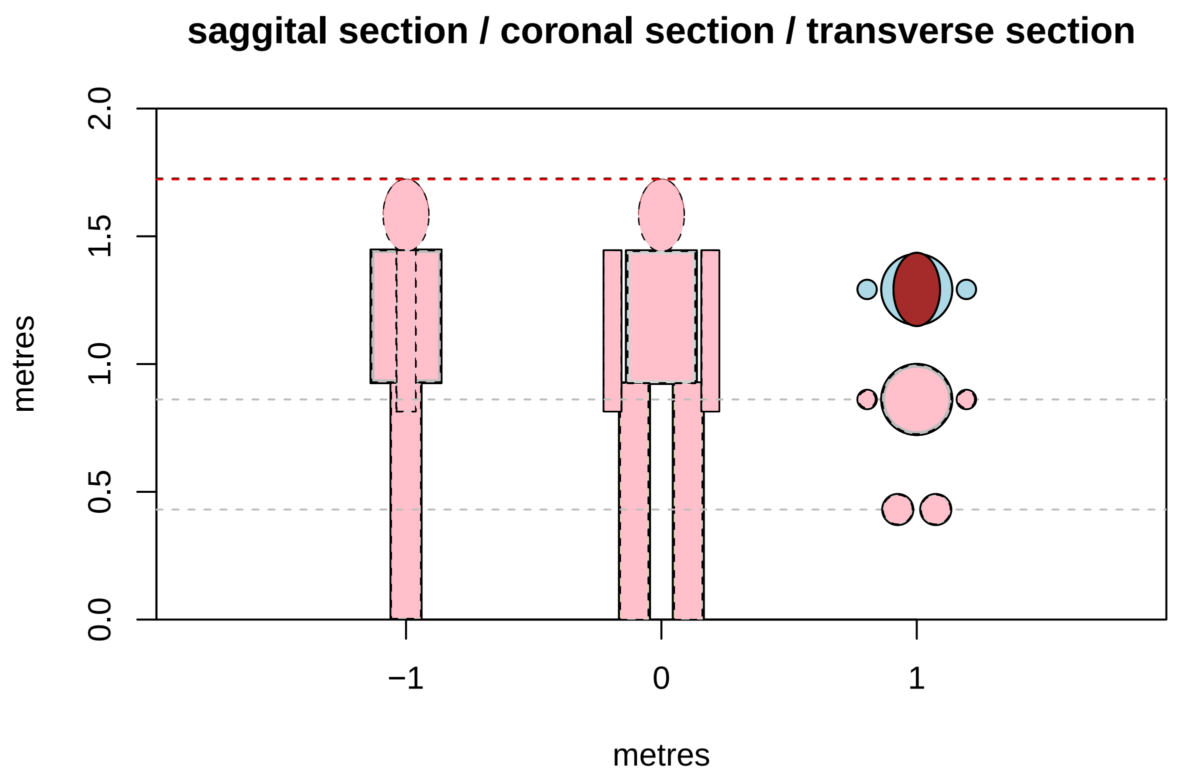


```
## [1] 1.725834
```

Run simulations.

```
HomoTherm.out_clothed <- HomoTherm_var(MASS = MASS,
                                       QMETAB_REST = QMETAB_REST,
                                       INSDEPDs = INSDEPDs,
                                       INSDEPVs = INSDEPVs,
                                       SHAPE_Bs = SHAPE_Bs,
                                       PJOINs = PJOINs,
                                       PCTBAREVAPs = PCTBAREVAPs,
                                       REFLDs = REFLDs,
                                       REFLVs = REFLVs,
                                       heights = heights,
                                       REFHYT = REFHYT,
                                       RUF = RUF,
                                       TAs = env.out$TAs,
                                       TAREFs = env.out$TAREFs,
                                       TSKYs = env.out$TSKYs,
                                       TGRDs = env.out$TGRDs,
```

```
                                                  RHs = env.out$RHs,
                                                  RHREFs = env.out$RHREFs,
                                                  VELs = env.out$VELs,
                                                  VREFs = env.out$VREFs,
                                                  QSOLRs = env.out$QSOLRs,
                                                  Zs = env.out$Zs,
                                                  PDIFs = env.out$PDIFs,
                                                  ELEV = micro$elev,
                                                  ABSSB = 1 - micro$REFL,
                                                  CONV_ENHANCE = env.out$CONV_ENHANCE)
G_m.G2s <- HomoTherm.out_clothed$balance$QMETAB / AREA
#G_m.G2s <- rep(G_m.G2, length(env.out$TAs))
clo <- colMeans(get_clo(HomoTherm.out_clothed,
                        INSDEPDs = INSDEPDs,
                        INSDEPVs = INSDEPVs))
MANMO.out_clothed <- run.MANMO(W = rep(1 / 100, length(env.out$TAs)),
                               Ht.H4 = HEIGHT,
                               Wt.W4 = MASS,
                               D3 = c(mean(INSDEPDs[2:4]), rep(1e-10, 3)),
                               Maximum.SR = 1000 / 60 / AREA,
                               G_m.G2s = G_m.G2s,
                               CLO.C4 = clo,
                               CLO.mode = CLO.mode,
                               T_clo.T9s = HomoTherm.out_clothed$balance$T_CLO,
                               a_skn.B4 = REFLDs[2],
                               a_clo.B5 = REFLDs[2],
                               TAs = env.out$TAs,
                               TSKYs = env.out$TSKYs,
                               TGNDs = env.out$TGRDs,
                               RH.H2s = env.out$RHs / 100,
                               Q_hs = Q_hs,
                               q_hs = q_hs,
                               Zs = env.out$Zs,
                               VELs = env.out$VELs,
                               a.B3 = micro$REFL)

# Iso7933
Iso7933.out <- lapply(1:length(env.out$TAs),
                         function(x){
                           calcIso7933_Tcl(accl = 100,
                                           Duration = 60 * duration,
                                           posture = 2,
                                           Ta = env.out$TAs[x],
                                           Pa = WETAIR(db = env.out$TAs[x],
                                                rh = env.out$RHs[x])$e / 1000,
                                           Tr = (env.out$TGRDs[x] +
                                                   env.out$TSKYs[x]) / 2 +
                                             (env.out$QSOLR[x] / 1366) * 30,
                                           Va = env.out$VELs[x],
                                           Tsk = env.out$TAs[x],
                                           Met = QMETAB_REST / AREA,
                                           Icl = clo,
                                           weight = MASS,
```

```
                                                            height = HEIGHT / 100,
                                                            Adu = AREA,
                                                            Tre = 36.8,
                                                            Tcr = 36.8,
                                                            SWp = 0.5
                                        )})
Iso7933.out_clothed<- as.data.frame(do.call(rbind, Iso7933.out))
```

Plot results.

```
plot.output(HomoTherm.out_clothed, MANMO.out_clothed, Iso7933.out_clothed,
            env.out)
```

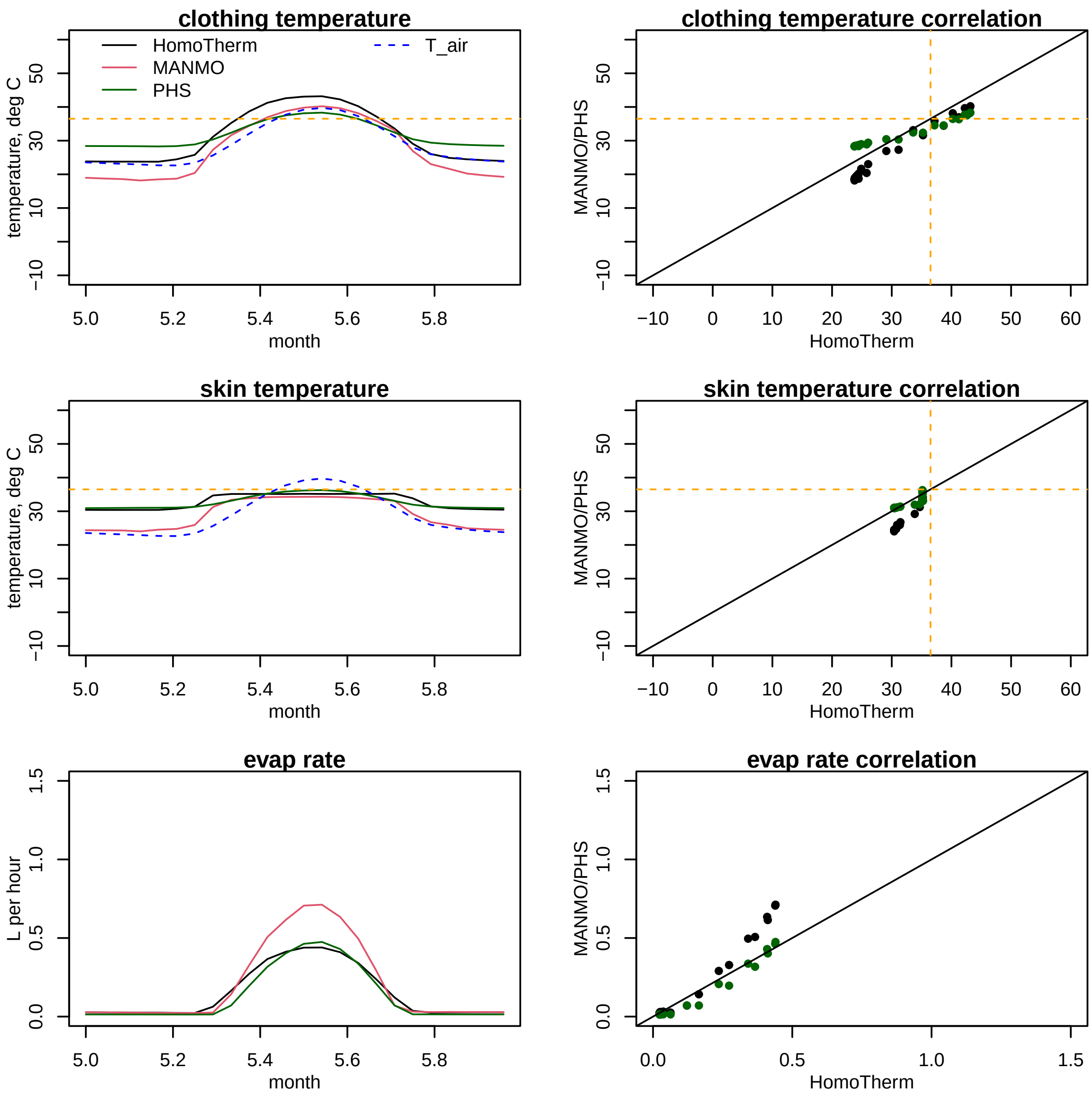

```r
plot.balance(HomoTherm.out_clothed, MANMO.out_clothed)
```

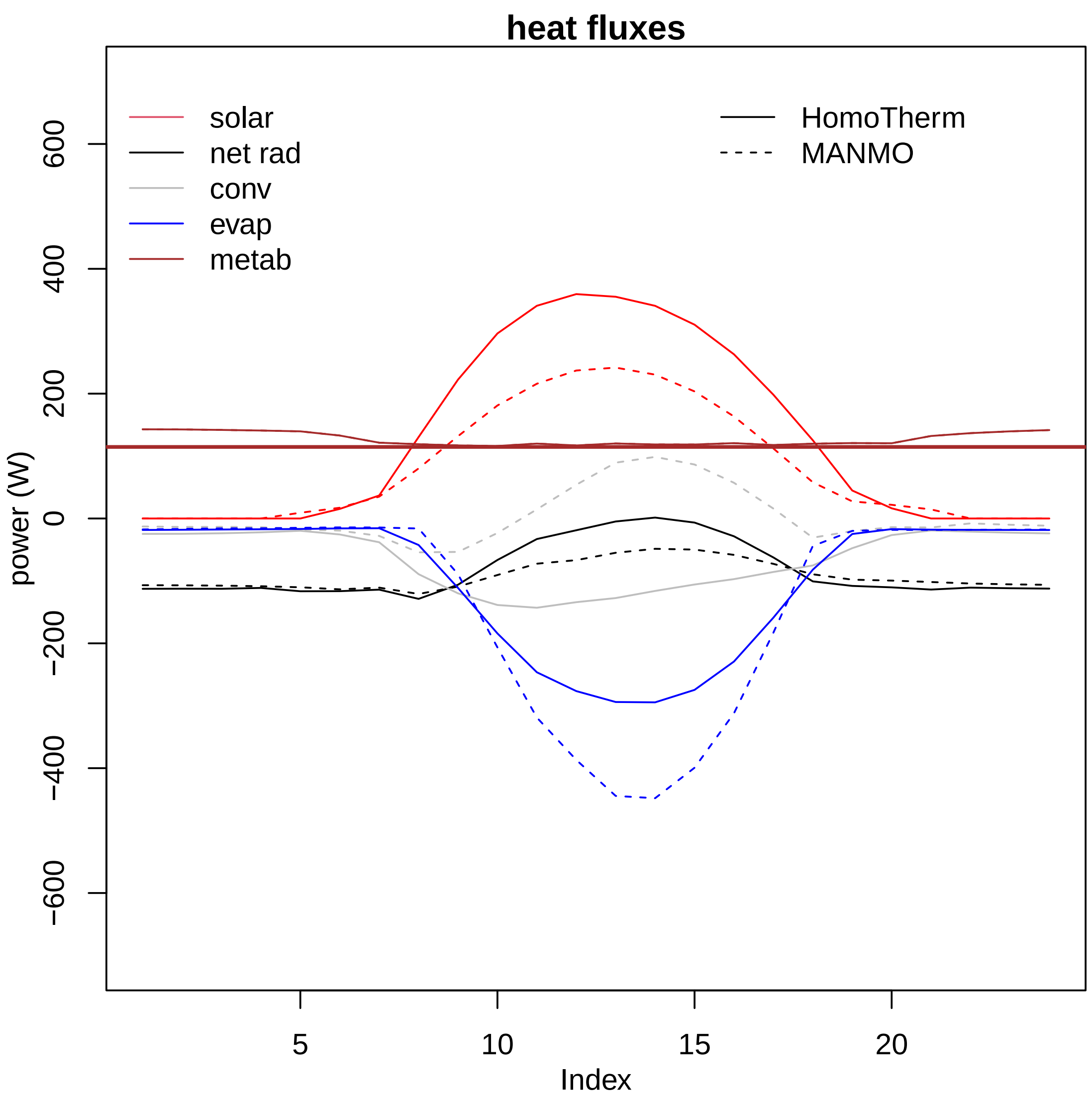

Dark-coloured clothing

Another simulation out of interest, showing the difference between previous light-coloured clothing and now dark clothing.

Set parameters.

```r
REFLDs <- rep(0.15, 4) # clothing/skin dorsal reflectivity dec %
REFLVs <- REFLDs # clothing/skin ventral reflectivity dec %
a_clo.B5 <- REFLDs[1] # albedo
a_skn.B4 <- a_clo.B5 # from Sellers (1965) cited in Myrup and Morgan
```

Run simulations.

```
HomoTherm.out_darkclothed <- HomoTherm_var(MASS = MASS,
                                           QMETAB_REST = QMETAB_REST,
                                           INSDEPDs = INSDEPDs,
                                           INSDEPVs = INSDEPVs,
                                           SHAPE_Bs = SHAPE_Bs,
                                           PJOINs = PJOINs,
                                           PCTBAREVAPs = PCTBAREVAPs,
                                           REFLDs = REFLDs,
                                           REFLVs = REFLVs,
                                           heights = heights,
                                           REFHYT = REFHYT,
                                           RUF = RUF,
                                           TAs = env.out$TAs,
                                           TAREFs = env.out$TAREFs,
                                           TSKYs = env.out$TSKYs,
                                           TGRDs = env.out$TGRDs,
                                           RHs = env.out$RHs,
                                           RHREFs = env.out$RHREFs,
                                           VELs = env.out$VELs,
                                           VREFs = env.out$VREFs,
                                           QSOLRs = env.out$QSOLRs,
                                           Zs = env.out$Zs,
                                           PDIFs = env.out$PDIFs,
                                           ELEV = micro$elev,
                                           ABSSB = 1 - micro$REFL,
                                           CONV_ENHANCE = env.out$CONV_ENHANCE)
clo <- colMeans(get_clo(HomoTherm.out_darkclothed,
                        INSDEPDs = INSDEPDs,
                        INSDEPVs = INSDEPVs))
G_m.G2s <- HomoTherm.out_darkclothed$balance$QMETAB / AREA
#G_m.G2s <- rep(G_m.G2, length(env.out$TAs))
MANMO.out_darkclothed <- run.MANMO(W = rep(1 / 100, length(env.out$TAs)),
                               Ht.H4 = HEIGHT,
                               Wt.W4 = MASS,
                               D3 = c(mean(INSDEPDs[2:4]), rep(1e-10, 3)),
                               Maximum.SR = 1000 / 60 / AREA,
                               G_m.G2s = G_m.G2s * 1.15,
                               CLO.C4 = clo,
                               CLO.mode = CLO.mode,
                               T_clo.T9s = HomoTherm.out_darkclothed$balance$T_CLO,
                               a_skn.B4 = REFLDs[2],
                               a_clo.B5 = REFLDs[2],
                               TAs = env.out$TAs,
                               TSKYs = env.out$TSKYs,
                               TGNDs = env.out$TGRDs,
                               RH.H2s = env.out$RHs / 100,
                               Q_hs = Q_hs,
                               q_hs = q_hs,
                               Zs = env.out$Zs,
                               VELs = env.out$VELs,
                               a.B3 = micro$REFL,
                               dd.V5 = 0)
```

Plot results.

```r
plot.output(HomoTherm.out_darkclothed, MANMO.out_darkclothed,
            Iso7933.out_clothed, env.out)
```

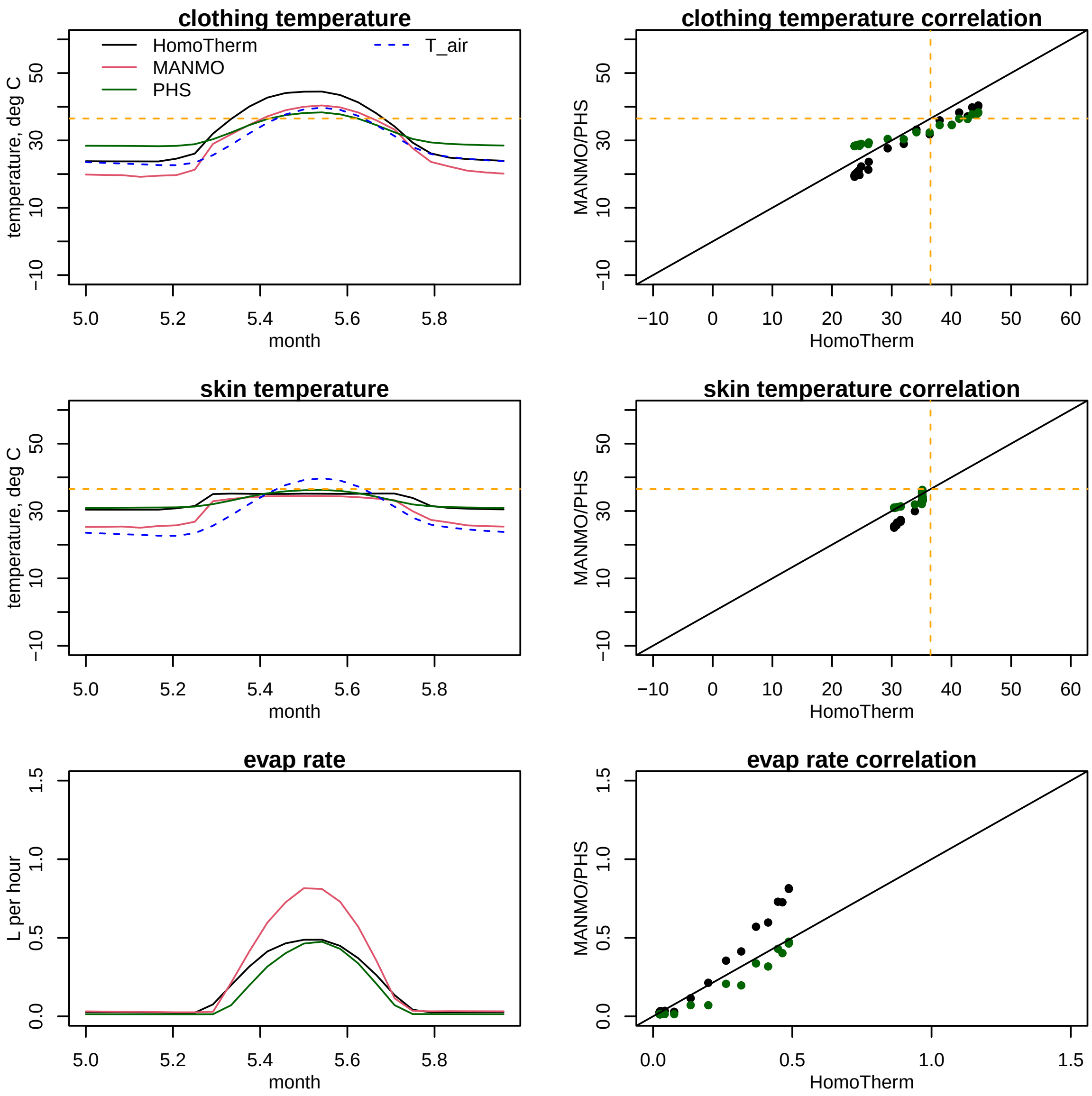


```r
plot.balance(HomoTherm.out_darkclothed, MANMO.out_darkclothed)
```

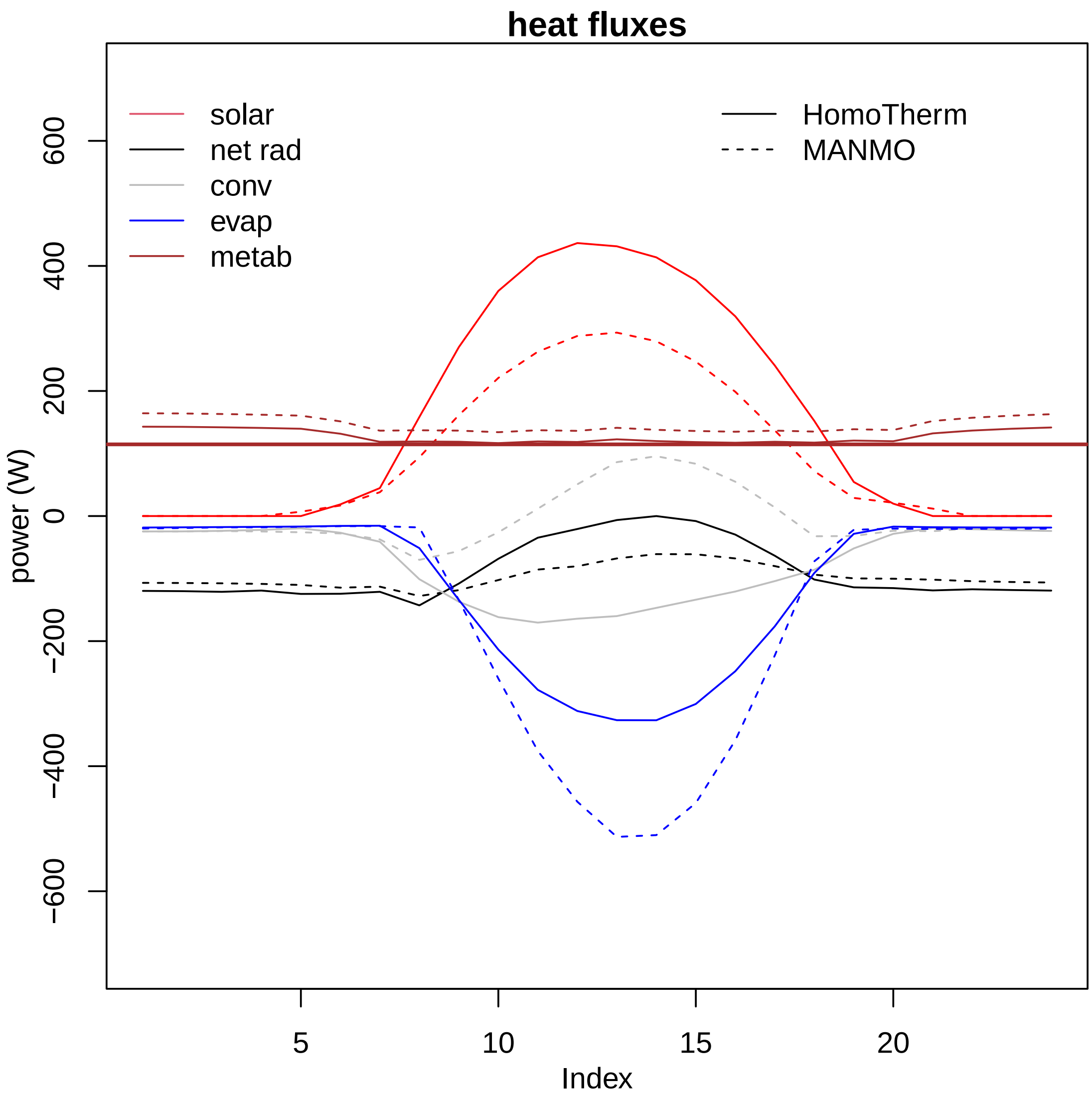

White robe

Now simulate the white robe scenario of Shkolnik et al. 1980.

Set parameters. Note changing radiation configuration factors to account for being a robe.

```
PCTBAREVAPs <- c(60, 70, 70, 70)
INSDEPDs <- c(0.01, 0.0025, 0.0025, 0.0025) # fur depth, dorsal (m)
INSDEPVs <- c(1e-9, 0.0025, 0.0025, 0.0025) # fur depth, ventral (m)
FSKREFs <- c(0.5, 0.5, 0.5, 0.5)
FGDREFs <- c(0.5, 0.5, 0.5, 0.5)
REFLDs <- rep(1 - 0.35, 4) # clothing/skin dorsal reflectivity dec %
REFLVs <- REFLDs # clothing/skin ventral reflectivity dec %
```

```r
a_clo.B5 <- REFLDs[1] # albedo
a_skn.B4 <- 0.44 # from Sellers (1965) cited in Myrup and Morgan
clo <- colMeans(get_clo(HomoTherm.out_nude,
                        INSDEPDs = INSDEPDs,
                        INSDEPVs = INSDEPVs))
par(mfrow = c(1, 1))
plot_human(MASS = MASS,
           HEIGHT = HEIGHT,
           INSDEPDs = INSDEPDs,
           INSDEPVs = INSDEPVs,
           SHAPE_Bs = SHAPE_Bs)
```

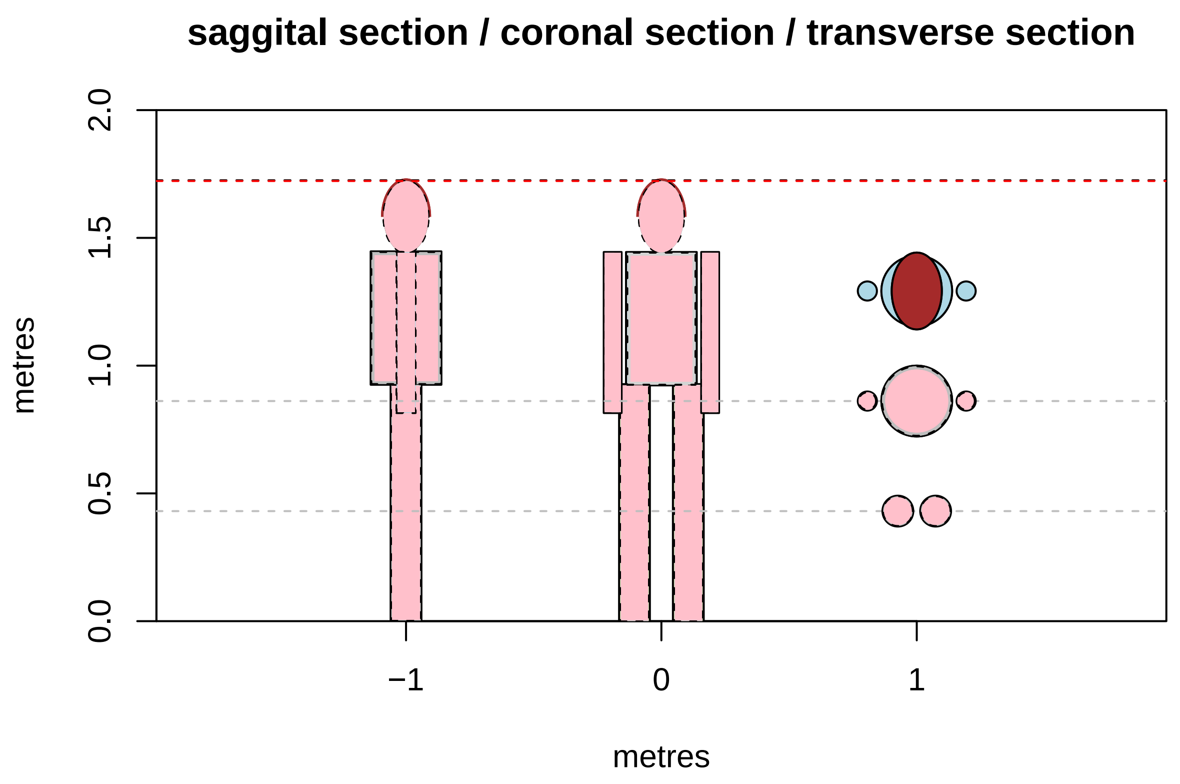


```
## [1] 1.725334
```

Run simulations.

```r
HomoTherm.out_white <- HomoTherm_var(MASS = MASS,
                                     QMETAB_REST = QMETAB_REST,
                                     INSDEPDs = INSDEPDs,
                                     INSDEPVs = INSDEPVs,
                                     SHAPE_Bs = SHAPE_Bs,
                                     PJOINs = PJOINs,
                                     PCTBAREVAPs = PCTBAREVAPs,
                                     REFLDs = REFLDs,
```

```
                                         REFLVs = REFLVs,
                                         FSKREFs = FSKREFs,
                                         FGDREFs = FGDREFs,
                                         heights = heights,
                                         REFHYT = REFHYT,
                                         RUF = RUF,
                                         TAs = env.out$TAs,
                                         TAREFs = env.out$TAREFs,
                                         TSKYs = env.out$TSKYs,
                                         TGRDs = env.out$TGRDs,
                                         RHs = env.out$RHs,
                                         RHREFs = env.out$RHREFs,
                                         VELs = env.out$VELs,
                                         VREFs = env.out$VREFs,
                                         QSOLRs = env.out$QSOLRs,
                                         Zs = env.out$Zs,
                                         PDIFs = env.out$PDIFs,
                                         ELEV = micro$elev,
                                         ABSSB = 1 - micro$REFL,
                                         CONV_ENHANCE = env.out$CONV_ENHANCE)
HomoTherm.out_white_chamber <- HomoTherm_var(MASS = MASS,
                                         QMETAB_REST = QMETAB_REST,
                                         INSDEPDs = INSDEPDs,
                                         INSDEPVs = INSDEPVs,
                                         SHAPE_Bs = SHAPE_Bs,
                                         PJOINs = PJOINs,
                                         PCTBAREVAPs = PCTBAREVAPs,
                                         REFLDs = REFLDs,
                                         REFLVs = REFLVs,
                                         FSKREFs = FSKREFs,
                                         FGDREFs = FGDREFs,
                                         heights = heights,
                                         REFHYT = REFHYT,
                                         RUF = RUF,
                                         TAs = env.out.chamber$TAs,
                                         TAREFs = env.out.chamber$TAREFs,
                                         TSKYs = env.out.chamber$TSKYs,
                                         TGRDs = env.out.chamber$TGRDs,
                                         RHs = env.out.chamber$RHs,
                                         RHREFs = env.out.chamber$RHREFs,
                                         VELs = env.out.chamber$VELs,
                                         VREFs = env.out.chamber$VREFs,
                                         QSOLRs = env.out.chamber$QSOLRs,
                                         Zs = env.out.chamber$Zs,
                                         PDIFs = env.out.chamber$PDIFs,
                                         ELEV = micro$elev,
                                         ABSSB = 1 - micro$REFL,
                                         CONV_ENHANCE =
                                           env.out.chamber$CONV_ENHANCE)
clo <- colMeans(get_clo(HomoTherm.out_white,
                        INSDEPDs = INSDEPDs,
                        INSDEPVs = INSDEPVs))
G_m.G2s <- HomoTherm.out_white$balance$QMETAB / AREA
```

```r
#G_m.G2s <- rep(G_m.G2, length(env.out$TAs))
MANMO.out_white <- run.MANMO(W = rep(1 / 100, length(env.out$TAs)),
                             Ht.H4 = HEIGHT,
                             Wt.W4 = MASS,
                             D3 = c(mean(INSDEPDs[2:4]), rep(1e-10, 3)),
                             Maximum.SR = 1000 / 60 / AREA,
                             G_m.G2s = G_m.G2s * 1.15,
                             CLO.C4 = clo,
                             CLO.mode = CLO.mode,
                             T_clo.T9s = HomoTherm.out_white$balance$T_CLO,
                             a_skn.B4 = REFLDs[1],
                             a_clo.B5 = REFLDs[1],
                             K6 = 1,
                             TAs = env.out$TAs,
                             TSKYs = env.out$TSKYs,
                             TGNDs = env.out$TGRDs,
                             RH.H2s = env.out$RHs / 100,
                             Q_hs = Q_hs,
                             q_hs = q_hs,
                             Zs = env.out$Zs,
                             VELs = env.out$VELs,
                             a.B3 = micro$REFL)
G_m.G2s <- HomoTherm.out_white_chamber$balance$QMETAB / AREA
#G_m.G2s <- rep(G_m.G2, length(env.out$TAs))
MANMO.out_white_chamber <- run.MANMO(W = rep(1 / 100, length(env.out$TAs)),
                                     Ht.H4 = HEIGHT,
                                     Wt.W4 = MASS,
                                     D3 = c(mean(INSDEPDs[2:4]), rep(1e-10, 3)),
                                     Maximum.SR = 1000 / 60 / AREA,
                                     G_m.G2s = G_m.G2s * 1.15,
                                     CLO.C4 = clo,
                                     CLO.mode = CLO.mode,
                                     T_clo.T9s =
                                       HomoTherm.out_white_chamber$balance$T_CLO,
                                     a_skn.B4 = REFLDs[1],
                                     a_clo.B5 = REFLDs[1],
                                     K6 = 1,
                                     TAs = env.out.chamber$TAs,
                                     TSKYs = env.out.chamber$TSKYs,
                                     TGNDs = env.out.chamber$TGRDs,
                                     RH.H2s = env.out.chamber$RHs / 100,
                                     Q_hs = Q_hs * 0,
                                     q_hs = q_hs * 0,
                                     Zs = env.out.chamber$Zs,
                                     VELs = env.out.chamber$VELs,
                                     a.B3 = micro$REFL)

# Iso7933
Iso7933.out <- lapply(1:length(env.out$TAs),
                           function(x){
                             calcIso7933_Tcl(accl = 100,
                                             Duration = 60 * duration,
                                             posture = 2,
```

```
                                             Ta = env.out$TAs[x],
                                             Pa = WETAIR(db = env.out$TAs[x], rh = env.out$RHs[x])$e / 1
                                             Tr = (env.out$TGRDs[x] +
                                                     env.out$TSKYs[x]) / 2
                                             + (env.out$QSOLR[x] / 1366) * 30 *
                                               1.2 * (1 - REFLDs[1]),
                                             Va = env.out$VELs[x],
                                             Tsk = env.out$TAs[x],
                                             Met = QMETAB_REST / AREA,
                                             Icl = clo,
                                             weight = MASS,
                                             height = HEIGHT / 100,
                                             Adu = AREA,
                                             Tre = 36.8,
                                             Tcr = 36.8,
                                             SWp = 0.5
                              )})
Iso7933.out_white <- as.data.frame(do.call(rbind, Iso7933.out))


# Iso7933
Iso7933.out <- lapply(1:length(env.out$TAs),
                       function(x){
                         calcIso7933_Tcl(accl = 100,
                                             Duration = 60 * duration,
                                             posture = 2,
                                             Ta = env.out.chamber$TAs[x],
                                             Pa = WETAIR(db =
                                                env.out.chamber$TAs[x],
                                                rh = env.out.chamber$RHs[x])$e
                                             / 1000,
                                             Tr = (env.out.chamber$TGRDs[x] +
                                                env.out.chamber$TSKYs[x]) / 2 +
                                               (env.out.chamber$QSOLR[x] / 1366)
                                             * 30 * 1.2 * (1 - REFLDs[1]),
                                             Va = env.out.chamber$VELs[x],
                                             Tsk = env.out.chamber$TAs[x],
                                             Met = QMETAB_REST / AREA,
                                             Icl = clo,
                                             weight = MASS,
                                             height = HEIGHT / 100,
                                             Adu = AREA,
                                             Tre = 36.8,
                                             Tcr = 36.8,
                                             SWp = 0.5
                              )})
Iso7933.out_white_chamber <- as.data.frame(do.call(rbind, Iso7933.out))
```

Plot results.

```
plot.output(HomoTherm.out_white, MANMO.out_white, Iso7933.out_white, env.out)
```

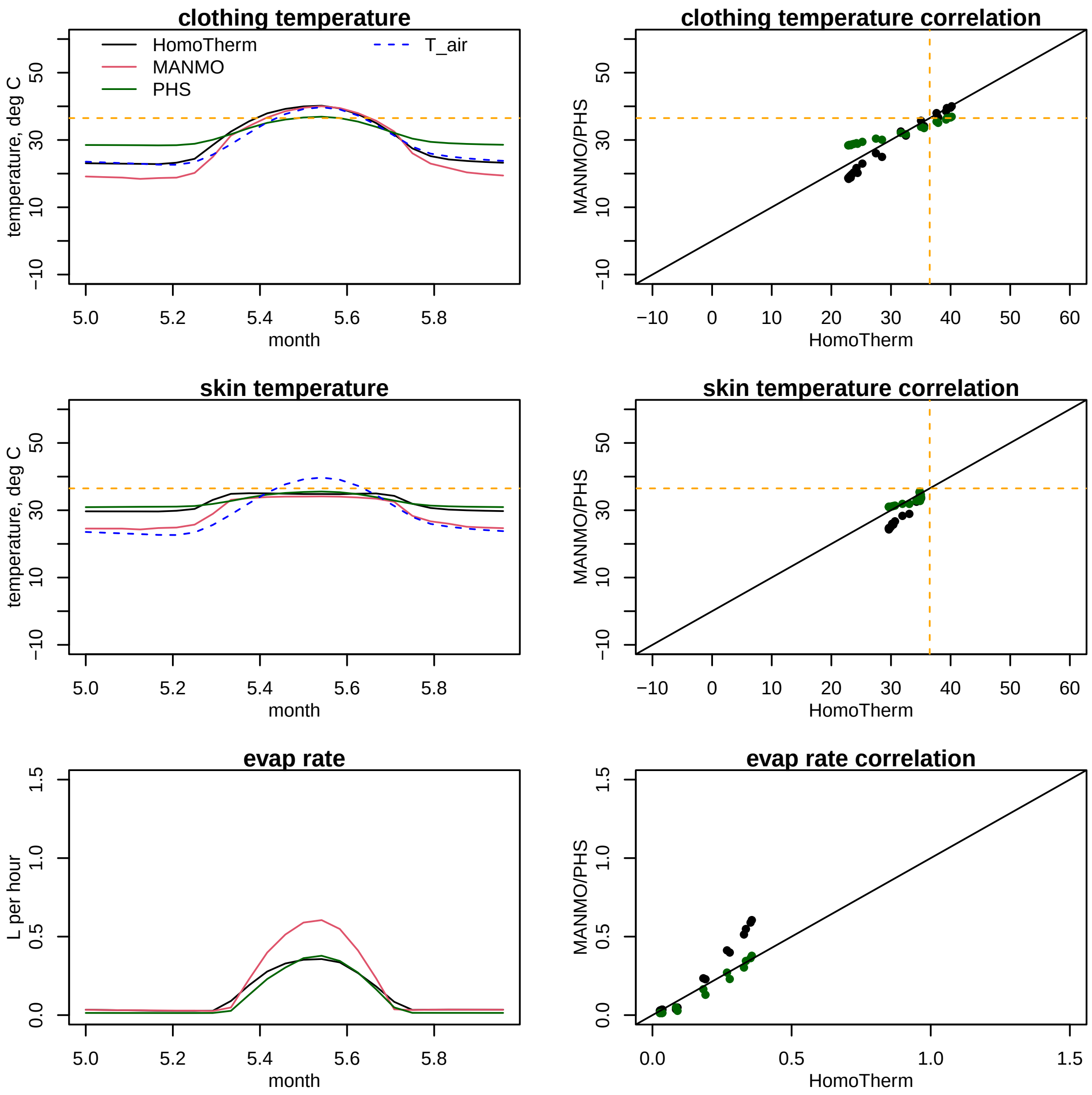


```r
plot.balance(HomoTherm.out_white, MANMO.out_white)
```

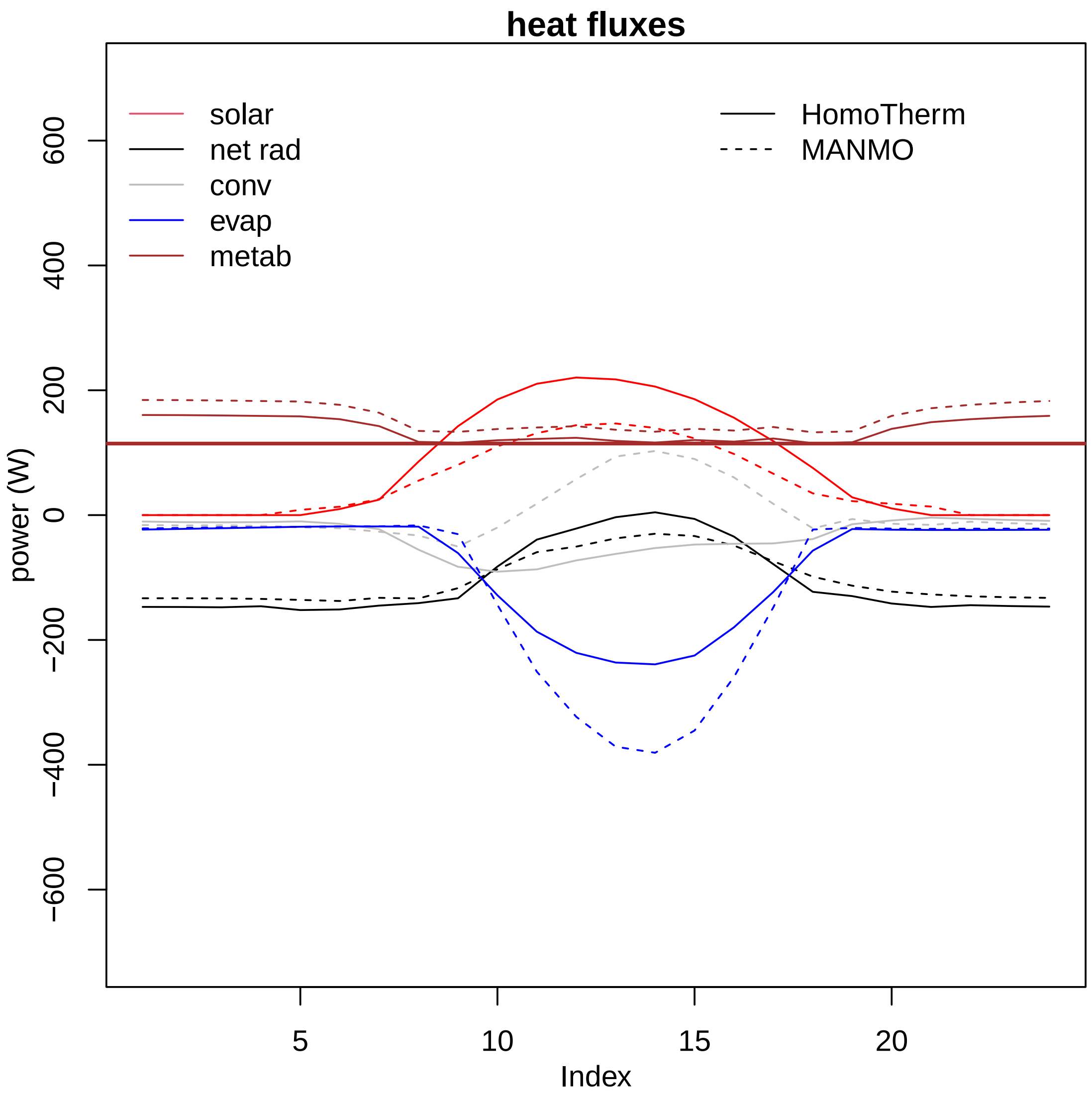


```
plot.output(HomoTherm.out_white_chamber, MANMO.out_white_chamber,  Iso7933.out_white_chamber, env.out.c
```

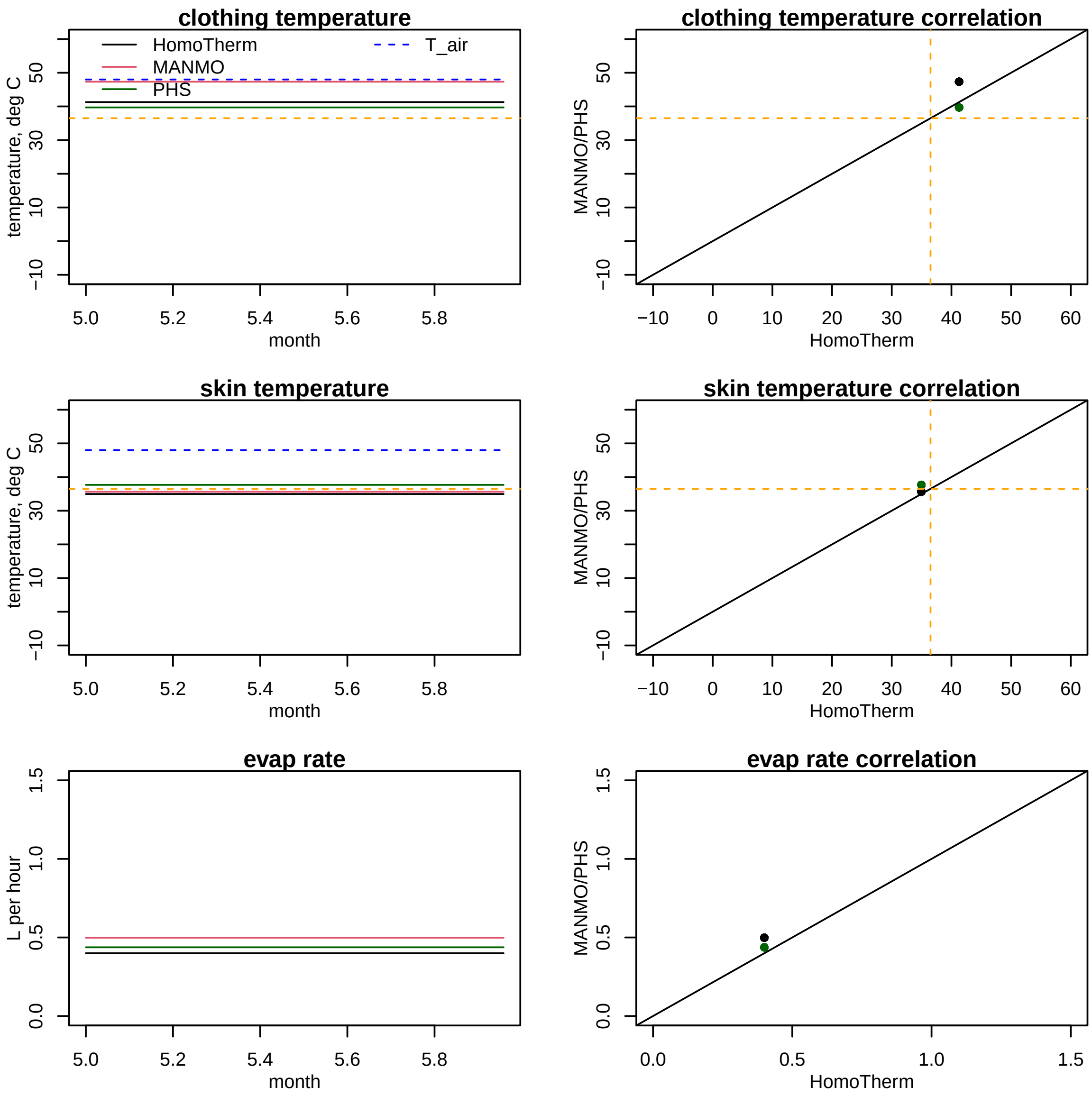


```
plot.balance(HomoTherm.out_white_chamber, MANMO.out_white_chamber)
```

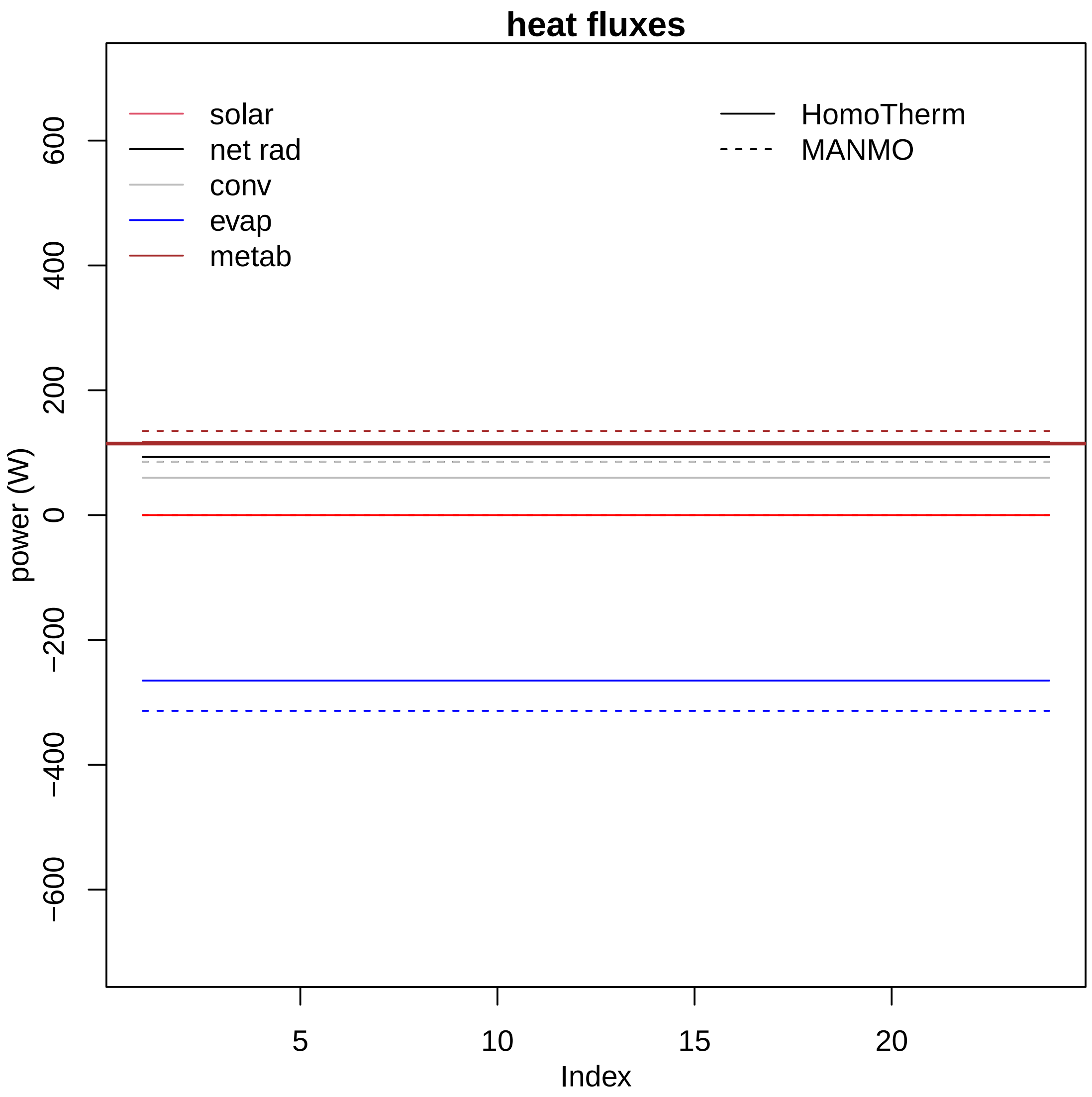

Dark robe

Now simulate the black robe scenario of Shkolnik et al. 1980.

Set parameters. Just changing solar reflectivity.

```
REFLDs <- rep(1 - 0.89, 4) # clothing/skin dorsal reflectivity dec %
REFLVs <- REFLDs # clothing/skin ventral reflectivity dec %
a_clo.B5 <- REFLDs[1] # albedo
```

Run simulations.

```r
HomoTherm.out_black <- HomoTherm_var(MASS = MASS,
                                     QMETAB_REST = QMETAB_REST,
                                     INSDEPDs = INSDEPDs,
                                     INSDEPVs = INSDEPVs,
                                     SHAPE_Bs = SHAPE_Bs,
                                     PJOINs = PJOINs,
                                     PCTBAREVAPs = PCTBAREVAPs,
                                     REFLDs = REFLDs,
                                     REFLVs = REFLVs,
                                     FSKREFs = FSKREFs,
                                     FGDREFs = FGDREFs,
                                     heights = heights,
                                     REFHYT = REFHYT,
                                     RUF = RUF,
                                     TAs = env.out$TAs,
                                     TAREFs = env.out$TAREFs,
                                     TSKYs = env.out$TSKYs,
                                     TGRDs = env.out$TGRDs,
                                     RHs = env.out$RHs,
                                     RHREFs = env.out$RHREFs,
                                     VELs = env.out$VELs,
                                     VREFs = env.out$VREFs,
                                     QSOLRs = env.out$QSOLRs,
                                     Zs = env.out$Zs,
                                     PDIFs = env.out$PDIFs,
                                     ELEV = micro$elev,
                                     ABSSB = 1 - micro$REFL,
                                     CONV_ENHANCE = env.out$CONV_ENHANCE)
HomoTherm.out_black_chamber <- HomoTherm_var(MASS = MASS,
                                       QMETAB_REST = QMETAB_REST,
                                       INSDEPDs = INSDEPDs,
                                       INSDEPVs = INSDEPVs,
                                       SHAPE_Bs = SHAPE_Bs,
                                       PJOINs = PJOINs,
                                       PCTBAREVAPs = PCTBAREVAPs,
                                       REFLDs = REFLDs,
                                       REFLVs = REFLVs,
                                       FSKREFs = FSKREFs,
                                       FGDREFs = FGDREFs,
                                       heights = heights,
                                       REFHYT = REFHYT,
                                       RUF = RUF,
                                       TAs = env.out.chamber$TAs,
                                       TAREFs = env.out.chamber$TAREFs,
                                       TSKYs = env.out.chamber$TSKYs,
                                       TGRDs = env.out.chamber$TGRDs,
                                       RHs = env.out.chamber$RHs,
                                       RHREFs = env.out.chamber$RHREFs,
                                       VELs = env.out.chamber$VELs,
                                       VREFs = env.out.chamber$VREFs,
                                       QSOLRs = env.out.chamber$QSOLRs,
                                       Zs = env.out.chamber$Zs,
                                       PDIFs = env.out.chamber$PDIFs,
```

```
                                          ELEV = micro$elev,
                                          ABSSB = 1 - micro$REFL,
                                          CONV_ENHANCE = env.out.chamber$CONV_ENHANCE)
clo <- colMeans(get_clo(HomoTherm.out_black,
                        INSDEPDs = INSDEPDs,
                        INSDEPVs = INSDEPVs))
G_m.G2s <- HomoTherm.out_black$balance$QMETAB / AREA
#G_m.G2s <- rep(G_m.G2, length(env.out$TAs))
MANMO.out_black <- run.MANMO(W = rep(1 / 100, length(env.out$TAs)),
                               Ht.H4 = HEIGHT,
                               Wt.W4 = MASS,
                               D3 = c(mean(INSDEPDs[2:4]), rep(1e-10, 3)),
                               Maximum.SR = 1000 / 60 / AREA,
                               G_m.G2s = G_m.G2s * 1.15,
                               CLO.C4 = clo,
                               CLO.mode = CLO.mode,
                               T_clo.T9s = HomoTherm.out_black$balance$T_CLO,
                               a_skn.B4 = REFLDs[1],
                               a_clo.B5 = REFLDs[1],
                               K6 = 1,
                               TAs = env.out$TAs,
                               TSKYs = env.out$TSKYs,
                               TGNDs = env.out$TGRDs,
                               RH.H2s = env.out$RHs / 100,
                               Q_hs = Q_hs,
                               q_hs = q_hs,
                               Zs = env.out$Zs,
                               VELs = env.out$VELs,
                               a.B3 = micro$REFL)
G_m.G2s <- HomoTherm.out_black_chamber$balance$QMETAB / AREA
#G_m.G2s <- rep(G_m.G2, length(env.out$TAs))
MANMO.out_black_chamber <- run.MANMO(W = rep(1 / 100, length(env.out$TAs)),
                               Ht.H4 = HEIGHT,
                               Wt.W4 = MASS,
                               D3 = c(mean(INSDEPDs[2:4]), rep(1e-10, 3)),
                               Maximum.SR = 1000 / 60 / AREA,
                               G_m.G2s = G_m.G2s * 1.15,
                               CLO.C4 = clo,
                               CLO.mode = CLO.mode,
                               T_clo.T9s = HomoTherm.out_black_chamber$balance$T_CLO,
                               a_skn.B4 = REFLDs[1],
                               a_clo.B5 = REFLDs[1],
                               K6 = 1,
                               TAs = env.out.chamber$TAs,
                               TSKYs = env.out.chamber$TSKYs,
                               TGNDs = env.out.chamber$TGRDs,
                               RH.H2s = env.out.chamber$RHs / 100,
                               Q_hs = Q_hs * 0,
                               q_hs = q_hs * 0,
                               Zs = env.out.chamber$Zs,
                               VELs = env.out.chamber$VELs,
                               a.B3 = micro$REFL)
```

```
# Iso7933
Iso7933.out <- lapply(1:length(env.out$TAs),
                      function(x){
                        calcIso7933_Tcl(accl = 100,
                                        Duration = 60 * duration,
                                        posture = 2,
                                        Ta = env.out$TAs[x],
                                        Pa = WETAIR(db = env.out$TAs[x],
                                                    rh = env.out$RHs[x])$e
                                        / 1000,
                                        Tr = (env.out$TGRDs[x] +
                                                env.out$TSKYs[x]) / 2 +
                                          (env.out$QSOLR[x] / 1366) * 30 *
                                          1.2 * (1 - REFLDs[1]),
                                        Va = env.out$VELs[x],
                                        Tsk = env.out$TAs[x],
                                        Met = QMETAB_REST / AREA,
                                        Icl = clo,
                                        weight = MASS,
                                        height = HEIGHT / 100,
                                        Adu = AREA,
                                        Tre = 36.8,
                                        Tcr = 36.8,
                                        SWp = 0.5
                        )})
Iso7933.out_black <- as.data.frame(do.call(rbind, Iso7933.out))


# Iso7933
Iso7933.out <- lapply(1:length(env.out$TAs),
                      function(x){
                        calcIso7933_Tcl(accl = 100,
                                        Duration = 60 * duration,
                                        posture = 2,
                                        Ta = env.out.chamber$TAs[x],
                                        Pa = WETAIR(db =
                                               env.out.chamber$TAs[x],
                                               rh = env.out.chamber$RHs[x])$e
                                        / 1000,
                                        Tr = (env.out.chamber$TGRDs[x] +
                                                env.out.chamber$TSKYs[x])
                                        / 2 + (env.out.chamber$QSOLR[x] / 1366) *
                                          30 * 1.2 * (1 - REFLDs[1]),
                                        Va = env.out.chamber$VELs[x],
                                        Tsk = env.out.chamber$TAs[x],
                                        Met = QMETAB_REST / AREA,
                                        Icl = clo,
                                        weight = MASS,
                                        height = HEIGHT / 100,
                                        Adu = AREA,
                                        Tre = 36.8,
                                        Tcr = 36.8,
                                        SWp = 0.5
```

```
                              )})
Iso7933.out_black_chamber <- as.data.frame(do.call(rbind, Iso7933.out))
```

Plot results.

```
plot.output(HomoTherm.out_black, MANMO.out_black,  Iso7933.out_black, env.out)
```

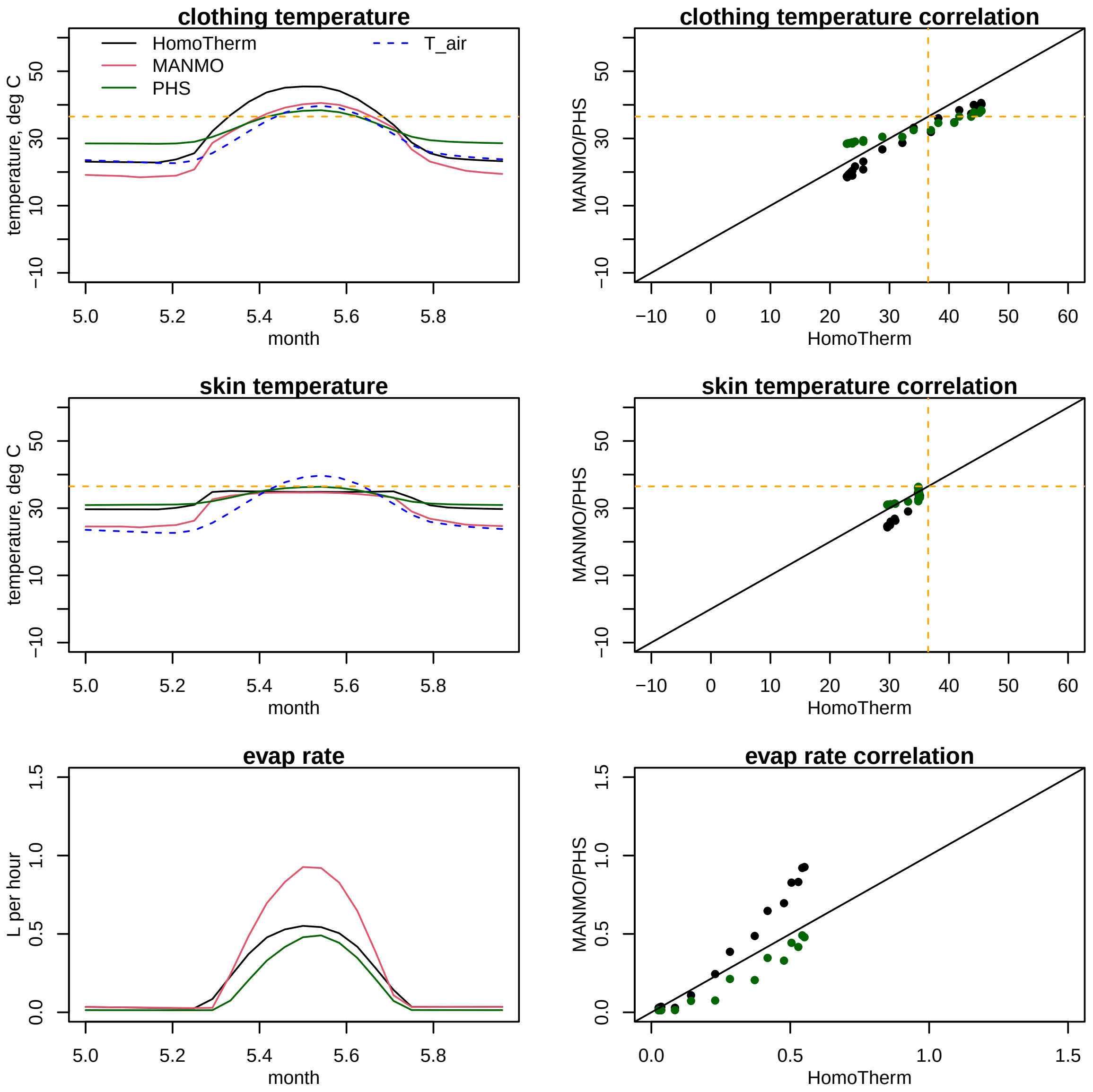


```
plot.balance(HomoTherm.out_black, MANMO.out_black)
```

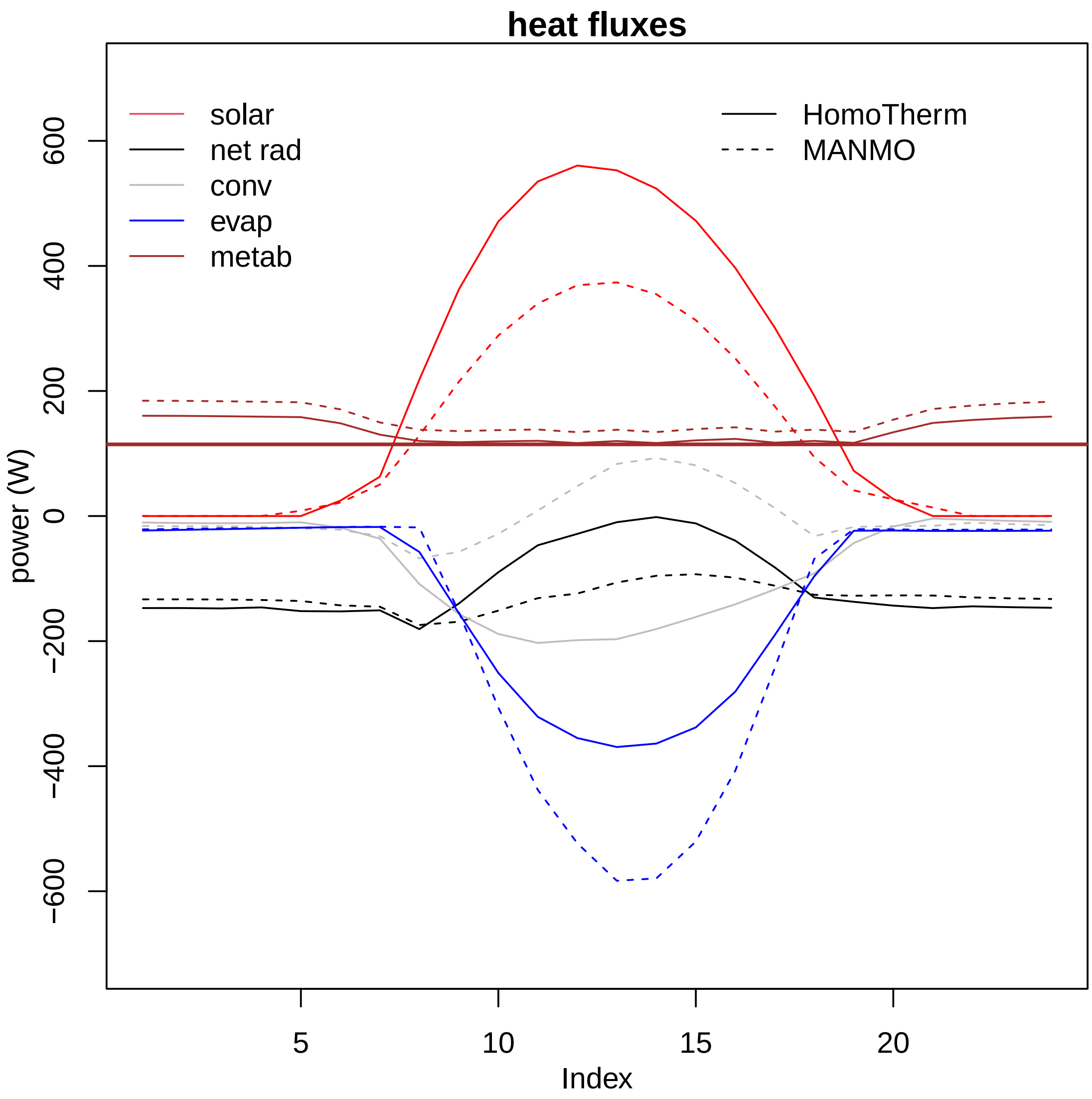


```
plot.output(HomoTherm.out_black_chamber, MANMO.out_black_chamber,  Iso7933.out_black_chamber, env.out.c
```

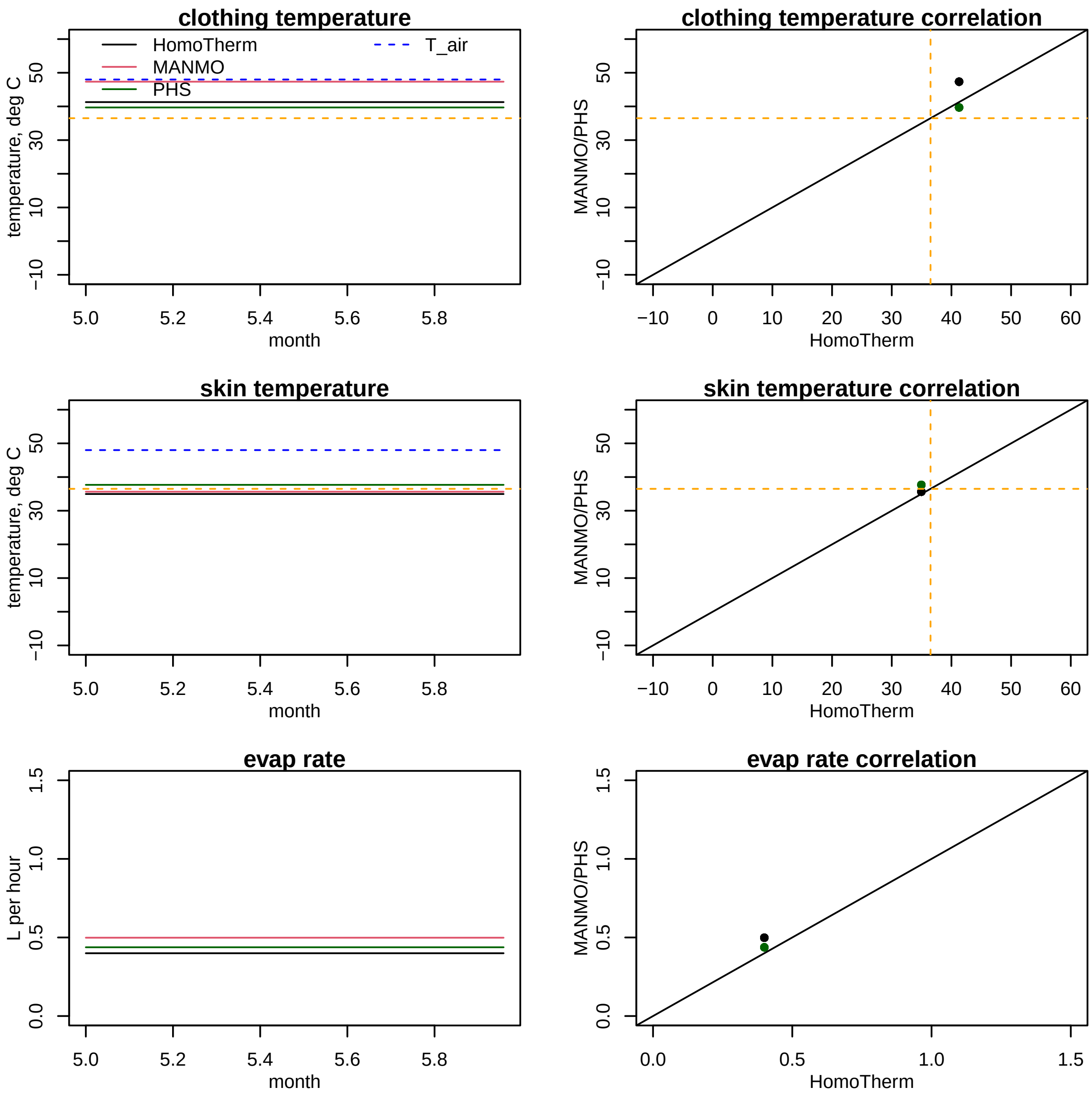


```
plot.balance(HomoTherm.out_black_chamber, MANMO.out_black_chamber)
```

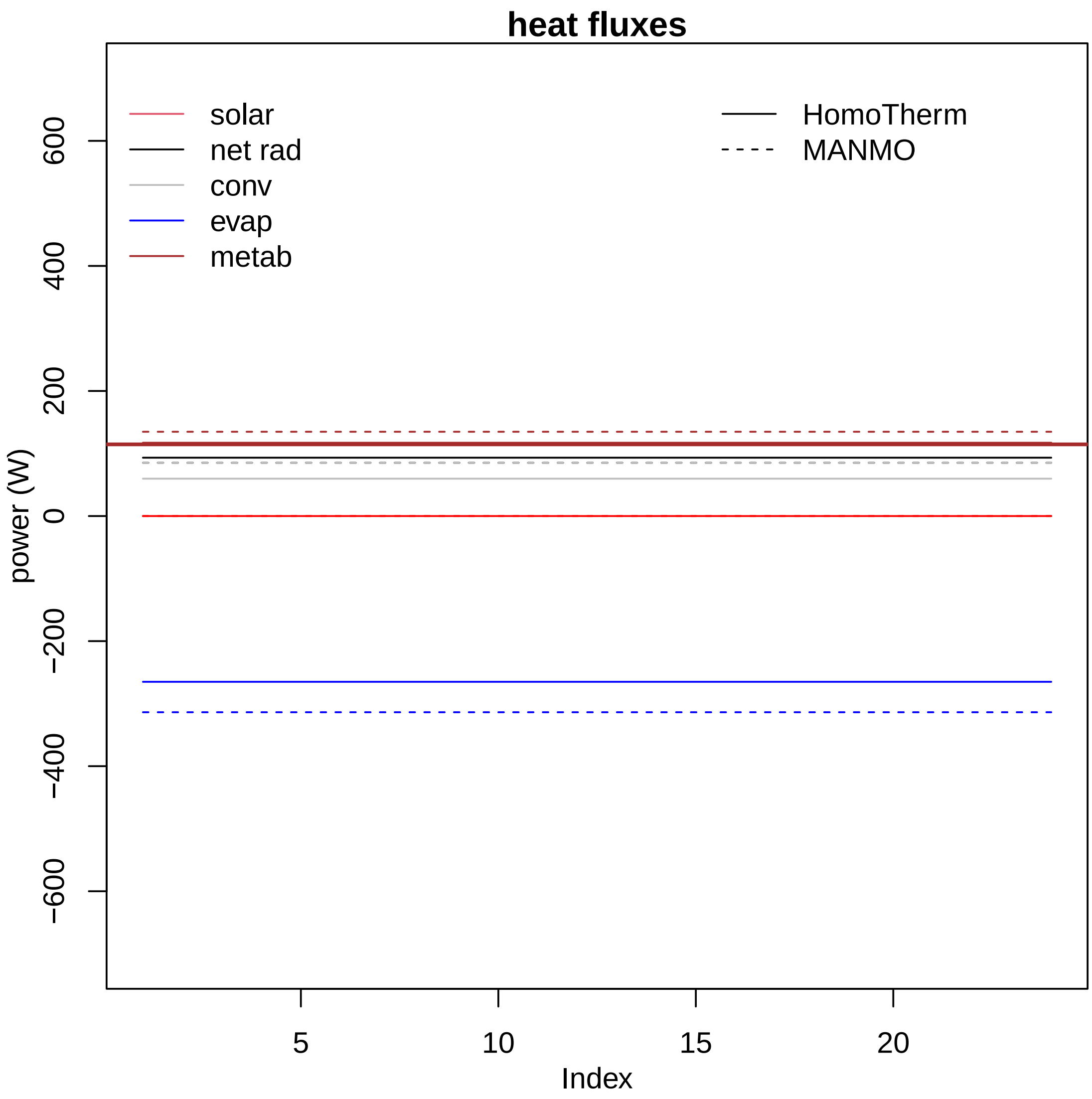

Shorts (semi-nude)

Now simulate the 'shorts' scenario of Shkolnik et al. 1980.

Set parameters - half-clothed on torso and legs.

```
REFLDs <- rep(1 - 0.66, 4) # clothing/skin dorsal reflectivity dec %
REFLVs <- REFLDs # clothing/skin ventral reflectivity dec %
a_clo.B5 <- REFLDs[1] # albedo
PCTBAREVAPs <- c(60, 50, 90, 50)
#KCLOs <- rep(0, 4) # manual override of fur thermal conductivity
INSDEPDs <- c(0.01, 0.002, 1e-9, 0.002) # fur depth, dorsal (m)
INSDEPVs <- c(1e-9, 1e-9, 1e-9, 1e-9) # fur depth, ventral (m)
```

```r
par(mfrow = c(1, 1))
plot_human(MASS = MASS,
           HEIGHT = HEIGHT,
           INSDEPDs = INSDEPDs,
           INSDEPVs = INSDEPVs,
           SHAPE_Bs = SHAPE_Bs)
```

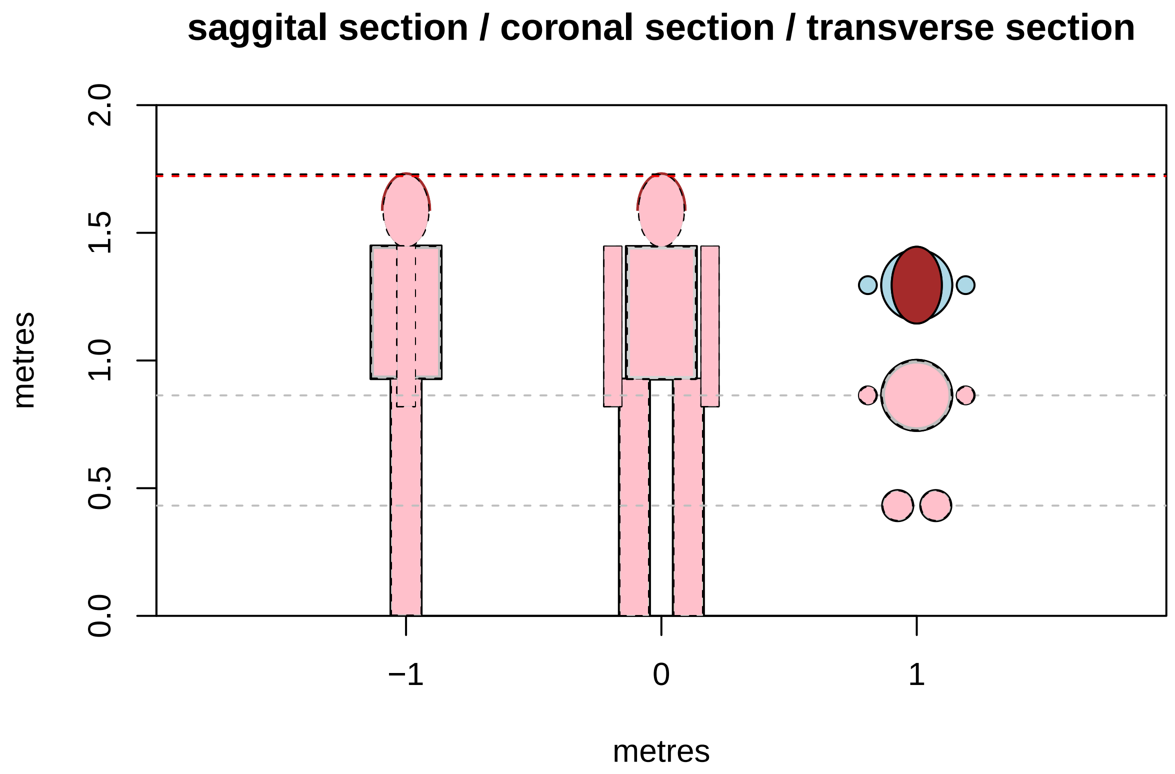


```
## [1] 1.728834
```

Run simulations.

```r
HomoTherm.out_shorts <- HomoTherm_var(MASS = MASS,
                                      QMETAB_REST = QMETAB_REST,
                                      INSDEPDs = INSDEPDs,
                                      INSDEPVs = INSDEPVs,
                                      SHAPE_Bs = SHAPE_Bs,
                                      PJOINs = PJOINs,
                                      PCTBAREVAPs = PCTBAREVAPs,
                                      REFLDs = REFLDs,
                                      REFLVs = REFLVs,
                                      heights = heights,
                                      REFHYT = REFHYT,
                                      RUF = RUF,
                                      TAs = env.out$TAs,
```

```
                                       TAREFs = env.out$TAREFs,
                                       TSKYs = env.out$TSKYs,
                                       TGRDs = env.out$TGRDs,
                                       RHs = env.out$RHs,
                                       RHREFs = env.out$RHREFs,
                                       VELs = env.out$VELs,
                                       VREFs = env.out$VREFs,
                                       QSOLRs = env.out$QSOLRs,
                                       Zs = env.out$Zs,
                                       PDIFs = env.out$PDIFs,
                                       ELEV = micro$elev,
                                       ABSSB = 1 - micro$REFL,
                                       CONV_ENHANCE = env.out$CONV_ENHANCE)
HomoTherm.out_shorts_chamber <- HomoTherm_var(MASS = MASS,
                                       QMETAB_REST = QMETAB_REST,
                                       INSDEPDs = INSDEPDs,
                                       INSDEPVs = INSDEPVs,
                                       SHAPE_Bs = SHAPE_Bs,
                                       PJOINs = PJOINs,
                                       PCTBAREVAPs = PCTBAREVAPs,
                                       REFLDs = REFLDs,
                                       REFLVs = REFLVs,
                                       heights = heights,
                                       REFHYT = REFHYT,
                                       RUF = RUF,
                                       TAs = env.out.chamber$TAs,
                                       TAREFs = env.out.chamber$TAREFs,
                                       TSKYs = env.out.chamber$TSKYs,
                                       TGRDs = env.out.chamber$TGRDs,
                                       RHs = env.out.chamber$RHs,
                                       RHREFs = env.out.chamber$RHREFs,
                                       VELs = env.out.chamber$VELs,
                                       VREFs = env.out.chamber$VREFs,
                                       QSOLRs = env.out.chamber$QSOLRs,
                                       Zs = env.out.chamber$Zs,
                                       PDIFs = env.out.chamber$PDIFs,
                                       ELEV = micro$elev,
                                       ABSSB = 1 - micro$REFL,
                                       CONV_ENHANCE = env.out.chamber$CONV_ENHANCE)
clo <- colMeans(get_clo(HomoTherm.out_shorts,
                        INSDEPDs = INSDEPDs,
                        INSDEPVs = INSDEPVs))
G_m.G2s <- HomoTherm.out_shorts$balance$QMETAB / AREA
#G_m.G2s <- rep(G_m.G2, length(env.out$TAs))
MANMO.out_shorts <- run.MANMO(W = rep(1 / 100, length(env.out$TAs)),
                               Ht.H4 = HEIGHT,
                               Wt.W4 = MASS,
                               D3 = c(mean(INSDEPDs[2:4]), rep(1e-10, 3)),
                               Maximum.SR = 1000 / 60 / AREA,
                               G_m.G2s = G_m.G2s * 1.15,
                               CLO.C4 = clo,
                               CLO.mode = CLO.mode,
                               T_clo.T9s = HomoTherm.out_shorts$balance$T_CLO,
```

```
                                  a_skn.B4 = REFLDs[1],
                                  a_clo.B5 = REFLDs[1],
                                  TAs = env.out$TAs,
                                  TSKYs = env.out$TSKYs,
                                  TGNDs = env.out$TGRDs,
                                  RH.H2s = env.out$RHs / 100,
                                  Q_hs = Q_hs,
                                  q_hs = q_hs,
                                  Zs = env.out$Zs,
                                  VELs = env.out$VELs,
                                  a.B3 = micro$REF)
G_m.G2s <- HomoTherm.out_shorts_chamber$balance$QMETAB / AREA
#G_m.G2s <- rep(G_m.G2, length(env.out$TAs))
MANMO.out_shorts_chamber <- run.MANMO(W = rep(1 / 100, length(env.out$TAs)),
                                  Ht.H4 = HEIGHT,
                                  Wt.W4 = MASS,
                                  D3 = c(mean(INSDEPDs[2:4]), rep(1e-10, 3)),
                                  Maximum.SR = 1000 / 60 / AREA,
                                  G_m.G2s = G_m.G2s * 1.15,
                                  CLO.C4 = clo,
                                  CLO.mode = CLO.mode,
                                  T_clo.T9s = HomoTherm.out_shorts_chamber$balance$T_CLO,
                                  a_skn.B4 = REFLDs[1],
                                  a_clo.B5 = REFLDs[1],
                                  TAs = env.out.chamber$TAs,
                                  TSKYs = env.out.chamber$TSKYs,
                                  TGNDs = env.out.chamber$TGRDs,
                                  RH.H2s = env.out.chamber$RHs / 100,
                                  Q_hs = Q_hs * 0,
                                  q_hs = q_hs * 0,
                                  Zs = env.out.chamber$Zs,
                                  VELs = env.out.chamber$VELs,
                                  a.B3 = micro$REFL)

# Iso7933
Iso7933.out <- lapply(1:length(env.out$TAs),
                           function(x){
                             calcIso7933_Tcl(accl = 100,
                                             Duration = 60 * duration,
                                             posture = 2,
                                             Ta = env.out$TAs[x],
                                             Pa = WETAIR(db = env.out$TAs[x],
                                                         rh = env.out$RHs[x])$e
                                             / 1000,
                                             Tr = (env.out$TGRDs[x] +
                                                     env.out$TSKYs[x]) / 2 +
                                               (env.out$QSOLR[x] / 1366) * 30,
                                             Va = env.out$VELs[x],
                                             Tsk = env.out$TAs[x],
                                             Met = QMETAB_REST / AREA,
                                             Icl = clo,
                                             weight = MASS,
                                             height = HEIGHT / 100,
```

```
                                                  Adu = AREA,
                                                  Tre = 36.8,
                                                  Tcr = 36.8,
                                                  SWp = 0.5
                                )})
Iso7933.out_shorts <- as.data.frame(do.call(rbind, Iso7933.out))


# Iso7933
Iso7933.out <- lapply(1:length(env.out$TAs),
                           function(x){
                             calcIso7933_Tcl(accl = 100,
                                             Duration = 60 * duration,
                                             posture = 2,
                                             Ta = env.out.chamber$TAs[x],
                                             Pa = WETAIR(db =
                                                  env.out.chamber$TAs[x],
                                                  rh = env.out.chamber$RHs[x])$e
                                             / 1000,
                                             Tr = (env.out.chamber$TGRDs[x]
                                               + env.out.chamber$TSKYs[x]) / 2 +
                                               (env.out.chamber$QSOLR[x] / 1366)
                                             * 30,
                                             Va = env.out.chamber$VELs[x],
                                             Tsk = env.out.chamber$TAs[x],
                                             Met = QMETAB_REST / AREA,
                                             Icl = clo,
                                             weight = MASS,
                                             height = HEIGHT / 100,
                                             Adu = AREA,
                                             Tre = 36.8,
                                             Tcr = 36.8,
                                             SWp = 0.5
                                )})
Iso7933.out_shorts_chamber <- as.data.frame(do.call(rbind, Iso7933.out))
```

Plot results.

```
plot.output(HomoTherm.out_shorts, MANMO.out_shorts, Iso7933.out_shorts, env.out)
```

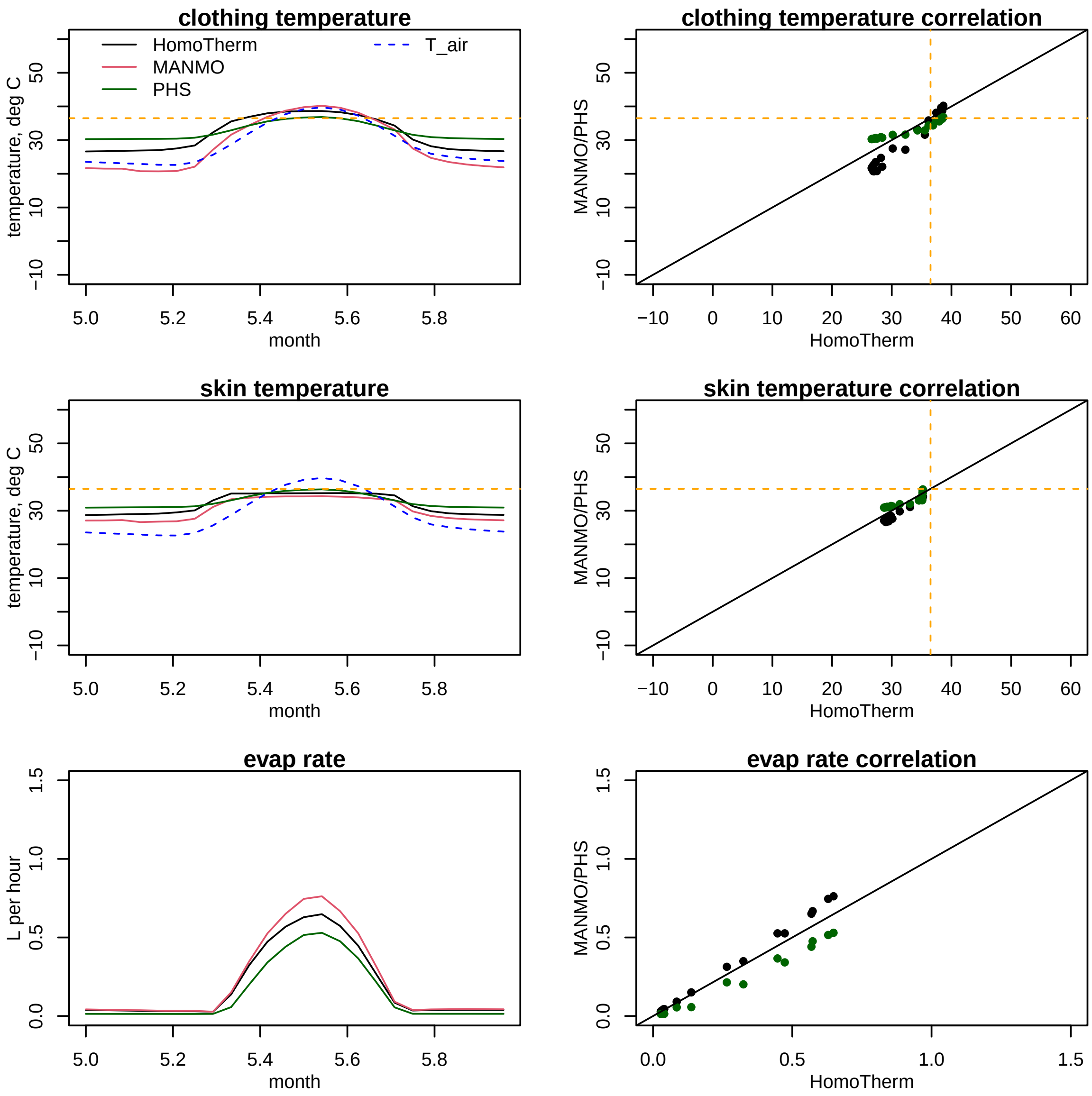


```
plot.balance(HomoTherm.out_shorts, MANMO.out_shorts)
```

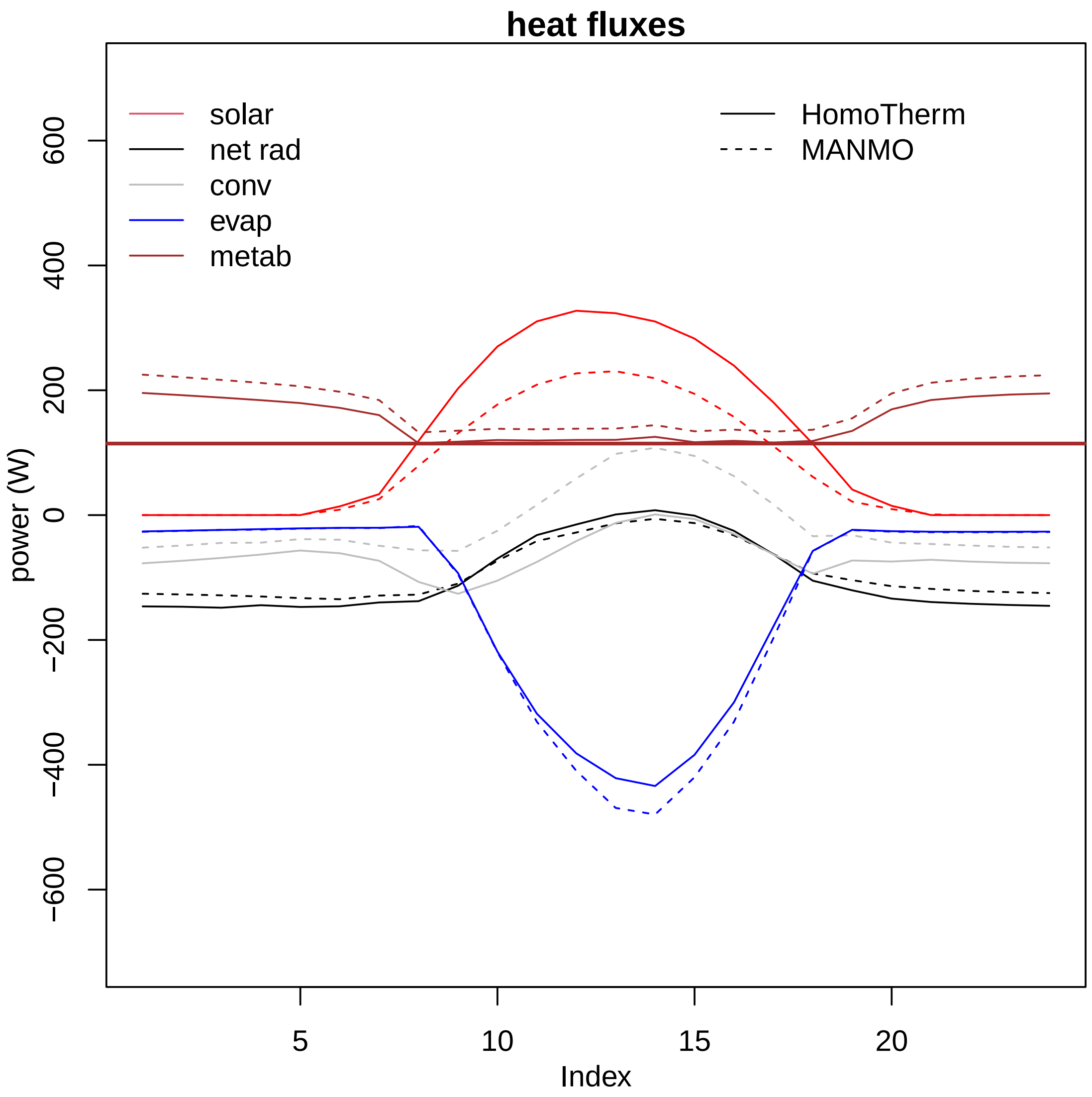


```
plot.output(HomoTherm.out_shorts_chamber, MANMO.out_shorts_chamber, Iso7933.out_shorts_chamber, env.out
```

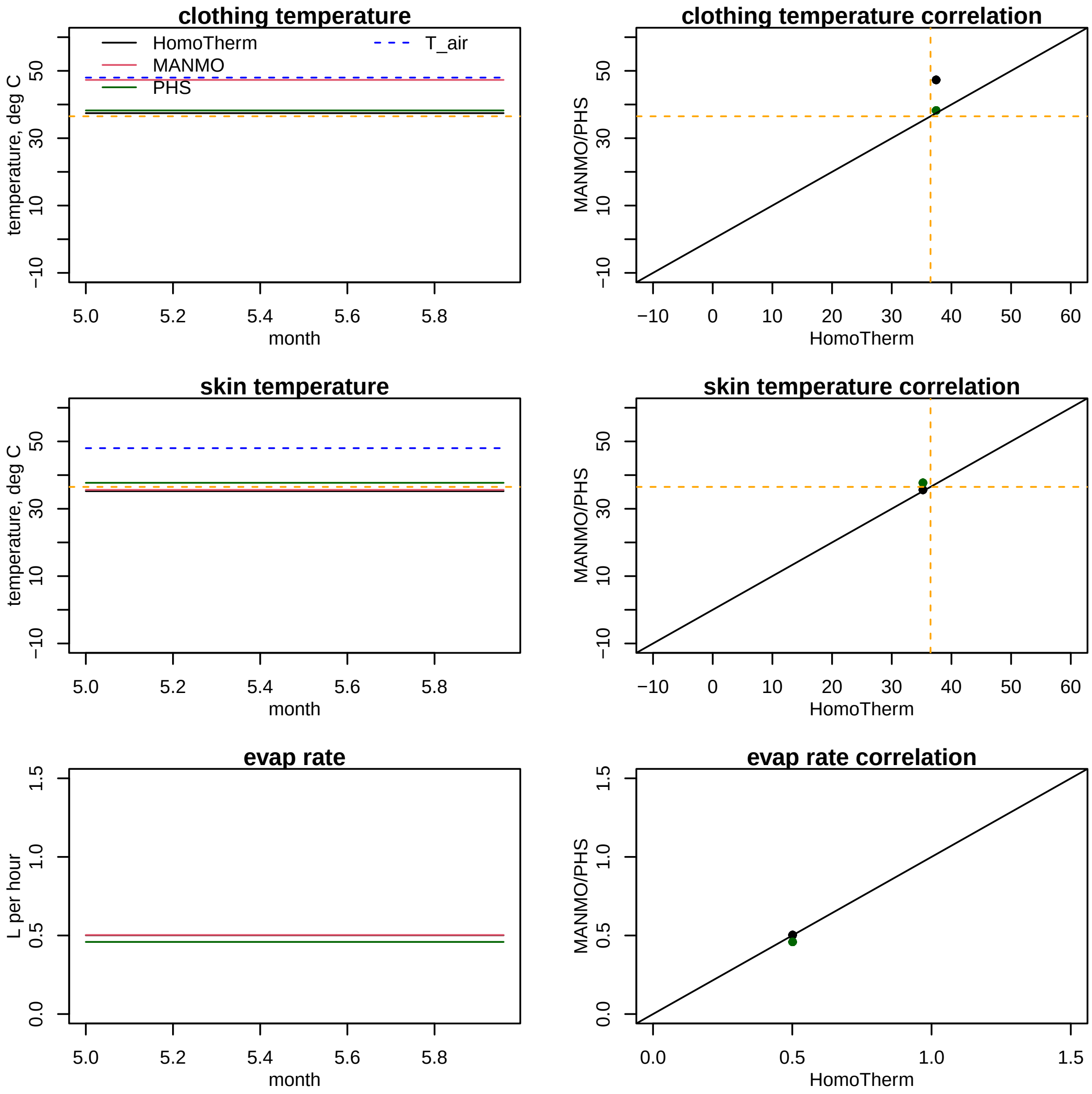


```
plot.balance(HomoTherm.out_shorts_chamber, MANMO.out_shorts_chamber)
```

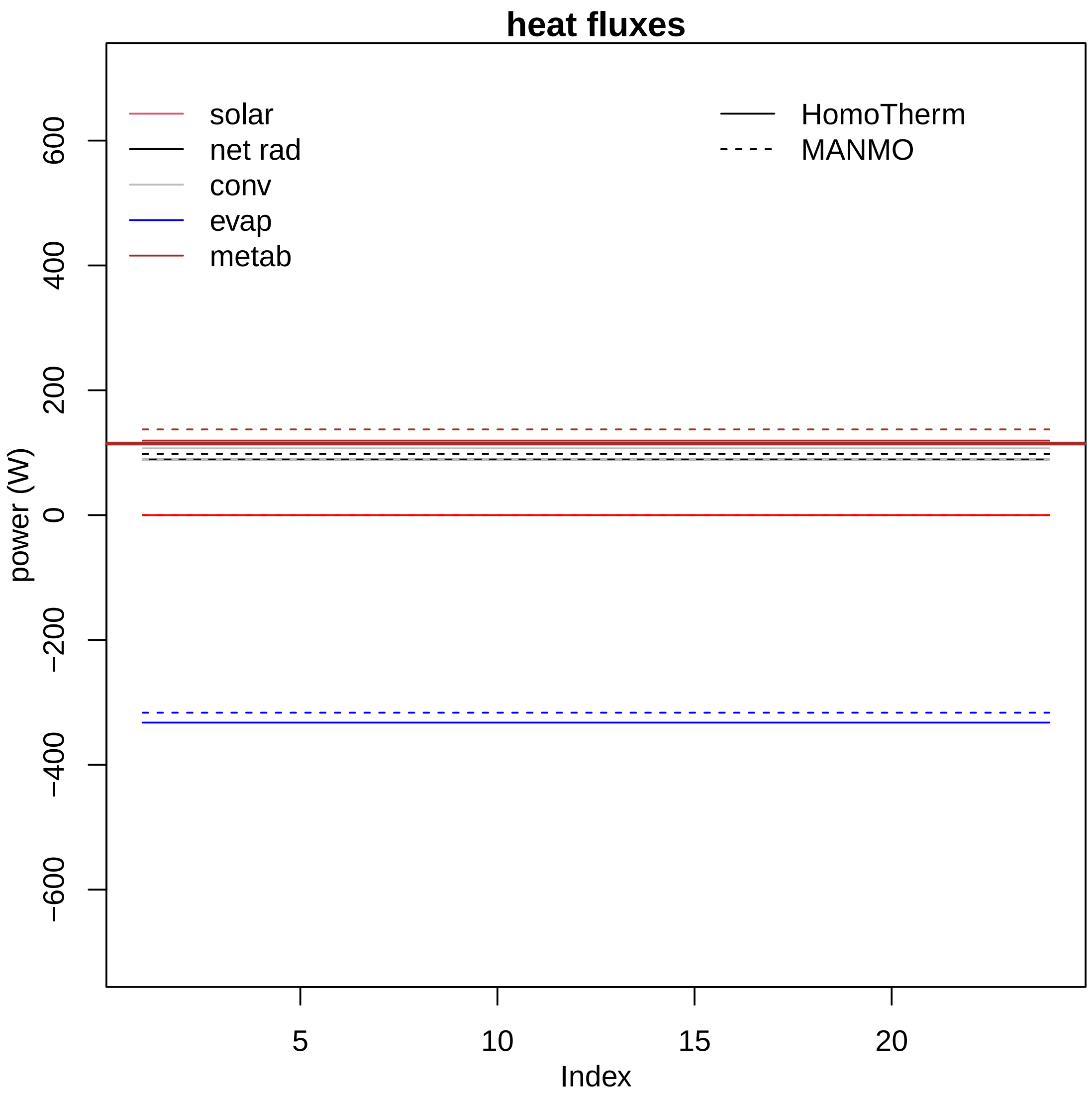

Tan army uniform

Finally, simulate the tan army uniform scenario of Shkolnik et al. 1980.

Set parameters - 'uniform' depth of clothing at 2mm thick.

```
REFLDs <- rep(1 - 0.72, 4) # clothing/skin dorsal reflectivity dec %
REFLVs <- REFLDs # clothing/skin ventral reflectivity dec %
a_clo.B5 <- REFLDs[1] # albedo
PCTBAREVAPs <- c(50, 10, 20, 10)
#KCLOs <- rep(0, 4) # manual override of fur thermal conductivity
INSDEPDs <- c(0.01, 0.002, 0.002, 0.002) # fur depth, dorsal (m)
INSDEPVs <- c(1e-9, 0.002, 0.002, 0.002) # fur depth, ventral (m)
```

```r
par(mfrow = c(1, 1))
plot_human(MASS = MASS,
           HEIGHT = HEIGHT,
           INSDEPDs = INSDEPDs,
           INSDEPVs = INSDEPVs,
           SHAPE_Bs = SHAPE_Bs)
```

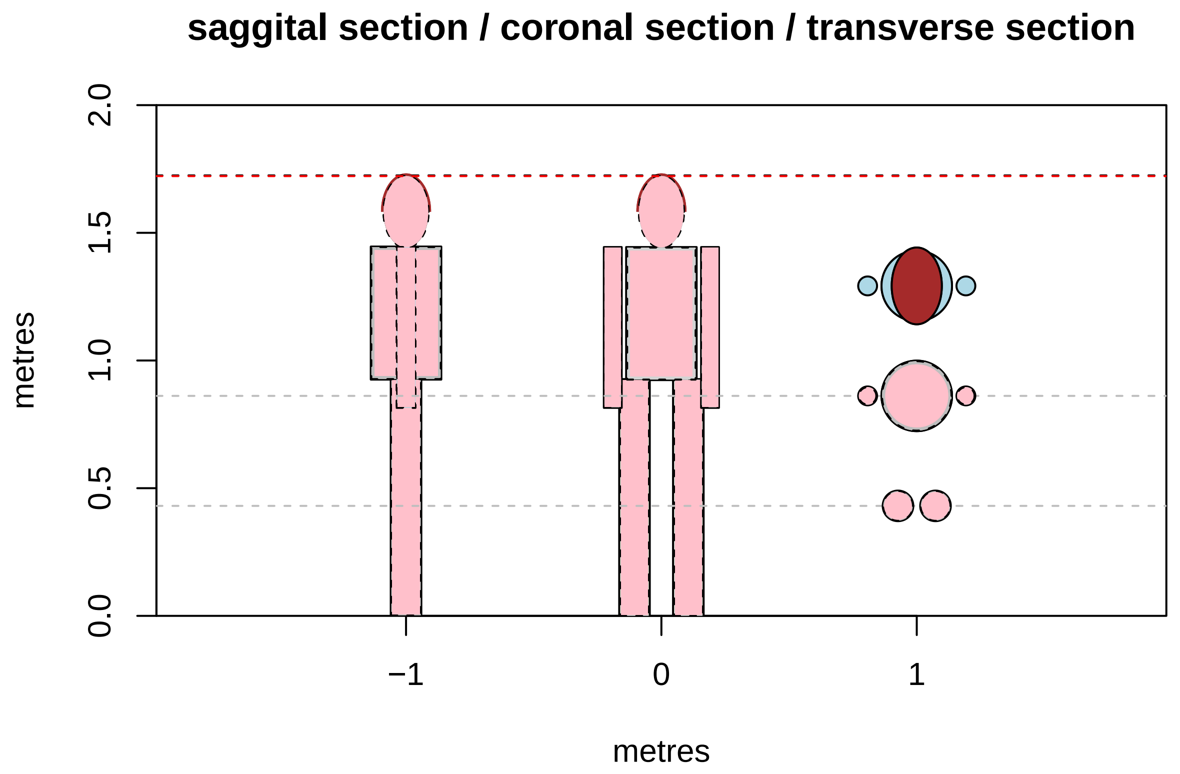


```
## [1] 1.724834
```

Run simulations.

```r
HomoTherm.out_army <- HomoTherm_var(MASS = MASS,
                                    QMETAB_REST = QMETAB_REST,
                                    INSDEPDs = INSDEPDs,
                                    INSDEPVs = INSDEPVs,
                                    SHAPE_Bs = SHAPE_Bs,
                                    PJOINs = PJOINs,
                                    PCTBAREVAPs = PCTBAREVAPs,
                                    REFLDs = REFLDs,
                                    REFLVs = REFLVs,
                                    heights = heights,
                                    REFHYT = REFHYT,
                                    RUF = RUF,
                                    TAs = env.out$TAs,
```

```
                                    TAREFs = env.out$TAREFs,
                                    TSKYs = env.out$TSKYs,
                                    TGRDs = env.out$TGRDs,
                                    RHs = env.out$RHs,
                                    RHREFs = env.out$RHREFs,
                                    VELs = env.out$VELs,
                                    VREFs = env.out$VREFs,
                                    QSOLRs = env.out$QSOLRs,
                                    Zs = env.out$Zs,
                                    PDIFs = env.out$PDIFs,
                                    ELEV = micro$elev,
                                    ABSSB = 1 - micro$REFL,
                                    CONV_ENHANCE = env.out$CONV_ENHANCE)
HomoTherm.out_army_chamber <- HomoTherm_var(MASS = MASS,
                                            QMETAB_REST = QMETAB_REST,
                                            INSDEPDs = INSDEPDs,
                                            INSDEPVs = INSDEPVs,
                                            SHAPE_Bs = SHAPE_Bs,
                                            PJOINs = PJOINs,
                                            PCTBAREVAPs = PCTBAREVAPs,
                                            REFLDs = REFLDs,
                                            REFLVs = REFLVs,
                                            FSKREFs = FSKREFs,
                                            FGDREFs = FGDREFs,
                                            heights = heights,
                                            REFHYT = REFHYT,
                                            RUF = RUF,
                                            TAs = env.out.chamber$TAs,
                                            TAREFs = env.out.chamber$TAREFs,
                                            TSKYs = env.out.chamber$TSKYs,
                                            TGRDs = env.out.chamber$TGRDs,
                                            RHs = env.out.chamber$RHs,
                                            RHREFs = env.out.chamber$RHREFs,
                                            VELs = env.out.chamber$VELs,
                                            VREFs = env.out.chamber$VREFs,
                                            QSOLRs = env.out.chamber$QSOLRs,
                                            Zs = env.out.chamber$Zs,
                                            PDIFs = env.out.chamber$PDIFs,
                                            ELEV = micro$elev,
                                            ABSSB = 1 - micro$REFL,
                                            CONV_ENHANCE = env.out.chamber$CONV_ENHANCE)
clo <- colMeans(get_clo(HomoTherm.out_army,
                        INSDEPDs = INSDEPDs,
                        INSDEPVs = INSDEPVs))
G_m.G2s <- HomoTherm.out_army$balance$QMETAB / AREA
#G_m.G2s <- rep(G_m.G2, length(env.out$TAs))
MANMO.out_army <- run.MANMO(W = rep(1 / 100, length(env.out$TAs)),
                            Ht.H4 = HEIGHT,
                            Wt.W4 = MASS,
                            D3 = c(mean(INSDEPDs[2:4]), rep(1e-10, 3)),
                            Maximum.SR = 1000 / 60 / AREA,
                            G_m.G2s = G_m.G2s * 1.15,
                            CLO.C4 = clo,
```

```
                            CLO.mode = CLO.mode,
                            T_clo.T9s = HomoTherm.out_army$balance$T_CLO,
                            a_skn.B4 = REFLDs[1],
                            a_clo.B5 = REFLDs[1],
                            TAs = env.out$TAs,
                            TSKYs = env.out$TSKYs,
                            TGNDs = env.out$TGRDs,
                            RH.H2s = env.out$RHs / 100,
                            Q_hs = Q_hs,
                            q_hs = q_hs,
                            Zs = env.out$Zs,
                            VELs = env.out$VELs,
                            a.B3 = micro$REFL)
G_m.G2s <- HomoTherm.out_army_chamber$balance$QMETAB / AREA
#G_m.G2s <- rep(G_m.G2, length(env.out$TAs))
MANMO.out_army_chamber <- run.MANMO(W = rep(1 / 100, length(env.out$TAs)),
                                    Ht.H4 = HEIGHT,
                                    Wt.W4 = MASS,
                                    D3 = c(mean(INSDEPDs[2:4]), rep(1e-10, 3)),
                                    Maximum.SR = 1000 / 60 / AREA,
                                    G_m.G2s = G_m.G2s * 1.15,
                                    CLO.C4 = clo,
                                    CLO.mode = CLO.mode,
                                    T_clo.T9s = HomoTherm.out_army_chamber$balance$T_CLO,
                                    a_skn.B4 = REFLDs[1],
                                    a_clo.B5 = REFLDs[1],
                                    TAs = env.out.chamber$TAs,
                                    TSKYs = env.out.chamber$TSKYs,
                                    TGNDs = env.out.chamber$TGRDs,
                                    RH.H2s = env.out.chamber$RHs / 100,
                                    Q_hs = Q_hs * 0,
                                    q_hs = q_hs * 0,
                                    Zs = env.out.chamber$Zs,
                                    VELs = env.out.chamber$VELs,
                                    a.B3 = micro$REFL)

# Iso7933
Iso7933.out <- lapply(1:length(env.out$TAs),
                      function(x){
                        calcIso7933_Tcl(accl = 100,
                                        Duration = 60 * duration,
                                        posture = 2,
                                        Ta = env.out$TAs[x],
                                        Pa = WETAIR(db = env.out$TAs[x],
                                                    rh = env.out$RHs[x])$e
                                        / 1000,
                                        Tr = (env.out$TGRDs[x] +
                                                env.out$TSKYs[x]) / 2 +
                                          (env.out$QSOLR[x] / 1366) * 30,
                                        Va = env.out$VELs[x],
                                        Tsk = env.out$TAs[x],
                                        Met = QMETAB_REST / AREA,
                                        Icl = clo,
```

```
                                              weight = MASS,
                                              height = HEIGHT / 100,
                                              Adu = AREA,
                                              Tre = 36.8,
                                              Tcr = 36.8,
                                              SWp = 0.5
                              )})
Iso7933.out_army <- as.data.frame(do.call(rbind, Iso7933.out))


# Iso7933
Iso7933.out <- lapply(1:length(env.out$TAs),
                            function(x){
                              calcIso7933_Tcl(accl = 100,
                                              Duration = 60 * duration,
                                              posture = 2,
                                              Ta = env.out.chamber$TAs[x],
                                              Pa = WETAIR(db =
                                                      env.out.chamber$TAs[x],
                                                       rh = env.out.chamber$RHs[x])$e
                                              / 1000,
                                              Tr = (env.out.chamber$TGRDs[x]
                                                    + env.out.chamber$TSKYs[x]) / 2
                                              + (env.out.chamber$QSOLR[x] / 1366)
                                              * 30,
                                              Va = env.out.chamber$VELs[x],
                                              Tsk = env.out.chamber$TAs[x],
                                              Met = QMETAB_REST / AREA,
                                              Icl = clo,
                                              weight = MASS,
                                              height = HEIGHT / 100,
                                              Adu = AREA,
                                              Tre = 36.8,
                                              Tcr = 36.8,
                                              SWp = 0.5
                              )})
Iso7933.out_army_chamber <- as.data.frame(do.call(rbind, Iso7933.out))
```

Plot results.

```
plot.output(HomoTherm.out_army, MANMO.out_army, Iso7933.out_army, env.out)
```

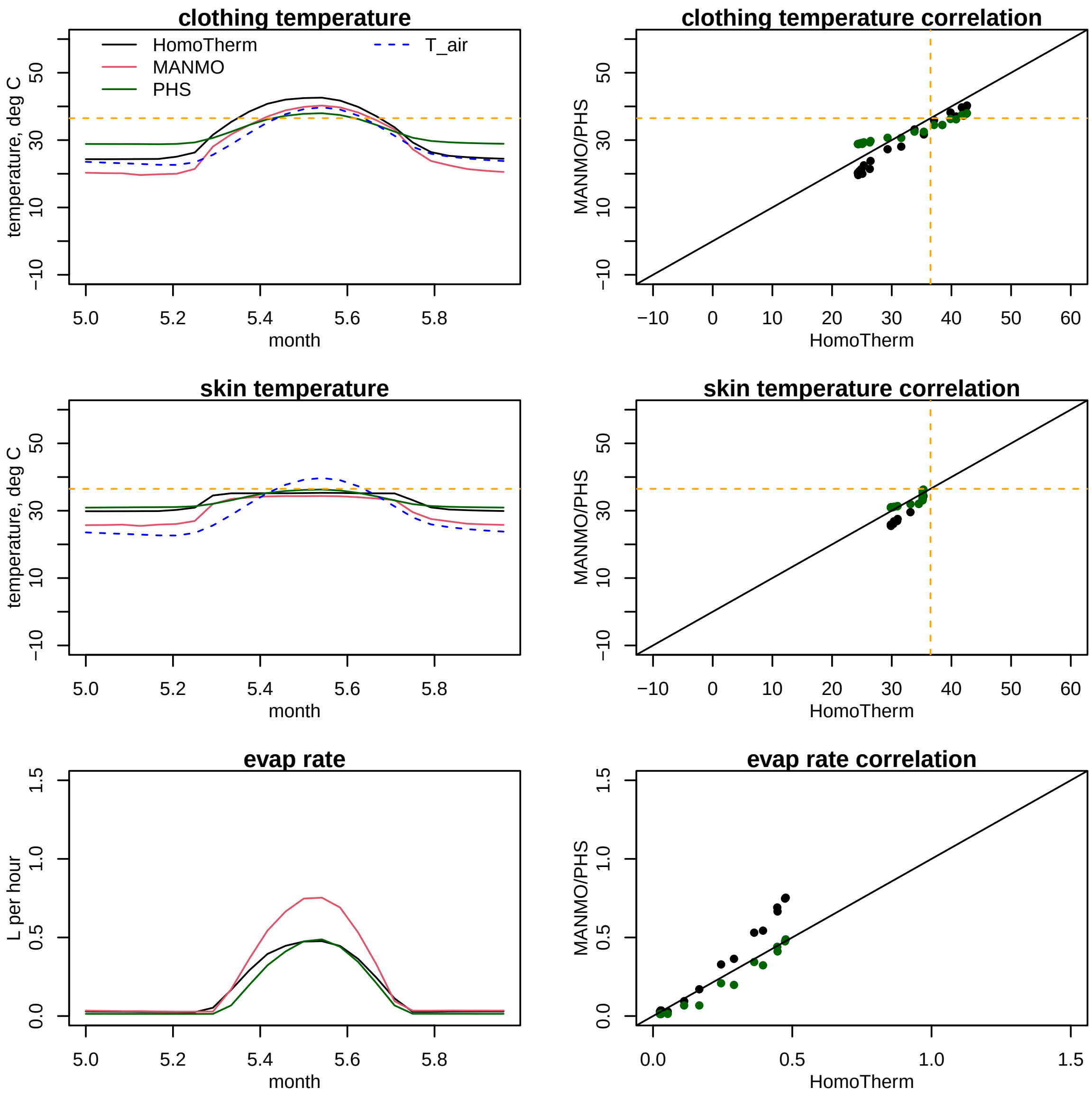


```
plot.balance(HomoTherm.out_army, MANMO.out_army)
```

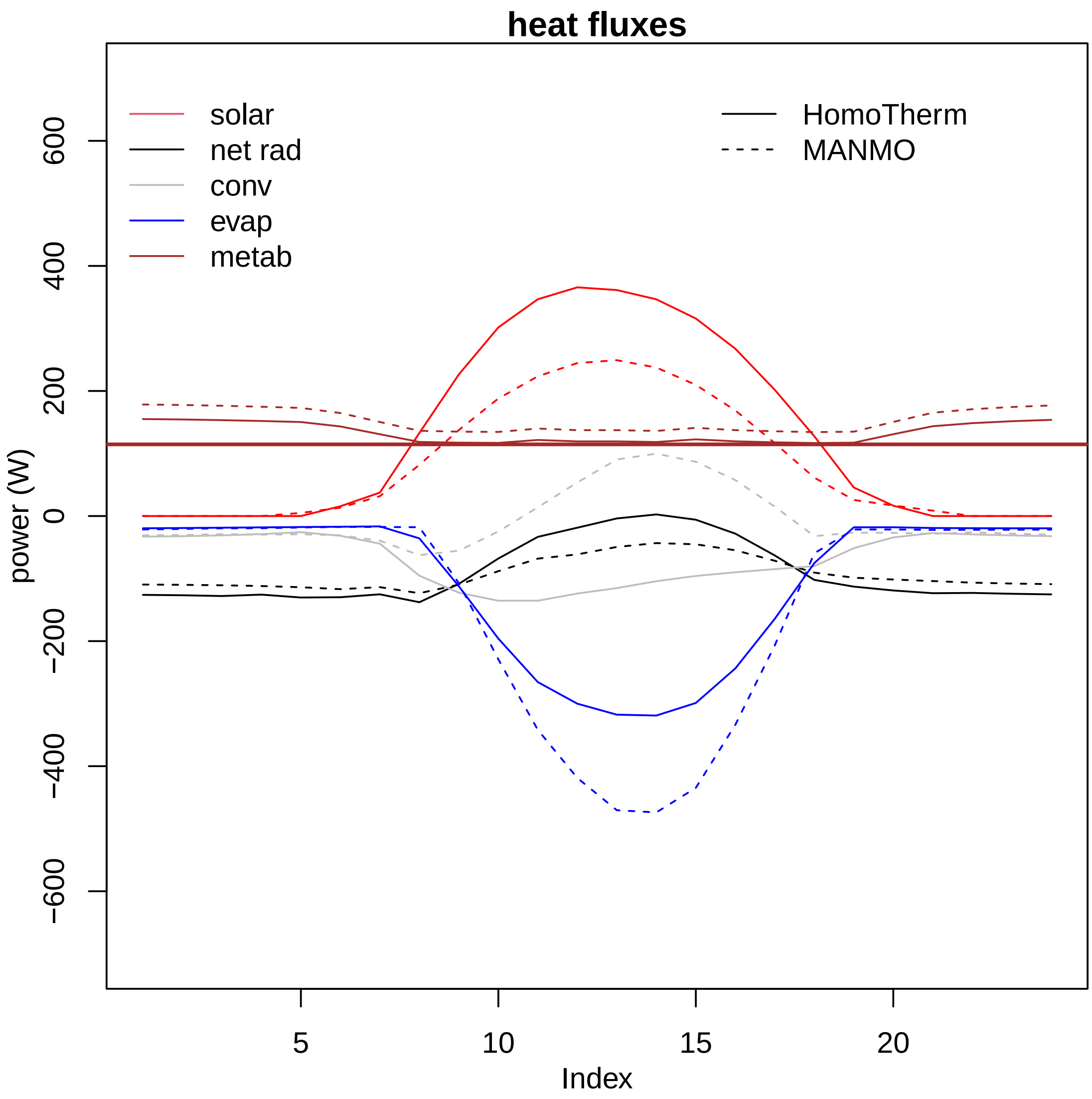


```
plot.output(HomoTherm.out_army_chamber, MANMO.out_army_chamber, Iso7933.out_army_chamber,  env.out.cham
```

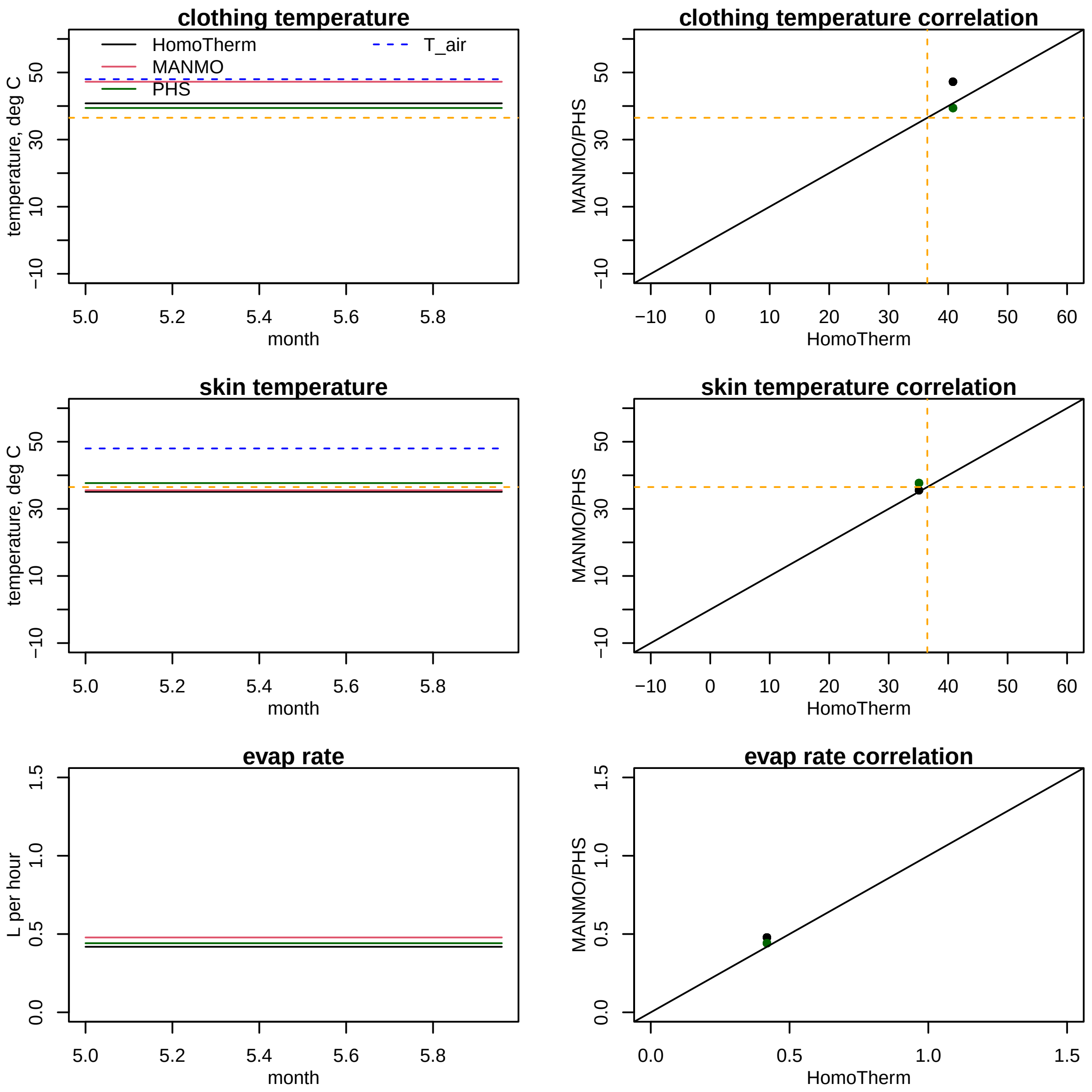


```r
plot.balance(HomoTherm.out_army_chamber, MANMO.out_army_chamber)
```

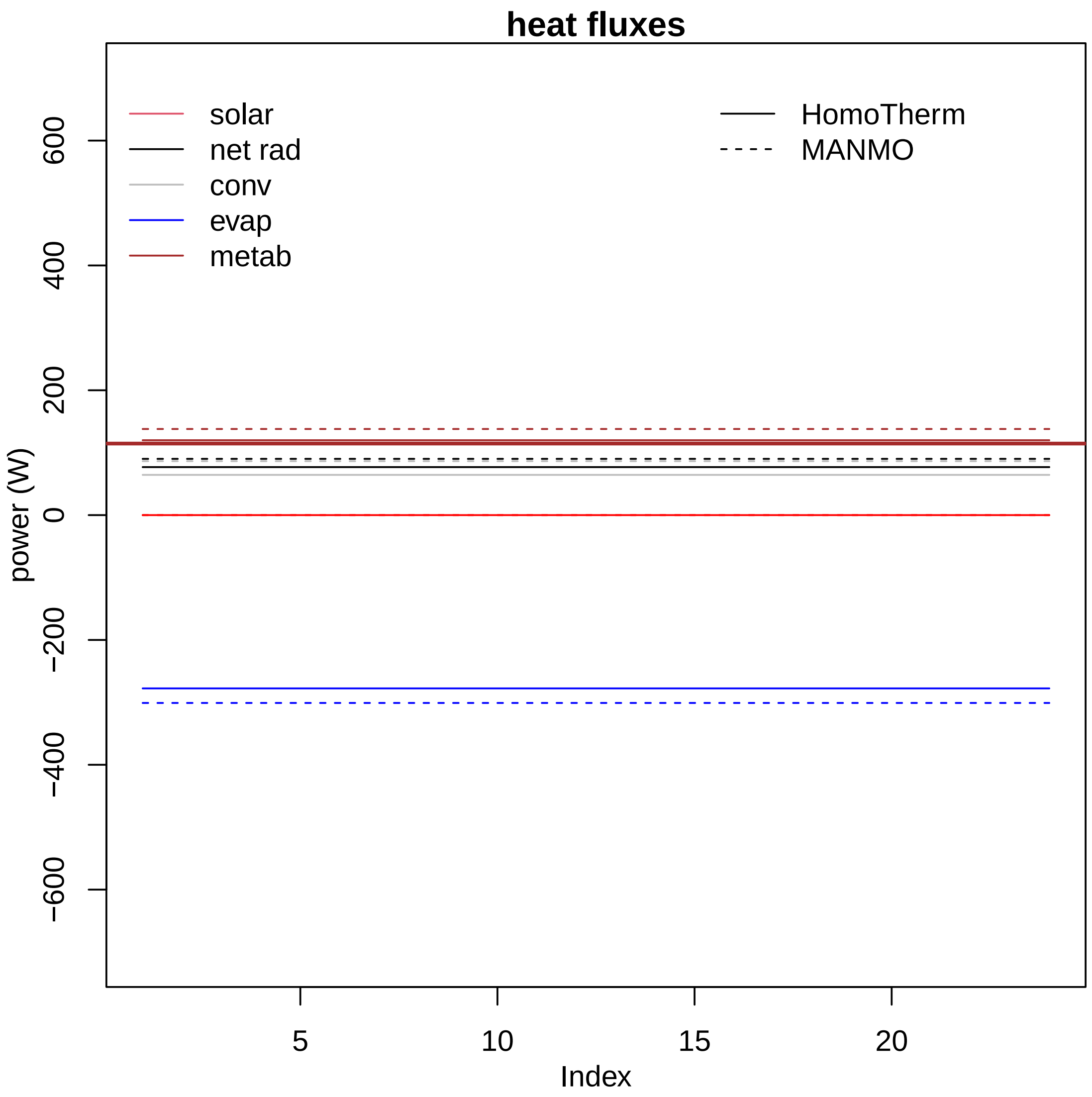

Summarise all results

First, get balance output tables from HomoTherm simulation.

```
HomoTherm_black <- HomoTherm.out_black$balance
HomoTherm_white <- HomoTherm.out_white$balance
HomoTherm_shorts <- HomoTherm.out_shorts$balance
HomoTherm_army <- HomoTherm.out_army$balance
HomoTherm_white_chamber <- HomoTherm.out_white_chamber$balance
HomoTherm_black_chamber <- HomoTherm.out_black_chamber$balance
HomoTherm_shorts_chamber <- HomoTherm.out_shorts_chamber$balance
```

Now summarise results for a chosen hour to compare with observations.

```r
hour <- 12 # 12 = 1 pm (starts at zero)

# divide HomoTherm energy fluxes by surface area
AREA.robe <- HomoTherm.out_black$balance$AREA[1]
AREA.shorts <- HomoTherm.out_shorts$balance$AREA[1]
AREA.army <- HomoTherm.out_army$balance$AREA[1]
pred.black <- HomoTherm_black[hour, ] / c(rep(1, 9), rep(AREA.robe, 8), rep(1, 2))
pred.white <- HomoTherm_white[hour, ] / c(rep(1, 9), rep(AREA.robe, 8), rep(1, 2))
pred.shorts <- HomoTherm_shorts[hour, ] / c(rep(1, 9), rep(AREA.shorts, 8),
                                            rep(1, 2))
pred.army <- HomoTherm_army[hour, ] / c(rep(1, 9), rep(AREA.army, 8), rep(1, 2))
pred.shorts_chamber <- HomoTherm_shorts_chamber[hour, ] /
  c(rep(1, 9), rep(AREA.shorts, 8), rep(1, 2))
pred.white_chamber <- HomoTherm_white_chamber[hour, ] /
  c(rep(1, 9), rep(AREA.robe, 8), rep(1, 2))
pred.black_chamber <- HomoTherm_black_chamber[hour, ] /
  c(rep(1, 9), rep(AREA.robe, 8), rep(1, 2))
pred.env <- metout[range, ][hour, ]
pred.soil <- soil[range, ][hour, ]

# get total longwave in for MANMO
MANMO.out_black$R_in <- MANMO.out_black$R_munc.R1 + MANMO.out_black$R_clo.I8
MANMO.out_white$R_in <- MANMO.out_white$R_munc.R1 + MANMO.out_white$R_clo.I8
MANMO.out_shorts$R_in <- MANMO.out_shorts$R_munc.R1 + MANMO.out_shorts$R_clo.I8
MANMO.out_army$R_in <- MANMO.out_army$R_munc.R1 + MANMO.out_army$R_clo.I8
MANMO.out_shorts_chamber$R_in <- MANMO.out_shorts_chamber$R_munc.R1 + MANMO.out_shorts_chamber$R_clo.I8
MANMO.out_white_chamber$R_in <- MANMO.out_white_chamber$R_munc.R1 + MANMO.out_white_chamber$R_clo.I8
MANMO.out_black_chamber$R_in <- MANMO.out_black_chamber$R_munc.R1 + MANMO.out_black_chamber$R_clo.I8

# divide energy fluxes by surface area
MANMO.out_pred.black <- MANMO.out_black[hour,
  c(1, 1, 1, 56, 2, 1, 64, 64, 63, 12, 65, 24, 23, 25, 15, 15, 16, 1, 1)] /
  c(rep(1, 9), rep(MANMO.out_black$A.A2[1], 7), rep(1, 1))
MANMO.out_pred.white <- MANMO.out_white[hour,
  c(1, 1, 1, 56, 2, 1, 64, 64, 63, 12, 65, 24, 23, 25, 15, 15, 16, 1, 1)] /
  c(rep(1, 9), rep(MANMO.out_white$A.A2[1], 7), rep(1, 1))
MANMO.out_pred.shorts <- MANMO.out_shorts[hour,
  c(1, 1, 1, 56, 2, 1, 64, 64, 63, 12, 65, 24, 23, 25, 15, 15, 16, 1, 1)] /
  c(rep(1, 9), rep(MANMO.out_shorts$A.A2[1], 7), rep(1, 1))
MANMO.out_pred.army <- MANMO.out_army[hour,
  c(1, 1, 1, 56, 2, 1, 64, 64, 63, 12, 65, 24, 23, 25, 15, 15, 16, 1, 1)] /
  c(rep(1, 9), rep(MANMO.out_army$A.A2[1], 7), rep(1, 1))
MANMO.out_pred.shorts_chamber <- MANMO.out_shorts_chamber[hour,
  c(1, 1, 1, 56, 2, 1, 64, 64, 63, 12, 65, 24, 23, 25, 15, 15, 16, 1, 1)] /
  c(rep(1, 9), rep(MANMO.out_shorts_chamber$A.A2[1], 7), rep(1, 1))
MANMO.out_pred.white_chamber <- MANMO.out_white_chamber[hour,
  c(1, 1, 1, 56, 2, 1, 64, 64, 63, 12, 65, 24, 23, 25, 15, 15, 16, 1, 1)] /
  c(rep(1, 9), rep(MANMO.out_white_chamber$A.A2[1], 7), rep(1, 1))
MANMO.out_pred.black_chamber <- MANMO.out_black_chamber[hour,
  c(1, 1, 1, 56, 2, 1, 64, 64, 63, 12, 65, 24, 23, 25, 15, 15, 16, 1, 1)] /
  c(rep(1, 9), rep(MANMO.out_black_chamber$A.A2[1], 7), rep(1, 1))

# make MANMO column names same as HomoTherm column names
```

```
colnames(MANMO.out_pred.black) <- colnames(pred.black)
colnames(MANMO.out_pred.white) <- colnames(pred.black)
colnames(MANMO.out_pred.shorts) <- colnames(pred.black)
colnames(MANMO.out_pred.army) <- colnames(pred.black)
colnames(MANMO.out_pred.shorts_chamber) <- colnames(pred.black)
colnames(MANMO.out_pred.white_chamber) <- colnames(pred.black)
colnames(MANMO.out_pred.black_chamber) <- colnames(pred.black)

# change manmo skin wetness to %
MANMO.out_pred.black$PCTWET <- MANMO.out_pred.black$PCTWET * 100
MANMO.out_pred.white$PCTWET <- MANMO.out_pred.white$PCTWET * 100
MANMO.out_pred.shorts$PCTWET <- MANMO.out_pred.shorts$PCTWET * 100
MANMO.out_pred.army$PCTWET <- MANMO.out_pred.army$PCTWET * 100
MANMO.out_pred.shorts_chamber$PCTWET <- MANMO.out_pred.shorts_chamber$PCTWET * 100
MANMO.out_pred.white_chamber$PCTWET <- MANMO.out_pred.white_chamber$PCTWET * 100
MANMO.out_pred.black_chamber$PCTWET <- MANMO.out_pred.black_chamber$PCTWET * 100

# create a table of predictions to match the observations table
predictions <- obs
predictions[, 3:10] <- NA
# radiation gain
predictions[1, 3] <- pred.black$QSLR + pred.black$QRAD_IN
predictions[2, 3] <- pred.white$QSLR + pred.white$QRAD_IN
predictions[3, 3] <- pred.shorts$QSLR + pred.shorts$QRAD_IN
predictions[4, 3] <- pred.army$QSLR + pred.army$QRAD_IN
predictions[5, 3] <- pred.black_chamber$QSLR + pred.black_chamber$QRAD_IN
predictions[6, 3] <- pred.white_chamber$QSLR + pred.white_chamber$QRAD_IN
predictions[7, 3] <- pred.shorts_chamber$QSLR + pred.shorts_chamber$QRAD_IN
# radiation loss
predictions[1, 4] <- pred.black$QRAD_OUT
predictions[2, 4] <- pred.white$QRAD_OUT
predictions[3, 4] <- pred.shorts$QRAD_OUT
predictions[4, 4] <- pred.army$QRAD_OUT
predictions[5, 4] <- pred.black_chamber$QRAD_OUT
predictions[6, 4] <- pred.white_chamber$QRAD_OUT
predictions[7, 4] <- pred.shorts_chamber$QRAD_OUT
# convective loss
predictions[1, 5] <- pred.black$QCONV + pred.black$QCONV_RESP
predictions[2, 5] <- pred.white$QCONV + pred.white$QCONV_RESP
predictions[3, 5] <- pred.shorts$QCONV + pred.shorts$QCONV_RESP
predictions[4, 5] <- pred.army$QCONV + pred.army$QCONV_RESP
predictions[5, 5] <- pred.black_chamber$QCONV + pred.black_chamber$QCONV_RESP
predictions[6, 5] <- pred.white_chamber$QCONV + pred.white_chamber$QCONV_RESP
predictions[7, 5] <- pred.shorts_chamber$QCONV + pred.shorts_chamber$QCONV_RESP
# evaporative loss
predictions[1, 7] <- pred.black$QEVAP_CUT + pred.black$QEVAP_RESP
predictions[2, 7] <- pred.white$QEVAP_CUT + pred.white$QEVAP_RESP
predictions[3, 7] <- pred.shorts$QEVAP_CUT + pred.shorts$QEVAP_RESP
predictions[4, 7] <- pred.army$QEVAP_CUT + pred.army$QEVAP_RESP
predictions[5, 7] <- pred.black_chamber$QEVAP_CUT + pred.black_chamber$QEVAP_RESP
predictions[6, 7] <- pred.white_chamber$QEVAP_CUT + pred.white_chamber$QEVAP_RESP
predictions[7, 7] <- pred.shorts_chamber$QEVAP_CUT + pred.shorts_chamber$QEVAP_RESP
# metabolic gain
```

```
predictions[1, 9] <- pred.black$QMETAB
predictions[2, 9] <- pred.white$QMETAB
predictions[3, 9] <- pred.shorts$QMETAB
predictions[4, 9] <- pred.army$QMETAB
predictions[5, 9] <- pred.black_chamber$QMETAB
predictions[6, 9] <- pred.white_chamber$QMETAB
predictions[7, 9] <- pred.shorts_chamber$QMETAB
predictions$conv_loss <- predictions$conv_loss * -1
predictions$h_evap <- predictions$h_evap * -1

# heat storage, 3.47 kJ/kg C, Finch et al. 1980 Physiological Zoology 53:19-25
TC_rest <- 36.8 # assumed resting core temp in HomoTherm simulations
predictions[1, 8] <- 3.47 * 1000 / 3600 * MASS * (pred.black$T_CORE - TC_rest) / AREA
predictions[2, 8] <- 3.47 * 1000 / 3600 * MASS * (pred.white$T_CORE - TC_rest) / AREA
predictions[3, 8] <- 3.47 * 1000 / 3600 * MASS * (pred.shorts$T_CORE - TC_rest) / AREA
predictions[4, 8] <- 3.47 * 1000 / 3600 * MASS * (pred.army$T_CORE - TC_rest) / AREA
predictions[5, 8] <- 3.47 * 1000 / 3600 * MASS *
  (pred.black_chamber$T_CORE - TC_rest) / AREA
predictions[6, 8] <- 3.47 * 1000 / 3600 * MASS *
  (pred.white_chamber$T_CORE - TC_rest) / AREA
predictions[7, 8] <- 3.47 * 1000 / 3600 * MASS *
  (pred.shorts_chamber$T_CORE - TC_rest) / AREA

# same for MANMO output
MANMO.out_predictions <- obs
MANMO.out_predictions[, 3:10] <- NA
# radiation gain
MANMO.out_predictions[1, 3] <- MANMO.out_pred.black$QSLR + pred.black$QRAD_IN
MANMO.out_predictions[2, 3] <- MANMO.out_pred.white$QSLR + pred.white$QRAD_IN
MANMO.out_predictions[3, 3] <- MANMO.out_pred.shorts$QSLR + pred.shorts$QRAD_IN
MANMO.out_predictions[4, 3] <- MANMO.out_pred.army$QSLR + pred.army$QRAD_IN
# radiation loss
MANMO.out_predictions[1, 4] <- MANMO.out_pred.black$QRAD_OUT
MANMO.out_predictions[2, 4] <- MANMO.out_pred.white$QRAD_OUT
MANMO.out_predictions[3, 4] <- MANMO.out_pred.shorts$QRAD_OUT
MANMO.out_predictions[4, 4] <- MANMO.out_pred.army$QRAD_OUT
# convective loss
MANMO.out_predictions[1, 5] <- MANMO.out_pred.black$QCONV
MANMO.out_predictions[2, 5] <- MANMO.out_pred.white$QCONV
MANMO.out_predictions[3, 5] <- MANMO.out_pred.shorts$QCONV
MANMO.out_predictions[4, 5] <- MANMO.out_pred.army$QCONV
# evaporative loss
MANMO.out_predictions[1, 7] <- MANMO.out_pred.black$QEVAP_CUT
MANMO.out_predictions[2, 7] <- MANMO.out_pred.white$QEVAP_CUT
MANMO.out_predictions[3, 7] <- MANMO.out_pred.shorts$QEVAP_CUT
MANMO.out_predictions[4, 7] <- MANMO.out_pred.army$QEVAP_CUT
MANMO.out_predictions[5, 7] <- MANMO.out_pred.black_chamber$QEVAP_CUT
MANMO.out_predictions[6, 7] <- MANMO.out_pred.white_chamber$QEVAP_CUT
MANMO.out_predictions[7, 7] <- MANMO.out_pred.shorts_chamber$QEVAP_CUT
# metabolic gain
MANMO.out_predictions[1, 9] <- MANMO.out_pred.black$QMETAB
MANMO.out_predictions[2, 9] <- MANMO.out_pred.white$QMETAB
MANMO.out_predictions[3, 9] <- MANMO.out_pred.shorts$QMETAB
```

```r
MANMO.out_predictions[4, 9] <- MANMO.out_pred.army$QMETAB
MANMO.out_predictions[5, 9] <- MANMO.out_pred.black_chamber$QMETAB
MANMO.out_predictions[6, 9] <- MANMO.out_pred.white_chamber$QMETAB
MANMO.out_predictions[7, 9] <- MANMO.out_pred.shorts_chamber$QMETAB
MANMO.out_predictions$conv_loss <- MANMO.out_predictions$conv_loss * -1
MANMO.out_predictions$h_evap <- MANMO.out_predictions$h_evap * -1

# compute radiant temperature ('ambient') at hour of interest
sigma <- 5.67e-8
sky_rad <- sigma * (metout$TSKYC[range][hour] + 273.15) ^ 4 / 2 # sky radiant
ground_rad <- sigma * (soil$D0cm[range][hour] + 273.15) ^ 4 / 2 # ground radiant
# solar load given absorptivity of clothing and ground
solar_rad_black <- (metout$SOLR[range][hour] + metout$SOLR[range][hour] *
                      micro$REFL) / 2 * 0.89
solar_rad_white <- (metout$SOLR[range][hour] + metout$SOLR[range][hour] *
                      micro$REFL) / 2 * 0.35
solar_rad_army <- (metout$SOLR[range][hour] + metout$SOLR[range][hour] *
                     micro$REFL) / 2 * 0.72
solar_rad_nude <- (metout$SOLR[range][hour] + metout$SOLR[range][hour] *
                     micro$REFL) / 2 * 0.66
# get total radiation as: total_rad <- sky_rad + ground_rad + solar_rad_black
# then conver to teperature via: rad_temp <- (total_rad / sigma) ^ (1 / 4) - 273.15
predictions[1, 10] <- ((sky_rad + ground_rad + solar_rad_black) / sigma) ^
  (1 / 4) - 273.15
predictions[2, 10] <- ((sky_rad + ground_rad + solar_rad_white) / sigma) ^
  (1 / 4) - 273.15
predictions[3, 10] <- ((sky_rad + ground_rad + solar_rad_army) / sigma) ^
  (1 / 4) - 273.15
predictions[4, 10] <- ((sky_rad + ground_rad + solar_rad_nude) / sigma) ^
  (1 / 4) - 273.15
predictions[5:7, 10] <- 48
```

Now plot skin and robe temperatures for the two coloured robes plus associated fluxes.

```r
par(mfrow = c(2, 1))
par(oma = c(2, 1, 1, 1) + 0.1) # margin spacing
par(mar = c(4, 4, 1, 1) + 0.1) # margin spacing
par(mgp = c(2, 1, 0) ) # margin spacing
plot(HomoTherm_black$T_CLO, col = '1', type = 'l', ylim = c(20, 60),
     ylab = 'temperature (°C)', xlab = 'hour of day',
     main = 'skin and robe temperature, June 1978')
points(HomoTherm_white$T_CLO, col = '1', type = 'l', lty = 2)
points(HomoTherm_black$T_SKIN, col = 'orange', type = 'l')
points(HomoTherm_white$T_SKIN, col = 'orange', type = 'l', lty = 2)
legend(0, 60, bty = 'n', legend = c('robe', 'skin'), col = c(1, 'orange'), lty = 1)
legend(17, 60, bty = 'n', legend = c('black', 'white'), col = 1, lty = c(1, 2))

plot(HomoTherm_black$QSLR, col = 'red', type = 'l', ylim = c(-700, 800),
     main = 'heat fluxes', ylab = 'power (W)')
points(HomoTherm_white$QSLR, col = 'red', type = 'l', lty = 2)
points(HomoTherm_black$QRAD_IN - HomoTherm_black$QRAD_OUT, col = '1', type = 'l',
       lty = 2)
points(HomoTherm_white$QRAD_IN - HomoTherm_white$QRAD_OUT, col = '1', type = 'l')
```

```
points(HomoTherm_black$QCONV, col = 'grey', type = 'l')
points(HomoTherm_white$QCONV, col = 'grey', type = 'l', lty = 2)
points(HomoTherm_black$QEVAP_CUT, col = 'blue', type = 'l')
points(HomoTherm_white$QEVAP_CUT, col = 'blue', type = 'l', lty = 2)
points(HomoTherm_black$QMETAB, col = 'brown', type = 'l')
points(HomoTherm_white$QMETAB, col = 'brown', type = 'l', lty = 2)
abline(h = QMETAB_REST, col = 'brown', lwd = 2)
legend(0, 850, bty = 'n', legend = c('solar', 'net rad', 'conv', 'evap', 'metab'),
       col = c(2, 1, 'grey', 'blue', 'brown'), lty = 1, horiz = TRUE, cex = 0.85)
legend(17, 700, bty = 'n', legend = c('black', 'white'), col = 1, lty = c(1, 2))
```

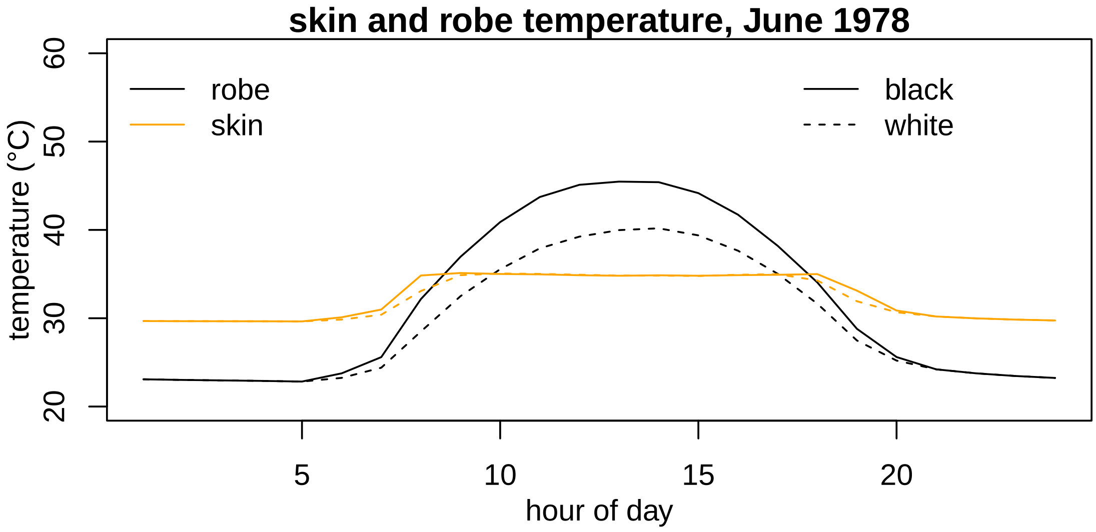

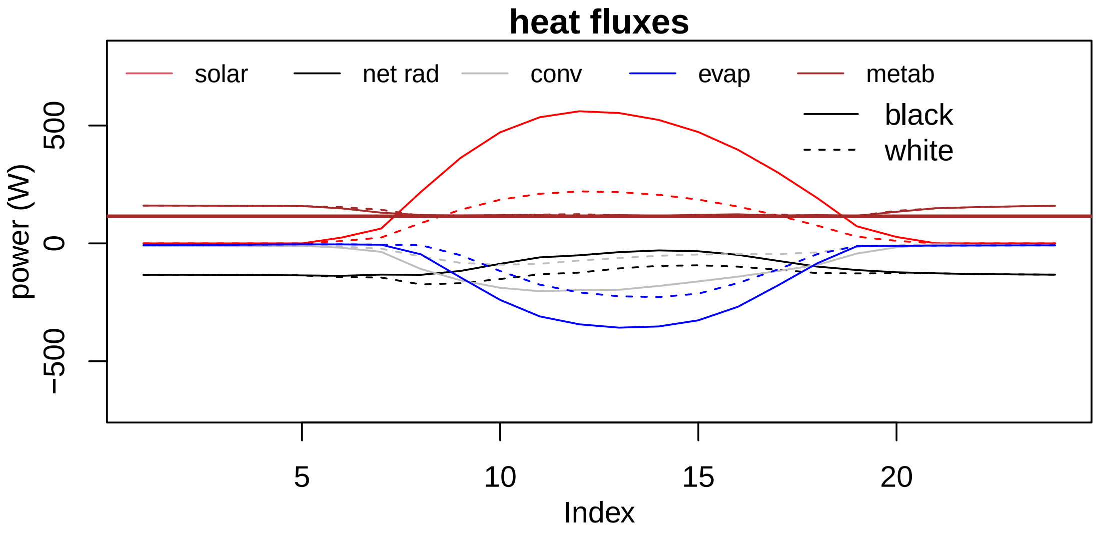

Now plot observations of fluxes against predictions.

```r
par(mfrow = c(1, 1))

# HomoTherm predictions
plot(obs$rad_gain, predictions$rad_gain, pch = 16, ylim = c(-50, 900),
     xlim = c(-50, 900), xlab = 'observed, W/m^2', ylab = 'predicted, W/m^2')
abline(0, 1)
points(obs$rad_loss, predictions$rad_loss, pch = 16, ylim = c(0, 550),
       xlim = c(0, 550), col = 'orange')
points(obs$conv_loss, predictions$conv_loss, pch = 16, ylim = c(0, 280),
       xlim = c(0, 280), col = 'cyan')
points(obs$h_evap, predictions$h_evap, pch = 16, col = 'blue')
points(obs$h_metab, predictions$h_metab, pch = 16, col = 'brown')
points(obs$h_metab[obs$environment == 'chamber'],
       predictions$h_metab[obs$environment == 'chamber'], pch = 1, cex = 2)
points(obs$h_evap[obs$environment == 'chamber'],
       predictions$h_evap[obs$environment == 'chamber'], pch = 1, cex = 2)
points(obs[1, c(3, 4, 5, 7, 9)], predictions[1, c(3, 4, 5, 7, 9)], pch = 3, cex = 2)
points(obs[2, c(3, 4, 5, 7, 9)], predictions[2, c(3, 4, 5, 7, 9)], pch = 2, cex = 2)
points(obs[3, c(3, 4, 5, 7, 9)], predictions[3, c(3, 4, 5, 7, 9)], pch = 0, cex = 2)
points(obs[4, c(3, 4, 5, 7, 9)], predictions[4, c(3, 4, 5, 7, 9)], pch = 4, cex = 2)
legend(0-50, 900, bty = 'n', legend =
         c('rad gain', 'rad loss', 'conv loss', 'h_evap', 'h_metab'),
       col = c('black', 'orange', 'cyan', 'blue', 'brown'), pch = 16)
legend(230-50, 900, bty = 'n', legend =
         c('chamber', 'black robe', 'white robe', 'shorts', 'army uniform'),
       pch = c(1, 3, 2, 0, 4), cex = 1)
```

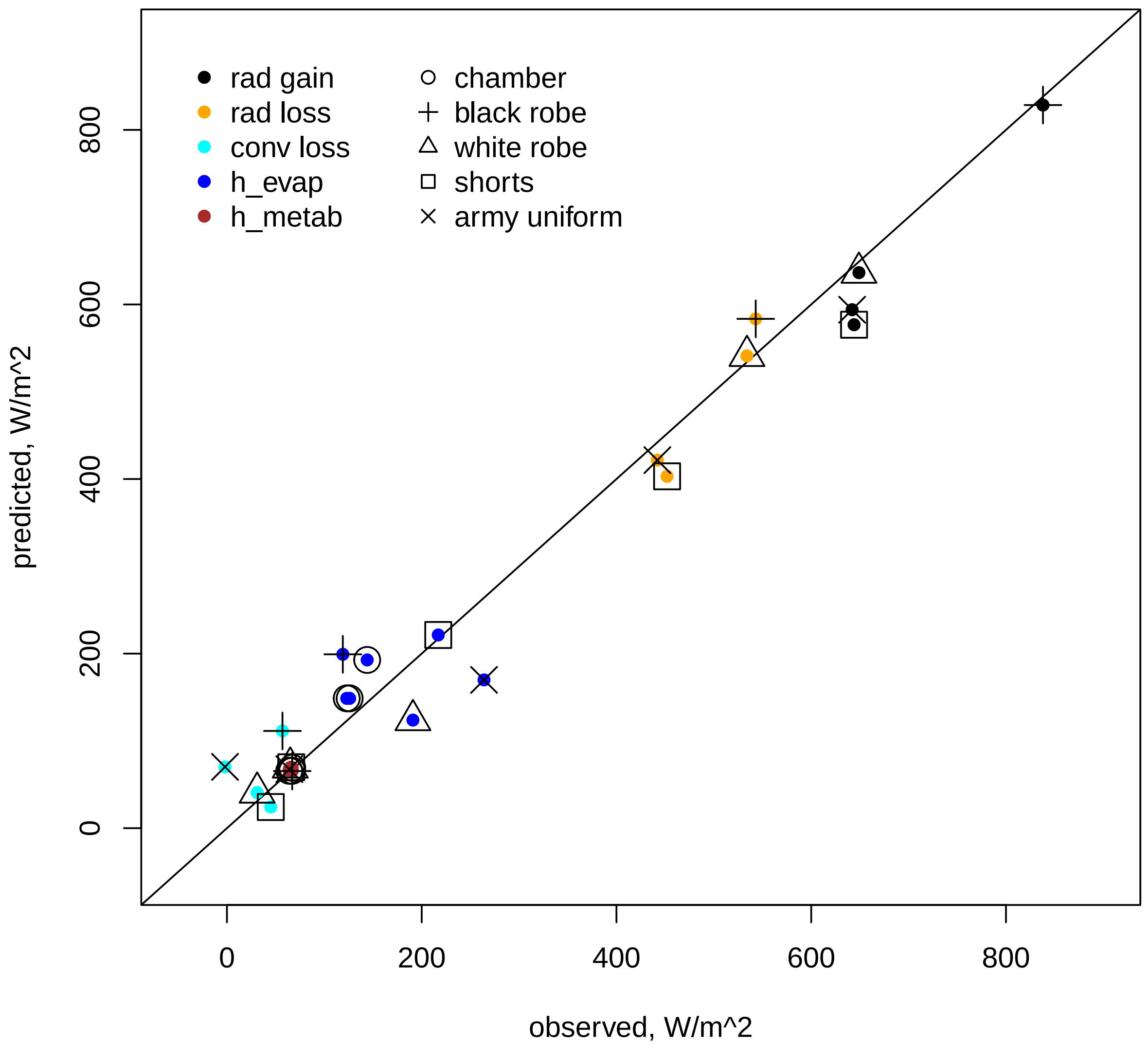


```
# MANMO predictions
plot(obs$rad_gain, MANMO.out_predictions$rad_gain, pch = 16, ylim =
       c(-50, 900), xlim = c(-50, 900), xlab = 'observed, W/m^2',
     ylab = 'predicted, W/m^2')
abline(0, 1)
points(obs$rad_loss, MANMO.out_predictions$rad_loss, pch = 16,
       ylim = c(0, 550), xlim = c(0, 550), col = 'orange')
points(obs$conv_loss, MANMO.out_predictions$conv_loss, pch = 16,
       ylim = c(0, 280), xlim = c(0, 280), col = 'cyan')
points(obs$h_evap, MANMO.out_predictions$h_evap, pch = 16, col = 'blue')
points(obs$h_metab, MANMO.out_predictions$h_metab, pch = 16, col = 'brown')
points(obs$h_metab[obs$environment == 'chamber'], MANMO.out_predictions$h_metab[obs$environment == 'cha
points(obs$h_evap[obs$environment == 'chamber'], MANMO.out_predictions$h_evap[obs$environment == 'chamb
points(obs[1, c(3, 4, 5, 7, 9)], MANMO.out_predictions[1, c(3, 4, 5, 7, 9)],
```

```
       pch = 3, cex = 2)
points(obs[2, c(3, 4, 5, 7, 9)], MANMO.out_predictions[2, c(3, 4, 5, 7, 9)],
       pch = 2, cex = 2)
points(obs[3, c(3, 4, 5, 7, 9)], MANMO.out_predictions[3, c(3, 4, 5, 7, 9)],
       pch = 0, cex = 2)
points(obs[4, c(3, 4, 5, 7, 9)], MANMO.out_predictions[4, c(3, 4, 5, 7, 9)],
       pch = 4, cex = 2)
legend(0-50, 900, bty = 'n', legend =
         c('rad gain', 'rad loss', 'conv loss', 'h_evap', 'h_metab'),
       col = c('black', 'orange', 'cyan', 'blue', 'brown'), pch = 16)
legend(230-50, 900, bty = 'n',
       legend = c('chamber', 'black robe', 'white robe', 'shorts', 'army uniform'),
       pch = c(1, 3, 2, 0, 4), cex = 1)
```

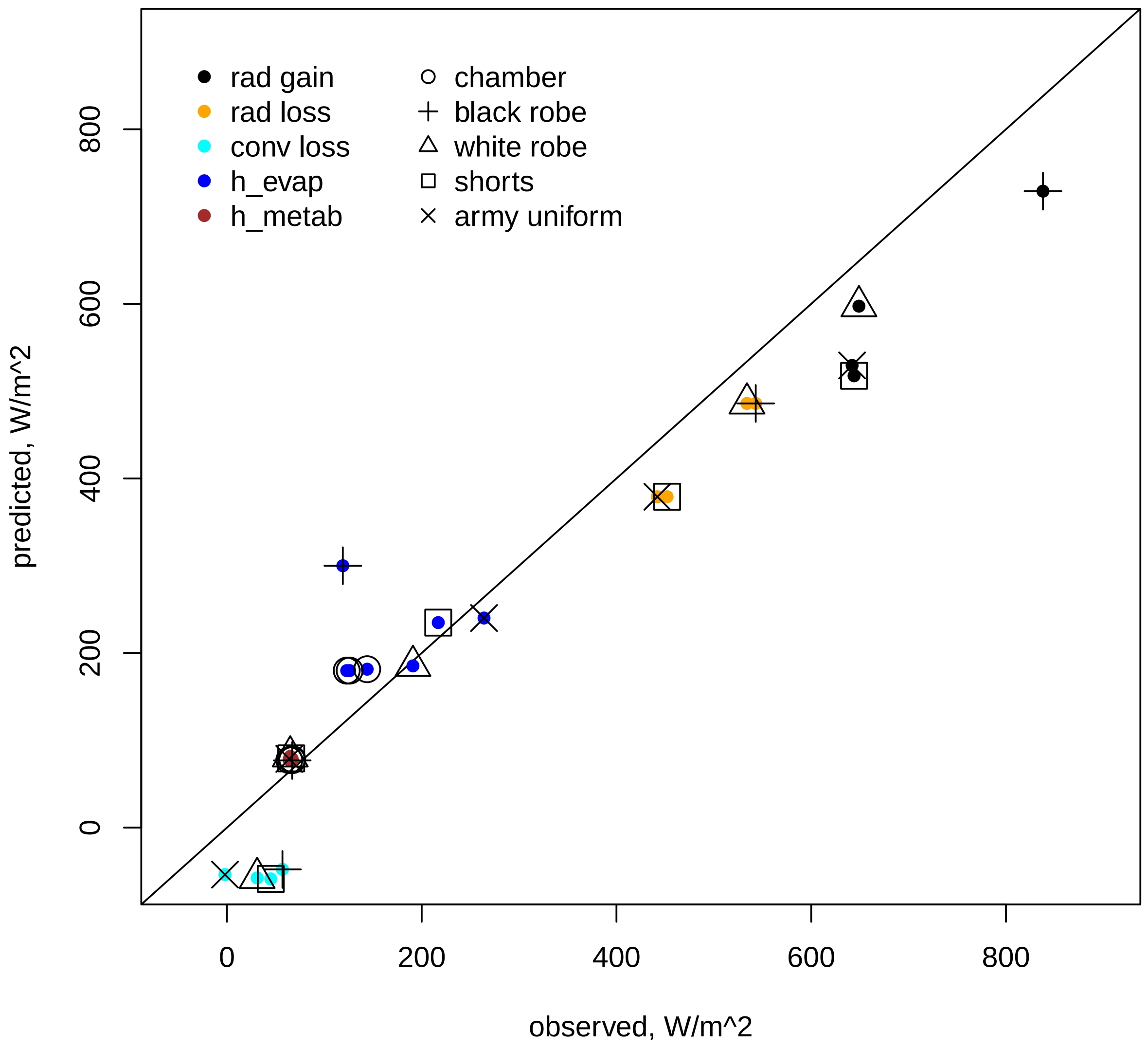

Summary statistics.

```
get.rmsd <- function(observed, predicted){
  mean((observed - predicted) ^ 2) ^ 0.5
}
obs <- c(obs$rad_gain, obs$rad_loss, obs$conv_loss, obs$h_evap, obs$h_metab)
H_pred <- c(predictions$rad_gain, predictions$rad_loss, predictions$conv_loss, predictions$h_evap, predi
M_pred <- c(MANMO.out_predictions$rad_gain, MANMO.out_predictions$rad_loss, MANMO.out_predictions$conv_

obs_pred <- na.omit(cbind(obs, H_pred, M_pred))

H_cor <- cor(obs_pred[, 1], obs_pred[, 2])
H_rmsd <- get.rmsd(obs_pred[, 1], obs_pred[, 2])

M_cor <- cor(obs_pred[, 1], obs_pred[, 3])
M_rmsd <- get.rmsd(obs_pred[, 1], obs_pred[, 3])

summary <- matrix(data = round(c(H_cor, M_cor, H_rmsd, M_rmsd), 3), nrow = 2, ncol = 2)
summary <- data.frame(summary, row.names = c("HomoTherm", "MANMO"))
colnames(summary) <- c("r", "rmsd")
knitr::kable(summary, digits = 3)
```

	r	rmsd
HomoTherm	0.987	41.004
MANMO	0.963	71.456

Summarise results in tables.

```
# environment
knitr::kable(metout[range[hour], ][c(3:8, 12:14)], digits = 3)
```

	TALOC	TAREF	RHLOC	RH	VLOC	VREF	ZEN	SOLR	TSKYC
132	37.709	35.859	12.208	13.506	1.49	1.747	15.297	833.613	13.048

```
# HomoTherm
# shorts
knitr::kable(pred.shorts[1:9], digits = 3)
```

	T_CORE	T_LUNG	T_SKIN	T_CLO	PCTWET	K_FLESH	EVAP_CUT_L	EVAP_RESP_L	SWEAT_L
12	37	36.553	35.176	38.462	29.5	1.481	0.551	0.018	0.576

```
knitr::kable(pred.shorts[10:17], digits = 3)
```

	QMETAB	QSLR	QRAD_IN	QRAD_OUT	QCONV_RESP	QEVAP_RESP	QEVAP_CUT	QCONV
12	69.808	189.855	386.923	403.093	-0.066	-6.895	-214.448	-24.128

```r
# army uniform
knitr::kable(pred.army[1:9], digits = 3)
```

	T_CORE	T_LUNG	T_SKIN	T_CLO	PCTWET	K_FLESH	EVAP_CUT_L	EVAP_RESP_L	SWEAT_L
12	37.05	36.748	35.192	42.029	33.5	1.431	0.43	0.018	0.455

```r
knitr::kable(pred.army[10:17], digits = 3)
```

	QMETAB	QSLR	QRAD_IN	QRAD_OUT	QCONV_RESP	QEVAP_RESP	QEVAP_CUT	QCONV
12	67.599	207.071	386.916	421.651	-0.081	-6.676	-163.197	-70.147

```r
# white robe
knitr::kable(pred.white[1:9], digits = 3)
```

	T_CORE	T_LUNG	T_SKIN	T_CLO	PCTWET	K_FLESH	EVAP_CUT_L	EVAP_RESP_L	SWEAT_L
12	36.9	36.498	34.923	39.241	15.5	1.781	0.311	0.018	0.314

```r
knitr::kable(pred.white[10:17], digits = 3)
```

	QMETAB	QSLR	QRAD_IN	QRAD_OUT	QCONV_RESP	QEVAP_RESP	QEVAP_CUT	QCONV
12	69.488	123.664	512.759	541.078	-0.061	-6.864	-116.946	-40.726

```r
# black robe
knitr::kable(pred.black[1:9], digits = 3)
```

	T_CORE	T_LUNG	T_SKIN	T_CLO	PCTWET	K_FLESH	EVAP_CUT_L	EVAP_RESP_L	SWEAT_L
12	36.95	36.481	34.87	45.108	24.5	1.231	0.512	0.017	0.528

```r
knitr::kable(pred.black[10:17], digits = 3)
```

	QMETAB	QSLR	QRAD_IN	QRAD_OUT	QCONV_RESP	QEVAP_RESP	QEVAP_CUT	QCONV
12	65.45	314.463	513.978	583.517	-0.056	-6.465	-192.718	-111.342

```r
# MANMO
# shorts
knitr::kable(MANMO.out_pred.shorts[1:9], digits = 3)
```

	T_CORE	T_LUNG	T_SKIN	T_CLO	PCTWET	K_FLESH	EVAP_CUT_	EVAP_RESP_	SWEAT_L
12	34.249	34.249	34.249	38.745	31.219	34.249	0.651	0.651	0.664

```r
knitr::kable(MANMO.out_pred.shorts[10:17], digits = 3)
```

	QMETAB	QSLR	QRAD_IN	QRAD_OUT	QCONV_RESP	QEVAP_RESP	QEVAP_CUT	QCONV
12	79.367	130.648	387.626	379.019	-6.948	-234.866	-234.866	58.73

```r
# army uniform
knitr::kable(MANMO.out_pred.army[1:9], digits = 3)
```

	T_CORE	T_LUNG	T_SKIN	T_CLO	PCTWET	K_FLESH	EVAP_CUT_	EVAP_RESP_	SWEAT_L
12	34.34	34.34	34.34	38.836	33.49	34.34	0.665	0.665	0.685

```r
knitr::kable(MANMO.out_pred.army[10:17], digits = 3)
```

	QMETAB	QSLR	QRAD_IN	QRAD_OUT	QCONV_RESP	QEVAP_RESP	QEVAP_CUT	QCONV
12	78.727	142.525	389.69	379.019	-6.892	-240.119	-240.119	53.819

```r
# white robe
knitr::kable(MANMO.out_pred.white[1:9], digits = 3)
```

	T_CORE	T_LUNG	T_SKIN	T_CLO	PCTWET	K_FLESH	EVAP_CUT_	EVAP_RESP_	SWEAT_L
12	34.052	34.052	34.052	38.549	26.3	34.052	0.513	0.513	0.511

```r
knitr::kable(MANMO.out_pred.white[10:17], digits = 3)
```

	QMETAB	QSLR	QRAD_IN	QRAD_OUT	QCONV_RESP	QEVAP_RESP	QEVAP_CUT	QCONV
12	81.657	84.592	498.254	485.921	-7.148	-185.288	-185.288	57.616

```r
# black robe
knitr::kable(MANMO.out_pred.black[1:9], digits = 3)
```

	T_CORE	T_LUNG	T_SKIN	T_CLO	PCTWET	K_FLESH	EVAP_CUT_	EVAP_RESP_	SWEAT_L
12	34.673	34.673	34.673	39.169	41.815	34.673	0.831	0.831	0.891

```r
knitr::kable(MANMO.out_pred.black[10:17], digits = 3)
```

	QMETAB	QSLR	QRAD_IN	QRAD_OUT	QCONV_RESP	QEVAP_RESP	QEVAP_CUT	QCONV
12	76.911	215.105	502.265	485.921	-6.733	-299.993	-299.993	47.87

References

Shkolnik, A., C. R. Taylor, V. Finch, and A. Borut. 1980. Why do Bedouins wear black robes in hot deserts? Nature 283:373–375.
