# Supplementary material for: HomoTherm: An Open‐Source Approach to Modelling Heat Exchange in Humans and Other Hominins in Diverse Environments
Source: Glob Chang Biol. 2026 Apr 1;32(4):e70830. doi: 10.1111/gcb.70830 (PMC13044332; doi:10.1111/gcb.70830)
Supplement: Supplementary file 6 — Appendix S6: gcb70830‐sup‐0006‐Appendix 6.pdf. [file GCB-32-e70830-s009.pdf]

Comparison of HomoTherm and Human Heat Balance model of Vanos et al. 2023

Michael Kearney

2026-01-14

Overview

This analysis compares the prescriptive zones calculated using an R implementation of the HHB model (Human Heat Budget; Vanos et al, 2023), the MANMO model (Myrup and Morgan, 1972), the PHS model (Predictive Heat Strain; Malchaire et al. 2001) and the HomoTherm model.

Load the libraries and data

```
library(NicheMapR)
#library(comf)
library(readxl)

localpath <- 'c:/Users/mrke/Dropbox/Current Research Projects/mammal_projects/manmo analysis/'
source(paste0(localpath, 'code/wetbulb/WetBulb.R')) # Davies-Jones function
source(paste0(localpath, 'code/HHB/HHB.R'))
source(paste0(localpath, 'code/HHB/run_HHB.R'))
source(paste0(localpath, 'code/MANMO/MANMO_R.R')) # the MANMO function
source(paste0(localpath, 'code/MANMO/run.MANMO.R'))
source(paste0(localpath, 'code/PHS/calcIso7933_Tcl.R'))
```

Environment

```
TAs <- seq(20, 60, 0.5)
Av_ms <- 1 #Air velocity in (m/s)
barometric_pressure <- 1013 # in hPa ONLY to obtain water vapor pressure when
#specific humidity is used as humidity metric.
CONV_ENHANCE <- 1 # NicheMapR parameter for augmentation of free convection
# outdoors according to Mitchell's correction
humidities <- c(0.1, 5, seq(10, 90, 10), 99.5)
Z <- 20 # zenith angle, degrees
sun <- FALSE

if(!sun){
  QSOLR <- 0 # W/m2
  mrt_Cs  <- TAs #indoor mrt = Ta (deg C)
```

```
}else{# if(sun == 'Day-Outdoors'){
  QSOLR <- 800 # W/m2
  #mrt_Cs <- TAs + 15 #outdoor mrt = Ta (deg C)
  #(Threshold to an hypothetical Partly Cloudy Condition) based on the
  #measurements from Guzman-Echavarria et al (2022)
  #mrt_Cs <- TAs + (QSOLR / 1366) * 15 # based on formula 6 from
  #Guzman-Echavarria et al (2022)
  mrt_Cs <- TAs + (QSOLR / 1366) * 30 # based on formula 7 from
  #Guzman-Echavarria et al (2022)
}

# HomoTherm environmental variables
VELs <- rep(Av_ms, length(TAs)) # wind speeds, m/s
Zs <- rep(Z, length(TAs)) # zenith angles, degrees
QSOLRs <- rep(QSOLR, length(TAs)) # solar radiation, W/m2
PDIFs <- rep(0.15, length(TAs)) # diffuse solar radiation fraction, -
TSKYs <- TAs # sky temperatures, deg C
TGRDs <- TAs # ground temperatures, deg C
BPs <- rep(barometric_pressure * 100, length(TAs)) # barometric pressures, Pa
```

Wetbulb calculations.

```
# compute Tw with Davies-Jones approach
load(paste0(localpath, 'output/Tw.DJs.35.Rda'))
load(paste0(localpath, 'output/Tw.DJs.37.Rda'))
load(paste0(localpath, 'output/Tw.DJs.39.Rda'))
if(!exists("Tw.DJs.35")){
  data_grid <- expand.grid(temperature = seq(25, 60, 1), humidity =
                             seq(0, 100, 0.5))
  Tw.DJs <- data_grid * NA
  Tw.DJs <- lapply(1:nrow(data_grid), function(x){tryCatch(
    wetbulb(data_grid[x, 1], barometric_pressure * 100,
            data_grid[x, 2], 1, 1)$Twb, error=function(err) NA)})
  Tw.DJs <- as.data.frame(do.call(rbind, Tw.DJs))
  Tw.DJs.35 <- na.omit(data_grid[Tw.DJs > 34.9 & Tw.DJs < 35.1, ])
  save(Tw.DJs.35, file = 'output/Tw.DJs.35.Rda')
}
if(!exists("Tw.DJs.37")){
  data_grid <- expand.grid(temperature = seq(25, 60, 1), humidity =
                             seq(0, 100, 0.5))
  Tw.DJs <- data_grid * NA
  Tw.DJs <- lapply(1:nrow(data_grid), function(x){tryCatch(
    wetbulb(data_grid[x, 1], barometric_pressure * 100,
            data_grid[x, 2], 1, 1)$Twb, error=function(err) NA)})
  Tw.DJs <- as.data.frame(do.call(rbind, Tw.DJs))
  Tw.DJs.37 <- na.omit(data_grid[Tw.DJs > 36.9 & Tw.DJs < 37.1, ])
  save(Tw.DJs.37, file = 'output/Tw.DJs.37.Rda')
}
if(!exists("Tw.DJs.39")){
  data_grid <- expand.grid(temperature = seq(25, 60, 1),
                           humidity = seq(0, 100, 0.5))
  Tw.DJs <- data_grid * NA
  Tw.DJs <- lapply(1:nrow(data_grid), function(x){tryCatch(
```

```
    wetbulb(data_grid[x, 1], barometric_pressure * 100,
            data_grid[x, 2], 1, 1)$Twb, error=function(err) NA)})
  Tw.DJs <- as.data.frame(do.call(rbind, Tw.DJs))
  Tw.DJs.39 <- na.omit(data_grid[Tw.DJs > 38.9 & Tw.DJs < 39.1, ])
  save(Tw.DJs.39, file = 'output/Tw.DJs.39.Rda')
}
  # par(mfrow = c(1, 1))
  # plot(Tw.DJs.35, ylim = c(0, 100), xlim = c(25, 60), xaxs = 'i', yaxs = 'i',
  #      type = 'l', col = 'lightblue')
  # points(Tw.DJs.37, ylim = c(0, 100), xlim = c(25, 60), xaxs = 'i', yaxs = 'i',
  #        type = 'l', col = 'lightblue')
  # points(Tw.DJs.39, ylim = c(0, 100), xlim = c(25, 60), xaxs = 'i', yaxs = 'i',
  #        type = 'l', col = 'lightblue')
```

HHB setup

```
# HHB setup
path_profiles  = paste0(localpath, 'data/Vanos/personal_profiles/')
list_profiles <- list.files(path_profiles, pattern = "\\.txt$", full.names = TRUE)

#Personal profile settings
name_profile <- 'Young_adult' # Options are 'Young_adult','65_over'
profile1 <- read_personal_profiles(paste0(path_profiles, name_profile,
                                          '_survivability.txt'))
```

```
## Warning in read.table(file = path_profile, header = FALSE, col.names =
## c("label", : not all columns named in 'colClasses' exist
```

```
profile <- as.data.frame(t(profile1$values))
colnames(profile) <- profile1$label
MASS  <- as.numeric(profile$Mass) # mass, kg
if(!is.finite(as.numeric(profile$AD))){
  AD <- AD_from_mass_height(as.numeric(profile$Mass),as.numeric(profile$Height)) #m2
}else{
  AD <- as.numeric(profile$AD)
}
exp_time <- 6 # Options are 1, 3, 6 (in hours)
profile$Icl <- 0 # Insulation (Clo units)
MET <- 1 # mets
# from MET_to_MetabolicRate_W_Mass, 1.225 is W/kg
M <- MET * as.numeric(profile$Mass) * 1.225

# Defining properties to estimate the combined convective and radiative heat
# fluxes. Skin temperature, assumed to be held constant at 35°C
Tsk_C <- as.numeric(profile$Tsk_C)
Emm_sk <- as.numeric(profile$Emm_sk) #Area-weighted emissivity of the clothed
# body surface (dimensionless)
#Effective radiative area of the body (dimensionless)
Ar_AD <- as.numeric(profile$A_eff)
Icl <- as.numeric(profile$Icl) #Insulation clothing value (CLO)
Smax <- as.numeric(profile$smax_rate) # maximum sweating rate, L/h
# Evaporative resistance of clothing in m2·kPa/W
```

```
Re_cl <- as.numeric(profile$Re_cl) #.001

Re_cl <- 0.01 * Icl / 0.36 # evaporative resistance of clothing in m2·kPa/W
```

HomoTherm setup

```
# morphology
HEIGHT <- as.numeric(profile$Height) * 100 # height, cm
AREA <- AD #0.00718 * MASS ^ 0.425 * HEIGHT ^ 0.725 # DuBois area, m2
if(is.na(HEIGHT)){ # get height from AD
  HEIGHT <- (AD / (.00718 * MASS ^ 0.425)) ^ (1 / 0.725)
}

# physiological properties
TC_RESTs <- rep(37, 4)
TC_ACTIVEs <- rep(40, 4)
ACTIVE <- FALSE
QMETAB_REF <- as.numeric(profile$Mass) * 1.225 # 1 MET
TC_REF <- 37
Q10 <- 2
Q10mult <- Q10 ^ ((mean(TC_RESTs) - TC_REF) / 10)
# adjusting resting metabolic rate for any departure of TC from TC_REF
QMETAB_REST <- M * Q10mult
PCTWETs <- rep(2, 4)
PCTWET_MAXs <- rep(100, 4)
MAXSWEAT <- Smax / AREA

# insulation properties
INSDENDs <- c(3e+8, 3e+5, 3e+5, 3e+5) # hair density, dorsal (1/m2)
INSDENVs <- c(3e+5, 3e+5, 3e+5, 3e+5) # hair density, ventral (1/m2)
INSDEPDs <- c(0.01, 0, 0, 0) # fur depth, dorsal (m), head has hair 1 cm thick
INSDEPVs <- c(0, 0, 0, 0) # fur depth, ventral (m)

# get clothing depth from CLO value
CLO.C4 <- Icl
# obtain insulation depth from clo value
INSDEP <- Icl * (0.155 * 0.04)
INSDEPDs[2:4] <- INSDEP
INSDEPVs[1:4] <- INSDEP

# compute bare evaporation area (from MANMO)
PT_clo.A9 <- min(1, -0.0239 + 1.794 * CLO.C4 - 1.101 * CLO.C4 ^ 2 + 0.225 *
                   CLO.C4 ^ 3)
if(CLO.C4 == 0){
  PT_clo.A9 <- 0
}
PCTBAREVAPs <- c(60, rep(1 - PT_clo.A9, 3) * 100) # head assumed to be 60% bare
```

MANMO

```
# model mode
if(Icl == 0){
  CLO.mode <- 2
```

```r
}else{
  CLO.mode <- 0
}

# MANMO physiological inputs
G_m.G2 <- QMETAB_REST / AREA # W / m2
K3 <- c(0.014, 0.01, 0.072, 1) * 4186 / 3600 # W m / (m2 C)
```

Now run the simulations.

Results differ between HHB and HomoTherm because of a) very rapid linear rise in TC predicted by the HHB approach compared to the steady state calculation of HomoTherm with the same TC_max assumed by HHB, and the assumed skin temperature remaining at 35 deg C the whole time with HHB. Can get similar results if cap skin temperature to a lower threshold but 41 should be the upper possible (approach pain threshold).

```r
run.HomoTherm <- FALSE
EXCEED.TCMAX <- TRUE
HomoThermInput <- FALSE
plot.legend <- TRUE
plot.variables <- TRUE
plot.manmo <- TRUE
save.out <- TRUE
MAX_TSKIN <- 40 # 36.7 matches HHB
TC_lethal <- 43
Tsk_C <- 35 # HHB T_skin

outdir <- 'c:/Users/mrke/Dropbox/Current Research Projects/mammal_projects/manmo analysis/output/HHB_Ho

survive <- matrix(nrow = length(humidities), ncol = 8)

balance2s <- NULL
balance1s <- NULL
HHB.outputs <- NULL

########################## Run and compare ######################
for(i in 1:length(humidities)){
  humidity <- humidities[i]

  # run HomoTherm
  if(run.HomoTherm){
    HomoTherm.out <- HomoTherm_var(MASS = MASS,
                                   QMETAB_REST = QMETAB_REST,
                                   MET = MET,
                                   ACTIVE = ACTIVE,
                                   TC_RESTs = TC_RESTs,
                                   TC_ACTIVEs = TC_ACTIVEs,
                                   TC_MAXs = rep(TC_lethal, 4),
                                   TC_INCs = rep(0.01, 4),
                                   PCTWET_INCs = rep(0.1, 4),
                                   INSDENDs = INSDENDs,
                                   INSDENVs = INSDENVs,
                                   INSDEPDs = INSDEPDs,
                                   INSDEPVs = INSDEPVs,
```

```
                                    PCTWETs = PCTWETs,
                                    PCTWET_MAXs = PCTWET_MAXs,
                                    PCTBAREVAPs = PCTBAREVAPs,
                                    MAXSWEAT = MAXSWEAT,
                                    QSOLRs = QSOLRs,
                                    Zs = Zs,
                                    PDIFs = PDIFs,
                                    TAs = TAs,
                                    TSKYs = TSKYs,
                                    TGRDs = TGRDs,
                                    RHs = rep(humidity, length(TAs)),
                                    VELs = VELs,
                                    EXCEED.TCMAX = EXCEED.TCMAX,
                                    CONV_ENHANCE = CONV_ENHANCE,
                                    MAXITER = 20000)
  if(save.out){
    if(sun){
      if(EXCEED.TCMAX){
        save(HomoTherm.out, file = paste0(outdir, 'HomoTherm.out_', i, '_sun.Rda'))
      }else{
        save(HomoTherm.out, file = paste0(outdir, 'HomoTherm.out_', i,
                                          '_sun_negative.Rda'))
      }
    }else{
      if(EXCEED.TCMAX){
        save(HomoTherm.out, file = paste0(outdir, 'HomoTherm.out_', i, '.Rda'))
      }else{
        save(HomoTherm.out, file = paste0(outdir, 'HomoTherm.out_', i,
                                          '_negative.Rda'))
      }
    }
  }
}else{
  if(sun){
    if(EXCEED.TCMAX){
      load(paste0(outdir, 'HomoTherm.out_', i, '_sun.Rda'))
    }else{
      load(paste0(outdir, 'HomoTherm.out_', i, '_sun_negative.Rda'))
    }
  }else{
    if(EXCEED.TCMAX){
      load(paste0(outdir, 'HomoTherm.out_', i, '.Rda'))
    }else{
      load(paste0(outdir, 'HomoTherm.out_', i, '_negative.Rda'))
    }
  }
}
balance <- HomoTherm.out$balance
G_m.G2s <- rep(G_m.G2, length(TAs))
Tskin1.T <- rep(2, length(TAs))
#iterate <- 1
W <- rep(PCTWETs[2] / 100, length(TAs))
M_HHB <- rep(M, length(TAs))
```

```r
Tsk_C_HHB <- rep(Tsk_C, length(TAs))
Tre_ISO <- rep(36.8, length(TAs))
Tsk_ISO <- TAs
if(HomoThermInput){
  G_m.G2s <- balance$QMETAB / AREA
  G_m.G2s[G_m.G2s < G_m.G2] <- G_m.G2
  #Tskin1.T <- balance$T_SKIN
  #iterate <- 1
  W <- balance$PCTWET / 100
  #W[Tskin1.T > 33] <- 0.25 * Tskin1.T[Tskin1.T > 33] - 8.25
  #W[Tskin1.T > 37] <- 1
  M_HHB <- balance$QMETAB
  Tsk_C_HHB <- balance$T_SKIN
  Tre_ISO <- balance$T_CORE
  Tsk_ISO <- balance$T_SKIN
}

# run MANMO_R twice, once at base skin wetness and once at max skin wetness
MANMO.out <- run.MANMO(W = W,
                       Ht.H4 = HEIGHT,
                       Wt.W4 = MASS,
                       #iterate = iterate,
                       #Tskin1.T = Tskin1.T,
                       K3 = K3,
                       D3 = c(mean(INSDEPDs[2:4]), rep(1e-10, 3)),
                       Maximum.SR = MAXSWEAT * 1000 / 60,
                       G_m.G2s = G_m.G2s,
                       CLO.C4 = CLO.C4,
                       CLO.mode = CLO.mode,
                       TAs = TAs,
                       TSKYs = TSKYs,
                       TGNDs = TGRDs,
                       RH.H2s = rep(humidity, length(TAs)) / 100,
                       Q_hs = QSOLRs / AREA,
                       q_hs = QSOLRs * PDIFs / AREA,
                       VELs = VELs)

HHB <- lapply(1:length(TAs),
              function(x){run_HHB(exp_time = exp_time,
                                  AD = AD,
                                  M = M_HHB[x],
                                  Tsk_C = Tsk_C_HHB[x],
                                  Emm_sk = Emm_sk,
                                  Ar_AD = Ar_AD,
                                  Icl = Icl,
                                  Ta_C = TAs[x],
                                  humidity = humidity,
                                  Av_ms = Av_ms,
                                  mrt_C = mrt_Cs[x],
                                  deltaT = TC_lethal - TC_RESTs[2],
                                  Mass = MASS,
                                  Smax = Smax,
                                  Re_cl = Re_cl,
```

```
                                   wmax_condition = PCTWET_MAXs[2] / 100
              )})
HHB.output <- as.data.frame(do.call(rbind, HHB))

HHB.output$Ereq[HHB.output$Ereq < 0] <- 0
HHB.output$Sreq[HHB.output$Sreq < 0] <- 0

Iso7933 <- lapply(1:length(TAs),
                  function(x){
                    calcIso7933_Tcl(accl = 100,
                                    posture = 1,
                                    Duration = exp_time * 60,
                                    Ta = TAs[x],
                                    Pa = WETAIR(db = TAs[x],
                                      rh = humidity)$e / 1000,
                                    Tr = mrt_Cs[x],
                                    Va = VELs[x],
                                    Tsk = Tsk_ISO[x],
                                    Met = G_m.G2s[x],
                                    Icl = Icl + 0.001,
                                    weight = MASS,
                                    height = HEIGHT / 100,
                                    Adu = AREA,
                                    Tre = Tre_ISO[x],
                                    Tcr = Tre_ISO[x],
                                    SWp = 0.1)
                  })
ISO.out <- as.data.frame(do.call(rbind, Iso7933))

HHB.output$TA <- TAs
balance$TA <- TAs
MANMO.out$TA <- TAs
ISO.out$TA <- TAs
HHB.output$RH <- humidity
balance$RH <- humidity
MANMO.out$RH <- humidity
ISO.out$RH <- humidity
HHB.output$VEL <- Av_ms
balance$VEL <- Av_ms
MANMO.out$VEL <- Av_ms
ISO.out$VEL <- Av_ms
HHB.output$SOLR <- QSOLR
balance$QSOLR <- QSOLR
MANMO.out$QSOLR <- QSOLR
ISO.out$QSOLR <- QSOLR
HHB.output$CLO <- Icl
balance$INSDEP <- INSDEPDs[2]
MANMO.out$CLO <- Icl
ISO.out$CLO <- Icl

MANMO.bal <- with(MANMO.out, M_m.M + R_m.R + I_m.I + E_m.E + H_m.H + D_m.D)
MANMO.balance <- cbind(TAs, MANMO.out$Tskin, MANMO.out$W, MANMO.out$sweat.L.h,
                       MANMO.out$M_m.M, MANMO.out$R_m.R, MANMO.out$I_e.E1,
```

```
                         MANMO.out$E_m.E, MANMO.out$H_m.H, MANMO.out$D_m.D,
                         MANMO.out$I.I1, MANMO.bal)
colnames(MANMO.balance) <- c("TA",  "Tskin", "W", "sweat", "Met", "Sol",
                               "IrIn", "Evap", "Conv", "Cond", "IrOut", "bal")
MANMO.balance <- as.data.frame(MANMO.balance)

# compute implied TC increase
cp <- 2.98 # kJ/kg/degC
MANMO.balance$heat.storage <- MANMO.balance$bal * 3600 * exp_time / 1000 # kJ
MANMO.balance$deltaC <- MANMO.balance$heat.storage / (cp * MASS) # deg C
MANMO.balance$TC_steady <- 37 + MANMO.balance$deltaC # deg C
# find the excess heat that needs to be evaporated but
# can't be due to lack of vapour pressure gradient
HHB.output$TC_steady <- 37 + ((HHB.output$Ereq - HHB.output$Emax_env)
                               * 3600 * exp_time / 1000) / (cp * MASS)
HHB.output$TC_steady[HHB.output$TC_steady < 37] <- 37

HomoTherm.bal <- QMETAB_REST +
  with(balance, QSLR + QRAD_IN - QRAD_OUT + QCONV_RESP + QEVAP_RESP +
         QEVAP_CUT + QCONV) # QMETAB_REST - balance$QMETAB
HomoTherm.bal[HomoTherm.bal < 0] <- 0
balance$bal <- HomoTherm.bal
if(!EXCEED.TCMAX){
  balance$heat.storage <- balance$bal * 3600 * exp_time / 1000 # kJ
  balance$deltaC <- balance$heat.storage / (cp * MASS) # deg C
  balance$TC_steady <- balance$T_CORE + balance$deltaC # deg C
}else{
  balance$TC_steady <- balance$T_CORE
}

# now remove MANMO output not in survivable zone
MANMO.balance2 <- subset(MANMO.balance, TC_steady <= TC_lethal &
                           Tskin < MAX_TSKIN)
lethal.MANMO <- max(MANMO.balance2$TA)

sweatlimit.MANMO <- TAs[which(MANMO.balance2$sweat > HHB.output$Smax[1])][1]
if(is.na(sweatlimit.MANMO) | sweatlimit.MANMO > lethal.MANMO){
  sweatlimit.MANMO <- lethal.MANMO
}

# now remove homotherm output not in survivable zone
# (don't let skin be so hot as to be causing rapid tissue damage
# (Gagge & Gonzalez review)
balance2 <- subset(balance, TC_steady <= TC_lethal & T_SKIN < MAX_TSKIN)
lethal.HomoTherm <- max(balance2$TA)
HomoTherm.survive <- balance2

sweatlimit.HomoTherm <- TAs[which(balance$SWEAT_L > HHB.output$Smax[1])][1]
if(is.na(sweatlimit.HomoTherm) | sweatlimit.HomoTherm > lethal.HomoTherm){
  sweatlimit.HomoTherm <- lethal.HomoTherm
}

survive.ISO <- subset(ISO.out, Tre <= TC_lethal & Tskeq < MAX_TSKIN)
```

```r
lethal.ISO <- max(survive.ISO$TA)
sweatlimit.ISO <- TAs[which(survive.ISO$SWtotg / exp_time / 1000 >
                              HHB.output$Smax[1])][1]
if(is.na(sweatlimit.ISO) | sweatlimit.ISO > lethal.HomoTherm){
  sweatlimit.ISO <- lethal.ISO
}

HHB.output2 <- HHB.output[balance$T_SKIN < MAX_TSKIN, ]
sweatlimit.HHB <- TAs[which(HHB.output2$survivability > 1)[1]]
lethal.HHB <- TAs[which(HHB.output2$survivability %in% c(3, 5))[1]]
#lethal.HHB <- TAs[which(HHB.output$TC_steady > TC_lethal)][1]
if(is.na(lethal.HHB)){
  lethal.HHB <- TAs[max(which(HHB.output2$r != -Inf))]
}

# HHB categories
# 2. survive despite exceeding sweating limits
# 3. not survive because the environment restricts heat loss too much
#(in high humidity)
# 4. not survive because the required sweat rate is not possible (in low humidity)
# 5. not survive due to both critical environmental heat loss restrictions
# (3rd argument) and not possible sweat rate to dissipate heat (4th argument).
#col.stress <- c('yellow', 'orange', 'violet', 'purple')

if(plot.variables){
  par(mfrow = c(2, 1))
  # par(oma = c(2, 1, 2, 2) + 0.1)
  # par(mar = c(3, 3, 1.5, 3) + 0.1)
  # par(mgp = c(2, 1, 0))
  par(oma = c(3, 1, 1, 1) + 0.1) # margin spacing
  par(mar = c(1, 3, 1, 1) + 0.1) # margin spacing
  par(mgp = c(2, 1, 0) ) # margin spacing
  # core and skin temperature plot
  plot(HHB.output2$TA, HHB.output2$TC_steady, type = 'l', ylab =
         expression('body temperature, '*degree*C),
       xlab = expression('air temperature, '*degree*C), ylim = c(30, 45),
       xlim = c(min(TAs), max(TAs)), lwd = 2, col = 'orange')
  points(balance$TA, Tsk_C_HHB, type = 'l', col = 'orange', lwd = 1, lty = 2)
  points(balance$TA, balance$TC_steady, type = 'l', col = 'black', lwd = 2)
  points(balance$TA, balance$T_CORE, type = 'l', col = 'black', lwd = 2, lty = 2)
  points(balance$TA, balance$T_SKIN, type = 'l', col = 'black', lwd = 1, lty = 2)
  if(plot.manmo){
    points(survive.ISO$TA, survive.ISO$Tre, type = 'l', col = 'darkgreen',
         lwd = 2, lty = 1)
    points(survive.ISO$TA, survive.ISO$Tskeq, type = 'l', col = 'darkgreen',
         lwd = 1, lty = 2)
    points(MANMO.balance$TA, MANMO.balance$TC_steady, type = 'l', col = 'grey',
           lty = 1, lwd = 2)
    points(MANMO.balance$TA, MANMO.balance$Tskin, type = 'l', col = 'grey',
           lty = 2,
           lwd = 1)
    abline(v = lethal.MANMO + 0.05, lty = 2, col = 'grey', lwd = 2)
    abline(v = sweatlimit.MANMO + 0.05, lty = 4, col = 'grey')
```

```
  }
  abline(v = lethal.HomoTherm, col = 'black', lty = 2, lwd = 2)
  abline(v = lethal.HHB - 0.05, lty = 2, col = 'orange', lwd = 2)
  abline(v = sweatlimit.HHB - 0.05, lty = 4, col = 'orange')
  abline(v = sweatlimit.HomoTherm, lty = 4, col = 'black')
  abline(v = lethal.ISO, col = 'darkgreen', lty = 2, lwd = 2)
  abline(v = sweatlimit.ISO, lty = 4, col = 'darkgreen')

  abline(h = TC_lethal, col = 'red', lty = 2)
  abline(h = MAX_TSKIN, col = 'pink', lty = 2)
  text(25, TC_lethal + 1, 'max T_core', col = 'red')
  text(25, MAX_TSKIN + 1, 'max T_skin', col = 'pink')

  # sweat plot
  plot(HHB.output2$TA, HHB.output2$Sreq, type = 'l', ylab = 'sweat, L / h',
       xlim = c(min(TAs), max(TAs)), ylim = c(0, 1.4), lwd = 2, col = 'orange')
  points(balance$TA, balance$SWEAT_L, type = 'l', col = 'black', lwd = 2)
  points(survive.ISO$TA, survive.ISO$SWtotg / exp_time / 1000, type = 'l',
         col = 'darkgreen', lwd = 2)
  abline(v = lethal.HomoTherm, col = 'black', lty = 2, lwd = 2)
  abline(v = lethal.HHB - 0.05, lty = 2, col = 'orange', lwd = 2)
  abline(h = HHB.output2$Smax[1], col = 'blue', lty = 2)
  abline(v = sweatlimit.HHB - 0.05, lty = 4, col = 'orange')
  abline(v = sweatlimit.HomoTherm, lty = 4, col = 'black')
  if(plot.manmo){
    abline(v = lethal.ISO, col = 'darkgreen', lty = 2, lwd = 2)
    abline(v = sweatlimit.ISO, lty = 4, col = 'darkgreen')
    points(MANMO.balance$TA, MANMO.balance$sweat, type = 'l', col = 'grey',
           lwd = 2)
    abline(v = lethal.MANMO + 0.05, lty = 2, col = 'grey', lwd = 2)
    abline(v = sweatlimit.MANMO + 0.05, lty = 4, col = 'grey')
  }
  text(25, HHB.output2$Smax[1] + 0.1, 'max sweat', col = 'blue')

  mtext(text = paste('RH =', humidity, '%, lethal =',
                     min(lethal.HomoTherm, sweatlimit.HomoTherm, na.rm = TRUE),
                     '/', min(sweatlimit.HHB, lethal.HHB, na.rm = TRUE), 'deg C'),
        outer = TRUE, side = 3, line = -.5)
  mtext(text = expression('air temperature, '*degree*C), outer = TRUE,
        side = 1, line = 1)
}
survive[i, 1] <- lethal.HomoTherm
survive[i, 2] <- lethal.HHB
survive[i, 3] <- lethal.MANMO
survive[i, 4] <- lethal.ISO
survive[i, 5] <- sweatlimit.HomoTherm
survive[i, 6] <- sweatlimit.HHB
survive[i, 7] <- sweatlimit.MANMO
survive[i, 8] <- sweatlimit.ISO
if(i == 1){
  balance1s <- balance
  balance2s <- balance2
  MANMO.balances <- MANMO.balance
```

```
    MANMO.balances2 <- MANMO.balance2
    HHB.outputs <- HHB.output
    survive.ISOs <- survive.ISO
  }else{
    balance1s <- rbind(balance1s, balance)
    balance2s <- rbind(balance2s, balance2)
    MANMO.balances <- c(MANMO.balances, MANMO.balance)
    MANMO.balances2 <- c(MANMO.balances2, MANMO.balance2)
    HHB.outputs <- rbind(HHB.outputs, HHB.output)
    survive.ISOs <- rbind(survive.ISOs, survive.ISO)
  }
}
```

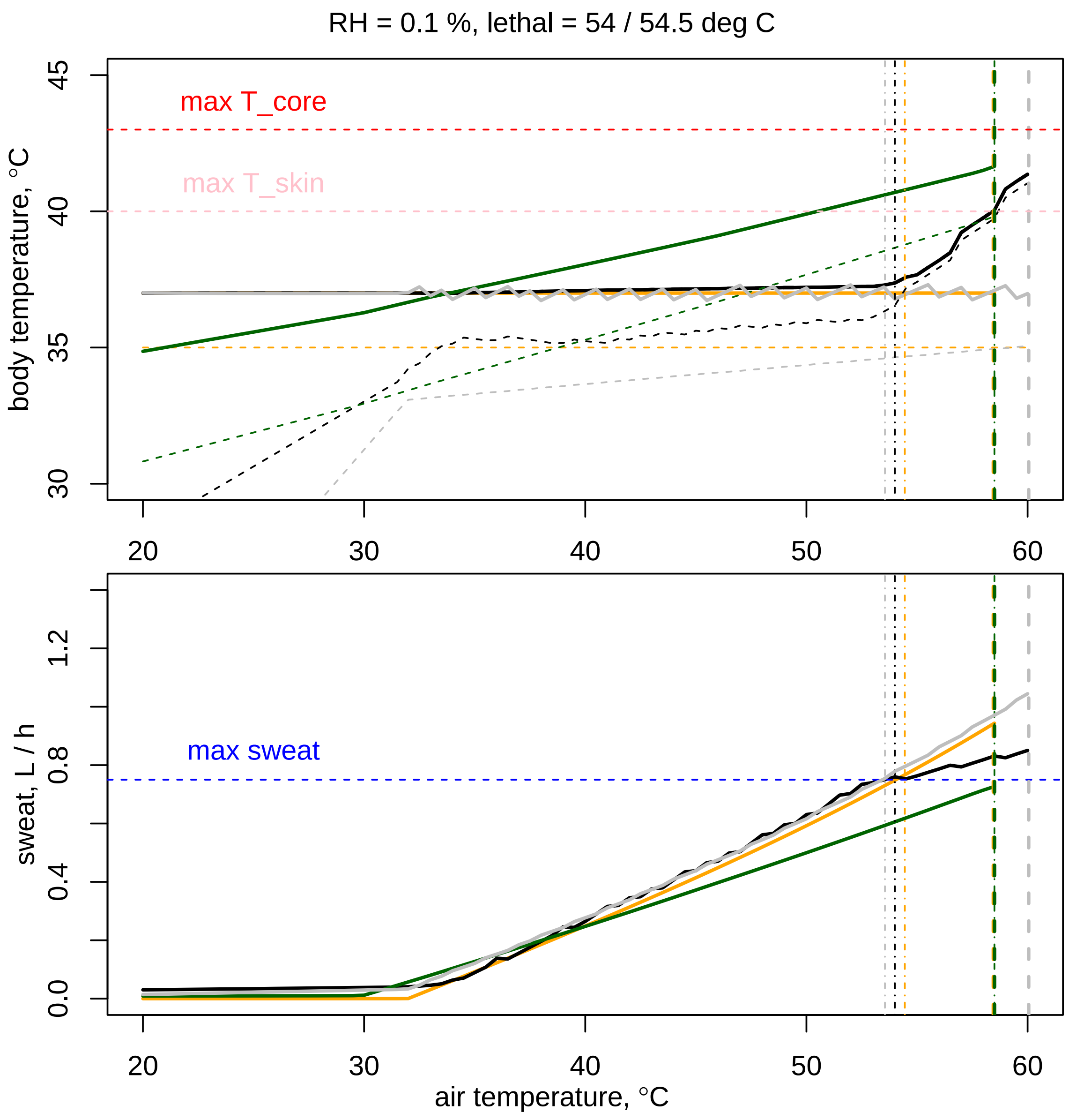

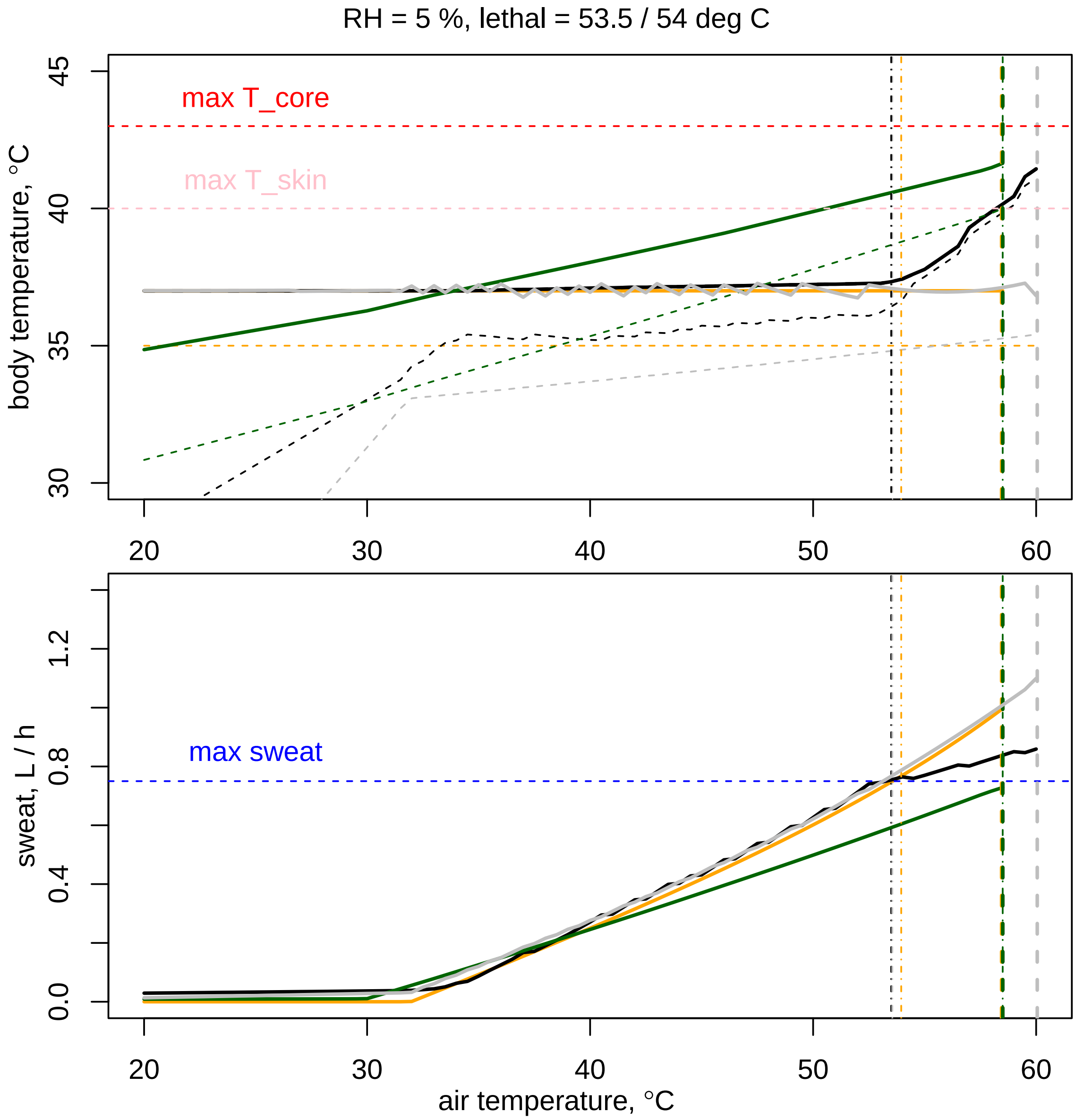

RH = 5 %, lethal = 53.5 / 54 deg C
max T_core
max T_skin
body temperature, °C
45
40
35
30
20
30
40
50
60
max sweat
sweat, L / h
1.2
0.8
0.4
0.0
air temperature, °C

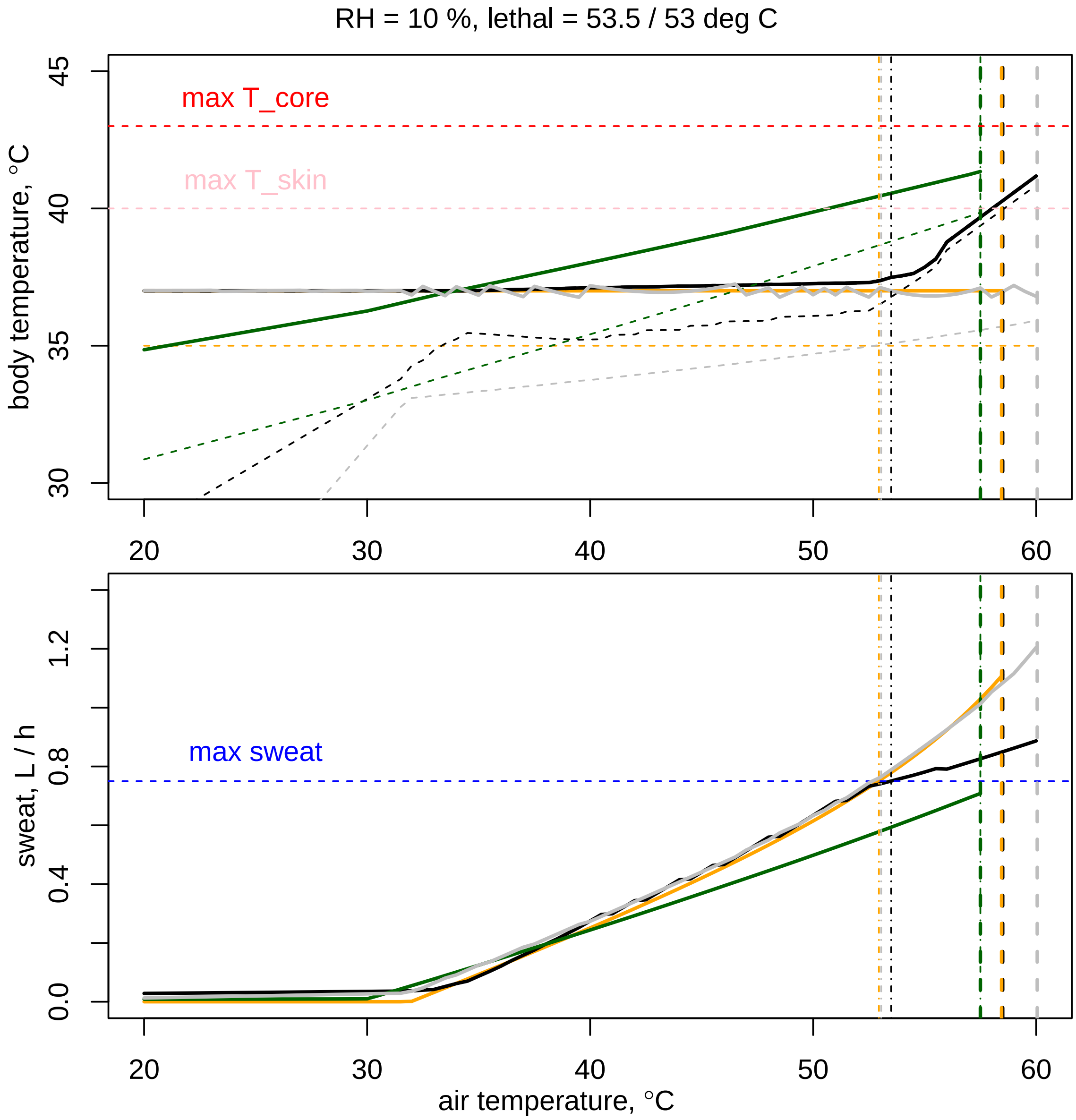
RH = 10 %, lethal = 53.5 / 53 deg C
max T_core
max T_skin
body temperature, °C
45
40
35
30
20
30
40
50
60
max sweat
sweat, L / h
1.2
0.8
0.4
0.0
20
30
40
50
60
air temperature, °C

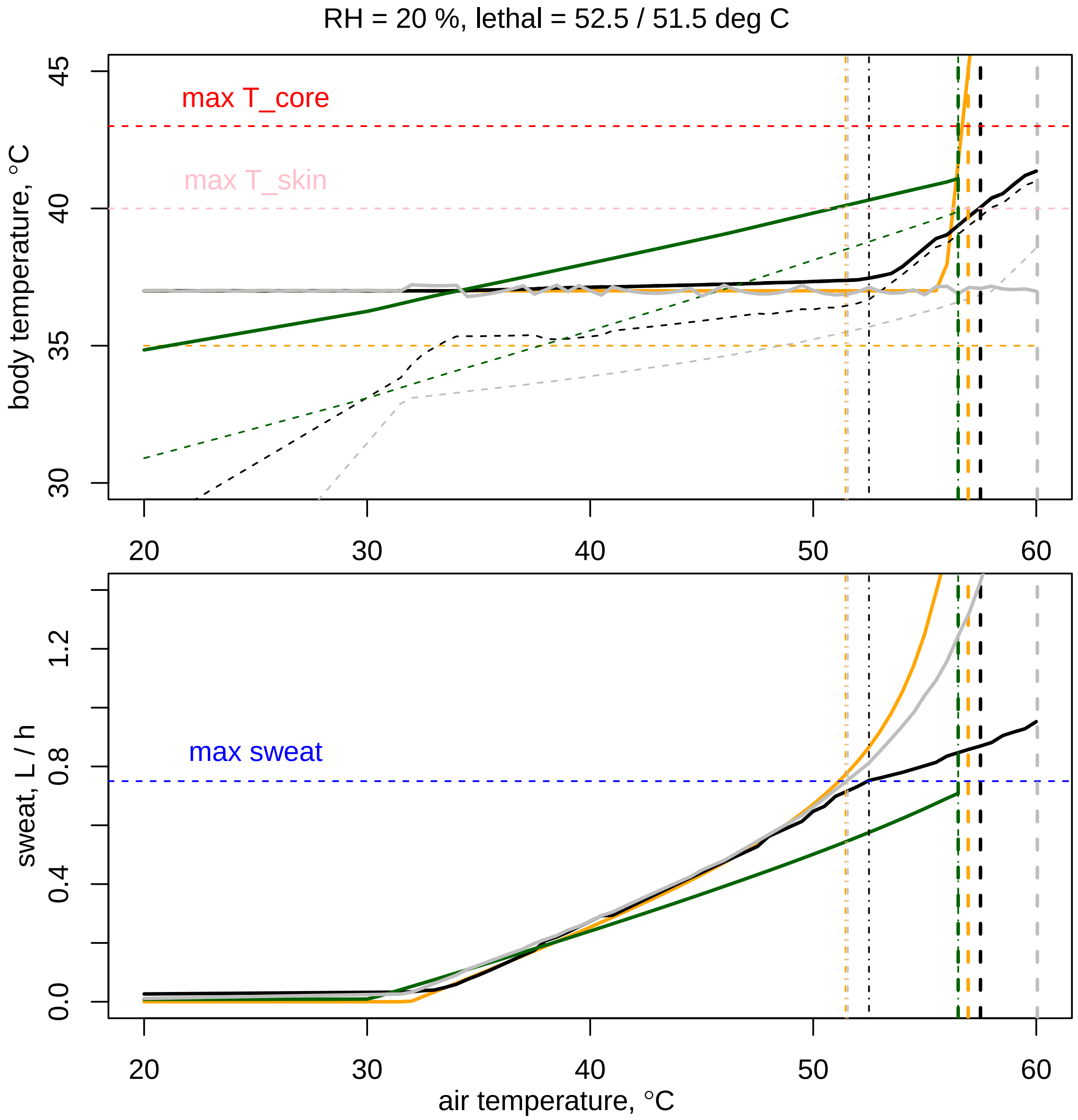

RH = 20 %, lethal = 52.5 / 51.5 deg C
max T_core
max T_skin
body temperature, °C
45
40
35
30
20
30
40
50
60
max sweat
sweat, L / h
1.2
0.8
0.4
0.0
20
30
40
50
60
air temperature, °C

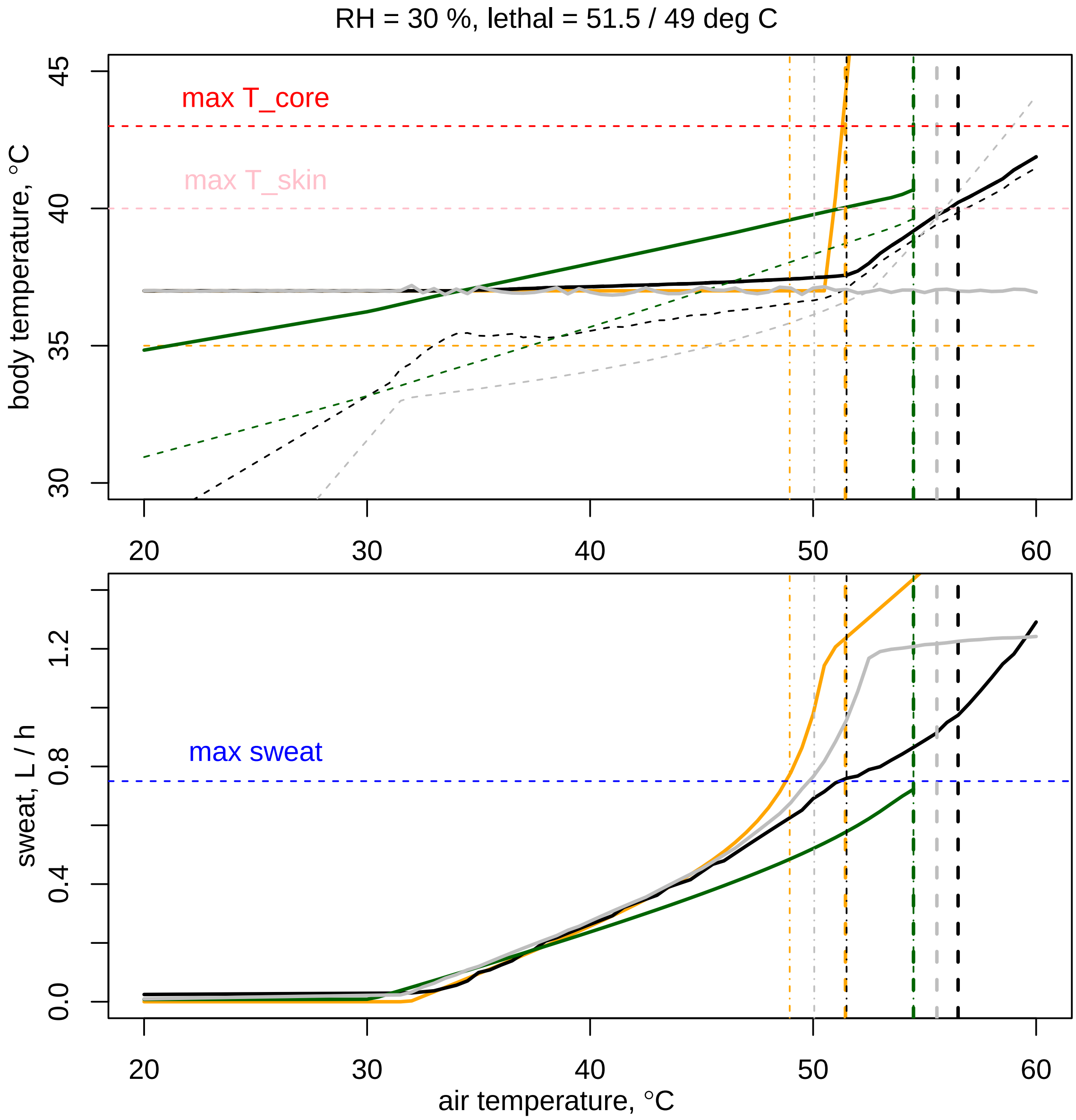
RH = 30 %, lethal = 51.5 / 49 deg C
max T_core
max T_skin
body temperature, °C
45
40
35
30
20
30
40
50
60
max sweat
sweat, L / h
1.2
0.8
0.4
0.0
air temperature, °C

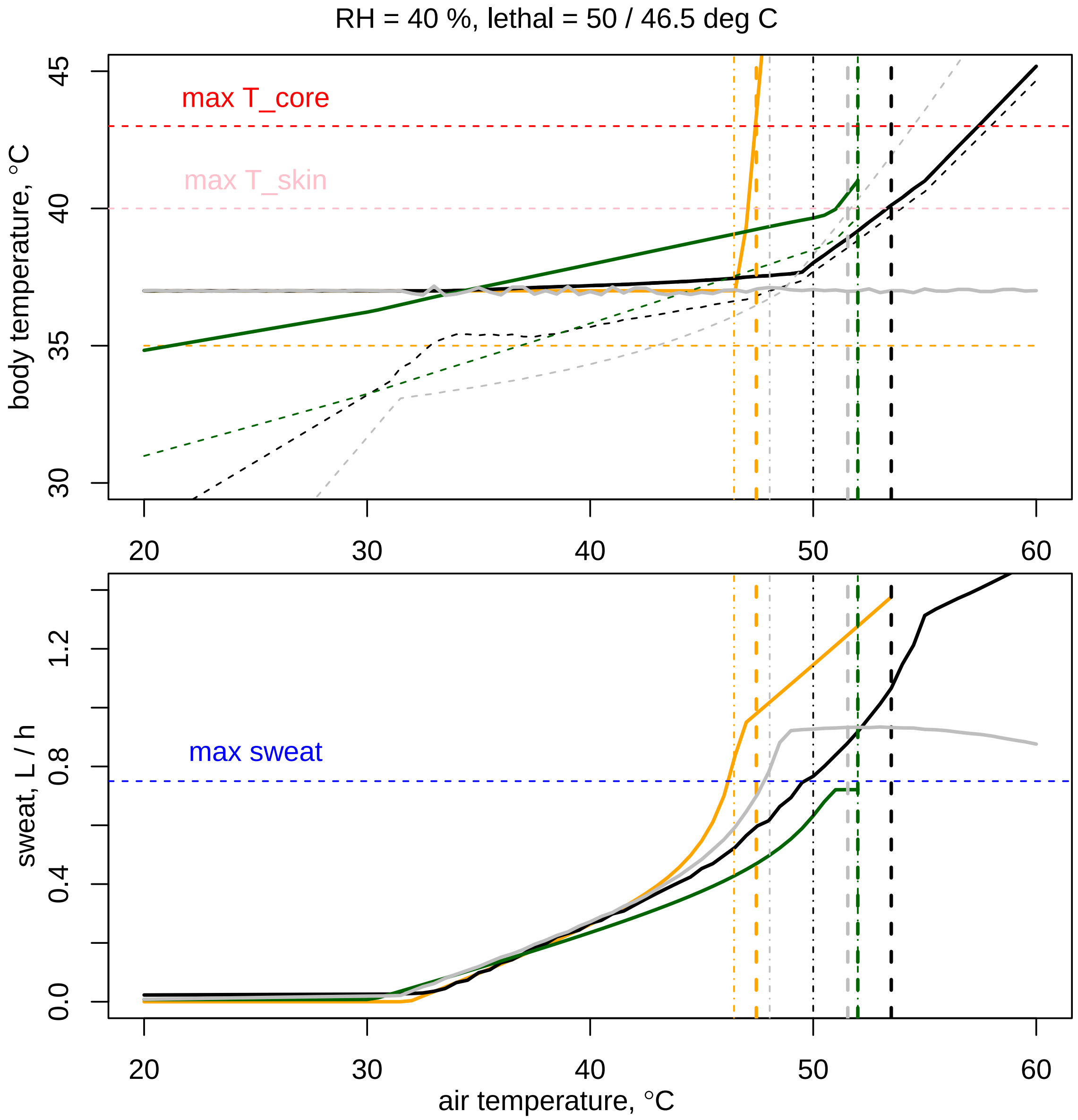

RH = 40 %, lethal = 50 / 46.5 deg C
max T_core
max T_skin
body temperature, °C
max sweat
sweat, L / h
air temperature, °C

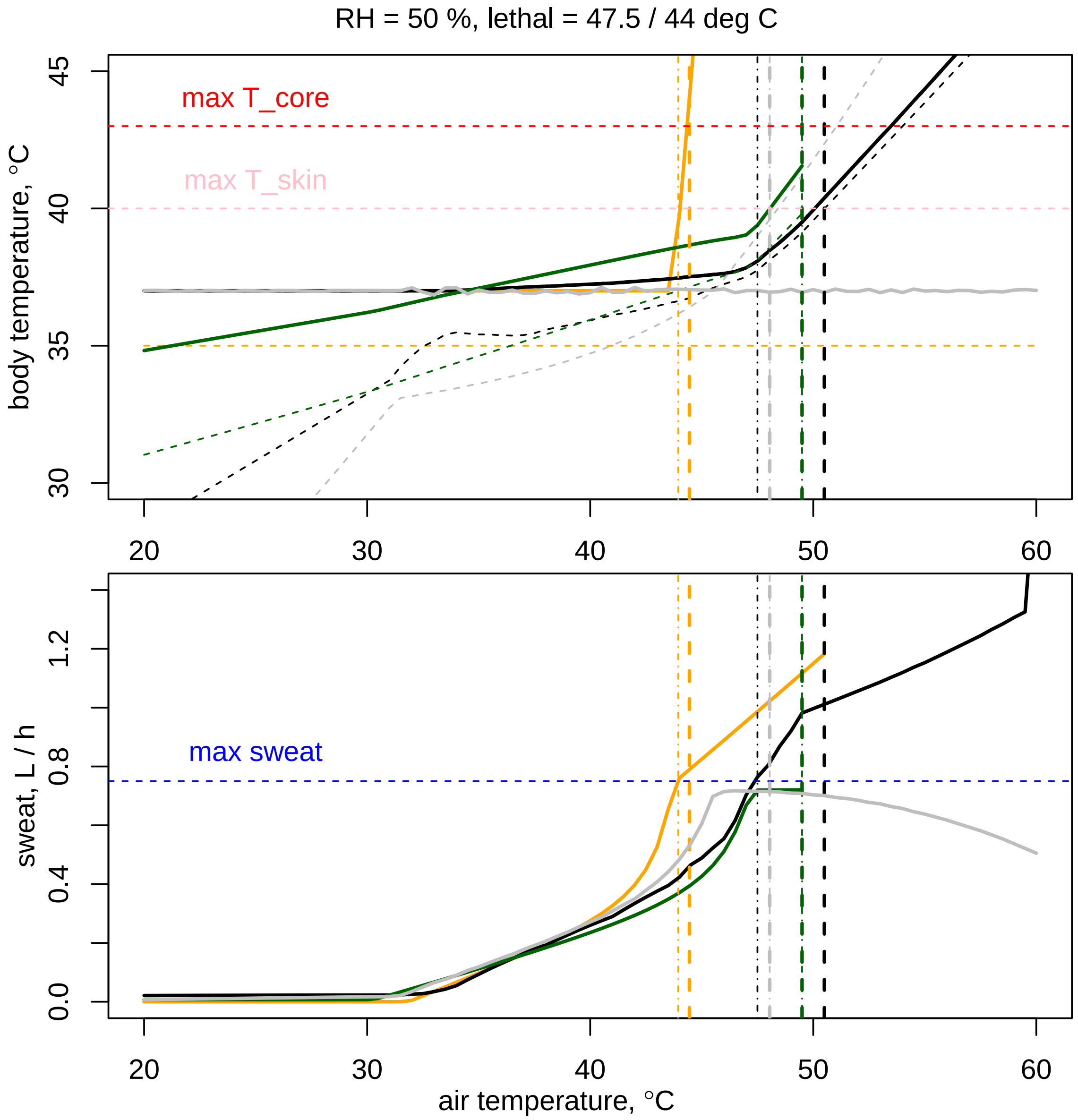

RH = 50 %, lethal = 47.5 / 44 deg C
max T_core
max T_skin
body temperature, °C
30
35
40
45
20
30
40
50
60
max sweat
sweat, L / h
0.0
0.4
0.8
1.2
air temperature, °C

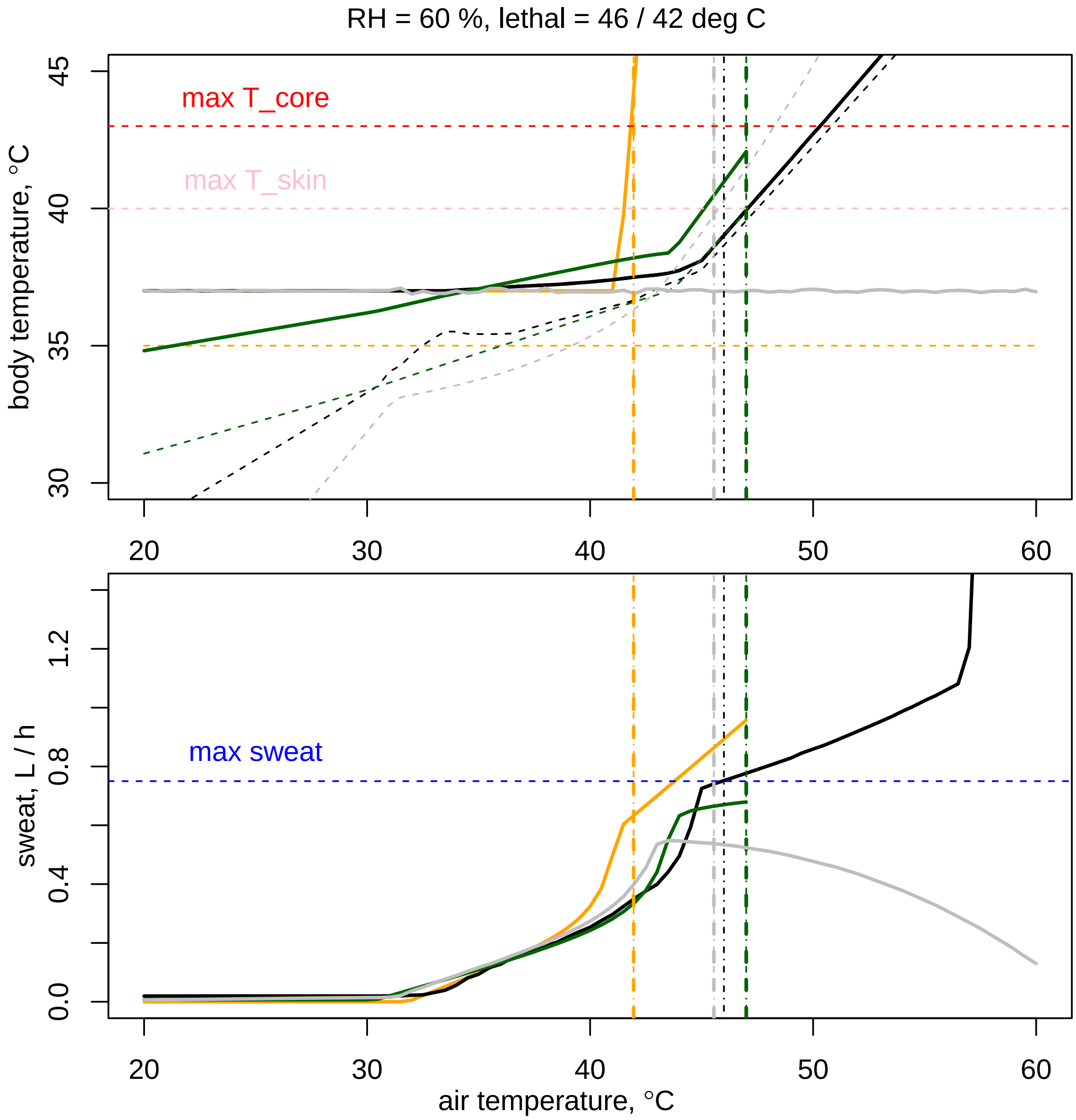
RH = 60 %, lethal = 46 / 42 deg C
max T_core
max T_skin
body temperature, °C
45
40
35
30
20
30
40
50
60
max sweat
sweat, L / h
1.2
0.8
0.4
0.0
20
30
40
50
60
air temperature, °C

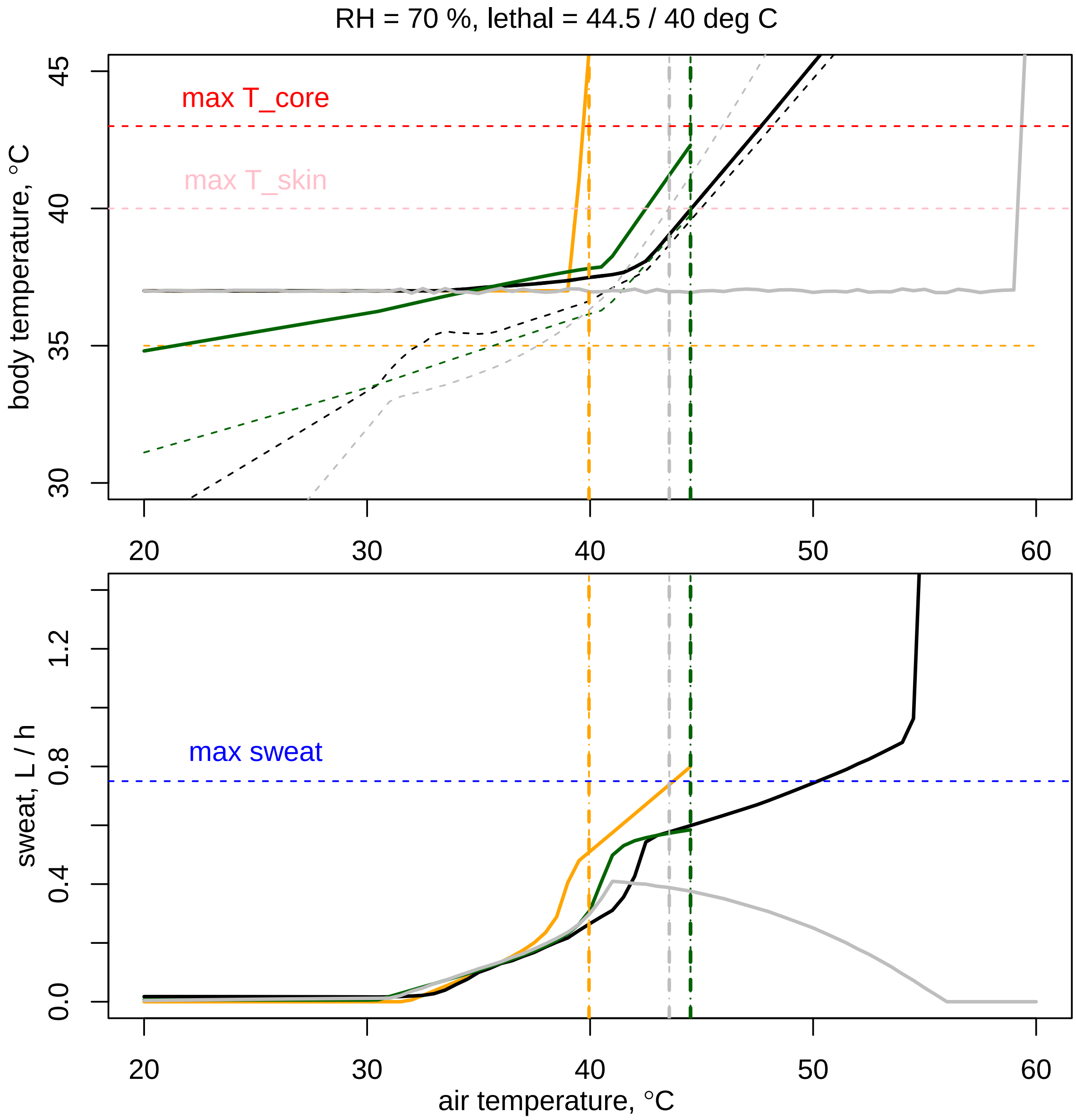
RH = 70 %, lethal = 44.5 / 40 deg C
max T_core
max T_skin
body temperature, °C
45
40
35
30
20
30
40
50
60
max sweat
sweat, L / h
1.2
0.8
0.4
0.0
20
30
40
50
60
air temperature, °C

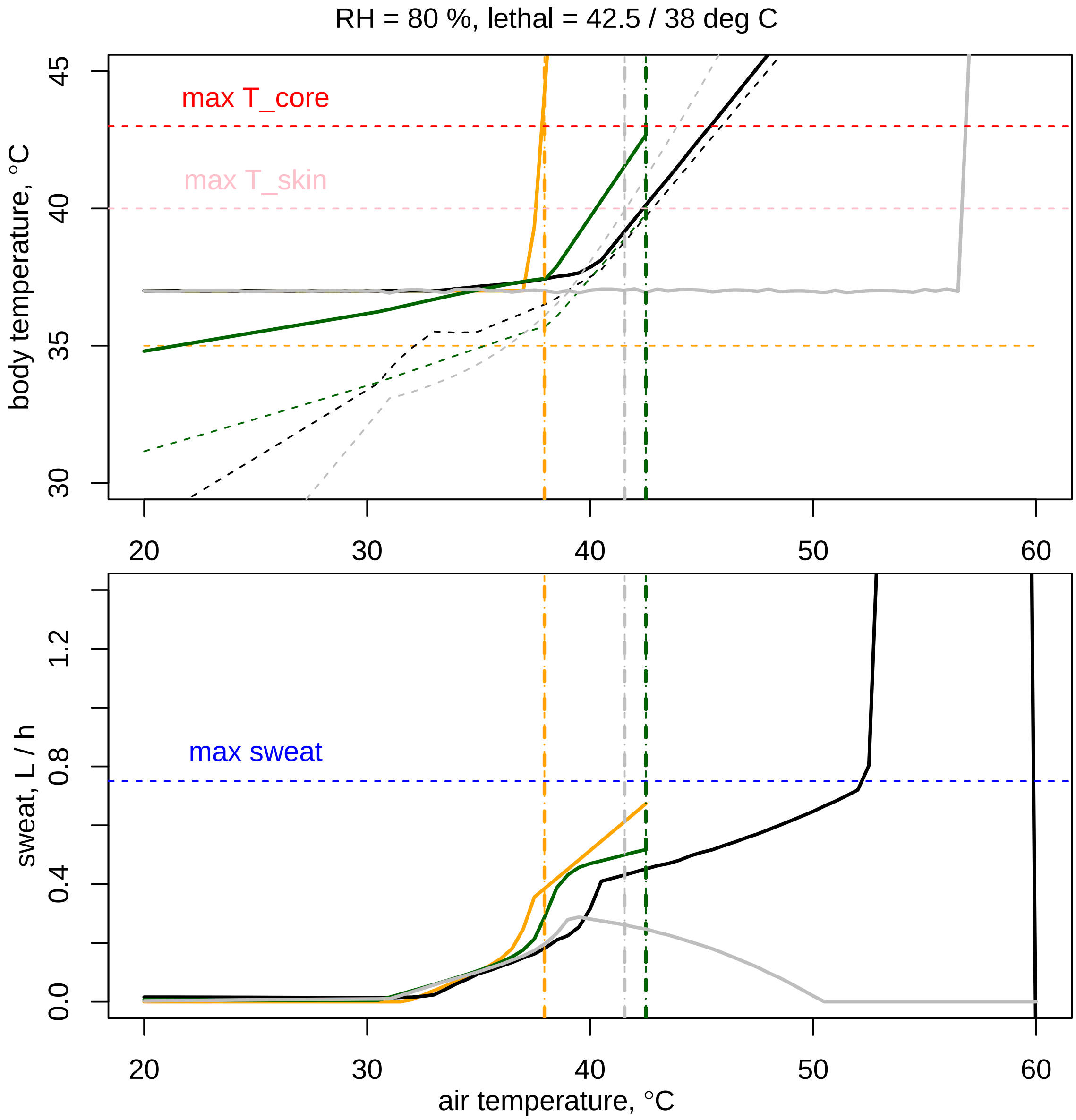

RH = 80 %, lethal = 42.5 / 38 deg C
max T_core
max T_skin
body temperature, °C
30
35
40
45
20
30
40
50
60
max sweat
sweat, L / h
0.0
0.4
0.8
1.2
20
30
40
50
60
air temperature, °C

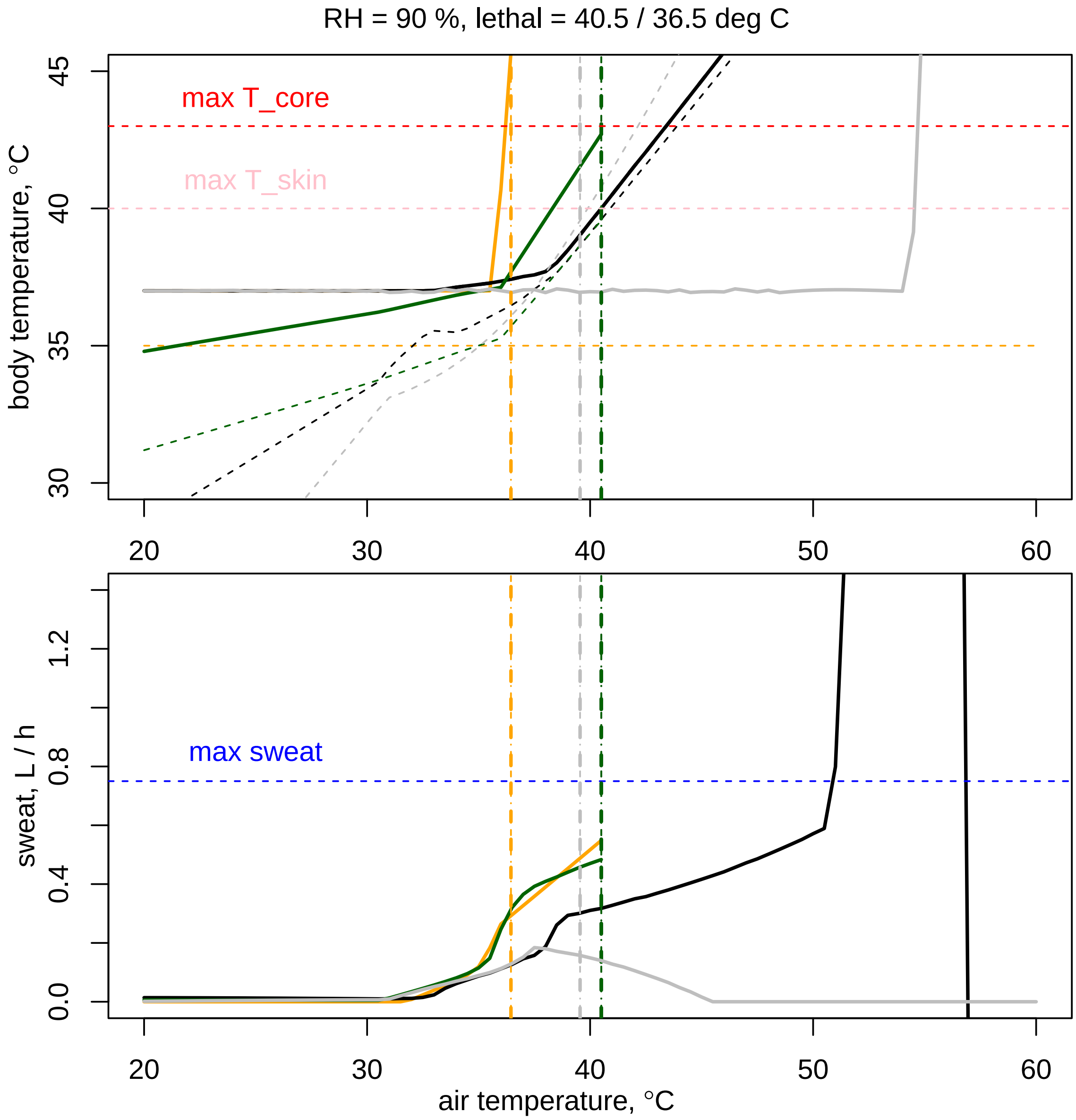
RH = 90 %, lethal = 40.5 / 36.5 deg C
max T_core
max T_skin
body temperature, °C
45
40
35
30
20
30
40
50
60
max sweat
sweat, L / h
1.2
0.8
0.4
0.0
air temperature, °C

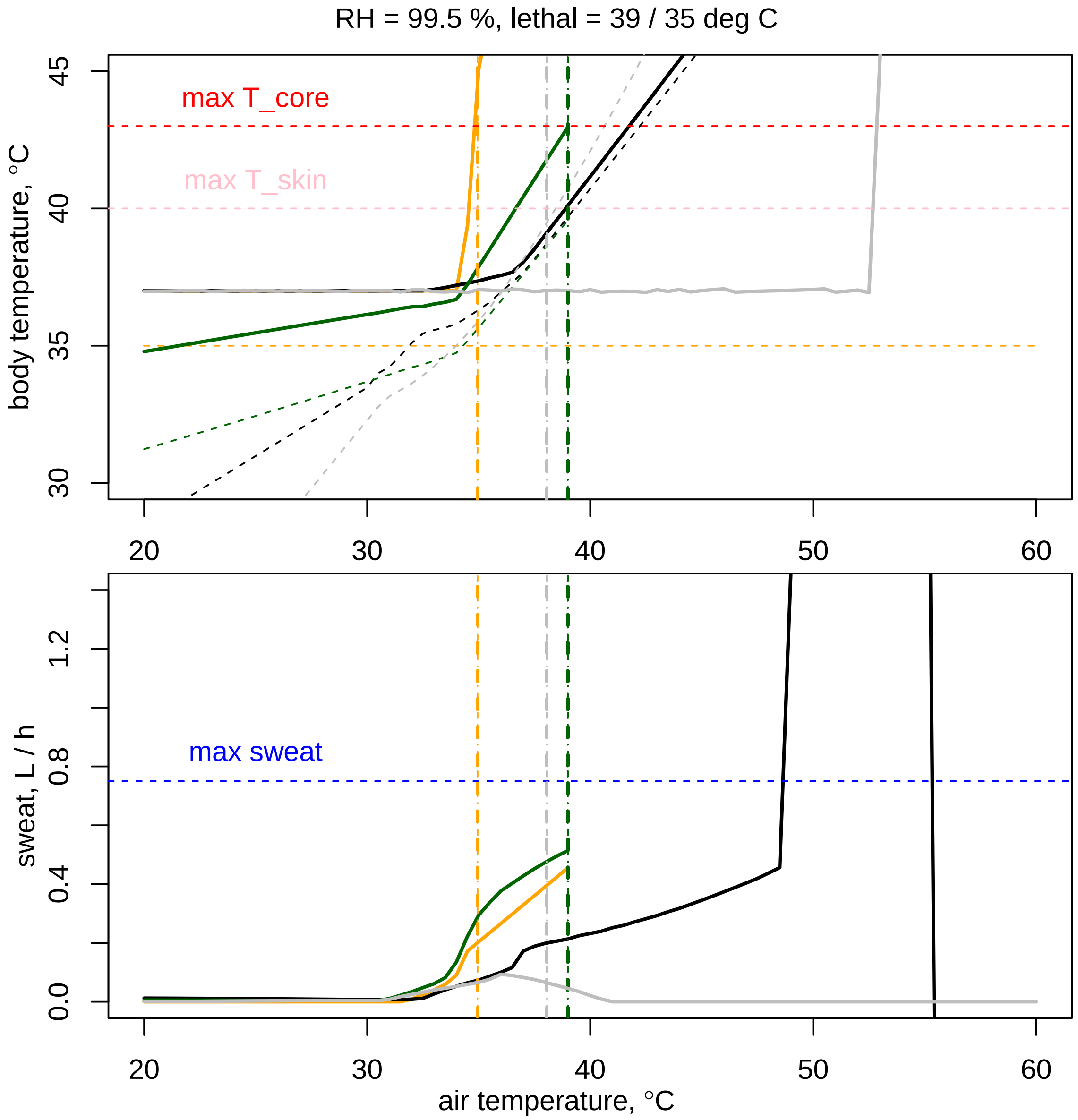


```
par(mfrow = c(1, 1))
par(oma = c(3, 1, 1, 1) + 0.1) # margin spacing
par(mar = c(3, 3, 1, 1) + 0.1) # margin spacing
par(mgp = c(2, 1, 0) ) # margin spacing
plot(Tw.DJs.35, ylim = c(0, 100), xlim = c(25, 60), xaxs = 'i', yaxs = 'i',
     type = 'l', col = 'lightblue', lwd = 3, lty = 5, ylab = 'humidity, %',
     xlab = expression('temperature, '*degree*C))
points(Tw.DJs.37, type = 'l', col = 'lightblue', lwd = 3, lty = 5)
points(Tw.DJs.39, type = 'l', col = 'lightblue', lwd = 3, lty = 5)
points(survive[, 1], humidities, col = 'black', lwd = 2, type = 'l',
       pch = 16, cex = 1.2)
points(survive[, 2], humidities, col = 'orange', lwd = 2, type = 'l',
       pch = 16, cex = 1.2)
if(plot.manmo){
```

```
  points(survive[, 3], humidities, col = 'grey', lwd = 2, type = 'l',
         pch = 16, cex = 1.2)
  points(survive[, 4], humidities, col = 'darkgreen', lwd = 2, type = 'l',
         pch = 16, cex = 1.2)
}

points(survive[, 5], humidities, col = 'black', lty = 2, lwd = 2, type = 'l',
       pch = 3, cex = 1.2)
points(survive[, 6], humidities, col = 'orange', lty = 2, lwd = 2, type = 'l',
       pch = 3, cex = 1.2)
if(plot.manmo){
  points(survive[, 7], humidities, col = 'grey', lty = 2, lwd = 2, type = 'l',
         pch = 3, cex = 1.2)
  points(survive[, 8], humidities, col = 'darkgreen', lty = 2, lwd = 2,
         type = 'l', pch = 3, cex = 1.2)
}

if(plot.legend){
  if(plot.manmo){
    legend(46, 100, legend = c('HomoTherm', 'MANMO', 'HHB', 'PHS', 'wetbulb'),
           lty = 1, col = c('black', 'grey', 'orange', 'darkgreen', 'lightblue'),
           bty = 'n', lwd = 2, cex = 0.8)
  }else{
    legend(46, 100, legend = c('HomoTherm', 'HHB', 'wetbulb'),
           lty = 1, col = c('black', 'orange', 'lightblue'),
           bty = 'n', lwd = 2, cex = 0.8)
  }
text(28, 22, 'limit')
legend(25, 20, legend = c('environment', 'sweat rate'), lty = c(1, 2), bty = 'n')

}
usr <- par("usr")  # plot limits
text(
  x = usr[2] + 0.02 * diff(usr[1:2]),
  y = c(18, 21.5, 25),
  labels = c("35", "37", "39"),
  col = "lightblue",
  cex = 0.75,
  xpd = NA
)
```

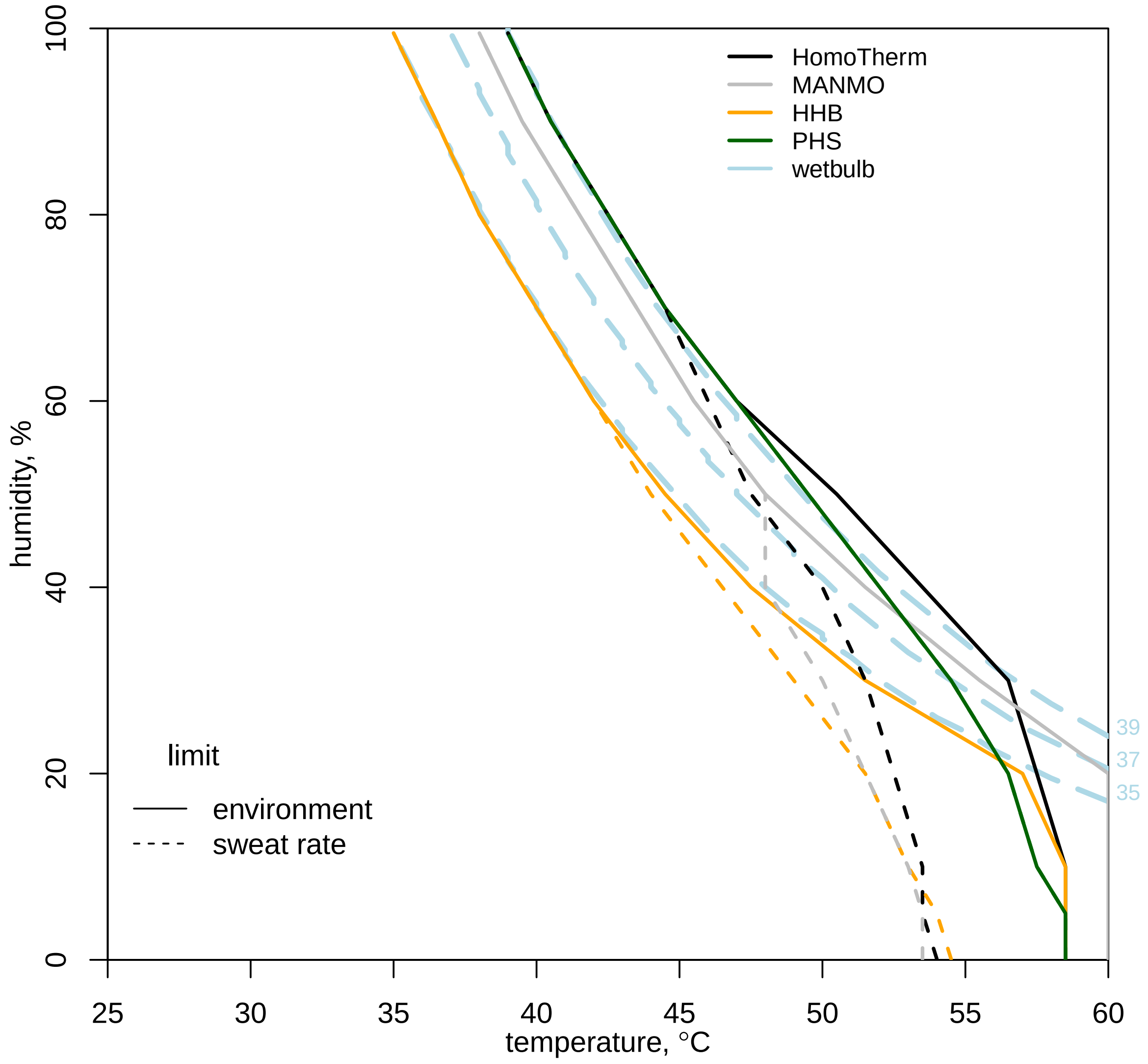

Now rerun, feeding the HomoTherm outputted skin temperature etc. as input to the HHB model.

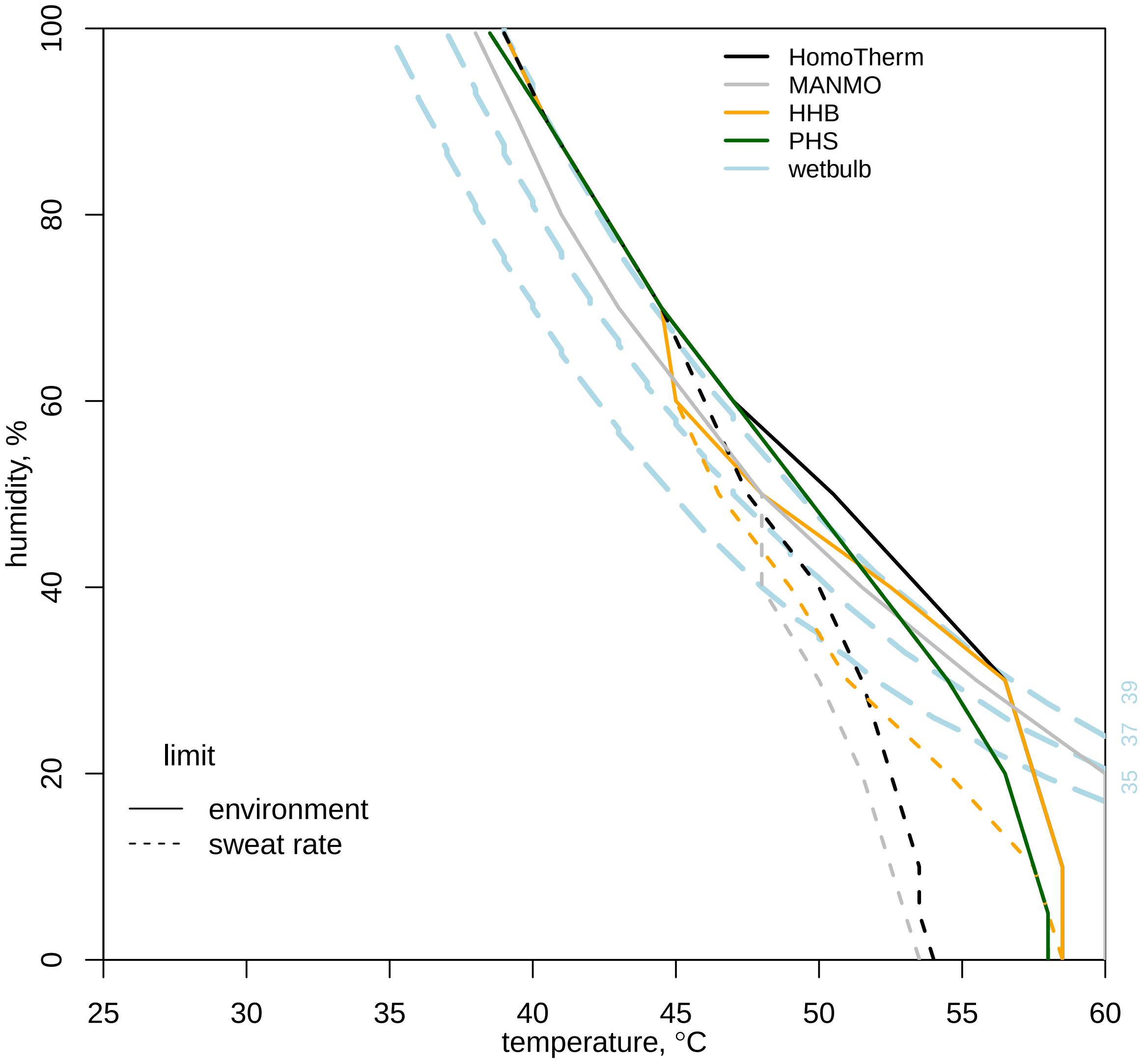

References

Malchaire, J., A. Piette, B. Kampmann, P. Mehnert, H. Gebhardt, G. Havenith, E. den Hartog, I. Holmer, K. Parsons, G. Alfano, and B. Griefahn. 2001. Development and validation of the predicted heat strain model. The Annals of Occupational Hygiene 45:123–135.

Myrup, L. O., and D. L. Morgan. 1972. Numerical model of the urban atmosphere. Volume I The city-surface interface. University of California, Davis.

Vanos, J., G. Guzman-Echavarria, J. W. Baldwin, C. Bongers, K. L. Ebi, and O. Jay. 2023. A physiological approach for assessing human survivability and liveability to heat in a changing climate. Nature Communications 14:7653.
