# Supplementary material for: HomoTherm: An Open‐Source Approach to Modelling Heat Exchange in Humans and Other Hominins in Diverse Environments
Source: Glob Chang Biol. 2026 Apr 1;32(4):e70830. doi: 10.1111/gcb.70830 (PMC13044332; doi:10.1111/gcb.70830)
Supplement: Supplementary file 7 — Appendix S7: gcb70830‐sup‐0007‐Appendix 7.pdf. [file GCB-32-e70830-s002.pdf]

Test of HomoTherm, Gagge and MANMO models against Hardy and Dubois 1937

Michael Kearney

2026-01-11

Overview

A test of the HomoTherm, Gagge (Gagge et al., 1971) and MANMO (Myrup and Morgan, 1971) models against the data reported in Hardy and Dubois (1937).

Load the libraries and data

```
library(NicheMapR)
library(readxl)
localpath <- 'c:/Users/mrke/Dropbox/Current Research Projects/mammal_projects/manmo analysis/'
source(paste0(localpath, 'code/MANMO/MANMO_R.R')) # the MANMO function
source(paste0(localpath, 'code/MANMO/run.MANMO.R'))
source(paste0(localpath, 'code/Gagge/gagge.R'))
source(paste0(localpath, 'code/HHB/HHB.R'))
source(paste0(localpath, 'code/HHB/run_HHB.R'))

Trectal_HD <- read_excel(
  path=paste0(localpath,
              "data/HardyDubois/Hardy and DuBois Fig2 T_rectal.xlsx"),
  sheet="Sheet1")
Tskin_HD <- read_excel(
  path=paste0(localpath,
              "data/HardyDubois/Hardy and DuBois Fig2 T_skin.xlsx"),
  sheet="Sheet1")
HeatLoss_HD <- read_excel(
  path=paste0(localpath,
              "data/HardyDubois/Hardy and DuBois Fig2 HeatLoss.xlsx"),
  sheet="Sheet1")
Vapourisation_HD <- read_excel(
  path=paste0(localpath,
              "data/HardyDubois/Hardy and DuBois Fig2 Vapourisation.xlsx"),
  sheet="Sheet1")
Conductivity_HD <- read_excel(
  path=paste0(localpath,
              "data/HardyDubois/Hardy and DuBois Fig2 Conductivity.xlsx"),
  sheet="Sheet1")
Fig1_HD <- read_excel(
  path=paste0(localpath,
```

```
              "data/HardyDubois/Hardy and DuBois Fig1.xlsx"),
  sheet="Sheet1")
```

Load the Hardy & Dubois observations

The R implementation of the HHB model includes all the functions in HHB.R and then run_HHB runs a calculation given the input parameters and variables.

Environmental conditions

```
# environmental variables
TAs <- seq(22, 36, 0.1) # air temperatures, deg C
WETAIR.out <- WETAIR(db = TAs, rh = 15) # assume air is min of 15% RH at max temp
RHs <- WETAIR.out$e[length(TAs)] / WETAIR.out$esat * 100
#RHs <- rep(10, length(TAs)) # relative humidities, %
VELs <- rep(0.076, length(TAs)) # wind speeds, m/s
```

Simulate Hardy

Hardy parameters

```
# person parameters
MASS <- 67 # MASS, kg
HEIGHT <- 168 # height, cm
AREA <- 0.00718 * MASS ^ 0.425 * HEIGHT ^ 0.725 # DuBois area, m2
QMETAB_REST <- 34.8 * 4184 / 3600 * AREA * 1.07 # basal metabolic rate, W
TC_RESTs <- rep(36.8, 4)
INSDEPDs <- c(0.01, 1e-9, 1e-9, 1e-9) # fur depth, dorsal (m)
INSDEPVs <- c(1e-9, 1e-9, 1e-9, 1e-9) # fur depth, ventral (m)
PCTBAREVAPs <- rep(90, 4)

plot_human(MASS = MASS,
           HEIGHT = HEIGHT,
           INSDEPDs = INSDEPDs,
           INSDEPVs = INSDEPVs)
```

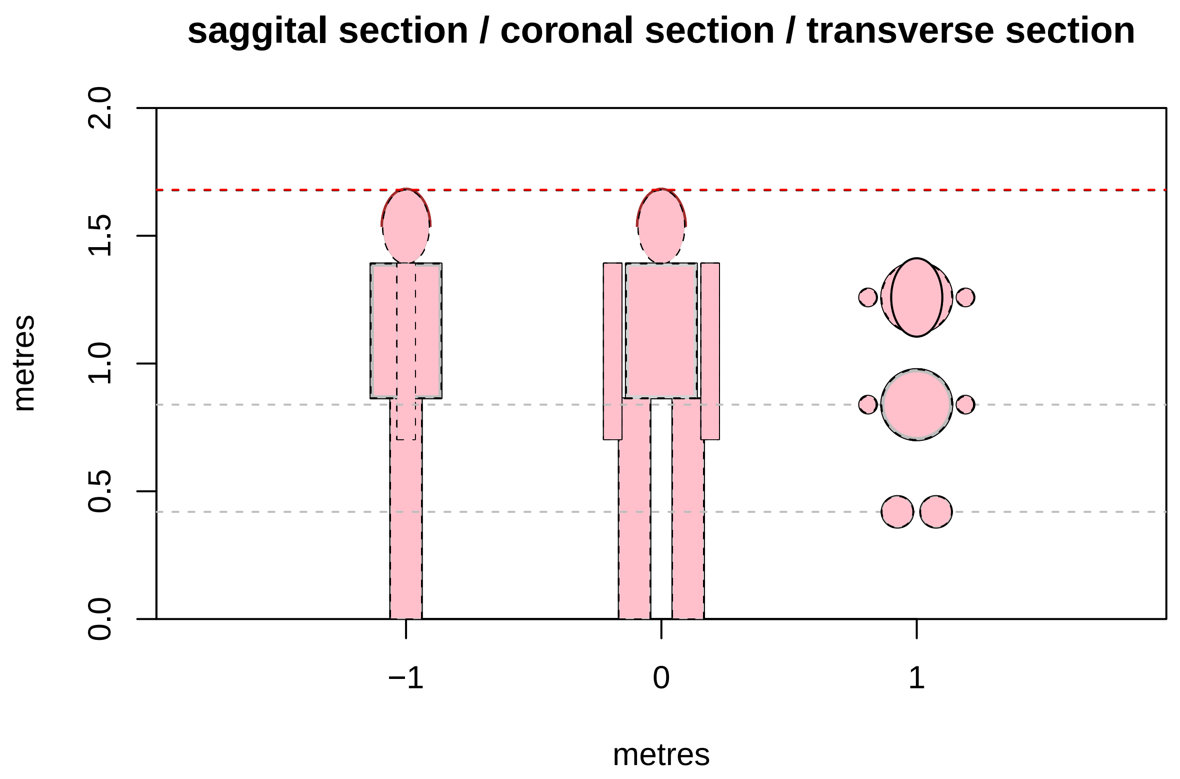


```
## [1] 1.678111
```

Run simulations.

```
# run HomoTherm simulation
HomoTherm.out <- HomoTherm_var(MASS = MASS,
                               QMETAB_REST = QMETAB_REST,
                               INSDEPDs = INSDEPDs,
                               INSDEPVs = INSDEPVs,
                               PCTBAREVAPs = PCTBAREVAPs,
                               TAs = TAs,
                               RHs = RHs,
                               VELs = VELs)
HomoTherm.Hardy <- HomoTherm.out$balance

# run MANMO simulation
getMR <- function(x){-3.2096E-03 * x ^ 4 + 3.5998E-01 * x ^ 3 - 1.4832E+01 *
    x ^ 2 + 2.6469E+02 * x ^ 1 - 1.6740E+03}
getTskinHardy <- function(x){-5.7936851E-04 * x ^ 4 + 6.1111159E-02 * x ^ 3 -
    2.3875866E+00 * x ^ 2 + 4.1297999E+01 * x ^ 1 - 2.3655855E+02}
G_m.G2s <- getMR(TAs) # used for MANMO
Tsk_Cs <- getTskinHardy(TAs)
clo <- colMeans(get_clo(HomoTherm.out, INSDEPDs = INSDEPDs, INSDEPVs = INSDEPVs))
MANMO.output <- run.MANMO(W = rep(1, length(TAs)) / 100,
                          Ht.H4 = HEIGHT,
```

```
                          Wt.W4 = MASS,
                          D3 = c(mean(INSDEPDs[2:4]), rep(1e-10, 3)),
                          G_m.G2s = G_m.G2s * 4184 / 3600,
                          CLO.C4 = clo,
                          K6 = 0.7,
                          CLO.mode = 0,
                          TAs = TAs,
                          RH.H2s = RHs / 100,
                          VELs = VELs)
MANMO.Hardy <- MANMO.output

# Gagge model
for(i in 1:length(TAs)){
  gagge.out <- gagge(W_0 = 1 / 100,
                     TA = TAs[i],
                     RH = RHs[i] / 100,
                     CLO = clo,
                     RM = G_m.G2s[i] * 4184 / 3600,
                     )
  if(i == 1){
    gagge.output <- gagge.out
  }else{
    gagge.output <- rbind(gagge.output, gagge.out)
  }
}
gagge.output <- as.data.frame(gagge.output)
gagge.Hardy <- gagge.output

HHB <- lapply(1:length(TAs),
              function(x){run_HHB(AD = AREA,
                                  M = G_m.G2s[x] * 4184 / 3600 * AREA,
                                  #M = HomoTherm.out$balance$QMETAB[x],
                                  Tsk_C = Tsk_Cs[x],
                                  Icl = clo,
                                  Ta_C = TAs[x],
                                  humidity = RHs[x],
                                  Av_ms = VELs[x],
                                  mrt_C = TAs[x],
                                  Mass = MASS
              )})
HHB.Hardy <- as.data.frame(do.call(rbind, HHB))
```

DuBois parameters

```
# person parameters
MASS <- 77.5 # mass, kg
HEIGHT <- 179 # height, cm
AREA <- 0.00718 * MASS ^ 0.425 * HEIGHT ^ 0.725 # DuBois area, m2
QMETAB_REST <- 34.9 * 4184 / 3600 * AREA * 1.07 # basal metabolic rate, W
TC_RESTs <- rep(37.1, 4)
getTskinDuBois <- function(x){3.3615452E-04 * x ^ 5 - 4.9218544E-02 * x ^ 4 +
    2.8668308E+00 * x ^ 3 - 8.3052783E+01 * x ^ 2 + 1.1973700E+03 * x ^ 1 -
    6.8451668E+03}
```

```
Tsk_Cs <- getTskinDuBois(TAs)
plot_human(MASS = MASS,
           HEIGHT = HEIGHT,
           INSDEPDs = INSDEPDs,
           INSDEPVs = INSDEPVs)
```

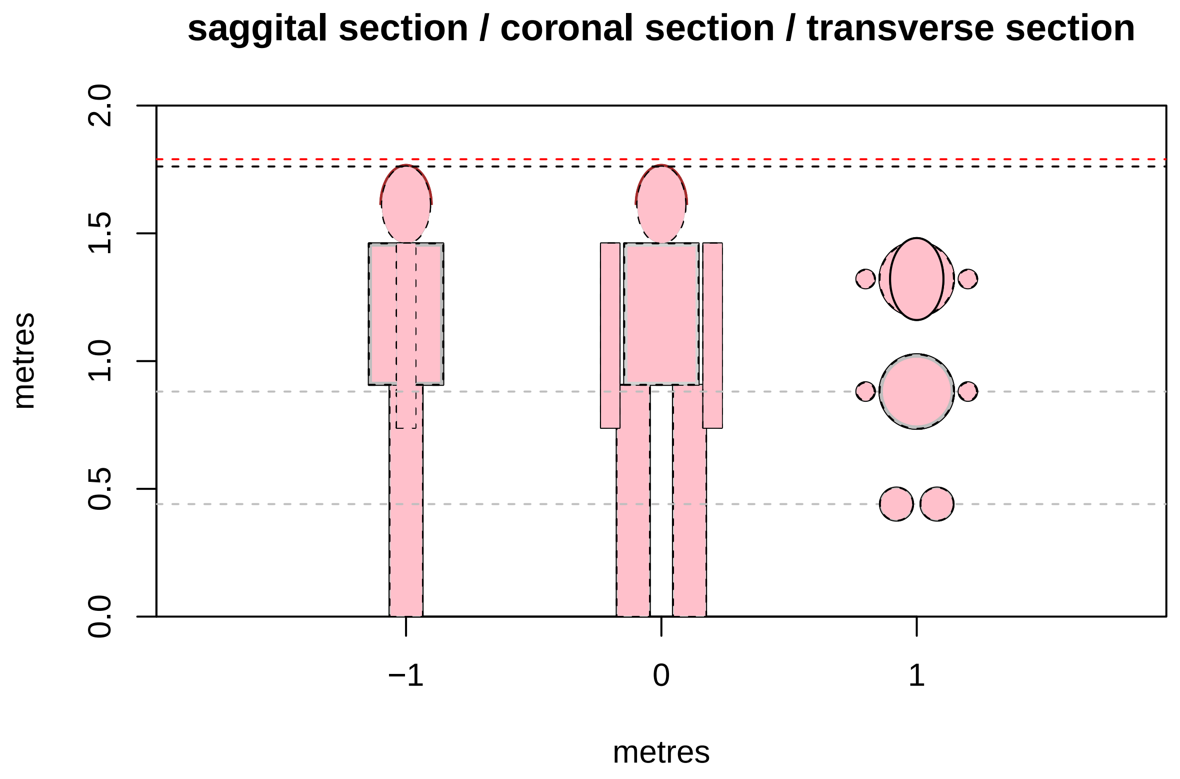


```
## [1] 1.761555
```

Run simulations.

```
# run HomoTherm simulation
HomoTherm.out <- HomoTherm_var(MASS = MASS,
                               QMETAB_REST = QMETAB_REST,
                               TC_RESTs = TC_RESTs,
                               INSDEPDs = INSDEPDs,
                               INSDEPVs = INSDEPVs,
                               PCTBAREVAPs = PCTBAREVAPs,
                               TAs = TAs,
                               RHs = RHs,
                               VELs = VELs)
HomoTherm.DuBois <- HomoTherm.out$balance

# run MANMO simulation
getMR <- function(x){-3.2096E-03 * x ^ 4 + 3.5998E-01 * x ^ 3 - 1.4832E+01 *
    x ^ 2 + 2.6469E+02 * x ^ 1 - 1.6740E+03}
```

```r
G_m.G2s <- getMR(TAs) # used for MANMO
clo <- colMeans(get_clo(HomoTherm.out, INSDEPDs = INSDEPDs, INSDEPVs = INSDEPVs))
MANMO.output <- run.MANMO(W = rep(1, length(TAs)) / 100,
                          Ht.H4 = HEIGHT,
                          Wt.W4 = MASS,
                          D3 = c(mean(INSDEPDs[2:4]), rep(1e-10, 3)),
                          G_m.G2s = G_m.G2s * 4184 / 3600,
                          CLO.C4 = clo,
                          K6 = 0.7,
                          CLO.mode = 0, # assume clothing temp = skin temp
                          TAs = TAs,
                          RH.H2s = RHs / 100,
                          VELs = VELs)
MANMO.DuBois <- MANMO.output

# Gagge model
for(i in 1:length(TAs)){
  gagge.out <- gagge(W_0 = 1 / 100,
                     TA = TAs[i],
                     RH = RHs[i] / 100,
                     CLO = clo,
                     RM = G_m.G2s[i] * 4184 / 3600,
                     )
  if(i == 1){
    gagge.output <- gagge.out
  }else{
    gagge.output <- rbind(gagge.output, gagge.out)
  }
}
gagge.output <- as.data.frame(gagge.output)
gagge.DuBois <- gagge.output

HHB <- lapply(1:length(TAs),
              function(x){run_HHB(AD = AREA,
                                  M = G_m.G2s[x] * 4184 / 3600 * AREA,
                                  #M = HomoTherm.out$balance$QMETAB[x],
                                  Tsk_C = Tsk_Cs[x],
                                  Icl = clo,
                                  Ta_C = TAs[x],
                                  humidity = RHs[x],
                                  Av_ms = VELs[x],
                                  mrt_C = TAs[x],
                                  Mass = MASS
              )})
HHB.DuBois <- as.data.frame(do.call(rbind, HHB))
```

Plot results.

```r
par(mfrow = c(2, 2))
par(oma = c(2, 1, 1, 1) + 0.1) # margin spacing
par(mar = c(4, 4, 1, 1) + 0.1) # margin spacing
par(mgp = c(2, 1, 0) ) # margin spacing
```

```r
with(subset(Tskin_HD, Subject == "Hardy"),
     plot(Tenv, T_skin, ylim = c(28, 38), xlim = c(22, 35),
          ylab = "temperature, °C", xlab = "temperature, °C", col = 'red',
          pch = 16, cex = 1.25, main = 'skin temperature'))
points(TAs, MANMO.Hardy$Tskin, col = "grey", lty = 1, type = 'l', lwd = 1.5)
points(TAs, HomoTherm.Hardy$T_SKIN, col = "black", pch = 16, type = 'l', lwd = 1.5)
points(TAs, gagge.Hardy$TSK, col = "black", pch = 16, lty = 3, type = 'l',
       lwd = 1.5)
#points(TAs, getTskinHardy(TAs), col = "orange", pch = 16, type = 'l', lwd = 1.5)
legend(22, 38, cex = 0.8,
       legend = c('Observed', 'HomoTherm', 'MANMO', 'Gagge',  'HHB'),
       col = c('red','black', 'grey', 'black', 'orange'),
       pch = c(16, NA, NA, NA, NA), lty = c(NA, 1, 1, 3, 1), bty = 'n', ncol = 1)

with(subset(Trectal_HD, Subject == "Hardy"),
     plot(Tenv, T_rectal, ylim = c(36, 38), xlim = c(22, 35),
          ylab = "temperature, °C", xlab = "temperature, °C", col = 'red',
          pch = 16, cex = 1.25, main = 'core temperature'))
points(TAs, HomoTherm.Hardy$T_CORE, col = "black", pch = 16, type = 'l', lwd = 1.5)

with(subset(HeatLoss_HD, Subject == "Hardy"),
     plot(Tenv, HeatLoss, ylim = c(30, 60), xlim = c(22, 35),
          ylab = "Cal / m2 / h", xlab = "temperature, °C", col = 'red',
          pch = 16, cex = 1.25, main = 'metabolic rate'))
#points(TAs, MANMO.Hardy$M_m.M / MANMO.Hardy$A.A2 * 3600 / 4184,
#       col = "grey", lty = 1, type = 'l', lwd = 1.5)
points(TAs, HomoTherm.Hardy$QMETAB / MANMO.Hardy$A.A2 * 3600 / 4184,
       col = "black", pch = 16, type = 'l', lwd = 1.5)

with(subset(Vapourisation_HD, Subject == "Hardy"),
     plot(Tenv, Vapourisation, ylim = c(0, 150), xlim = c(22, 35),
          ylab = "g H2O / h", xlab = "temperature, °C", col = 'red',
          pch = 16, cex = 1.25, main = 'evaporation'))
points(TAs, MANMO.Hardy$evap.L.h * 1000, col = "grey", lty = 1, type = 'l',
       lwd = 1.5)
points(TAs, (HomoTherm.Hardy$SWEAT_L + HomoTherm.Hardy$EVAP_RESP_L) * 1000,
       col = "black", pch = 16, type = 'l', lwd = 1.5)
HTOVPR <- 2.5012E+06 - 2.3787E+03 * TAs
points(TAs, gagge.Hardy$EV * 2 * 3600 * 1000 / HTOVPR, col = "black", lty = 3,
       pch = 16, type = 'l', lwd = 1.5)
points(TAs, HHB.Hardy$Sreq * 1000, col = "orange", pch = 16, type = 'l', lwd = 1.5)
mtext("Hardy data", side = 3, line = 0, outer = TRUE)
```

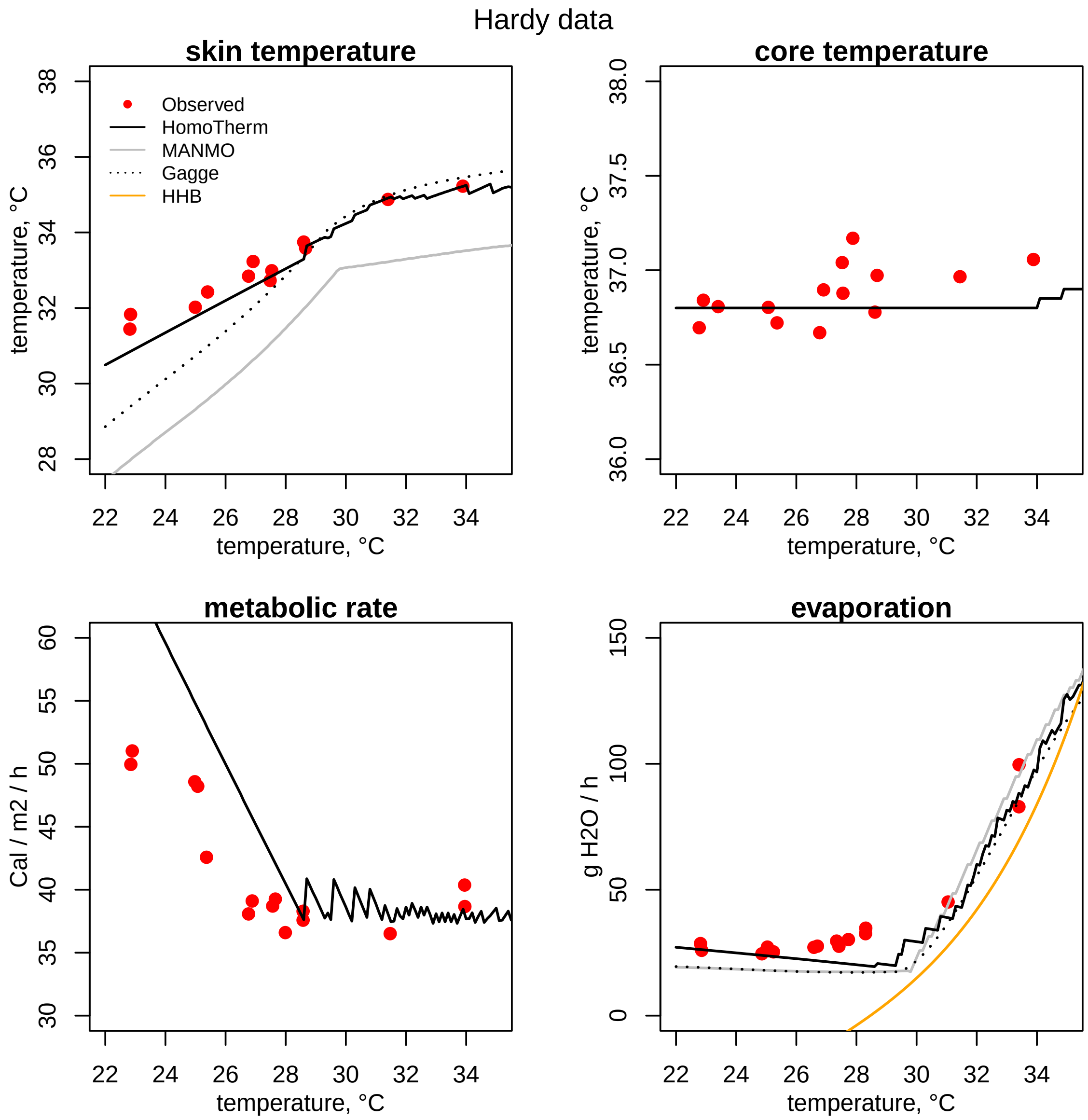


```r
with(subset(Tskin_HD, Subject == "DuBois"),
     plot(Tenv, T_skin, ylim = c(28, 38), xlim = c(22, 35), ylab = "temperature,
          °C", xlab = "temperature, °C", col = 'red', pch = 16, cex = 1.25,
          main = 'skin temperature'))
points(TAs, MANMO.DuBois$Tskin, col = "grey", lty = 1, type = 'l', lwd = 1.5)
points(TAs, gagge.Hardy$TSK, col = "black", lty = 3, pch = 16, type = 'l',
       lwd = 1.5)
points(TAs, HomoTherm.DuBois$T_SKIN, col = "black", pch = 16, type = 'l',
       lwd = 1.5)
#points(TAs, getTskinDuBois(TAs), col = "orange", pch = 16, type = 'l', lwd = 1.5)
legend(22, 38, cex = 0.8,
       legend = c('Observed', 'HomoTherm', 'MANMO', 'Gagge',  'HHB'),
       col = c('red','black', 'grey', 'black', 'orange'),
       pch = c(16, NA, NA, NA, NA), lty = c(NA, 1, 1, 3, 1), bty = 'n', ncol = 1)
```

```r
with(subset(Trectal_HD, Subject == "DuBois"),
     plot(Tenv, T_rectal, ylim = c(36, 38), xlim = c(22, 35), ylab = "temperature,
          °C", xlab = "temperature, °C", col = 'red', pch = 16, cex = 1.25,
          main = 'core temperature'))
points(TAs, HomoTherm.DuBois$T_CORE, col = "black", pch = 16, type = 'l', lwd = 1.5)

with(subset(HeatLoss_HD, Subject == "DuBois"),
     plot(Tenv, HeatLoss, ylim = c(30, 60), xlim = c(22, 35),
          ylab = "Cal / m2 / h", xlab = "temperature, °C", col = 'red',
          pch = 16, cex = 1.25, main = 'metabolic rate'))
#points(TAs, MANMO.DuBois$M_m.M / MANMO.DuBois$A.A2 * 3600 / 4184,
#        col = "grey", lty = 1, type = 'l', lwd = 1.5)
points(TAs, HomoTherm.DuBois$QMETAB / MANMO.DuBois$A.A2 * 3600 / 4184,
       col = "black", pch = 16, type = 'l', lwd = 1.5)

with(subset(Vapourisation_HD, Subject == "DuBois"),
     plot(Tenv, Vapourisation, ylim = c(0, 150), xlim = c(22, 35),
          ylab = "g H2O / h", xlab = "temperature, °C", col = 'red',
          pch = 16, cex = 1.25, main = 'evaporation'))
points(TAs, MANMO.DuBois$evap.L.h * 1000, col = "grey", lty = 1, type = 'l',
       lwd = 1.5)
points(TAs, (HomoTherm.DuBois$SWEAT_L + HomoTherm.DuBois$EVAP_RESP_L) * 1000,
       col = "black", pch = 16, type = 'l', lwd = 1.5)
points(TAs, gagge.DuBois$EV * 2 * 3600 * 1000 / HTOVPR, col = "black", lty = 3,
       pch = 16, type = 'l', lwd = 1.5)
points(TAs, HHB.DuBois$Sreq * 1000, col = "orange", pch = 16, type = 'l', lwd = 1.5)
mtext("DuBois data", side = 3, line = 0, outer = TRUE)
```

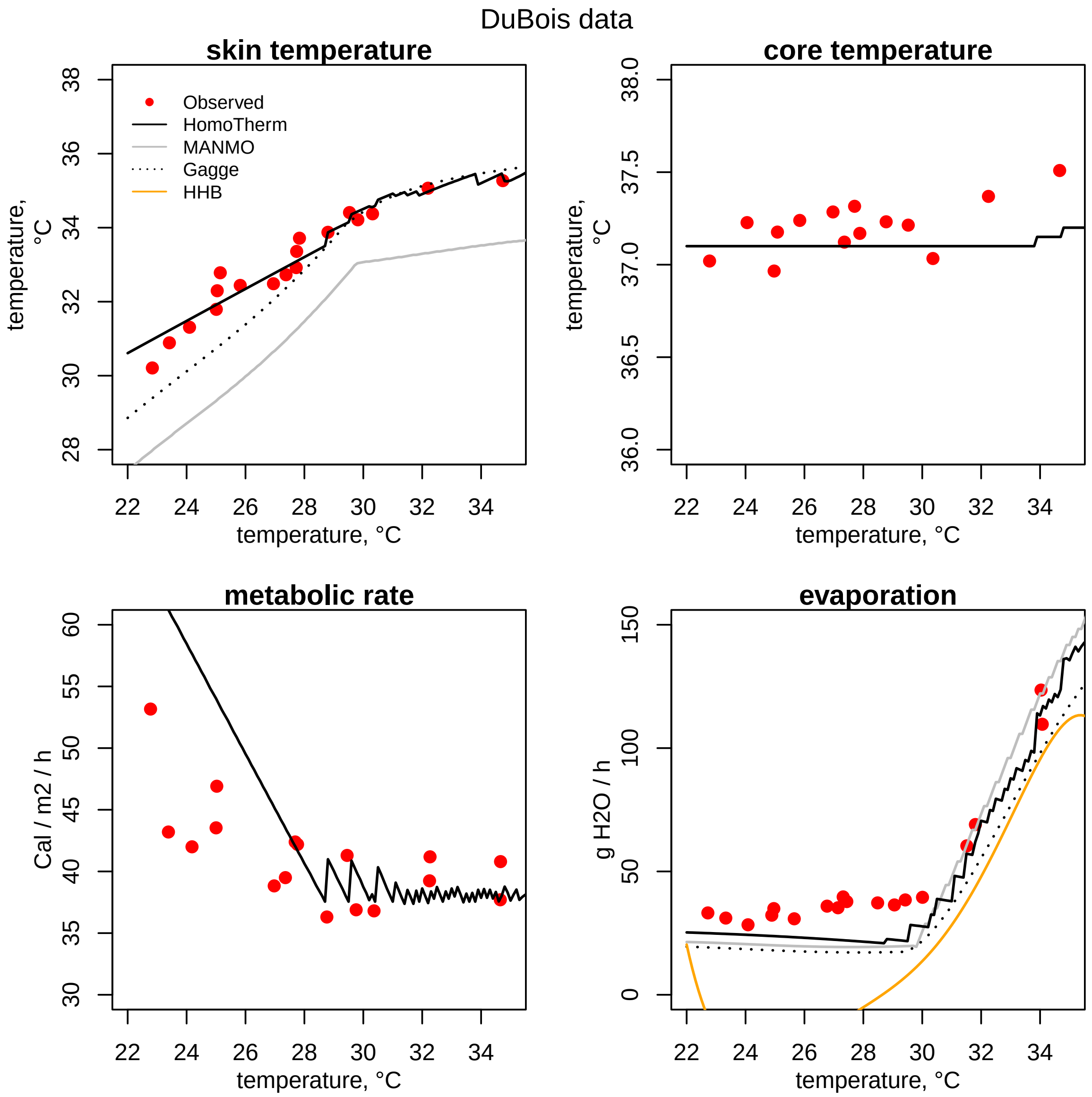


```r
par(mfrow = c(2, 2))
par(oma = c(2, 1, 1, 1) + 0.1) # margin spacing
par(mar = c(4, 4, 1, 1) + 0.1) # margin spacing
par(mgp = c(2, 1, 0) ) # margin spacing
with(subset(Fig1_HD, Subject == "Hardy"),
     plot(Tenv, `Evap&Conv&Rad`, ylim = c(40, 120), xlim = c(22,35),
          col = 'red', pch = 16, cex = 1.25, ylab = 'Metabolism, Cal / h'))
# with(MANMO.Hardy, points(TAs, M_m.M * 3600 / 4184, col = 'grey', lty = 1,
#                          type = 'l', lwd = 1.5))
with(HomoTherm.Hardy, points(TAs, QMETAB * 3600 / 4184, col = 'black',
                             pch = 16, type = 'l', lwd = 1.5))
legend(28, 125, cex = 0.8, legend = c('Observed', 'HomoTherm', 'MANMO', 'HHB'),
       col = c('red','black', 'grey', 'orange'), pch = c(16, NA, NA, NA),
       lty = c(NA, 1, 1, 1), bty = 'n', ncol = 1)
```

```r
mtext("Hardy data", side = 3, line = 0, outer = TRUE)

with(subset(Fig1_HD, Subject == "Hardy"),
     plot(Tenv, Evaporation, ylim = c(0, 100), xlim = c(22,35), col = 'red',
          pch = 16, cex = 1.25, ylab = 'Evaporation, Cal / h'))
with(MANMO.Hardy, points(TAs, E_m.E * 3600 / 4184 * -1, col = 'grey', lty = 1,
                         type = 'l', lwd = 1.5))
with(HomoTherm.Hardy, points(TAs, (QEVAP_CUT + QEVAP_RESP) * 3600 / 4184 * -1,
                             col = 'black', pch = 16, type = 'l', lwd = 1.5))
with(gagge.Hardy, points(TAs, EV * 3600 / 4184 * 2, col = "black", lty = 3,
                         pch = 16, type = 'l', lwd = 1.5))
with(HHB.Hardy, points(TAs, Ereq * 3600 / 4184, col = 'orange', pch = 16,
                       type = 'l', lwd = 1.5))

with(subset(Fig1_HD, Subject == "Hardy"),
     plot(Tenv, Radiation, ylim = c(0, 100), xlim = c(22,35), col = 'red',
          pch = 16, cex = 1.25, ylab = 'Radiation, Cal / h'))
with(MANMO.Hardy, points(TAs, I_m.I * 3600 / 4184 * -1, col = 'grey', lty = 1,
                         type = 'l', lwd = 1.5))
with(HomoTherm.Hardy, points(TAs, (QRAD_IN - QRAD_OUT) * 3600 / 4184 * -1,
                             col = 'black', pch = 16, type = 'l', lwd = 1.5))

with(subset(Fig1_HD, Subject == "Hardy"),
     plot(Tenv, Convection, ylim = c(0, 100),
          xlim = c(22,35), col = 'red', pch = 16, cex = 1.25,
          ylab = 'Convection, Cal / h'))
with(MANMO.Hardy, points(TAs, H_m.H * -1, col = 'grey', lty = 1, type = 'l',
                         lwd = 1.5))
with(HomoTherm.Hardy, points(TAs, (QCONV + QCONV_RESP) * 3600 / 4184 * -1,
                             col = 'black', pch = 16, type = 'l', lwd = 1.5))
```

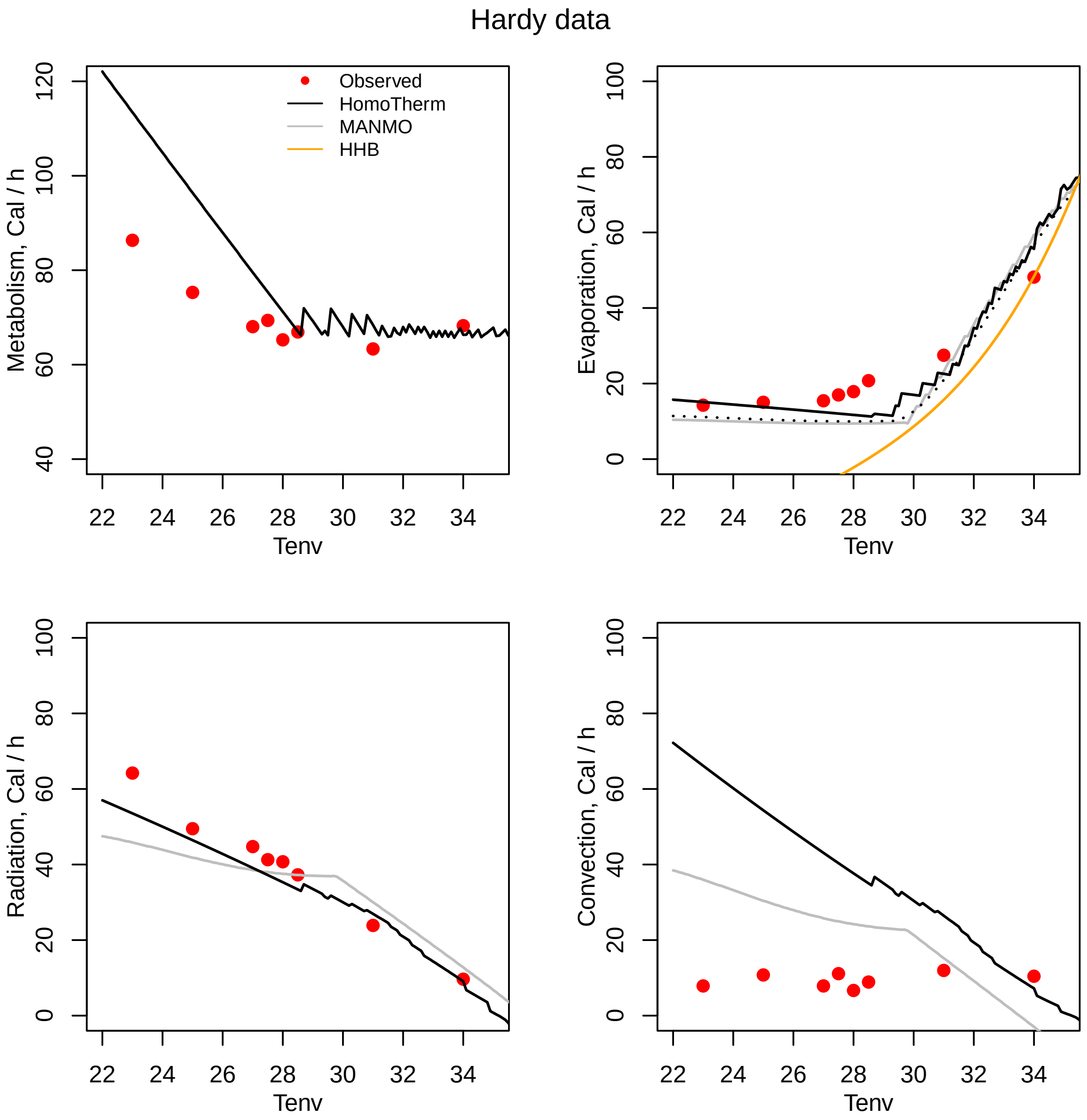


```
with(subset(Fig1_HD, Subject == "DuBois"),
     plot(Tenv, `Evap&Conv&Rad`, ylim = c(40, 120), xlim = c(22,35), col = 'red',
          pch = 16, cex = 1.25, ylab = 'Metabolism, Cal / h'))
# with(MANMO.DuBois, points(TAs, M_m.M * 3600 / 4184, col = 'grey', lty = 1,
#                           type = 'l', lwd = 1.5))
with(HomoTherm.DuBois, points(TAs, QMETAB * 3600 / 4184, col = 'black',
                              pch = 16, type = 'l', lwd = 1.5))
mtext("DuBois data", side = 3, line = 0, outer = TRUE)

with(subset(Fig1_HD, Subject == "DuBois"),
     plot(Tenv, Evaporation, ylim = c(0, 100), xlim = c(22,35), col = 'red',
          pch = 16, cex = 1.25, ylab = 'Evaporation, Cal / h'))
with(MANMO.DuBois, points(TAs, E_m.E * 3600 / 4184 * -1, col = 'grey', lty = 1,
                          type = 'l', lwd = 1.5))
```

```r
with(HomoTherm.DuBois,
     points(TAs, (QEVAP_CUT + QEVAP_RESP) * (3600 / 4184) * -1, col = 'black',
            pch = 16, type = 'l', lwd = 1.5))
with(gagge.DuBois, points(TAs, EV * 3600 / 4184 * 2, col = "black", lty = 3,
                          pch = 16, type = 'l', lwd = 1.5))
with(HHB.DuBois, points(TAs, Ereq * 3600 / 4184, col = 'orange', pch = 16,
                        type = 'l', lwd = 1.5))

with(subset(Fig1_HD, Subject == "DuBois"),
     plot(Tenv, Radiation, ylim = c(0, 100), xlim = c(22,35), col = 'red',
          pch = 16, cex = 1.25, ylab = 'Radiation, Cal / h'))
with(MANMO.DuBois, points(TAs, I_m.I * 3600 / 4184 * -1, col = 'grey', lty = 1,
                          type = 'l', lwd = 1.5))
with(HomoTherm.DuBois, points(TAs, (QRAD_IN - QRAD_OUT) * 3600 / 4184 * -1,
                              col = 'black', pch = 16, type = 'l', lwd = 1.5))

with(subset(Fig1_HD, Subject == "DuBois"),
     plot(Tenv, Convection, ylim = c(0, 100), xlim = c(22,35), col = 'red',
          pch = 16, cex = 1.25, ylab = 'Convection, Cal / h'))
with(MANMO.DuBois, points(TAs, H_m.H * 3600 / 4184 * -1, col = 'grey', lty = 1,
                          type = 'l', lwd = 1.5))
with(HomoTherm.DuBois, points(TAs, (QCONV + QCONV_RESP) * 3600 / 4184 * -1,
                              col = 'black', pch = 16, type = 'l', lwd = 1.5))
```

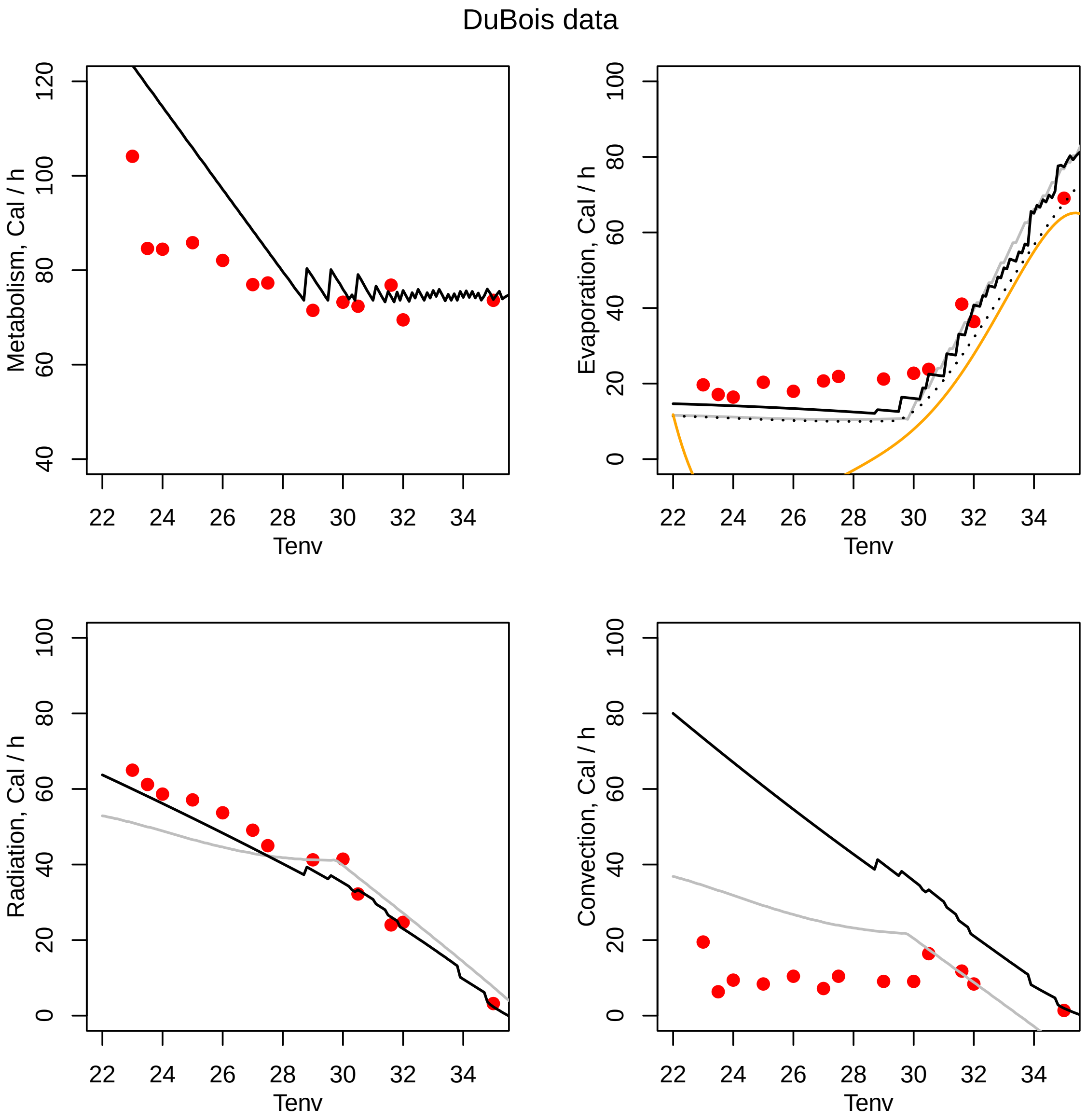

Finally, plot dry heat exchange for each subject.

```
par(mfrow = c(2, 1))
par(oma = c(2, 1, 1, 1) + 0.1) # margin spacing
par(mar = c(4, 4, 1, 1) + 0.1) # margin spacing
par(mgp = c(2, 1, 0) ) # margin spacing
with(subset(Fig1_HD, Subject == "Hardy"),
     plot(Tenv, Radiation + Convection, ylim = c(0, 120), xlim = c(22,35),
          col = 'red', pch = 16, cex = 1.25, xlab = 'temperature, °C',
          ylab = 'dry heat, Cal / h', main = 'Hardy'))
with(MANMO.Hardy, points(TAs, (I_m.I + H_m.H) * (3600 / 4184) * -1,
                         col = 'grey', lty = 1, type = 'l', lwd = 1.5))
with(HomoTherm.Hardy,
     points(TAs, -(QCONV + QCONV_RESP + QRAD_IN - QRAD_OUT) * (3600 / 4184),
```

```r
            col = 'black', pch = 16, type = 'l', lwd = 1.5))
with(HHB.Hardy,
     points(TAs, Dry_Heat_Loss * (3600 / 4184), col = 'orange', pch = 16,
            type = 'l', lwd = 1.5))
legend(26, 125, cex = 0.8, legend = c('Observed', 'HomoTherm', 'MANMO', 'HHB'),
       col = c('red', 'black', 'grey', 'orange'), pch = c(16, NA, NA, NA),
       lty = c(NA, 1, 1, 1), bty = 'n', ncol = 1)
#mtext("dry heat loss", side = 3, line = 0, outer = TRUE)

with(subset(Fig1_HD, Subject == "DuBois"),
     plot(Tenv, Radiation + Convection, ylim = c(0, 120), xlim = c(22,35),
          col = 'red', pch = 16, cex = 1.25, xlab = 'temperature, °C',
          ylab = 'dry heat, Cal / h', main = 'DuBois'))
with(MANMO.DuBois,
     points(TAs, (I_m.I + H_m.H) * (3600 / 4184) * -1, col = 'grey', lty = 1,
            type = 'l', lwd = 1.5))
with(HomoTherm.DuBois,
     points(TAs, -(QCONV + QCONV_RESP + QRAD_IN - QRAD_OUT) * (3600 / 4184),
            col = 'black', pch = 16, type = 'l', lwd = 1.5))
with(HHB.DuBois,
     points(TAs, Dry_Heat_Loss * (3600 / 4184), col = 'orange', pch = 16,
            type = 'l', lwd = 1.5))
```

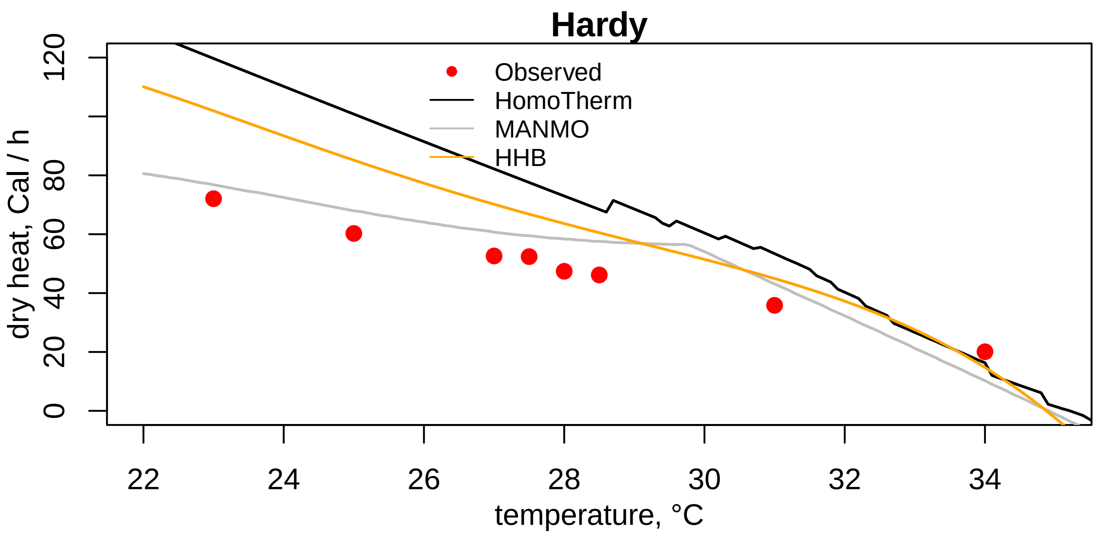

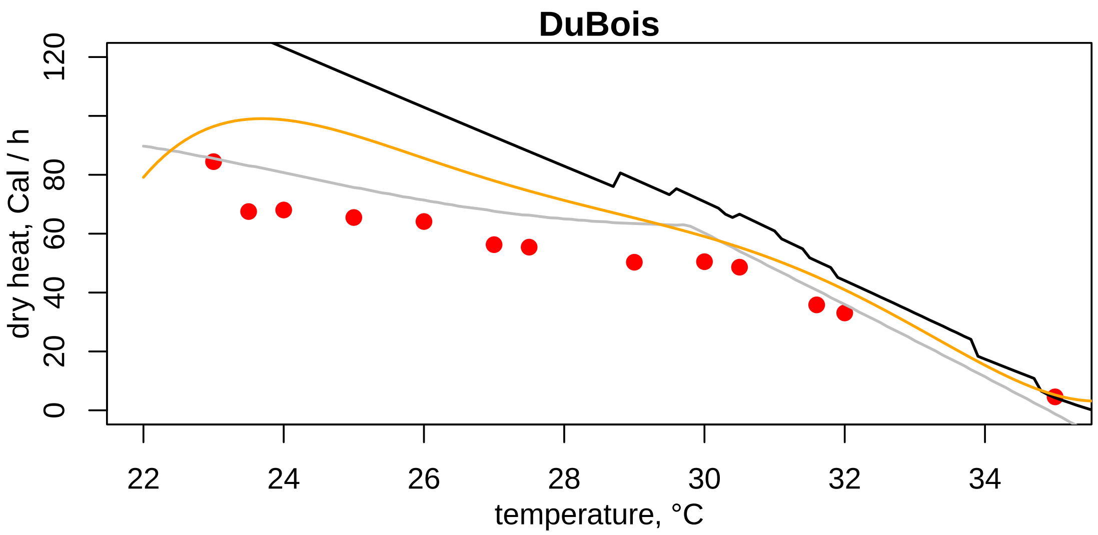


```
# legend(26, 125, cex = 0.8, legend = c('Observed', 'HomoTherm', 'MANMO', 'HHB'),
#        col = c('red','black', 'grey', 'orange'), pch = c(16, NA, NA, NA),
#        lty = c(NA, 1, 1, 1), bty = 'n', ncol = 1)
```

References

Gagge, A. P., J. A. J. Stolwijk, and Y. Nishi. 1971. An effective temperature scale based on a simple model of human physiological regulatory response. ASHRAE Transactions 77:247–262.

Hardy, J. D., & DuBois, E. F. (1937). Regulation of heat loss from the human body. Proceedings of the National Academy of Sciences, 23(12), 624–631.

Myrup, L. O., and D. L. Morgan. 1972. Numerical model of the urban atmosphere. Volume I The city-surface interface. University of California, Davis.
