# Supplementary material for: HomoTherm: An Open‐Source Approach to Modelling Heat Exchange in Humans and Other Hominins in Diverse Environments
Source: Glob Chang Biol. 2026 Apr 1;32(4):e70830. doi: 10.1111/gcb.70830 (PMC13044332; doi:10.1111/gcb.70830)
Supplement: Supplementary file 8 — Appendix S8: gcb70830‐sup‐0008‐Appendix 8.pdf. [file GCB-32-e70830-s012.pdf]

Test of HomoTherm, MANMO and HHB models against Winslow et al. 1936

Michael Kearney

2026-01-09

Overview

A test of the HomoTherm, MANMO (Myrup and Morgan, 1937) and HHB (Vanos et al., 2023) models against the data reported in Winslow et al. (1936). A set of hourly exposures of different air temperature and radiation combinations were applied to two nude subjects sitting in a chair. The two subjects were of very different build: subject I was pycnic (stout) and subject II was leptosomic (skinny).

The environments were set up to comply with the Linearity Criterion, whereby the relationship between radiation loss and the sum of metabolic rate, evaporation and storage is linear. Other data was collected under colder conditions that violated the Linearity Criterion which means that changes in core temperature don't fully capture the storage due to peripheral cooling. This data is dealt with in the test against the Winslow et al. (1937) data. Data warmer than the linear zone are where dripping occurs so the mass loss isn't fully representative of evaporation.

Considerable thought was given to determining the effective area for radiative exchange A_R, found to be around 1.6 for the stout subject and 1.1 for the skinny subject, which was about 75% of the total surface area.

Load the libraries and data

```
library(NicheMapR)
localpath <- 'c:/Users/mrke/Dropbox/Current Research Projects/mammal_projects/manmo analysis/'
source(paste0(localpath, '/code/MANMO/MANMO_R.R')) # the MANMO function
source(paste0(localpath, '/code/MANMO/run.MANMO.R'))
source(paste0(localpath, '/code/HHB/HHB.R'))
source(paste0(localpath, '/code/HHB/run_HHB.R'))
source(paste0(localpath, 'code/PHS/calcIso7933_Tcl.R'))
```

Load the Winslow et al. observations

Data columns are: 1) subject (I or II) 2) group (different air movement levels) 3) condition (groups based on combinations of air and radiant temperature, with combinations 3 to 6 and 8 to 10 meeting the Linearity Criterion as reported in Gagge (1936)) 4) vel (wind speed, ft/min) 5) RH (relative humidity, %) 6) T__A (air temperature, deg C) 7) T__W (wall temperature, deg C) 8) T__S (skin temperature, deg C) 9) deltaMASS (change in body weight, g/min) 10) deltaTB (change in body temperature deg C/hr) 11) M (metabolic rate, kg-calories/hr = kilocalories/hr) 12) S (storage rate, kg-calories/hr, negative = gain) 13) E (evaporation rate, kg-calories/hr, negative = loss) 14) R (radiation rate, kg-calories/hr, negative = loss) 15) C (metabolic rate, kg-calories/hr, negative = loss)

```
obs <- read.csv('c:/Users/mrke/Dropbox/Current Research Projects/mammal_projects/manmo analysis/data/Wi
obs$vel[is.na(obs$vel)] <- 20 # authors note this was probably the speed
obs1 <- subset(obs, subject == 1)
obs2 <- subset(obs, subject == 2)
```

Environmental conditions

Set up environmental conditions and compute operative temperatures as the independent variable based on coefficients from Winslow et al (1937) for these two subjects.

```
# environmental variables
TAs1 <- obs1$T_A # air temperatures, deg C
TRADs1 <- obs1$T_W # radiant temperatures, deg C
QGENs1 <- obs1$M * 4184 / 3600 # metabolic rate, W
RHs1 <- obs1$RH # relative humidities, %
VELs1 <- obs1$vel * 0.3048 / 60 # wind speeds, from ft/min to m/s
K_R1 <- 8.8 # from Windslow et al. 1937
K_C1 <- 9.2 # from Windslow et al. 1937
TOs1 <- (K_R1 * TRADs1 + K_C1 * TAs1) / (K_R1 + K_C1)

TAs2 <- obs2$T_A # air temperatures, deg C
TRADs2 <- obs2$T_W # radiant temperatures, deg C
QGENs2 <- obs2$M * 4184 / 3600 # metabolic rate, W
RHs2 <- obs2$RH # relative humidities, %
VELs2 <- obs2$vel * 0.3048 / 60 # wind speeds, from ft/min to m/s
K_R2 <- 6.3 # from Windslow et al. 1937
K_C2 <- 7.4 # from Windslow et al. 1937
TOs2 <- (K_R2 * TRADs2 + K_C2 * TAs2) / (K_R2 + K_C2)
```

Simulate Person 1

First set up parameters for the stout person.

```
# person parameters
MASS <- 104.326 # MASS, kg
HEIGHT <- 170.18 # height, cm
AREA <- 0.00718 * MASS ^ 0.425 * HEIGHT ^ 0.725 # DuBois area, m2
QMETAB_REST <- quantile(obs1$M, 0.1) * 4184 / 3600 # basal metabolic rate, W
INSDEPDs <- c(0.01, 0, 0, 0) # fur depth, dorsal (m)
INSDEPVs <- c(0, 0, 0, 0) # fur depth, ventral (m)
PCTBAREVAPs <- rep(90, 4)
MASSFRACs <- c(0.0761, 0.501, 0.049, 0.162)

SHAPE_Bs <- c(1.6, 1.73, 10, 5)#c(1.6, 1.9, 11, 7.0)
shapes <- GET_SHAPES(MASSs = MASS * MASSFRACs,
                     AREA = AREA,
                     SHAPE_Bs = SHAPE_Bs,
                     SHAPE_Bs.min = c(1.6, 1.2, 6, 5),
                     SHAPE_Bs.max = c(1.6, 1.73, 10, 7.0))
SHAPE_Bs <- shapes$SHAPE_Bs
PJOINs <- shapes$PJOINs
HEIGHT_out <- shapes$HEIGHT_out
```

```r
AREA_out <- shapes$AREA_out
rbind(AREA, AREA_out, HEIGHT/100, HEIGHT_out)
```

```
##                  [,1]
## AREA       2.144618
## AREA_out   2.193644
##            1.701800
## HEIGHT_out 1.706274
```

```r
par(mfrow = c(1, 1))
plot_human(MASS = MASS,
           HEIGHT = HEIGHT,
           INSDEPDs = INSDEPDs,
           INSDEPVs = INSDEPVs,
           SHAPE_Bs = SHAPE_Bs)
```

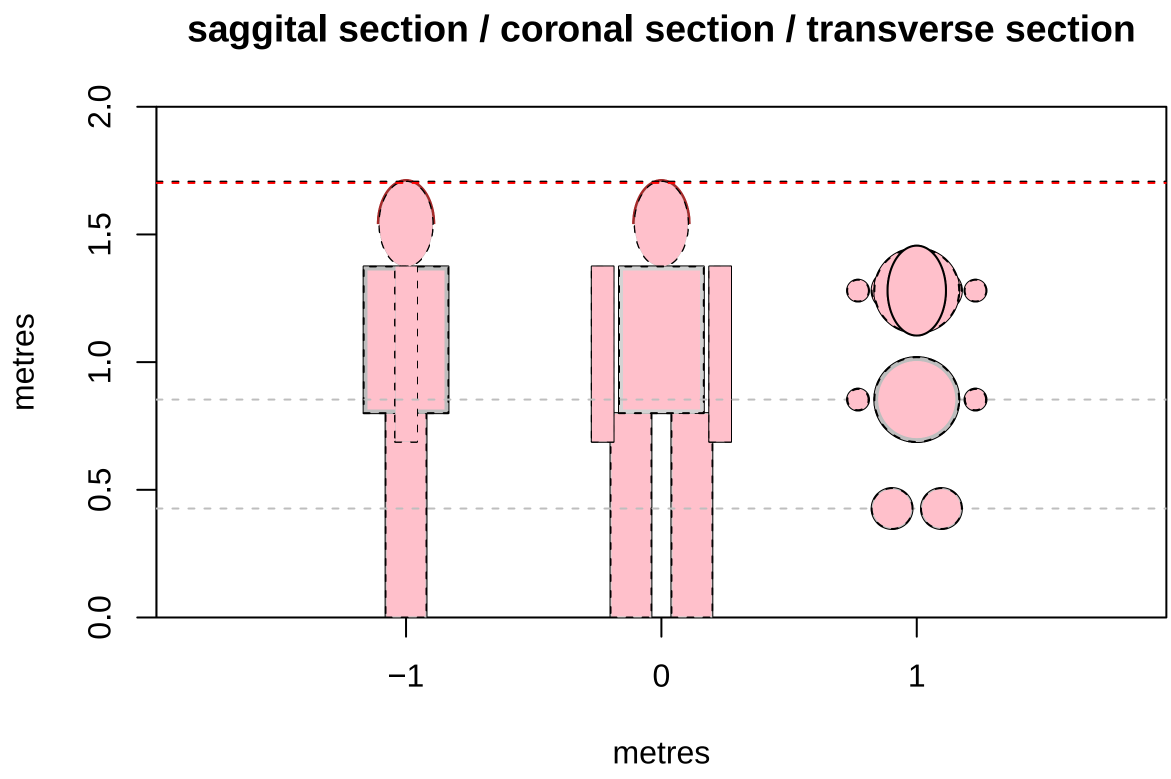


```
## [1] 1.706764
```

Run simulations, first HomoTherm, then

```r
# run HomoTherm simulation
for(i in 1:length(TAs1)){
  HomoTherm.out <- HomoTherm_var(MASS = MASS,
                                 QMETAB_REST = QMETAB_REST,#QGENs1[i],
```

```
                           INSDEPDs = INSDEPDs,
                           INSDEPVs = INSDEPVs,
                           SHAPE_Bs = SHAPE_Bs,
                           PJOINs = PJOINs,
                           PCTBAREVAPs = PCTBAREVAPs,
                           TAs = TAs1[i],
                           TSKYs = TRADs1[i],
                           TGRDs = TRADs1[i],
                           RHs = RHs1[i],
                           VELs = VELs1[i],
                           CONV_ENHANCE = 1.4)
  balance <- HomoTherm.out$balance
  if(i == 1){
    HomoTherm.I <- balance
  }else{
    HomoTherm.I <- rbind(HomoTherm.I, balance)
  }
}

# run MANMO simulation
G_m.G2s <- obs1$M * 4.184 * 1000 / 3600 / AREA # used for MANMO
#clo <- colMeans(get_clo(HomoTherm.out, INSDEPDs = INSDEPDs,
# INSDEPVs = INSDEPVs))
clo <- 0.1
MANMO.output <- run.MANMO(W = rep(1, length(TAs1)) / 100,
                          Ht.H4 = HEIGHT,
                          Wt.W4 = MASS,
                          D3 = c(mean(INSDEPDs[2:4]), rep(1e-10, 3)),
                          Maximum.SR = 1000 / 60 / AREA,
                          G_m.G2s = G_m.G2s,
                          CLO.C4 = clo,
                          CLO.mode = 0,
                          TAs = TAs1,
                          TSKYs = TRADs1,
                          TGNDs = TRADs1,
                          RH.H2s = RHs1 / 100,
                          VELs = VELs1)
MANMO.I <- MANMO.output

HHB <- lapply(1:length(TAs1),
              function(x){run_HHB(exp_time = 6,
                                  AD = AREA,
                                  M = obs1$M[x] * 4184 / 3600,
                                  Tsk_C = obs1$T_S[x], #HomoTherm.I$T_SKIN[x],
                                  Emm_sk = 0.98,
                                  Ar_AD = 0.7,
                                  Icl = clo,
                                  Ta_C = TAs1[x],
                                  humidity = RHs1[x],
                                  Av_ms = VELs1[x],
                                  mrt_C = TRADs1[x],
                                  deltaT = 13 - 36.8, # default TC_REST 36.8
                                  Mass = MASS,
```

```
                                  Smax = 1.5,
                                  Re_cl = 0,
                                  wmax_condition = 1
              )})
HHB.I <- as.data.frame(do.call(rbind, HHB))

# Iso7933
for(i in 1:length(TAs1)){
  Iso7933.out <- calcIso7933_Tcl(accl = 0,
                              posture = 1,
                              Ta = TAs1[i],
                              Pa = WETAIR(db = TAs1[i], rh = RHs1[i])$e / 1000,
                              Tr = TRADs1[i],
                              Va = VELs1[i],
                              Tsk = TAs1[i],
                              Met = QMETAB_REST / AREA,
                              Icl = clo,
                              weight = MASS,
                              height = HEIGHT / 100,
                              Adu = AREA,
                              Tre = 36.8, # default TC_REST 36.8
                              Tcr = 36.8, # default TC_REST 36.8
                              SWp = 0.5)
  if(i == 1){
    Iso7933.out.I <- Iso7933.out
  }else{
    Iso7933.out.I <- rbind(Iso7933.out.I, Iso7933.out)
  }
}

all.I <- cbind(obs1, MANMO.I, HomoTherm.I, HHB.I, Iso7933.out.I)

HomoTherm.I <- cbind(TOs1, HomoTherm.I)
colnames(HomoTherm.I)[1] <- 'TOs'
HomoTherm.I <- HomoTherm.I[order(HomoTherm.I$TOs), ]

MANMO.I <- cbind(TOs1, MANMO.I)
colnames(MANMO.I)[1] <- 'TOs'
MANMO.I <- MANMO.I[order(MANMO.I$TOs), ]

HHB.I <- cbind(TOs1, HHB.I)
colnames(HHB.I)[1] <- 'TOs'
HHB.I <- HHB.I[order(HHB.I$TOs), ]

Iso7933.out.I <- cbind(TOs1, Iso7933.out.I)
colnames(Iso7933.out.I)[1] <- 'TOs'
Iso7933.out.I <- Iso7933.out.I[order(Iso7933.out.I$TOs), ]

head.morph <- HomoTherm.out$head.morph
trunk.morph <- HomoTherm.out$trunk.morph
arm.morph <- HomoTherm.out$arm.morph
leg.morph <- HomoTherm.out$leg.morph
```

```r
# check that HomoTherm default configuration factor values (FSKREFs, FGDREFS)
# captures observed ration of radiation area to total area - should be ~0.75
balance$AREA_RAD / balance$AREA
```

```
## [1] 0.7603852
```

Check results.

```r
par(mfrow = c(2, 3))
par(oma = c(4, 2, 2, 2) + 0.1) # margin spacing
par(mar = c(4, 4, 1, 1) + 0.1) # margin spacing
par(mgp = c(3, 1, 0) ) # margin spacing

with(obs1,
     plot(TOs1, T_S, ylim = c(31, 36.5), xlim = c(27, 42),
          ylab = "Temperature, deg C",
          xlab = expression("Env. Temperature, "*degree*C),
          col = 'red', pch = 16, cex = 1.25, main = 'skin temperature'))
points(MANMO.I$TOs, MANMO.I$Tskin, col = "grey", pch = 16)
points(HomoTherm.I$TOs, HomoTherm.I$T_SKIN, col = "black", pch = 16)
points(Iso7933.out.I$TOs, Iso7933.out.I$Tskeq, col = "darkgreen", pch = 16)
legend(26.5, 36.7, cex = 0.8,
       legend = c('Observed', 'MANMO', 'HomoTherm', 'HHB', 'PHS'),
       col = c('red','grey', 'black', 'orange', 'darkgreen'),
       pch = 16, bty = 'n', ncol = 1)

with(obs1, plot(TOs1, M * 4184 / 3600, ylim = c(70, 140), xlim = c(27, 42),
                ylab = "QMETAB, W",
                xlab = expression("Env. Temperature, "*degree*C),
                col = 'red', pch = 16, cex = 1.25, main = 'metabolic rate'))
points(HomoTherm.I$TOs, HomoTherm.I$QMETAB, col = "black", pch = 16)

with(obs1, plot(TOs1, -E * 4184 / 3600, ylim = c(-20, 300), xlim = c(27, 42),
                ylab = "QEVAP, W",
                xlab = expression("Env. Temperature, "*degree*C),
                col = 'red', pch = 16, cex = 1.25, main = 'evaporation'))
points(MANMO.I$TOs, -MANMO.I$E_m.E, col = "grey", pch = 16)
points(HomoTherm.I$TOs, -(HomoTherm.I$QEVAP_RESP + HomoTherm.I$QEVAP_CUT),
       col = "black", pch = 16)
points(HHB.I$TOs, HHB.I$Ereq, col = "orange", pch = 16)
points(Iso7933.out.I$TOs, (Iso7933.out.I$SWp + Iso7933.out.I$Eres) * AREA,
       col = "darkgreen", pch = 16)

with(obs1, plot(TOs1, R * 4184 / 3600, ylim = c(-100, 300), xlim = c(27, 42),
                ylab = "QRAD, W", xlab =
                  expression("Env. Temperature, "*degree*C), col = 'red',
                pch = 16, cex = 1.25, main = 'radiation'))
points(MANMO.I$TOs, MANMO.I$I_m.I, col = "grey", pch = 16)
points(HomoTherm.I$TOs, HomoTherm.I$QRAD_IN - HomoTherm.I$QRAD_OUT,
       col = "black", pch = 16)

with(obs1, plot(TOs1, C * 4184 / 3600, ylim = c(-250, 50), xlim = c(27, 42),
                ylab = "QCONV, W",
```

```
                xlab = expression("Env. Temperature, "*degree*C),
                col = 'red', pch = 16, cex = 1.25, main = 'convection'))
points(MANMO.I$TOs, MANMO.I$H_m.H, col = "grey", pch = 16)
points(HomoTherm.I$TOs, HomoTherm.I$QCONV_RESP + HomoTherm.I$QCONV,
       col = "black", pch = 16)

with(obs1, plot(TOs1, deltaMASS, ylim = c(0, 8), xlim = c(27, 42),
                ylab = "g/min",
                xlab = expression("Env. Temperature, "*degree*C),
                col = 'red', pch = 16, cex = 1.25, main = 'water loss'))
points(MANMO.I$TOs, MANMO.I$evap.L.h * 1000 / 60, col = "grey", pch = 16)
points(HomoTherm.I$TOs, (HomoTherm.I$EVAP_CUT_L + HomoTherm.I$EVAP_RESP_L)
       * 1000 / 60, col = "black", pch = 16)
points(HHB.I$TOs, HHB.I$Sreq * 1000 / 60, col = "orange", pch = 16)
points(Iso7933.out.I$TOs, Iso7933.out.I$SWtotg / 480, col = "darkgreen",
       pch = 16)
```

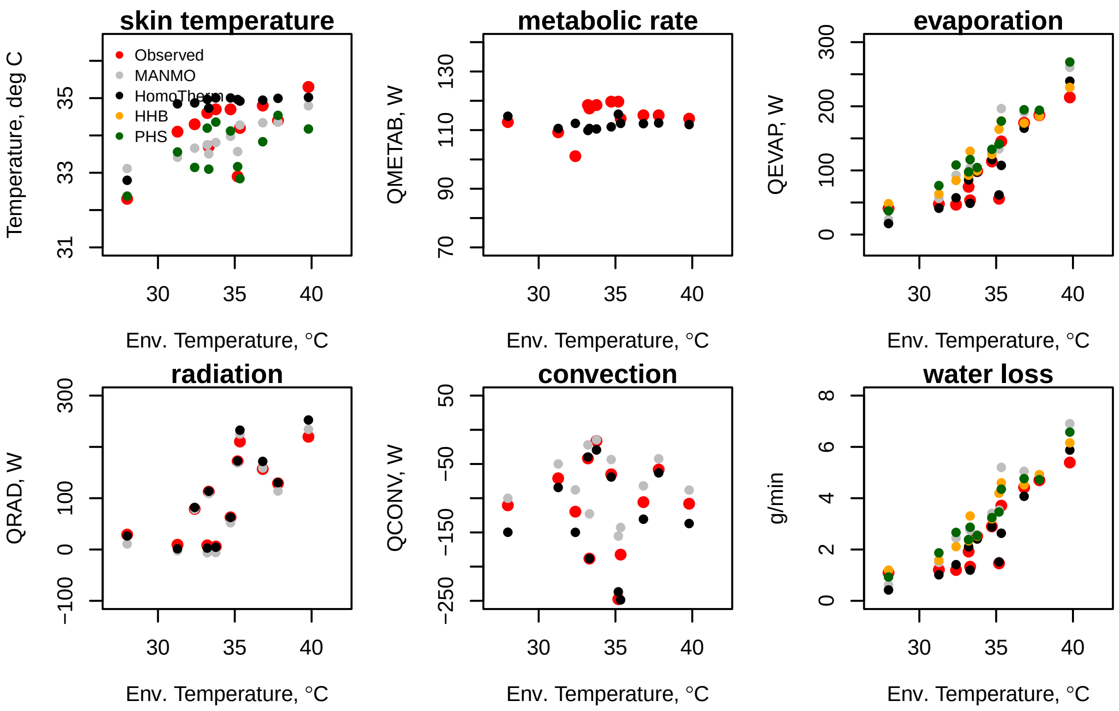

Now plot correlations.

```
par(mfrow = c(2, 3))
par(oma = c(4, 2, 2, 2) + 0.1) # margin spacing
par(mar = c(4, 4, 1, 1) + 0.1) # margin spacing
par(mgp = c(3, 1, 0) ) # margin spacing
sub <- all.I$conditions %in% all.I$conditions#c(3, 4, 5, 6, 8, 9, 10)
with(all.I[sub, ],
     plot(T_S, Tskin, ylim = c(31, 36.5), xlim = c(31, 36.5),
```

```
          ylab = "pred, deg C",
          xlab = "obs, deg C", col = 'grey', pch = 16, cex = 1.25,
          main = 'skin temperature'))
with(all.I[sub, ], points(T_S, T_SKIN, col = "black", pch = 16, cex = 1.25))
with(all.I, points(T_S, Tsk, col = "darkgreen", pch = 16, cex = 1.25))
abline(0, 1)
legend(31, 36.8, cex = 0.8, legend = c('MANMO', 'HomoTherm', 'HHB', 'PHS'),
       col = c('grey', 'black', 'orange', 'darkgreen'), pch = 16,
       bty = 'n', ncol = 1)

with(all.I[sub, ],
     plot(M * 4184 / 3600, M_m.M, ylim = c(70, 140), xlim = c(70, 140),
          ylab = "pred, W", xlab = "obs, W", col = 'white', pch = 16,
          cex = 1.25, main = 'metabolic rate'))
with(all.I[sub, ], points(M * 4184 / 3600, QMETAB, col = "black", pch = 16,
                          cex = 1.25))
abline(0, 1)

with(all.I[sub, ],
     plot(-E * 4184 / 3600, -E_m.E, ylim = c(-20, 300), xlim = c(-20, 300),
          ylab = "pred, W", xlab = "obs, W", col = 'grey', pch = 16, cex = 1.25,
          main = 'evaporation'))
with(all.I[sub, ], points(-E * 4184 / 3600, -(QEVAP_RESP + QEVAP_CUT),
                          col = "black", pch = 16, cex = 1.25))
with(all.I[sub, ], points(-E * 4184 / 3600, (Eres + SWp) * AREA,
                          col = "darkgreen", pch = 16, cex = 1.25))
with(all.I[sub, ], points(-E * 4184 / 3600, Ereq, col = "orange", pch = 16,
                          cex = 1.25))
abline(0, 1)

with(all.I[sub, ],
     plot(R * 4184 / 3600, I_m.I, ylim = c(-100, 300), xlim = c(-100, 300),
          ylab = "pred, W", xlab = "obs, W", col = 'grey', pch = 16,
          cex = 1.25, main = 'radiation'))
with(all.I[sub, ], points(R * 4184 / 3600, QRAD_IN - QRAD_OUT, col = "black",
                          pch = 16, cex = 1.25))
abline(0, 1)

with(all.I[sub, ],
     plot(C * 4184 / 3600, H_m.H, ylim = c(-250, 50), xlim = c(-250, 50),
          ylab = "pred, W", xlab = "obs, W", col = 'grey', pch = 16,
          cex = 1.25, main = 'convection'))
with(all.I[sub, ], points(C * 4184 / 3600, QCONV_RESP + QCONV, col = "black",
                          pch = 16, cex = 1.25))
abline(0, 1)

with(all.I[sub, ],
     plot(deltaMASS, evap.L.h * 1000 / 60, ylim = c(0, 8), xlim = c(0, 8),
          ylab = "pred, g/min", xlab = "obs, g/min", col = 'grey', pch = 16,
          cex = 1.25, main = 'water loss'))
with(all.I[sub, ], points(deltaMASS, (EVAP_CUT_L + EVAP_RESP_L) * 1000 / 60,
                          col = "black", pch = 16, cex = 1.25))
with(all.I[sub, ], points(deltaMASS, SWtotg / 480, col = "darkgreen", pch = 16,
```

```
                          cex = 1.25))
with(all.I[sub, ], points(deltaMASS, Sreq * 1000 / 60, col = "orange", pch = 16,
                          cex = 1.25))
abline(0, 1)
```

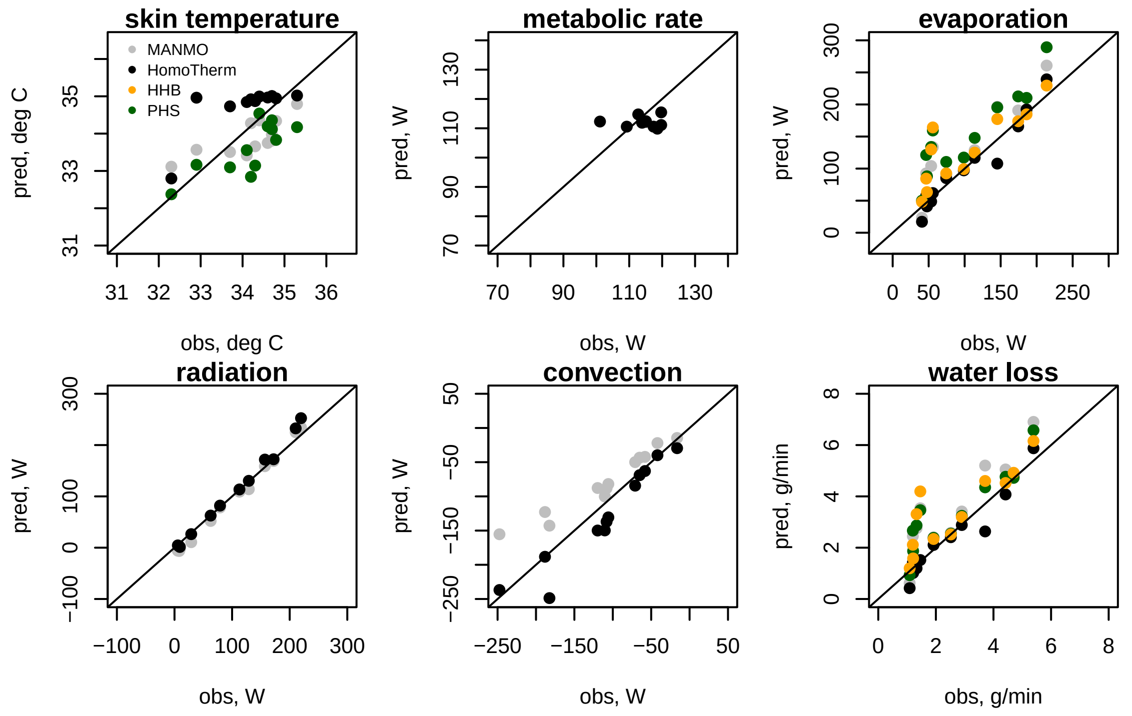

Finally plot the dry heat exchange.

```
par(mfrow = c(1, 2))
par(oma = c(4, 2, 2, 2) + 0.1) # margin spacing
par(mar = c(4, 4, 1, 1) + 0.1) # margin spacing
par(mgp = c(3, 1, 0) ) # margin spacing

with(obs1,
     plot(TOs1, (C + R) * 4184 / 3600, ylim = c(-100, 150), xlim = c(27, 42),
          ylab = "QCONV + QRAD, W",
          xlab = expression("Env. Temperature, "*degree*C),
          col = 'red', pch = 16, cex = 1.25, main = 'dry heat'))
points(MANMO.I$TOs, MANMO.I$H_m.H + MANMO.I$I_m.I, col = "grey", pch = 16)
points(HomoTherm.I$TOs, HomoTherm.I$QCONV_RESP + HomoTherm.I$QCONV +
         HomoTherm.I$QRAD_IN - HomoTherm.I$QRAD_OUT, col = "black", pch = 16)
points(HHB.I$TOs, -HHB.I$Dry_Heat_Loss, col = "orange", pch = 16)

with(all.I,
     plot((C + R) * 4184 / 3600, H_m.H + I_m.I, ylim = c(-100, 150),
          xlim = c(-100, 150), ylab = "pred, W", xlab = "obs, W", col = 'grey',
          pch = 16, cex = 1.25, main = 'dry heat'))
```

```
with(all.I, points((C + R) * 4184 / 3600, QCONV_RESP + QCONV + QRAD_IN -
                     QRAD_OUT, col = "black", pch = 16, cex = 1.25))
with(all.I, points((C + R) * 4184 / 3600, -Dry_Heat_Loss, col = "orange",
                   pch = 16, cex = 1.25))
abline(0, 1)
```

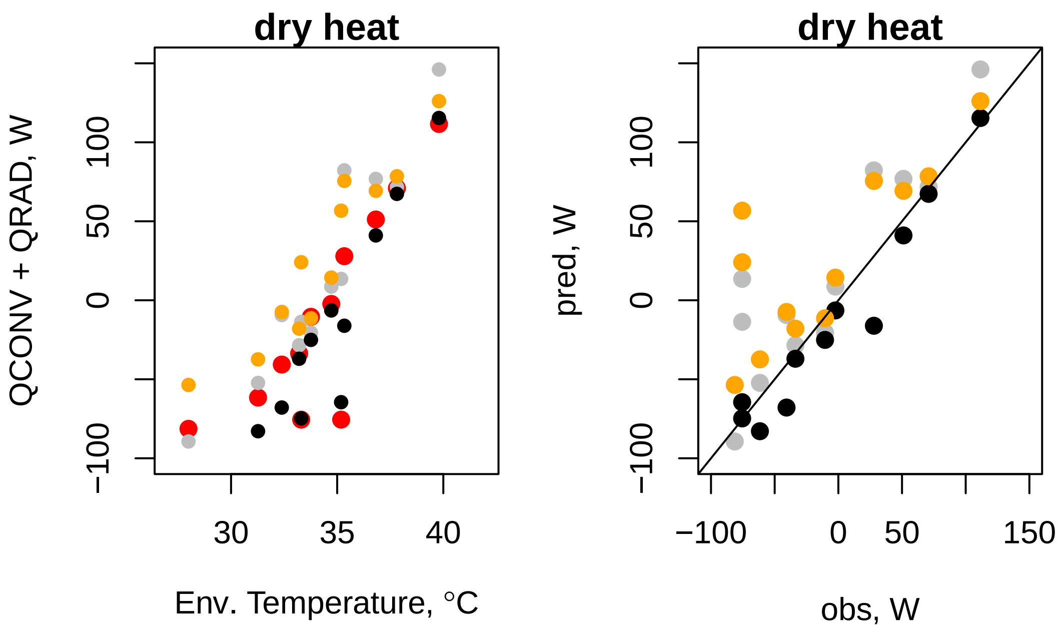

Simulate Person 2

Subject II parameters.

```
# person parameters
MASS <- 47.6 # mass, kg
HEIGHT <- 165 # height, cm
AREA <- 0.00718 * MASS ^ 0.425 * HEIGHT ^ 0.725 # DuBois area, m2
QMETAB_REST <- quantile(obs2$M, 0.1) * 4184 / 3600 # basal metabolic rate, W
SHAPE_Bs <- c(1.4, 1.9, 22, 8)#c(1.6, 1.9, 11, 7.0)
shapes <- GET_SHAPES(MASSs = MASS * MASSFRACs,
                     AREA = AREA,
                     SHAPE_Bs = SHAPE_Bs,
                     SHAPE_Bs.min = c(1.6, 1.2, 6, 5),
                     SHAPE_Bs.max = c(1.4, 2.5, 22, 8))
SHAPE_Bs <- shapes$SHAPE_Bs
PJOINs <- shapes$PJOINs
HEIGHT_out <- shapes$HEIGHT_out
```

```
AREA_out <- shapes$AREA_out
rbind(AREA, AREA_out, HEIGHT/100, HEIGHT_out)
```

```
##                 [,1]
## AREA        1.502392
## AREA_out    1.492342
##             1.650000
## HEIGHT_out 1.642035
```

```
par(mfrow = c(1, 1))
plot_human(MASS = MASS,
           HEIGHT = HEIGHT,
           INSDEPDs = INSDEPDs,
           INSDEPVs = INSDEPVs,
           SHAPE_Bs = SHAPE_Bs)
```

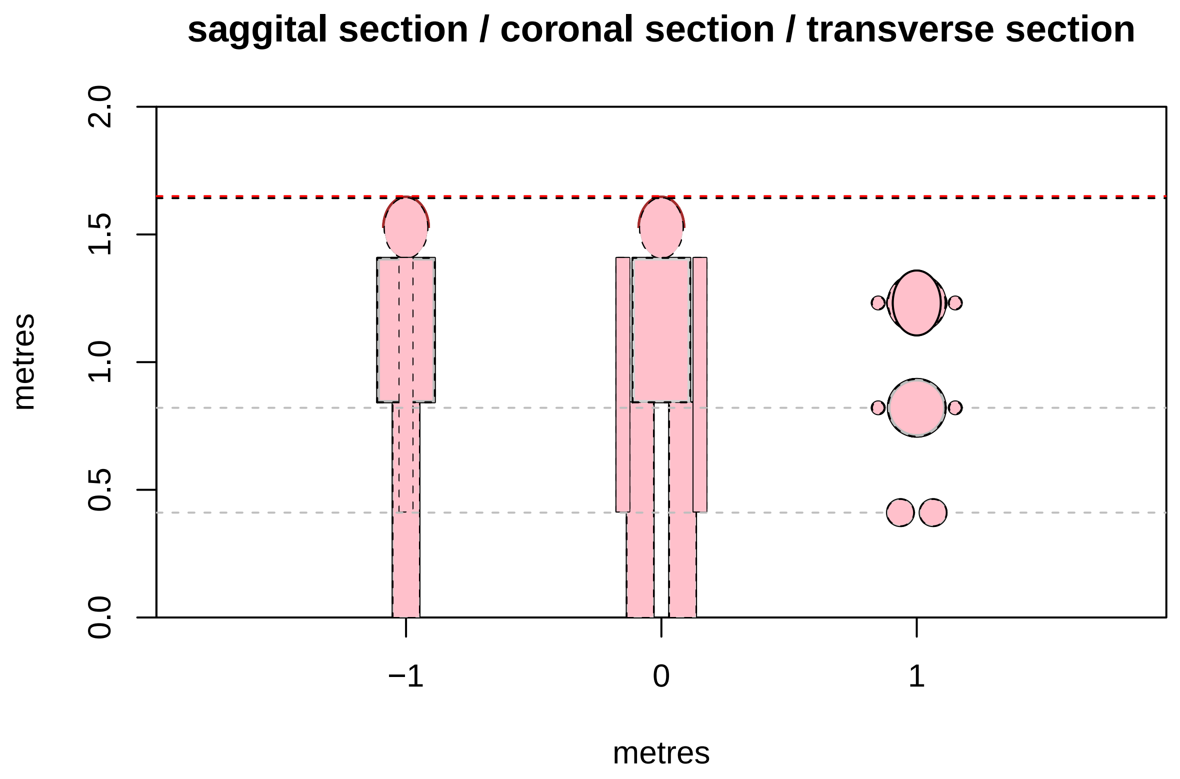


```
## [1] 1.642289
```

Run simulations.

```
# run HomoTherm simulation
for(i in 1:length(TAs2)){
  HomoTherm.out <- HomoTherm_var(MASS = MASS,
                                 QMETAB_REST = QMETAB_REST,#QGENs2[i],
```

```r
                              INSDEPDs = INSDEPDs,
                              INSDEPVs = INSDEPVs,
                              SHAPE_Bs = SHAPE_Bs,
                              PJOINs = PJOINs,
                              PCTBAREVAPs = PCTBAREVAPs,
                              TAs = TAs2[i],
                              TSKYs = TRADs2[i],
                              TGRDs = TRADs2[i],
                              RHs = RHs2[i],
                              VELs = VELs2[i],
                              CONV_ENHANCE = 1.)
    balance <- HomoTherm.out$balance
  if(i == 1){
    HomoTherm.II <- balance
  }else{
    HomoTherm.II <- rbind(HomoTherm.II, balance)
  }
}

# run MANMO simulation
G_m.G2s <- obs2$M * 4.184 * 1000 / 3600 / AREA # used for MANMO
#clo <- colMeans(get_clo(HomoTherm.out, INSDEPDs = INSDEPDs,
# INSDEPVs = INSDEPVs))
clo <- 0.1
MANMO.output <- run.MANMO(W = rep(1, length(TAs2)) / 100,
                          Ht.H4 = HEIGHT,
                          Wt.W4 = MASS,
                          D3 = c(mean(INSDEPDs[2:4]), rep(1e-10, 3)),
                          Maximum.SR = 1000 / 60 / AREA,
                          G_m.G2s = G_m.G2s,
                          CLO.C4 = clo,
                          CLO.mode = 0,
                          TAs = TAs2,
                          TSKYs = TRADs2,
                          TGNDs = TRADs2,
                          RH.H2s = RHs2 / 100,
                          VELs = VELs2)
MANMO.II <- MANMO.output

HHB <- lapply(1:length(TAs2),
              function(x){run_HHB(exp_time = 6,
                                  AD = AREA,
                                  M = obs2$M[x] * 4184 / 3600,
                                  Tsk_C = obs2$T_S[x], #HomoTherm.II$T_SKIN[x],
                                  Emm_sk = 0.98,
                                  Ar_AD = 0.7,
                                  Icl = clo,
                                  Ta_C = TAs2[x],
                                  humidity = RHs2[x],
                                  Av_ms = VELs2[x],
                                  mrt_C = TRADs2[x],
                                  deltaT = 13 - 36.8, # default TC_REST 36.8
                                  Mass = MASS,
```

```
                                    Smax = 1.5,
                                    Re_cl = 0,
                                    wmax_condition = 1
                    )})
HHB.II <- as.data.frame(do.call(rbind, HHB))

# Iso7933
for(i in 1:length(TAs2)){
  Iso7933.out <- calcIso7933_Tcl(accl = 0,
                                  posture = 1,
                                  Ta = TAs2[i],
                                  Pa = WETAIR(db = TAs2[i], rh = RHs2[i])$e / 1000,
                                  Tr = TRADs2[i],
                                  Va = VELs2[i],
                                  Tsk = TAs2[i],
                                  Met = QMETAB_REST / AREA,
                                  Icl = clo,
                                  weight = MASS,
                                  height = HEIGHT / 100,
                                  Adu = AREA,
                                  Tre = 36.8, # default TC_REST 36.8
                                  Tcr = 36.8, # default TC_REST 36.8
                                  SWp = 0.5)
  if(i == 1){
    Iso7933.out.II <- Iso7933.out
  }else{
    Iso7933.out.II <- rbind(Iso7933.out.II, Iso7933.out)
  }
}

all.II <- cbind(obs2, MANMO.II, HomoTherm.II, HHB.II, Iso7933.out.II)

HomoTherm.II <- cbind(TOs2, HomoTherm.II)
colnames(HomoTherm.II)[1] <- 'TOs'
HomoTherm.II <- HomoTherm.II[order(HomoTherm.II$TOs), ]

MANMO.II <- cbind(TOs2, MANMO.II)
colnames(MANMO.II)[1] <- 'TOs'
MANMO.II <- MANMO.II[order(MANMO.II$TOs), ]

HHB.II <- cbind(TOs2, HHB.II)
colnames(HHB.II)[1] <- 'TOs'
HHB.II <- HHB.II[order(HHB.II$TOs), ]

Iso7933.out.II <- cbind(TOs2, Iso7933.out.II)
colnames(Iso7933.out.II)[1] <- 'TOs'
Iso7933.out.II <- Iso7933.out.II[order(Iso7933.out.II$TOs), ]

head.morph <- HomoTherm.out$head.morph
trunk.morph <- HomoTherm.out$trunk.morph
arm.morph <- HomoTherm.out$arm.morph
leg.morph <- HomoTherm.out$leg.morph
```

```r
balance$AREA_RAD / balance$AREA
```

```
## [1] 0.756511
```

Plot the results.

```r
par(mfrow = c(2, 3))
par(oma = c(4, 2, 2, 2) + 0.1) # margin spacing
par(mar = c(4, 4, 1, 1) + 0.1) # margin spacing
par(mgp = c(3, 1, 0) ) # margin spacing

with(obs2,
     plot(TOs2, T_S, ylim = c(31, 36.5), xlim = c(27, 42),
          ylab = expression("Temperature, "*degree*C), xlab = expression("Env. Temperature, "*degree*C)
          col = 'red', pch = 16, cex = 1.25, main = 'skin temperature'))
points(MANMO.II$TOs, MANMO.II$Tskin, col = "grey", pch = 16)
points(HomoTherm.II$TOs, HomoTherm.II$T_SKIN, col = "black", pch = 16)
points(Iso7933.out.II$TOs, Iso7933.out.II$Tskeq, col = "darkgreen", pch = 16)
legend(26.5, 36.7, cex = 0.8,
       legend = c('Observed', 'MANMO', 'HomoTherm', 'HHB', 'PHS'),
       col = c('red','grey', 'black', 'orange', 'darkgreen'),
       pch = 16, bty = 'n', ncol = 1)

with(obs2, plot(TOs2, M * 4184 / 3600, ylim = c(70, 140), xlim = c(27, 42),
          ylab = "QMETAB, W", xlab = expression("Env. Temperature, "*degree*C),
                col = 'red', pch = 16, cex = 1.25, main = 'metabolic rate'))
points(HomoTherm.II$TOs, HomoTherm.II$QMETAB, col = "black", pch = 16)

with(obs2, plot(TOs2, -E * 4184 / 3600, ylim = c(-20, 300), xlim = c(27, 42),
          ylab = "QEVAP, W", xlab = expression("Env. Temperature, "*degree*C),
                col = 'red', pch = 16, cex = 1.25, main = 'evaporation'))
points(MANMO.II$TOs, -MANMO.II$E_m.E, col = "grey", pch = 16)
points(HomoTherm.II$TOs, -(HomoTherm.II$QEVAP_RESP + HomoTherm.II$QEVAP_CUT),
       col = "black", pch = 16)
points(HHB.II$TOs, HHB.II$Ereq, col = "orange", pch = 16)
points(Iso7933.out.II$TOs, (Iso7933.out.II$SWp + Iso7933.out.II$Eres) * AREA,
       col = "darkgreen", pch = 16)

with(obs2, plot(TOs2, R * 4184 / 3600, ylim = c(-100, 300), xlim = c(27, 42),
                ylab = "QRAD, W", xlab =
                  expression("Env. Temperature, "*degree*C), col = 'red',
                pch = 16, cex = 1.25, main = 'radiation'))
points(MANMO.II$TOs, MANMO.II$I_m.I, col = "grey", pch = 16)
points(HomoTherm.II$TOs, HomoTherm.II$QRAD_IN - HomoTherm.II$QRAD_OUT,
       col = "black", pch = 16)

with(obs2, plot(TOs2, C * 4184 / 3600, ylim = c(-250, 50), xlim = c(27, 42),
            ylab = "QCONV, W", xlab = expression("Env. Temperature, "*degree*C),
                col = 'red', pch = 16, cex = 1.25, main = 'convection'))
points(MANMO.II$TOs, MANMO.II$H_m.H, col = "grey", pch = 16)
points(HomoTherm.II$TOs, HomoTherm.II$QCONV_RESP + HomoTherm.II$QCONV,
       col = "black", pch = 16)
```

```
with(obs2, plot(TOs2, deltaMASS, ylim = c(0, 8), xlim = c(27, 42),
              ylab = "g/min", xlab = expression("Env. Temperature, "*degree*C),
                col = 'red', pch = 16, cex = 1.25, main = 'water loss'))
points(MANMO.II$TOs, MANMO.II$evap.L.h * 1000 / 60, col = "grey", pch = 16)
points(HomoTherm.II$TOs, (HomoTherm.II$EVAP_CUT_L + HomoTherm.II$EVAP_RESP_L)
       * 1000 / 60, col = "black", pch = 16)
points(HHB.II$TOs, HHB.II$Sreq * 1000 / 60, col = "orange", pch = 16)
points(Iso7933.out.II$TOs, Iso7933.out.II$SWtotg / 480, col = "darkgreen",
       pch = 16)
```

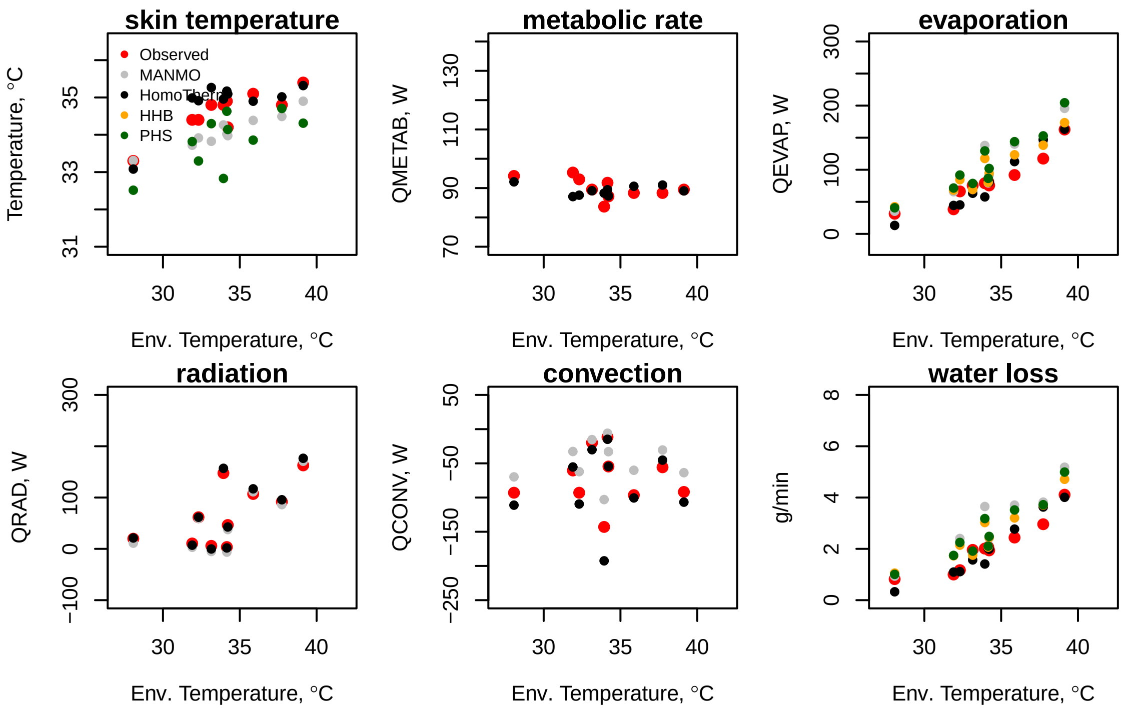

Now plot correlations.

```
par(mfrow = c(2, 3))
par(oma = c(4, 2, 2, 2) + 0.1) # margin spacing
par(mar = c(4, 4, 1, 1) + 0.1) # margin spacing
par(mgp = c(3, 1, 0) ) # margin spacing
sub <- all.II$conditions %in% all.II$conditions#c(3, 4, 5, 6, 8, 9, 10)
with(all.II[sub, ],
     plot(T_S, Tskin, ylim = c(31, 36.5), xlim = c(31, 36.5),
          ylab = "pred, deg C", xlab = "obs, deg C", col = 'grey', pch = 16,
          cex = 1.25, main = 'skin temperature'))
with(all.II[sub, ], points(T_S, T_SKIN, col = "black", pch = 16, cex = 1.25))
with(all.II, points(T_S, Tsk, col = "darkgreen", pch = 16, cex = 1.25))
abline(0, 1)
legend(31, 36.8, cex = 0.8, legend = c('MANMO', 'HomoTherm', 'HHB', 'PHS'),
       col = c('grey', 'black', 'orange', 'darkgreen'), pch = 16,
```

```r
       bty = 'n', ncol = 1)

with(all.II[sub, ],
     plot(M * 4184 / 3600, M_m.M, ylim = c(70, 140), xlim = c(70, 140),
          ylab = "pred, W", xlab = "obs, W", col = 'white', pch = 16,
          cex = 1.25, main = 'metabolic rate'))
with(all.II[sub, ], points(M * 4184 / 3600, QMETAB, col = "black", pch = 16,
                           cex = 1.25))
abline(0, 1)

with(all.II[sub, ],
     plot(-E * 4184 / 3600, -E_m.E, ylim = c(-20, 300), xlim = c(-20, 300),
          ylab = "pred, W", xlab = "obs, W", col = 'grey', pch = 16, cex = 1.25,
          main = 'evaporation'))
with(all.II[sub, ], points(-E * 4184 / 3600, -(QEVAP_RESP + QEVAP_CUT),
                           col = "black", pch = 16, cex = 1.25))
with(all.II[sub, ], points(-E * 4184 / 3600, (Eres + SWp) * AREA,
                           col = "darkgreen",
                   pch = 16, cex = 1.25))
with(all.II[sub, ], points(-E * 4184 / 3600, Ereq, col = "orange", pch = 16,
                          cex = 1.25))
abline(0, 1)

with(all.II[sub, ],
     plot(R * 4184 / 3600, I_m.I, ylim = c(-100, 300), xlim = c(-100, 300),
          ylab = "pred, W", xlab = "obs, W", col = 'grey', pch = 16,
          cex = 1.25, main = 'radiation'))
with(all.II[sub, ], points(R * 4184 / 3600, QRAD_IN - QRAD_OUT, col = "black",
                          pch = 16, cex = 1.25))
abline(0, 1)

with(all.II[sub, ],
     plot(C * 4184 / 3600, H_m.H, ylim = c(-250, 50), xlim = c(-250, 50),
          ylab = "pred, W", xlab = "obs, W", col = 'grey', pch = 16,
          cex = 1.25, main = 'convection'))
with(all.II[sub, ], points(C * 4184 / 3600, QCONV_RESP + QCONV, col = "black",
                          pch = 16, cex = 1.25))
abline(0, 1)

with(all.II[sub, ],
     plot(deltaMASS, evap.L.h * 1000 / 60, ylim = c(0, 8), xlim = c(0, 8),
          ylab = "pred, g/min", xlab = "obs, g/min", col = 'grey', pch = 16,
          cex = 1.25, main = 'water loss'))
with(all.II[sub, ], points(deltaMASS, (EVAP_CUT_L + EVAP_RESP_L) * 1000 / 60,
                          col = "black", pch = 16, cex = 1.25))
with(all.II[sub, ], points(deltaMASS, SWtotg / 480, col = "darkgreen", pch = 16,
                          cex = 1.25))
with(all.II[sub, ], points(deltaMASS, Sreq * 1000 / 60, col = "orange",
                           pch = 16, cex = 1.25))
abline(0, 1)
```

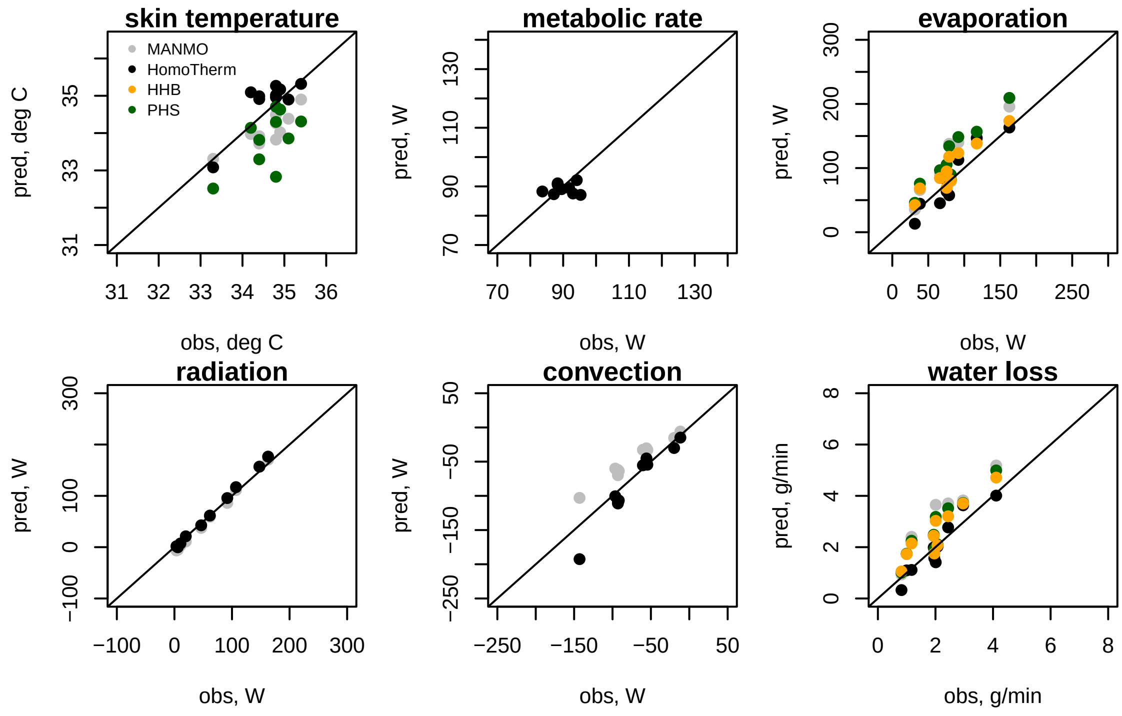

Finally plot the dry heat exchange.

```
par(mfrow = c(1, 2))
par(oma = c(4, 2, 2, 2) + 0.1) # margin spacing
par(mar = c(4, 4, 1, 1) + 0.1) # margin spacing
par(mgp = c(3, 1, 0) ) # margin spacing

with(obs2,
     plot(TOs2, (C + R) * 4184 / 3600, ylim = c(-100, 150), xlim = c(27, 42),
          ylab = "QCONV + QRAD, W",
          xlab = expression("Env. Temperature, "*degree*C),
          col = 'red', pch = 16, cex = 1.25, main = 'dry heat'))
points(MANMO.II$TOs, MANMO.II$H_m.H + MANMO.II$I_m.I, col = "grey", pch = 16)
points(HomoTherm.II$TOs, HomoTherm.II$QCONV_RESP + HomoTherm.II$QCONV +
         HomoTherm.II$QRAD_IN - HomoTherm.II$QRAD_OUT, col = "black", pch = 16)
points(HHB.II$TOs, -HHB.II$Dry_Heat_Loss, col = "orange", pch = 16)

with(all.II,
     plot((C + R) * 4184 / 3600, H_m.H + I_m.I, ylim = c(-100, 150),
          xlim = c(-100, 150), ylab = "pred, W", xlab = "obs, W", col = 'grey',
          pch = 16, cex = 1.25, main = 'dry heat'))
with(all.II, points((C + R) * 4184 / 3600, QCONV_RESP + QCONV + QRAD_IN -
                      QRAD_OUT,
                    col = "black", pch = 16, cex = 1.25))
with(all.II, points((C + R) * 4184 / 3600, -Dry_Heat_Loss, col = "orange",
                    pch = 16, cex = 1.25))
```

```
abline(0, 1)
```

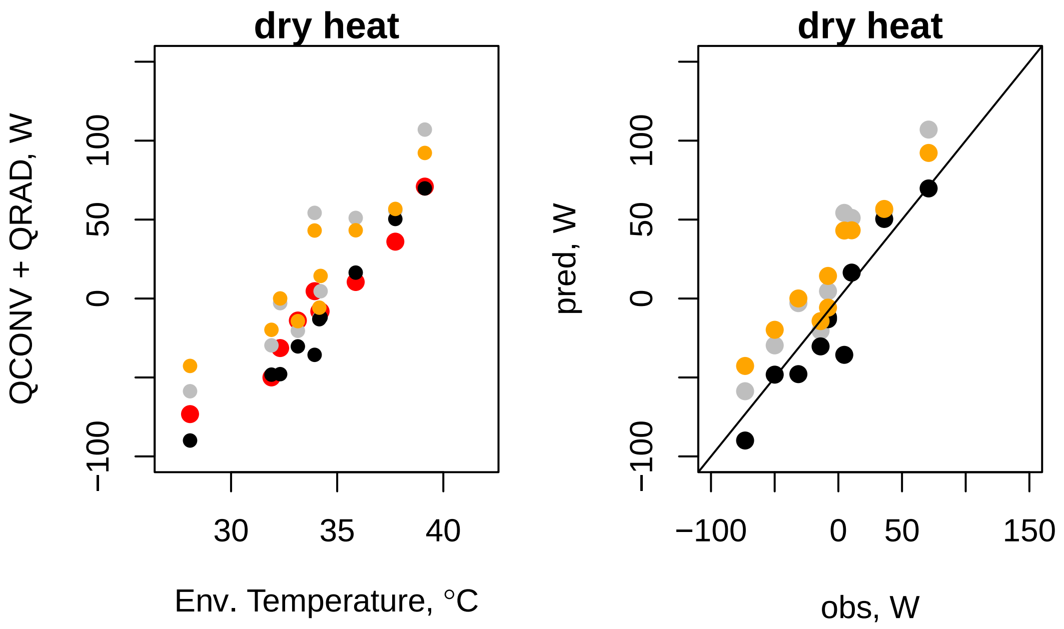

References

Gagge, A. P. 1936. The linearity criterion as applied to partitional calorimetry. American Journal of Physiology-Legacy Content 116:656–668.

Myrup, L. O., and D. L. Morgan. 1972. Numerical model of the urban atmosphere. Volume I The city-surface interface. University of California, Davis.

Vanos, J., G. Guzman-Echavarria, J. W. Baldwin, C. Bongers, K. L. Ebi, and O. Jay. 2023. A physiological approach for assessing human survivability and liveability to heat in a changing climate. Nature Communications 14:7653.

Winslow, C.-E. A., L. P. Herrington, and A. P. Gagge. 1936. The determination of radiation and convection exchanges by partitional calorimetry. American Journal of Physiology-Legacy Content 116:669–684

Winslow, C.-E. A., L. P. Herrington, and A. P. Gagge. 1937. Physiological reactions of the human body to varying environmental temperatures. American Journal of Physiology-Legacy Content 120:1–22.
