# Supplementary material for: HomoTherm: An Open‐Source Approach to Modelling Heat Exchange in Humans and Other Hominins in Diverse Environments
Source: Glob Chang Biol. 2026 Apr 1;32(4):e70830. doi: 10.1111/gcb.70830 (PMC13044332; doi:10.1111/gcb.70830)
Supplement: Supplementary file 9 — Appendix S9: gcb70830‐sup‐0009‐Appendix 9.pdf. [file GCB-32-e70830-s014.pdf]

MANMO_R tests

Michael Kearney

2024-12-20

Contents

Overview . . . 1
Notes on coding R version of MANMO . . . 1
Load required packages and functions . . . 2
Myrup and Morgan 1972 Mode Comparisons . . . 2
References . . . 44

Overview

This document tests the MANMO model by Myrup and Morgan (1972). The BASIC code provided in Myrup and Morgan 1972 Tables 4-11 ('Climatological Mode') and 4-12 ('Standard Measurement Mode') was merged into an R fuction MANMO_R.R. This R function has flags to run in either mode.

The first 'Climatological' mode (`mode = 1`) computes diffuse and direct solar radiation from location, date and time, and uses cloud parameters to compute both the solar radiation and the infra-red environmental radiation. The latter assumes ground temperature is equal to air temperature and uses an algorithm to estimate sky radiation from air temperature and vapour pressure. The second 'Standard Measurement' mode (`mode = 2`) requires user-input of direct and diffuse radiation, as well as ground and sky temperature.

The model can be run with either a fixed clothing temperature (`CLO.mode = 1`) or with the empirical function $T_{clo} = T_a - 21.9 + T_s$. It can also be run with SI units as input (`SImode = 1`) or with the original $Kcalh^{-1}$ units (`SI.mode = 0`). It can also be run using the 'bisection' or 'shooting' method to solve for T_{skin}, where the value is iteratively guessed starting with bracketing values until the heat balance for a given skin wettedness W is found (`iterate = 1`). If this is turned off, it runs for a specified T_{skin} and W value.

The variables have been given symbols that comprise the symbol used in the main text of Myrup and Morgan (1972), followed by a period, followed by the original computer code value in their BASIC program.

Notes on coding R version of MANMO

For the TC mode, Table 4-11 in Myrup and Morgan (1972), there were never terms for the computation of environmental longwave radiation `I_e.E1` and the subtraction of longwave radiation lost `I.I1` to estimate `I_m.I`, despite this term being referred to later in the code. This omission was rectified by inserting Myrup and Morgan's equation 24 for `I_e.E1`. However, the formula in equation 24 of Myrup and Morgan is incorrect because it uses the Brunt formula for mbar vapour pressure (not mmHg) and units of Cal / min / cm, not Kcal / h (see Morgan et al. 1971).

There was also an error in all Myrup and Morgan BASIC versions of MANMO, where the computation of `E_clo.C1` should have been `C1 = X0 * F2 * A4`, as it is in the first part of the code, but when it is

recomputed during the bisection part, it was erroneously entered with the total surface area instead of the clothed surface area, i.e. it was `C1 = X0 * F2 * A2`. This error is explored further in relation to Table 4-11 below.

Another error was that the formulae for the convection through skin and through clothing had their areas reversed in the original code, but also the factor accounting for the resistance of the clothing was incorrectly applied to the skin term so there was no overall error in the calcs. This has been rectified in the computations below but you can see the disparity in the comparison of outputs `H_unc.K1` and `H_clo.S1`, because I kept the Myrup and Morgan results as they presented them.

There was also an error in the formula for `E_r.L1`, where the code had `E_r.L1 = G_m.G2 * 0.0023 * (RH.H2 * E_a.E2 - 44) * A.A2` but equation 30 in Myrup and Morgan was `E_r.L1 = G_m.G2 * 0.0023 * (44 - RH.H2 * E_a.E2) * A.A2`. The latter is correct and gives the same results as what Myrup and Morgan reported (i.e. negative values for `E_r.L1`, as to be expected). For base cutaneous water loss, Myrup and Morgan's equations 31 and 32 had wetness partitioned as: `(0.06 + 0.94 * W)`, i.e. that the base wetness was 6%. I have altered this equation so that the assumption is removed and one just specifies the initial skin wetness to define base cutaneous water loss. In the code below, where tests involved a specific value of W, I changed it to be 0.06 + W * 0.94.

Also, in the SDM mode BASIC code the conduction was not recomputed in the bisection section but there is no good reason for this. It has been included in MANMO_R.R.

Load required packages and functions

```r
source("MANMO_R.R")  # the MANMO function
source("MANMO_R_area_bug.R")  # a function with bugs included from the original BASIC code
library(NicheMapR)  # for WETAIR function
library("knitr")
```

```
## Warning: package 'knitr' was built under R version 4.4.2
```

Myrup and Morgan 1972 Mode Comparisons

These comparisons run simulations with the parameter settings of various tables in section 4 of Myrup and Morgan, comparing the outputs between their values and the R version.

Table 4-15

MANMO in TC (climatological) mode, for a 180 cm tall, 64 kg male aged 22 on 21st July 1971.

```r
# mode of operation

mode <- 1  # 1 climatological, 2 standard innputs
CLO.mode <- 0  # use empirical clothes temp function (0), fixed clothing temp (1) or equal to Tskin (2)

# physiological inputs

age.Y6 <- 22  # age, y
sex.Y7 <- 1  # sex, 1 = male, 2 = female
Ht.H4 <- 180  # height, cm
Wt.W4 <- 64  # weight, kg
```

```r
a_skn.B4 <- 0.35  # albedo of skin, -
e_skin.Y4 <- 0.98  # emissivity of skin, -
K6 <- 0.78  # radiation area coefficient, -
K7 <- 1e-11  # skin contact area coefficient, -
O2 <- 0.1  # orientaion of man, degrees
V3 <- 1.5  # movement of man, m/s
G_m.G2 <- 130  # activity (metabolic costs), Kcal / (h m2)
K8 <- 0.023  # clothes contact area coefficient, -
a_clo.B5 <- 0.3  # albedo of clothes, -
e_clo.Y5 <- 0.95  # emissivity of clothes, -
K3 <- c(0.014, 0.01, 0.072, 1)  # conductivity of clothes, 4 layers, Kcal m / (m2 h C)
D3 <- c(0.001, 0.001, 0.001, 1e-11)  # thickness of clothing, 4 layers, m
CLO.C4 <- 0.6  # clothing units, -

# environmental inputs

month.Z2 <- 7  # month, -
day.Z3 <- 21  # day, -
time.Z4 <- 13  # time, LST
lat.Z5 <- 38.54  # latitude, degrees
lon.Z6 <- 121.78  # longitude, degrees
TZone.Z7 <- 8  # time zone, -
trans.R2 <- 0.6  # transmissivity coefficient, -
a.B3 <- 0.2  # albedo of surface, -
e_sfc.Y3 <- 1  # emissivity of surface, -
k.C2 <- 0  # cloud type code, -
kk.C3 <- 0  # cloud type code, -
n.N2 <- 0  # cloud amount, tenths
T_a.T0 <- 36.2  # air temp, deg C
T_sky.T2 <- T_a.T0  # sky temp, deg C
T_gnd.T7 <- T_a.T0  # ground temp, deg C
T_m.S2 <- T_a.T0  # substrate temp, deg C
RH.H2 <- 0.28  # relative humidity, -
ff.V2 <- 5  # wind speed, m/s
dd.V5 <- 180  # wind direction, degrees
k_x.K2 <- 0.3  # conductivity substrate, Kcal m / (m2 h deg C)
d_x.D2 <- 0.01  # heat thickness substrate, m


out <- MANMO_R(SI.mode = 0, Ht.H4 = Ht.H4, Wt.W4 = Wt.W4, a_skn.B4 = a_skn.B4,
    e_skin.Y4 = e_skin.Y4, K6 = K6, K7 = K7, O2 = O2, V3 = V3,
    G_m.G2 = G_m.G2, K8 = K8, a_clo.B5 = a_clo.B5, e_clo.Y5 = e_clo.Y5,
    K3 = K3, D3 = D3, CLO.C4 = CLO.C4, month.Z2 = month.Z2, day.Z3 = day.Z3,
    time.Z4 = time.Z4, lat.Z5 = lat.Z5, lon.Z6 = lon.Z6, TZone.Z7 = TZone.Z7,
    trans.R2 = trans.R2, a.B3 = a.B3, e_sfc.Y3 = e_sfc.Y3, k.C2 = k.C2,
    kk.C3 = kk.C3, n.N2 = n.N2, T_a.T0 = T_a.T0, T_sky.T2 = T_a.T0,
    T_gnd.T7 = T_a.T0, T_m.S2 = T_m.S2, RH.H2 = RH.H2, ff.V2 = ff.V2,
    dd.V5 = dd.V5, k_x.K2 = k_x.K2, d_x.D2 = d_x.D2, CLO.mode = CLO.mode,
    mode = mode)
out <- t(round(out, 2))
Myrup_Morgan_4_15 <- c(34.38, 0.35, 36.2, 36.2, 36.2, 36.2, 5,
    0.28, NA, NA, 13, 235.9, 241.9, -48.5, -471.6, 28.6, 0.4,
    77.7, 152.9, 106.9, 145.4, 164.2, 562.7, 611.2, -17.1, -253.2,
```

```r
    -201.4, -0.2, 13.3, 15.6, 0, 0.4, 405.1, 683.2, 58.9, 80.1,
    199.8, 35.7, 432.4, 178.8, 1.81, 0.22, 1.28, 0.54, 1.42,
    0, 0.04, 0.7, 44.76, 40.34, 0.78, 0.37, 0.33, 3.5, 15.98,
    37.82, NA, 0.19, 21.41, 1.88, 0.2, NA, NA, NA)
kable(cbind(Myrup_Morgan_4_15, out))
```

	Myrup_Morgan_4_15	
Tskin	34.38	34.66
W	0.35	0.41
T_a.T0	36.20	36.20
T_gnd.T7	36.20	36.20
T_sky.T2	36.20	36.20
T_m.S2	36.20	36.20
ff.V2	5.00	5.00
RH.H2	0.28	0.28
Q_h.Q2	NA	1.08
q_h.Q7	NA	0.13
N	13.00	13.00
M_m.M	235.90	235.92
R_m.R	241.90	223.79
I_m.I	-48.50	-49.47
E_m.E	-471.60	-434.26
H_m.H	28.60	24.23
D_m.D	0.40	0.35
R_munc.R1	77.70	74.41
Q_m.Q1	152.90	136.39
q_v.D1	106.90	106.48
q_g.G1	145.40	144.85
R_mclo.M1	164.20	149.38
I_e.E1	562.70	563.86
I.I1	611.20	613.32
E_r.L1	-17.10	-17.07
E_clo.C1	-253.20	-195.99
E_unc.U1	-201.40	-221.19
H_r.P1	-0.20	-0.22
H_unc.K1	13.30	13.21
H_clo.S1	15.60	11.24
D_unc.A1	0.00	0.00
D_clo.B1	0.40	0.35
R8	405.10	387.72
Q8	683.20	680.80
D8	58.90	58.67
Qq_r.G8	80.10	79.81
R_clo.I8	199.80	191.27
H_clo.H8	35.70	41.89
I_clo.C8	432.40	433.94
I_unc.S8	178.80	179.39
A.A2	1.81	1.81
S_h.A3	0.22	0.20
PA_clo.A4	1.28	1.28
PA_unc.A5	0.54	0.54
A_r.A6	1.42	1.42

	Myrup_Morgan_4_15	
A_kunc.A7	0.00	0.00
A_clo.A8	0.04	0.04
PT_clo.A9	0.70	0.70
E_a.E2	44.76	44.76
E_s.E3	40.34	40.87
W0	0.78	0.78
f_pd.F2	0.37	0.37
f_cl.F3	0.33	0.33
V.V4	3.50	3.50
h_c.H3	15.98	15.98
T_clo.T9	37.82	38.10
T8	NA	36.20
Y0	0.19	0.19
z.K0	21.41	21.41
U0	1.88	1.87
Q0	0.20	0.20
Y	NA	0.56
sweat.g.h	NA	770.72
evap.g.h	NA	802.27

```
plot(out ~ Myrup_Morgan_4_15, ylab = "MANMO_R", xlab = "MANMO_TC")
points(Myrup_Morgan_4_15[26], out[26], col = "red", pch = 16)
abline(0, 1)
```

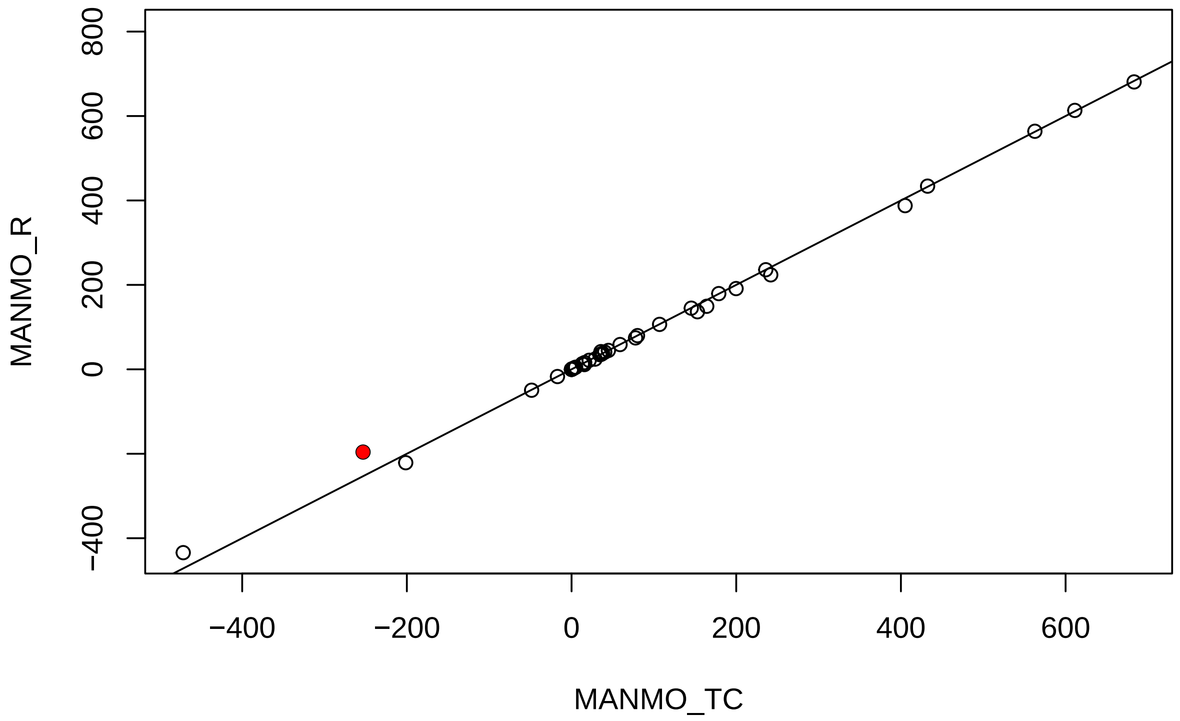

Not everything checks out in the comparison.

A problem with the match was traced back to the bug to do with clothing area mentioned above. In this next run, the model is run without the iteration mode and using the predicted skin temperature and wettedness obtained by Myrup and Morgan's run, using the correct clothing area. It gives a different value for `E_clo.C1` (red dot in scatter plot), and a number of values are out, with the sum of the energy balance `Y` being much greater than zero (105.38).

```
outb <- MANMO_R(SI.mode = 0, Ht.H4 = Ht.H4, Wt.W4 = Wt.W4, a_skn.B4 = a_skn.B4,
    e_skin.Y4 = e_skin.Y4, K6 = K6, K7 = K7, O2 = O2, V3 = V3,
    G_m.G2 = G_m.G2, K8 = K8, a_clo.B5 = a_clo.B5, e_clo.Y5 = e_clo.Y5,
    K3 = K3, D3 = D3, CLO.C4 = CLO.C4, month.Z2 = month.Z2, day.Z3 = day.Z3,
    time.Z4 = time.Z4, lat.Z5 = lat.Z5, lon.Z6 = lon.Z6, TZone.Z7 = TZone.Z7,
    trans.R2 = trans.R2, a.B3 = a.B3, e_sfc.Y3 = e_sfc.Y3, k.C2 = k.C2,
    kk.C3 = kk.C3, n.N2 = n.N2, T_a.T0 = T_a.T0, T_sky.T2 = T_a.T0,
    T_gnd.T7 = T_a.T0, T_m.S2 = T_m.S2, RH.H2 = RH.H2, ff.V2 = ff.V2,
    dd.V5 = dd.V5, k_x.K2 = k_x.K2, d_x.D2 = d_x.D2, CLO.mode = CLO.mode,
    mode = mode, iterate = 0, Tskin1.T = 34.38, W = 0.35 * 0.94 +
        0.06)
outb <- t(round(outb, 2))
kable(cbind(Myrup_Morgan_4_15, out, outb))
```

	Myrup_Morgan_4_15		
Tskin	34.38	34.66	34.38
W	0.35	0.41	0.35
T_a.T0	36.20	36.20	36.20
T_gnd.T7	36.20	36.20	36.20
T_sky.T2	36.20	36.20	36.20
T_m.S2	36.20	36.20	36.20
ff.V2	5.00	5.00	5.00
RH.H2	0.28	0.28	0.28
Q_h.Q2	NA	1.08	1.08
q_h.Q7	NA	0.13	0.13
N	13.00	13.00	0.00
M_m.M	235.90	235.92	235.92
R_m.R	241.90	223.79	270.09
I_m.I	-48.50	-49.47	-47.27
E_m.E	-471.60	-434.26	-357.92
H_m.H	28.60	24.23	28.63
D_m.D	0.40	0.35	0.41
R_munc.R1	77.70	74.41	74.41
Q_m.Q1	152.90	136.39	136.39
q_v.D1	106.90	106.48	106.48
q_g.G1	145.40	144.85	144.85
R_mclo.M1	164.20	149.38	195.68
I_e.E1	562.70	563.86	563.86
I.I1	611.20	613.32	611.13
E_r.L1	-17.10	-17.07	-17.07
E_clo.C1	-253.20	-195.99	-160.13
E_unc.U1	-201.40	-221.19	-180.72
H_r.P1	-0.20	-0.22	-0.22
H_unc.K1	13.30	13.21	15.59
H_clo.S1	15.60	11.24	13.26

	Myrup_Morgan_4_15		
D_unc.A1	0.00	0.00	0.00
D_clo.B1	0.40	0.35	0.41
R8	405.10	387.72	387.72
Q8	683.20	680.80	680.80
D8	58.90	58.67	58.67
Qq_r.G8	80.10	79.81	79.81
R_clo.I8	199.80	191.27	191.27
H_clo.H8	35.70	41.89	-4.42
I_clo.C8	432.40	433.94	432.39
I_unc.S8	178.80	179.39	178.74
A.A2	1.81	1.81	1.81
S_h.A3	0.22	0.20	0.20
PA_clo.A4	1.28	1.28	1.28
PA_unc.A5	0.54	0.54	0.54
A_r.A6	1.42	1.42	1.42
A_kunc.A7	0.00	0.00	0.00
A_clo.A8	0.04	0.04	0.04
PT_clo.A9	0.70	0.70	0.70
E_a.E2	44.76	44.76	44.76
E_s.E3	40.34	40.87	40.34
W0	0.78	0.78	0.78
f_pd.F2	0.37	0.37	0.37
f_cl.F3	0.33	0.33	0.33
V.V4	3.50	3.50	3.50
h_c.H3	15.98	15.98	15.98
T_clo.T9	37.82	38.10	37.82
T8	NA	36.20	36.20
Y0	0.19	0.19	0.19
z.K0	21.41	21.41	21.41
U0	1.88	1.87	1.87
Q0	0.20	0.20	0.20
Y	NA	0.56	129.85
sweat.g.h	NA	770.72	629.70
evap.g.h	NA	802.27	661.24

```r
plot(out ~ Myrup_Morgan_4_15, ylab = "MANMO_R", xlab = "MANMO_TC")
points(outb ~ Myrup_Morgan_4_15, col = "red", pch = 2, ylab = "MANMO_R",
    xlab = "MANMO_TC")
points(Myrup_Morgan_4_15[26], outb[26], col = "red", pch = 16)
abline(0, 1)
```

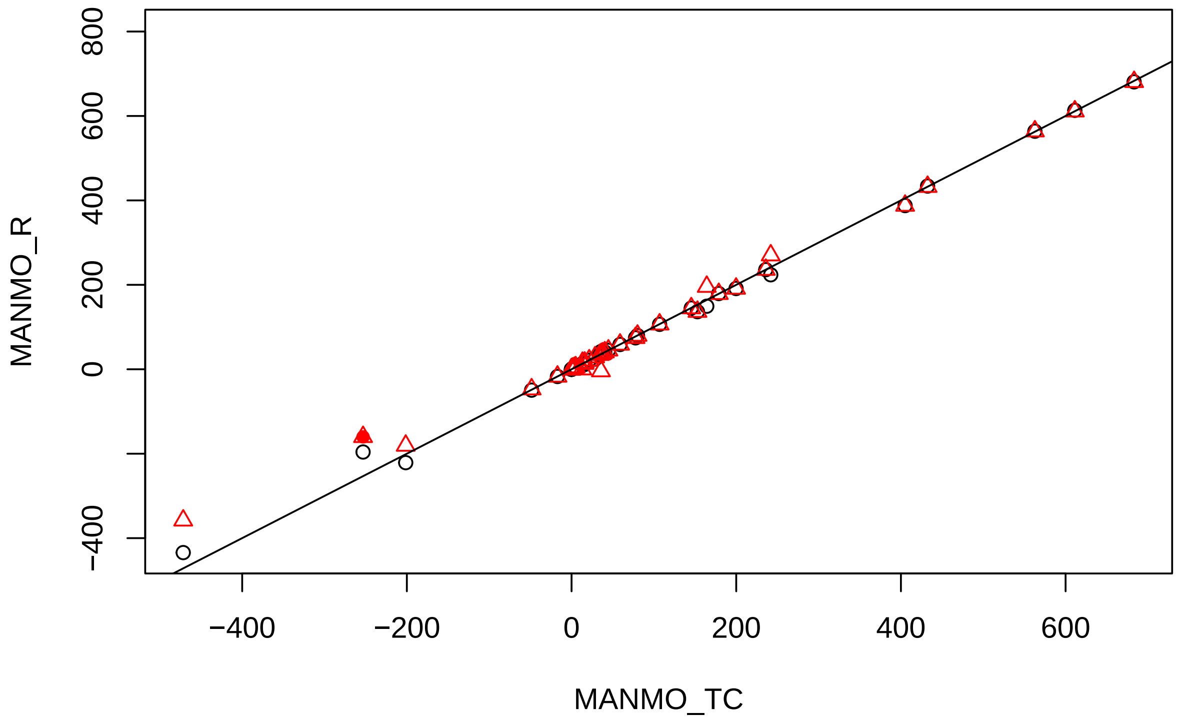

Now trying again but this time using a version of the function with the erroneous version of the formula described above in the code, i.e. using the wrong area for `E_clo.C1`.

```
outc <- MANMO_R_area_bug(SI.mode = 0, Ht.H4 = Ht.H4, Wt.W4 = Wt.W4,
    a_skn.B4 = a_skn.B4, e_skin.Y4 = e_skin.Y4, K6 = K6, K7 = K7,
    O2 = O2, V3 = V3, G_m.G2 = G_m.G2, K8 = K8, a_clo.B5 = a_clo.B5,
    e_clo.Y5 = e_clo.Y5, K3 = K3, D3 = D3, CLO.C4 = CLO.C4, month.Z2 = month.Z2,
    day.Z3 = day.Z3, time.Z4 = time.Z4, lat.Z5 = lat.Z5, lon.Z6 = lon.Z6,
    TZone.Z7 = TZone.Z7, trans.R2 = trans.R2, a.B3 = a.B3, e_sfc.Y3 = e_sfc.Y3,
    k.C2 = k.C2, kk.C3 = kk.C3, n.N2 = n.N2, T_a.T0 = T_a.T0,
    T_sky.T2 = T_a.T0, T_gnd.T7 = T_a.T0, T_m.S2 = T_m.S2, RH.H2 = RH.H2,
    ff.V2 = ff.V2, dd.V5 = dd.V5, k_x.K2 = k_x.K2, d_x.D2 = d_x.D2,
    CLO.mode = CLO.mode, mode = mode, iterate = 0, Tskin1.T = 34.38,
    W = 0.35 * 0.94 + 0.06)
outc <- t(round(outc, 2))
kable(cbind(Myrup_Morgan_4_15, out, outb, outc))
```

	Myrup_Morgan_4_15			
Tskin	34.38	34.66	34.38	34.38
W	0.35	0.41	0.35	0.35
T_a.T0	36.20	36.20	36.20	36.20
T_gnd.T7	36.20	36.20	36.20	36.20
T_sky.T2	36.20	36.20	36.20	36.20
T_m.S2	36.20	36.20	36.20	36.20

	Myrup_Morgan_4_15			
ff.V2	5.00	5.00	5.00	5.00
RH.H2	0.28	0.28	0.28	0.28
Q_h.Q2	NA	1.08	1.08	1.08
q_h.Q7	NA	0.13	0.13	0.13
N	13.00	13.00	0.00	0.00
M_m.M	235.90	235.92	235.92	235.92
R_m.R	241.90	223.79	270.09	240.82
I_m.I	-48.50	-49.47	-47.27	-47.27
E_m.E	-471.60	-434.26	-357.92	-471.48
H_m.H	28.60	24.23	28.63	28.63
D_m.D	0.40	0.35	0.41	0.41
R_munc.R1	77.70	74.41	74.41	77.47
Q_m.Q1	152.90	136.39	136.39	152.31
q_v.D1	106.90	106.48	106.48	106.48
q_g.G1	145.40	144.85	144.85	144.85
R_mclo.M1	164.20	149.38	195.68	163.36
I_e.E1	562.70	563.86	563.86	563.86
I.I1	611.20	613.32	611.13	611.13
E_r.L1	-17.10	-17.07	-17.07	-17.07
E_clo.C1	-253.20	-195.99	-160.13	-253.10
E_unc.U1	-201.40	-221.19	-180.72	-201.30
H_r.P1	-0.20	-0.22	-0.22	-0.22
H_unc.K1	13.30	13.21	15.59	15.59
H_clo.S1	15.60	11.24	13.26	13.26
D_unc.A1	0.00	0.00	0.00	0.00
D_clo.B1	0.40	0.35	0.41	0.41
R8	405.10	387.72	387.72	403.64
Q8	683.20	680.80	680.80	680.80
D8	58.90	58.67	58.67	58.67
Qq_r.G8	80.10	79.81	79.81	79.81
R_clo.I8	199.80	191.27	191.27	199.12
H_clo.H8	35.70	41.89	-4.42	35.76
I_clo.C8	432.40	433.94	432.39	432.39
I_unc.S8	178.80	179.39	178.74	178.74
A.A2	1.81	1.81	1.81	1.81
S_h.A3	0.22	0.20	0.20	0.22
PA_clo.A4	1.28	1.28	1.28	1.28
PA_unc.A5	0.54	0.54	0.54	0.54
A_r.A6	1.42	1.42	1.42	1.42
A_kunc.A7	0.00	0.00	0.00	0.00
A_clo.A8	0.04	0.04	0.04	0.04
PT_clo.A9	0.70	0.70	0.70	0.70
E_a.E2	44.76	44.76	44.76	44.76
E_s.E3	40.34	40.87	40.34	40.34
W0	0.78	0.78	0.78	0.78
f_pd.F2	0.37	0.37	0.37	0.37
f_cl.F3	0.33	0.33	0.33	0.33
V.V4	3.50	3.50	3.50	3.50
h_c.H3	15.98	15.98	15.98	15.98
T_clo.T9	37.82	38.10	37.82	37.82
T8	NA	36.20	36.20	36.20
Y0	0.19	0.19	0.19	0.19

	Myrup_Morgan_4_15			
z.K0	21.41	21.41	21.41	21.41
U0	1.88	1.87	1.87	1.87
Q0	0.20	0.20	0.20	0.20
Y	NA	0.56	129.85	-12.97
sweat.g.h	NA	770.72	629.70	839.49
evap.g.h	NA	802.27	661.24	871.03

```
plot(out ~ Myrup_Morgan_4_15, ylab = "MANMO_R", xlab = "MANMO_TC")
points(outb ~ Myrup_Morgan_4_15, col = "red", pch = 2, ylab = "MANMO_R",
    xlab = "MANMO_TC")
points(outc ~ Myrup_Morgan_4_15, col = "blue", pch = 16, ylab = "MANMO_R",
    xlab = "MANMO_TC")
points(Myrup_Morgan_4_15[26], outc[26], col = "red", pch = 16)
abline(0, 1)
```

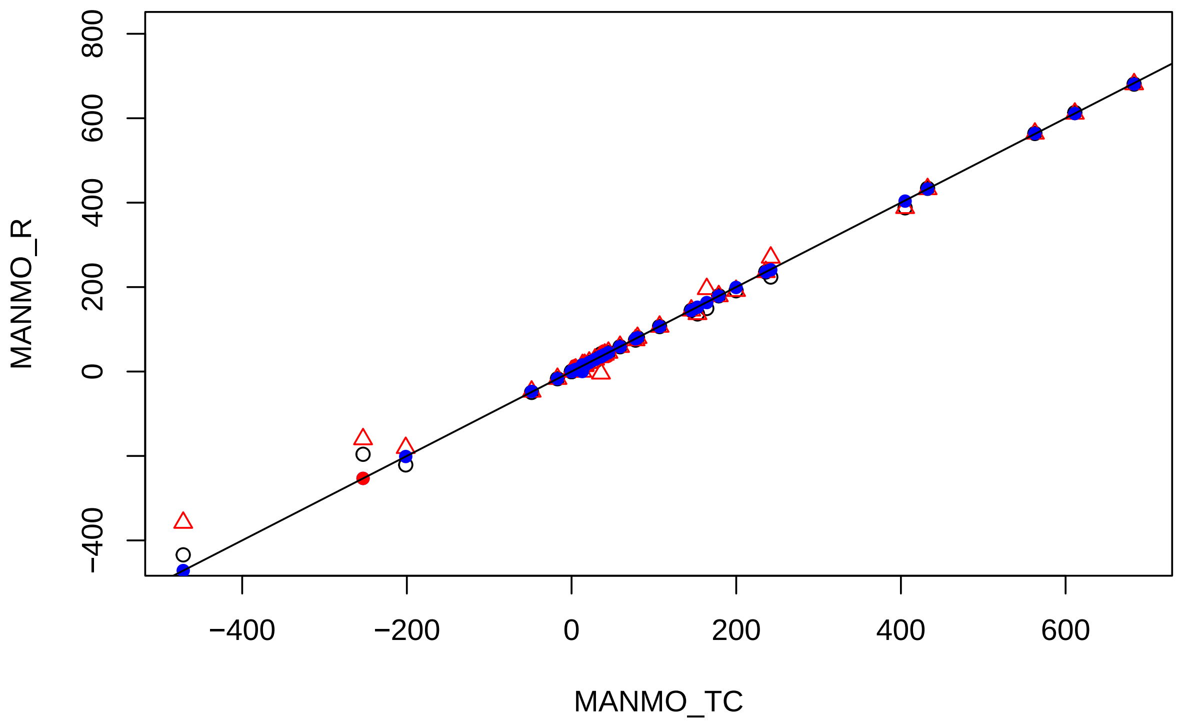

The match is now virtually perfect, the only discrepancy being because of the reversal of the values for convection from clothes and skin. Finally, try with the bisection method turned on.

```
outd <- MANMO_R_area_bug(SI.mode = 0, Ht.H4 = Ht.H4, Wt.W4 = Wt.W4,
    a_skn.B4 = a_skn.B4, e_skin.Y4 = e_skin.Y4, K6 = K6, K7 = K7,
    O2 = O2, V3 = V3, G_m.G2 = G_m.G2, K8 = K8, a_clo.B5 = a_clo.B5,
    e_clo.Y5 = e_clo.Y5, K3 = K3, D3 = D3, CLO.C4 = CLO.C4, month.Z2 = month.Z2,
    day.Z3 = day.Z3, time.Z4 = time.Z4, lat.Z5 = lat.Z5, lon.Z6 = lon.Z6,
```

```
    TZone.Z7 = TZone.Z7, trans.R2 = trans.R2, a.B3 = a.B3, e_sfc.Y3 = e_sfc.Y3,
    k.C2 = k.C2, kk.C3 = kk.C3, n.N2 = n.N2, T_a.T0 = T_a.T0,
    T_sky.T2 = T_a.T0, T_gnd.T7 = T_a.T0, T_m.S2 = T_m.S2, RH.H2 = RH.H2,
    ff.V2 = ff.V2, dd.V5 = dd.V5, k_x.K2 = k_x.K2, d_x.D2 = d_x.D2,
    CLO.mode = CLO.mode, mode = mode, iterate = 1)
outd <- t(round(outd, 2))
kable(cbind(Myrup_Morgan_4_15, out, outb, outc, outd))
```

	Myrup_Morgan_4_15				
Tskin	34.38	34.66	34.38	34.38	34.90
W	0.35	0.41	0.35	0.35	0.47
T_a.T0	36.20	36.20	36.20	36.20	36.20
T_gnd.T7	36.20	36.20	36.20	36.20	36.20
T_sky.T2	36.20	36.20	36.20	36.20	36.20
T_m.S2	36.20	36.20	36.20	36.20	36.20
ff.V2	5.00	5.00	5.00	5.00	5.00
RH.H2	0.28	0.28	0.28	0.28	0.28
Q_h.Q2	NA	1.08	1.08	1.08	1.08
q_h.Q7	NA	0.13	0.13	0.13	0.13
N	13.00	13.00	0.00	0.00	12.00
M_m.M	235.90	235.92	235.92	235.92	235.92
R_m.R	241.90	223.79	270.09	240.82	229.43
I_m.I	-48.50	-49.47	-47.27	-47.27	151.97
E_m.E	-471.60	-434.26	-357.92	-471.48	-636.05
H_m.H	28.60	24.23	28.63	28.63	20.45
D_m.D	0.40	0.35	0.41	0.41	0.29
R_munc.R1	77.70	74.41	74.41	77.47	77.47
Q_m.Q1	152.90	136.39	136.39	152.31	152.31
q_v.D1	106.90	106.48	106.48	106.48	106.48
q_g.G1	145.40	144.85	144.85	144.85	144.85
R_mclo.M1	164.20	149.38	195.68	163.36	151.97
I_e.E1	562.70	563.86	563.86	563.86	563.86
I.I1	611.20	613.32	611.13	611.13	615.21
E_r.L1	-17.10	-17.07	-17.07	-17.07	-17.07
E_clo.C1	-253.20	-195.99	-160.13	-253.10	-344.77
E_unc.U1	-201.40	-221.19	-180.72	-201.30	-274.21
H_r.P1	-0.20	-0.22	-0.22	-0.22	-0.22
H_unc.K1	13.30	13.21	15.59	15.59	11.17
H_clo.S1	15.60	11.24	13.26	13.26	9.50
D_unc.A1	0.00	0.00	0.00	0.00	0.00
D_clo.B1	0.40	0.35	0.41	0.41	0.29
R8	405.10	387.72	387.72	403.64	403.64
Q8	683.20	680.80	680.80	680.80	680.80
D8	58.90	58.67	58.67	58.67	58.67
Qq_r.G8	80.10	79.81	79.81	79.81	79.81
R_clo.I8	199.80	191.27	191.27	199.12	199.12
H_clo.H8	35.70	41.89	-4.42	35.76	47.15
I_clo.C8	432.40	433.94	432.39	432.39	435.27
I_unc.S8	178.80	179.39	178.74	178.74	179.95
A.A2	1.81	1.81	1.81	1.81	1.81
S_h.A3	0.22	0.20	0.20	0.22	0.22
PA_clo.A4	1.28	1.28	1.28	1.28	1.28

	Myrup_Morgan_4_15				
PA_unc.A5	0.54	0.54	0.54	0.54	0.54
A_r.A6	1.42	1.42	1.42	1.42	1.42
A_kunc.A7	0.00	0.00	0.00	0.00	0.00
A_clo.A8	0.04	0.04	0.04	0.04	0.04
PT_clo.A9	0.70	0.70	0.70	0.70	0.70
E_a.E2	44.76	44.76	44.76	44.76	44.76
E_s.E3	40.34	40.87	40.34	40.34	41.32
W0	0.78	0.78	0.78	0.78	0.78
f_pd.F2	0.37	0.37	0.37	0.37	0.37
f_cl.F3	0.33	0.33	0.33	0.33	0.33
V.V4	3.50	3.50	3.50	3.50	3.50
h_c.H3	15.98	15.98	15.98	15.98	15.98
T_clo.T9	37.82	38.10	37.82	37.82	38.34
T8	NA	36.20	36.20	36.20	36.20
Y0	0.19	0.19	0.19	0.19	0.19
z.K0	21.41	21.41	21.41	21.41	21.41
U0	1.88	1.87	1.87	1.87	1.87
Q0	0.20	0.20	0.20	0.20	0.20
Y	NA	0.56	129.85	-12.97	2.01
sweat.g.h	NA	770.72	629.70	839.49	1143.52
evap.g.h	NA	802.27	661.24	871.03	1175.07

```r
plot(out ~ Myrup_Morgan_4_15, ylab = "MANMO_R", xlab = "MANMO_TC")
points(outb ~ Myrup_Morgan_4_15, col = "red", pch = 2, ylab = "MANMO_R",
    xlab = "MANMO_TC")
points(outc ~ Myrup_Morgan_4_15, col = "blue", pch = 16, ylab = "MANMO_R",
    xlab = "MANMO_TC")
points(outd ~ Myrup_Morgan_4_15, col = "purple", pch = 3, ylab = "MANMO_R",
    xlab = "MANMO_TC")
abline(0, 1)
```

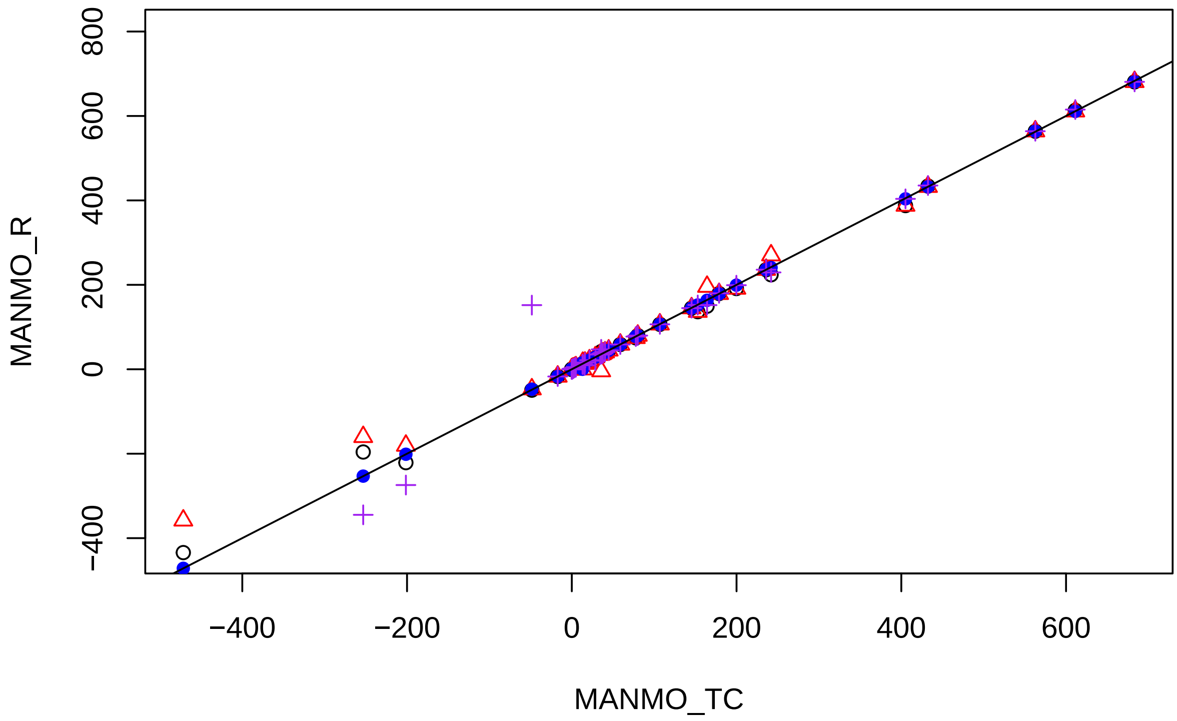

Match not as good - without the bisection calculations, the balance was -13, and in achieving a closer fit, it has altered the terms from what Myrup and Morgan had. Not sure why this is occurring.

Table 4-17

MANMO in SDM (standard input) mode, for a 180 cm tall, 64 kg male aged 22 on 21st July 1971. Try with corrected R function first.

```
# mode of operation

mode <- 2  # 1 climatological, 2 standard innputs
CLO.mode <- 1  # use empirical clothes temp function (0) or fix clothing temp (1)

# physiological inputs

age.Y6 <- 22  # age, y
sex.Y7 <- 1  # sex, 1 = male, 2 = female
Ht.H4 <- 180  # height, cm
Wt.W4 <- 64  # weight, kg

a_skn.B4 <- 0.35  # albedo of skin, -
e_skin.Y4 <- 0.98  # emissivity of skin, -
K6 <- 0.78  # radiation area coefficient, -
K7 <- 1e-11  # skin contact area coefficient, -
O2 <- 0.1  # orientaion of man, degrees
```

```r
V3 <- 1.5  # movement of man, m/s
G_m.G2 <- 130  # activity (metabolic costs), Kcal / (h m2)
K8 <- 0.023  # clothes contact area coefficient, -
a_clo.B5 <- 0.3  # albedo of clothes, -
e_clo.Y5 <- 0.95  # emissivity of clothes, -
K3 <- c(0.014, 0.01, 0.072, 1)  # conductivity of clothes, 4 layers, Kcal m / (m2 h C)
D3 <- c(0.001, 0.001, 0.001, 1e-11)  # thickness of clothing, 4 layers, m
CLO.C4 <- 0.6

# environmental inputs

month.Z2 <- 7  # month, -
day.Z3 <- 21  # day, -
time.Z4 <- 13  # time, LST
lat.Z5 <- 38.54  # latitude, degrees
lon.Z6 <- 121.78  # longitude, degrees
TZone.Z7 <- 8  # time zone, -
trans.R2 <- 0.6  # transmissivity coefficient, -
a.B3 <- 0.2  # albedo of surface, -
e_sfc.Y3 <- 1  # emissivity of surface, -
k.C2 <- 1e-11  # cloud type code, -
kk.C3 <- 1e-11  # cloud type code, -
n.N2 <- 1e-11  # cloud amount, tenths
Q_h.Q2 <- 1.35  # direct (actually direct + diffuse) radiation, ly / min = Cal/cm2/min = 4.1868 J / cm2
q_h.Q7 <- 0.24  # diffuse radiation, ly / min
T_a.T0 <- 36.2  # air temp, deg C
T_sky.T2 <- 24  # sky temp, deg C
T_gnd.T7 <- 57  # ground temp, deg C
T_clo.T9 <- 41.2  # clothing temp, deg C
T_m.S2 <- 57  # substrate temp, deg C
RH.H2 <- 0.28  # relative humidity, -
ff.V2 <- 5  # wind speed, m/s
dd.V5 <- 180  # wind direction, degrees
k_x.K2 <- 0.3  # conductivity substrate, Kcal m / (m2 h deg C)
d_x.D2 <- 0.01  # heat thickness substrate, m

out <- MANMO_R(SI.mode = 0, Ht.H4 = Ht.H4, Wt.W4 = Wt.W4, a_skn.B4 = a_skn.B4,
    e_skin.Y4 = e_skin.Y4, K6 = K6, K7 = K7, O2 = O2, V3 = V3,
    G_m.G2 = G_m.G2, K8 = K8, a_clo.B5 = a_clo.B5, e_clo.Y5 = e_clo.Y5,
    K3 = K3, D3 = D3, CLO.C4 = CLO.C4, month.Z2 = month.Z2, day.Z3 = day.Z3,
    time.Z4 = time.Z4, lat.Z5 = lat.Z5, lon.Z6 = lon.Z6, TZone.Z7 = TZone.Z7,
    trans.R2 = trans.R2, a.B3 = a.B3, e_sfc.Y3 = e_sfc.Y3, k.C2 = k.C2,
    kk.C3 = kk.C3, n.N2 = n.N2, Q_h.Q2 = Q_h.Q2, q_h.Q7 = q_h.Q7,
    T_a.T0 = T_a.T0, T_sky.T2 = T_sky.T2, T_gnd.T7 = T_gnd.T7,
    T_clo.T9 = T_clo.T9, T_m.S2 = T_m.S2, RH.H2 = RH.H2, ff.V2 = ff.V2,
    dd.V5 = dd.V5, k_x.K2 = k_x.K2, d_x.D2 = d_x.D2, CLO.mode = CLO.mode,
    mode = mode)
out <- t(round(out, 2))
Myrup_Morgan_4_17 <- c(34.45, 0.36, 36.2, 57, 24, 57, 5, 0.28,
    1.35, 0.24, 13, 235.9, 187.1, 38, -494.9, 27.4, 5.1, 83.3,
    156.6, 130.7, 147, 103.8, 668.5, 630.5, -17.1, -266.2, -211.7,
    -0.2, 12.7, 14.9, 0, 5.1, 434.1, 699.3, 72, 81, 214, 110.4,
    451.6, 178.9, 1.81, 0.22, 1.28, 0.54, 1.42, 0, 0.04, 0.7,
```

```r
    44.76, 40.48, 0.78, 0.37, 0.33, 3.5, 15.98, 41.2, 40.5, 0.19,
    24.41, NA, NA, NA, NA, NA)
kable(cbind(Myrup_Morgan_4_17, out))
```

	Myrup_Morgan_4_17	
Tskin	34.45	34.81
W	0.36	0.45
T_a.T0	36.20	36.20
T_gnd.T7	57.00	57.00
T_sky.T2	24.00	24.00
T_m.S2	57.00	57.00
ff.V2	5.00	5.00
RH.H2	0.28	0.28
Q_h.Q2	1.35	1.35
q_h.Q7	0.24	0.24
N	13.00	13.00
M_m.M	235.90	235.92
R_m.R	187.10	175.88
I_m.I	38.00	37.15
E_m.E	-494.90	-477.01
H_m.H	27.40	21.83
D_m.D	5.10	5.00
R_munc.R1	83.30	80.18
Q_m.Q1	156.60	140.10
q_v.D1	130.70	130.66
q_g.G1	147.00	147.00
R_mclo.M1	103.80	95.71
I_e.E1	668.50	668.39
I.I1	630.50	631.24
E_r.L1	-17.10	-17.07
E_clo.C1	-266.20	-216.08
E_unc.U1	-211.70	-243.85
H_r.P1	-0.20	-0.22
H_unc.K1	12.70	11.91
H_clo.S1	14.90	10.14
D_unc.A1	0.00	0.00
D_clo.B1	5.10	5.00
R8	434.10	417.76
Q8	699.30	699.30
D8	72.00	72.00
Qq_r.G8	81.00	81.00
R_clo.I8	214.00	206.09
H_clo.H8	110.40	110.38
I_clo.C8	451.60	451.50
I_unc.S8	178.90	179.74
A.A2	1.81	1.81
S_h.A3	0.22	0.20
PA_clo.A4	1.28	1.28
PA_unc.A5	0.54	0.54
A_r.A6	1.42	1.42
A_kunc.A7	0.00	0.00
A_clo.A8	0.04	0.04

	Myrup_Morgan_4_17	
PT_clo.A9	0.70	0.70
E_a.E2	44.76	44.76
E_s.E3	40.48	41.15
W0	0.78	0.78
f_pd.F2	0.37	0.37
f_cl.F3	0.33	0.33
V.V4	3.50	3.50
h_c.H3	15.98	15.98
T_clo.T9	41.20	41.20
T8	40.50	40.50
Y0	0.19	0.19
z.K0	24.41	21.41
U0	NA	NA
Q0	NA	NA
Y	NA	-1.22
sweat.g.h	NA	849.69
evap.g.h	NA	881.24

```
plot(out ~ Myrup_Morgan_4_17, ylab = "MANMO_R", xlab = "MANMO_TC")
abline(0, 1)
```

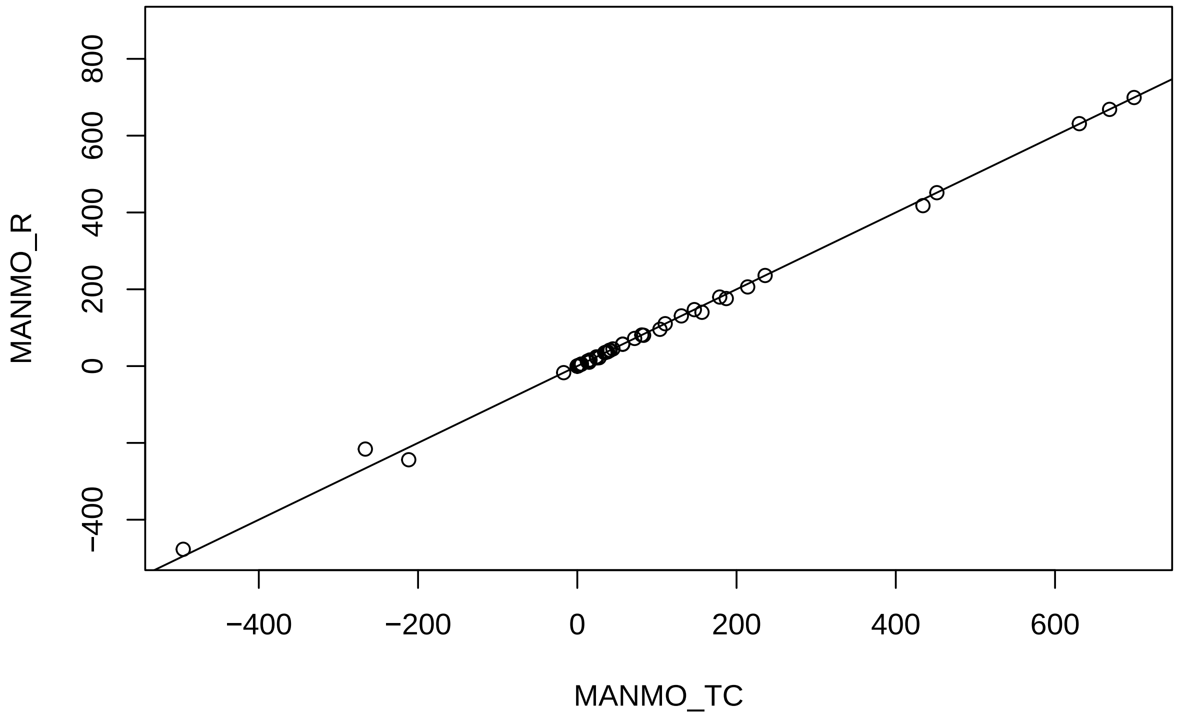

Now try without bisection method, using Myrup and Morgan Tskin and W.

```r
outb <- MANMO_R(SI.mode = 0, Ht.H4 = Ht.H4, Wt.W4 = Wt.W4, a_skn.B4 = a_skn.B4,
    e_skin.Y4 = e_skin.Y4, K6 = K6, K7 = K7, O2 = O2, V3 = V3,
    G_m.G2 = G_m.G2, K8 = K8, a_clo.B5 = a_clo.B5, e_clo.Y5 = e_clo.Y5,
    K3 = K3, D3 = D3, CLO.C4 = CLO.C4, month.Z2 = month.Z2, day.Z3 = day.Z3,
    time.Z4 = time.Z4, lat.Z5 = lat.Z5, lon.Z6 = lon.Z6, TZone.Z7 = TZone.Z7,
    trans.R2 = trans.R2, a.B3 = a.B3, e_sfc.Y3 = e_sfc.Y3, k.C2 = k.C2,
    kk.C3 = kk.C3, n.N2 = n.N2, Q_h.Q2 = Q_h.Q2, q_h.Q7 = q_h.Q7,
    T_a.T0 = T_a.T0, T_sky.T2 = T_sky.T2, T_gnd.T7 = T_gnd.T7,
    T_clo.T9 = T_clo.T9, T_m.S2 = T_m.S2, RH.H2 = RH.H2, ff.V2 = ff.V2,
    dd.V5 = dd.V5, k_x.K2 = k_x.K2, d_x.D2 = d_x.D2, CLO.mode = CLO.mode,
    mode = mode, iterate = 0, Tskin1.T = 34.45, W = 0.36 * 0.94 +
        0.06)
outb <- t(round(outb, 2))
kable(cbind(Myrup_Morgan_4_17, out, outb))
```

	Myrup_Morgan_4_17		
Tskin	34.45	34.81	34.45
W	0.36	0.45	0.36
T_a.T0	36.20	36.20	36.20
T_gnd.T7	57.00	57.00	57.00
T_sky.T2	24.00	24.00	24.00
T_m.S2	57.00	57.00	57.00
ff.V2	5.00	5.00	5.00
RH.H2	0.28	0.28	0.28
Q_h.Q2	1.35	1.35	1.35
q_h.Q7	0.24	0.24	0.24
N	13.00	13.00	0.00
M_m.M	235.90	235.92	235.92
R_m.R	187.10	175.88	175.88
I_m.I	38.00	37.15	37.99
E_m.E	-494.90	-477.01	-376.94
H_m.H	27.40	21.83	27.52
D_m.D	5.10	5.00	5.08
R_munc.R1	83.30	80.18	80.18
Q_m.Q1	156.60	140.10	140.10
q_v.D1	130.70	130.66	130.66
q_g.G1	147.00	147.00	147.00
R_mclo.M1	103.80	95.71	95.71
I_e.E1	668.50	668.39	668.39
I.I1	630.50	631.24	630.40
E_r.L1	-17.10	-17.07	-17.07
E_clo.C1	-266.20	-216.08	-169.06
E_unc.U1	-211.70	-243.85	-190.80
H_r.P1	-0.20	-0.22	-0.22
H_unc.K1	12.70	11.91	14.99
H_clo.S1	14.90	10.14	12.75
D_unc.A1	0.00	0.00	0.00
D_clo.B1	5.10	5.00	5.08
R8	434.10	417.76	417.76
Q8	699.30	699.30	699.30
D8	72.00	72.00	72.00
Qq_r.G8	81.00	81.00	81.00

	Myrup_Morgan_4_17		
R_clo.I8	214.00	206.09	206.09
H_clo.H8	110.40	110.38	110.38
I_clo.C8	451.60	451.50	451.50
I_unc.S8	178.90	179.74	178.91
A.A2	1.81	1.81	1.81
S_h.A3	0.22	0.20	0.20
PA_clo.A4	1.28	1.28	1.28
PA_unc.A5	0.54	0.54	0.54
A_r.A6	1.42	1.42	1.42
A_kunc.A7	0.00	0.00	0.00
A_clo.A8	0.04	0.04	0.04
PT_clo.A9	0.70	0.70	0.70
E_a.E2	44.76	44.76	44.76
E_s.E3	40.48	41.15	40.47
W0	0.78	0.78	0.78
f_pd.F2	0.37	0.37	0.37
f_cl.F3	0.33	0.33	0.33
V.V4	3.50	3.50	3.50
h_c.H3	15.98	15.98	15.98
T_clo.T9	41.20	41.20	41.20
T8	40.50	40.50	40.50
Y0	0.19	0.19	0.19
z.K0	24.41	21.41	21.41
U0	NA	NA	NA
Q0	NA	NA	NA
Y	NA	-1.22	105.45
sweat.g.h	NA	849.69	664.82
evap.g.h	NA	881.24	696.36

```r
plot(out ~ Myrup_Morgan_4_17, ylab = "MANMO_R", xlab = "MANMO_TC")
points(outb ~ Myrup_Morgan_4_17, col = "red", pch = 2, ylab = "MANMO_R",
    xlab = "MANMO_TC")
abline(0, 1)
```

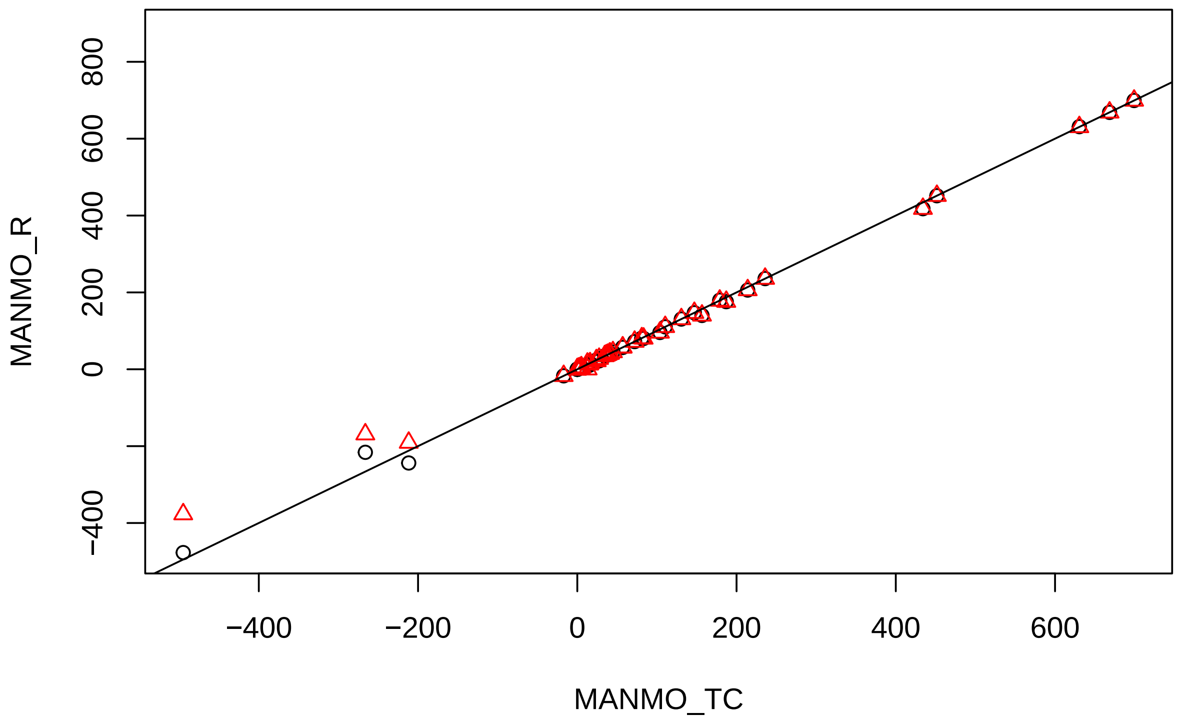

Now trying again but this time putting the erroneous version of the code.

```
outc <- MANMO_R_area_bug(SI.mode = 0, Ht.H4 = Ht.H4, Wt.W4 = Wt.W4,
    a_skn.B4 = a_skn.B4, e_skin.Y4 = e_skin.Y4, K6 = K6, K7 = K7,
    O2 = O2, V3 = V3, G_m.G2 = G_m.G2, K8 = K8, a_clo.B5 = a_clo.B5,
    e_clo.Y5 = e_clo.Y5, K3 = K3, D3 = D3, CLO.C4 = CLO.C4, month.Z2 = month.Z2,
    day.Z3 = day.Z3, time.Z4 = time.Z4, lat.Z5 = lat.Z5, lon.Z6 = lon.Z6,
    TZone.Z7 = TZone.Z7, trans.R2 = trans.R2, a.B3 = a.B3, e_sfc.Y3 = e_sfc.Y3,
    k.C2 = k.C2, kk.C3 = kk.C3, n.N2 = n.N2, Q_h.Q2 = Q_h.Q2,
    q_h.Q7 = q_h.Q7, T_a.T0 = T_a.T0, T_sky.T2 = T_sky.T2, T_gnd.T7 = T_gnd.T7,
    T_clo.T9 = T_clo.T9, T_m.S2 = T_m.S2, RH.H2 = RH.H2, ff.V2 = ff.V2,
    dd.V5 = dd.V5, k_x.K2 = k_x.K2, d_x.D2 = d_x.D2, CLO.mode = CLO.mode,
    mode = mode, iterate = 0, Tskin1.T = 34.45, W = 0.36 * 0.94 +
        0.06)
outc <- t(round(outc, 2))
kable(cbind(Myrup_Morgan_4_17, out, outb, outc))
```

	Myrup_Morgan_4_17			
Tskin	34.45	34.81	34.45	34.45
W	0.36	0.45	0.36	0.36
T_a.T0	36.20	36.20	36.20	36.20
T_gnd.T7	57.00	57.00	57.00	57.00
T_sky.T2	24.00	24.00	24.00	24.00
T_m.S2	57.00	57.00	57.00	57.00

	Myrup_Morgan_4_17			
ff.V2	5.00	5.00	5.00	5.00
RH.H2	0.28	0.28	0.28	0.28
Q_h.Q2	1.35	1.35	1.35	1.35
q_h.Q7	0.24	0.24	0.24	0.24
N	13.00	13.00	0.00	0.00
M_m.M	235.90	235.92	235.92	235.92
R_m.R	187.10	175.88	175.88	187.09
I_m.I	38.00	37.15	37.99	37.99
E_m.E	-494.90	-477.01	-376.94	-493.21
H_m.H	27.40	21.83	27.52	27.52
D_m.D	5.10	5.00	5.08	5.08
R_munc.R1	83.30	80.18	80.18	83.31
Q_m.Q1	156.60	140.10	140.10	156.45
q_v.D1	130.70	130.66	130.66	130.66
q_g.G1	147.00	147.00	147.00	147.00
R_mclo.M1	103.80	95.71	95.71	103.78
I_e.E1	668.50	668.39	668.39	668.39
I.I1	630.50	631.24	630.40	630.40
E_r.L1	-17.10	-17.07	-17.07	-17.07
E_clo.C1	-266.20	-216.08	-169.06	-265.21
E_unc.U1	-211.70	-243.85	-190.80	-210.93
H_r.P1	-0.20	-0.22	-0.22	-0.22
H_unc.K1	12.70	11.91	14.99	14.99
H_clo.S1	14.90	10.14	12.75	12.75
D_unc.A1	0.00	0.00	0.00	0.00
D_clo.B1	5.10	5.00	5.08	5.08
R8	434.10	417.76	417.76	434.11
Q8	699.30	699.30	699.30	699.30
D8	72.00	72.00	72.00	72.00
Qq_r.G8	81.00	81.00	81.00	81.00
R_clo.I8	214.00	206.09	206.09	214.16
H_clo.H8	110.40	110.38	110.38	110.38
I_clo.C8	451.60	451.50	451.50	451.50
I_unc.S8	178.90	179.74	178.91	178.91
A.A2	1.81	1.81	1.81	1.81
S_h.A3	0.22	0.20	0.20	0.22
PA_clo.A4	1.28	1.28	1.28	1.28
PA_unc.A5	0.54	0.54	0.54	0.54
A_r.A6	1.42	1.42	1.42	1.42
A_kunc.A7	0.00	0.00	0.00	0.00
A_clo.A8	0.04	0.04	0.04	0.04
PT_clo.A9	0.70	0.70	0.70	0.70
E_a.E2	44.76	44.76	44.76	44.76
E_s.E3	40.48	41.15	40.47	40.47
W0	0.78	0.78	0.78	0.78
f_pd.F2	0.37	0.37	0.37	0.37
f_cl.F3	0.33	0.33	0.33	0.33
V.V4	3.50	3.50	3.50	3.50
h_c.H3	15.98	15.98	15.98	15.98
T_clo.T9	41.20	41.20	41.20	41.20
T8	40.50	40.50	40.50	40.50
Y0	0.19	0.19	0.19	0.19

Myrup_Morgan_4_17				
z.K0	24.41	21.41	21.41	21.41
U0	NA	NA	NA	NA
Q0	NA	NA	NA	NA
Y	NA	-1.22	105.45	0.38
sweat.g.h	NA	849.69	664.82	879.63
evap.g.h	NA	881.24	696.36	911.18

```
plot(out ~ Myrup_Morgan_4_17, ylab = "MANMO_R", xlab = "MANMO_TC")
points(outb ~ Myrup_Morgan_4_17, col = "red", pch = 2, ylab = "MANMO_R",
    xlab = "MANMO_TC")
points(outc ~ Myrup_Morgan_4_17, col = "blue", pch = 16, ylab = "MANMO_R",
    xlab = "MANMO_TC")
abline(0, 1)
```

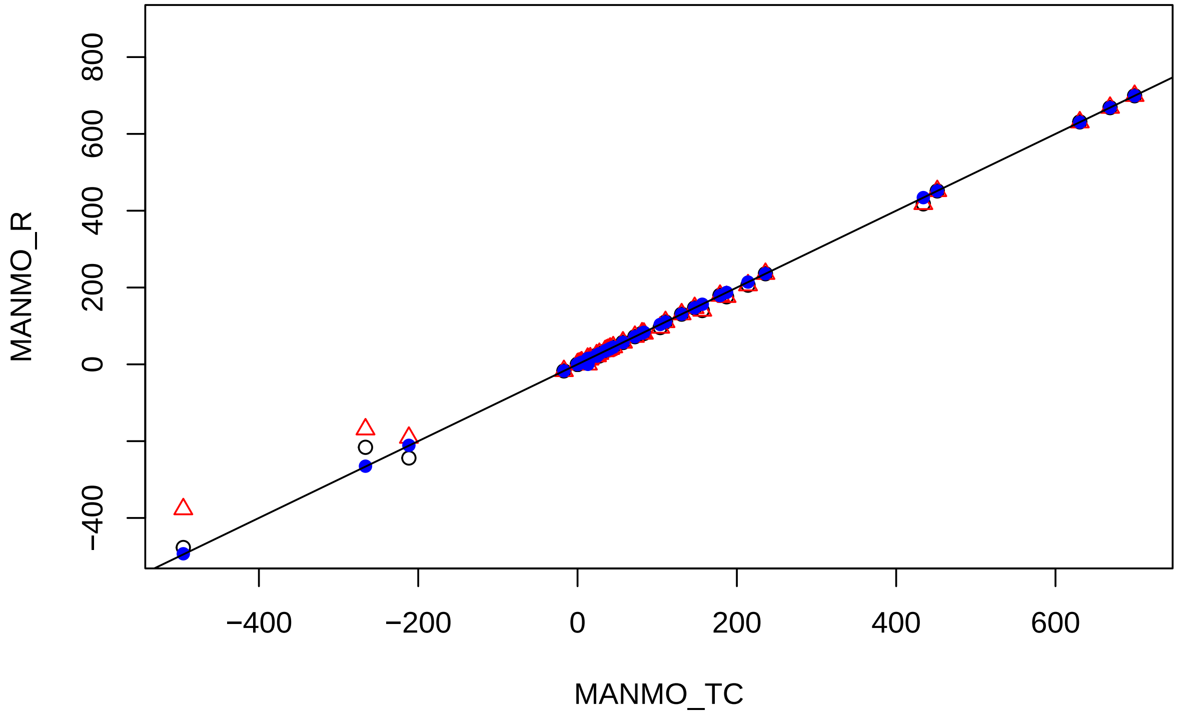

Now the match is virtually perfect. Finally turn on bisection method.

```
outd <- MANMO_R_area_bug(SI.mode = 0, Ht.H4 = Ht.H4, Wt.W4 = Wt.W4,
    a_skn.B4 = a_skn.B4, e_skin.Y4 = e_skin.Y4, K6 = K6, K7 = K7,
    O2 = O2, V3 = V3, G_m.G2 = G_m.G2, K8 = K8, a_clo.B5 = a_clo.B5,
    e_clo.Y5 = e_clo.Y5, K3 = K3, D3 = D3, CLO.C4 = CLO.C4, month.Z2 = month.Z2,
    day.Z3 = day.Z3, time.Z4 = time.Z4, lat.Z5 = lat.Z5, lon.Z6 = lon.Z6,
    TZone.Z7 = TZone.Z7, trans.R2 = trans.R2, a.B3 = a.B3, e_sfc.Y3 = e_sfc.Y3,
    k.C2 = k.C2, kk.C3 = kk.C3, n.N2 = n.N2, Q_h.Q2 = Q_h.Q2,
```

```
    q_h.Q7 = q_h.Q7, T_a.T0 = T_a.T0, T_sky.T2 = T_sky.T2, T_gnd.T7 = T_gnd.T7,
    T_clo.T9 = T_clo.T9, T_m.S2 = T_m.S2, RH.H2 = RH.H2, ff.V2 = ff.V2,
    dd.V5 = dd.V5, k_x.K2 = k_x.K2, d_x.D2 = d_x.D2, CLO.mode = CLO.mode,
    mode = mode, iterate = 1)
outd <- t(round(outd, 2))
kable(cbind(Myrup_Morgan_4_17, out, outb, outc, outd))
```

	Myrup_Morgan_4_17				
Tskin	34.45	34.81	34.45	34.45	34.46
W	0.36	0.45	0.36	0.36	0.36
T_a.T0	36.20	36.20	36.20	36.20	36.20
T_gnd.T7	57.00	57.00	57.00	57.00	57.00
T_sky.T2	24.00	24.00	24.00	24.00	24.00
T_m.S2	57.00	57.00	57.00	57.00	57.00
ff.V2	5.00	5.00	5.00	5.00	5.00
RH.H2	0.28	0.28	0.28	0.28	0.28
Q_h.Q2	1.35	1.35	1.35	1.35	1.35
q_h.Q7	0.24	0.24	0.24	0.24	0.24
N	13.00	13.00	0.00	0.00	12.00
M_m.M	235.90	235.92	235.92	235.92	235.92
R_m.R	187.10	175.88	175.88	187.09	187.09
I_m.I	38.00	37.15	37.99	37.99	37.97
E_m.E	-494.90	-477.01	-376.94	-493.21	-494.95
H_m.H	27.40	21.83	27.52	27.52	27.43
D_m.D	5.10	5.00	5.08	5.08	7.88
R_munc.R1	83.30	80.18	80.18	83.31	83.31
Q_m.Q1	156.60	140.10	140.10	156.45	156.45
q_v.D1	130.70	130.66	130.66	130.66	130.66
q_g.G1	147.00	147.00	147.00	147.00	147.00
R_mclo.M1	103.80	95.71	95.71	103.78	103.78
I_e.E1	668.50	668.39	668.39	668.39	668.39
I.I1	630.50	631.24	630.40	630.40	630.42
E_r.L1	-17.10	-17.07	-17.07	-17.07	-17.07
E_clo.C1	-266.20	-216.08	-169.06	-265.21	-266.17
E_unc.U1	-211.70	-243.85	-190.80	-210.93	-211.70
H_r.P1	-0.20	-0.22	-0.22	-0.22	-0.22
H_unc.K1	12.70	11.91	14.99	14.99	14.94
H_clo.S1	14.90	10.14	12.75	12.75	12.71
D_unc.A1	0.00	0.00	0.00	0.00	0.00
D_clo.B1	5.10	5.00	5.08	5.08	7.88
R8	434.10	417.76	417.76	434.11	434.11
Q8	699.30	699.30	699.30	699.30	699.30
D8	72.00	72.00	72.00	72.00	72.00
Qq_r.G8	81.00	81.00	81.00	81.00	81.00
R_clo.I8	214.00	206.09	206.09	214.16	214.16
H_clo.H8	110.40	110.38	110.38	110.38	110.38
I_clo.C8	451.60	451.50	451.50	451.50	451.50
I_unc.S8	178.90	179.74	178.91	178.91	178.92
A.A2	1.81	1.81	1.81	1.81	1.81
S_h.A3	0.22	0.20	0.20	0.22	0.22
PA_clo.A4	1.28	1.28	1.28	1.28	1.28
PA_unc.A5	0.54	0.54	0.54	0.54	0.54

	Myrup_Morgan_4_17				
A_r.A6	1.42	1.42	1.42	1.42	1.42
A_kunc.A7	0.00	0.00	0.00	0.00	0.00
A_clo.A8	0.04	0.04	0.04	0.04	0.04
PT_clo.A9	0.70	0.70	0.70	0.70	0.70
E_a.E2	44.76	44.76	44.76	44.76	44.76
E_s.E3	40.48	41.15	40.47	40.47	40.48
W0	0.78	0.78	0.78	0.78	0.78
f_pd.F2	0.37	0.37	0.37	0.37	0.37
f_cl.F3	0.33	0.33	0.33	0.33	0.33
V.V4	3.50	3.50	3.50	3.50	3.50
h_c.H3	15.98	15.98	15.98	15.98	15.98
T_clo.T9	41.20	41.20	41.20	41.20	41.20
T8	40.50	40.50	40.50	40.50	40.50
Y0	0.19	0.19	0.19	0.19	0.19
z.K0	24.41	21.41	21.41	21.41	21.41
U0	NA	NA	NA	NA	NA
Q0	NA	NA	NA	NA	NA
Y	NA	-1.22	105.45	0.38	1.35
sweat.g.h	NA	849.69	664.82	879.63	882.84
evap.g.h	NA	881.24	696.36	911.18	914.38

```r
plot(out ~ Myrup_Morgan_4_17, ylab = "MANMO_R", xlab = "MANMO_TC")
points(outb ~ Myrup_Morgan_4_17, col = "red", pch = 2, ylab = "MANMO_R",
    xlab = "MANMO_TC")
points(outc ~ Myrup_Morgan_4_17, col = "blue", pch = 16, ylab = "MANMO_R",
    xlab = "MANMO_TC")
points(outd ~ Myrup_Morgan_4_17, col = "purple", pch = 3, ylab = "MANMO_R",
    xlab = "MANMO_TC")
abline(0, 1)
```

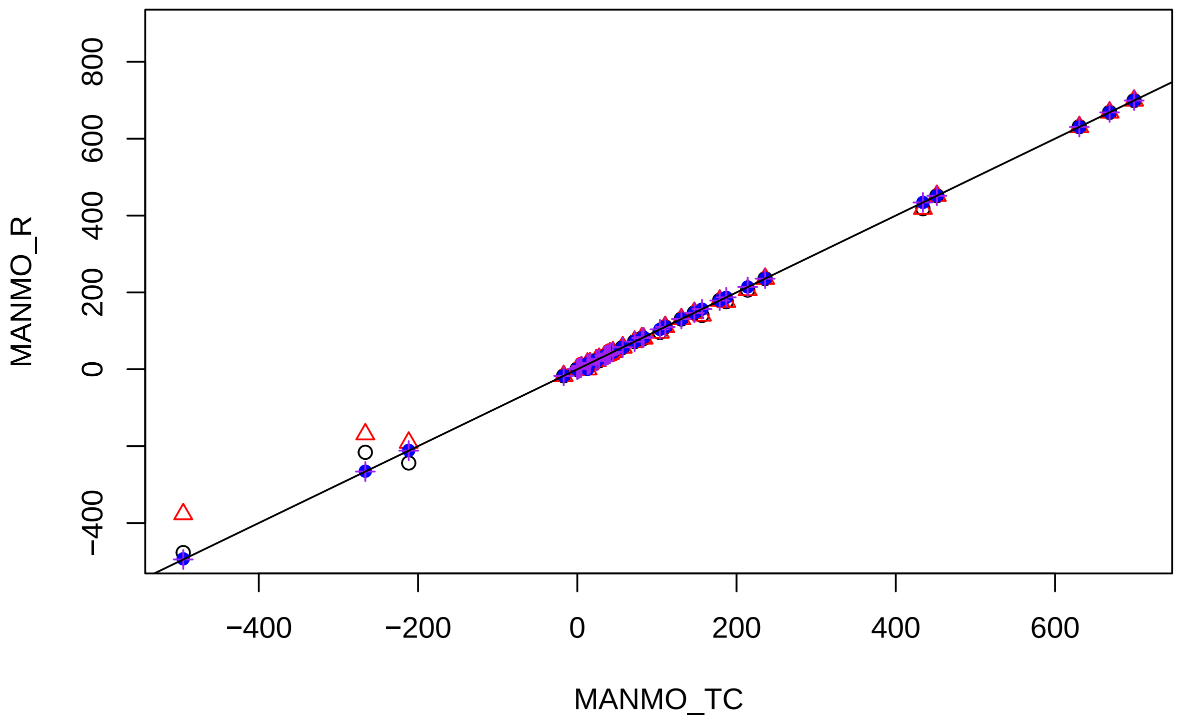

Same results. However, to achieve this, a section of code had to be turned off that was missing from the original BASIC version of MANMO-SDM, but which was present in MANMO-TC. This is incorrect, and has been rectified in the MANMO_R.R version.

Table 4-20

MANMO in TC (climatological input) mode, for a 180 cm tall, 64 kg male aged 22 on 4th August 1971.

```
# mode of operation

mode <- 1  # 1 climatological, 2 standard innputs
CLO.mode <- 0  # use empirical clothes temp function (0) or fix clothing temp (1)

# physiological inputs

age.Y6 <- 22  # age, y
sex.Y7 <- 1  # sex, 1 = male, 2 = female
Ht.H4 <- 180  # height, cm
Wt.W4 <- 64  # weight, kg

a_skn.B4 <- 0.35  # albedo of skin, -
e_skin.Y4 <- 0.98  # emissivity of skin, -
K6 <- 0.78  # radiation area coefficient, -
K7 <- 1e-11  # skin contact area coefficient, -
O2 <- 0.1  # orientaion of man, degrees
```

```r
V3 <- 1.5  # movement of man, m/s
G_m.G2 <- 130  # activity (metabolic costs), Kcal / (h m2)
K8 <- 0.023  # clothes contact area coefficient, -
a_clo.B5 <- 0.3  # albedo of clothes, -
e_clo.Y5 <- 0.95  # emissivity of clothes, -
K3 <- c(0.014, 0.01, 0.072, 1)  # conductivity of clothes, 4 layers, Kcal m / (m2 h C)
D3 <- c(0.001, 0.001, 0.001, 1e-11)  # thickness of clothing, 4 layers, m
CLO.C4 <- 0.6

# environmental inputs

month.Z2 <- 8  # month, -
day.Z3 <- 4  # day, -
time.Z4 <- 10.25  # time, LST
lat.Z5 <- 38.54  # latitude, degrees
lon.Z6 <- 121.78  # longitude, degrees
TZone.Z7 <- 8  # time zone, -
trans.R2 <- 0.6  # transmissivity coefficient, -
a.B3 <- 0.22  # albedo of surface, -
e_sfc.Y3 <- 1  # emissivity of surface, -
k.C2 <- 0  # cloud type code, -
kk.C3 <- 0  # cloud type code, -
n.N2 <- 0  # cloud amount, tenths
T_a.T0 <- 22.3  # air temp, deg C
T_sky.T2 <- T_a.T0  # sky temp, deg C
T_gnd.T7 <- T_a.T0  # ground temp, deg C
T_m.S2 <- T_a.T0  # substrate temp, deg C
RH.H2 <- 0.73  # relative humidity, -
ff.V2 <- 1  # wind speed, m/s
dd.V5 <- 250  # wind direction, degrees
k_x.K2 <- 0.5  # conductivity substrate, Kcal m / (m2 h deg C)
d_x.D2 <- 0.01  # heat thickness substrate, m


out <- MANMO_R(SI.mode = 0, Ht.H4 = Ht.H4, Wt.W4 = Wt.W4, a_skn.B4 = a_skn.B4,
    e_skin.Y4 = e_skin.Y4, K6 = K6, K7 = K7, O2 = O2, V3 = V3,
    G_m.G2 = G_m.G2, K8 = K8, a_clo.B5 = a_clo.B5, e_clo.Y5 = e_clo.Y5,
    K3 = K3, D3 = D3, CLO.C4 = CLO.C4, month.Z2 = month.Z2, day.Z3 = day.Z3,
    time.Z4 = time.Z4, lat.Z5 = lat.Z5, lon.Z6 = lon.Z6, TZone.Z7 = TZone.Z7,
    trans.R2 = trans.R2, a.B3 = a.B3, e_sfc.Y3 = e_sfc.Y3, k.C2 = k.C2,
    kk.C3 = kk.C3, n.N2 = n.N2, T_a.T0 = T_a.T0, T_sky.T2 = T_a.T0,
    T_gnd.T7 = T_a.T0, T_m.S2 = T_m.S2, RH.H2 = RH.H2, ff.V2 = ff.V2,
    dd.V5 = dd.V5, k_x.K2 = k_x.K2, d_x.D2 = d_x.D2, CLO.mode = CLO.mode,
    mode = mode)
out <- t(round(out, 2))
Myrup_Morgan_4_20 <- c(34.38, 0.35, 22.3, 22.3, 22.3, 22.3, 1,
    0.73, NA, NA, 13, 235.9, 256.1, -88.7, -300.1, -122.3, -2.7,
    90.5, 230.9, 101.7, 138.9, 165.6, 470.8, 559.6, -15.9, -187,
    -97.2, -4.1, -63.9, -54.3, 0, -2.7, 471.6, 583.8, 56.1, 76.5,
    232.6, 67, 380.8, 178.8, 1.81, 0.4, 1.28, 0.54, 1.42, 0,
    0.04, 0.7, 20.22, 40.34, 0.79, 0.57, 0.46, 1.49, 8.39, 28.08,
    NA, 0.19, 32.58, 1.7, 0.19, NA, NA, NA)
kable(cbind(out, Myrup_Morgan_4_20))
```

		Myrup_Morgan_4_20
Tskin	34.35	34.38
W	0.34	0.35
T_a.T0	22.30	22.30
T_gnd.T7	22.30	22.30
T_sky.T2	22.30	22.30
T_m.S2	22.30	22.30
ff.V2	1.00	1.00
RH.H2	0.73	0.73
Q_h.Q2	1.08	NA
q_h.Q7	0.13	NA
N	13.00	13.00
M_m.M	235.92	235.90
R_m.R	205.99	256.10
I_m.I	-100.79	-88.70
E_m.E	-217.29	-300.10
H_m.H	-122.03	-122.30
D_m.D	-2.72	-2.70
R_munc.R1	76.40	90.50
Q_m.Q1	158.49	230.90
q_v.D1	101.28	101.70
q_g.G1	138.32	138.90
R_mclo.M1	129.59	165.60
I_e.E1	458.53	470.80
I.I1	559.32	559.60
E_r.L1	-15.87	-15.90
E_clo.C1	-115.91	-187.00
E_unc.U1	-85.51	-97.20
H_r.P1	-4.07	-4.10
H_unc.K1	-54.19	-63.90
H_clo.S1	-63.77	-54.30
D_unc.A1	0.00	0.00
D_clo.B1	-2.72	-2.70
R8	398.09	471.60
Q8	581.27	583.80
D8	55.81	56.10
Qq_r.G8	76.22	76.50
R_clo.I8	196.39	232.60
H_clo.H8	66.80	67.00
I_clo.C8	380.63	380.80
I_unc.S8	178.68	178.80
A.A2	1.81	1.81
S_h.A3	0.27	0.40
PA_clo.A4	1.28	1.28
PA_unc.A5	0.54	0.54
A_r.A6	1.42	1.42
A_kunc.A7	0.00	0.00
A_clo.A8	0.04	0.04
PT_clo.A9	0.70	0.70
E_a.E2	20.22	20.22
E_s.E3	40.29	40.34
W0	0.79	0.79
f_pd.F2	0.57	0.57

		Myrup_Morgan_4_20
f_cl.F3	0.46	0.46
V.V4	1.49	1.49
h_c.H3	8.39	8.39
T_clo.T9	28.06	28.08
T8	22.30	NA
Y0	0.19	0.19
z.K0	32.58	32.58
U0	1.69	1.70
Q0	0.19	0.19
Y	-0.91	NA
sweat.g.h	372.12	NA
evap.g.h	401.43	NA

```
plot(out ~ Myrup_Morgan_4_20, ylab = "MANMO_R", xlab = "MANMO_TC")
abline(0, 1)
```

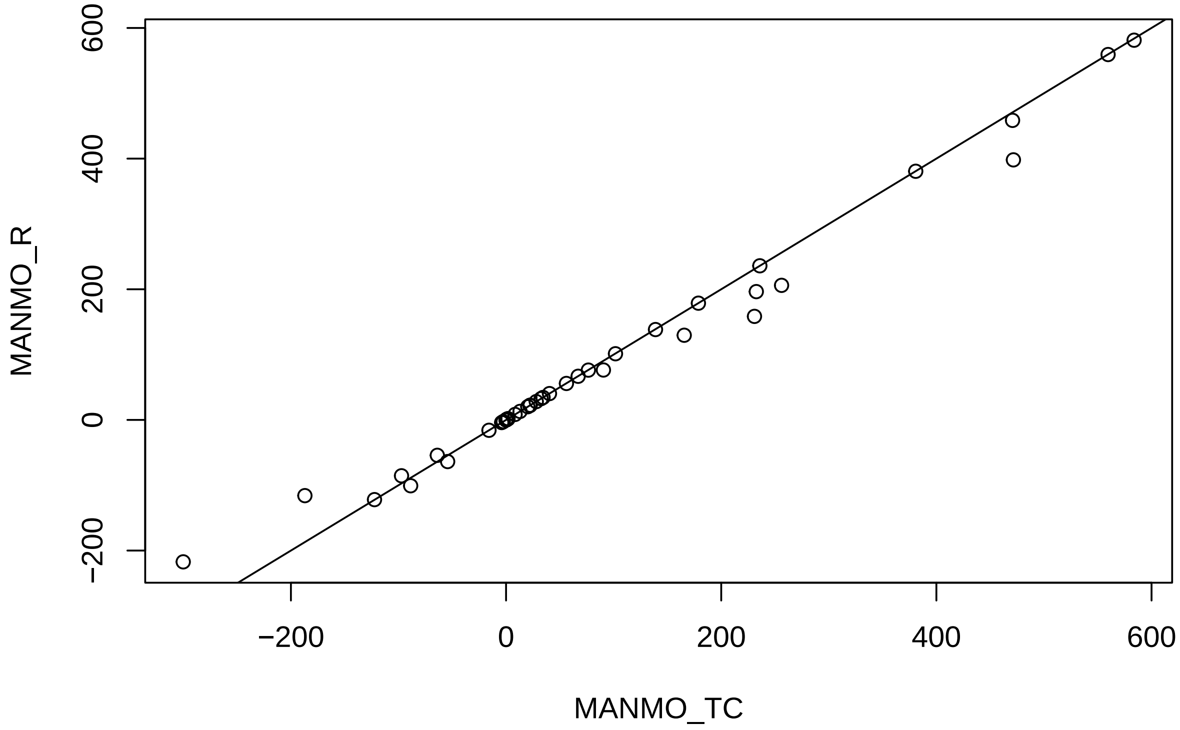

Run buggy version without bisection method.

```
outb <- MANMO_R_area_bug(SI.mode = 0, Ht.H4 = Ht.H4, Wt.W4 = Wt.W4,
    a_skn.B4 = a_skn.B4, e_skin.Y4 = e_skin.Y4, K6 = K6, K7 = K7,
    O2 = O2, V3 = V3, G_m.G2 = G_m.G2, K8 = K8, a_clo.B5 = a_clo.B5,
    e_clo.Y5 = e_clo.Y5, K3 = K3, D3 = D3, CLO.C4 = CLO.C4, month.Z2 = month.Z2,
    day.Z3 = day.Z3, time.Z4 = time.Z4, lat.Z5 = lat.Z5, lon.Z6 = lon.Z6,
```

```
    TZone.Z7 = TZone.Z7, trans.R2 = trans.R2, a.B3 = a.B3, e_sfc.Y3 = e_sfc.Y3,
    k.C2 = k.C2, kk.C3 = kk.C3, n.N2 = n.N2, T_a.T0 = T_a.T0,
    T_sky.T2 = T_a.T0, T_gnd.T7 = T_a.T0, T_m.S2 = T_m.S2, RH.H2 = RH.H2,
    ff.V2 = ff.V2, dd.V5 = dd.V5, k_x.K2 = k_x.K2, d_x.D2 = d_x.D2,
    CLO.mode = CLO.mode, mode = mode, iterate = 0, Tskin1.T = 34.38,
    W = 0.35 * 0.94 + 0.06)
outb <- t(round(outb, 2))
kable(cbind(Myrup_Morgan_4_20, out, outb))
```

	Myrup_Morgan_4_20		
Tskin	34.38	34.35	34.38
W	0.35	0.34	0.35
T_a.T0	22.30	22.30	22.30
T_gnd.T7	22.30	22.30	22.30
T_sky.T2	22.30	22.30	22.30
T_m.S2	22.30	22.30	22.30
ff.V2	1.00	1.00	1.00
RH.H2	0.73	0.73	0.73
Q_h.Q2	NA	1.08	1.08
q_h.Q7	NA	0.13	0.13
N	13.00	13.00	0.00
M_m.M	235.90	235.92	235.92
R_m.R	256.10	205.99	254.64
I_m.I	-88.70	-100.79	-100.97
E_m.E	-300.10	-217.29	-300.06
H_m.H	-122.30	-122.03	-122.28
D_m.D	-2.70	-2.72	-2.72
R_munc.R1	90.50	76.40	90.11
Q_m.Q1	230.90	158.49	229.92
q_v.D1	101.70	101.28	101.28
q_g.G1	138.90	138.32	138.32
R_mclo.M1	165.60	129.59	164.53
I_e.E1	470.80	458.53	458.53
I.I1	559.60	559.32	559.50
E_r.L1	-15.90	-15.87	-15.87
E_clo.C1	-187.00	-115.91	-186.98
E_unc.U1	-97.20	-85.51	-97.21
H_r.P1	-4.10	-4.07	-4.07
H_unc.K1	-63.90	-54.19	-54.30
H_clo.S1	-54.30	-63.77	-63.91
D_unc.A1	0.00	0.00	0.00
D_clo.B1	-2.70	-2.72	-2.72
R8	471.60	398.09	469.52
Q8	583.80	581.27	581.27
D8	56.10	55.81	55.81
Qq_r.G8	76.50	76.22	76.22
R_clo.I8	232.60	196.39	231.62
H_clo.H8	67.00	66.80	67.09
I_clo.C8	380.80	380.63	380.76
I_unc.S8	178.80	178.68	178.74
A.A2	1.81	1.81	1.81
S_h.A3	0.40	0.27	0.40

Myrup_Morgan_4_20			
PA_clo.A4	1.28	1.28	1.28
PA_unc.A5	0.54	0.54	0.54
A_r.A6	1.42	1.42	1.42
A_kunc.A7	0.00	0.00	0.00
A_clo.A8	0.04	0.04	0.04
PT_clo.A9	0.70	0.70	0.70
E_a.E2	20.22	20.22	20.22
E_s.E3	40.34	40.29	40.34
W0	0.79	0.79	0.79
f_pd.F2	0.57	0.57	0.57
f_cl.F3	0.46	0.46	0.46
V.V4	1.49	1.49	1.49
h_c.H3	8.39	8.39	8.39
T_clo.T9	28.08	28.06	28.09
T8	NA	22.30	22.30
Y0	0.19	0.19	0.19
z.K0	32.58	32.58	32.58
U0	1.70	1.69	1.69
Q0	0.19	0.19	0.19
Y	NA	-0.91	-35.47
sweat.g.h	NA	372.12	525.03
evap.g.h	NA	401.43	554.34

```r
plot(out ~ Myrup_Morgan_4_20, ylab = "MANMO_R", xlab = "MANMO_TC")
points(outb ~ Myrup_Morgan_4_20, col = "red", pch = 2, ylab = "MANMO_R",
    xlab = "MANMO_TC")
abline(0, 1)
```

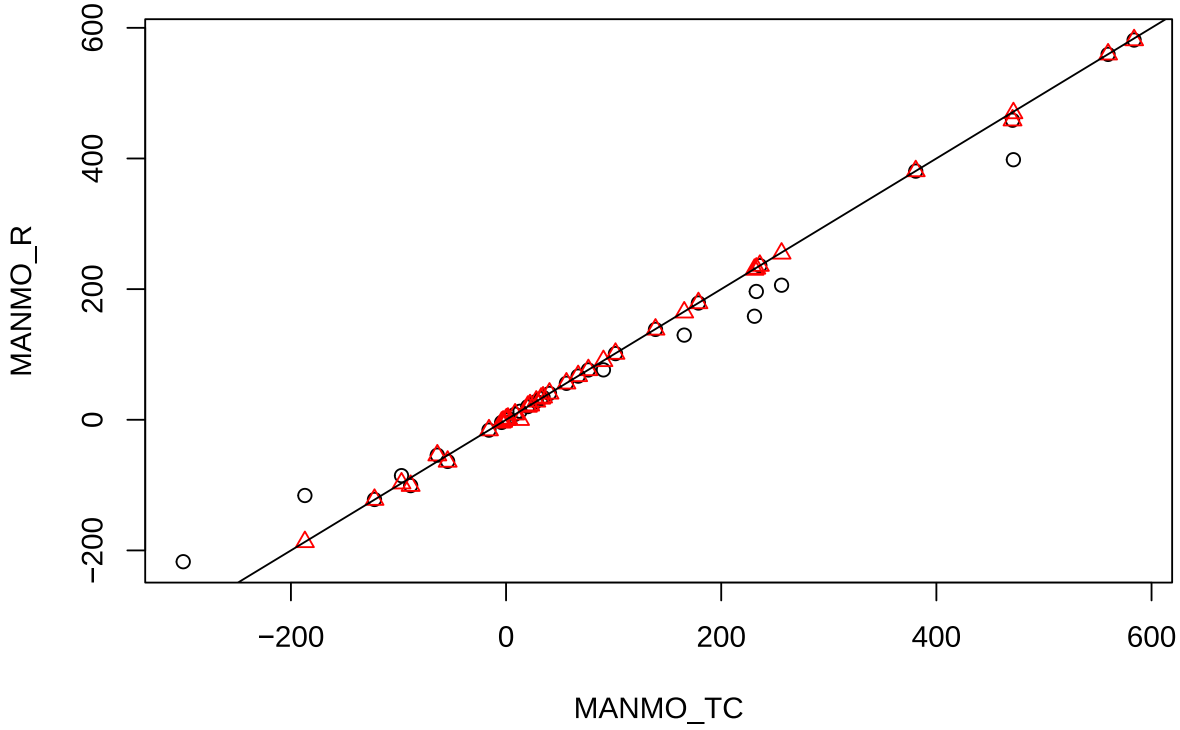

Run buggy version with bisection method.

```
outc <- MANMO_R_area_bug(SI.mode = 0, Ht.H4 = Ht.H4, Wt.W4 = Wt.W4,
    a_skn.B4 = a_skn.B4, e_skin.Y4 = e_skin.Y4, K6 = K6, K7 = K7,
    O2 = O2, V3 = V3, G_m.G2 = G_m.G2, K8 = K8, a_clo.B5 = a_clo.B5,
    e_clo.Y5 = e_clo.Y5, K3 = K3, D3 = D3, CLO.C4 = CLO.C4, month.Z2 = month.Z2,
    day.Z3 = day.Z3, time.Z4 = time.Z4, lat.Z5 = lat.Z5, lon.Z6 = lon.Z6,
    TZone.Z7 = TZone.Z7, trans.R2 = trans.R2, a.B3 = a.B3, e_sfc.Y3 = e_sfc.Y3,
    k.C2 = k.C2, kk.C3 = kk.C3, n.N2 = n.N2, Q_h.Q2 = Q_h.Q2,
    q_h.Q7 = q_h.Q7, T_a.T0 = T_a.T0, T_sky.T2 = T_sky.T2, T_gnd.T7 = T_gnd.T7,
    T_clo.T9 = T_clo.T9, T_m.S2 = T_m.S2, RH.H2 = RH.H2, ff.V2 = ff.V2,
    dd.V5 = dd.V5, k_x.K2 = k_x.K2, d_x.D2 = d_x.D2, CLO.mode = CLO.mode,
    mode = mode, iterate = 1)
outc <- t(round(outc, 2))
kable(cbind(Myrup_Morgan_4_20, out, outb, outc))
```

	Myrup_Morgan_4_20			
Tskin	34.38	34.35	34.38	35.34
W	0.35	0.34	0.35	0.58
T_a.T0	22.30	22.30	22.30	22.30
T_gnd.T7	22.30	22.30	22.30	22.30
T_sky.T2	22.30	22.30	22.30	22.30
T_m.S2	22.30	22.30	22.30	22.30
ff.V2	1.00	1.00	1.00	1.00

	Myrup_Morgan_4_20			
RH.H2	0.73	0.73	0.73	0.73
Q_h.Q2	NA	1.08	1.08	1.35
q_h.Q7	NA	0.13	0.13	0.24
N	13.00	13.00	0.00	12.00
M_m.M	235.90	235.92	235.92	235.92
R_m.R	256.10	205.99	254.64	243.55
I_m.I	-88.70	-100.79	-100.97	153.44
E_m.E	-300.10	-217.29	-300.06	-498.44
H_m.H	-122.30	-122.03	-122.28	-131.63
D_m.D	-2.70	-2.72	-2.72	-2.94
R_munc.R1	90.50	76.40	90.11	90.11
Q_m.Q1	230.90	158.49	229.92	229.92
q_v.D1	101.70	101.28	101.28	101.28
q_g.G1	138.90	138.32	138.32	138.32
R_mclo.M1	165.60	129.59	164.53	153.44
I_e.E1	470.80	458.53	458.53	458.53
I.I1	559.60	559.32	559.50	566.60
E_r.L1	-15.90	-15.87	-15.87	-15.87
E_clo.C1	-187.00	-115.91	-186.98	-317.51
E_unc.U1	-97.20	-85.51	-97.21	-165.07
H_r.P1	-4.10	-4.07	-4.07	-4.07
H_unc.K1	-63.90	-54.19	-54.30	-58.60
H_clo.S1	-54.30	-63.77	-63.91	-68.96
D_unc.A1	0.00	0.00	0.00	0.00
D_clo.B1	-2.70	-2.72	-2.72	-2.94
R8	471.60	398.09	469.52	469.52
Q8	583.80	581.27	581.27	581.27
D8	56.10	55.81	55.81	55.81
Qq_r.G8	76.50	76.22	76.22	76.22
R_clo.I8	232.60	196.39	231.62	231.62
H_clo.H8	67.00	66.80	67.09	78.18
I_clo.C8	380.80	380.63	380.76	385.62
I_unc.S8	178.80	178.68	178.74	180.98
A.A2	1.81	1.81	1.81	1.81
S_h.A3	0.40	0.27	0.40	0.40
PA_clo.A4	1.28	1.28	1.28	1.28
PA_unc.A5	0.54	0.54	0.54	0.54
A_r.A6	1.42	1.42	1.42	1.42
A_kunc.A7	0.00	0.00	0.00	0.00
A_clo.A8	0.04	0.04	0.04	0.04
PT_clo.A9	0.70	0.70	0.70	0.70
E_a.E2	20.22	20.22	20.22	20.22
E_s.E3	40.34	40.29	40.34	42.16
W0	0.79	0.79	0.79	0.79
f_pd.F2	0.57	0.57	0.57	0.57
f_cl.F3	0.46	0.46	0.46	0.46
V.V4	1.49	1.49	1.49	1.49
h_c.H3	8.39	8.39	8.39	8.39
T_clo.T9	28.08	28.06	28.09	29.05
T8	NA	22.30	22.30	22.30
Y0	0.19	0.19	0.19	0.19
z.K0	32.58	32.58	32.58	32.58

	Myrup_Morgan_4_20			
U0	1.70	1.69	1.69	1.69
Q0	0.19	0.19	0.19	0.19
Y	NA	-0.91	-35.47	-0.09
sweat.g.h	NA	372.12	525.03	891.52
evap.g.h	NA	401.43	554.34	920.84

```
plot(out ~ Myrup_Morgan_4_20, ylab = "MANMO_R", xlab = "MANMO_TC")
points(outb ~ Myrup_Morgan_4_20, col = "red", pch = 2, ylab = "MANMO_R",
    xlab = "MANMO_TC")
points(outc ~ Myrup_Morgan_4_20, col = "blue", pch = 16, ylab = "MANMO_R",
    xlab = "MANMO_TC")
abline(0, 1)
```

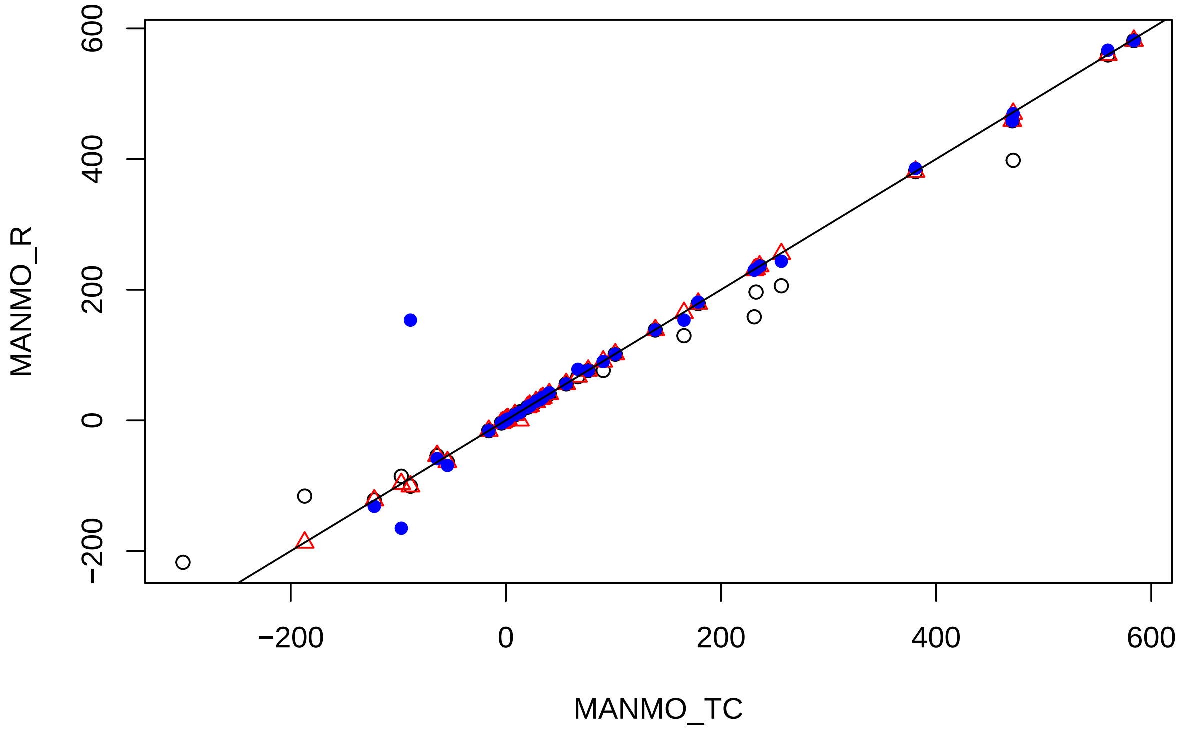

Again, gets worse again when bisection turned on.

Table 4-21

MANMO in SDM (standard input) mode, for a 180 cm tall, 64 kg male aged 22 on 4th August 1971.

```
# mode of operation

mode <- 2  # 1 climatological, 2 standard innputs
```

```
CLO.mode <- 1  # use empirical clothes temp function (0) or fix clothing temp (1)

# physiological inputs

age.Y6 <- 22  # age, y
sex.Y7 <- 1  # sex, 1 = male, 2 = female
Ht.H4 <- 180  # height, cm
Wt.W4 <- 64  # weight, kg

a_skn.B4 <- 0.35  # albedo of skin, -
e_skin.Y4 <- 0.98  # emissivity of skin, -
K6 <- 0.78  # radiation area coefficient, -
K7 <- 1e-11  # skin contact area coefficient, -
O2 <- 0.1  # orientaion of man, degrees
V3 <- 1.5  # movement of man, m/s
G_m.G2 <- 130  # activity (metabolic costs), Kcal / (h m2)
K8 <- 0.023  # clothes contact area coefficient, -
a_clo.B5 <- 0.3  # albedo of clothes, -
e_clo.Y5 <- 0.95  # emissivity of clothes, -
K3 <- c(0.014, 0.01, 0.072, 1)  # conductivity of clothes, 4 layers, Kcal m / (m2 h C)
D3 <- c(0.001, 0.001, 0.001, 1e-11)  # thickness of clothing, 4 layers, m
CLO.C4 <- 0.6

# environmental inputs

month.Z2 <- 8  # month, -
day.Z3 <- 4  # day, -
time.Z4 <- 10.25  # time, LST
lat.Z5 <- 38.54  # latitude, degrees
lon.Z6 <- 121.78  # longitude, degrees
TZone.Z7 <- 8  # time zone, -
trans.R2 <- 0.6  # transmissivity coefficient, -
a.B3 <- 0.22  # albedo of surface, -
e_sfc.Y3 <- 1  # emissivity of surface, -
k.C2 <- 1e-11  # cloud type code, -
kk.C3 <- 1e-11  # cloud type code, -
n.N2 <- 1e-11  # cloud amount, tenths
Q_h.Q2 <- 1.08  # direct (actually direct + diffuse) radiation, ly / min = Cal/cm2/min = 4.1868 J / cm2
q_h.Q7 <- 0.133  # diffuse radiation, ly / min
T_a.T0 <- 22.3  # air temp, deg C
T_sky.T2 <- 2  # sky temp, deg C
T_gnd.T7 <- 36  # ground temp, deg C
T_clo.T9 <- 36  # clothing temp, deg C
T_m.S2 <- 36  # substrate temp, deg C
RH.H2 <- 0.73  # relative humidity, -
ff.V2 <- 1  # wind speed, m/s
dd.V5 <- 250  # wind direction, degrees
k_x.K2 <- 0.5  # conductivity substrate, Kcal m / (m2 h deg C)
d_x.D2 <- 0.01  # heat thickness substrate, m

out <- MANMO_R(SI.mode = 0, Ht.H4 = Ht.H4, Wt.W4 = Wt.W4, a_skn.B4 = a_skn.B4,
    e_skin.Y4 = e_skin.Y4, K6 = K6, K7 = K7, O2 = O2, V3 = V3,
    G_m.G2 = G_m.G2, K8 = K8, a_clo.B5 = a_clo.B5, e_clo.Y5 = e_clo.Y5,
```

```
    K3 = K3, D3 = D3, CLO.C4 = CLO.C4, month.Z2 = month.Z2, day.Z3 = day.Z3,
    time.Z4 = time.Z4, lat.Z5 = lat.Z5, lon.Z6 = lon.Z6, TZone.Z7 = TZone.Z7,
    trans.R2 = trans.R2, a.B3 = a.B3, e_sfc.Y3 = e_sfc.Y3, k.C2 = k.C2,
    kk.C3 = kk.C3, n.N2 = n.N2, Q_h.Q2 = Q_h.Q2, q_h.Q7 = q_h.Q7,
    T_a.T0 = T_a.T0, T_sky.T2 = T_sky.T2, T_gnd.T7 = T_gnd.T7,
    T_clo.T9 = T_clo.T9, T_m.S2 = T_m.S2, RH.H2 = RH.H2, ff.V2 = ff.V2,
    dd.V5 = dd.V5, k_x.K2 = k_x.K2, d_x.D2 = d_x.D2, CLO.mode = CLO.mode,
    mode = mode)
out <- t(round(out, 2))
Myrup_Morgan_4_21 <- c(33.66, 0.17, 22.3, 36, 2, 36, 1, 0.73,
    1.08, 0.133, 13, 235.9, 141, -96.3, -166.5, -115, 0.5, 84,
    236, 72.4, 129.4, 57.2, 503.8, 599.5, -15.9, -99.1, -51.5,
    -4.1, -60.1, -51.1, 0, 0.5, 437.8, 596.6, 39.9, 71.3, 216,
    158.8, 422.4, 177.1, 1.81, 0.4, 1.28, 0.54, 1.42, 0, 0.04,
    0.7, 20.22, 38.96, NA, 0.57, 0.46, 1.49, 8.39, 36, 19, 0.19,
    32.58, NA, NA, NA, NA, NA)
kable(cbind(out, Myrup_Morgan_4_21))
```

		Myrup_Morgan_4_21
Tskin	33.70	33.660
W	0.18	0.170
T_a.T0	22.30	22.300
T_gnd.T7	36.00	36.000
T_sky.T2	2.00	2.000
T_m.S2	36.00	36.000
ff.V2	1.00	1.000
RH.H2	0.73	0.730
Q_h.Q2	1.08	1.080
q_h.Q7	0.13	0.133
N	13.00	13.000
M_m.M	235.92	235.900
R_m.R	90.98	141.000
I_m.I	-96.41	-96.300
E_m.E	-115.42	-166.500
H_m.H	-115.66	-115.000
D_m.D	0.52	0.500
R_munc.R1	69.94	84.000
Q_m.Q1	162.68	236.000
q_v.D1	72.41	72.400
q_g.G1	129.36	129.400
R_mclo.M1	21.03	57.200
I_e.E1	503.12	503.800
I.I1	599.53	599.500
E_r.L1	-15.87	-15.900
E_clo.C1	-57.29	-99.100
E_unc.U1	-42.26	-51.500
H_r.P1	-4.07	-4.100
H_unc.K1	-51.26	-60.100
H_clo.S1	-60.33	-51.100
D_unc.A1	0.00	0.000
D_clo.B1	0.52	0.500
R8	364.44	437.800

		Myrup_Morgan_4_21
Q8	596.61	596.600
D8	39.90	39.900
Qq_r.G8	71.28	71.300
R_clo.I8	179.79	216.000
H_clo.H8	158.75	158.800
I_clo.C8	422.36	422.400
I_unc.S8	177.18	177.100
A.A2	1.81	1.810
S_h.A3	0.27	0.400
PA_clo.A4	1.28	1.280
PA_unc.A5	0.54	0.540
A_r.A6	1.42	1.420
A_kunc.A7	0.00	0.000
A_clo.A8	0.04	0.040
PT_clo.A9	0.70	0.700
E_a.E2	20.22	20.220
E_s.E3	39.04	38.960
W0	0.79	NA
f_pd.F2	0.57	0.570
f_cl.F3	0.46	0.460
V.V4	1.49	1.490
h_c.H3	8.39	8.390
T_clo.T9	36.00	36.000
T8	19.00	19.000
Y0	0.19	0.190
z.K0	32.58	32.580
U0	NA	NA
Q0	NA	NA
Y	-0.08	NA
sweat.g.h	183.92	NA
evap.g.h	213.23	NA

```r
plot(out ~ Myrup_Morgan_4_21, ylab = "MANMO_R", xlab = "MANMO_TC")
abline(0, 1)
```

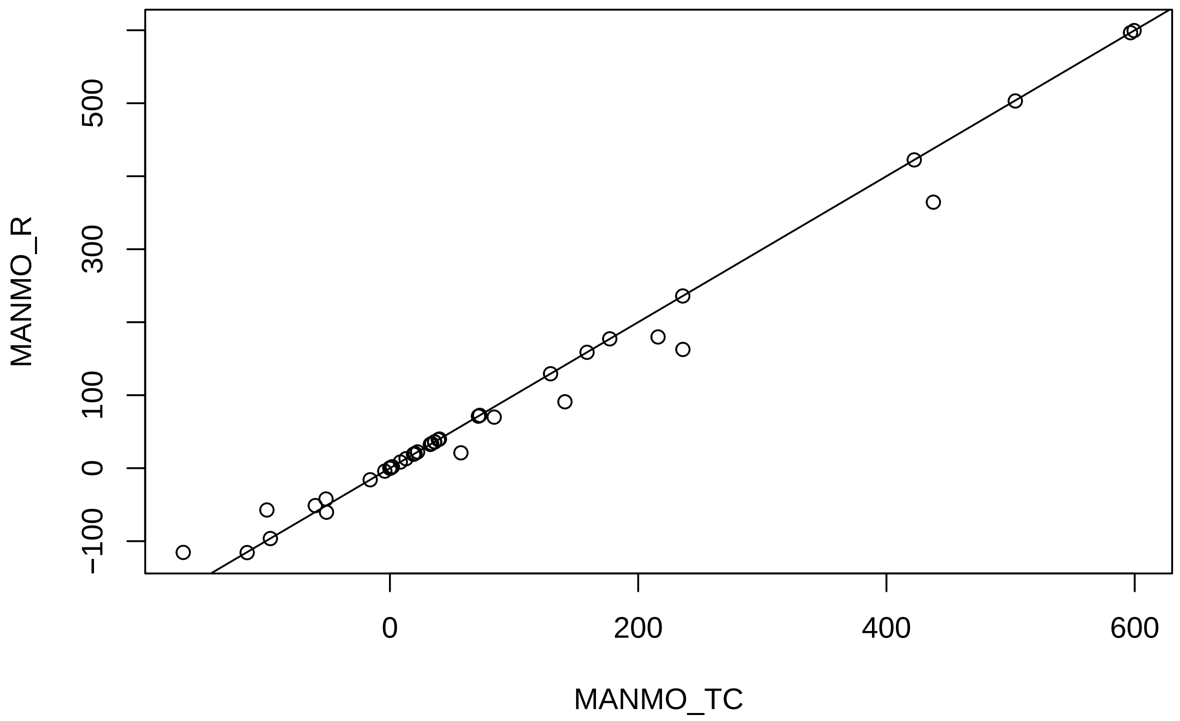

Run buggy version with bisection method and results are spot on.

```
outb <- MANMO_R_area_bug(SI.mode = 0, Ht.H4 = Ht.H4, Wt.W4 = Wt.W4,
    a_skn.B4 = a_skn.B4, e_skin.Y4 = e_skin.Y4, K6 = K6, K7 = K7,
    O2 = O2, V3 = V3, G_m.G2 = G_m.G2, K8 = K8, a_clo.B5 = a_clo.B5,
    e_clo.Y5 = e_clo.Y5, K3 = K3, D3 = D3, CLO.C4 = CLO.C4, month.Z2 = month.Z2,
    day.Z3 = day.Z3, time.Z4 = time.Z4, lat.Z5 = lat.Z5, lon.Z6 = lon.Z6,
    TZone.Z7 = TZone.Z7, trans.R2 = trans.R2, a.B3 = a.B3, e_sfc.Y3 = e_sfc.Y3,
    k.C2 = k.C2, kk.C3 = kk.C3, n.N2 = n.N2, Q_h.Q2 = Q_h.Q2,
    q_h.Q7 = q_h.Q7, T_a.T0 = T_a.T0, T_sky.T2 = T_sky.T2, T_gnd.T7 = T_gnd.T7,
    T_clo.T9 = T_clo.T9, T_m.S2 = T_m.S2, RH.H2 = RH.H2, ff.V2 = ff.V2,
    dd.V5 = dd.V5, k_x.K2 = k_x.K2, d_x.D2 = d_x.D2, CLO.mode = CLO.mode,
    mode = mode, iterate = 1)
outb <- t(round(outb, 2))
kable(cbind(Myrup_Morgan_4_21, out, outb))
```

	Myrup_Morgan_4_21		
Tskin	33.660	33.70	33.67
W	0.170	0.18	0.17
T_a.T0	22.300	22.30	22.30
T_gnd.T7	36.000	36.00	36.00
T_sky.T2	2.000	2.00	2.00
T_m.S2	36.000	36.00	36.00
ff.V2	1.000	1.00	1.00

	Myrup_Morgan_4_21		
RH.H2	0.730	0.73	0.73
Q_h.Q2	1.080	1.08	1.08
q_h.Q7	0.133	0.13	0.13
N	13.000	13.00	12.00
M_m.M	235.900	235.92	235.92
R_m.R	141.000	90.98	141.21
I_m.I	-96.300	-96.41	-96.34
E_m.E	-166.500	-115.42	-168.39
H_m.H	-115.000	-115.66	-115.34
D_m.D	0.500	0.52	3.15
R_munc.R1	84.000	69.94	84.01
Q_m.Q1	236.000	162.68	235.99
q_v.D1	72.400	72.41	72.41
q_g.G1	129.400	129.36	129.36
R_mclo.M1	57.200	21.03	57.20
I_e.E1	503.800	503.12	503.12
I.I1	599.500	599.53	599.46
E_r.L1	-15.900	-15.87	-15.87
E_clo.C1	-99.100	-57.29	-100.35
E_unc.U1	-51.500	-42.26	-52.17
H_r.P1	-4.100	-4.07	-4.07
H_unc.K1	-60.100	-51.26	-51.12
H_clo.S1	-51.100	-60.33	-60.16
D_unc.A1	0.000	0.00	0.00
D_clo.B1	0.500	0.52	3.15
R8	437.800	364.44	437.75
Q8	596.600	596.61	596.61
D8	39.900	39.90	39.90
Qq_r.G8	71.300	71.28	71.28
R_clo.I8	216.000	179.79	215.95
H_clo.H8	158.800	158.75	158.75
I_clo.C8	422.400	422.36	422.36
I_unc.S8	177.100	177.18	177.10
A.A2	1.810	1.81	1.81
S_h.A3	0.400	0.27	0.40
PA_clo.A4	1.280	1.28	1.28
PA_unc.A5	0.540	0.54	0.54
A_r.A6	1.420	1.42	1.42
A_kunc.A7	0.000	0.00	0.00
A_clo.A8	0.040	0.04	0.04
PT_clo.A9	0.700	0.70	0.70
E_a.E2	20.220	20.22	20.22
E_s.E3	38.960	39.04	38.98
W0	NA	0.79	0.79
f_pd.F2	0.570	0.57	0.57
f_cl.F3	0.460	0.46	0.46
V.V4	1.490	1.49	1.49
h_c.H3	8.390	8.39	8.39
T_clo.T9	36.000	36.00	36.00
T8	19.000	19.00	19.00
Y0	0.190	0.19	0.19
z.K0	32.580	32.58	32.58

Myrup_Morgan_4_21			
U0	NA	NA	NA
Q0	NA	NA	NA
Y	NA	-0.08	0.21
sweat.g.h	NA	183.92	281.78
evap.g.h	NA	213.23	311.09

```r
plot(out ~ Myrup_Morgan_4_21, ylab = "MANMO_R", xlab = "MANMO_TC")
points(outb ~ Myrup_Morgan_4_21, col = "red", pch = 2, ylab = "MANMO_R",
    xlab = "MANMO_TC")
abline(0, 1)
```

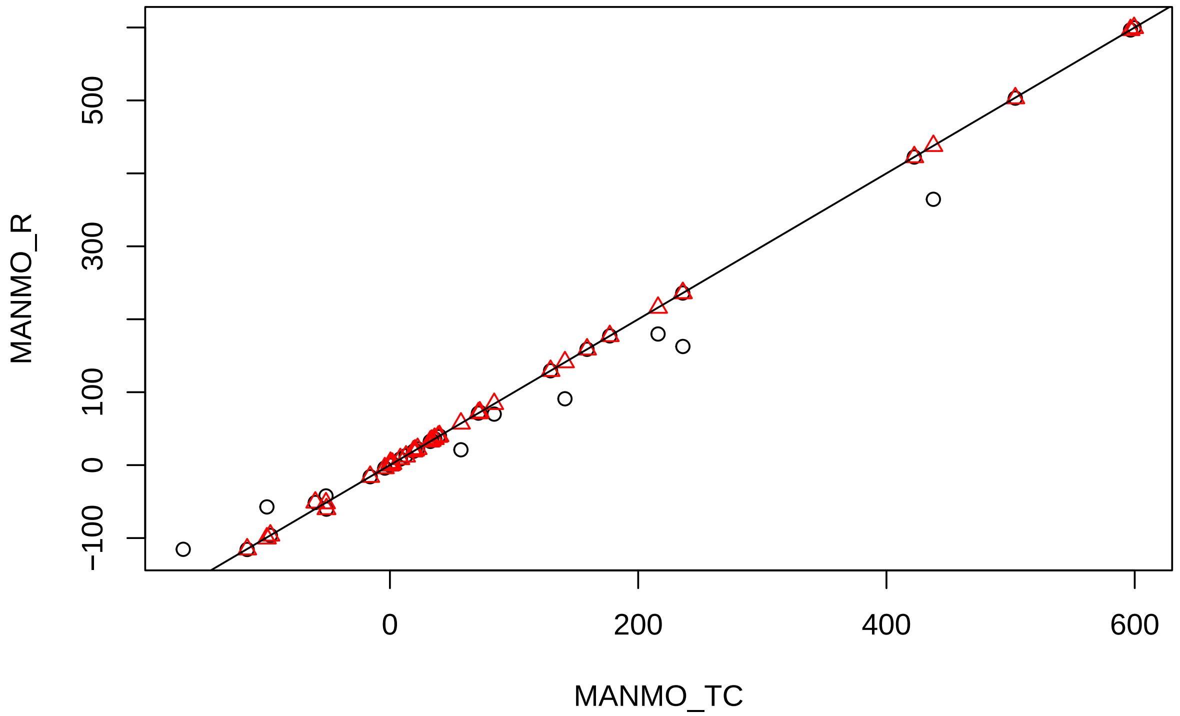


```r
########################### Fig. 4-3
########################### ################################

# mode of operation

mode <- 1   # 1 climatological, 2 standard inputs
CLO.mode <- 1   # use empirical clothes temp function (0) or fix clothing temp (1)

# physiological inputs

age.Y6 <- 22   # age, y
```

```
sex.Y7 <- 1  # sex, 1 = male, 2 = female
Ht.H4 <- 180  # height, cm
Wt.W4 <- 64  # weight, kg

a_skn.B4 <- 0.35  # albedo of skin, -
e_skin.Y4 <- 0.98  # emissivity of skin, -
K6 <- 0.78  # radiation area coefficient, -
K7 <- 1e-11  # skin contact area coefficient, -
O2 <- 0.1  # orientaion of man, degrees
V3 <- 0  # movement of man, m/s
G_m.G2 <- 130  # activity (metabolic costs), Kcal / (h m2)
K8 <- 0.023  # clothes contact area coefficient, -
a_clo.B5 <- 0.3  # albedo of clothes, -
e_clo.Y5 <- 0.95  # emissivity of clothes, -
K3 <- c(0.014, 0.01, 0.072, 1)  # conductivity of clothes, 4 layers, Kcal m / (m2 h C)
D3 <- c(0.001, 0.001, 0.001, 1e-11)  # thickness of clothing, 4 layers, m
CLO.C4 <- 0.6

# environmental inputs

month.Z2 <- 7  # month, -
day.Z3 <- 21  # day, -
time.Z4 <- 13  # time, LST
lat.Z5 <- 38.54  # latitude, degrees
lon.Z6 <- 121.78  # longitude, degrees
TZone.Z7 <- 8  # time zone, -
trans.R2 <- 0.6  # transmissivity coefficient, -
a.B3 <- 0.2  # albedo of surface, -
e_sfc.Y3 <- 1  # emissivity of surface, -
k.C2 <- 0  # cloud type code, -
kk.C3 <- 0  # cloud type code, -
n.N2 <- 0  # cloud amount, tenths
Q_h.Q2 <- 1.35  # direct (actually direct + diffuse) radiation, ly / min = Cal/cm2/min = 4.1868 J / cm2
q_h.Q7 <- 0.24  # diffuse radiation, ly / min
T_a.T0 <- 36.2  # air temp, deg C
T_sky.T2 <- 24  # sky temp, deg C
T_gnd.T7 <- 57  # ground temp, deg C
T_clo.T9 <- 41.2  # clothing temp, deg C
T_m.S2 <- 57  # substrate temp, deg C
RH.H2 <- 0.28  # relative humidity, -
ff.V2 <- 5  # wind speed, m/s
dd.V5 <- 180  # wind direction, degrees
k_x.K2 <- 0.3  # conductivity substrate, Kcal m / (m2 h deg C)
d_x.D2 <- 0.01  # heat thickness substrate, m



albedo <- seq(0.1, 0.6, 0.01)
wind <- c(0.5, 1, 2, 4, 8)
cloud <- c(0, 1)  # cloud amount, tenths
k.C2 <- 0.75  # cloud type code, -

for (j in 1:length(cloud)) {
```

```
    for (i in 1:length(wind)) {

        ff.V2 <- wind[i]  # wind speed, m/s
        n.N2 <- cloud[j]  #
        out <- lapply(1:length(albedo), function(x) {
            MANMO_R(SI.mode = 0, Ht.H4 = Ht.H4, Wt.W4 = Wt.W4,
                a_skn.B4 = a_skn.B4, e_skin.Y4 = e_skin.Y4, K6 = K6,
                K7 = K7, O2 = O2, V3 = V3, G_m.G2 = G_m.G2, K8 = K8,
                a_clo.B5 = albedo[x], e_clo.Y5 = e_clo.Y5, K3 = K3,
                D3 = D3, CLO.C4 = CLO.C4, month.Z2 = month.Z2,
                day.Z3 = day.Z3, time.Z4 = time.Z4, lat.Z5 = lat.Z5,
                lon.Z6 = lon.Z6, TZone.Z7 = TZone.Z7, trans.R2 = trans.R2,
                a.B3 = a.B3, e_sfc.Y3 = e_sfc.Y3, k.C2 = k.C2,
                kk.C3 = kk.C3, n.N2 = n.N2, Q_h.Q2 = Q_h.Q2,
                q_h.Q7 = q_h.Q7, T_a.T0 = T_a.T0, T_sky.T2 = T_sky.T2,
                T_gnd.T7 = T_gnd.T7, T_clo.T9 = T_clo.T9, T_m.S2 = T_m.S2,
                RH.H2 = RH.H2, ff.V2 = ff.V2, dd.V5 = dd.V5,
                k_x.K2 = k_x.K2, d_x.D2 = d_x.D2, CLO.mode = CLO.mode,
                mode = mode)
        })
        out <- do.call("rbind", lapply(out, data.frame))

        if (i == 1 & j == 1) {
            plot(out$R_m.R ~ albedo, type = "l", ylim = c(0,
                300), ylab = "SHORTWAVE RADIATION, Rm, (KCAL / H)",
                xlab = "ALBEDO OF CLOTHING")
        } else {
            if (j == 1) {
                points(out$R_m.R ~ albedo, type = "l")
            } else {
                points(out$R_m.R ~ albedo, type = "l", lty = 2)
            }
        }

    }
}
```

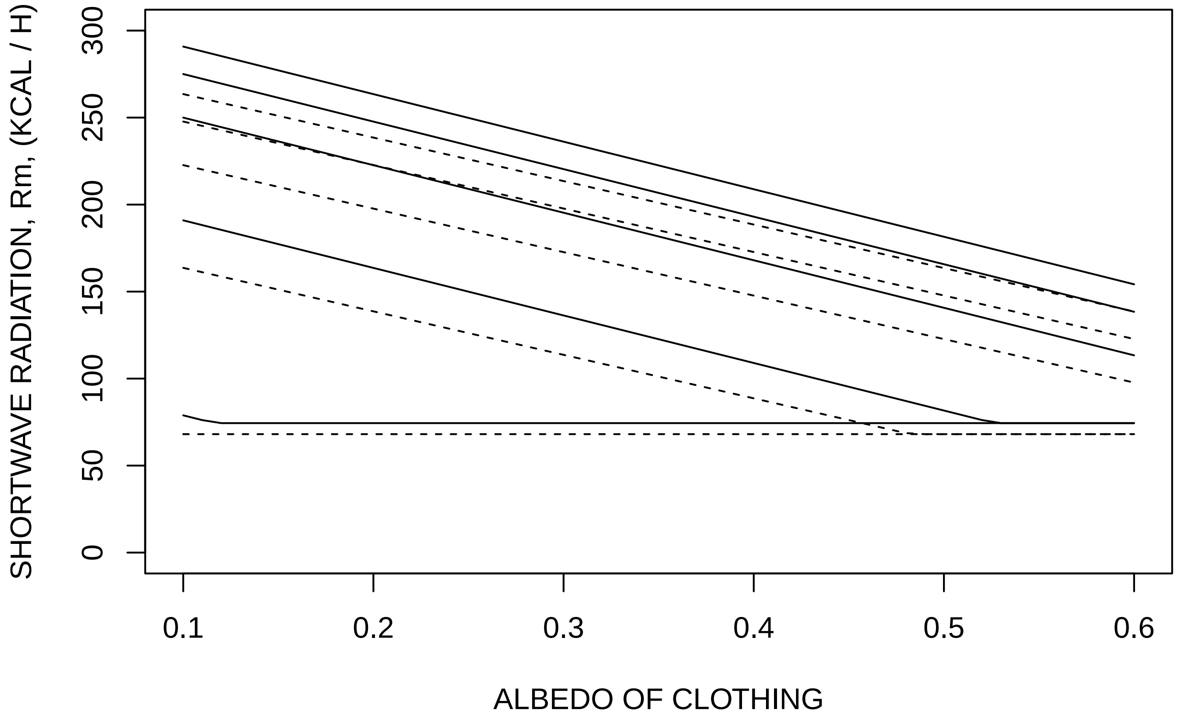


```
############################ Fig. 4-4
############################ #################################

# mode of operation

mode <- 1  # 1 climatological, 2 standard inputs
CLO.mode <- 1  # use empirical clothes temp function (0) or fix clothing temp (1)

# physiological inputs

age.Y6 <- 22  # age, y
sex.Y7 <- 1  # sex, 1 = male, 2 = female
Ht.H4 <- 180  # height, cm
Wt.W4 <- 64  # weight, kg

a_skn.B4 <- 0.35  # albedo of skin, -
e_skin.Y4 <- 0.98  # emissivity of skin, -
K6 <- 0.78  # radiation area coefficient, -
K7 <- 1e-11  # skin contact area coefficient, -
O2 <- 0.1  # orientaion of man, degrees
V3 <- 0  # movement of man, m/s
G_m.G2 <- 130  # activity (metabolic costs), Kcal / (h m2)
K8 <- 0.023  # clothes contact area coefficient, -
a_clo.B5 <- 0.3  # albedo of clothes, -
e_clo.Y5 <- 0.95  # emissivity of clothes, -
```

```r
K3 <- c(0.014, 0.01, 0.072, 1)  # conductivity of clothes, 4 layers, Kcal m / (m2 h C)
D3 <- c(0.001, 0.001, 0.001, 1e-11)  # thickness of clothing, 4 layers, m
CLO.C4 <- 0.6

# environmental inputs

month.Z2 <- 7  # month, -
day.Z3 <- 21  # day, -
time.Z4 <- 13  # time, LST
lat.Z5 <- 38.54  # latitude, degrees
lon.Z6 <- 121.78  # longitude, degrees
TZone.Z7 <- 8  # time zone, -
trans.R2 <- 0.6  # transmissivity coefficient, -
a.B3 <- 0.2  # albedo of surface, -
e_sfc.Y3 <- 1  # emissivity of surface, -
k.C2 <- 0  # cloud type code, -
kk.C3 <- 0  # cloud type code, -
n.N2 <- 0  # cloud amount, tenths
Q_h.Q2 <- 1.35  # direct (actually direct + diffuse) radiation, ly / min = Cal/cm2/min = 4.1868 J / cm2
q_h.Q7 <- 0.24  # diffuse radiation, ly / min
T_a.T0 <- 36.1  # air temp, deg C
T_sky.T2 <- T_a.T0  # sky temp, deg C
T_gnd.T7 <- T_a.T0  # ground temp, deg C
T_clo.T9 <- T_a.T0  # clothing temp, deg C
T_m.S2 <- T_a.T0  # substrate temp, deg C
RH.H2 <- 0.28  # relative humidity, -
ff.V2 <- 5  # wind speed, m/s
dd.V5 <- 180  # wind direction, degrees
k_x.K2 <- 0.3  # conductivity substrate, Kcal m / (m2 h deg C)
d_x.D2 <- 0.01  # heat thickness substrate, m

cloud <- c(0, 0.3, 0.7, 1, 1)  # cloud amount, tenths
ctype <- c(0, 0.75, 0.75, 0.75, 0.2)  # cloud type code, -
ctype2 <- c(0, 0.91, 0.91, 0.91, 0.11)  # cloud type code, -
tskin <- seq(30, 36, 0.5)
W <- 0.06

for (i in 1:length(cloud)) {

    n.N2 <- cloud[i]
    k.C2 <- ctype[i]
    kk.C3 <- ctype2[i]
    out <- lapply(1:length(tskin), function(x) {
        MANMO_R(SI.mode = 0, Tskin1.T = tskin[x], W = W, Ht.H4 = Ht.H4,
            Wt.W4 = Wt.W4, a_skn.B4 = a_skn.B4, e_skin.Y4 = e_skin.Y4,
            K6 = K6, K7 = K7, O2 = O2, V3 = V3, G_m.G2 = G_m.G2,
            K8 = K8, a_clo.B5 = a_clo.B5, e_clo.Y5 = e_clo.Y5,
            K3 = K3, D3 = D3, CLO.C4 = CLO.C4, month.Z2 = month.Z2,
            day.Z3 = day.Z3, time.Z4 = time.Z4, lat.Z5 = lat.Z5,
            lon.Z6 = lon.Z6, TZone.Z7 = TZone.Z7, trans.R2 = trans.R2,
            a.B3 = a.B3, e_sfc.Y3 = e_sfc.Y3, k.C2 = k.C2, kk.C3 = kk.C3,
            n.N2 = n.N2, Q_h.Q2 = Q_h.Q2, q_h.Q7 = q_h.Q7, T_a.T0 = T_a.T0,
            T_sky.T2 = T_sky.T2, T_gnd.T7 = T_gnd.T7, T_clo.T9 = tskin[x],
```

```
            T_m.S2 = T_m.S2, RH.H2 = RH.H2, ff.V2 = ff.V2, dd.V5 = dd.V5,
            k_x.K2 = k_x.K2, d_x.D2 = d_x.D2, CLO.mode = CLO.mode,
            mode = mode, iterate = 0)
    })
    out <- do.call("rbind", lapply(out, data.frame))

    if (i == 1) {
        plot(out$Tskin, out$I_m.I, type = "l", ylim = c(-200,
            800), ylab = "LONGWAVE RADIATION, Im, (KCAL / H)",
            xlab = "T_a - T_clo (deg C)")
    } else {
        points(out$Tskin, out$I_m.I, type = "l")
    }
}
```

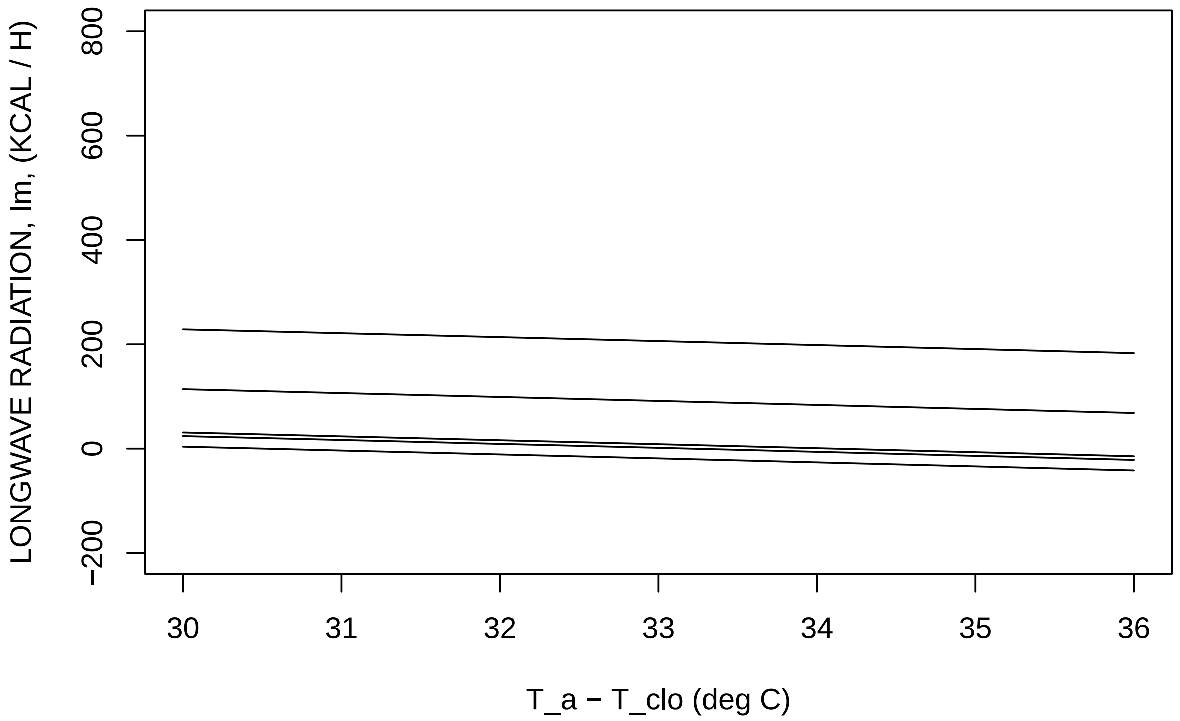


```
mode <- 1  # 1 climatological, 2 standard inputs
CLO.mode <- 1  # use empirical clothes temp function (0) or fix clothing temp (1)
cloud <- seq(0, 1, 0.1)  # cloud amount, tenths
k.C2 <- 0.75  # cloud type code, -
kk.C3 <- 0.91  # cloud type code, -
T_a.T0 <- 36.1  # air temp, deg C
ff.V2 <- 0.5  # wind speed, m/s

out <- lapply(1:length(cloud), function(x) {
```

```
    MANMO_R(SI.mode = 0, W = W, Ht.H4 = Ht.H4, Wt.W4 = Wt.W4,
        a_skn.B4 = a_skn.B4, e_skin.Y4 = e_skin.Y4, K6 = K6,
        K7 = K7, O2 = O2, V3 = V3, G_m.G2 = G_m.G2, K8 = K8,
        a_clo.B5 = a_clo.B5, e_clo.Y5 = e_clo.Y5, K3 = K3, D3 = D3,
        CLO.C4 = CLO.C4, month.Z2 = month.Z2, day.Z3 = day.Z3,
        time.Z4 = time.Z4, lat.Z5 = lat.Z5, lon.Z6 = lon.Z6,
        TZone.Z7 = TZone.Z7, trans.R2 = trans.R2, a.B3 = a.B3,
        e_sfc.Y3 = e_sfc.Y3, k.C2 = k.C2, kk.C3 = kk.C3, n.N2 = cloud[x],
        Q_h.Q2 = Q_h.Q2, q_h.Q7 = q_h.Q7, T_a.T0 = T_a.T0, T_sky.T2 = T_sky.T2,
        T_gnd.T7 = T_gnd.T7, T_clo.T9 = T_clo.T9, T_m.S2 = T_m.S2,
        RH.H2 = RH.H2, ff.V2 = ff.V2, dd.V5 = dd.V5, k_x.K2 = k_x.K2,
        d_x.D2 = d_x.D2, CLO.mode = CLO.mode, mode = mode, iterate = 0)
})
out <- do.call("rbind", lapply(out, data.frame))

plot(cloud, out$I_e.E1, type = "b", ylim = c(500, 800), ylab = "LONGWAVE RADIATION, Ie, (KCAL / H)",
    xlab = "CLOUDINESS")
```

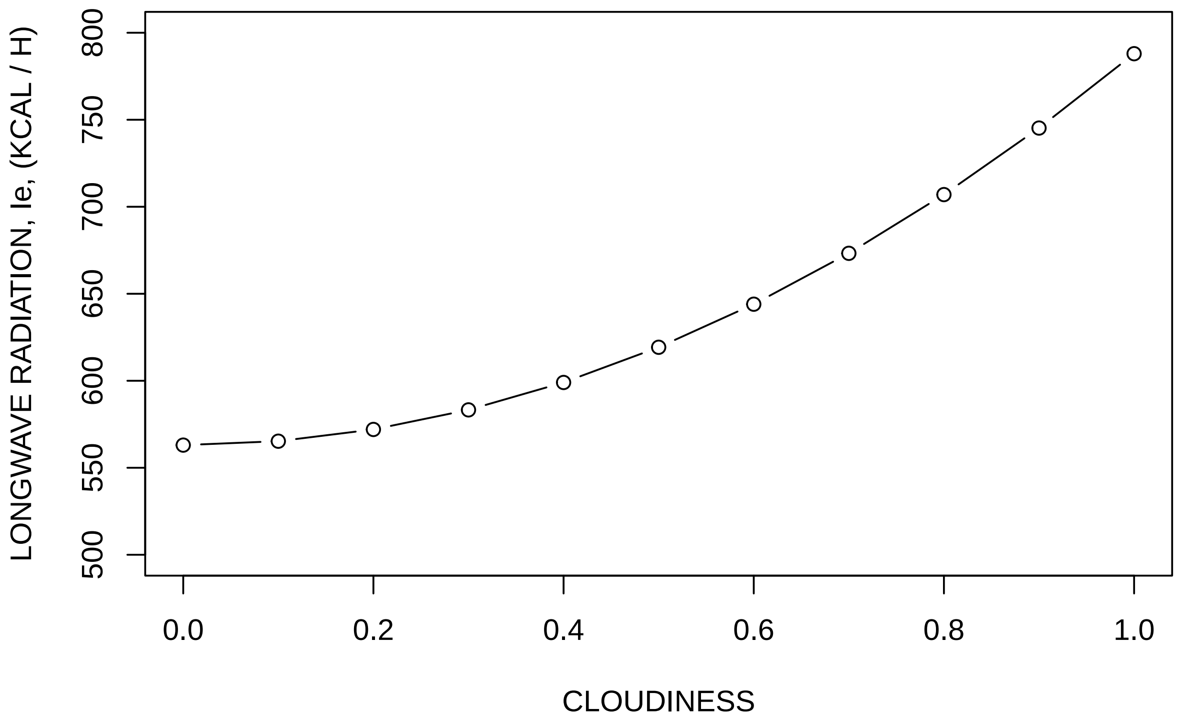

References

Morgan, D. L., Pruitt, W. O., & Lourence, F. J. (1971). Estimation of atmospheric radiation. Journal of Applied Meteorology, 10, 463-468.

Myrup, L. O., & Morgan, D. L. (1972). Numerical model of the urban atmosphere. Volume I The city-surface

interface. University of California, Davis.
