# Supplementary material for: HomoTherm: An Open‐Source Approach to Modelling Heat Exchange in Humans and Other Hominins in Diverse Environments
Source: Glob Chang Biol. 2026 Apr 1;32(4):e70830. doi: 10.1111/gcb.70830 (PMC13044332; doi:10.1111/gcb.70830)
Supplement: Supplementary file 10 — Appendix S10: gcb70830‐sup‐0010‐Appendix 10.pdf. [file GCB-32-e70830-s006.pdf]

```
#' MANMO human heat budget model
#'
#' An R implementation of the MANMO model of Myrup and Morgan (1972).
#' Can handle vector or raster input for environmental variables.
#'
#' @encoding UTF-8
#' @param mode = 1, 1 climatological, 2 standard inputs
#' @param iterate = 1, use bisection method to solve for Tskin? (1) or just report consequences
for current Tskin value (0)
#' @param SI.mode = 1, using SI units for inputs (metabolic rate, flesh conductivity, solar
radiation) and outputs (1) or c.g.s units (0)
#' @param CLO.mode = 0, use empirical clothes temp function (0), fixed clothing temp (1) or
equal to Tskin (2)
#' @param Tskin1.T = 2, skin temperature for calculations (lower bound initial guess for
bisection method), °C
#' @param Tskin2.S = 64, skin temperature (upper bound initial guess for bisection method), °C
#' @param W = 0.02, proportion of skin that is wet, -
#' @param Ht.H4 = 180, height, cm
#' @param Wt.W4 = 64, weight, kg
#' @param a_skn.B4 = 0.30, albedo of skin, -
#' @param e_skin.Y4 = 0.98, emissivity of skin, -
#' @param Maximum.SR = 9 * 0.85, maximum possible sweat rate, g / (min m2)
#' @param K6 = 0.78, radiation area coefficient, -
#' @param K7 = 0.1e-10, skin contact area coefficient, -
#' @param O2 = 0.1, orientation of person, degrees
#' @param V3 = 1.5, movement of person, m/s
#' @param G_m.G2 = 130, activity (metabolic costs), Kcal / (h m2)
#' @param K8 = 0.023, clothes contact area coefficient, -
#' @param a_clo.B5 = 0.3, albedo of clothes, -
#' @param e_clo.Y5 = 0.95, emissivity of clothes, -
#' @param K3 = c(0.014, 0.01, 0.072, 1), conductivity of clothes, 4 layers, Kcal m / (m2 h °C)
#' @param D3 = c(0.001, 0.001, 0.001, 0.1e-10), thickness of clothing, 4 layers, m
#' @param CLO.C4 = 0.6, clothing units, -
#' @param T_clo.T9 = 36, clothing temperature, °C
#' @param month.Z2 = 7, month, -
#' @param day.Z3 = 21, day, -
#' @param time.Z4 = 13, time, LST
#' @param lat.Z5 = 38.54, latitude, degrees
#' @param lon.Z6 = 121.78, longitude, degrees
#' @param TZone.Z7 = 8, time zone, -
#' @param trans.R2 = 0.6, transmissivity coefficient, -
#' @param a.B3 = .2, albedo of surface, -
#' @param e_sfc.Y3 = 1, emissivity of surface, -
#' @param e_air = 1, emissivity of air, -
#' @param Q_h.Q2 = 1.08, global solar radiation, ly/min = Cal/cm2/min, multiply W / m2 by
4.183995 * 60 / 10000 to convert to ly / min
#' @param q_h.Q7 = 0.133, diffuse solar radiation, ly/min = Cal/cm2/min, multiply W / m2 by
4.183995 * 60 / 10000 to convert to ly / min
#' @param z.K0 = 0, solar zenith angle, degrees
#' @param k.C2 = 0, cloud type code solar, -
#' @param kk.C3 = 0, cloud type code infrared, -
#' @param n.N2 = 0, cloud amount, tenths
#' @param T_a.T0 = 36.2, air temp, °C
#' @param T_sky.T2 = T_a.T0, sky temp, °C
#' @param T_gnd.T7 = T_a.T0, ground temp, °C
#' @param T_m.S2 = T_a.T0, substrate temp, °C
#' @param RH.H2 = 0.28, relative humidity, -
#' @param ff.V2 = 5, wind speed, m/s
#' @param dd.V5 = 180, wind direction, degrees
#' @param k_x.K2 = 0.3, conductivity substrate, Kcal m / (m2 h °C)
#' @param d_x.D2 = 0.01, heat thickness substrate, m
#' @param P = 101325, air pressure, Pa
MANMO_R <- function(Tskin1.T = 2,
                    Tskin2.S = 64,
                    W = 0.02,
                    sex.Y7 = 1,
                    Ht.H4 = 180,
                    Wt.W4 = 64,
                    a_skn.B4 = 0.30,
```

```
                 e_skin.Y4 = 0.98,
                 K6 = 0.78,
                 K7 = 1e-10,
                 O2 = 0.1,
                 V3 = 1.5,
                 G_m.G2 = 130,
                 K8 = 0.023,
                 a_clo.B5 = 0.3,
                 e_clo.Y5 = 0.95,
                 K3 = c(0.014, 0.01, 0.072, 1),
                 D3 = c(0.001, 0.001, 0.001, 1e-10),
                 CLO.C4 = 0.6,
                 month.Z2 = 8,
                 day.Z3 = 4,
                 time.Z4 = 10.25,
                 lat.Z5 = 38.54,
                 lon.Z6 = 121.78,
                 TZone.Z7 = 8,
                 trans.R2 = 0.6,
                 a.B3 = 0.12,
                 e_sfc.Y3 = 1,
                 Q_h.Q2 = 1.08,
                 q_h.Q7 = 0.133,
                 T_a.T0 = 22.3,
                 T_sky.T2 = 2,
                 T_gnd.T7 = 36,
                 T_clo.T9 = 36,
                 T_m.S2 = 36,
                 RH.H2 = 0.73,
                 ff.V2 = 1,
                 dd.V5 = 218.5,
                 k_x.K2 = 0.5,
                 d_x.D2 = 0.01,
                 CLO.mode = 1,
                 k.C2 = 0,
                 kk.C3 = 0,
                 n.N2 = 0,
                 mode = 2,
                 e_air = 1,
                 T_clo.offset = 0.7,
                 Maximum.SR = 9 * 0.85,
                 iterate = 1,
                 SI.mode = 1,
                 z.K0 = 0,
                 P = 101325){


# title inputs
# C9 <- 1
# R9 <- 1
# month.Z2 <- 2 # month
# day.Z3 <- 2 # day

# physiological inputs
# Ht.H4 <- 180 # height, cm
# Wt.W4 <- 64 # weight, kg
# a_skn.B4 <- .30 # albedo of skin, -
# e_skin.Y4 <- .98 # emissivity of skin, -
# K6 <- .78 # radiation area coefficient, -
# K7 <- 1e-10 # skin contact area coefficient, -
# O2 <- .1 # orientation of person, degrees
# V3 <- 1.5 # movement of person, m/s
# G_m.G2 <- 130 # activity (metabolic costs), Kcal / (h m2)
# K8 <- .023 # clothes contact area coefficient, -
# a_clo.B5 <- .3 # albedo of clothes, -
# e_clo.Y5 <- .95 # emissivity of clothes, -
# K3[1] <- .014 # conductivity of clothes, 1st layer, Kcal m / (m2 h C)
# K3[2] <- .01 # conductivity of clothes, 2nd layer, Kcal m / (m2 h C)
# K3[3] <- .072 # conductivity of clothes, 3rd layer, Kcal m / (m2 h C)
```

```
  # K3[4] <- 1 # conductivity of clothes, 4th layer, Kcal m / (m2 h C)
  # D3[1] <- .001 # thickness of clothing, 1st layer, m
  # D3[2] <- .001 # thickness of clothing, 2nd layer, m
  # D3[3] <- .001 # thickness of clothing, 3rd layer, m
  # D3[4] <- 1e-10 # thickness of clothing, 4th layer, m
  # CLO.C4 <- .6 # clothing units, -

  # environmental inputs
  # month.Z2 <- 8 # month, -
  # day.Z3 <- 4 # day, -
  # time.Z4 <- 10.25 # time, LST
  # lat.Z5 <- 38.54 # latitude, degrees
  # lon.Z6 <- 121.78 # longitude, degrees
  # TZone.Z7 <- 8 # time zone, -
  # trans.R2 <- .6 # transmissivity coefficient, -
  # a.B3 <- .12 # doesn't work fo original value .22 # albedo of surface, -
  # e_sfc.Y3 <- 1 # emissivity of surface, -
  # Q_h.Q2 <- 1.08 # global (direct + diffuse) radiation, ly / min = Cal/cm2/min = 4.1868 J /
cm2 / min = 697.8 W / m2
  # q_h.Q7 <- .133 # diffuse radiation, ly / min
  # k.C2 <- 0 # cloud type code, solar, -
  # kk.C3 <- 0 # cloud type code, IR, -
  # n.N2 <- 0 # cloud amount, tenths
  # T_a.T0 <- 22.3 # air temp, °C
  # T_sky.T2 <- 2 # sky temp, °C
  # T_gnd.T7 <- 36 # ground temp, °C
  # T_clo.T9 <- 36 # clothing temp, °C
  # T_m.S2 <- 36 # substrate temp, °C
  # RH.H2 <- .73 # relative humidity, -
  # ff.V2 <- 1 # wind speed, m/s
  # dd.V5 <- 218.5 # can't get correct V.V4 with 250 # wind direction, degrees
  # k_x.K2 <- .5 # conductivity substrate, Kcal m / (m2 h °C)
  # d_x.D2 <- .01 # heat thickness substrate, m
  if(max(RH.H2) > 1){
    message("error: humidity (RH.H2) > 1 - make sure it's fractional and not % \n")
    break
  }
  T_a.T0[T_a.T0 == 0] <- 0.00001
  if(class(Tskin1.T) != "RasterLayer"){ # check this logic - problem if T_a == 0)
    Tskin1.T <- (T_a.T0 / T_a.T0) * Tskin1.T
  }
  Tskin2.S <- (T_a.T0 / T_a.T0) * Tskin2.S
  W <- (T_a.T0 / T_a.T0) * W

  # SI to original units
  if(SI.mode == 1){
    G_m.G2 <- G_m.G2 / 4186 * 3600 # W / m2 to Kcal / (h m2)
    K3 <- K3 / 4186 * 3600 # W m / (m2 C) to Kcal m / (m2 h C)
    Q_h.Q2 <- Q_h.Q2 / 4.183995 * 60 / 10000 # W / m2 to ly / min = Cal/cm2/min
    q_h.Q7 <- q_h.Q7 / 4.183995 * 60 / 10000 # W / m2 to ly / min = Cal/cm2/min
  }

  D0 <- vector(length = 12)

  FNT <- function(Z){
    (Z + 273.15) ^ 4
  }

  O3 <- O2 * 0.01745 #(pi/180)
  V6 <- dd.V5 * 0.01745 #(pi/180)

  # calculation of areas

  A.A2 <- 0.00718 * Wt.W4 ^ 0.425 * Ht.H4 ^ 0.725 # DuBois area, m2
  A_r.A6 <- K6 * A.A2 # radiation area, m2
  PT_clo.A9 <- -0.0239 + 1.794 * CLO.C4 - 1.101 * CLO.C4 ^ 2 + .225 * CLO.C4 ^3 # percent area
clothed, %
  if(CLO.C4 == 0 | PT_clo.A9 < 0){
    PT_clo.A9 <- 0
```

```
  }
  PA_clo.A4 <- PT_clo.A9 * A.A2 # area of clothes, m2
  PA_unc.A5 <- A.A2 - PA_clo.A4 # area of skin, m2

  # metabolic heat flux term
  M_m.M <- G_m.G2 * A.A2 # metabolic heat, Kcal / h

  # calculate h_c.H3
  X2 <- ff.V2 * cos(V6)
  X3 <- ff.V2 * sin(V6)
  X4 <- V3 * cos(O3)
  X5 <- V3 * sin(O3)
  V.V4 <- ((X2 + X4) * (X2 + X4) + (X3 + X5) * (X3 + X5)) ^ (1 / 2)

  h_c.H3 <- V.V4 * 0
  h_c.H3[V.V4 < 0.5] <- 2.3 + 5.6 * V.V4 ^ 0.67 * (P / 101325) ^ 0.67
  h_c.H3[V.V4 >= 0.5 & V.V4 < 2] <- 6.56 * V.V4 ^ 0.618 * (P / 101325) ^ 0.618
  h_c.H3[V.V4 >= 2 & V.V4 < 4] <- 5.83 * V.V4 ^ 0.805 * (P / 101325) ^ 0.805
  h_c.H3[V.V4 >= 4] <- 5.38 * V.V4 ^ 0.9 * (P / 101325) ^ 0.9

  # solar radiation

  D0[1] <- 0
  D0[2] <- 31
  D0[3] <- 59
  D0[4] <- 90
  D0[5] <- 120
  D0[6] <- 151
  D0[7] <- 181
  D0[8] <- 212
  D0[9] <- 243
  D0[10] <- 273
  D0[11] <- 304
  D0[12] <- 334
  M0 <- day.Z3 + D0[month.Z2]
  G0 <- 0.4093 * cos(0.0172 * (M0 - 172))
  J0 <- time.Z4 - lon.Z6 / 15 + TZone.Z7
  C0 <- cos(G0) * cos(0.01745 * lat.Z5) * cos(0.26175 * J0 + 3.1416) #(pi/180)
  C0 <- C0 + sin(G0) * sin(0.01745 * lat.Z5) #(pi/180)
  C0[C0 < 0] <- 1e-11
  S0 <- (1 - C0 ^ 2) ^ (1/2)
  H0 <- atan(S0 / C0)
  if(class(z.K0) != "RasterLayer"){
    z.K0 <- H0 * 57.3
  }
  I0 <- 1 / cos(H0)
  #S_h.A3 <- A.A2 * 4.07464 * exp(-.051 * (90 - z.K0)) # shadow area from Terjung and Louie
1971 (wrong? too large)
  theta <- (90 - z.K0) * pi / 180 # solar altitude
  theta2 <- 0 # solar azimuth (facing sun)
  S_h.A3 <- (0.043 * sin(theta) + 2.997 * cos(theta) * (0.02133 * cos(theta2) ^ 2 + 0.0091 *
sin(theta2)^2) ^ 0.5) / 1.81 * A.A2 # Underwood and Ward formula
  if(mode == 1){
    M0 <- M0 * .01
    V0 <- .9836 - 8.91e-5 * M0 + 4.84e-4 * M0 ^ 2
    V0 <- V0 - 2.357e-5 * M0 ^ 3 + 2.6e-5 * M0 ^ 4 + 1.1e-6 * M0 ^ 5
    U0 <- 1.952 * V0 ^ (-2) * C0
  }else{
    U0 <- NA
  }

  # theoretical-climatological radiation term

  if(mode == 1){
    Q8 <- 1.05 * 600 * U0 * trans.R2 ^ I0 * (1 - (1 - k.C2) * n.N2)
    Q_m.Q1 <- Q8 * S_h.A3
    Q0 <- (0.053 + 0.048 * I0) * U0
    D8 <- 0.5 * 600 * Q0 * (1 + (1 - k.C2) * n.N2)
    q_v.D1 <- D8 * A.A2
```

```
    Qq_r.G8 <- (D8 + .5 * Q8) * a.B3
  }else{
    Q0 <- NA
  }

  # standard measurements case radiation term

  if(mode == 2){
    Q8 <- 1.05 * 600 * (Q_h.Q2 - q_h.Q7) # total - diffuse, Kcal / (m2 h), 1.05 is because of arm movement
    Q_m.Q1 <- Q8 * S_h.A3 # direct solar by silhouette area
    D8 <- 0.5 * 600 * q_h.Q7 # diffuse solar, Kcal / (m2 h)
    q_v.D1 <- D8 * A_r.A6 # diffuse solar by total surface area, A.A2 should probably change to A_r.A6
    Qq_r.G8 <- 0.5 * 600 * Q_h.Q2 * a.B3 # reflected solar from ground, Kcal / (m2 h)
  }

  q_g.G1 <- Qq_r.G8 * A_r.A6 # A.A2 should probably change to A_r.A6
  R8 <- Q_m.Q1 + q_v.D1 + q_g.G1
  R_munc.R1 <- R8 * (1 - a_skn.B4) * (1 - PT_clo.A9) # adjust direct for area unclothed and albedo of skin
  R_clo.I8 <- R8 * (1 - a_clo.B5) * PT_clo.A9 # adjust for area clothed and albedo of clothes
  H_clo.H8 <- h_c.H3 * (T_clo.T9 - T_a.T0) * 1.08 * A.A2 * PT_clo.A9 # heat from total radiation conducted through clothes
  if(R_clo.I8 > 0){
    R_mclo.M1 <- R_clo.I8 - H_clo.H8 # solar radiant heat absorbed through clothes (as in code - incorrect?) - not sure how this relates to I_clo.C8 i.e. longwave from clothes
  }else{
    R_mclo.M1 <- 0
  }
  R_mclo.M1[R_mclo.M1 < 0] <- 0
  R_m.R <- R_munc.R1 + R_mclo.M1


  # longwave radiation on man
  if(CLO.mode == 0){
    T_clo.T9 <- T_clo.offset * T_a.T0 - 21.9 + Tskin1.T # clothing temperature, °C
    T_clo.T9[Q8 < 0.001] <- T_a.T0
  }

  if(CLO.mode == 2){ # also checking if no solar radiation
    T_clo.T9 <- Tskin1.T
    T_clo.T9[Q8 < 0.001] <- T_a.T0
  }
  I_clo.C8 <- 4.879e-8 * e_clo.Y5 * FNT(T_clo.T9) * PT_clo.A9 * A_r.A6 # I_clo, Kcal / h
  I_unc.S8 <- 4.879e-8 * e_skin.Y4 * FNT(Tskin1.T) * (1 - PT_clo.A9) * A_r.A6 # I_unc, Kcal / h
  I.I1 <- I_clo.C8 + I_unc.S8
  E_a.E2 <- 1.372 + 0.851 * T_a.T0 - 0.0161 * T_a.T0 ^ 2 + 0.00071 * T_a.T0 ^ 3 # saturation vapour pressure of air, replace with wetair
  #E_a.E2 <- WETAIR(db = T_a.T0, bp = P)$esat / 133.332
  if(mode == 1){ # equation 24 in Murup and Morgan, not in BASIC code!
    T8 <- T_a.T0
    I_e.E1 <- (4.879e-8 * e_sfc.Y3 * FNT(T8) + (4.879e-8 * e_air * FNT(T_a.T0) * (0.66 + 0.039 * (E_a.E2 / 6 * 1.3332239) ^ (1 / 2))) * (1 + kk.C3 * n.N2 ^ 2)) * A_r.A6 / 2 # note term with E_a.E2 is divided by 6 to convert from ly (cal / m / cm 2) to KCal / m2, and 1.33 term is to convert E_a.E2 itself form mmHg to mbar, as in Morgan, D. L., Pruitt, W. O., & Lourence, F. J. (1971). Estimation of atmospheric radiation. Journal of Applied Meteorology, 10, 463-468.
  }
  if(mode == 2){
    T8 <- (T_sky.T2 + T_gnd.T7) * 0.5
    I_e.E1 <- 4.879e-8 * e_sfc.Y3 * A_r.A6 * FNT(T8)
  }
  I_m.I <- I_e.E1 - I.I1
  W0 <- (0.66 + 0.039 * (E_a.E2 * 0.743 * RH.H2) ^ (1 / 2)) # does not appear to be used!

  # evaporative heat flux term

  E_s.E3 <- 1.91 * Tskin1.T - 25.33 # saturation vapor pressure at Tskin, mmHg, replace with wetair
```

```
  E_r.L1 <- G_m.G2 * 0.0023 * (RH.H2 * E_a.E2 - 44) * A.A2 # respiratory evaporative heat
exchange (manuscript equation has 44 - RH.H2 * E_a.E2)
  #E_r.L1 <- G_m.G2 * 0.0023 * (44 - RH.H2 * E_a.E2) * A.A2 # respiratory evaporative heat
exchange (manuscript equation has 44 - RH.H2 * E_a.E2)
  if(iterate){
    W[Tskin1.T > 33] <- 0.25 * Tskin1.T[Tskin1.T > 33] - 8.25
    W[Tskin1.T > 37] <- 1
  }
  f_pd.F2 <- 0 # permeation efficiency through clothes

  V8 <- V.V4 ^ 2
  V9 <- V.V4 ^ 3
  if(CLO.C4 > 0){
    if(CLO.C4 <= 0.1){
      f_pd.F2 <- 0.971 - 0.059 * V.V4 + 0.00251 * V8 # permeation efficiency through clothes
    }else{
      if(CLO.C4 <= 0.3){
        f_pd.F2 <- 0.915 - 0.17 * V.V4 + 0.0238 * V8 - 0.0012 * V9 # permeation efficiency
through clothes
      }else{
        if(CLO.C4 <= 0.6){
          f_pd.F2 <- 0.858 - 0.246 * V.V4 + 0.0372 * V8 - 0.0019 * V9 # permeation efficiency
through clothes
        }else{
          if(CLO.C4 <= 0.8){
            f_pd.F2 <- 0.833 - 0.298 * V.V4 + 0.0537 * V8 - 0.003 * V9 # permeation efficiency
through clothes
          }else{
            if(CLO.C4 <= 1){
              f_pd.F2 <- 0.784 - 0.296 * V.V4 + 0.0499 * V8 - 0.00269 * V9 # permeation
efficiency through clothes
            }else{
              if(CLO.C4 <= 1.5){
                f_pd.F2 <- 0.727 - 0.34 * V.V4 + 0.0652 * V8 - 0.00377 * V9 # permeation
efficiency through clothes
              }else{
                #if(CLO.C4 <= 2){
                f_pd.F2 <- 0.62 - 0.301 * V.V4 + 0.054 * V8 - 0.00296 * V9 # permeation
efficiency through clothes
              }}}}}}
  #X0 <- 2.2 * h_c.H3 * (0.06 + 0.94 * W) * (RH.H2 * E_a.E2 - E_s.E3) # 2.2 is based at sea
level - 'Lewis relation', h_e = kappa * h_c
  X0 <- 2.2 * h_c.H3 * W * (RH.H2 * E_a.E2 - E_s.E3) # 2.2 is based at sea level - 'Lewis
relation', h_e = kappa * h_c
  E_clo.C1 <- X0 * f_pd.F2 * PA_clo.A4 # multiply through by permeation efficiency and area
clothed
  E_unc.U1 <- X0 * PA_unc.A5 # multiply through by area of skin
  E_m.E <- E_r.L1 + E_clo.C1 + E_unc.U1

  if(iterate == 0){
    maxrate <- Maximum.SR * A.A2 * 60 * 2264.76 / 4.183995 / 1000 * -1 # max rate g / m2 x area
m2 x 60 m / h * 2264.76 J/g / 4.184 J / Cal / 1000 Cal / KCal
    predrate <- E_clo.C1 + E_unc.U1
    test <- predrate
    predrate[predrate < maxrate] <- maxrate
    E_m.E <- E_r.L1 + predrate # g / min to g / h to kJ / h to kCal / h  # cap at maximum sweat
rate
  }
  # convective heat flux term
  H_r.P1 <- 0.001173 * G_m.G2 * (T_a.T0 - 37) * A.A2
  f_cl.F3 <- 0 # convective efficiency factor for clothing

  if(CLO.C4 > 0){
    if(CLO.C4 <= 0.1){
      f_cl.F3 <- 0.879 - 0.0542 * V.V4 + 0.00284 * V8 # convective efficiency factor for
clothing
    }else{
      if(CLO.C4 <= 0.3){
        f_cl.F3 <- 0.727 - 0.0823 * V.V4 + 0.00469 * V8 # convective efficiency factor for
```

```
clothing
      }else{
        if(CLO.C4 <= 0.6){
          f_cl.F3 <- 0.577 - 0.0886 * V.V4 + 0.00516 * V8 # convective efficiency factor for
clothing
        }else{
          if(CLO.C4 <= 0.8){
            f_cl.F3 <- 0.52 - 0.129 * V.V4 + 0.0182 * V8 - 0.00088 * V9 # convective efficiency
factor for clothing
          }else{
            if(CLO.C4 <= 1){
              f_cl.F3 <- 0.452 - 0.114 * V.V4 + 0.0165 * V8 - 0.00085 * V9 # convective
efficiency factor for clothing
            }
            if(CLO.C4 <= 1.5){
              f_cl.F3 <- 0.39 - 0.127 * V.V4 + 0.021 * V8 - 0.0011 * V9 # convective efficiency
factor for clothing
            }else{
              #if(CLO.C4 == 2){
              f_cl.F3 <- 0.2725 - 0.0877 * V.V4 + 0.0147 * V8 - 0.00082 * V9 # convective
efficiency factor for clothing
            }}}}}}
  H_unc.K1 <- h_c.H3 * (T_a.T0 - Tskin1.T) * PA_unc.A5
  H_clo.S1 <- h_c.H3 * (T_a.T0 - Tskin1.T) * f_cl.F3 * PA_clo.A4 * 1.08
  H_m.H <- H_r.P1 + H_unc.K1 + H_clo.S1

  # conductive heat flux term

  A_kunc.A7 <- K7 * A.A2
  A_clo.A8 <- K8 * A.A2
  Y0 <- D3[1] / K3[1] + D3[2] / K3[2] + D3[3] / K3[3] + D3[4] / K3[4]
  D_unc.A1 <- k_x.K2 * (T_m.S2 - Tskin1.T) * A_kunc.A7 / d_x.D2
  D_clo.B1 <- (T_m.S2 - Tskin1.T) * A_clo.A8 / Y0
  D_m.D <- D_unc.A1 + D_clo.B1
  X <- M_m.M + R_m.R + I_m.I + E_m.E + H_m.H + D_m.D

  Tskin <- Tskin1.T
  Y <- X
  N <- 0

  if(iterate == 1){

    # bisection method solution to equation

    while(abs(Tskin1.T - Tskin2.S ) > 0.01 & N < 100){
      T1 <- (Tskin1.T + Tskin2.S ) * 0.5
      if(T1 > 33){
        W <- 0.25 * T1 - 8.25
      }
      if(T1 > 37){
        W <- 1
      }
      N <- N + 1
      if(CLO.mode == 0){
        T_clo.T9 <- T_clo.offset * T_a.T0 - 21.9 + T1 # clothing temperature, °C
        I_clo.C8 <- 4.879e-8 * e_clo.Y5 * FNT(T_clo.T9) * PT_clo.A9 * A_r.A6 # I_clo, Kcal /
(m2 h)
      }
      if(CLO.mode == 2){
        T_clo.T9 <- T1
        I_clo.C8 <- 4.879e-8 * e_clo.Y5 * FNT(T_clo.T9) * PT_clo.A9 * A_r.A6 # I_clo, Kcal /
(m2 h)
      }
      I_unc.S8 <- 4.879e-8 * e_skin.Y4 * FNT(T1) * (1 - PT_clo.A9) * A_r.A6 # I_unc, Kcal / (m2
h)
      I.I1 <- I_clo.C8 + I_unc.S8
      E_s.E3 <- 1.91 * T1 - 25.33 # saturation vapor pressure at Tskin, mmHg
      #X0 <- 2.2 * h_c.H3 * (0.06 + 0.94 * W) * (RH.H2 * E_a.E2 - E_s.E3) # 2.2 = kappa = Lewis
relation (deg C mmHg-1) at sea level
```

```
      X0 <- 2.2 * h_c.H3 * W * (RH.H2 * E_a.E2 - E_s.E3) # 2.2 = kappa = Lewis relation (deg C mmHg-1) at sea level
      E_clo.C1 <- X0 * f_pd.F2  * PA_clo.A4 # error in original code - had A.A2 instead of PA_clo.A4 - can't get results from their table unless this error is inserted above and code run to just before bisection
      E_unc.U1 <- X0 * PA_unc.A5
      H_unc.K1 <- h_c.H3 * (T_a.T0 - T1) * PA_unc.A5
      H_clo.S1 <- h_c.H3 * (T_a.T0 - T1) * f_cl.F3 * PA_clo.A4 * 1.08
      I_m.I <- I_e.E1 - I.I1
      E_m.E <- E_r.L1 + E_unc.U1 + E_clo.C1
      H_m.H <- H_r.P1 + H_unc.K1 + H_clo.S1
      D_unc.A1 <- k_x.K2 * (T_m.S2 - T1) * A_kunc.A7 / d_x.D2
      D_clo.B1 <- (T_m.S2 - T1) * A_clo.A8 / Y0
      D_m.D <- D_unc.A1 + D_clo.B1
      H_clo.H8 <- h_c.H3 * (T_clo.T9 - T_a.T0) * 1.08 * A.A2 * PT_clo.A9 # added here - not in original code, but this gets updated in the while loop if in CLO.mode 0 or 2
      if(R_clo.I8 > 0){
        R_mclo.M1 <- R_clo.I8 - H_clo.H8 # solar radiant heat absorbed through clothes (as in code - incorrect?) - not sure how this relates to I_clo.C8 i.e. longwave from clothes
      }else{
        R_mclo.M1 <- 0
      }
      R_mclo.M1[R_mclo.M1 < 0] <- 0
      R_m.R <- R_munc.R1 + R_mclo.M1
      Y <- M_m.M + R_m.R + I_m.I + E_m.E + H_m.H + D_m.D
      Z <- Y * X
      Tskin2.S[Z < 0] <- T1
      Tskin1.T[Z >= 0] <- T1
      X[Z >= 0] <- Y
    }

    Tskin <- T1

  } # end check for iterate == 0

  r <- 1 - W ^ 2 / 2 # Parsons p. 43, sweating efficiency
  evap.L.h <- E_m.E * 4.183995 * 1000 / 2264.76 * -1 / 1000 # convert Kcal / h to  kJ / h to J / h to g H2O / h to L / h
  sweat.L.h <- (1 / r) * max(0, (E_clo.C1 + E_unc.U1) * 4.183995 * 1000 / 2264.76 * -1  / 1000) # convert Kcal / h to  kJ / h to J / h to g H2O / h to L / h
  output <- cbind(Tskin, W, T_a.T0, T_gnd.T7, T_sky.T2, T_m.S2, ff.V2, RH.H2, Q_h.Q2, q_h.Q7, N, M_m.M, R_m.R, I_m.I, E_m.E, H_m.H, D_m.D, R_munc.R1, Q_m.Q1, q_v.D1, q_g.G1, R_mclo.M1, I_e.E1, I.I1, E_r.L1, E_clo.C1, E_unc.U1, H_r.P1, H_unc.K1, H_clo.S1, D_unc.A1, D_clo.B1,
                  R8, Q8, D8, Qq_r.G8, R_clo.I8, H_clo.H8, I_clo.C8, I_unc.S8, A.A2, S_h.A3, PA_clo.A4, PA_unc.A5, A_r.A6, A_kunc.A7, A_clo.A8, PT_clo.A9,
                  E_a.E2, E_s.E3, W0, f_pd.F2, f_cl.F3, V.V4, h_c.H3, T_clo.T9, T8, Y0, z.K0, U0, Q0, Y, sweat.L.h, evap.L.h)
  names(output) <- c("Tskin", "W", "T_a.T0", "T_gnd.T7", "T_sky.T2", "T_m.S2", "ff.V2", "RH.H2", "Q_h.Q2", "q_h.Q7", "N", "M_m.M", "R_m.R", "I_m.I", "E_m.E", "H_m.H", "D_m.D", "R_munc.R1", "Q_m.Q1", "q_v.D1", "q_g.G1", "R_mclo.M1", "I_e.E1", "I.I1", "E_r.L1", "E_clo.C1", "E_unc.U1", "H_r.P1", "H_unc.K1", "H_clo.S1", "D_unc.A1", "D_clo.B1", "R8", "Q8", "D8", "Qq_r.G8", "R_clo.I8", "H_clo.H8", "I_clo.C8", "I_unc.S8", "A.A2", "S_h.A3", "PA_clo.A4", "PA_unc.A5", "A_r.A6", "A_kunc.A7", "A_clo.A8", "PT_clo.A9", "E_a.E2", "E_s.E3", "W0", "f_pd.F2", "f_cl.F3", "V.V4", "h_c.H3", "T_clo.T9", "T8", "Y0", "z.K0", "U0", "Q0", "Y", "sweat.L.h", "evap.L.h")
  if(SI.mode == 1){
    output[9:10] <- output[9:10] * 4186 / 3600 # Kcal / h / m2 to W / m2
    output[12:40] <- output[12:40] * 4186 / 3600 # Kcal / h to W
    output[49:50] <- output[49:50] * 4186 # mmHG to KPa
    output[55] <- output[55] * 4186 / 3600 # Kcal / (h m2 °C) to W / (m2 °C)
    output[58] <- output[58] * 4186 / 3600 # Kcal m / (h m2 °C) to W m / (m2 °C)
    output[62] <- output[62] * 4186 / 3600 # Kcal / h to W
  }
  return(output)
}

run.MANMO <- function(W = rep(0.2, length(TAs)),
                      a_clo.B5 = 0.35,
                      a_skn.B4 = 0.30,
```

```
                    a.B3 = 0.15,
                    e_skin.Y4 = 0.98,
                    e_clo.Y5 = 0.95,
                    e_sfc.Y3 = 0.95,
                    Maximum.SR = 9 * 0.85,
                    K3 = c(0.014, 0.01, 0.072, 1),
                    D3 = c(0.001, 0.001, 0.001, 0.1e-10),
                    iterate = 1,
                    Tskin1.T = rep(2, length(TAs)),
                    SI.mode = 1,
                    Ht.H4 = 168,
                    Wt.W4 = 67,
                    V3 = 0,
                    G_m.G2s = 50,
                    CLO.C4 = 0,
                    TAs = 20,
                    RH.H2s = 0.05,
                    TSKYs = TAs,
                    TGNDs = TAs,
                    TMs = TAs,
                    VELs = 0.5,
                    dd.V5 = 180,
                    k_x.K2 = 0.3,
                    d_x.D2 = 0.01,
                    CLO.mode = 2,
                    manmo.mode = 2,
                    Q_hs = TAs * 0,
                    q_hs = TAs * 0,
                    Zs = TAs * 0,
                    T_clo.T9s = TAs,
                    K7 = 0,
                    K8 = 0,
                    K6 = 0.78){

MANMO.out<- matrix(data = 0, nrow = length(TAs), ncol = 64)

for(j in 1:length(TAs)){
  manout <- MANMO_R(W = W[j],
                    a_clo.B5 = a_clo.B5,
                    a_skn.B4 = a_skn.B4,
                    a.B3 = a.B3,
                    e_skin.Y4 = e_skin.Y4,
                    e_clo.Y5 = e_clo.Y5,
                    e_sfc.Y3 = e_sfc.Y3,
                    Maximum.SR = Maximum.SR,
                    K3 = K3,
                    D3 = D3,
                    iterate = iterate,
                    Tskin1.T = Tskin1.T[j],
                    SI.mode = SI.mode,
                    Ht.H4 = Ht.H4,
                    Wt.W4 = Wt.W4,
                    V3 = V3,
                    G_m.G2 = G_m.G2s[j],
                    CLO.C4 = CLO.C4,
                    T_a.T0 = TAs[j],
                    T_sky.T2 = TSKYs[j],
                    T_gnd.T7 = TGNDs[j],
                    T_m.S2= TMs[j],
                    T_clo.T9 = T_clo.T9s[j],
                    RH.H2 = RH.H2s[j],
                    ff.V2 = VELs[j],
                    dd.V5 = dd.V5,
                    k_x.K2 = k_x.K2,
                    d_x.D2 = d_x.D2,
                    CLO.mode = CLO.mode,
                    mode = manmo.mode,
                    Q_h.Q2 = Q_hs[j],
                    q_h.Q7 = q_hs[j],
```

```
                      z.K0 = Zs[j],
                      K7 = K7,
                      K8 = K8,
                      K6 = K6)
    MANMO.out[j,] <- manout
  }

  MANMO.out <- as.data.frame(MANMO.out)
  colnames(MANMO.out) <- colnames(manout)
  return(MANMO.out)
}
```
