# Supplementary material for: HomoTherm: An Open‐Source Approach to Modelling Heat Exchange in Humans and Other Hominins in Diverse Environments
Source: Glob Chang Biol. 2026 Apr 1;32(4):e70830. doi: 10.1111/gcb.70830 (PMC13044332; doi:10.1111/gcb.70830)
Supplement: Supplementary file 11 — Appendix S11: gcb70830‐sup‐0011‐Appendix 11.pdf. [file GCB-32-e70830-s001.pdf]

```
#' Created on Fri Nov  5 11:24:20 2021
#'
#' @author: Gisel Guzman-Echavarria
#'
#' # ============================================================================
#' # Objetive model:
#' # ============================================================================
#'
#' In this model, Survivability and Livability are assessed via the heat exchanges
#' required and possible by the human body as explained in Vanos et al (2023),
#' A physiological approach for assessing human survivability and liveability
#' to heat in a changing climate
#'
#' Those heat exchanges are obtained using the heat balance equation based on a
#'  partitional calorimetry approach as stated in Cramer & Jay (2018).
#'  J Appl Physiol; CORP: Partitional Calorimetry v2.0
#'
#' Disclaimer: The variables in this model follow the International System of Units (SI),
#' However there are some equations in which temperature is required in degrees Celsius.
#'
#' The implementation of this model as it is assume steady-state conditions in environmental
#' and physiological variables (Tskin and sweat rate).
#'
#' Documentation of functions in this module follows
https://www.softwaretestinghelp.com/python-docstring-tutorial/
#'
#'
#' # ============================================================================
#' # Input data in Cramer & Jay (2018):
#' # ============================================================================
#'
#' Subject Characteristics - Corporal
#'     - Mass   = Mass oh human body (kg)
#'     - Height = height of the human body (m)
#'     - AD     = Dubois-Dubois surface corporal area (m2)
#'
#'
#'     - Tsk_C  = Mean skin temperature (ºC)
#'     - Ar_AD  = Effective radiative area of the body (dimentionless)
#'
#'     - Emm_sk = Area weighted emissivity of the clothed body surface (dimentionless)
#'
#'     - Icl     =  Insulation value (Iclo)
#'     - Re_cl   = Evaporative heat transfer resistance of the clothing layer (m2·kPa·W-1)
#'
#'      - M - W, with W=0   Energy expenditure based on Look-up tables (W) as in
#'     Ainsworth, B. E., Haskell, W. L., Herrmann, S. D., Meckes, N., Bassett, D. R., Tudor-
Locke, C., Greer, J. L., Vezina, J.,
#'     Whitt-Glover, M. C., & Leon, A. S. (2011). 2011 compendium of physical activities: A
second update of codes and MET values.
#'     Medicine and Science in Sports and Exercise, 43(8), 1575–1581.
https://doi.org/10.1249/MSS.0b013e31821ece12
#'
#'     - wmax_condition =  Maximum skin wettedness (dimentionless)
#'     - Max sweat rate (L/hr)
#'
#'
#' Environmental Characteristics - Ambiental
#'     - Ta_C = Ambient temperature (ºC)
#'     - Tr_C=  Mean radiant temperature (ºC)
#'     - RH=            Relative humidity (%)
#'     - PB_kPa=        Barometric pressure (kPa)
#'     - Av_ms=     Air velocity (m·s-1)
#'
#'
#' Constants
#'     σ        5.67E-08        #W·m-2·K-4      Stefan-Boltzmann constant.
#'     Lh_vap_water     = 2430  #J·g-1  Heat of vaporisation of water at 30ºC.
#'     LR       = 16.5  #K·kPa-1        Lewis Relation.
```

```
#'
#' ##############################################################################
#' # Disclaimer, not all the convertion factors listed here were used, it was preferred
#' # to use MetPy package to move between the diferent metrics of humidity.
#' ##############################################################################
#'
#' """

# ============================================================================
# Defining constants
# ============================================================================
σ = 5.67E-08    #W·m-2·K-4      Stefan-Boltzmann constant.
LR      = 16.5  #K·kPa-1        Lewis Relation.

Lh_vap =  2426  #J·g-1  Heat of vaporisation of Sweat heat at 30ºC.
mmHg2kPa = 0.133322 # mmHg/kPa conversion
#Gagnon, D., & Crandall, C. G. (2018). Sweating as a heat loss thermoeffector.
# Handbook of Clinical Neurology, 156, 211–232. https://doi.org/10.1016/B978-0-444-63912-
7.00013-8

# ============================================================================
# Ambiental transformations, convertions and/or estimations
# ============================================================================

T_Celsius_to_Kelvin <- function(T_c) {
  # This function converts temperature from degrees Celsius to Kelvin.
  #
  # Parameters
  # ----------
  # T_c : numeric
  #   Ambient temperature in degrees Celsius
  #
  # Returns
  # ------
  # T_K : numeric
  #   Ambient temperature in degrees Kelvin

  T_K <- T_c + 273.15
  return(T_K)
}

# Function to estimate absolute humidity from ambient temperature and atmospheric pressure
Abs_Hum_from_TaC_PakPa <- function(T_C, Pa_kPa) {
  # This function estimates absolute humidity from ambient temperature and atmospheric
pressure.
  #
  # Parameters
  # ----------
  # T_C  : numeric
  #   Ambient temperature in degrees Celsius
  # Pa_kPa : numeric
  #   Atmospheric pressure in kPa
  #
  # Returns
  # -------
  # Abs_Hum: numeric
  #   Absolute Humidity in kg·m-3

  Abs_Hum <- 2.17 * (Pa_kPa / (T_C + 273.15))
  return(Abs_Hum)
}

# Function to convert wind speed from kilometers per hour to meters per second
Av_kmh_from_Av_ms <- function(Av_ms) {
  # This function converts wind speed from kilometers per hour to meters per second.
  #
  # Parameters
  # ----------
  # Av_ms : numeric
```

```
  #   Wind speed in meters per second
  #
  # Returns
  # -------
  # Av_kmh : numeric
  #   Wind speed in kilometers per hour

  Av_kmh <- Av_ms * 3.6
  return(Av_kmh)
}

# Function to estimate saturated vapour pressure from ambient temperature
Psa_kPa_from_TaC <- function(T_C) {
  # This function estimates saturated vapour pressure from ambient temperature.
  #
  # Parameters
  # ----------
  # T_C  : numeric
  #   Ambient temperature in degrees Celsius
  #
  # Returns
  # -------
  # Psa_kPa: numeric
  #   Saturated vapour pressure in kPa

  Psa_kPa <- exp(18.956 - (4030.18 / (T_C + 235))) / 10
  return(Psa_kPa)
}

# Function to estimate vapor pressure in air mixed with water vapor from vapor pressure and
relative humidity
Pv_kPa_from_Psa_RH <- function(Psa_kPa, RH) {
  # This function estimates vapor pressure in air mixed with water vapor,
  # from vapor pressure and relative humidity.
  #
  # Parameters
  # ----------
  # Psa_kPa  : numeric
  #   Saturated vapor pressure in kPa
  # RH  : numeric
  #   Relative humidity in percentage
  #
  # Returns
  # -------
  # Pv_kPa : numeric
  #   Water vapor pressure (for air mixed with water vapor) in kPa

  Pv_kPa <- Psa_kPa * (RH / 100)
  return(Pv_kPa)
}

# Function to convert METs to metabolic rate in Watts (for a given body mass)
MET_to_MetabolicRate_W_Mass <- function(METs, Mass) {
  # The conversion factor from watts to metabolic equivalents (METs) and
  # vice versa is taken as from MET definition.
  # Parameters:
  # M_MET : numeric - Energy expenditure in METS
  # Mass : numeric - Body mass in kg
  #
  # Returns:
  # M_W : numeric - Energy expenditure in Watts

  Watts <- METs * 1.225 * Mass
  return(Watts)
}

# Function to convert metabolic rate in Watts to METs (for a given body mass)
MetabolicRate_W_to_MET_Mass <- function(Watts, Mass) {
  # The conversion factor from watts to metabolic equivalents (METs) and
```

```
  # vice versa is taken as from MET definition.
  # Parameters:
  # M_WAT : numeric - Energy expenditure in METS
  # Mass : numeric - Body mass in kg
  #
  # Returns:
  # M_MET : numeric - Energy expenditure in Watts

  METs <- Watts / (1.225 * Mass)
  return(METs)
}

# Function to read personal profiles from a text file
read_personal_profiles <- function(path_profile) {
  # This function reads the personal profile of the person to run the model.
  #
  # Parameters
  # ----------
  # path_profile : character
  #   Path+name of the text file in which the personal profiles is located in the computer
  #
  # Returns
  # -------
  # profile : list
  #   Information about the personal profile to run the model

  # Define data types for columns
  dtype_profile <- list(
    id = "character",
    name = "character",
    name_file = "character",
    Mass = "numeric",
    Height = "numeric",
    AD = "numeric",
    Tsk_C = "numeric",
    A_eff = "numeric",
    Emm_sk = "numeric",
    Icl = "numeric",
    Re_cl = "numeric",
    M = "numeric",
    W = "numeric",
    wmax_condition = "character",
    wmax_rate = "numeric"
  )

  # Read data from text file
  profile <- read.table(
    file = path_profile,
    header = FALSE,
    col.names = c("label", "values"),
    colClasses = dtype_profile,
    sep = "\t",
    na.strings = "-9999"
  )

  # Convert data frame to named list
  #profile <- as.list(profile$values)

  return(profile)
}

# Example usage
#path_to_profile <- "path/to/your/profile.txt"
#personal_profile <- read_personal_profiles(path_to_profile)
#print(personal_profile)

# Function to calculate Dubois-Dubois surface corporal area
AD_from_mass_height <- function(Mass, Height) {
  # This function returns Dubois-Dubois surface corporal area.
```

```
  #
  # Parameters
  # ----------
  # Mass : numeric
  #   Mass in kg
  # Height : numeric
  #   Height in meters
  #
  # Returns
  # -------
  # ad : numeric
  #   Corporal surface area based on Dubois-Dubois equation

  ad <- 0.202 * ((Mass^0.425) * (Height^0.725))
  return(ad)
}

# Function to estimate the clothing area factor
fcl_from_Icl <- function(Icl) {
  # This function estimates the clothing area factor (dimensionless).
  #
  # Parameters
  # ----------
  # Icl : numeric
  #   Insulation value (Iclo)
  #
  # Returns
  # -------
  # fcl : numeric
  #   Clothing area factor

  fcl <- 1 + (0.31 * Icl)
  return(fcl)
}

# Function to estimate convective heat transfer coefficient
hc_cof_from_Av <- function(Av_ms) {
  # This function estimates convective heat transfer based on Parsons (2014).
  #
  # Parameters
  # ----------
  # Av_ms : numeric
  #   Wind speed in meters per second
  #
  # Returns
  # -------
  # hc_cof : numeric
  #   Convective heat transfer coefficient in W/(m2.C)

  hc_cof <- ifelse(Av_ms < 0.2, 3.61, (Av_ms^0.6) * 8.3)
  return(hc_cof)
}

# Function to estimate dry heat transfer resistance of clothing
Rcl_from_Icl <- function(Icl) {
  # This function estimates dry heat transfer resistance of clothing from "clo" units.
  #
  # Parameters
  # ----------
  # Icl : numeric
  #   Insulation value (Iclo)
  #
  # Returns
  # -------
  # Rcl : numeric
  #   Dry heat transfer resistance of clothing in m2.C/W

  Rcl <- 0.155 * Icl
  return(Rcl)
```

```
}

# Function to estimate radiative heat transfer coefficient
hr_cof_from_radiant_features <- function(Tr_C, Tsk_C, Emm_sk, A_eff) {
  # This function estimates radiative heat transfer coefficient.
  #
  # Parameters
  # ----------
  # Tr_C : numeric
  #   Mean radiant temperature in degrees Celsius
  # Tsk_C : numeric
  #   Mean skin temperature in degrees Celsius
  # Emm_sk : numeric
  #   Area weighted emissivity of the clothed body surface (dimensionless)
  # A_eff : numeric
  #   Effective radiative area of the body (dimensionless)
  #
  # Returns
  # -------
  # hr_coef : numeric
  #   Radiative heat transfer coefficient W/m2.K

  Boltzmann <- 5.67 * 10^(-8)  # W·m-2·K-4, Stefan-Boltzmann constant
  hr_cof <- 4 * Emm_sk * Boltzmann * A_eff * ((273.2 + (Tsk_C + Tr_C) / 2)^3)

  return(hr_cof)
}

# Function to estimate combined convective heat transfer coefficient
h_coef_from_hc_hr <- function(hc_cof, hr_cof) {
  # This function estimates combined convective heat transfer coefficient.
  #
  # Parameters
  # ----------
  # hc_cof : numeric
  #   Convective heat transfer coefficient in W·m-2·K-1
  # hr_cof : numeric
  #   Radiative heat transfer coefficient in W·m-2·K-1
  #
  # Returns
  # -------
  # h_cof : numeric
  #   Combined heat transfer coefficient in W·m-2·K-1

  h_cof <- hc_cof + hr_cof
  return(h_cof)
}

# Function to estimate operative temperature from radiative, convective, and air temperature
to_from_hr_tr_hc_ta <- function(hr_cof, mrt_C, hc_cof, Ta_C) {
  # This function estimates operative temperature from the radiative heat transfer coefficient,
  # mean radiant temperature, convective heat transfer coefficient, and air temperature.
  #
  # Parameters
  # ----------
  # hr_cof : numeric
  #   Radiative heat transfer coefficient in W·m-2·K-1
  # mrt_C : numeric
  #   Mean radiant temperature in degrees Celsius
  # hc_cof : numeric
  #   Convective heat transfer coefficient in W·m-2·K-1
  # Ta_C : numeric
  #   Ambient temperature in degrees Celsius
  #
  # Returns
  # -------
  # to_C : numeric
  #   Operative temperature in degrees Celsius
```

```
  to_C <- ((hr_cof * mrt_C) + (hc_cof * Ta_C)) / (hr_cof + hc_cof)
  return(to_C)
}

# Function to estimate combined dry heat loss via convection and radiation
Dry_Heat_Loss_c_plus_r <- function(Tsk_C, to_C, Icl, h_cof, Ad) {
  # This function estimates combined dry heat loss via convection and radiation.
  #
  # Parameters
  # ----------
  # Tsk_C : numeric
  #   Mean skin temperature in degrees Celsius
  # to_C : numeric
  #   Operative temperature in degrees Celsius
  # Icl : numeric
  #   Insulation clothing value in CLO
  # h_cof : numeric
  #   Combined convective heat transfer coefficient in W·m-2·K-1
  # Ad : numeric
  #   Corporal surface area in m2
  #
  # Returns
  # -------
  # Dry_Heat_Loss : numeric
  #   Combined dry heat loss via convection and radiation in W

  fcl <- fcl_from_Icl(Icl)  # ND Clothing area factor. Ratio of clothed body surface to nude
body surface.
  Rcl <- Rcl_from_Icl(Icl)  # m2·ºC·W-1 Intrinsic clothing insulation

  Dry_Heat_Loss_AD <- (Tsk_C - to_C) / (Rcl + (1 / (h_cof * fcl)))
  Dry_Heat_Loss <- Dry_Heat_Loss_AD * Ad  # Conversion from W·m-2 to W
  return(Dry_Heat_Loss)
}

# Function to estimate respiratory heat loss via convection
Cres_from_M_Ta <- function(M, Ta_C, Ad) {
  # This function estimates the respiratory heat loss via convection.
  #
  # Parameters
  # ----------
  # M : numeric
  #   Rate of metabolic energy expenditure in W
  # Ta_C : numeric
  #   Ambient temperature in degrees Celsius
  # Ad : numeric
  #   Corporal surface area in m2
  #
  # Returns
  # -------
  # Cres : numeric
  #   Respiratory heat loss via convection in W

  Cres <- 0.0014 * M * (34 - Ta_C) #* Ad
  return(Cres)
}

# Function to estimate latent respiratory heat loss
Eres_from_M_Pa <- function(M, Pa_kPa, Ad) {
  # This function estimates latent respiratory heat loss.
  #
  # Parameters
  # ----------
  # M : numeric
  #   Rate of metabolic energy expenditure in W
  # Pa_kPa : numeric
  #   Water vapor pressure (for air mixed with water vapor) in kPa
  # Ad : numeric
  #   Corporal surface area in m2
```

```
  #
  # Returns
  # -------
  # Eres : numeric
  #   Respiratory heat loss via evaporation in W

  Eres <- 0.0173 * M * (5.86618428 - Pa_kPa) #* Ad
  return(Eres)
}
# Function to estimate the amount of evaporative heat loss required for heat balance
Ereq_from_HeatFluxes <- function(M, W, Dry_Heat_Loss, Cres, Eres) {
  # This function estimates the amount of evaporative heat loss required for heat balance.
  #
  # Ereq  = (M - Wk) - (C+R) - (Cres + Eres)
  # Ereq = Hprod - Dry_Heat_Loss - CEplus_res
  #
  # Parameters
  # ----------
  # M : numeric
  #   If W corresponds to internal heat production in W, otherwise to metabolic rate in W
  # W : numeric
  #   External work done for the human body in W
  # Dry_Heat_Loss : numeric
  #   Heat dry heat transfer by radiation and convection through the skin in W
  # Cres : numeric
  #   Dry respiratory heat loss by convection in W
  # Eres : numeric
  #   Latent respiratory heat loss in W
  #
  # Returns
  # -------
  # Ereq : numeric
  #   Rate of evaporation in watts required for heat balance to the whole body

  Hprod <- M - W
  CEplus_res <- Cres + Eres
  Ereq <- Hprod  - Dry_Heat_Loss - CEplus_res

  return(Ereq)
}

# Function to estimate the evaporative heat transfer coefficient using the Lewis relation
he_cof <- function(hc_cof) {
  # This function estimates the evaporative heat transfer coefficient using the Lewis relation
(16.5 K/kPa).
  #
  # Parameters
  # ----------
  # hc_cof : numeric
  #   Convective heat transfer coefficient in W/m2·K
  #
  # Returns
  # -------
  # he_cof : numeric
  #   Evaporative heat transfer coefficient in W/m2·kPa
  # As in partitional calorimetry model excel spreadsheet from Ollie Jay

  LR <- 16.5
  he_cof <- hc_cof * LR
  return(he_cof)
}

# Function to provide the maximum skin wettedness depending on the characteristic set in the
personal profile file
wmax <- function(person_condition) {
  # This function provides the maximum skin wettedness depending on the characteristic
  # set in the personal profile file:
  #
  # ISO:
```

```
  # Unacclimated = 0.85
  # Acclimatized = 1.00
  #
  # Ravanelli et al. MSSE (2018):
  # Untrained & Unacclimated = 0.72
  # Trained & Unacclimated = 0.84
  # Trained & Acclimated = 0.95
  #
  # Morris 2021
  # 0.85 for the YNG model (Candas et al., 1979a);
  # 0.65 for the OLD model
  #
  # NOTE: This is a factor to be improved in the future once there is more data
  # around from thermal physiologists.
  #
  # Parameters
  # ----------
  # person_condition : character
  #   Describe if the person is acclimatized or not. Also if they have sweating impairments or
not.
  #
  # Returns
  # -------
  # wmax : numeric
  #   Maximum skin wettedness

  if (person_condition == 'Unacclimated') wmax <- 0.85
  else if (person_condition == 'fully acclimated') wmax <- 1
  else if (person_condition == 'Untrained & Unacclimated') wmax <- 0.72
  else if (person_condition == 'Trained & Unacclimated') wmax <- 0.84
  else if (person_condition == 'Trained & Acclimated') wmax <- 0.95
  else if (person_condition == 'YNG_Morris_2021') wmax <- 0.85
  else if (person_condition == 'OLD_Morris_2021') wmax <- 0.65
  else {
    cat('Invalid "person_condition"\n')
    wmax <- NA
  }

  return(wmax)
}

# Function to estimate the maximum evaporative heat loss for a given thermal environment and
clothing
Emax_env <- function(Psk_s, Pv_kPa, Re_cl, he_cof, Icl, AD) {
  # This function estimates the maximum evaporative heat loss for a given thermal environment
  # and clothing, also known as the biophysical evaporative heat loss.
  #
  # Parameters
  # ----------
  # Psk_s : numeric
  #   Vapor pressure at the skin surface while saturated with sweat in kPa
  # Pv_kPa : numeric
  #   Ambient vapor pressure in kPa
  # Re_cl : numeric
  #   Evaporative resistance of clothing in m2·kPa·W-1
  # he_cof : numeric
  #   Evaporative heat transfer coefficient in W·m-2·kPa-1
  # Icl : numeric
  #   Insulation value (Iclo)
  # AD : numeric
  #   Dubois surface corporal area in m2
  #
  # Returns
  # -------
  # Emax_env: numeric
  #   Biophysical evaporative heat loss (caused by ambient environment and the
  #   clothes people wear) in Watts

  fcl <- fcl_from_Icl(Icl)  # Ratio of clothed body surface to nude body surface
```

```
(dimensionless)

  Emax_env_AD <- (Psk_s - Pv_kPa) / (Re_cl + (1 / (he_cof * fcl)))
  Emax_env <- Emax_env_AD * AD
  return(Emax_env)
}

# Function to estimate the biophysical evaporative heat loss accounting as an additional
constraint
# the capacity to physiologically wet the skin and thus distribute sweat across the skin
surface in humid environments.
# This restriction is applied using the maximum skin wettedness.
Emax_wettedness <- function(wmax, Psk_s, Pv_kPa, Re_cl, he_cof, Icl, AD) {
  # This function estimates the biophysical evaporative heat loss accounting as an additional
constraint
  # the capacity to physiologically wet the skin and thus distribute sweat across the skin
surface in humid environments.
  # This restriction is applied using the maximum skin wettedness.
  #
  # Parameters
  # ----------
  # wmax : numeric
  #   Maximum or critical skin wettedness (dimensionless)
  # Psk_s : numeric
  #   Vapor pressure at the skin surface while saturated with sweat in kPa
  # Pv_kPa : numeric
  #   Ambient vapor pressure in kPa
  # Re_cl : numeric
  #   Evaporative resistance of clothing in m2·kPa·W-1
  # he_cof : numeric
  #   Evaporative heat transfer coefficient in W·m-2·kPa-1
  # Icl : numeric
  #   Insulation value (Iclo)
  # AD : numeric
  #   Dubois surface corporal area in m2
  #
  # Returns
  # -------
  # Emax_wettedness: numeric
  #   Biophysical evaporative heat loss modified by the constrain of max wettedness in Watts

  fcl <- fcl_from_Icl(Icl)  # Ratio of clothed body surface to nude body surface
(dimensionless)

  Emax_wettedness_AD <- wmax * (Psk_s - Pv_kPa) / (Re_cl + (1 / (he_cof * fcl)))
  Emax_wettedness <- Emax_wettedness_AD * AD
  return(Emax_wettedness)
}

# Function to estimate the evaporative heat loss after the evaporation of the maximum volume of
sweat
# that people can release based on the maximum hourly sweat rate (Smax) and accounting sweat
efficiency. (r)
Emax_sweat_rate <- function(Smax, Lh_vap, density, r) {
  # This function estimates the evaporative heat loss after the evaporation of the maximum
volume of sweat
  # that people can release based on the maximum hourly sweat rate (Smax) and accounting sweat
efficiency. (r)
  #
  # Parameters
  # ----------
  # Smax : numeric
  #   Maximum sweat rate for a given personal profile in L/h
  # Lh_vap: numeric
  #   Heat latent of vaporization of sweat in J·g-1 or the amount of energy in the form of
  #   enthalpy that is added to the air when sweat evaporates
  # density: numeric
  #   Density of sweat, assumed here as
  # r : numeric
```

```
  #   Sweating efficiency (dimensionless)
  #
  # Returns
  # -------
  # Emax_sweat_rate : numeric
  #   Maximum evaporative heat loss in W linked with the sweat evaporation of the maximum sweat
rate

  Emax <- ((Smax * Lh_vap * density) / 3.6) * r
  return(Emax)
}


# Function to estimate biophysical skin wettedness required (ω_req) for heat balance.
wreq_HSI_skin_wettedness <- function(Ereq, Emax_env) {
  # This function estimates biophysical skin wettedness required (ω_req) for heat balance.
  #
  # Parameters
  # ----------
  # Ereq : numeric
  #   Evaporative heat rate required for heat balance  W
  # Emax_env : numeric
  #   Biophysical evaporative heat loss (environment + clothing) in W
  #
  # Returns
  # -------
  # wreq: numeric
  #   Biophysical skin wettedness required for heat balance

  wreq <- Ereq / Emax_env
  return(wreq)
}

# Function to estimate sweating efficiency based on the skin wettedness.
Sweating_efficiency_r <- function(wreq) {
  # This function estimates the sweating efficiency based on the skin wettedness.
  # That value can be taken as the proportion of sweat produced that is not dripped off
  # the body and evaporated from the skin surface, thus contributing to evaporative heat loss.
  # A minimum of sweating efficiency was set at 0.55 based on (Candas et al., 1979a).
  #
  # Note that the minimum value is set to 0.5 at wreq = 1, for future applications in cold
conditions
  # ask if there is a need to truncate the r to 1.
  #
  # Parameters
  # ----------
  # wreq: numeric
  #   Skin wettedness also know as Heat Stress Index
  #
  # Returns
  # -------
  # r : numeric
  #   Sweating efficiency (dimensionless)

  r <- ifelse(wreq < 1, 1 - (wreq^2) / 2, 0.5)
  r <- ifelse(r > 1, 1, r)
  return(r)
}

# Function to estimate the required sweat rate to maintain heat balance
# and therefore compensate an imposed thermal load.
Sreq <- function(Ereq, r, Lh_vap) {
  # This functions estimates the required sweat rate to maintain heat balance
  # and therefore compensate an imposed thermal load.
  #
  # In this equation 3.6 is the conversion factor to account the time from hours
  # to seconds and the volume of sweat to mass, assuming that sweat density like
  # water density.
  #
```

```
  # Parameters
  # ----------
  # Ereq : numeric
  #   Rate of evaporation required for heat balance to whole body
  # Lh_vap: numeric
  #   heat latent of vaporization of sweat in J·g-1 or the amount of energy in form of
  #   enthalpy that is add to the air when sweat evaporates
  # r : numeric
  #   Sweating efficiency (dimensionless)
  #
  # Returns
  # -------
  # Sreq : numeric
  #   Required sweat rate L·h-1

  Sreq <- ((Ereq / r) / Lh_vap) * 3.6
  return(Sreq)
}

# Function to estimate the critical rate of heat storage (Ssurv) before inevitable
# heat stroke death during rest for exposure times of 6 and 3 hours.
# Here is assumed to have a linear increase of 6°C (Starting temperature at 37 °C)
# and a Human body-specific heat capacity of C_p= 2.98 kJ/(Kg.°C).
# Cp: Xiaojiang Xu, Timothy P. Rioux & Michael P. Castellani (2022) The specific heat of the
human
# body is lower than previously believed: The Journal Temperature toolbox, Temperature,
# DOI: 10.1080/23328940.2022.2088034
Ssurvive_from_Exposure_time <- function(Exp_time, deltaT) {
  # This function estimates the critical rate of heat storage (Ssurv) before inevitable
  # heat stroke death during rest for exposure times of 6 and 3 hours.
  #
  # Parameters
  # ----------
  # Exp_time : numeric
  #   Exposure time in hours (valid values: 1, 3, 6)
  #
  # Returns
  # -------
  # Ssurvive : numeric
  #   Critical rate of heat storage in W (J/s)

  if (!(Exp_time %in% c(1, 3, 6))) {
    stop("results: status must be one of 1, 3, 6.")
  }

  # Estimation of constant power along exposure time
  DQ <- 2.98 * deltaT  # CP.dT (change of temperature of deltaT degrees)
  Ssurvive <- (DQ * 1000) / (Exp_time * 3600)  # 1000 is the conversion from kJ to J, and 3600
is the conversion from seconds to hours

  return(Ssurvive)
}

# Function to assess survivability on humans before inevitable
# heat stroke death during rest for exposure times of 6 and 3 hours
# in a given thermal environment. In the function Ssurvive_from_Exposure_time of this
# very same module is the estimation of the critical rate of heat storage
# (Ssurv) before death.
#
# ** This assessment assumes People will survive, even if they cannot thermally
# compensate the environment and sometimes the heat storage can be greater than zero,
# while the body core temperature does not surpass 43°C
#
# ** See all the environmental and physiological assumptions in the main paper.
#
# After ends the decision-making process of the algorithm also is assigned a flag
# that categorized the survivability type according to the physiological
# constraints imposed in this model (See in Supplemental material, Figure S1 Model workflows
for (a)
```

```
#     survivability and (b) liveability)
#
# Thus, the algorithm determines survivability (as a dichotomous variable: yes/no)
# and assigns outcomes based on combined environmental and physiological restrictions.
#
# Based on this framework, a person will (Notice the numbers represent the survivability
# zones in the output flag_survivability):
#
# 1. survive while remaining within sweating limits
# 2. survive despite exceeding sweating limits
# 3. not survive because the environment restricts heat loss too much (in high humidity)
# 4. not survive because the required sweat rate is not possible (in low humidity)
# 5. not survive due to both critical environmental heat loss restrictions (3rd argument) and
not possible sweat rate to dissipate heat (4th argument).
Survivability <- function(Exp_time, Ereq, Emax_wettedness, Emax_sweat, Sreq, Smax, Mass,
deltaT) {
  # This function assesses survivability on humans before inevitable
  # heat stroke death during rest for exposure times of 6 and 3 hours
  # in a given thermal environment.
  #
  # Parameters
  # ----------
  # Exp_time : numeric
  #   Exposure time in hours. Valid values are: 1, 3, 6
  # Ereq : numeric
  #   Rate of evaporation in Watts required for heat balance to the whole body
  # Emax_wettedness : numeric
  #   Biophysical evaporative heat loss modified by the constraint of max wettedness in Watts
  # Emax_sweat : numeric
  #   Maximum evaporative heat loss in Watts linked with the sweat evaporation of the maximum
sweat rate
  # Sreq : numeric
  #   Required sweat rate in L·h-1
  # Smax : numeric
  #   Maximum sweat rate for a personal profile in L·h-1
  # Mass : numeric
  #   Mass of a person in kg
  #
  # Returns
  # -------
  # survivability : logical
  #   Could a person with a given personal profile exposed to a certain survive a given thermal
environment? yes, no
  # flag_survivability : numeric
  #   Flag that indicates the survivability type according to the physiological constraints
imposed in this model

  valid <- c(1, 3, 6)
  if (!(Exp_time %in% valid)) {
    stop("results: status must be one of 1, 3, 6.")
  }

  Ssurvive <- Ssurvive_from_Exposure_time(Exp_time, deltaT)

  # For the explanation of the workflow or criteria followed here, also See
  # Figure S1 (Model workflows for (a) survivability and (b) liveability) in the supplemental
material.
  # Criteria 1:
  condition1 <- (Ereq - Emax_wettedness) <= (Ssurvive * Mass)

  # Criteria 2.1:
  condition21 <- Sreq <= Smax

  # Criteria 2.2:
  condition22 <- (Ereq - Emax_sweat) <= (Ssurvive * Mass)

  # Assessing survivability:
  survivability <- rep(FALSE, length(condition1))
  flag_survivability <- rep(NA, length(condition1))
```

```
  # survivability[condition1 == FALSE] <- FALSE
  flag_survivability[condition1 == FALSE] <- 3

  aux1 <- condition1 & condition21  # if condition 1 and 2.1 are true
  survivability[aux1] <- TRUE
  flag_survivability[aux1] <- 1

  aux2 <- condition1 & !condition21  # if condition is true 1 and 2.1 is false
  aux3 <- aux2 & condition22  # Emax_sweat from Smax is enough
  survivability[aux3] <- TRUE
  flag_survivability[aux3] <- 2

  aux4 <- aux2 & !condition22  # Emax_sweat from Smax is not enough
  # survivability[aux4 == FALSE] <- FALSE
  flag_survivability[aux4] <- 4

  aux5 <- !condition1 & !condition22
  flag_survivability[aux5] <- 5

  return(list(survivability, flag_survivability))
}

# Liveability is the maximum metabolic rate (Mmax) that can be generated before S≥0,
# or sustained compensable heat stress, with M = Hprod. The Mmax value indicates the
# sustained activity levels (intensity but not duration) possible without unchecked
# rises in Tcore (i.e., uncompensable heat stress) within a given steady-state
# thermal environment.
#
# After checking that heat stress is compensable (Ereq≤Emaxlim), then Mmax is estimated
# as follows.
#
# M_max  = Emax_lim  -  H_loss
#
# Notice that:
#     - Emax_lim is the minimum value among Emax_wettedness and Emax_sweat
#     - H_loss can be represented either by (Hdry +Cres +Eres) or (Ereq + Hprod).
#
# Also, if Mmax is zero or less than zero, it means the thermal load is non-compensable,
# then that person can survive but is not able to live (no activity is possible
# without storage heat internally).
# However, there is a limit, and that is why the survivability assessment is
# accounted for this variable.
#
# Parameters
# ----------
# survivability : logical
#     Boolean variable indicating if a person can survive in a given thermal environment
# Ereq : numeric
#     Rate of evaporation in Watts required for heat balance to the whole body
# Emax_wettedness : numeric
#     Biophysical evaporative heat loss modified by the constraint of max wettedness in Watts
# Emax_sweat : numeric
#     Maximum evaporative heat loss in Watts linked with the sweat evaporation of the maximum
sweat rate
# M_rest : numeric
#     Metabolic energetic expenditure while people are resting in W (here M_rest = than Hprod)
#
# Returns
# -------
# Mmax : numeric
#     Maximum metabolic rate in W that a person can tolerate after surviving,
#     that reflects the range of activities could be performed
#
# mask_non_livable : logical
#     Flag to indicate if these environmental conditions lead to a survivable but not
#     liveable condition (no activity is possible without storage heat internally).
livability_Mmax <- function(survivability, Ereq, Emax_wettedness, Emax_sweat, M_rest) {
  Emax_constrain <- pmin(Emax_wettedness, Emax_sweat)
```

```
  compensability <- Ereq < Emax_constrain
  Mmax <- Emax_constrain - Ereq + M_rest
  Mmax[!compensability] <- NA
  non_livable <- survivability & !compensability
  return(list(Mmax, non_livable))
}

# This function extracts the survivability limits from the survivability
# results of the model (yes/no matrix), given the assessment results
# from a temperature and humidity range.
#
# This works from assessments based on arrays of the temperature and humidity matrix, with
# other values fixed. But notice, if windspeed, radiation, pressure, and any feature in
# the personal profiles change this lines and still this function could be used
# in such cases ONLY if those variables are constant.
#
# Parameters
# ----------
# Survibability: logical
#     Matrix with the boolean results from the survivability assessment
# humidity : numeric
#     Array with the humidities evaluated in the model
# type_humidity : character
#     Name of the humidity metric used in the analysis
# temperature : numeric
#     Array with the humidities evaluate in the model
# tw : data.frame
#     Array with
#
# Returns
# -------
# New_survivability : data.frame
SurvivabilityLines_from_SurvivabilityMatrix <- function(Survivability, humidity, type_humidity,
temperature, tw) {
  New_survivability <- data.frame(matrix(NA, nrow = length(humidity), ncol = 3))
  colnames(New_survivability) <- c(type_humidity, 'Tair', 'Tw')
  New_survivability[, type_humidity] <- humidity


  for (i in 1:length(humidity)) {
    index_array_h <- which(humidity == humidity[i])[1]
    survivability_row <- Survivability[index_array_h, ]
    index_change_true_false <- which(diff(survivability_row) != 0)

    # Check if indices change
    if (length(index_change_true_false) > 0) {
      dbt_limit <- temperature[index_change_true_false]
      tw_limit <- tw[index_array_h, index_change_true_false]
      New_survivability$Tair[i] <- dbt_limit
      New_survivability$Tw[i] <- tw_limit
    } else {
      # Handle case when no indices change
      New_survivability$Tair[i] <- NA
      New_survivability$Tw[i] <- NA
    }
  }


  return(New_survivability)
}

# Colormap
Survivability_cmap <- function() {
  colores_cat <- matrix(c(
    c(252/255, 252/255, 205/255),
    c(237/255, 232/255, 131/255),
    c(254/255, 192/255, 169/255),
    c(205/255, 206/255, 254/255),
    c(191/255, 200/255, 209/255)
```

```
  ), ncol = 3, byrow = TRUE)

  cmap_survivability <- colorRampPalette(colores_cat, space = "rgb")(256)
  bins_survivability <- c(0.5, 1.5, 2.5, 3.5, 4.5, 5.5)
  norm_survivability <- cut(1:256, breaks = c(0, bins_survivability, 256), include.lowest =
TRUE)
  ticks_survivability <- c(1, 2, 3, 4, 5)

  return(list(cmap_survivability, bins_survivability, norm_survivability, ticks_survivability))
}

# Example usage:
# colormap_result <- Survivability_cmap()
# cmap_survivability <- colormap_result[[1]]
# bins_survivability <- colormap_result[[2]]
# norm_survivability <- colormap_result[[3]]
# ticks_survivability <- colormap_result[[4]]

run_HHB <- function(exp_time = 6,
                    AD = 1.6,
                    M = 68.845,
                    Tsk_C = 35,
                    Emm_sk = 0.98,
                    Ar_AD = 0.7,
                    Icl = 0,
                    Re_cl = 0,
                    Ta_C = 20,
                    humidity = 50,
                    Av_ms = 0.1,
                    mrt_C = 20,
                    wmax_condition = 1,
                    Smax = 1.5,
                    Mass = 56.2,
                    deltaT = 6
){

  Psa_kPa <- Psa_kPa_from_TaC(Ta_C)
  Pv <- Pv_kPa_from_Psa_RH(Psa_kPa, humidity)
  Pv_kPa <- Pv

  hc_cof  = hc_cof_from_Av(Av_ms) #W/m2K Convective heat transfer coefficient.
  hr_cof  = hr_cof_from_radiant_features(mrt_C,Tsk_C,Emm_sk,Ar_AD) #Linear radiative heat
transfer coefficient (W/m2K).
  h_cof   = h_coef_from_hc_hr(hc_cof,hr_cof) # Combined heat transfer coefficient (W/m2K).
  to_C    = to_from_hr_tr_hc_ta(hr_cof,mrt_C,hc_cof,Ta_C) #Operative temperature (ºC)

  #Estimation of combined dry heat loss via convection and radiation (W)
  Dry_Heat_Loss =  Dry_Heat_Loss_c_plus_r(Tsk_C,to_C,Icl,h_cof,AD)

  # Heat loss from respiration (Convection + Evaporation)

  # Estimation of dry respiratory heat loss (W)
  Cres = Cres_from_M_Ta(M,Ta_C,AD)
  # Estimation of latent respiratory heat loss (W)
  Eres = Eres_from_M_Pa(M,Pv_kPa,AD)

  #Once we have all the avenues of heat exchange (Equation 4 in the supplemental material), we
can estimate the Evaporative required heat loss Ereq (Equation 14 in the supplemental
material).
  #Re_cl = as.numeric(profile$Re_cl) #Evaporative resistance of clothing in m2·kPa/W

  #Estimation of evaporative required heat loss (W)
  Ereq = Ereq_from_HeatFluxes(M,0,Dry_Heat_Loss,Cres, Eres)

  # Now, we are getting closer, and we are ready to estimate the different evaporative heat
loss restrictions (Emax_env, Emax_wettedness, and Emax_sweat) that will ultimately determine if
a person can survive or not in a thermal environment for 3 or 6 hours of steady-state exposure.
  # Estimation biophysical (environmental + clothing) evaporative heat loss limit
  # This is directly linked with the humidity gradient between the environment and the skin
```

```
surface and clothing resistance in the heat exchange (Equation 16 in the supplemental
material). In very high humid climates, the drivers of evaporation are highly limiting.
  #Water vapour pressure at the skin (kPa), assumed to be that of saturated water (100% HR)
vapour at skin temperature.
  Psk_s= Psa_kPa_from_TaC(Tsk_C)
  # Evaporative heat transfer coefficient (W/m2kPa).
  he_cof1 = he_cof(hc_cof)

  # Estimation of biophysical Emax (Env + clothing) (W)
  Emax_env1 = Emax_env(Psk_s,Pv_kPa,Re_cl,he_cof1,Icl,AD)
  Emax_env1[Emax_env1<0] = 0 #This heat flux can not be negative.

  # Estimation of the physiological (environmental + clothing + sweating capacity) evaporative
heat loss limit
  # This is linked with the physiological capacity to saturate the skin surface due to a
limited maximum skin wettedness (wmax) given their age and/or degree of
acclimation/acclimatization, among other factors. In this evaporative limit, the biophysical
limit is scaled by the maximum skin wettedness (Equation 19 in the supplemental material).
  # Maximum skin wettedness: maximum portion of total body surface area that can be saturated
with sweat.
  wmax1 = wmax_condition

  # Physiological Emax (Env + clothing + wettedness)
  Emax_wettedness1 = Emax_wettedness(wmax1,Psk_s,Pv_kPa,Re_cl,he_cof1,Icl,AD)
  Emax_wettedness1[Emax_wettedness1<0] = 0   #This heat flux can not be negative.

  # Estimation of the physiological evaporative heat loss limit given by sweat production
  # In very hot and dry environments, evaporation is so efficient, and the sweating rate
required to attain Emax_env may not be physiologically possible. The evaporative heat loss
limit should account for the maximum rate (Smax, from profile dictionary) at which a person can
segregate sweat.
  # To be able to estimate the required sweat rate Sreq using the required evaporative heat
loss to achieve heat balance Ereq , it is also necessary to know ahead of the expected sweating
efficiency r, estimated as follows:

  # Estimation of required skin wettedness (dimensionless)
  wreq = wreq_HSI_skin_wettedness(Ereq,Emax_env1)
  # Estimation of expected sweating efficiency (dimensionless)
  r = Sweating_efficiency_r(wreq)
  #Estimation of required sweat rate  to maintain heat balance (L/h).
  Sreq1 =  Sreq(Ereq,r,Lh_vap)

  #Disclaimer: Emax_sweat assumes that all sweat evaporates over the skin, and the heat loss is
equivalent to the heat released by sweat's latent heat of vaporization.
  Smax = Smax #Maximum sweat rate (L/h).

  #Estimation of the physiological evaporative heat loss based on capacity to secret sweat
  Emax_sweat = Emax_sweat_rate(Smax,Lh_vap,1,r)


  survivability1 =
Survivability(exp_time,Ereq,Emax_wettedness1,Emax_sweat,Sreq1,Smax,Mass,deltaT)
  flag_survivability <- survivability1[[1]]
  if(flag_survivability){
    survive <- 1
  }else{
    survive <- 0
  }
  survivability <- survivability1[[2]]
  results <- c(Dry_Heat_Loss, Cres, Eres, Ereq, Emax_env1, wmax1, Emax_wettedness1, wreq, r,
Sreq1, Smax, Emax_sweat, survivability, survive)
  names(results) <- c("Dry_Heat_Loss", "Cres", "Eres", "Ereq", "Emax_env", "wmax",
"Emax_wettedness", "wreq", "r", "Sreq", "Smax", "Emax_sweat", "survivability", "survive")
  return(results)
}
```
