# Supplementary material for: HomoTherm: An Open‐Source Approach to Modelling Heat Exchange in Humans and Other Hominins in Diverse Environments
Source: Glob Chang Biol. 2026 Apr 1;32(4):e70830. doi: 10.1111/gcb.70830 (PMC13044332; doi:10.1111/gcb.70830)
Supplement: Supplementary file 12 — Appendix S12: gcb70830‐sup‐0012‐Appendix 12.pdf. [file GCB-32-e70830-s004.pdf]

```
# constants
Boltzmann <- 5.67*10^-8 #W·m-2·K-4  Stefan-Boltzmann constant.
LR <- 16.5  #K·kPa-1    Lewis Relation.
Lh_vap <- 2426  #J·g-1  Heat of vaporisation of Sweat heat at 30ºC.
density <- 1 # density of water, g/cm^3

# Human parameters
Mass <- 60 # mass, kg
Icl <- 0 # clo, -
Emm_sk <- 0.98 # skin emissivity, -
Ad <- 1.7 # DuBois area, m^2
A_eff <- Ad*0.78 # effective radiation area, m^2
M <- 60 # metabolic rate, W
W <- 0 # external work, W
Re_cl <- 0 # resistance of clothing to evaporation, m2·kPa/W
Wmax <- 1 # maximum skin wettedness
Smax <- 0.75 # maximum sweat rate
deltaT <- 6 # difference between normal and lethal core temperature
Exp_time <- 6 # exposure time to the environment

# Environmental variables
Av_ms <- 1 # wind speed, m/s
Tsk_C <- 35 # skin temp, deg C
mrt_C <- 20 # mean radiant temp, deg C
Ta_C <- 20 # air temp, deg C
RH <- 50 # relative humidity, %

# Equations, 24 lines
fcl <- 1 + (0.31 * Icl)
Rcl <- 0.155 * Icl
hc_cof <- ifelse(Av_ms < 0.2, 3.61, ((Av_ms ^ 0.6) * 8.3))
hr_cof <- 4 * Emm_sk * Boltzmann * A_eff * ((273.2 + (Tsk_C + mrt_C) / 2) ^ 3)
h_cof <- hc_cof + hr_cof
to_C <- ((hr_cof * mrt_C) + (hc_cof * Ta_C)) / (hr_cof + hc_cof)
Dry_Heat_Loss <- (Tsk_C - to_C) / (Rcl + (1 / (h_cof * fcl))) * Ad
Hprod <- M - W
Cres <- 0.0014 * M * (34 - Ta_C)
Psa_kPa <- exp(18.956 - (4030.18 / (Ta_C + 235))) / 10
Psk_s <- exp(18.956 - (4030.18 / (Tsk_C + 235))) / 10
Pv_kPa <- Psa_kPa * (RH / 100)
Eres <- 0.0173 * M * (5.86618428 - Pv_kPa)
CEplus_res <- Cres + Eres
Ereq <- Hprod - Dry_Heat_Loss - CEplus_res
He_cof <- hc_cof * LR
Emax_env1 <- (Psk_s - Pv_kPa) / (Re_cl + (1 / (He_cof * fcl))) * Ad
Emax_wettedness1 <- Wmax * (Psk_s - Pv_kPa) / (Re_cl + (1 / (He_cof * fcl))) * Ad
wreq <- Ereq / Emax_env1
r <- min(1, ifelse(wreq < 1, 1 - (wreq ^ 2) / 2, 0.5))
Sreq1 <- ((Ereq / r) / Lh_vap) * 3.6
Emax_sweat <- ((Smax * Lh_vap * density) / 3.6) * r
DQ <- 2.98 * deltaT
Ssurvive <- (DQ * 1000) / (Exp_time * 3600)

# testing for surivivability
condition1 <- (Ereq - Emax_wettedness1) <= (Ssurvive * Mass)
condition21 <- Sreq1 <= Smax
condition22 <- (Ereq - Emax_sweat) <= (Ssurvive * Mass)
survivability <- rep(FALSE, length(condition1))
flag_survivability <- rep(NA, length(condition1))
flag_survivability[condition1 == FALSE] <- 3
aux1 <- condition1 & condition21  # if condition 1 and 2.1 are true
survivability[aux1] <- TRUE
flag_survivability[aux1] <- 1
aux2 <- condition1 & !condition21  # if condition is true 1 and 2.1 is false
aux3 <- aux2 & condition22  # Emax_sweat from Smax is enough
survivability[aux3] <- TRUE
flag_survivability[aux3] <- 2
aux4 <- aux2 & !condition22  # Emax_sweat from Smax is not enough
flag_survivability[aux4] <- 4
```

```
aux5 <- !condition1 & !condition22
flag_survivability[aux5] <- 5
if(flag_survivability){
  survive <- 1
}else{
  survive <- 0
}

results <- c(Dry_Heat_Loss, Cres, Eres, Ereq, Emax_env1, Wmax, Emax_wettedness1, wreq, r,
Sreq1, Smax, Emax_sweat, survivability, survive)
names(results) <- c("Dry_Heat_Loss", "Cres", "Eres", "Ereq", "Emax_env", "wmax",
"Emax_wettedness", "wreq", "r", "Sreq", "Smax", "Emax_sweat", "survivability", "survive")
```
