# Supplementary material for: HomoTherm: An Open‐Source Approach to Modelling Heat Exchange in Humans and Other Hominins in Diverse Environments
Source: Glob Chang Biol. 2026 Apr 1;32(4):e70830. doi: 10.1111/gcb.70830 (PMC13044332; doi:10.1111/gcb.70830)
Supplement: Supplementary file 13 — Appendix S13: gcb70830‐sup‐0013‐Appendix 13.pdf. [file GCB-32-e70830-s013.pdf]

# Comparison of HomoTherm with Ruxton and Wilkinson 2011 version of the Wheeler model for extinct homonins

Michael Kearney

2026-01-07

### Overview

A comparison of the model against the model and simulations reported in Ruxton and Wilkinson (2011a,b), which were based on Wheeler's (1990) model. The aim of this simulation is simply to show how one would undertake the same kind of analysis as Ruxton and Wiklinson's using the Homotherm model. The integration of the microclimate modelling capacity of NicheMapR would improve the realism of the simulated microclimates beyond the simple functions used by Ruxton and Wilkinson.

### Load the libraries and data

```
library(NicheMapR)
```

### Ruxton & Wilkinson model

Note that this model does not account for water vapour pressure. The full code for the Ruxton and Wilkinson and Wheeler models were not provided. The code below closely, but not completely, recreates the plots in Ruxton and Wilkinson (2011a). See also David-Barret and Dunbar (2016). Note that skin temperature is (unrealistically) assumed equal to core in this model and core temperature was not assumed to rise under heat load.

```
# environmental parameters
tau_200 <- 40 # max air temp at 200 cm, deg C
alpha <- 0.41 # wind speed correction factor, -
Smax <- 865 # max direct solar radiation, W/m2
r <- 0.15 # substrate reflectivity, -
sigma <- 5.67e-8 # W/m2/K^4
T_a_range <- 25 # deg C, difference between min and max air temp at 200 cm
T_g_range <- 35 # deg C, difference between min and max ground temp at 200 cm
t_min <- 5 # time of minimum air temperature, deg C
delta_Tg <- 5 # offset of max ground temp relative to max air temp, deg C

# hominin parameters
T_c <- 37 # skin temperature, deg C
a <- 0.8 # absorptivity of hair, -
C <- 1 # W/m2/C, conductance of pelt
k <- 9.8 # W/m2/C, convection coefficient
M <- 74 # kg, mass
```

```r
L <- 0.83 # leg length, m
f_hair <- 0.15 # fraction of body covered by thick hair
f_sun_max <- 0.23 # max fraction of body exposed to sun
Q_sweat_max <- 500 # W/m2, max sweat rate

# environmental variables

t <- seq(5, 19, 0.2) # time of day, h

T_200 <- tau_200 - T_a_range + T_a_range *
  sin(pi * (t - t_min) / 18) # 200 cm air temperatures, deg C
TGRDs <- tau_200 + delta_Tg - T_g_range +
  T_g_range * sin(pi * (t - t_min) / 16) # ground temperature
TAs <- T_200 + alpha * (TGRDs - T_200)
plot(t, T_200, pch = 16, ylim = c(-10, 50), ylab = 'temperature, deg C', xlab = 'hour of day')
points(t, TAs, pch = 16, col = 'blue')
points(t, TGRDs, pch = 16, col = 'red')
legend(13, 10, legend = c('2m air', 'hominin air', 'ground'), pch = 16,
       col = c('black', 'blue', 'red'))
```

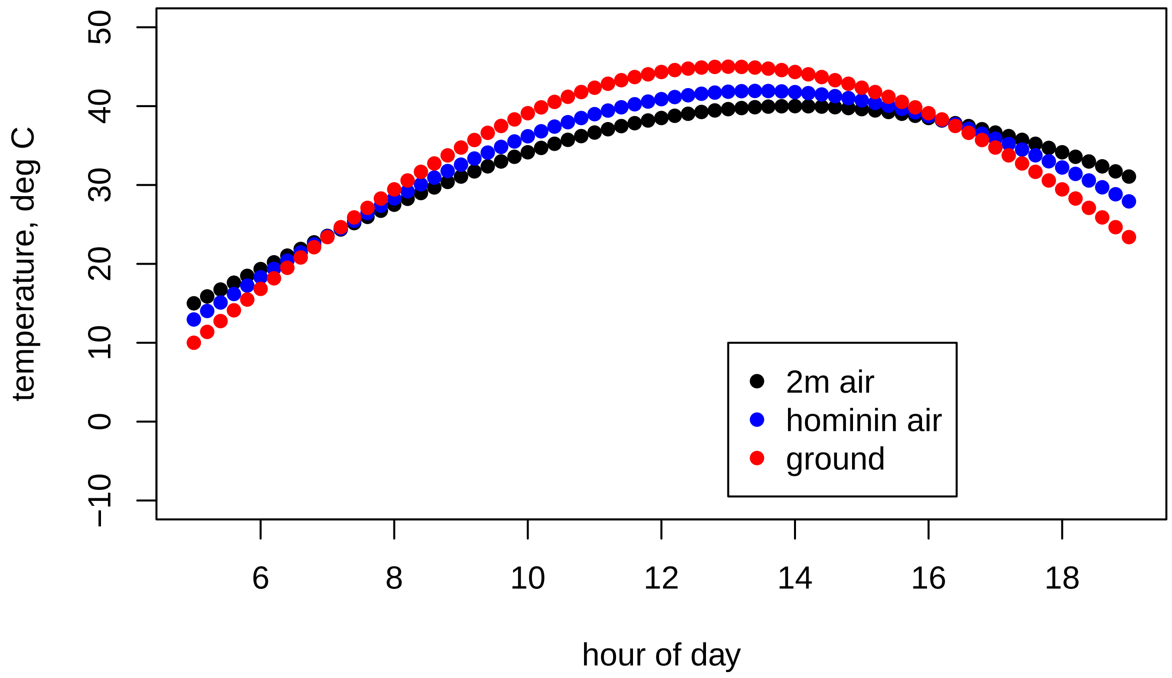


```r
VEL <- 3.5 * L^0.5 # wind speed, m/s
VELs <- rep(VEL, length(TAs))# wind speeds, m/s
S <- Smax * sin(pi * (t - 5) / 16) # direct solar, W/m2
S[S < 0] <- 0
plot(t, S, pch = 16, ylim = c(0, Smax), ylab = 'solar radiation, W/m2')
```

```r
s <- S / 9 # 10% diffuse solar, W/m2
s[s < 0] <- 0
points(t, s, pch = 16, col = 'grey')
legend(13, 400, legend = c('direct', 'scattered'), pch = 16,
       col = c('black', 'grey'))
QSOLRs <- S + s
#plot(t, QSOLRs, pch = 16, ylim = c(0, Smax * 1.1))
R_reflect <- r * QSOLRs
Rsky <- 213 + 5.5 * T_200
TSKYs <- (Rsky / sigma)^(1 / 4) - 273.15 # deg C
Rg <- sigma * (TGRDs + 273.15)^4

# heat budget calculations
Q_inc_vent <- 0.5 * (s + r * (S + s) + Rsky + Rg) # indirect radiation, W/m2
Q_inc_dors <- S + Q_inc_vent # direct radiation, W/m2
Q_abs_vent <- 0.5 * (a * s + a * r * (S + s) + Rsky + Rg) # indirect radiation exchange through coat, W
Q_abs_dors <- a * s + Q_abs_vent # direct radiation through coat, W/m2

#plot(Q_abs_dors, pch = 16)
#points(Q_abs_vent, pch = 16, col = 'grey')

# get surface temperature
get_bal <- function(T_s) {
  x <- Q_ab - (sigma * (T_s + 273.15)^4 + k * v^0.5 * (T_s - T_a))
  - C * (T_s - T_c)
}

T_s_dors_coat <- NULL
for (i in 1:length(TAs)) {
  T_a <- TAs[i]
  v <- VELs[i]
  Q_ab <- Q_abs_dors[i]
  T_s <- uniroot(f = get_bal, c(T_a - 5, 100), check.conv = TRUE)$root
  T_s_dors_coat[i] <- T_s
}
#plot(t, T_s_dors_coat, pch = 16, col = 2)

T_s_vent_coat <- NULL
for (i in 1:length(TAs)) {
  T_a <- TAs[i]
  v <- VELs[i]
  Q_ab <- Q_abs_vent[i]
  T_s <- uniroot(f = get_bal, c(T_a - 5, 100), check.conv = TRUE)$root
  T_s_vent_coat[i] <- T_s
}
#points(t, T_s_vent_coat, pch = 16)

# pelt gain
Q_gain_dors_coat <- Q_abs_dors - (sigma * (T_s_dors_coat + 273.15)^4 + k
                                  * VELs^0.5 * (T_s_dors_coat - TAs))
Q_gain_vent_coat <- Q_abs_vent - (sigma * (T_s_vent_coat + 273.15)^4 + k
                                  * VELs^0.5 * (T_s_vent_coat - TAs))
Q_gain_dors_skin <- Q_inc_dors - (sigma * (T_c + 273.15)^4 + k
```

```
                                        * VELs^0.5 * (T_c - TAs))
Q_gain_vent_skin <- Q_inc_vent - (sigma * (T_c + 273.15)^4 + k
                                        * VELs^0.5 * (T_c - TAs))

#plot(Q_gain_dors_skin, pch = 15, col = 1)
#points(Q_gain_vent_skin, pch = 15, col = 2)
#points(Q_gain_dors_coat, pch = 16, col = 1)
#points(Q_gain_vent_coat, pch = 16, col = 2)

A <- 0.11 * M^0.67 # total surface area, m2
beta <- f_sun_max - f_hair * sin((t - 6) * pi / 12) # fract sun-exposed skin
#plot(beta, type = 'l')
abline(h = 0.23)
abline(h = 0.08)
```

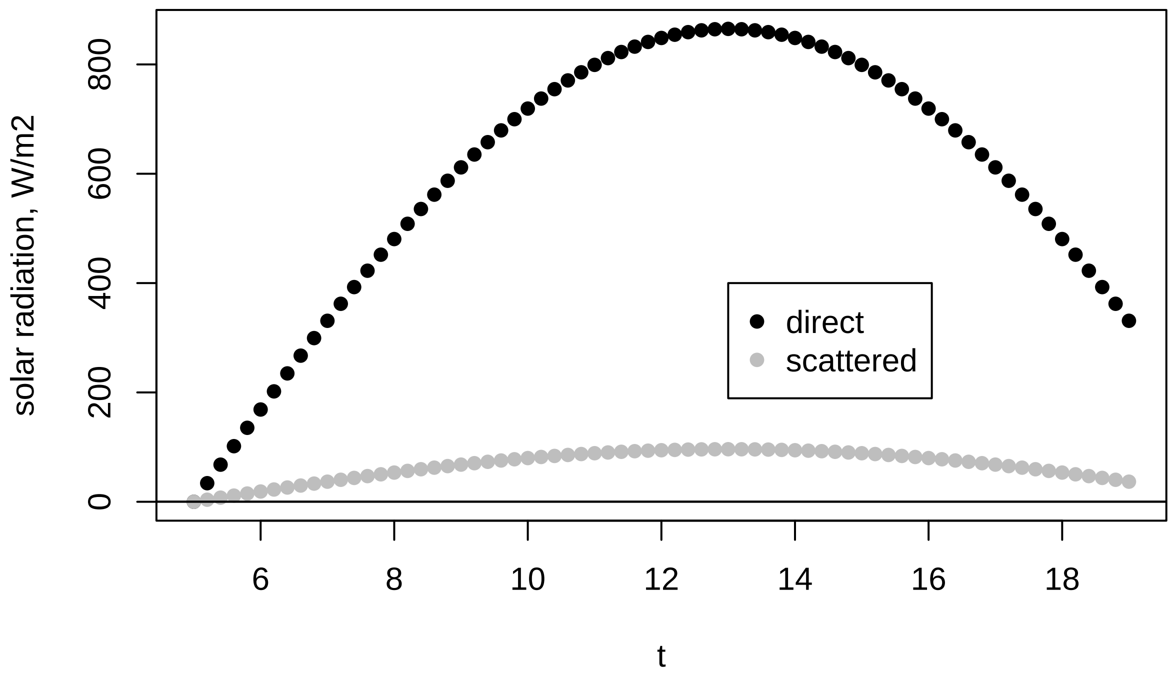


```
A_sun <- beta * A
A_shade <- (1 - beta) * A
A_skin_sun <- A_sun * (1 - f_hair)
A_skin_shade <- A_shade * (1 - f_hair)
A_hair_sun <- A_sun * f_hair
A_hair_shade <- A_shade * f_hair
#plot(A_skin_sun + A_skin_shade + A_hair_sun + A_hair_shade)
#abline(h = A)

Q_env <- A_skin_sun * Q_gain_dors_skin + A_skin_shade * Q_gain_vent_skin + A_hair_sun * Q_gain_dors_coa
```

```r
BMR <- 3.39 * M^0.75
C_mov <- 6.03 * M^0.7 + 10.7 * VELs * M^0.68 # cost of locomotion, W
Q_met <- 0.9 * (3.39 * M^0.75 + 10.7 * VELs * M^0.68)
#Q_met <- 0.9 * (6.03 * M ^ 0.7 + 10.7 * VELs * M ^ 0.68)

Q_load <- Q_met + Q_env
plot(t, Q_load, pch = 3, cex = 0.75, xlim = c(6, 18), ylim = c(-200, 1200)
     , ylab = 'W/m^2', xlab = 'hour of day')
Q_sweat <- 500 * A
abline(h = Q_sweat, lty = 'dotted')
legend(14, 300, legend = c('production', 'dissipation'), pch = c(3, NA),
  lty = c(NA, 'dotted')
)
```

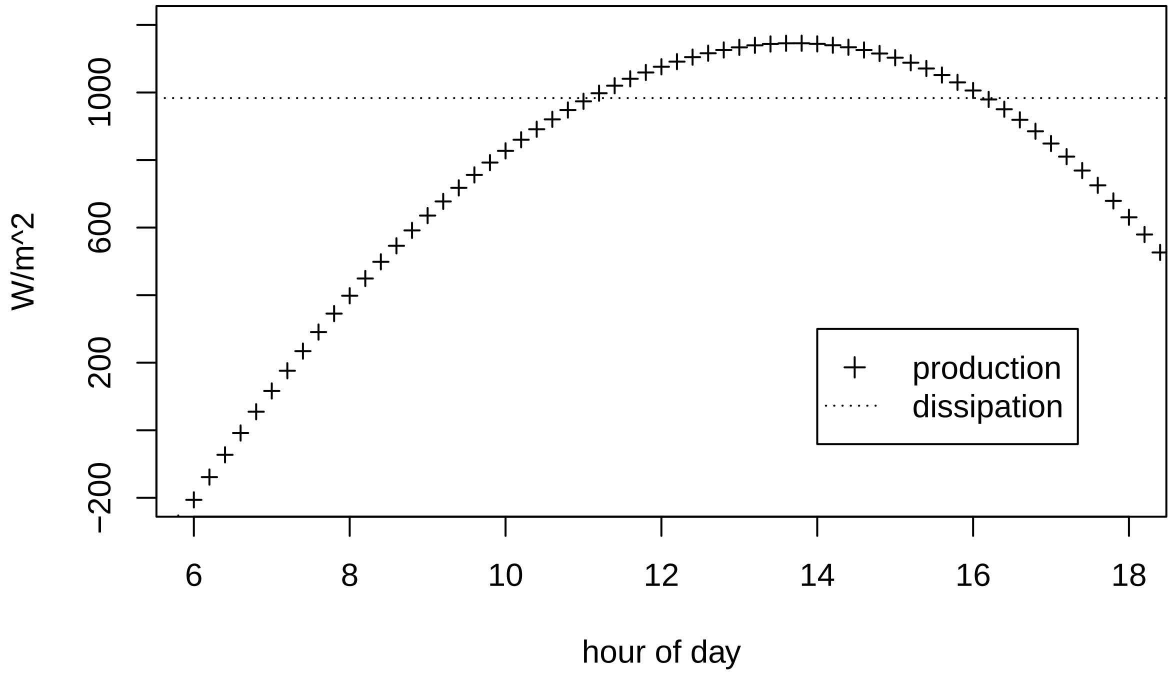


Note that the Ruxton and Wilkinson calculation does not explicitly compute the capacity for evaporative dissipation but rather plots the maximum possible dissipation. So where the production exceeds dissipation, core temperature would be expected to rise.

### Homotherm simulation

Set the environment and human parameters, here for *Homo sapiens* from Ruxton and Wiklinson's (2011a) Table 1 to mimic males of their Figure 1. Note that core temperature rise and the Q10 effect of this was not accounted for in their calculations. Setting Q10 to 1 and/or not allowing core temperature to rise or be exceeded in the simulation will give different results under high heat load.

```r
# environment
RHs <- rep(20, length(TAs))
ABSSB <- (1 - r) # substrate absorptivity, -

# person parameters
MASS <- M
QMETAB_REST <- Q_met
AREA <- A
MASSFRACs <- c(0.0761, 0.501, 0.049, 0.162)
AREAFRACs <- c(0.08291887, 0.32698460, 0.11025155, 0.18479669)
REFLD <- rep(1 - a, 4)
REFLV <- REFLD
INSDEPDs <- c(0.01, 0.0001, 0.0001, 0.0001) # fur depth, dorsal (m)
INSDEPVs <- c(0, 0.0001, 0.0001, 0.0001) # fur depth, ventral
SHAPE_Bs <- c(1.6, 2, 14, 7.9)
shapes <- GET_SHAPES(MASSs = MASS * MASSFRACs,
                     AREA = AREA,
                     SHAPE_Bs = SHAPE_Bs,
                     SHAPE_Bs.min = SHAPE_Bs,
                     SHAPE_Bs.max = SHAPE_Bs)
shapes$AREA_out
```

```
## [1] 1.912458
```

```r
SHAPE_Bs <- shapes$SHAPE_Bs
PJOINs <- shapes$PJOINs
HEIGHT <- (A / (0.00718 * MASS ^ 0.425)) ^ (1 / 0.725)
plot_human(MASS = MASS,
           SHAPE_Bs = SHAPE_Bs,
           PJOINs = PJOINs,
           HEIGHT = HEIGHT,
           INSDEPDs = INSDEPDs,
           INSDEPVs = INSDEPVs,)
```

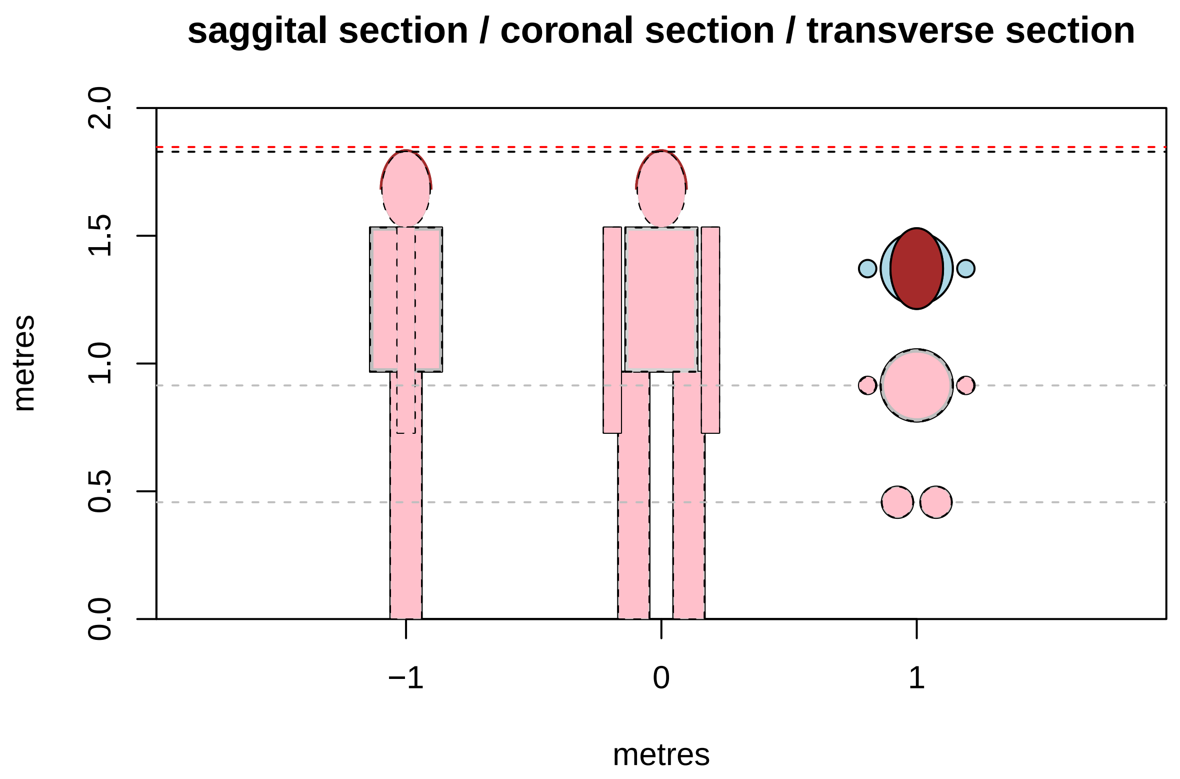


```
## [1] 1.828699
```

```
PCTWET_MAXs <- rep(1, 4)
Q10 <- 2
PCTBAREVAPs <- c(60, 90, 90, 90)
TC_RESTs <- rep(37, 4)
TC_MAXs <- rep(38, 4)
MAXSWEAT <- 500 / 2.26e6 * 3600 # L/h/m^2 (close to default of 0.75)
EXCEED.TCMAX <- TRUE
```

Run HomoTherm simulation and plot in comparison with Ruxton and Wilkinson model.

```
HomoTherm.out <- HomoTherm_var(MASS = MASS,
                               QMETAB_REST = QMETAB_REST[1],
                               TC_RESTs = TC_RESTs,
                               TC_MAXs = TC_MAXs,
                               INSDEPDs = INSDEPDs,
                               INSDEPVs = INSDEPVs,
                               SHAPE_Bs = SHAPE_Bs,
                               PJOINs = PJOINs,
                               REFLD = REFLD,
                               REFLV = REFLV,
                               TAs = TAs,
                               TSKYs = TSKYs,
                               TGRDs = TGRDs,
```

```
                                              RHs = RHs,
                                              VELs = VELs,
                                              QSOLRs = QSOLRs,
                                              PCTBAREVAPs = PCTBAREVAPs,
                                              MAXSWEAT = MAXSWEAT,
                                              EXCEED.TCMAX = EXCEED.TCMAX)
balance <- HomoTherm.out$balance
Q_load_NMR <- Q_met + balance$QSLR + balance$QRAD_IN - balance$QRAD_OUT + balance$QCONV_RESP + balance$
Q_load_NMR <- balance$QMETAB + balance$QSLR + balance$QRAD_IN - balance$QRAD_OUT + balance$QCONV_RESP +
plot(t, Q_load_NMR, xlim = c(6, 18), ylim = c(-200, 1200),
     ylab = 'W/m^2', xlab = 'hour of day')
points(t, Q_load, pch = 3, cex = 0.75)
abline(h = Q_sweat, lty = 'dotted')
points(t, -balance$QEVAP_CUT, type = 'l', lty = 2, col = 'blue')
legend(12, 350, legend = c('production R&W2011', 'dissipation R&W2011',
                           'production Homotherm', 'evaporation Homotherm'),
       pch = c(3, NA, 1, NA), lty = c(NA, 'dotted', NA, 'dashed'),
       col = c(1, 1, 1, 'blue'))
```

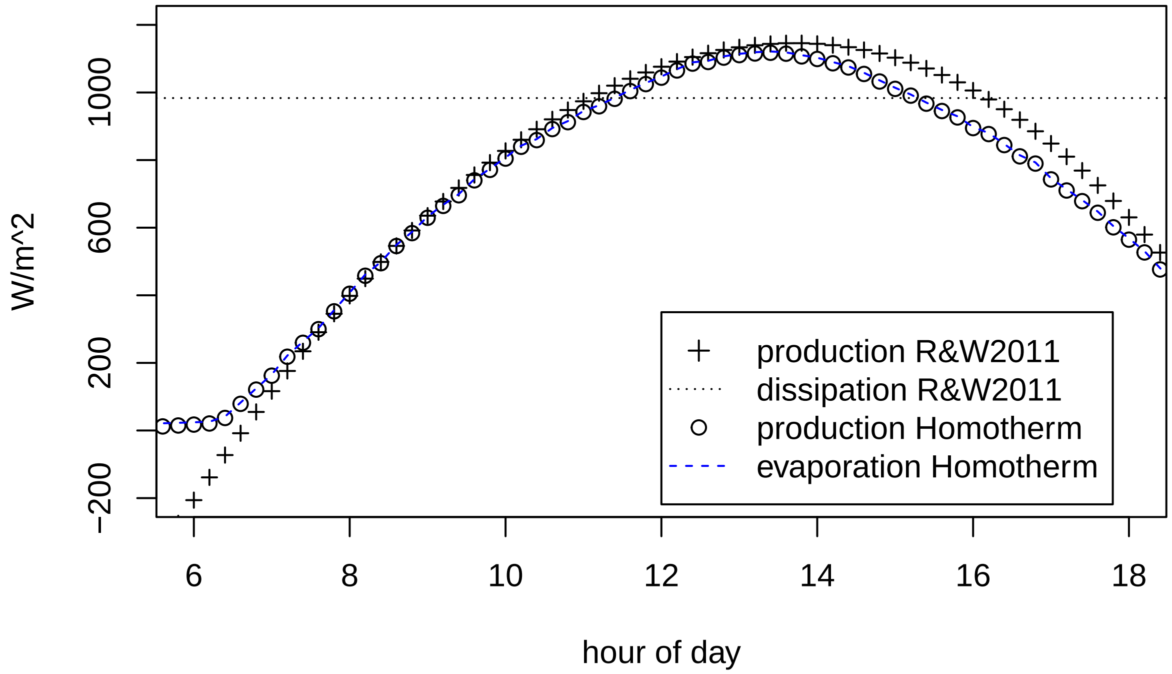


Note that the HomoTherm calculation includes the net heat production and required dissipation. The HomoTherm model additionally computes the required production in cold environments, hence the value of heat 'production' computed by the HomoTherm model is never negative.

In this simulation the parameters resulted in sufficient dissipation to match production over all environments, unlike the Ruxton and Wilkinson model, despite the same maximum sweating limit being used (i.e., MAXSWEAT <- 500 J/s/m^2 / 2.26e6 J/L * 3600 s/h, which is 0.8 L/h). Given the close match between

predictions of the HomoTherm model to human thermal responses generally, this implies that the simplifications of the Ruxton and Wilkinson approach led to an underestimation of the thermal loads tolerable by a hominin.

## References


Dávid-Barrett, T., & Dunbar, R. I. M. (2016). Bipedality and hair loss in human evolution revisited: The impact of altitude and activity scheduling. Journal of Human Evolution, 94, 72–82. https://doi.org/10.1016/j.jhevol.2016.02.006

Ruxton, G. D., & Wilkinson, D. M. (2011a). Thermoregulation and endurance running in extinct hominins: Wheeler's models revisited. Journal of Human Evolution, 61(2), 169–175. https://doi.org/10.1016/j.jhevol.2011.02.012

Ruxton, G. D., & Wilkinson, D. M. (2011b). Avoidance of overheating and selection for both hair loss and bipedality in hominins. Proceedings of the National Academy of Sciences, 108(52), 20965–20969. https://doi.org/10.1073/pnas.1113915108

Wheeler, P. E. (1991). The thermoregulatory advantages of hominid bipedalism in open equatorial environments: The contribution of increased convective heat loss and cutaneous evaporative cooling. Journal of Human Evolution, 21(2), 107–115. https://doi.org/10.1016/0047-2484(91)90002-d
