# Supplementary material for: HomoTherm: An Open‐Source Approach to Modelling Heat Exchange in Humans and Other Hominins in Diverse Environments
Source: Glob Chang Biol. 2026 Apr 1;32(4):e70830. doi: 10.1111/gcb.70830 (PMC13044332; doi:10.1111/gcb.70830)
Supplement: Supplementary file 14 — Appendix S14: gcb70830‐sup‐0014‐Appendix 14.pdf. [file GCB-32-e70830-s008.pdf]

Test of HomoTherm, MANMO and HHB models against Meade et al. 2023

Michael Kearney

2026-03-12

Overview

A test of the data reported in Meade et al. (2023) against the HomoTherm, MANMO (Myrup and Morgan, 1937), HHB (Human Heat Budget model; Vanos et al., 2023) and PHS (Predictive Heat Strain model; Malchaire et al. 2001). Meade et al. (2023) studied the response of young (19-31 yrs) and old (64-78) men and women to 9 hours at 41 degrees C and 9% relative humidity, reporting whole body heat exchange, body core and skin temperatures. A Snellen air calorimeter was used to measure dry heat and evaporative exchange in the first three hours. Individuals were measured while seated and resting, wearing light clothing (sandals, shorts, & light top for female participants; male participants did not wear a shirt.

Load the libraries and data

```
library(NicheMapR)
localpath <- 'c:/Users/mrke/Dropbox/Current Research Projects/mammal_projects/manmo analysis/'
source(paste0(localpath, '/code/MANMO/MANMO_R.R')) # the MANMO function
source(paste0(localpath, '/code/MANMO/run.MANMO.R'))
source(paste0(localpath, '/code/HHB/HHB.R'))
source(paste0(localpath, '/code/HHB/run_HHB.R'))
source(paste0(localpath, 'code/PHS/calcIso7933_Tcl.R'))
```

Load the Meade et al. observations

```
Fig1a_young <- read.csv('c:/Users/mrke/Dropbox/Current Research Projects/mammal_projects/manmo analysis,
Fig1a_older <- read.csv('c:/Users/mrke/Dropbox/Current Research Projects/mammal_projects/manmo analysis,
Fig1b_young <- read.csv('c:/Users/mrke/Dropbox/Current Research Projects/mammal_projects/manmo analysis,
Fig1b_older <- read.csv('c:/Users/mrke/Dropbox/Current Research Projects/mammal_projects/manmo analysis,
Fig2a_young <- read.csv('c:/Users/mrke/Dropbox/Current Research Projects/mammal_projects/manmo analysis,
Fig2a_older <- read.csv('c:/Users/mrke/Dropbox/Current Research Projects/mammal_projects/manmo analysis,
Fig2b_young <- read.csv('c:/Users/mrke/Dropbox/Current Research Projects/mammal_projects/manmo analysis,
Fig2b_older <- read.csv('c:/Users/mrke/Dropbox/Current Research Projects/mammal_projects/manmo analysis,
```

Environmental conditions

Set up environmental conditions.

```r
# environmental variables
TA <- 40 # air temperature, deg C
RH <- 9 # relative humidity, %
VEL <- 0.1 # wind speeds, from ft/min to m/s
```

Simulate two ages

First set up human parameters.

```r
# person parameters
MASSs <- c(66.3, 75.8) # MASS, kg
HEIGHTs <- c(169, 170) # height, cm
AREAs <- 0.00718 * MASSs ^ 0.425 * HEIGHTs ^ 0.725 # DuBois area, m2
INSDEPDs <- c(0.01, 0.001, 0.001, 0) # fur depth, dorsal (m)
INSDEPVs <- c(0, 0.0, 0.0, 0) # fur depth, ventral (m)
PCTBAREVAPs <- rep(70, 4)
MASSFRACs <- c(0.0761, 0.501, 0.049, 0.162)
QMETAB_REST_young <- 34.8 * 4184 / 3600 * AREAs[1] * 1.30 # basal metabolic rate, W
QMETAB_REST_older <- 34.8 * 4184 / 3600 * AREAs[2] * 1.25 # basal metabolic rate, W
TC_RESTs <- rep(36.8, 4)

SHAPE_Bs_young <- c(1.6, 2, 12, 7.0)


shapes_young <- GET_SHAPES(MASSs = MASSs[1] * MASSFRACs,
                     AREA = AREAs[2],
                     SHAPE_Bs = SHAPE_Bs_young,
                     SHAPE_Bs.min = c(1.6, 1.2, 6, 5),
                     SHAPE_Bs.max = c(1.6, 2, 10, 7.0))

SHAPE_Bs_older <- c(1.6, 1.6, 12, 7.0)

shapes_older <- GET_SHAPES(MASSs = MASSs[2] * MASSFRACs,
                     AREA = AREAs[2],
                     SHAPE_Bs = SHAPE_Bs_older,
                     SHAPE_Bs.min = c(1.6, 1.2, 6, 5),
                     SHAPE_Bs.max = c(1.6, 1.7, 10, 7.0))

plot_human(MASS = MASSs[1],
           HEIGHT = HEIGHTs[1],
           INSDEPDs = INSDEPDs,
           INSDEPVs = INSDEPVs,
           SHAPE_Bs = shapes_young$SHAPE_Bs)
```

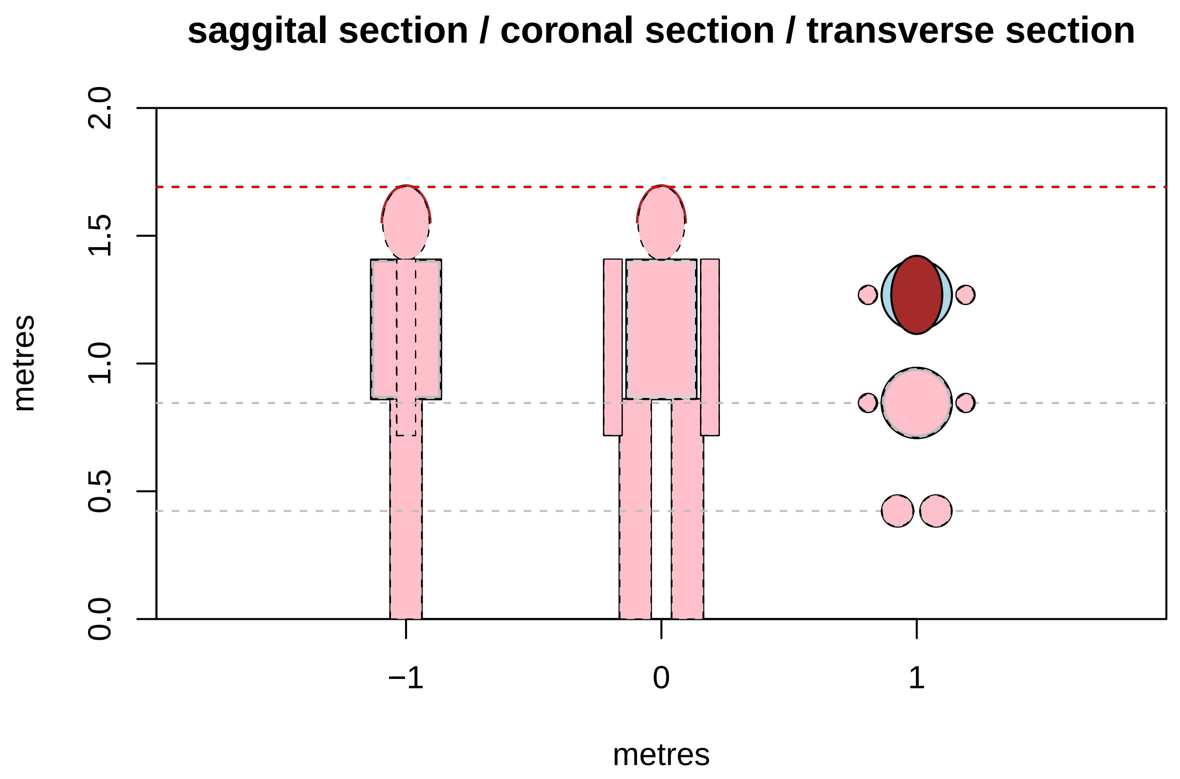


```
## [1] 1.691539
```

```
plot_human(MASS = MASSs[2],
           HEIGHT = HEIGHTs[2],
           INSDEPDs = INSDEPDs,
           INSDEPVs = INSDEPVs,
           SHAPE_Bs = shapes_older$SHAPE_Bs)
```

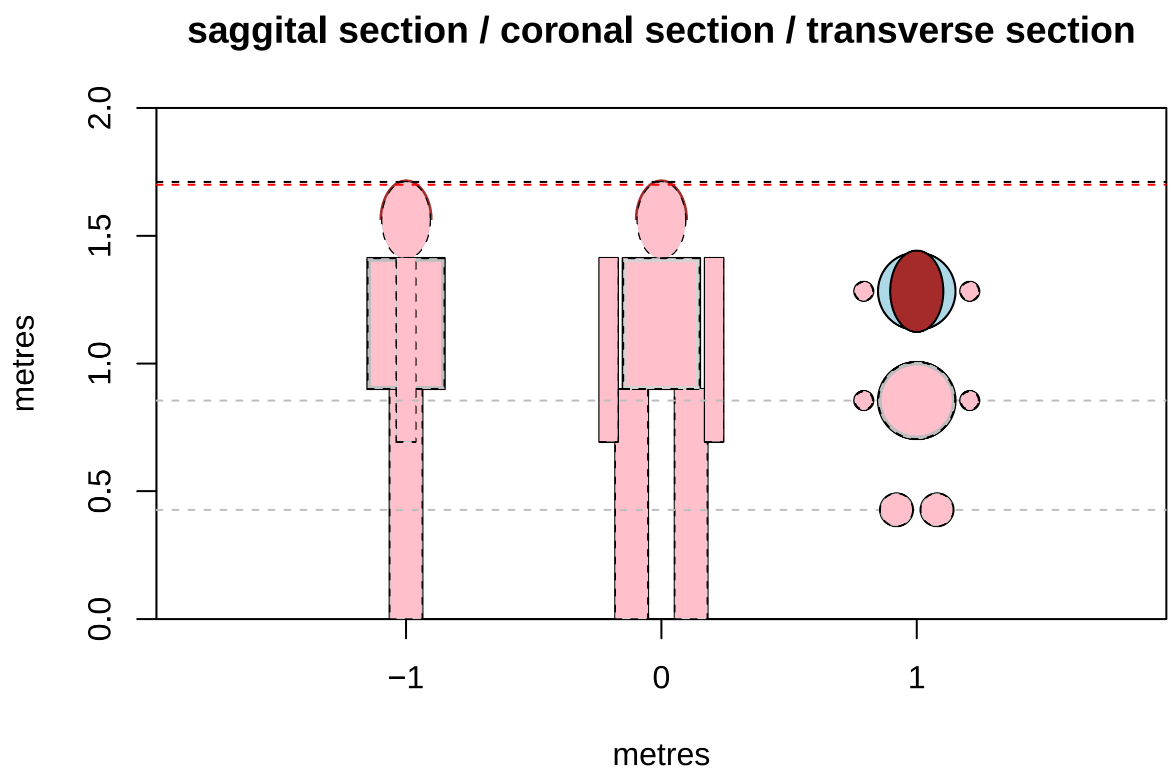


```
## [1] 1.710402
```

Run the simulations.

```
# run HomoTherm simulation
HomoTherm.young <- HomoTherm(MASS = MASSs[1],
                             QMETAB_REST = QMETAB_REST_young,
                             TC_RESTs = TC_RESTs,
                             SHAPE_Bs = shapes_young$SHAPE_Bs,
                             #KFLESH_INCs = rep(0.05, 4),
                             PCTWET_INCs = rep(0.1, 4),
                             #TC_INCs = rep(0.05, 4),
                             #KFLESH_MAXs = rep(3, 4),
                             INSDEPDs = INSDEPDs,
                             INSDEPVs = INSDEPVs,
                             PCTBAREVAPs = PCTBAREVAPs,
                             TA = TA,
                             TSKY = TA,
                             TGRD = TA,
                             RH = RH,
                             VEL = VEL)
balance_young <- as.data.frame(t(HomoTherm.young$balance))

HomoTherm.older <- HomoTherm(MASS = MASSs[2],
                             QMETAB_REST = QMETAB_REST_older,
```

```
                              SHAPE_Bs = shapes_older$SHAPE_Bs,
                              #KFLESH_INCs = rep(0.05, 4),
                              PCTWET_INCs = rep(0.1, 4),
                              TC_INCs = rep(0.06, 4),
                              #KFLESH_MAXs = rep(1.5, 4),
                              TC_RESTs = TC_RESTs,
                              INSDEPDs = INSDEPDs,
                              INSDEPVs = INSDEPVs,
                              PCTBAREVAPs = PCTBAREVAPs,
                              TA = TA,
                              TSKY = TA,
                              TGRD = TA,
                              RH = RH,
                              VEL = VEL)
balance_older <- as.data.frame(t(HomoTherm.older$balance))

# run MANMO simulations
G_m.G2s <- balance_young$QMETAB * 4.184 * 1000 / 3600 / AREAs[1] # used for MANMO
clo <- 0.1
MANMO.output <- run.MANMO(W = 0.01,
                          Ht.H4 = HEIGHTs[1],
                          Wt.W4 = MASSs[1],
                          D3 = c(mean(INSDEPDs[2:4]), rep(1e-10, 3)),
                          Maximum.SR = 1000 / 60 / AREAs[1],
                          G_m.G2s = G_m.G2s,
                          CLO.C4 = clo,
                          CLO.mode = 0,
                          TAs = TA,
                          TSKYs = TA,
                          TGNDs = TA,
                          RH.H2s = RH / 100,
                          VELs = VEL)
MANMO.young <- MANMO.output

G_m.G2s <- balance_older$QMETAB * 4.184 * 1000 / 3600 / AREAs[2] # used for MANMO
clo <- 0.1
MANMO.output <- run.MANMO(W = 0.01,
                          Ht.H4 = HEIGHTs[2],
                          Wt.W4 = MASSs[2],
                          D3 = c(mean(INSDEPDs[2:4]), rep(1e-10, 3)),
                          Maximum.SR = 1000 / 60 / AREAs[2],
                          G_m.G2s = G_m.G2s,
                          CLO.C4 = clo,
                          CLO.mode = 0,
                          TAs = TA,
                          TSKYs = TA,
                          TGNDs = TA,
                          RH.H2s = RH / 100,
                          VELs = VEL)
MANMO.older <- MANMO.output

HHB.young <- run_HHB(exp_time = 6,
                     AD = AREAs[1],
```

```
                     M =  balance_young$QMETAB,
                     Tsk_C = 35,
                     Emm_sk = 0.98,
                     Ar_AD = 0.7,
                     Icl = clo,
                     Ta_C = TA,
                     humidity = RH,
                     Av_ms = VEL,
                     mrt_C = TA,
                     deltaT = 13 - 36.8, # default TC_REST 36.8
                     Mass = MASSs[1],
                     Smax = 1.5,
                     Re_cl = 0,
                     wmax_condition = 1)
HHB.young <- as.data.frame(t(HHB.young))

HHB.older <- run_HHB(exp_time = 6,
                     AD = AREAs[2],
                     M =  balance_older$QMETAB,
                     Tsk_C = 35,
                     Emm_sk = 0.98,
                     Ar_AD = 0.7,
                     Icl = clo,
                     Ta_C = TA,
                     humidity = RH,
                     Av_ms = VEL,
                     mrt_C = TA,
                     deltaT = 13 - 36.8, # default TC_REST 36.8
                     Mass = MASSs[2],
                     Smax = 1.5,
                     Re_cl = 0,
                     wmax_condition = 1)
HHB.older <- as.data.frame(t(HHB.older))

# Iso7933

Iso7933.young <- calcIso7933_Tcl(accl = 0,
                                 posture = 1,
                                 Ta = TA,
                                 Pa = WETAIR(db = TA, rh = RH)$e / 1000,
                                 Tr = TA,
                                 Va = VEL,
                                 Tsk = TA,
                                 Met = balance_young$QMETAB / AREAs[1],
                                 Icl = clo,
                                 weight = MASSs[1],
                                 height = HEIGHTs[1] / 100,
                                 Adu = AREAs[1],
                                 Tre = 36.8, # default TC_REST 36.8
                                 Tcr = 36.8, # default TC_REST 36.8
                                 SWp = 0.5)

Iso7933.older <- calcIso7933_Tcl(accl = 0,
```

```
                                  posture = 1,
                                  Ta = TA,
                                  Pa = WETAIR(db = TA, rh = RH)$e / 1000,
                                  Tr = TA,
                                  Va = VEL,
                                  Tsk = TA,
                                  Met = balance_older$QMETAB / AREAs[2],
                                  Icl = clo,
                                  weight = MASSs[2],
                                  height = HEIGHTs[2] / 100,
                                  Adu = AREAs[2],
                                  Tre = 36.8, # default TC_REST 36.8
                                  Tcr = 36.8, # default TC_REST 36.8
                                  SWp = 0.5)
```

Compare results against observations in figures 1 A & B and 2 & B in Meade et al. (2023).

```
par(mfrow = c(4, 2))
par(oma = c(4, 2, 2, 2) + 0.1) # margin spacing
par(mar = c(4, 4, 1, 1) + 0.1) # margin spacing
par(mgp = c(3, 1, 0) ) # margin spacing

plot(c(seq(0, 3), seq(6, 9)), Fig1a_young[, 1],
     ylim = c(40, 140), xlim = c(0, 9),
          ylab = "W/m^2",
          xlab = "hours of exposure",
          col = 'red', pch = 16, cex = 1.25, main = 'whole-body heat gain (metabolic + dry)'
)
heat_gain_young <- (balance_young$QMETAB + balance_young$QRAD_IN -
                      balance_young$QRAD_OUT + balance_young$QCONV -
                      balance_young$QCONV_RESP) / AREAs[1]
abline(h = heat_gain_young)
heat_gain_young_MANMO <- (MANMO.young$M_m.M + MANMO.young$I_m.I +
                            MANMO.young$H_m.H) / AREAs[1]
abline(h = heat_gain_young_MANMO, lty = 2)
abline(h = (-HHB.young$Dry_Heat_Loss + balance_young$QMETAB) / AREAs[1],
       col = 'orange')
abline(h = (balance_young$QMETAB - Iso7933.young$Dry) / AREAs[1], col = 'darkgreen', lty = 2)
legend(0, 140, legend = c('observed', 'MANMO', 'HomoTherm', 'HHB', 'PHS'),
       col = c('red', 'black', 'black', 'orange', 'darkgreen'), lty = c(NA, 2, 1, 1, 1, 2),
       pch  = c(16, rep(NA, 3)),
       bty = 'n', horiz = FALSE, ncol = 2, cex = 0.8)
mtext(side = 3, "young", outer = TRUE, adj = 0.25, padj = -0.2)

plot(c(seq(0, 3), seq(6, 9)), Fig1a_older[, 1],
     ylim = c(40, 140), xlim = c(0, 9),
          ylab = "W/m^2",
          xlab = "hours of exposure",
          col = 'red', pch = 16, cex = 1.25,
     main = 'whole-body heat gain (metabolic + dry)'
)
heat_gain_older <- (balance_older$QMETAB + balance_older$QRAD_IN -
                      balance_older$QRAD_OUT + balance_older$QCONV -
```

```
                      balance_older$QCONV_RESP) / AREAs[2]
abline(h = heat_gain_older)
heat_gain_older_MANMO <- (MANMO.older$M_m.M + MANMO.older$I_m.I + MANMO.older$H_m.H) / AREAs[2]
abline(h = heat_gain_older_MANMO, lty = 2)
abline(h = (-HHB.older$Dry_Heat_Loss + balance_older$QMETAB) / AREAs[2],
       col = 'orange')
abline(h = (balance_older$QMETAB - Iso7933.older$Dry) / AREAs[2], col = 'darkgreen', lty = 2)
mtext(side = 3, "older", outer = TRUE, adj = 0.75, padj = -0.2)

plot(c(seq(0, 3), seq(6, 9)), Fig1b_young[, 1],
     ylim = c(0, 120), xlim = c(0, 9),
          ylab = "W/m^2",
          xlab = "hours of exposure",
          col = 'red', pch = 16, cex = 1.25, main = 'whole-body heat loss (evaporative)'
)
heat_loss_young <- (balance_young$QEVAP_RESP + balance_young$QEVAP_CUT) / AREAs[1]
abline(h = heat_gain_young)
abline(h = -MANMO.young$E_m.E / AREAs[1], lty = 2)
abline(h = HHB.young$Ereq / AREAs[1], col = 'orange')
abline(h = (Iso7933.young$Eres + Iso7933.young$SWp) / AREAs[1], col = 'darkgreen', lty = 2)

plot(c(seq(0, 3), seq(6, 9)), Fig1b_older[, 1],
     ylim = c(0, 120), xlim = c(0, 9),
          ylab = "W/m^2",
          xlab = "hours of exposure",
          col = 'red', pch = 16, cex = 1.25, main = 'whole-body heat loss (evaporative)'
)
heat_loss_older <- (balance_older$QEVAP_RESP + balance_older$QEVAP_CUT) / AREAs[2]
abline(h = heat_gain_older)
abline(h = -MANMO.older$E_m.E / AREAs[2], lty = 2)
abline(h = HHB.older$Ereq / AREAs[2], col = 'orange')
abline(h = (Iso7933.older$Eres + Iso7933.older$SWp) / AREAs[2], col = 'darkgreen', lty = 2)

plot(seq(0, 9), Fig2a_young[, 1],
     ylim = c(36.2, 38.6), xlim = c(0, 9),
          ylab = "deg C",
          xlab = "hours of exposure",
          col = 'red', pch = 16, cex = 1.25, main = 'body core temperature (rectal)'
)
abline(h = balance_young$T_CORE)
abline(h = 37, col = 'orange')
abline(h = 37, lty = 2)
abline(h = Iso7933.young$Tre, col = 'darkgreen', lty = 2)

plot(seq(0, 9), Fig2a_older[, 1],
     ylim = c(36.2, 38.6), xlim = c(0, 9),
          ylab = "deg C",
          xlab = "hours of exposure",
          col = 'red', pch = 16, cex = 1.25, main = 'body core temperature (rectal)'
)
abline(h = balance_older$T_CORE)
abline(h = 37, col = 'orange')
abline(h = 37, lty = 2)
```

```r
abline(h = Iso7933.older$Tre, col = 'darkgreen', lty = 2)

plot(seq(0, 9), Fig2b_young[, 1],
     ylim = c(30, 39), xlim = c(0, 9),
          ylab = "deg C",
          xlab = "hours of exposure",
          col = 'red', pch = 16, cex = 1.25, main = 'mean skin temperature'
)
abline(h = balance_young$T_SKIN)
abline(h = 35, col = 'orange')
abline(h = MANMO.young$Tskin, lty = 2)
abline(h = Iso7933.young$Tsk, col = 'darkgreen', lty = 2)

plot(seq(0, 9), Fig2b_older[, 1],
     ylim = c(30, 39), xlim = c(0, 9),
          ylab = "deg C",
          xlab = "hours of exposure",
          col = 'red', pch = 16, cex = 1.25, main = 'mean skin temperature'
)
abline(h = balance_older$T_CORE)
abline(h = 35, col = 'orange')
abline(h = MANMO.older$Tskin, lty = 2)
abline(h = Iso7933.older$Tsk, col = 'darkgreen', lty = 2)
```

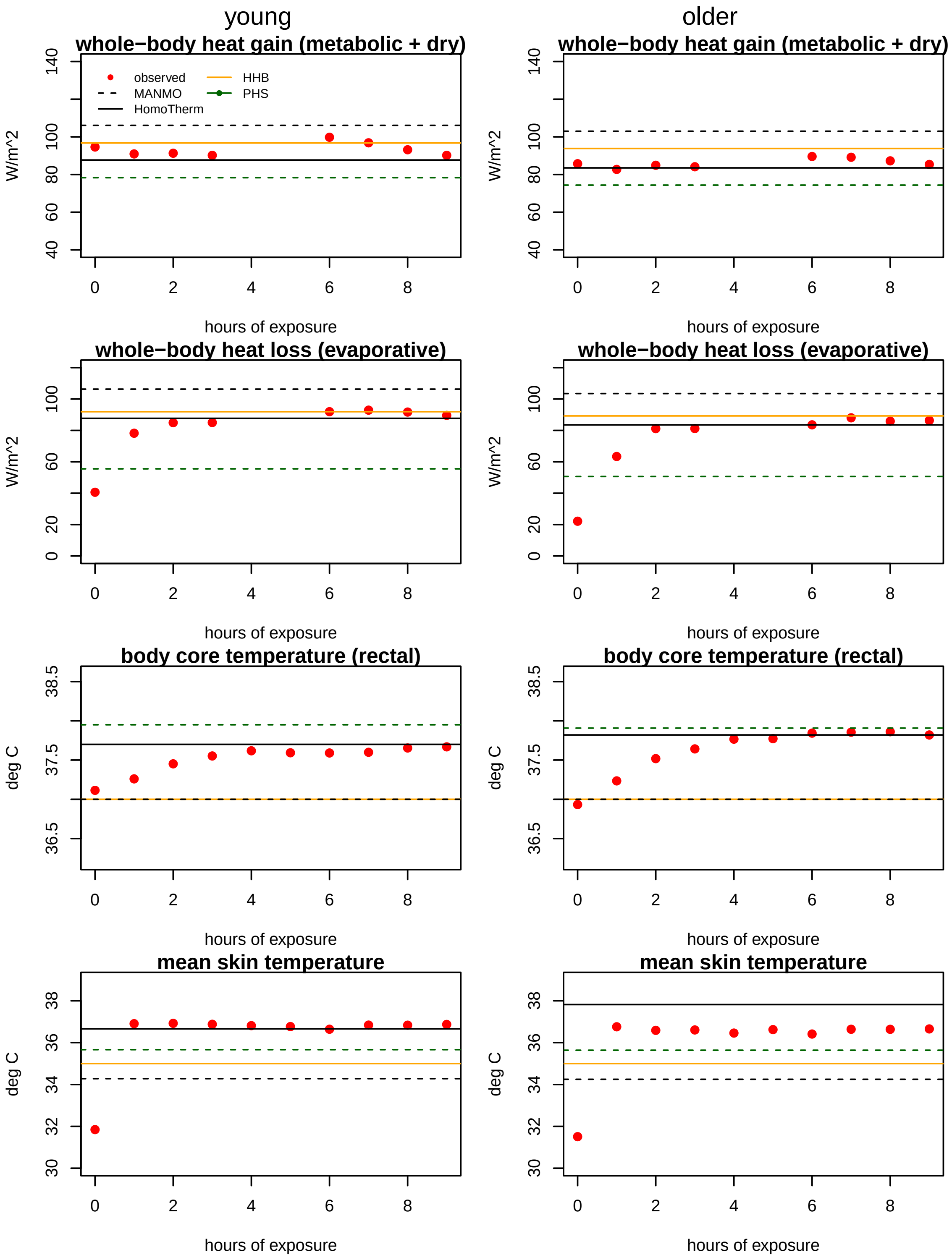
young
older
whole−body heat gain (metabolic + dry)
observed
MANMO
HomoTherm
HHB
PHS
W/m^2
hours of exposure
whole−body heat loss (evaporative)
body core temperature (rectal)
deg C
mean skin temperature

References

Malchaire, J., Piette, A., Kampmann, B., Mehnert, P., Gebhardt, H., Havenith, G., den Hartog, E., Holmer, I., Parsons, K., Alfano, G., & Griefahn, B. (2001). Development and validation of the predicted heat strain model. The Annals of Occupational Hygiene, 45(2), 123–135. https://doi.org/10.1016/S0003-4878(00)00030-2

Meade, R. D., Notley, S. R., Akerman, A. P., McGarr, G. W., Richards, B. J., McCourt, E. R., King, K. E., McCormick, J. J., Boulay, P., Sigal, R. J., & Kenny, G. P. (2023). Physiological responses to 9 hours of heat exposure in young and older adults. Part I: Body temperature and hemodynamic regulation. Journal of Applied Physiology, 135(3), 673–687. https://doi.org/10.1152/japplphysiol.00227.2023

Myrup, L. O., and D. L. Morgan. 1972. Numerical model of the urban atmosphere. Volume I The city-surface interface. University of California, Davis.

Vanos, J., G. Guzman-Echavarria, J. W. Baldwin, C. Bongers, K. L. Ebi, and O. Jay. 2023. A physiological approach for assessing human survivability and liveability to heat in a changing climate. Nature Communications 14:7653.
